# Assessing the Impact of a Viral Infection on the Expression of Transposable Elements in the Cabbage Looper Moth (*Trichoplusia ni*)

**DOI:** 10.1093/gbe/evab231

**Published:** 2021-10-06

**Authors:** Héloïse Muller, Vincent Loiseau, Sandra Guillier, Richard Cordaux, Clément Gilbert

**Affiliations:** 1 Universite Paris Saclay, CNRS, IRD, UMR Evolution, Genomes, Comportement et Ecologie, Gif-sur-Yvette, France; 2 Laboratoire Ecologie et Biologie des Interactions, Equipe Ecologie Evolution Symbiose, Universite de Poitiers, CNRS, France

**Keywords:** mobile elements, dsDNA virus, AcMNPV, horizontal transfer, Lepidoptera

## Abstract

Most studies of stress-induced transposable element (TE) expression have so far focused on abiotic sources of stress. Here, we analyzed the impact of an infection by the AcMNPV baculovirus on TE expression in a cell line (Tnms42) and midgut tissues of the cabbage looper moth (*Trichoplusia ni*). We find that a large fraction of TE families (576/636 in Tnms42 cells and 503/612 in midgut) is lowly expressed or not expressed at all [≤ 4 transcripts per million (TPM)] in the uninfected condition (median TPM of 0.37 in Tnms42 and 0.46 in midgut cells). In the infected condition, a total of 62 and 187 TE families were differentially expressed (DE) in midgut and Tnms42 cells, respectively, with more up- (46) than downregulated (16) TE families in the former and as many up- (91) as downregulated (96) TE families in the latter. Expression log2 fold changes of DE TE families varied from −4.95 to 9.11 in Tnms42 cells and from −4.28 to 7.66 in midgut. Large variations in expression profiles of DE TEs were observed depending on the type of cells and on time after infection. Overall, the impact of AcMNPV on TE expression in *T. ni* is moderate but potentially sufficient to affect TE activity and genome architecture. Interestingly, one host-derived TE integrated into AcMNPV genomes is highly expressed in infected Tnms42 cells. This result shows that virus-borne TEs can be expressed, further suggesting that they may be able to transpose and that viruses may act as vectors of horizontal transfer of TEs in insects.

## Introduction

TEs are selfish genetic elements able to move in the genome of their hosts and that account for a large fraction of eukaryotic genomes (Schnable et al. 2009; Sotero-Caio et al. 2017). Based on their ability to transpose, TEs are classified into two categories: TEs that move through an RNA intermediate are class I TEs and those moving through a DNA intermediate are class II TEs (Wicker et al. 2007). The raw genetic material deposited by each new transposition event has sometimes been recycled during evolution, fueling genomic novelty and adaptation (Arkhipova 2018; Bourque et al. 2018). While domestication of many TE-coding sequences has been reported (Volff 2006), most co-option events involve TE regulatory sequences, which have sometimes led to profound changes into expression landscapes (Chuong et al. 2017). However, like many other mutation types, most transposition events are neutral or harmful and are thought to negatively impact host fitness (Brookfield and Badge 1997; Barrón et al. 2014; Mita and Boeke 2016). In response to the deleterious effects of TEs, several TE-repressing mechanisms have evolved in host genomes, such as DNA methylation, histone modifications, or posttranscriptional repression through the PIWI-interacting RNA pathway (Slotkin et al. 2007; Deniz et al. 2019). Thus, host–TE interactions are often referred to as an arms race and they often result in the complete extinction of TE families and degradation of TE copies that are eliminated from the genome with time, mainly due to the neutral evolution of most TE sequences (Le Rouzic et al. 2007; Blumenstiel 2019).

Typically, few TE families are expected to be transpositionally active in a genome, most of them being repressed and thus not expressed (Yoder et al. 1997; Zilberman et al. 2007). However, perturbations of genome stability, environmental changes, or infection can lead to a stress-mediated modulation of TE expression (Miousse et al. 2015). Several examples of TE activation due to environmental stress have been reported in plants, and this phenomenon appears to also occur in other eukaryotes such as yeasts, human, and other mammals, insects, and nematodes (Menees and Sandmeyer 1996, Van Meter et al. 2014; Voronova et al. 2014; Romero-Soriano and Garcia Guerreiro 2016; Zovoilis et al. 2016; Huang et al. 2017; Hummel et al. 2017; Ryan et al. 2017; Dubin et al. 2018). Such activation is often thought to be caused by epigenetic modifications or activation of transcription factors (Capy et al. 2000; Horváth et al. 2017). Interestingly, some TEs even bear a stress response element, that is, a regulatory sequence activated in response to a stress, enabling TEs to be upregulated in stressful conditions (Bucher et al. 2012; Casacuberta and González 2013). However, the impact of stress on TE expression appears to be hardly predictable. For instance, studies of stress-induced TE expression in *Drosophila* have shown that depending on cases, TEs can be upregulated, downregulated, or transiently upregulated before being downregulated in response to a stress (Horváth et al. 2017). The complexity of the interplay between stress and TE expression is likely due to several factors. First, the impact of stress on transcription varies along the genome, being seemingly higher in facultative heterochromatin, which is generally gene rich and poorer in TEs than in constitutive heterochromatin, which is generally associated with gene-poor, TE-rich regions (Trojer and Reinberg 2007; Saksouk et al. 2015). Consistently, the distribution of a TE family along the genome is often highly correlated to chromatin state (Lanciano and Mirouze 2018). Moreover, stress-induced TE activation can generate new copies in the genome via transposition. These new copies can bear *cis*-regulatory elements that can contribute to rewire the stress response network, in turn modulating the interaction between stress and TE expression during a stress (Cowley and Oakey 2013; Galindo-González et al. 2017). Finally, the epigenetic landscape influencing TE repression is variable between closely related species and even between populations of a single species (Barah et al. 2013; Niederhuth et al. 2016; Fouché et al. 2020).

In the study of eukaryotic TE response to stress, most efforts focused on plants. To our knowledge, few studies have investigated the impact of a biotic stress like a viral infection on TE expression in animals. A recent study reanalyzed transcriptomic data of several human and mouse cell lines infected by various viruses and found a genome-wide TE upregulation in host cells (Macchietto et al. 2020). This pattern was observed particularly near antiviral response genes and was common to all analyzed data sets, whatever the virus type, the host species or the cell type studied. The authors concluded that TE upregulation during a viral infection could be a common phenomenon in human and mouse. A second study analyzed the impact of the single-stranded RNA Sindbis virus (SINV) on *Drosophila simulans* and *Drosophilamelanogaster* flies (Roy et al. 2020). It was found that viral infection can modulate the piRNA and siRNA pathways known to be involved in TE expression control. In turn, a global decrease in TE transcript amounts was observed in *D. simulans* and *D. melanogaster* flies during the exponential phase of SINV replication. Overall, these studies suggest that viral infection can affect TE activity in animals.

Interestingly, several other studies reporting host TEs integrated in baculovirus genomes provide direct evidence that some TEs can be active during a viral infection (Fraser et al. 1985; Jehle et al. 1998; Gilbert et al. 2014, 2016; Loiseau et al. 2020). For example, Gilbert et al. (2016) found thousands of TE copies belonging to 13 TE superfamilies integrated in the genome of the AcMNPV baculovirus after the infection of noctuid moth larvae. They estimated that in these viral populations, 4.8% of AcMNPV genomes on average carried at least one host TE. Furthermore, long-read sequencing revealed that many TE copies were integrated in AcMNPV genomes as full-length copies, bearing all the components necessary to transpose (Loiseau et al. 2020). These studies clearly indicated that many class I and class II TEs are expressed and capable of actively transposing during infection by the AcMNPV baculovirus. They also raised several questions regarding the possible interaction between AcMNPV and host TEs. First, host TE expression has never been measured during infection by large dsDNA viruses. Thus, it is unknown whether the TEs found in viral genomes are expressed in the host genome in normal, noninfected conditions, or whether they are normally repressed but become activated or overexpressed in infected hosts. Whether TEs found in viral genomes during an infection are also those that are the most highly expressed in the host genome is also unknown. Furthermore, the influence of factors such as TE age, copy number, and position in the host genome on the level of host TE expression remains unclear. Finally, whether TE copies integrated into viral genomes are expressed during infection has never been assessed.

Here, we addressed these questions by reanalyzing two published time course RNA-seq data sets that were initially produced to measure variation in gene expression levels of the moth *Trichoplusia ni* in response to an infection by the AcMNPV baculovirus (Chen et al. 2013; Shrestha et al. 2018). These experiments were carried out in the Tnms42 cell line and in the midgut of *T. ni* fifth instar larvae. We found three times more DE TE families in Tnms42 cells than in *T. ni* midguts. One of the *T. ni* TE families previously found inserted in AcMNPV genomes (TFP3) was particularly overexpressed in infected Tnms42 cells compared to all other TE families, likely due to the expression of copies inserted into AcMNPV genomes. Overall, our study shows that infection by AcMNPV affects the expression of a moderate number of TE families in Tnms42 cells and *T. ni* midgut. It further reveals that TEs inserted in viral genomes can be transcribed, in agreement with the possible role viruses may play as vectors of horizontal transfer of TEs between insects.

## Results

### TE Landscape in *T. ni* Genomes

**Fig. 1.**
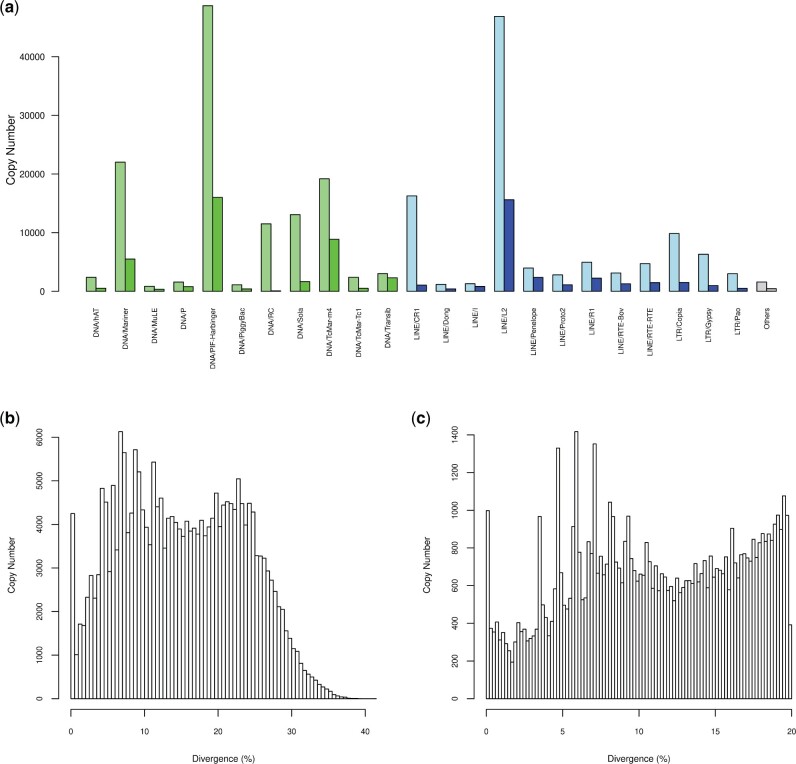
Transposable element landscape of the *T. ni* larva genome. (*a*) Copy number of the different TE superfamilies detected before filtering (light colors on left) and after filtering (bright colors on right). Class I TE superfamilies are in green, class II TE superfamilies are in blue, and superfamilies with low copy number (<115 copies) are in gray. (*b*) Histogram of observed TE copy nucleotide divergence to consensus for the nonfiltered 702 TE families. (c) Histogram of observed TE copy nucleotide divergence to consensus for the 614 TE families included in the study, after filtering.

We first annotated TE copies de novo in the two *T. ni* genomes used in this study [HighFive (Hi5) germ cell line and larva]. The TE landscape of the *T. ni* larva genome was not characterized in the original publication (Chen et al. 2019). Using RepeatMasker and a library of 847 TE consensus sequences obtained through various searches (see methods), we masked 231,670 copies (or TE fragments) that make up 8.66% of the *T.ni* larva genome. These copies were masked by 702 out of the 847 TE consensus. DNA TEs were the most abundant with 126,660 copies (54.7%), including 11,511 Helitron copies, followed by LINEs (85,814 copies, 37%) and LTRs (19,196 copies, 8,3%). The most abundant superfamilies were the class II DNA/PIF-Harbinger (48,702 copies), the class I LINE/L2 (46,890 copies), and the class II DNA/mariner (22,017 copies), which collectively accounted for half of all TE copies (fig. 1*a*). On the contrary, some superfamilies were less abundant, like DNA/MuLE (835 copies), DNA/PiggyBac (1,111 copies), or LINE/Dong (1,181 copies). The overall nucleotide divergence between copies and consensus sequences ranged from 0% to 41.3% (median 15%). A total of 3,103 copies (1.34%) were identical to their consensus (0% divergence).

To map RNA-seq reads on TE copies less fragmented than those produced by our automatic TE annotation procedure, we ran the tool “One Code to Find them All” (Bailly-Bechet et al. 2014). We used the options –unknown and –strict, filtering copies greater than 80 bp in length and with more than 80% identity with the consensus. The filtered TE landscape contains 66,683 copies masked by 612 TE consensus and making 8.25% of the *T.ni* larva genome. These copies contain 55.7% of DNA TEs, 39.9% of LINEs and 4.5% of LTRs (fig. 1). This filtering and aggregation step decreased the number of TE copies by a factor of 3.5 but the overall size of the resulting TE copies is relatively similar to the total size of the nonfiltered TE copies (28.8 vs. 27.5 Mb).

De novo TE annotation of the *T. ni* Hi5 germ cell line genome was previously done (Fu et al. 2018). However, to facilitate comparison between expression profiles of the *T. ni* cell line and midgut, we performed our own TE annotation of this genome using the same pipeline as the one used for the *T. ni* larva genome. We annotated 11.5% and 9.98% of the cell line genome as TEs before and after aggregating and filtering copies, respectively. This is similar to the figure obtained by Fu et al. when excluding SINEs (9.41%). We retained copies masked by 636 consensus sequences after filtering. The TE landscape was overall very similar to that of the larva genome, with 53.9% of DNA TEs, 37.1% of LINEs, and 9.0% of LTRs before filtering, versus 55.0% of DNA, 40% of LINE, and 5.0% of LTR after filtering.

### Genome-Wide TE Differential Expression during AcMNPV Infection of Tnms42 Cells

Reads produced by Chen et al. (2013) were mapped on the 75,680 TE copies annotated in the *T. ni* Hi5 genome and the differential expression was computed by TE consensus, which we consider here as each representing a separate family (636 TE families included in this analysis). Among the 636 TE families, 576 are considered as not or very lowly expressed in the mock condition [transcripts per million (TPM) < 4]. The median for TE expression in the mock is 0.37 TPM and the maximum is 65.6 TPM for Tni_Contig_13_Harbinger. Thus, few TE families are expressed in the mock condition in Tnms42 cells, with only two of them being highly expressed, that is, their average TPM is higher than 50.

**Fig. 2.**
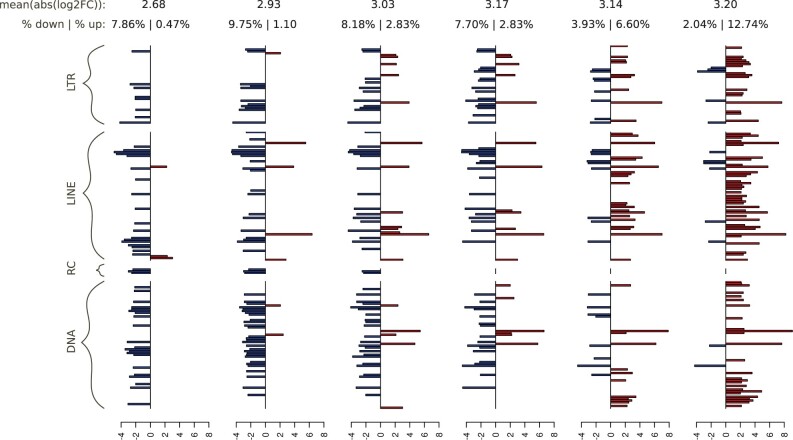
DE TE families in the Tnms42 cell line data set.. Only the 187 significant DE TE families with and absolute log2 fold change superior to 2 are considered. On top, the first line shows the average absolute log2FC at each time point, while the second line shows the percentage of TE families downregulated and upregulated at each time point. For each time point, log2FC is indicated in red for upregulated TE families or in blue for downregulated ones. The 187 DE TE family names can be found in supplementary table S1.

The number of DE TE families varied from 53 (8.3% of all TE families) to 94 (14.8%) depending on the time point during the course of the AcMNPV infection in *T. ni* cells (fig. 2 and supplementary table S1). When considering all time points together, a total of 187 TE families (29% of all families) were found to be DE during at least one time point. Overall, the strength of differential expression went from 30-fold decrease to 553-fold increase (log2 fold change = −4.95 to 9.11), with a median over all DE TE families and all time points at −2.01 log2 fold change and an average at 0.07. Among all 187 DE TE families, 91 and 96 were up- and downregulated during at least one time point, respectively, and one was alternatively down- and upregulated during the course of the experiment (table 1 and fig. 2). Among the 91 upregulated TE families, 8 were induced, that is, they showed no or very low expression in the mock (TPM < 4) and were expressed in at least one time point in the infected condition (TPM > 4), including two TE families (LINE/R1_8 and LINE/Proto_3) that became highly expressed (TPM ≥ 50). Among the 96 downregulated TE families, 7 can be considered repressed in at least one time point, that is, their TPM was higher than 4 in the mock and less than 4 in the infected condition. No repressed TE families were highly expressed in the mock (TPM ≥ 50). Altogether, these observations show that infection by AcMNPV moderately affects the expression of a substantial proportion of TE families in the *T. ni* Tnms42 cell line genome, with a similar number of up- and downregulated TE families.

**Table 1:** DE TE families in the Cell Line and Midgut Data Set for Each Class

Data Set	TE Families		DE TEs	LTRs	LINEs	DNA	Helitron
Cell line	638	Upregulated	91	20	41	30	0
		Downregulated	95	23	29	40	3
		Other^a^	1	0	1	0	0
Midgut	614	Upregulated	46	23	7	16	0
		Downregulated	16	4	3	9	0

a This TE is alternatively down- and upregulated during the course of the experiment.

The time course RNA-seq data produced by Chen et al. (2013) shows that a number of TEs (8% of all TE families) are DE as early as 0 hpi, which in fact corresponds to the first 1-h incubation period of the cell line with the virus. As observed for host genes (Chen et al. 2014), the impact of the viral infection on TE expression is thus rapidly measurable. The number of TE families impacted by the viral infection is then quite stable through time until 36 hpi, followed by a slight increase afterwards (around 10.5–11% of TE families are DE from 6 to 36 hpi, and 14.8% at 48 hpi; fig. 2). However, the direction of the impact varies with time after infection, with the majority of DE TE families being downregulated early after infection (from 0 to 18 hpi) and upregulated at later time points (fig. 2). Contrary to TEs which are relatively stably affected by the infection throughout the experiment, the impact of AcMNPV infection on the expression of *T. ni* genes continuously increases with time, with about 20% of *T. ni* unigenes being DE at 0 hpi and 40% at 48 hpi (Chen et al. 2014). Moreover, the direction of gene differential expression is inverted compared to that of TEs, with most DE *T. ni* genes being upregulated early after infection (0 and 6 hpi) and downregulated at later time points (Chen et al. 2014). The processes underlying how genes and TE expression is affected by AcMNPV infection in Tnms42 cells are thus different.

**Fig. 3.**
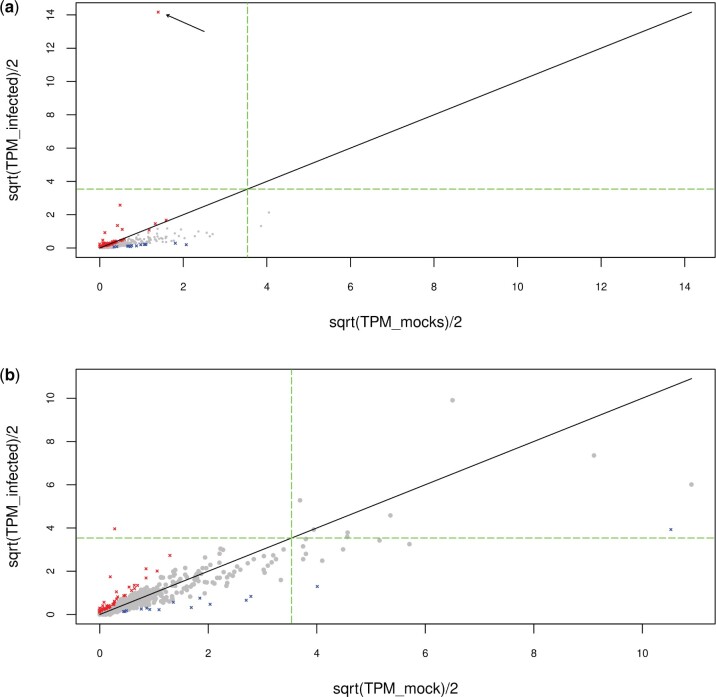
TE family expression in mock condition as a function of TE expression in infected condition.. TE families that are significantly DE are colored in red (upregulated) and blue (downregulated). The green dash line represents the cutoff for high expression, at 50 TPM. (*a*) TE expression in the Tnms42 cell line data set at 48 hpi. The arrow points to TFP3. (*b*) TE expression in the midgut data set at 72 hpi. The same analysis has been performed for all time points of the two time course RNA-seq experiments and results are shown in supplementary figs. S1 and S2.

The proportion of DE TE families was relatively similar for both retrotransposons (113 out of 354 or 31.9%) and DNA transposons (70 out of 278 or 25.2%). Furthermore, among DE TE families, the proportion of upregulated ones differed only moderately between the two types of TEs (43% and 54% for DNA transposons and retrotransposons, respectively) (table 1). Thus, overall, our results do not reveal any important difference in the way expression of the two TE classes is affected by AcMNPV infection in Tnms42 cells. Among all downregulated TE families (95) 11 showed a log2FC lower than −4, with an extremum at −4.95 (i.e., 31-fold less expressed). Three of these were DNA transposons (DNA/TcMar-Tc_7, DNA/PIF-Harbinger_3, and DNA/PiggyBac_10) and the remaining eight were retrotransposons (LTR/Gypsy_12, LTR/Copia1_4, LINE/R1_2, LINE/R1_3, LINE/R1_4, LINE/L2_10, LINE/L2_21, LINE/Dong-R4_1). Among the most upregulated TE families, six had log2FC higher than six, with a maximum at 9.11 for DNA/TFP3 (i.e., 553-fold more expressed). Four of them were retrotransposons (LINE/Proto_3, LINE/I, LINE/R1_8, and LTR/Gypsy_11) and the two others were DNA transposons (DNA/TFP3 and DNA/PiggyBac_9). DNA/TFP3 particularly stood out among these upregulated TE families because of its remarkably high expression level. From an expression of 7.8 TPM in the mock, it reaches 52 TPM at 6 hpi and 802 TPM at 48 hpi in the infected condition (fig. 3*A*). For comparison, the next highest expression level after TFP3 in the infected condition is 104 TPM. TFP3 is a 831-bp-long non-autonomous TE belonging to the piggyBac superfamily. It was first discovered inserted in AcMNPV genomes purified from *T. ni* (TN-368) cells (Fraser et al. 1983; Wang and Fraser 1993).

Out of 94 TE families found inserted in AcMNPV genomes in previous studies (Fraser et al. 1983; Jehle et al. 1998; Gilbert et al. 2014, 2016), 41 passed our filters to be included in our library (i.e., best match to a TE protein over at least 50% of the length of this protein). Among these 41 TE families, 28 were found in the genome of Tnms42 cells (marked with an asterisk in supplementary file 1). Only eight of them were found to be DE (DNA/TFP3, DNA/PIF-Harbinger_4, DNA/Sola_6, DNA/hAT_1, and LTR/Gypsy_15 were upregulated, whereas DNA/Sola_2, DNA/Sola_3, and DNA/PiggyBac_2 were downregulated). Among the 20 remaining TE families, only one was highly expressed (TPM ≥ 50) in the mock condition and 17 were not expressed (TPM < 4). Thus, there is no link between a specific TE expression pattern in infected Tnms42 cells and integration of TEs into AcMNPV in previous studies.

### Genome-Wide TE Differential Expression during AcMNPV Infection of *T. ni* Larvae Midguts

Reads produced by Shrestha et al. (2018) were mapped on the 66,683 TE copies annotated in the *T. ni* larva genome and the differential expression was computed by TE consensus, which we consider here as each representing a separate family (612 TE families included here). Among these 612 TE families, 148 are expressed in at least one time point in mocks (TPM ≥ 4). More precisely, 464 are considered as not or very lowly expressed in mocks (TPM < 4 in all time points), whereas 81 can be considered as always expressed with confidence (TPM ≥ 4 in all time points). Considering all time points, the median for TE expression in mocks is 0.41 TPM (compared to 0.37 TPM in the Tnms42 cell line) and the maximum is 1,847 TPM. Thus, as for the cell line data set, most TE families are not expressed at one or more time points in mock conditions, although the strength of expression is overall slightly higher in the midgut data set.

**Fig. 4.**
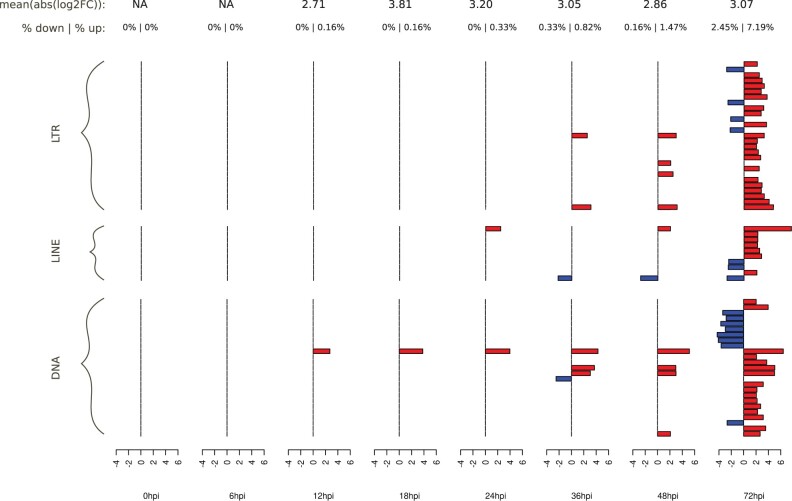
DE TE families in the midgut data set.. Only the 62 significant DE TE families with an absolute log2 fold change above 2 are shown. On top, the first line shows the average absolute log2FC at each time point, while the second line shows the percentage of TE families downregulated and upregulated at each time point. For each time point, log2 fold change is indicated in red for upregulated TE families or in blue for downregulated ones. TE family names can be found in table S2.

The number of DE TE families varied from 0 to 59 (9.64% of all TE families) depending on the time point during the course of the AcMNPV infection in *T. ni* larvae midgut (fig. 4 and supplementary table S2). When considering all time points together, a total of 62 TE families (10.13% of all families) were found to be DE during at least one time point. Overall, the strength of differential expression went from 19-fold decrease to 202-fold increase (i.e., log2FC = −4.28 to 7.66), with a median at 2.26 and an average at 1.31 log2 fold change. Among all 59 DE TE families, 46 were upregulated and 16 were downregulated during at least one time point (fig. 4 and table 1). Among the 46 upregulated TE families, 10 were induced, that is, they showed no or very low expression in the mock (TPM < 4) and were expressed in at least one time point in the infected condition (TPM ≥ 4), including one TE family (LINE/Proto_1) that became highly expressed (TPM > 50). Nine TE families were repressed in *T. ni* larval midgut (TPM ≥ 4 in mocks and TPM < 4 in the infected condition). Overall, these data show that as in the *T. ni* Tnms42 cell line, AcMNPV infection moderately affects the expression of several TE families, the majority of which are upregulated in the infected condition. The impact of AcMNPV infection on TE expression is lower than in the cell line as only 10.13% of TE families are affected (compared to 29% in the cell line) and both positive and negative maximum log2 fold changes (log2FCs) are slightly lower than in the cell line.

The time course RNA-seq data produced by Shrestha et al. (2018) reveals that contrary to the Tnms42 cell line, the number of TE families affected by AcMNPV and the strength of differential expression increase with time, with no DE TE family from 0 to 6 hpi and only one (upregulated) from 12 to 18 hpi and two at 24 hpi (fig. 4 and supplementary table S2). The number of DE TE families really began to increase at 36 hpi with seven DE TE families, followed by 10 DE TE families at 48 hpi and finally 59 at 72 hpi. Regarding the strength of differential expression, 94% of DE TE families reached their extremum of differential expression at 72 hpi (vs. 41.7% in the cell line data set). In contrast to the cell line, in which TE and gene expression seemingly responded differently to AcMNPV infection, the pattern observed in larval midguts is very much similar to that of *T. ni* genes. Indeed, only very few DE genes were detected at early time points (67 in total at 0, 6, and 12 hpi), followed by medium numbers at intermediate time points (82–475 genes) and a sharp increase at 72 hpi (1,910 genes) (Shrestha et al. 2019). Thus, in midgut cells, TE and gene regulation seems to be affected in a more similar way than in Tnms42 cells.

As in Tnms42 cells, the proportion of DE TE families was similar for both retrotransposons (37/338 or 10.9%) and DNA transposons (25/269 or 9.3%). However, among DE TE families, the proportion of upregulated ones is higher for retrotransposons (81%) than for DNA transposons (64%) (table 1). One of these retrotransposons (LINE/Proto_1) is the most upregulated TE family in *T. ni* larvae midgut, being 202-fold more expressed in the infected condition than in mock at 72 hpi (i.e., log2FC = 7.66) (fig. 4). This TE family is activated at 72 hpi, reaching 63 TPM. The two most downregulated TEs were two TcMar families, being repressed at 72 hpi (i.e., log2FC = −4.3 and −4.1 and TPM < 4), whereas in mocks they were expressed at 11 and 5 TPM at 72 hpi.

Moreover, out of 41 TE families found inserted in AcMNPV genomes in previous studies and that passed our filters to be included here (Fraser et al. 1983; Jehle et al. 1998; Gilbert et al. 2014, 2016), 23 were found in the *T. ni* larva genome (indicated with “°” in supplementary file 1). Only one of them (DNA/Sola_3) is DE, being upregulated at 72 hpi (log2FC = 3.7). This DNA/Sola was also DE (log2FC = −2.6 at 6 hpi) in the cell line. Among the 22 remaining TE families, two were highly expressed in at least one time point (TPM ≥ 50), with a median expression of 48 and 196 TPM in mocks and a maximum of 63.3 and 331.8 TPM, respectively. As observed in the Tnms42 cell line, there is no link between a specific TE expression pattern in *T. ni* midgut and the propensity of TEs to integrate into AcMNPV genomes.

### Investigation of Possible Factors Affecting TE Expression in *T. ni* Somatic Tissues

We assessed whether the expression level of TE families in the *T. ni* larvae mock condition could be associated with factors such as copy number, average proximity with genes, or age (as approximated by the average percent similarity between TE copies and the TE family consensus sequence) (supplementary fig. S3). Overall, we did not find any strong correlations with these three factors. We found a weak but significant positive correlation between TE family expression level and copy number (*r* = 0.09 at 0 hpi and *r* = 0.38 at 72 hpi in mock, *P* < 0.05 and *P* < 0.001, respectively). This correlation could be expected as it is difficult to see how increasing copy number could lead to a decrease in TE family expression level. Similarly, we found a very weak but significant positive correlation between TE family expression level and TE family age (*r* ∼ 0.12 at 0 and 72 hpi, *P* < 0.05). This correlation may appear counterintuitive as one could expect that younger copies may be more likely to be functional and more strongly expressed. However, it may be partly explained by the fact that TE copy number is also weakly but significantly correlated to TE family age (supplementary fig. S3). TE family expression levels were not correlated to average proximity of TE copy with genes. Interestingly, we also noticed that TE family expression levels were correlated in the mock and infected conditions (supplementary fig. S3), reinforcing the idea that, though AcMNPV infection impacts expression of a number of TE families in *T. ni* midgut, this impact is overall moderate.

### Expression of AcMNPV-Borne TE Copies

Our search for TE-virus chimeric reads revealed no such read in the RNA-seq data set from *T. ni* larvae infected by AcMNPV (Shrestha et al. 2018). This absence may be due to the fact that the AcMNPV genomes used to infect *T. ni* larvae bore no TE and/or no TE transposed de novo into AcMNPV during the experiment. Another possibility is that TEs carried by AcMNPV genomes used for these experiments were not expressed. However, we previously found that while a substantial proportion of AcMNPV genomes carry moth TEs, the vast majority of individual TE insertions segregate at extremely low frequency (Gilbert et al. 2016). For example, 99% of the 1,983 different TE insertions found in the AcMNPV-infecting *T. ni* G0 data set (the most deeply sequenced data set) were at a frequency lower than 0.1% and the highest insertion frequency in this data set was 1.4% (Gilbert et al. 2016). Furthermore, only a subset of these TE insertions may be cotranscribed with their neighboring gene. Thus, the absence of TE-virus chimeras in these data might not necessarily reflect absence of AcMNPV-borne TEs but such TEs might simply be expressed at levels too low to be detected with our approach. In this context, the short read-length (51 bp) might have further hampered our ability to detect TE-virus chimeras, as the blastn options we used does not allow finding alignments shorter than 28 bp. In addition, the average sequencing depth did not exceed 2,550× in this study. Though sufficient to detect TE insertions in principle (Gilbert et al. 2016), deeper sequencing would have undoubtedly increased the likelihood to detect expressed TEs.

By contrast, we were able to detect a large number of TE-virus chimeras in the RNA-seq data set from the AcMNPV-infected *T. ni* cell line (Chen et al. 2014). Considering the seven time points (six plus the 24 hpi not included in the DEseq analysis, see Materails and Methods) and the various biological replicates at each time point, 11,914 chimeric reads were identified. Among the fourteen TE families involved in these chimeras (supplementary file S2), six were found integrated into AcMNPV genomes in previous studies (Bauser et al. 1996; Fraser et al. 1996; Gilbert et al. 2016). Three class II piggybac and one Harbinger TE families were found in different replicates at different time points (table 2). The eight other TE families (seven class II and one class I) were found in a single or a just a few replicates or time points. The various TE copies found here integrated into AcMNPV genomes might result from de novo transposition from the Tnms42 cell genome or might have been present in the AcMNPV isolate used to infect these cells.

**Table 2:** Number of TE/Virus Chimeric Reads in the Chen et al. (2013) AcMNPV RNA-Seq Data Sets

		0 h		6 h		12 h		18 h		24 h		36 h		48 h	
		5′	3′	5′	3′	5′	3′	5′	3′	5′	3′	5′	3′	5′	3′
Replicate 1	TFP3	0	1	13	8	230	218	287	284	369	388	309	293	336	379
	PiggyBac (2105_S. frugiperda)	0		0		2	0	3	0	9	0	3	0	5	0
	PiggyBac (22360_S. mediterranea)					0	1	0	0	0	1	0	0	0	1
	Harbinger (HITCHHIKER)					1	1	0	1	0	0	0	0	0	1
	Others					1	0	1	1	1	2	5	0	3	3
Replicate 2	TFP3	0	2	16	8	440	519	241	229	347	390	394	423	379	373
	PiggyBac (2105_S. frugiperda)	0		0		4	0	1	0	3	0	4	0	6	0
	PiggyBac (22360_S. mediterranea)					0	2	0	1	0	3	0	1	0	1
	Harbinger (HITCHHIKER)					0	0	0	0	0	0	0	0	1	1
	Others					0	0	3	0	2	2	4	2	2	1
Replicate 3	TFP3	0		32	30	467	553	474	541	282	289	332	303	632	722
	PiggyBac (2105_S. frugiperda)			0		5	0	7	0	1	0	1	0	10	0
	PiggyBac (22360_S. mediterranea)					0	5	0	1	0	1	0	1	0	3
	Harbinger (HITCHHIKER)					1	1	0	2	1	0	1	0	2	2
	Others					7	0	0	0	2	0	2	0	4	1
	Total	0	3	61	46	1,158	1,300	1,017	1,060	1,017	1,076	1,055	1,023	1,380	1,488
		3		107		2,458		2,077		2,093		2,078		2,868	

Note.—TE families for which less than 10 chimeric reads were found in all data sets were lumped in the “Others” category. This table includes only chimeric reads mapping at the 5′ or 3′ extremity of TE families (*N* = 11,684).

Importantly, a single TE (TFP3) accounted for the vast majority of the chimeric reads (11,533 out of 11,914), with 5,580 and 5,953 reads aligning at its 5′ and 3′ extremity, respectively. Among the other chimeras, 64 aligned at the 5′ end of piggybac (2105_S.frugiperda), 22 reads aligned at the 3′ end of piggybac (22360_S.mediterranea), and insertions of Harbinger Hitchhiker TE were supported by 16 reads (7 at the 5′ extremity and 9 at the 3′ extremity). Among all 11,914 TE-virus chimeras, only 1.93% did not align at the TE tips but on their internal part, indicating that the vast majority of chimeras correspond to expression of TEs that were generated by bona fide transposition. Further supporting the biological nature of the chimeras detected in this analysis, we found target site duplications (TSDs) for TFP3 and Harbinger TEs. For example, for Harbinger, two chimeric reads were found to align on the viral genome 3 bp apart from each other, separated by a TTA motif (supplementary fig. S4), known to be typically duplicated during Harbinger transposition (Sinzelle et al. 2008). For TFP3, 19,491 reads were identified supporting TSDs: 4,940 reads at 12 hpi, 3,984 at 18 hpi, 2,784 at 24 hpi, 2,826 at 36 hpi, and 4,958 at 48 hpi. These reads indicated the expression of 202 different TFP3 insertions among which 44 were expressed at 12 hpi, 38 at 18 hpi, 24 hpi, and 36 hpi and 45 at 48 hpi. The two target site duplication (TSD) motifs flanking these insertions (TTAA and ATAA) correspond to those typically generated upon transposition of piggybac elements (supplementary fig. S4; Bouallègue et al. 2017).

Regarding the dynamics of virus-borne TEs during infection, we observed a sharp increase in the number of chimeric reads from 12 hpi followed by relatively steady counts afterwards. Three chimeric reads were detected at 0 hpi, 107 at 6 hpi, 2,458 at 12 hpi, 2,077 at 18 hpi, 2,093 at 24 hpi, 2,078 at 36 hpi, and 2,868 at 48 hpi (table 2). The peak of TE-virus chimeras detected at 12 hpi was in line with the results of Chen et al. (2014), who showed that the expression of AcMNPV genes reaches its highest levels at this time of the infection.

**Fig. 5.**
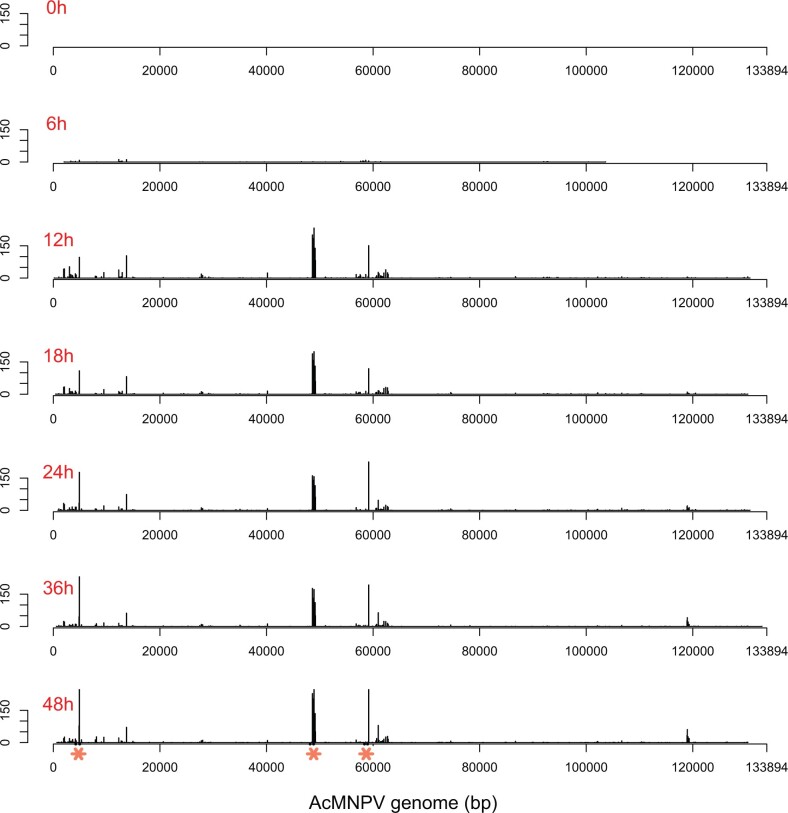
Distribution of expressed TE insertions along the AcMNPV genome for each time point in the Chen et al. (2013) data sets. The *Y*-axis corresponds to the number of reads. Insertions are binned into 50-bp windows. The three major insertion hotspots, shown by orange asterisks on 48-hpi graph, correspond from left to right to the *PH*, *FP*, and *Orf78* genes.

We then mapped the distribution of TE-virus chimeras along the viral genome for each time point pooling all replicates, only focusing on TFP3, which is by far the most expressed virus-borne TE. Figure 5 illustrates the sharp increase followed by steady expression of virus-borne TFP3 insertions at 12 hpi. It also reveals the presence of three highly expressed TFP3 copies, integrated at positions 4,856, 48,732, and 59,176 of the AcMNPV genome, in three different viral genes: *PH* (polyhedrin), *FP* (few polyhedra), and *Ac-Orf78* (fig. 5). To obtain further insight into the expression level of TFP3 copies inserted in these genes, we compared their expression to the overall expression of *PH*, *FP*, and *Ac-Orf78* as reported by Chen et al. (2013). Because Chen et al. (2013) measured AcMNPV gene expression levels in reads per kilobase per million reads mapped (RPKM), we also calculated the average RPKM over the three replicates for the three TFP3 copies inserted in each gene, for each time point (supplementary table S3). Importantly, TFP3 RPKM were calculated only taking the reads mapping on the virus-TFP3 junctions. Measuring the expression of viral-borne TFP3 copies over their entire length is impossible because it is impossible to assess which reads mapping internally to the TFP3 sequence come from TFP3 copies located in the *T. ni* genomes and which ones correspond to viral-borne copies. Thus, we measured the coexpression of TFP3 copies and neighboring viral genes. This is reflected by the fact that RPKM calculated for viral gene–TFP3 junctions are strongly correlated to those calculated by Chen et al. (2013) for *PH* (Pearson’s rho = 0.94, *P* < 0.001) and *FP* (Pearson’s rho = 0.75, *P* < 0.05) genes. In other words, the more *PH* and *FP* are expressed, the higher the expression of TPF3 copies inserted in those genes. Because only the TFP3–virus junction is considered for measuring TFP3 expression, it is likely that the true expression level of viral-borne TFP3 is much higher. Yet, it is noteworthy that even underestimated, the overall expression of the three viral-borne TFP3 copies at 12 hpi (935 RPKM) is higher than the maximum expression level reached by about 35% of AcMNPV genes during the entire duration of the experiment (see supplementary fig. 3 in Chen et al. 2013).

These results are in line with the high upregulation of TFP3 during the course of the infection we observed in our analysis of DE TEs in the cell line data set. Indeed, in the cell line data set, this TE was found to be the most upregulated TE and the most expressed in late infected conditions (fig. 3 and supplementary fig. S1). Interestingly, our results also suggest that the upregulation of TFP3 upon viral infection may be due in large part to expression of viral-borne TFP3 copies rather than to enhanced expression of TFP3 copies located in the *T. ni* genome, which would explain why TFP3 was such an outlier. If the absence of TFP3 chimeric reads in the midgut data set is biological, the absence of insertion of this TE in AcMNPV might explain why TFP3 was not DE in the midgut.

## Discussion

### Impact of a Baculovirus Infection on TE Expression in *T. ni*

We characterized expression patterns of TE families in midgut of larvae as well as in Tnms42 cells facing a biotic stress in the form of an AcMNPV infection. We found that the genome of *T. ni* larvae (Chen et al. 2019) and Tnms42 cells (Fu et al. 2018) has a similar percentage of DNA, LTR, and LINE families, with 636 TE families in the cell line genome and 612 TE families in the *T. ni* larvae genome, including 587 families in common. We further showed that in the mock condition at 0 hpi, a larger number of TE families are confidently considered expressed in the midgut data set (60 in Tnms42 cells vs. 101 in midgut), with 30 shared expressed TE families. Taking all time points postinfection together, our analyses reveal that a moderate number of TE families are DE in AcMNPV-infected *T. ni* Tnms42 cells (187/636 TE families) and midgut (62/612 TE families), with widely overlapping ranges of fold changes in expression in the two data sets (from 30-fold decrease to 553-fold increase in cells and from 19-fold decrease to 202-fold increase in midgut). Of note, these fold changes overlap with those calculated for non-TE *T. ni* gene expression in Tnms42 cells (from 134-fold decrease to 20.8-fold increase; Chen et al. 2014) and midgut (from 640-fold decrease to 163-fold increase, Shrestha et al. 2019) but they tend to be shifted toward stronger upregulation. Altogether, our results are in line with earlier studies and further suggest that a viral infection can affect TE activity and thus influence genome architecture in animals (Macchietto et al. 2020; Roy et al. 2020). The overall magnitude of the changes in TE family expression level is, however, moderate here, with TE expression levels in mocks being overall strongly correlated to those in infected conditions, no sign of strong global TE unleashing, and relatively small fold changes for most TE families in both data sets.

### Differences in TE Expression in Response to AcMNPV Infection in *T. ni* Midgut Tissues and Tnms42 Cells

Besides a marked difference in the proportion of TE families affected by AcMNPV infection in Tnms42 cells (29.4% of all TE families) and *T. ni* midgut (10.1%), the response to AcMNPV also differs in terms of the proportion of up- versus downregulated TE families between the two data sets. While there are in total three times more upregulated than downregulated TEs in the midgut (fig. 4 and table 1), there are about as many up- as downregulated TE families inTnms42 cells (fig. 2 and table 1). The impact of AcMNPV infection on TE expression tends to be unidirectional in the midgut, with a relatively steady increase in both the number of DE TE families and strength of differential expression with time (fig. 4). By contrast, in Tnms42 cells, the pattern of differential expression is more erratic, with many TE families that are DE at early time points postinfection not remaining DE in the next time points, and extrema of differential expression at any time point depending on TE families (fig. 2 and supplementary table S1). Altogether, these findings are rather consistent with what is known about cell lines, which generally grow under fewer constraints than tissues and often undergo important chromatin remodeling and chromosomal rearrangements, as described for the *T.ni* Hi5 cell line (Fu et al. 2018). Such modifications could lead to higher TE activity in cell lines during stress conditions, as TE-restriction pathways may be less efficient, in part due to the presence of a unique cell type (ovarian germ cells, in the case of *T. ni* Hi5 cells; Granados et al. 1986; Granados et al. 1994). The larger proportion of upregulated TE families in the cell line versus midgut (91 vs. 46) is also in accordance with Macchietto et al. (2020) who found a global upregulation of TE expression after viral infections of various cell lines. However, while Macchietto et al. (2020) observed an early wave of TE upregulation, here, we find that TE upregulation occurs mainly at later time points in Tnms42 cells.

When looking at the impact of AcMNPV infection on TE expression by TE types, there are clear differences between Tnms42 cells and *T. ni* midgut tissues. These differences are perhaps best illustrated by the fact that only 26 shared TE families were DE in both data sets (supplementary table S1) and that for a given TE family, the direction of differential expression was not necessarily the same in Tnms42 cells and *T. ni* midgut. In fact, only 14 of these shared DE TEs were DE in the same direction (10 upregulation and 4 downregulation) in both data sets. Furthermore, the TE families with the highest or lowest log2FCs in one data set were not DE in the other data set. More globally, the differential impact of AcMNPV infection in the two data sets is also illustrated by the fact that the proportion of TE families that are DE among each TE type significantly differs between Tnms42 cells and *T. ni* midgut (*P* < 0.01, chi2 test). For example, the proportion of DE DNA transposons is twice higher in *T. ni* midgut than in Tnms42 cells (9% of DNA TEs in midgut and 25% of DNA TEs in cells). Furthermore, while a relatively similar proportion of LINE and LTR families is DE in the cell line (35% of LINEs and 27% of LTR), there are 3.5 times more LTR than LINE families that are DE in the midgut (18% of LTR vs. 5% of LINE). These differences of regulation in both data sets are possibly due to differences in the regulatory landscapes of the two cell types. Another explanation can be the expression of copies specific to each genome (i.e., present in one but not in the other genome) located in different genomic regions. The low number of DE TEs shared between both data sets is not unexpected given what is known about the impact of stress on TE expression in eukaryotes, as no unique, clear trend emerges (Horváth et al. 2017). This suggests the nature and/or the strength of the interactions between host cells, TEs and the virus differ between the cell line and a living organ, at least in *T. ni*. In this respect, it is noteworthy that *T. ni* Hi5 cells (from which the Tnms42 cell line derives) differ widely from ovary (the tissue from which the cell line is derived) and other *T. ni* tissues in their piRNA response. For example, only 71 piRNA clusters were annotated in Hi5 cells compared to 348 in *T. ni* ovaries and many piRNA clusters that are active in ovaries only produce few piRNA in Hi5 cells (Fu et al. 2018). In addition to the piRNA pathway that actively represses TEs in lepidopterans (Lewis et al. 2018), epigenetic marks, such as 5-methylcytosine, are involved in TE regulation (Deniz et al. 2019). Thus, differences in the strength of the piRNA response and/or in epigenetic landscape may explain the variation of TE expression observed between the larvae and cell line.

### Transposable Elements Previously Found Integrated into AcMNPV Genomes Show No Specific Expression Pattern

Interestingly, the *T.ni* TE families previously found to be inserted in AcMNPV genomes (Gilbert et al. 2014, 2016) are not more represented in DE TEs, and even the DE ones are as much down- as upregulated. This suggests that the ability of some *T. ni* TEs to transpose in viral genomes upon infection is not linked to stress-mediated overexpression of these TEs. Looking at the absolute TE expression, we observed that even after upregulation, most DE TE families are overall not the most expressed TEs (fig. 3 and supplementary figs. S1 and S2). Thus, it might be possible that DE TEs were not found inserted in AcMNPV genome because their upregulation was not strong enough to reach sufficient expression. Moreover, some TEs found inserted in AcMNPV were weakly expressed in our study, suggesting that a weak expression might be enough for insertion in AcMNPV. Alternatively, transposition into viral genomes may occur in tissues other than those constituting the midgut or this cell line, in which TE expression might be different. In this regard, it is noteworthy that the tissue tropism of AcMNPV includes most cell types of lepidopteran larvae (Engelhard et al. 1994; Barrett et al. 1998; Rahman et al. 2004). It would thus be interesting to repeat this analysis on several other tissues and/or on whole larvae.

### Transposable Elements Integrated into Viral Genomes Can Be Expressed

Our results show that at least 11 TE families from a *T. ni* cell line can be inserted in and transcribed from AcMNPV genomes. Our approach only allows us to detect TE copies that are cotranscribed with the upstream or downstream viral gene. Yet we predict that viral-borne TFP3 copies may be expressed from their own promoter, as piggybac elements are known to carry such a promoter, located in their 5′ repeated sequence (Cadiñanos and Bradley 2007). Chimeric reads are not expected if AcMNPV-borne TEs are transcribed from their own promoters. Viral-borne TFP3 copies are identical to TFP3 copies located in the genome of *T. ni* and it is thus not possible to assess which proportion of the RNA-seq reads mapping to the internal part of the element correspond to viral-borne or host-borne TFP3 copies. For the same reason, it was not possible to assess whether some TE transcripts were coencapsidated into virions but not inserted into the viral genome in our data sets, as found in several RNA viruses (e.g., Routh et al. 2012). Thus, although the expression level of virus-borne TFP3 copies is here equivalent to that of some AcMNPV genes, the expression of these virus-borne TFP3 copies is likely underestimated.

The three genes (*FP*, *PH*, and *Ac-Orf78*) bearing highly expressed TFP3 copies are known to be involved in the formation of occlusion bodies (OBs). Inactivation of *FP* or *PH* leads to a drop of AcMNPV OB formation (Hink and Vail 1973; Fraser et al. 1983) and *Ac-Orf78* is associated with a structural protein that is essential for infectious OB formation (Tao et al. 2013). Interestingly, OBs are not necessary for the virus to replicate in cell lines and viruses unable to make OBs have a replication advantage over OB-forming viruses (Wood 1980). This likely explains why most TEs found integrated in AcMNPV genomes in early studies were located in the *FP* or *PH* genes (Fraser et al. 1983; Bauser et al. 1996). It is thus likely that the TFP3 insertions in *FP*, *PH*, and *Ac-Orf78* increased in frequency during passage of the virus in the *T. ni* cell line because their fitness cost is much lower in these genes than elsewhere in the AcMNPV genome, or because they may provide a replication advantage to the genomes bearing them. However, the presence of TFP3 copies integrated in these genes did not impede their expression, as shown by the presence of many TE-virus transcripts (chimeric RNAseq reads), increasing our ability to detect TE-virus chimeras in this data set. Importantly, the longer read length (101 bp) produced by Chen et al. (2014) also probably contributed to more efficiently detect TE-virus chimeras than in the Shrestha et al. (2018) data set (read length 51 bp).

In conclusion, we found that TEs integrated into AcMNPV genomes can be expressed at substantial levels, a prerequisite for such TEs to be able to further transpose from viral genomes to other viral genomes or to the genome of another host. Thus, our results further contribute to support viruses as potential vectors of TEs between animals. Importantly, they also suggest that analyses of DE TEs during a viral infection must be interpreted with caution as an increase in TE expression level could be in part caused by expression of viral-borne TE copies rather than overexpression of host-borne TE copies.

## Materials and Methods

### RNA-Seq Data of Tnms42 Cells Infected by AcMNPV

RNA-seq data were retrieved from Chen et al. (2013) [Sequence Read Archive (SRA) accession number SRA057390]. Briefly, *T. ni* cells from the Tnms42 cell line, which derives from Hi5 cells, were infected with the wild-type AcMNPV strain E2 (Chen et al. 2013). For infections, 3 × 10^6^ Tnms42 cells were infected at a multiplicity of infection (MOI) of 10. After a 1-h incubation, the inoculum was removed and the cells were rinsed and further cultured with new medium. The time at which the inoculum was removed was designated 0 hpi. Total RNA was isolated from AcMNPV-infected cells, as well as from a set of parallel control cells (uninfected or mock infected), at 0, 6, 12, 18, 24, 36, and 48 hpi. Polyadenylated RNA isolated from 20 µg total RNA was used for sequencing. The sequencing library was constructed with the TruSeq protocol and sequenced on an Illumina platform. Single-end reads of 101 bp were produced for the infected condition and for the 0-hpi mock condition, whereas 51-bp reads were produced for the mock condition of the other time points and for one replicate at each time point of the infected condition. To avoid any bias potentially introduced by different read lengths, we only used replicates produced from 101-bp reads. Further information can be found in Chen et al. (2013). Please note that reads corresponding to the mock condition at 24 hpi cannot be retrieved from the SRA.

### RNA-Seq Data of Midgut *T. ni* Larvae Infected by AcMNPV

RNA-seq data were retrieved from Shrestha et al. (2018) (SRA accession number PRJNA484772). In this study, *T. ni* fourth-instar larvae (Cornell strain) that were ready to molt were held for 0–5 h without diet, and newly molted 5th instar larvae were used for oral infections. Larvae were orally inoculated with 5 µl of a 10% sucrose solution containing a total of 7 × 10^4^ OBs of wild-type AcMNPV strain E2 (as in Chen et al. 2013). Mock-infected control larvae were fed a similar sucrose solution containing no virus. Midgut tissue was dissected at eight time points post infection: 0, 6, 12, 18, 24, 36, 48, and 72 hpi. For each time point sampled post infection, a parallel mock-infected control midgut sample was dissected. For each time point and treatment (infected or control), three replicate samples were prepared, with midgut samples from six larvae pooled for each replicate. Total RNA extraction was performed on pooled midgut samples. Poly(A) mRNAs isolated from 3 µg of total RNA were used to construct a library with the TruSeq protocol and sequenced on an Illumina platform. Single-end reads of 51 bp were generated. Further information is provided in Shrestha et al. (2018).

### *T. ni* Genomes Used in TE Differential Expression Analyses

Two *T. ni* genome assemblies were retrieved from GenBank: 1) one derived from a single male *T. ni* larva (accession number PPHH01000000; Chen et al. 2019) and 2) one derived from the *T. ni* Hi5 germ cell line (accession number NKQN00000000; Fu et al. 2018). Importantly, the larvae used to sequence the genome in Chen et al. (2019) and to produce the midgut RNA-seq reads in Shrestha et al. (2018) arise from the same strain (Cornell strain). Midgut RNA-seq reads were thus mapped onto TE copies retrieved from the genome assembled by Chen et al. (2019). Similarly, the Tnms42 cell line, used to produce RNA-seq reads in Chen et al. (2013) is an alphanodavirus-free derivative from the Hi5 cell line, for which a genome is available (Chen et al. 2019). The cell line RNA-seq reads were thus mapped onto TE copies retrieved from the Hi5 cell line genome.

### TE Identification and Database

The TE library used to annotate TE copies in *T. ni* genomes was compiled as follows. First, RepeatModeler version 1.0.11 (http://www.repeatmasker.org) was run with default options on the in vivo *T. ni* genome, which allowed us to identify 567 TE consensus sequences. In addition, 458 TE consensus sequences of the *T. ni* Hi5 genome were retrieved on https://cabbagelooper.org/. We also added to our TE library 94 TEs previously found inserted in viral genomes (Wang and Fraser 1993; Fraser et al. 1995; Gilbert et al. 2016). Finally, we annotated TEs in the RNA-seq data. The 48 data sets produced by Shrestha et al. (2018) were pooled and assembled with Trinity version 2.1.1 (Grabherr et al. 2011). The resulting 45,094 contigs were then mapped onto the AcMNPV strain E2 genome (GenBank accession number KM667940.1), which led us to remove 45 viral contigs. RepeatModeler version 1.0.11 was then run on the remaining contigs, which yielded 183 TE families. We also aligned the 45,049 nonviral contigs on a library of TE proteins (“RepeatPeps”) provided in the RepeatModeler package using diamond (Buchfink et al. 2015, options: “diamond blastx -more-sensitive”). We retained 151 contigs which aligned over at least half of a TE protein. The same approach was applied to the RNA-seq data sets from Chen et al. (2013). After the Trinity assembly, we found 103,650 nonviral contigs out of 103,790. Among them, 472 TE families were identified by RepeatModeler and 612 by alignment on the RepeatPeps library. A total of 2,535 TE sequences were retrieved in the genome and transcriptome assemblies. Clustering of these sequences using Vsearch (options used: “–target_cov 80.0 –query_cov 80.0 –id 0.95”) (Rognes et al. 2016) revealed that they were all unique. Finally, to remove TE sequences for which a robust annotation could not be achieved, we aligned the 2,535 TE sequences on the RepeatPeps library and kept only TEs being >300 bp in length and aligning on at least half of a TE protein. All sequences identified as “SINE,” “tRNA,” “rRNA,” or “Unknown” were discarded. Our final TE library containing 847 TE families is provided in supplementary file S1 and was used to annotate TE copies in the two *T. ni* genomes using RepeatMasker version 4.0.7 (http://www.repeatmasker.org). We then grouped RepeatMasker hits into more complete TE copies with the tool “One code to find them all” with the option –strict and –unknown (keeps only the copies greater than 80 bp in length and with more than 80% identity with the consensus) (Bailly-Bechet et al. 2014).

### Mapping of TE Copies

The RNA-seq data were trimmed using Trimmomatic version 0.38 (Bolger et al. 2014) to remove adapters and low-quality bases. After trimming, reads <40 bp in length were discarded (command line used: java -jar trimmomatic-0.38.jar SE -threads 30 -phred33 reads_R1.fastq reads_R1_TRIMMED.fastq ILLUMINACLIP: TruSeq2-3-SE.fa : 2:30:10 LEADING : 3 TRAILING : 3 SLIDINGWINDOW : 4:15 MINLEN : 40). The trimmed RNA-seq data were mapped to the TE copies of their corresponding *T. ni* genomes with Bowtie2 v2.2.4 (Langmead and Salzberg 2012) with the most sensitive option and keeping a single alignment for reads mapping to multiple positions (–very-sensitive for Bowtie2). The minimum criteria for a read to align on a TE copy with the “very-sensitive” option is at least an alignment of 20-bp substring without any mismatch, with a 6-bp interval. It corresponds to 6 and 14 20-bp substrings for a read of 51 or 101 bp, respectively.

### Count Tables of TEs and Genes

We produced count tables for the two time course RNA-seq data sets (Chen et al. 2013; Shrestha et al. 2018), independently for each data set and time point. Gene count tables were generated with Kallisto (Bray et al. 2016) for normalization purpose. The entry files provided to Kallisto were fastq files containing trimmed RNA-seq reads and a file containing the host transcriptome (tni_transcript_v1.fa at tnibase.org for the midgut data set and GBKU01.1.fsa_nt on NCBI for the Tnms42 cells data set) and the 156 CDS of AcMNPV reference genome (NC_001623). TE count tables were generated with the module TEcount of TEtools version 1.0.0 (Lerat et al. 2016). Entry files provided to TEtools were the sam files containing mapping information on the TE copies for all replicates of each time point, both for mock and infected conditions, the fasta file of the TE copies and a rosette file giving the name of the TE family for each TE copy. Gene counts and TE counts were then concatenated.

### Differential Expression Analysis

Differential expression analysis was computed on the concatenated count tables with the R Bioconductor package DESeq2 (Love 2014 Genome Biology), using an FDR level of 0.05 (Benjamini and Hochberg 1995). For this, the DESeqDataSet object was built with DESeqDataSetFromTximport for the gene counts and with DESeqDataSetFromMatrix for the TEs counts, both with the design ∼ condition. DESeq was then run on the concatenated DESeqDataSet object, and the results were generated with the contrast c(“condition,” “infected,” “mock”). For the midgut data set, we followed the procedure used by Shrestha et al. (2018) to study *T. ni* gene expression and compared normalized read counts between infected larvae and mocks for each time point postinfection. For the cell line data set, we also followed Chen et al. (2013). They reasoned that contrary to AcMNPV-infected *T. ni* cells, which stop dividing, uninfected cultured cells undergo important stresses as they grow due to space constraints, which may induce variation in gene expression in the mock condition after some time. For this reason, Chen et al. (2013) calculated differential expression of Tnms42 genes by comparing normalized read counts for each time point postinfection in the infected condition to read counts obtained in the mock at the first time point postinfection [0 hpi, corresponding to an hour of incubation by the virus, as mentioned in Chen et al. (2013)]. We were interested only in DE TE families; thus, we discarded the differential expressed genes that we initially included only for normalization purpose. TE families were considered as differentially expressed if their adjusted *P*-value was <0.05 and their absolute log2 fold change was ≥2. All analyses were performed using R version 4.0.4 (R Core Team 2019, https://www.R-project.org/).

### TPM Computation

Based on the concatenated gene and TE count tables, TE family counts were normalized to TPM. For each data set, we first calculated the number of reads per kilobase (RPK) by dividing the count of each gene or TE family in each replicate by the length of the gene or the length of the corresponding TE consensus in kilobases. RPK of all genes and TE families were then summed up by replicate and divided by one million. We finally used this million-factor for each replicate to divide all RPK values previously calculated. Since Wagner et al. (2013) suggested to use either 2 or 4 TPM as cutoff for nonexpressed genes, we chose 4 TPM as a cutoff. The cutoff for a highly expressed TE family being quite arbitrary, we chose 50 TPM because it corresponds to about 100 RPKM in our data set, which was used as a cutoff for highly expressed genes in Chen et al. (2013). For visual purpose, we chose to plot the TPM with a square-root transformation. Indeed, as explained by Wagner et al. (2013) and also as observed with our data, the standard log transformation leads to an over-dispersion at low TPMs, which might let one think that TE families show a relatively large and continuous range of expression levels, whereas most of them are actually lowly expressed.

### Correlations with TE Expression

We investigated possible correlations between the expression level of TE families in *T. ni* larvae mock condition and the following factors: TE copy number, TE age, and TE proximity with genes. For TE copy number, we counted the number of copies included in our study for each TE family (the copies which passed the “One code to find them all” filters). TE age was estimated by the percentage of divergence to the consensus for each copy. We used the average divergence of copies to estimate the age of a TE family. About TE proximity with genes, we indicated a distance of 0 for TE copies inside genes, otherwise, we counted the distance to the closest gene, in base pairs (genome annotation tni_gene_v1.gff3 at tnibase.org). The closest gene could be downstream or upstream; in any case, we calculated only positive values. Then, we calculated the average distance to nearest gene for each family. We investigated correlations between factors with the R package “corrplot,” using the Pearson method.

### Detection of TE/Virus Junctions in Transcriptomic Data

In addition to the DE analysis, we measured the expression of host TEs integrated into viral genomes. For this, we identified RNA-seq reads carrying a junction between a moth TE sequence and the AcMNPV genome. Such chimeric reads correspond to portions of transcripts that start in a viral gene and continue in a TE sequence integrated in the viral genome. This approach allowed us to ensure that only TE-containing transcripts initiating in the viral (not host) genome were included. To identify chimeric reads, all reads were aligned to the AcMNPV WP10 genome (GenBank accession number KM609482) and to a library of TEs including all TEs annotated in this study (supplementary file S1 and see above) and many other TEs found in various databases. Analyses to identify chimeric reads were performed on R (R Core team 2019). The pipeline we used was developed by Gilbert et al. (2016). Briefly, reads are aligned separately on host sequences and the viral genome using blastn (-task megablast). Chimeric reads for which a portion aligns on a host sequence *only* and the other portion aligns on the viral genome *only* are then identified based on alignment coordinates. All TEs found integrated into and expressed from AcMNPV genomes are provided in supplementary file S2.

### Identification of Target Site Duplications

To confirm host TE insertions in viral genomes, we searched for TSD that are signatures of canonical transposition. We separated chimeric reads in 5′ of a TE sequence from those in 3′. To be sure reads in 5′ and 3′ corresponded to the same insertion, we used different criteria. The viral insertion coordinate had to be equal to more or less 5 bp between the 5′ and 3′ chimeric reads. The same TE had to be detected at this insertion point. The 5′ and 3′ chimeric reads had to have a concordant orientation.

## Data Availability

The data used in this article are available in the GenBank Sequence Read Archive [SRA] database under accession numbers SRA057390 (Chen et al. 2013) and PRJNA484772 (Shrestha et al. 2018). Supplementary file S1 contains the consensus sequence of the 847 TE families used to annotate TE copies in *T. ni* genomes. Supplementary file S2 contains all TEs found integrated into and expressed from AcMNPV genomes.

## Supplementary Material

Supplementary data are available at *Genome Biology and Evolution* online.

## Acknowledgments

C.G. acknowledges funding from ANR grants ANR-15-CE32-0011-01 TransVir and ANR-18-CE02-0021-01TranspHorizon.

## Supplementary Material

evab231_Supplementary_Data
