# Supplementary material for: Assessing the Impact of a Viral Infection on the Expression of Transposable Elements in the Cabbage Looper Moth (Trichoplusia ni)
Source: Genome Biol Evol. 2021 Oct 6;13(11):evab231. doi: 10.1093/gbe/evab231 (PMC8634313; doi:10.1093/gbe/evab231)
Supplement: evab231_Supplementary_Data [file evab231_supplementary_data.zip › FileS1.docx]

>Spodo_Contig_7_Harbinger

NNNNNNNNNNNNNNNNNNNNNNNNNNNNNNNNNNNNNNNNAGAGCCAGTCCACACGGCGCGTTGCGTTGCGTTGCGTCGT

CGCAACGCAAATATTTTGACGCATAGCCGGCCACACGGGCGCTGCAGCGTTACTATGACGCAGTCCGTTCCGTCGGCGCA

GCGGCGACGCACAGTTGCGGCAAGTATTTTGAAAAAAATTCGCCAGTCTATAGCAAATCATGGATCGGAAAAGACGTCTA

GCATTGCTCCTATTACTACGTCACCGCCGAAATCGACGCAAGATCACAAGACGGTACTGGATCAGTCCTTTCATTTCATT

AAGAAATCGTGATGGACAGTTTTTTTTTTTAGAATATCGGGAGTTGCTATTAGACGAAAAGAAGTTTTATAATTTTTTTA

GAATGAGTGTATCCAGCTTCGAATGTTTATTGAAGTCCTTAGAACCACACATACGAAAAAACTATACTAATATGAGGAAT

CCAGTGGAACCAGTAGAAATGCTGGGAATCACTTTAAGGTAACTATATAAATATATTTAAGTAATAAAAATATAACAGTT

TTTTTGGTTTGTTTAATTAGTAATCAACTCACAAATAATCAGCAATTGTCATAATTTTAAATTTAGACATTATTTAAGAG

AGATATCATCCAGTAATTACAAATTAGAAAAATCGTATTCAGTTTCTTCACTTGCTTGAGATGTTTCTGGAGATGTAATT

GAATTATTATAACCAGAAATGTATGTACTTTGAAAATTTGCTGTTTGATACCCATATGGTGGTTGGTGAGACGTTGATGG

GCCACTCACATATGATGATGTTTGTCCATACCGCATTTCATCTATAATTTGTATAACTTTACTTTGGAAACGGAGAGTTT

CTCGGTCGGAAAATTCTTGAATAGATGGCAATATAGCTCTGAAAAAGGACATATGGCGATTTTCCGGCTCCTTGAGAAGT

TTTAGTCCTTCTCTTTCAAACTCATCCATTTGCATTGCCCTTTTGCGCGCGACTGGAACATAGCGCGGAGTGTTGTCTGT

GGTAATGTCATCATTTGTAGACGTAGATTCATTGTTGATTTCTCTAGGCGAGTCTAAGCTAGATTCGGTGGCATTTTGTT

CTACTTTTCTCAAAAAAAGGAGTTGATTGTATAAATGATACTTCTTCAGTTTCGGAGCGCCCGAGCCCGATTTTGATGTT

TCTTTTAATTTTTTTGAATATCTGAAGTAATTATCTTTTACACTTCTCCATTTTTTTATTAAATCATTACCTGCAAAACC

AATAGTAAACATGATTTAATAATTTATACATAGTGAATTTCTCGATAAAAATTTAATGACATATTCAGATATCGTTAACA

AACACTATGTGATCGACTCTGGAATCGTGAAAATATAACAGCTGTTACATACAAAAAGCAATACCTACTGAATCAAAATG

TATGCAACAGCTGCTATTTTTTTCCAGAATTAATTGGTTACATAGAGAAAGTTATTTGCTATCGATGTCTGAATATGTCA

GTATTTTTTTCGGGTATCTATCTATCTCATAAATGCATATTATAATAATTATTTTCTTTTCAGATACCTAGGAAGTGGAA

ATTCAATAACTGATTTACATTTCAAATTCAAACGGGGAAAATCTACTATTGCATATATAATACAAAGAGTTTGTCGTGCT

ATATGGACCAATCTTCTTCGAGACAACATCCCTGAACTGACAACTGAAAGTTTCCAAACAATAGCGAGGGGTTTTGATGT

AAAGGCAAATTTTCCTCAATGTGTTGGTGCCATCGACGGCAAACATATCCGCGTGTGTAATCCTGCAAATAGTGGCTCAC

TTTTTTTTAATTATAAAGCCTTTTTTTCGATTGTGTTGCTAGCTATTGTGGATTCAAATTACAAATTCGTATTTGTCGAC

ATCGGTGCATACGGAAAAGAATGCGATTCAACCATATTACAAAATTCTAAACTGTACGAGCTAATGATTAACAACAACTT

ACCACTACCTCAACCCCAGCCACTCTCTGGTAACAATATACCAACCCCGTATGTATTTGTGGGTGACGAAGCTTTTGGAC

TGAGCAAACATATTATGCGTCCATATGGCGGTCAAAATCTCGACTTACAACAAAAGGTTTTCAATTACCGTCTAAGCAGA

GCCAGAAGATATGTCGAATGCGCTTTTGGGATTATGGCTAACAAATGGCGCATTTTTCACAGACCGATAGACGTGTCCTA

TGACTTCGCTACTGACATTATAAAAGCATGTTGTGTATTACACAATTTTGTCGCTGATCGAGATGGTTTTAGACAAAGAG

ATAAATTTGCTATAAGTGTTGATGAATTTCTCCCAATACAACCCGTACATGAAGAACAGACAGCACCGAATGTCATAAGA

CAGCAATATGCGACCTATTTTATGACTAGAGGAACTCTGCCTTGGCAGCTAAATAAGGTATAATTGTATTATGAGGGTTT

TCAAGATTTTCTTGCACCGCCAATTCCGCGATCAGTAAAAAAATAATATTTTATACCCACTCAGCGTAGTCTAGGTTAGT

TTTGAGCTAAGTAATAAAATAGTACTCACGCTAATCTAGATTCCCAAAGTTTTAAAATTTTAGAGTGTCGAGATATTAAT

TTTGTACTGATCGCGGAATTGGCGGTGCAAGAAAATCTTGAAAGCCCTCTTAAATAAAAAAAAAACATAAATATCTGTAC

TGTGAACTCACCAACTTTGTTTTTTCGTTCAGCTGTGAAATTCTCGTAATCAGGAAATAAAACTTTTGATATGTCTTCCC

AGGCTTTTGTTTTTAAATTTTTATTTTTAAAATCTACGTTTGACTTGTCCCATATAATTTCCCTTTCCTGTATCAATGTA

ATCAAGTAATCTATGTCAATTTCTTCAATGGTATTCATTTTACCCTGCAAATAAAACTAAATACAAAAACGTAAACGTAA

AACGAAATGAGCTTCCGTGTATCCTCTCTGTCAACGCACAAGCAACTGAGCGCTGGCGCTCCGACGTCGCACGAGTACAC

CCCCTCCCGCTTCGTCTCGGGGGCTGCTGTCCGTCATGTGTGCGTTGCGACGACGCAGCGCGCCGTGTGAACACCTTGGT

TATTTGTATGTAAAACAACGCAGTGGTAGCGCTGCGTCGACGCAACGCTGACGCAACGCGCCGTGTGGACAGTCTC

>Spodo_Contig_24_undetermined *°

TATAAATAAGTTTATTTTAAATATATAAAAAAAATTCTTACACATTAAAATCCTGATCGCTCTGGGTTGACCCAACTCCC

GATTGAATGAAAAAAATGTTCTTTAAATTTTTAATGAGAAGGTTCATATCAAACAACTTATTTAGATTACATAACAAATA

GTTTTTCTTCATAGTAATAAAATATTTTTAGTTATATTAACAATACTTTTGCCACAATTTGAATTTAAAATCGTGACCGG

GCATTTTATCTCATCGCTCTGCGCTGAGGTAAAACATCGCGCACATCCCGAGATACGCGTTTTAGACACTATGTTCCCCT

CCCGCGGGGCAACGCCAGTAGTGATCTAACCGCCGAGCAGGATACATGTGTGCCACGTTCCCGCGCTCCAACATTACGAA

GGTAAGTTTATGATTGCTCTGTATTTGGTTCTCGAATTTTATGATTGCTATGGCACGTTATTAAAAATAAATAGTAAGCT

TGTGAATGTGTTTGTTATGTGATTTAAGTAATCGTTGTGATGTTTTTTGTGGGAAGTAATCACAACTGTCTTCTGCTTTT

ACATTCGGCTATATTTATGATTTTGTGACTTTATCTGATCGCTATGGCTGTGATTGCTCTGTCCAAAAGGACCAGAGCGA

TGATATTTTACATTAATTGGACGTTATAAATCTATTTTAAGTAGTATTACCGAACTGTAATAATGACAAACGATGCATAT

AACATTTAATTTATAGCCTTTGTTGGGTTTCTTTGGGTAAGCTAAATCGATAAACAGATTTTGACGTATTTTATCTTAAT

GATATTATTATTATATTTTAATTTAGTTAACCACAACTATAATCCTTTCTTCTCTAAAATTTAATGGCAACTATTGTTGC

AGATTTAAATTATGCCTCAAAATAAATTATCAATTGAAGAAAAACGTCTTAGGAAGAAATTGGCTGAGCGTCGTAGAATG

GCCAAAATAAAAAATGATCCAGATTTATACGCGCAATGGCTTGTAAAGTCAAGAGAGAGTTACCAAAGGAAAAAAGCTCG

GGGCACAGTACTACCAATGAGTGTTTTGACTCCAAGCCAGCAAAAGATTCGTAGGAAGAAAAGTAGGCAGAGTTCTCGAC

GATATTATCTAAAGAAAAAAATGGATAAGTTATCAGCAACCGATGAGCTCCCAATAAATGAAGAACCCATCTATAAATCT

GAGAACCGTGATCCTCTTTCAAATGTTCCTCAATCAGCTCCAACCTTAAACACCTACAAAGTAACAACAAAACATAGAAA

GATACTGCAAACTGTAACACAAGATGTTGAAAATGAGTTTAACATTATTAATCGTGTAAAAGATCATTCTCAGACAAGTT

TTTGTCTTCCACAGACTTCTAAAGAATATGACAATGTGGCTAATTTTGATACACCCGCATCACCGGTTAGTAGACTAAGA

CGAATAACAAGATCTCAGCGGCTATCGCAAGGTACTGAACGAACGAACGAACTAACATGCATTGGTAGTGCTTCCTCCAG

CATCTGTCCCGATAGTCCACAAAGTTCTATTAGCAACATATCAAATACGTCTCAAAAAAGTCTAGATTTATTCCCTAAAC

ATAAAATATCGTCAGTTGCAAAAAAAACCTCTCCATGGAAAGCTTTATTAAGAAGGATGAAATATAAAGCTAATAGGGAA

TTAAAACTGAAAGATCAAAAGATTCAAAAGTTGAAAATAAGTAATGAACGTATTAGAAGACAGTTGAACAGGTTAAAAAA

ACAAAGTAAAGAAGGAAAACGTTTAGAAAGAAAAATGTTGGTTTCAACTCCTAACAAAGATAACGTACAAGAAATAAAAT

ATACAGTAAAACAACAATTAATAAAAGATATAAAACTGTTTTTTGAAGATGATGATATTAGCCGCATGACATCTGGGAAA

AAGGAGTTTATTACTAGAAATAAAATAAAAAAGCAAAAGCGCTACTTAAATGATACCTTACACAATTTGCACCAAAAATT

TACTTTAAGATTTCCACAGCACAACATCAGTTACAGCTTCTTTTGTAAGCTACGAGCTACGTTTGCCTTTTGGGCCGTTA

TCCCAGATGCAAAATGCCGTGATACATGTCTTTGTGTAGAGCATGAGAACATGGATTTGGTTACCCTATCATTGAAAAAA

TACTGCATTATTAAAGAAAAATCATCAACGGAAATCTTAACCTCCTTAACGTGTAATTTGAGAAGTATAGACTGCCTTAC

AAGATGTTGCATTATCTGTAAAGAGAAACACCTGGAATATAAAGAGTTTGATAACAGTAAGCAAATTGAATATTTTAAGT

GGACCAAGATCAAGAAGACTTACATGAAAAATGGAAAAGAAATGAAAACTACACAAACCTTAAAGCAAAAAGTCGAAGCT

AATCCCAAGGATGTTATTAATTATTTTGAAAATATATGCAGCTCGTACATGCATCACTGCGCCAATATTATAGCCCAGAA

TAGCTACATTAAAAAACTTAAAGCTAATTTAAATTACGATGAGTGTTTAATCCACTGTGACTTCAGTGAAAATTATAATA

CCAAATACGCGACTGAAATTCAATCATTTCACTTTGGCGGTTCGCGCCAACAAGTAACCTTGCATACCGTAGTAATTTAT

TACAAAGAAGATGAGTTACTCCAGTCACAGTGCTTCTGTACGATTAGTGAGAGCTTGAGACATGACGCTGTAGCTGTTTG

GGAACATTTGGTGCCAGTCCTCCTTTATATAGAAAACACATTGCCTAGAGTAACGTCTTTGCACTTCTTGTCAGACTCAC

CTTCGGGTCAATACCGAAACAAGAAATTGTTTCATATTATATCAAAACTACACTGGCAATACCCAAGTCTACGAAAAGTT

ATATGGAACTATTCGGAAAAGGGTCACGGTAAAGGTGCTCCTGATGGTGTTGGAGGAACGCTTAAAAGGACTGCCGATAA

AATGGTGGCTCATGGATTAGATATACCTGATACGAAAACGTTTTTAGCTTATCTCAAAACAAATGTGTCAGGAATTATCC

TGGAAGAAGTTGCAGAATCTGCCATATTAGAAAAAGACATGTTAATCCCCCAAGATATAAAAGAATTTCAGGGTACTATG

AAGGTACATCAAGTAATTTGGTCAGCGAATACTAAAAACGTTTTGGCCATGAGGCGACTAAGCTGCGATTTAGGCAATTG

TTCACAAGAAGCTGTACAATGTCCGCACGGCAATCACATAGGCTTTTACCAAATTGATAGCGACCCGAACCTAAACACCT

CAAAGAAAAACATGTCACGGACATCAAAGAAGACCAGTCGGCCTTCGCTGGTCAAGCGTTCAAAAAACTGCGATCAGGAG

AAGCAAATTCAGCTTCCACAAATGCTAAATAATAGACAGACTATAACTGCAAATCAAAGTCAAGAATCATTACATAACTC

GGACCTGCCGGATAGTTTTTGGGATGAAGTCCCATCTATTGACTATGAAAATTTAATTACTCCAAGTATTTCCGAGGTAG

TTTTATCTTCGTTGCAAAACGATAAAACCTTGACAAAGGCTGCTGCAGCAGAAGATTTATCCGACTCCGATGACTATTCA

ATTTTCTGAGCCTTATAGAAATTGTATTTTGGTGTTAAAATTTGTATCATTATAATTTTCAAACAATATTTTAATCTGAT

CTGATCATCTGAATTGCATTTTACTTTGTGTTGTTCTTTTTAATTCTTTACTTTCCACTAAAGATATAGTTAAGGAAAAT

GTGATTATTACAAATCTCTTAGATAATTTTGAAAAGACTGTCATAATCTTAGTCATAATTCTAAGTCCTATTAAGCATTA

TGATTACAAATAGTTACTTTAACCAATTGTTTTTTTGTTAGAATTTTATCTTCTTTAATTACTTTTTAAAAGCTAATATT

TAATGAGTCTAAGTCATTTTATAACTATAAAAGAATGTAGGTAGGTTGGTTGATTTCAAAATATTTTTAATTTTGTGTCT

TTTACCTAGAAGTGATTAATTAAGTCCAAATAAATGAAACTAAGCCAAAAAGTTGTTTTTTATAGTTATGTTAATTTGTT

ATTTAAGCAATTATTTTATACTAGCTTTTGCCAGCGACTTCGTCCGCGTGGAATAGTGACTTCCGGCAGATCTTTTACCC

ACATTTGTAGTTCCCGTGGAATGGGAACGTCTCGAAAACGAGACGTTCCCGTTCCTGTATGAAGGTCGCGTTTAGAAAGT

GTACATTTCTAACATATTATTATAATCTTGTTACACGTACCCTGAATAACACTGAAAACGGTTAAATAAATAATATTTTA

TCAATATTAATTTTTTGTTTCACTAATTTCACAGTTTTTGATTGCTCTGGCACCAATTATGATTGCTCTGGTCGCAAAAA

TTTTTAAACTTAAATATTTCCATAACTGTTTATGTAAATTGAAGGCCAATAGTAAAAATGTACTATTTGAATATCATTAT

TTGTTATAAGATATATAATTACCATTAAATATCAATATCTGAAAAGCACCAGAGCGATCACACGAATACGCCAGTTTCGG

AGATTGATC

>Spodo_Contig_10_piggyBac

CCCTTTGTCTAGTAGAGGCATTATATTTGCCGACATGTTTTCATTGCTCCACAAAAAGAAGATTTGTATTAAAGAAACGT

TTTATCGTTTCGGGTAATAGAGAACAAACTAGTACGACGAAAAAAAATATAAAAATTGGTAACATTTATATGACATGTGT

CAAATAAATGTCAAGTAAGTTTGAATTTACAGCTTTTTCTGTTCTCAAGAGTAATCCGGCAAAATCTCGAAAAAAAACTG

TTATTTTGGTAAGTTTTCTTTTATAATCGTAAGATTTGTGTTTTTATGACAATCTTAGATAATTATTTTAGATCAATATG

TAATATTATTGGCGAAATCTGGAAAAATCACGTCGGCAGTATATTTGCCACTACCAGTTAGTACATTACCCGTTGTTGGC

AATATATATGCCAGTACCAGTGAGATTTATTTTTATTTACAGAATAAACAATGGAGCACCGTCGGTCGATAAACGACCGC

GAAATTGAACAGCAGTTGGAAGCCATGTTTGGTCTTCCAGATGGTACAGATTCAGAAGACAGTTTGGAAGAATCCGATAC

TGATGACCTTGTACAAATGCTTTCGGCTAACGATTCCTCTTTTCAACCTTGCATCGAACCAGACTTTGCAGACTCGTTGT

TGACGGCTAGAAGTAGTAGAAGACACGGAAAACATGGGCCAGAACAGGCAACGGATGAATGGGAAGAAGTTGACGACGTT

GAGAGTGACGACGAAATAAGTACAGGAAGCAAAAACAGAAGAATGGAGAATGGTAATATACGGACTGATAGTGAAGACCA

AATAGATACAGAAAGCAACATCATTCGGAATGAGACAAGTGAAGGGACAACAGATTGTGAAAGTGATAATGAAGTGGGAG

GAGGAGGAAACGTTAGAGTACTCCGTGCTTCAACATCTAGCGCCACCACTGCACGGCCCTTAGATTCTACATCTAATGTG

GTGACACGAGATGCAGAGGTGACTGCACCCACTTCCTTAGTTTCAGAAGAATCAGATTCTGATGAAGATGAAATAGAATG

GAAGAAAGTTGATTGGCTAAATGATCCGAATGTGCCTTCCTTCGACGAAAACGAACTTCAATCGCAGAACCATTTTCCGA

GTAGATCTAGACCAATCGCTTACTTCGAAGTATTTTTTGATAATGAAGTGATTGAAAACCTACTTGTTCAATCAAACTTG

TTTGCTAGTCAATCCAATGTTAGAAATTTTACTCAAATTAGTAAAGACGAACTCAAAGCATATCTTGGAATGCTAATACA

AATGGGGATACACAAACTACCGTCAATTGAAGATTACTGGTCTAGCAATCCAGCATTATGTGTCCCCGAGATTGCTGAAA

CCATGACACTCCAAAGATTTCAAAAGATTTCTAGATGTCTACATGTAAATGACAACGAACAGATGCCCAGAAGAGGTGAA

ACAGGTTTCGACAAACTTTATAAAATTCGCCCACTACTAGATCAAATAAACCAGAGATGTCAAAATAATGCCAGAAATAC

AAAATCTCAGTCCATTGATGAATCAATGGTGAAATTCAAGGGACGCAGTGCGCTAAAACAGTATATGCCCCTAAAACCTA

TCAAGCGCGGTTATAAAATTTGGGCTAGAGCAGATAGCAAGACTGGTTATTTGTTTCACTTCCAAGTCTATACTGGTAAA

AGTGACAATGTGGAAACTGGTCTAGGAAGTAGTGTGGTAAAAACTTTAGCCCAACCACTTATTGATGAAGGTTGCTCAGC

ACATATTAGCTTCGACAACTTTTTTAGTAGCTATGACCTTCTTCAGTATTTATATGACCACGGCATATACAGTACAGCAA

CTGCGAGAAATGATAGATTGGGAATGCCTGTTTTAGTAAAGAAACCTACAGGTCTAAGAAATTGCGAAGAAATCATGAAG

AGACAAAATAGAAAACTGAAACAGTTACAAAAAGGGCAGTACAAATGGCGAGTTCGGAACAACGTAGGGTTTTCAATCTG

GAAAGACACAAAATTAGTGACCATTTTGAGTACAGCCTTTCATCCAAAAGAAGAGGCTACATGTCAACGTACACAAAAGG

ATGGAAACAAAAGACCCTTTTCCTGTCCACGTGCAGTTGTAGAGTATTCGAAGCGAATGGGCGGGGTCGATAGATTCGAT

CAGCAAAAAGCCATTTATGATGTCGCTCGTCGAAGCAAGAAATGGTGGAAGCGGTTGTTTTATTTTTTGTTCGATGTAGC

AATCACAAATGCGTATATATTGTACTCAAAGAATTCAAGAGTGCACAACCCCATGAGCCAAAAGATGTTCAGGCTTACTC

TTGCCAGAGAACTGGTCAACAATTTGACATTTCGGAAACGAAAATTTTCATCTGCTCCCAAATTCTTAGCAAAGAAAAGG

AAGAATAGTGCAGAGCCAGTTACTCGCCAGAAGAAACTTTTTGGTGTTCCGGAAGATATTCGTTTCACTAATGTTGGTGA

GCACTTACCAGAAGGCATAGAAACGTACAGGCGCTGCCGAGTATGCAGCAGCGCAACTAAGAATAAAAGATCAAACATAC

AATGCGGCAAATGTGAAGTGCCACTTTGTGCTGTTCCTTGTTTTAAACAATTTCACATGTGTGCTGACCAGTAGTGGCAA

TTATATGGCCAACATATAAAAAAAATAAAAAATAAACAAAAAAATAAAATATTCTTCTTTATAATACATAATTACATAAC

ATATCTGAGTTTCATTACATTTGCACGGCTAGTTTCCTCTACCAGTTAAAGGG

>Spodo_Contig_4_Gypsy

TTACTAGACGCCGGCATTATTAAAGAATCTGAGTCAGATTATGCCTCTCCTGTTATCTTAGTGAAGAAAAAGAACGGTGA

TAGTCGGTTATGTATTGATTATCGTGCTCTGAACTCAATCACTATAAAAGATAGGTATCCGTTACCAAACATTGACGATC

AAGTTTCAAAGTTGGCTGGTATGAAGTACTTCACAAGTCTCGATATGGCCCAAGGTTATCACCAATTACGAATTGCACCT

GAAGACACTCACAAAACTGCATTTATAACACCTCAAGGTCATTATGAGTATATGCGCGTTCCGTTTGGTTTAGCCAATGC

ACCCTCAGTTTTTATGCGAATGATTAGTAAAATAGTTGATTCCATTCGTTCCCAGACGTCGCAAACTGACAGTTCTAATG

GTGAAATTCTTGCATTTTTAGATGATTTATTACTACCCTCACCTGATTTTGGTTCTGGTATAAAAATGTTAAAATTGGTG

TTGCAAAAGTTTGAATCAGAAAATTTAAAACTAAATATGAAAAAGTGCTCTTTTTTACAACAGAAAGTGACTTACTTAGG

ACATGAAATTTCCCATGAAGGTGTGCAACCGGCAGAACTAAAATTAATTGCAGTTTCGCGGTTTCCTGTACCAAGTAATG

TACATGAGGTACGTCAATTTATAGGCCTGTGTAGTTACTTCCGAAAATTTATATATAAATTCGCGGTTATAGCTCGTCCT

CTAACGGAATTAACTAAGAAAAATGTATCGTGGGTATGGAGTGATAGACAGACCGATAGTTTTAACAAACTGAAAACATG

TTTATGTTCTAAACCAGTGTTAGCTTTGTATGACCCTACTTTGGATATAGAAATTCATACAGATGCATGCAAATTAGGAA

TAGCAGGAATTTTATTACAAAAGCAGTCCGACAACACTTTGCGTCCTGTTATGTATTTTAGCCGAGTGACGTCTAGGGAG

GAAACTATGTATCATAGCTACGAGCTCGAAACATTAGCGGTCGTAGAATCGTTGCGTAAATTTCGGATGTACGTAGTAGG

TAAACACGTAAAAGTAGTTACCGACTGTACTGCCGTCCGAGCTACCCTCACTAAACGCGACATTATACCGCGTATTGCGC

GATGGTGGCTTTTAATTCAGGAATACGACATTAGCGTAGAATATAGACCGGGTGAACGAATGAAACATGTCGATGCGTTA

AGTAGGAATCCAATCGATGCTGTAAATATTAATCGTTTGGAAGTTACTGATTGGTTTTATACAGTTCAATATCAAGATGA

CAAATTAAAAAATATTATTGACAAATTAAAAGCCGGATCAGCTGAACGGGATATAATAAACAATTATGTGTTAATAGATG

ACAGATTATATAGAAAGACGCTCGATGGAAATCGACTTGTAGTGCCTGGGGCAGCTAGGTGGAAAATACTTCAAATGCAT

CATGACGAAATAGGACATGTAGGACTAAAACGATGCACGGATTTAATTAAAAATAATTTTTGGTTCGCTAAAATGACAAG

ATTCATTCGTAAATATGTAACCGCTTGTTTAAGTTGTGCATATGGAAAGGGAGATCATGGTAGGAAGGAAGGCATGTTGC

ATCCGATTCCTAAACCAAATGAACCGTTTAGAATGATTCATGTCGACCATTTAGGCCCGTTTTGTAGGACAAAGAAAGGT

TATGAATATATGTTAGTTATAACTGACGCTTTTTCAAAATTTGTTATTGCAGAGTGCAGTCGATCAGTTAATTCAGTTGA

AACAATTAGAATACTTAAAAGAATATTCGCATTATTTGGCTATCCGGATAGAGTAGTAACTGACCATGGCAAGGCATTTA

CTAGTAGGTATTTTAAGAAATTTGCAAGTGATAAGCAGTTTAAACATACGTTAAATGCAATTGCGTGCCCTCGTGCCAAC

GGGCAAGTTGAGCGCACCAATCGCACAATATTGAATGCTCTCCGTGCATCTGACCCTAGTGAAACCGCTAATAGCTGGTC

AAACCGTCTACCAGATGTAATTTGGGGGATAAATAATACTATAAATGATACTACCGGGTTTAAGCCTTATGATTTAATGT

TTGCGCGTAGTGGTAGGCCAGTGTGTGATGTATCCATCCCAGACCAAGTATGTGAATCCACTCAAGCGAAACGTAGCAAG

GCCAGTACACGTATCAATAAAGCTAGCGATAGGATGAAACGTAACTTTGATAAACGTCGCAAAAAATGTCATGTATATAA

GAAAGGAGAATTAGTATTATGGAGACAGGCACCCACTAGTAGTGCTGCTAGGGTCAACACAAAGTTGGACGACTTGTATT

CAGGCCCCTATATAATAACTAAAGTGCTTGGCAATGACAGATACAGGATCAGGAGCATTAAGGGATTAAGAGGTTACAAA

AACTTTACGGGGCTTGTTTCAGCAGACTCTCTGCGACCATATCGTAGCATAGCTCCTGTATCTGACAGTGCCAGCAGTTC

TGATGACCAACTAGAGACTGAAGATTTAATTGACCTATTAGAGAGTTAAAGCTAATACAATCTATGTGCTTCATTGCGGG

AGCAATTGTTACAGGATGGCCGACTGTAACGCCACGCCACGTGTCCAGCCACGCCAAGCTAAGCCAAGCCAAGCCAATGC

CACGCCAATGCCGAGATTGTTAGTCAAGCCAGTTAGTGACGTCACATCCCAACGGCCGCAGGGTATAAATAGTCGAACCG

CTCATTTTGTGACACATTTGGCCGTTGTATACGAGATAGGTCGAAACTCTACCCGATTGTGTAAAAAATATATTTCTTTA

ATAAATAGCTGTGATTATTCGTAAGGTGTATAAGTTTATTATTTCATCTGCGTTTCACTCAGAAGTGGGATATGAAGCGT

AATCGCCTTACTGCATCTGTTGGCTGTGGTGTCCGTCCGCCTGGCTGGAGTGAAGAAGAGCAAGGCCACATCAGTGAAGA

CGAAGAATTCCCACCGCCACCAGCTAGAAAACTTCTTAAAAGTATGCTAGAAAACGAAGTGGGTAGGGCGATACAAGAAC

TCAATGACGAAACCTTGCCGCTCAACCAATTTAAAGGCCATCGTGCGGCATCCCTTATCCCAGAATTCGACCCTGAAGGA

GAAGATTGCACAGTATCGGTATGGTTGAAGAAAATTGATCAGTTGGGAGACATACACGGCTGGAGCGACAAAGTAAAATC

TTTTCACCTTCAAGATAAACTACGTGGTCAAGCGCGTAAGTGGTACAATAGACTCGATGATTATAACTACTCCTGGGAGG

AATGGAAGTGTATGTTAGTAAGAGCATTTCCCAAACACCGTGACTTCAGTGCTTTGTTAGAAGAAATGATGAACAGGAAG

AAGATGCCGAACGAAACCATGACAAAATATTACCAGGATAAAATTGCCATGTGTTTTCGTTGTAACCTATCTGATACTGC

TAGCGTTTCGTGTATAATCAGAGGGTTACCGTCTACCTTACAACCAAATGCAAGGGCCTTCCAATGCCAGCGACCGGATG

AACTATATGAAGCCTTCCTCTGTGCCCTTGATGATTATCGCCCACCGATAATTGAAGCACGTGACAAGCCGAACAAGGAT

GCTAGACAGCTGCCGAACCCAGATAAGAAAGTGAACCTTGAGACTGACCCCTGTCCGCGTTGTAAGAAGACTGGCCACAT

TTTACGAAATTGCTCATTGCCTGACCAACGCATTTGTTTCAAGTGTGGTGTACAGGGTCATATTGCTACCCGCTGCCCTG

GCTCTTCAATTACACAGCTTAAGTCGTCTGCCGGATCGAACAGCAACATCAAGGAAGTTAAGCTAGTGCAAGATTATAAT

GACATTTACAAGAAGGTCGTGAAGGTAAACGGGATTTTTTTGAAATCTTACATTGACACTGGAAGTCAGGTAAATGTTAT

AAATGCAGATATTGCTGATATTTTGGCACTCAAGGTTATACCTAAGAGCACTATCTTAAAAGGCTTTTCTGGTGGTTTAC

TTACGAGCCGCGGAGAAGTAGAATTTGACCTAGAAATTGATGACATTCATTTTAGATGTTCTGCTCATATAACAGAAGCC

GACATGCAGGACATTCAATTATTAGTTGGTCAGCCAGTTATCAACGCTGATCATGTGTCATTGGTGGTGACCAATAGGAC

TGCTTCGTTAAGATTGGACCCTGACCCTTTTGCTGAACTTCAAGTAGTCGAAGATATTCAGAAATTTAACGTCGTGACAT

CAAACAAAGAATCCTTGCCGCCCGGTACCTCCCTCATCAAAGTCCGAGTGCTGGGCAATACCGAGGAAGATGATGTCTGC

ACAGCGCCACGCCACTATGAACTGCATGGAGTTTCCTATTCGCTACCAGCTACGTTACTTCGAGGCGGAGAGGGCTTCAT

GAAGGTTATCAACACCGGCACTAAAGACATCGAGTGGGAAGCCGGCCAGGTCCTCGCACGAGCAGTTAGCTGCGACCAAT

CTGCGATTCAGCCAAGCCAGGTGAGTTCAGCCAAGTCAATGTTATCTTGCTTGCCAAGCATTTTGCCAAATAAGCCAACC

GGTTTAGCCACTAGTTTTTCCAATTCGTATGGCCTTACAGTCCCGTCTGCTACGATTTTAACAACTAATAAAATCACTAA

TGATACTATAGGCGGAGTATCTATATCTGAAATCAATATGGGGAGCTTAAATAAAGACGAGCGTGATAGTTTAATACATT

TATTATTACAATATAAAAACACCTTTGCAAATGACACAAAAGATCTCGGTTGCACTAATTTGTTAAGCATGAAAATAAAG

CTTACCACTTCGCAACCAATTTGTCGTCAACCTTATAGGTTATCGCACACTGAACAACAAATAGTAAATTCAAAAGTTCG

TGATTTACTAGACGCCGGCATTATTAAAGAATCTGAGTCAGATTATGCCTCTCCTGTTAT

>Spodo_Contig_5_piggyBac

CACGTTGAACGCCGCCTTGATTTTTGAGACTTTCTCCTTGGGCCATGGGGGACACCATTTGGGCCACACACAGTTGTTCC

GTTCGGGCCGTGGGGGACACCGTATGACCGCGAGCTAGAGGTGAATTCAAACTTCGCTACAGAGCCGTGGGACACAGATT

TCAAGCTAAAATGTATTGTACGTGACCCCCACGGCCCTGTAGACAAATGCACTCTACTTGGTTTTTAGTAAAAAATATAA

CTTTAAAAAATAAGTTACGTAATGTTGGGACAATATTTTCACTGCAAGGTTGAGGTATAGTATATTATCTTATTTCGTAC

AAAATAGCTTTTTATTGTGTTTTAAATAGTTCTTTTGAACAGAATAAACACACATTATTACTATTTAGATATTGAATTAG

CCAGAATGTAAGGTTTCACGATGAGCATGATATAAGAACATAGTTGAATTGACTTTATCGGGGACACTTTTTTGAATGAA

ACAACATTTTTTTTCTGTGAATCAGACGTATAATTTATTATTTGAAGTTTAAAGAAAGCAAAATGAAATCAGACTTTAAA

TTGAGATCAACGTAATTTATACATATCTAATAATTAGTGAATTTTATGGAAACAGTCAAAACATAAAAATGGTTTATTTG

GACAAGTGTCGCAAAAAGTAACAACTTTTTTTGTTTTATTTTTGGCCAACTTTCGCCCCATCACTTGTGTATTTTCTTTA

TAGCACTGCTGACATGTTCTTCGATTATTTTTACTAGAACCGTCTTTCCTTGCAAGTTTATGTCTCATTCTTTTTGGCCT

TGTAACAGCATCTGTTGAAGTTTCTTCACTTCTCATCAACAAACCTTTCACCAGCTGTTTCCTAAACTCAACTATTTGCA

TTTTCGTTCCAGTTACTATCTGATATAATATTAGTGCATTTACAACCGCAGTATTTAGGAGTAGGTTTATGCCAACTTTT

TTATACCACTTTACAGATTTCCTTAGTGGCGAGGAGTATGCCGCCATCTGGTCGGACAAATCCACTGCTCCTTTCGCTCT

ATTATAGTCGAGAACGATTTTCGGCTTCAAAACTGTTTTTCCTCGCTTTGTGATTGTCATAAAACGGTCTGAATGTTTCG

TCGAAAGAACAAGCACGTCCCTTTTATCTTTCCATTTCATGATTGTTATCCCATCGTTGCTTTCTCTGACTATATGCTCG

CCTCTCTTCAATTTAGCAGTAGTTACTTCTTTTGGGATATTCCTTCGGTTTTTTCTTATCGTGCCAACTAGATGCGTTTC

ATTTTTCAATAAATTCCTAGCTAAATCAATACTTGTGTACCAATTATCTGTGTACAAGGTATGTCCCTTGTTCAATAAGC

CACTGCAAAGTGACATTACTACGTTTTCTGGCGTAGTGTTAACGTTATCATTTGCTTTTCCTGCATAAATATTAATTTTG

TAAGTGTAGCCCGGTAGTGTGCATAATTTAAACTCTTTAATGCCATATTTGTGGCGCTTCTGTTTGTTATACTGTCGGAA

AATGATTCTGCCACGAAATGGCACCACGCTTTCATCGATACATAAATCTTCGCCTGGTGTGTAATGCTTTGCAAAATTGG

TGTTCATGGTATCAAGTAAGTGTCTAATGCGGTGTAATCTATCAGATCTGTGAGATTCGTCATGCCGGGAAAGATGAAGC

ATTTGCAGAAGCAATTCAAAGCGATTACGAGACATAATTGTTGATGCAAAACGGTGACCTGTTATTTCGTCCTTTGACCA

GTAGTCGGACAGTTTAGGAAGTTTCACCAATCCCATAAACATAATTAATGCAAAAAAGCTTTTCATCTCATGCACATTAG

TAGGTGACCAATTACGTATTCGTGCTGCTTTTGAAGCATTGCGCTTTGTGATGGTGTTAATAGCGAATTGATTCGTATCT

TCAACTATCTGCCCAAAAACGTCATCCGGAACCAAAAGGGAGTAAAAATCAATTGGTGATTTTTTACTCATAGCCAAACG

TAGATAGGTAGGCAAGCCAGGATTTTCCGTAAACGGTATAATACTTCTCTGGTTACCTGTTGGTAGAGTCCAAGTGCGTT

TACGCGGCGTCGTCTGCCCCGAGGTAGCTGGAACTGGACGCTCTTCGGACTCGCTCTCTTCACTATCTATCACCATAATT

CGACGACGGTTACGCGAAACTGGATGAATATTGTCTTCTTCATCTGAGCTTATATGGAAACTATTATTCAACAAATTAAT

TTCAATTTGATCTAATTGCGATAAATCGTCCTGACTTATATCAAAATCTTCGAACATATCCATTTCAAACTAAACGTATC

AAATAAAACTAGAATCAATTTACGTAACAAACGCAAATTAGCGAAATAGTAGCGATATAACAACGTACAGTTGGTAACAA

CCTCAGTTTGTCACTGACACAAGGCGCGAGCGCGCGGGAATCGCAACACGATTGGCGTCGGAAATGCACGAGGATGACCG

ACACCCCGACCCCCGCGGCCCCCGCAAGCGAACAAGCGAATTATTTACTGCTGAGGCCGTGGGGGACAACTGTTGAGTGA

CAGTAGTATTTTAACGAAATAGAATCACTAATTTTATTTCTACAATATATTTCATATTATTTACCACATTTTCTTGCCTA

TTAATTAATAAAAACGTATATTCGAAATCGGGTGAACAACGATTTTTAACCTGGCCCCAACCAGAAAATCAGTGTGGCCC

ATACGTTGTCCCCCATGGCGTTTAACGTG

>Spodo_Contig_23_piggyBac *°

NCCCTTCTTTGCATGATGATGGAAATTTCCATCACAAACATTTACTGGAAAAAAAAAGTACACAAAGCAGGCGTACTGTA

AATTGTTTGTTGTTGGCAACTTTTTACACGACCAAGGCATGGAACGAAACGCAGTTGTCAAAAAAAAAAACAAACGTCAC

TGTCAATTACAAGGCCTTGAACTTGTCATTCAGCTATTTGTTGTGGTTCGAAGAAACGAGTGAATTTATAAATTGTTTTA

CGGAACTTATCTTAGATTTGTCCACTAATAAAAGTGAGAATGAGTGAAAAAAAGGTTTGTTTTTAGTGTTTCTGTTAAAT

TATGTGCAATTTATATTGAAATACCCTGAATAAATTTCTCAAAACTATGTGACAGAAATTTACATCACTATGCAATAACT

TCACTTGTAATGTATGGCGAAAATGGTTTTTAGTAATTTTTGTTTTTTGTAGGGAAACTTGAAAGACAGAGACATTGAAG

CTTATTTGAGTCAGTTAGAAGACGGTTGGCTCTCGGAAGATGGCCTTGATAACGAAGATTCCGACGATGAGTACCGTGAT

GCCGATGAAATTATTAGAGTGTTGCAGGAGGAAAATGATAATGATGATGTGGAAGATGAAAGTGCAGACATTGATCCTCC

TCTTGTAGAAGAGGTCACCACAGAAGACCAGGAACCCTCACACTCATCATCTTCATCCAATTTTCAGTCTTCATCCAGTT

ATTTTTTATTTGACAAAAGAAACTTGATATGGAAAAAGAGGAATCTTGTGTATGATGCAGATAAGATTACTTTCTTGGGG

AGTTCTGCTGCTCCAGATGAAATATCACACCTGGAAACCCCATATCAGTGTTTCAACCATTTCATAACAAAGGATTTTAT

GAATAGATTAGTGGAGCAAACTAATTTATATATAATACAAAGTAATCCCAATTCATCTATAACATTTACAGAATATGATT

TATACAAGTTCTTTGGTATCCTGTTATACATGTCAGTACAAAAATTTCCTAGCACAAGATCTTACTGGTCACCAAAGTTT

GGTTATGACCCCATTTCTTCAACAATGCCACTAAACAAATTTGAAAAAATAAAATTGTCATTGCATTTCAACAATAATGA

ACTTCATAAACCTATAGCCCATCCAGAACATGACAGGCTTTTTAAAATCCGCCCTGTAATCAAGCACCTCAATGAGAGGT

TTGCTACAGTGCCAATGGACCAAAGACTTTCTGTGGATGAACAGATGTGTTCCACAAAAATAGGGCACTTCTTAAAACAA

TATCTGCCCAATAAGCCCCACAAATGGGGCTTCAAATTATATGTTCTGTGTGATTTGATGGGATATGCCCATAAGTTTGA

GGTATATTCTGGACAAGAAAATTCAGAAAAACTCTCCAATGAACCAGATCTTGGTGCAACAGGTAATGTTGTAGTGCGTT

TGCTCCGGGGAGTGCCAAGAAGGCTAAACCACATAATATACTTTGATAATTTTTATACCTCTTTGCCATTGGTATATTTT

CTAGCTAAAGAAGGAATACATACTGTGGGGACTGTGCAACAAAACAGGATTCCAAATAATAAACTACCAGATAAAAAAGA

TTTCATGAAGAAATCGGTGCCCAGAGGTAGTTATGAAGAGAGAGTTTCTATGCTTGATGGTATAGATATGAGTTGTGTAG

TATGGAAAGATAATAAGATAGTAACCCTGCTATCTACTTATGCTGGAGCATTGCCTGTGGCTAAAGTAAACAGGTATGAT

AAGGCAAAACGAGAAAAGATTGATATTACTTGCCCATTTATTGTCCAAGAATACAATAAACACATGGGAGGAGTAGACCT

CATGGACAGCTATTTGGGTAGAAATCACATAACACTGAGATCCAAAAAGTGGTACCTACGTATTTTTTTTCATTTATTTG

ATCTTGCAGTTATAAATGCTTGGATTGTCTATAAAAAAAATGCTCAGAAAAGGGGTGAACCTAAAAATAGTCTGCTTACA

ATGAGCCAATTCAGAAATGAATTGGCCTTTGTGCTTTGCAACAAAGGAACCATCAGAGATGCTAAAAGAGGGCGTCCTAG

CAGCAGTACCCTAGAAGAAGAATTGCTGACAAAAAAAAGAAAAGGTTCTCCAGCTCCTCCACCTCCTAAAGACATAAGGA

GGGATGGTTCTGAACATTGGCCAAAAGTAGGTGGGTCACTCCGGTGCAAATATCCTAAATGCAAAGGTTATTCCACAGTA

TCATGCAGTAAATGTGGAGTTAATTTGTGCCTAAATAAGAATAATAATTGTTTTCTAAATTATCACCAAGAATAAAGAAT

GATTAAGGATATTCTAGTTTTATTTATTTAGTACTGAGCTAAACATGCAGAATTGCATAATGATGGATATTTCCATCATA

CTATTTTTTACTTATTTCGTATTATTTATGTATACTAGGATTTTTTTAAATATTTTTCCCTTAATTTAGAGCATGAATTA

CATCACCCTGATTTATTTCACAAAAATCTGAAAAATCATGCAAAGAAGGGTTAAAGCGTTCGGGAA

>Spodo_Contig_13_Harbinger *°

TGGTACGTTTACACGCTTGCCAAGCCTTGGCAAGGATCGACAATCGCAAGCATGTAAAAAGGGCGTTTGGCTTGGCTTGC

GTGGGCTTGTGCCTGCTTGCTTCCGATCCAGTCGAGTGTCGGTTTTTTTACGCAGATCAAAGGGCGTTTGCGGCAAGCGC

TTGCGATGCATTTACATGTTGGTCCGCGTTTAGTAGCCATTGTGATCTCGCGGCGAGTACACGCTTGCTTGATTTTACAA

AAAATAAATAAAAAATGAGTTGCTGGTCGAATGAGGAGAGTCTTTCGTTTCTGGAACATTACCAAATGGAACCATGCATC

TGGAATCCGAAAGATGCAAATCATAAAGATAAGAAGAAACAAGCAGATGCATGGATTCGTCTTGCTGAACTTACTGGAAG

GCCAGTAAAGGAAATAAAAAATAAAAAAGAGATATTGATGACTACTTTTCGCAAGCACTTGAAAAAAAAACGCGAATCTA

TGCGTTCTGGCGCAGGTATGTTCCTACGATACATTTATAATTATTTTGATAGTCTGCTTAGCAAGGTGTAACTTATGTTT

AAAAGCGTTGGTTAGTTTCTCGATTATTCCGCAACTATCTCGCTCTAATTTTCGATGTTTTTATTTTATTTTAGGTGCCG

AAGAAATATACAATCCAACTTGGTATGCTTACGAAACCATGGAGTCATTTTTACTACCAGTATATACTTGCAATGAAGGG

ATAAACACCGAAACAGTAAGTAAATAAATATATTTATCTATACTTATAATAAATCTGTAGAGAGGTCAATTCTGTACATG

AAATATATTTCCAAAATAACTATCAGGGGGTGATTAGGGATCGATACTGATGCCAAAACTGCAATCAGTAAATTTTTTGT

CTGTCTGTCTGTATAACCGTTATAGAAACAAAAACTACTCGACGGATTTTAACGAAACTTGGTACAATTATTTTTCATAC

TCCTGAGCTGGTTATAGTATACTTTTCATCACGCTACAATCAATAGGAGCAGAGCAGTGAAGGGAAATGCAGTGAAACCA

AAGTTATACATGAAATACTCTAATAGTAAGCCATCGCGCGTGAATACTGAATCATGCTATAAGGATTATCTACACTAATA

TTATAAAGAGGAATACTTTGTATTTTTGTTTGTTTGTTTGTATATAGTAATATTATAAAGAGGAAAGATTTGTTTGTTTG

TTTGTTTGTATTGAATAGGCTCCGAAACTACTGGACCGATTTCAATAATTCTTTCACCAGTTGAAAGCTGATTTGGTTCT

GAGTGACATAGGAAGTAAGGGGGGAGGGGGGGGGGAGCGTGCGAAGCCGCTGGCAAAAGCTAGTTCTAAATAAAAAAAAA

CACCGTTGGTTATTATACCTACATTTATTTACACATTTTTATCAACTTATCCAATCCTATTAAAATATGAAGTAAATTCT

TCCCTGACTTCTCTGGCGTTCACCGGAGATCTTCGTGGTATTTGTGGTAAATTTTGCAATGCATTATATTCGTCGTTATC

TCTACGCCATGAACCAGGGGTAACCATATTTCCACTACTGTCATATACGTCAAACGAACTCTGAGGAGTATATCGATTTG

AAGAACTTTTACTTGTTCTCAAAAAGTTATGTAGTAATACACAGGTCATCGTAAATTCCGTTACAGTTTGTGGTTCTAAA

TTGATAGGGCGTCTGAATATTCTGAAAACTGAGGTTAAAATTCCAAACGTATTTTCAACTACAACTCGGGATCTAGATAA

ATTTTGGTTAAATAACCTTTTTGGTGAGCCTACTTCATGGTTTCCAGGGTAGGGTTTCATTACGTGTTGACTCAATGCAA

AAGCACCGTCAGCTAAGAAGACGTATGGAATATTAATATTCGATCCTGGAAGTGGACATGGTGGTGGAAAATCTATCTCG

TTTCTAATTATTCTTTTGCATAGTAAACAATTATTAAAGACGCCGCCGTCACTTATTCTTCCTTGACTCCCGATGTCTGC

GAATACAAATTTGTATTTGCTATCCACTAAAGCCAAAAGGACAATGCTAAAAGTTTTCTTATAATTGAAATACTCTGTTC

CACTATGAGTTGGGCTTTCAATGACTACATGCTTGCCATCCAATGCACCTATGCAATGTGGAAATTTAGTCCTGAATCCT

TCTTCTATATTAAGCCATCCTTCTGCTGTGGTGGGCATCTGTAAAAAGAAAGTAAATGAAGTGTAAGTTTAAGTATATTA

TTTTTTTGCAGCAATCAGAACAAATAGAAGAAGATACAGATCAGACAGAGGATGACCCTCTGATAAGAGTTAGTACACCA

TCCAGACCACCATCTAGATCACCATCCAGACCACCATCCATACAAGCTGTTACTAATAAACGTCGTCGCCCAGCTTCTGA

AACGGCTGAAAAGCAAATGGCCGTGGCTTTTGGACAATTAACAAATGTTTTGGGTCAAAGGCAGAATGAAAATATACCTG

CTCACAAAGACGATGATTGCGATCTATACGCAAAATTATTGGCTATAAAGCTACGTGAACTGTCAACTGATGAACGAAAA

ATTATGATGTACCAAATAGATGGGTTGTTTATAAACAGAATCAGTCAAAAATCAAATGAGCGTCATACACCATACCCTCA

ATATTATTCTCGACCGTCGTCAATGGCTGGTGCATATTCAACACCATCTCCTCAAGTCATTCCGAGTCGGCCAACATCAG

TTAATAGTGTTTATTCTGAACCTATTCATAACAACCAAAGTTTTTTTCCCTCTCCAAACACACAAACCTCTTATTCAGCG

CCAGACATGACAAAACATATACCAACTCCAATAGCTCCAGCTCCATCTACATCAACAGCACCCTCGTCATCTATTCGTAT

CATATCGGACCAAATCATCAACCCTACCCCACGTGAAACCAACATTATAAACATGGCTATGTTGAATGCTTTCGAAGATT

TTGATTCGTCGACATAATTCTTATTTAAATTAATAAATAATTAAACAATAAAGCAGTCGGCTAACATACATTCATAGAAC

AGATTTTCTATGATTCTATTATAATCTAAATAAATGTATTTAATTAACATAATGTTGTGTTTTATTCCTACCTTAACTAG

ATCCTTTAGAATTTCATTAAGAGCTCGGCAAACTTCACGAATGATTATTGATATCATAGGGACAGATACTTTAAACAAGT

AGTGAAGGCTTTTGTAAGAATCACCAGATGCCAAAAATCGTAGCGTAATGGCAAGACGAAATTTGGCTGGTATAGCTTCT

CTGAAGTCTGTATCATGCTTGGATATCATTGGTGAAATTTTTTGCAGTAAAAACTCAAAATCAGAATAGCTCATTCGGCA

GAAGTTATCAAACTCTCCAGATGGTTCATATAAAAGTTCATTAAATTTCTCTTCTATAAATTGTCTGTAACAAATAAAAT

ATATCGTAAGTATTGTGGCATAACTCAACTGCAACAGATAATGGTTAAATCAAAATAGTAGTTACCTGGTACGATTATGA

TGAATCTTTTTCATCCACCATCTTCGCTGTCGTCGAGCTCTCCTTTTTTTTATTTCACTTTGCTTACACTTCAAATAAAA

GTACGTTAAACCAAATGCAATAGCCGTCACAAGTTCGGTGTCCGCCATGCTTGGATGTATATTGCAGACTGACGCATTCA

CAAGCGTATAAACGCCTTTCAAACGCCAAACGAGATTTGTGGAGGCTTGGGACTGTTTGCAGTTTGCCAAGGCTTGGCAA

GCATGTAAACGTAC

>Spodo_Contig_14_Gypsy *

TATTGGTTCGATAAAATGAGTTCTACTATTCGAAGATTTGTGGACAACTGTGTGATATGCCGAACATCAAARGGAAGATC

TGGTGCCATTCAAGCCCAACTGCACCCAATAGAAAAGCCGTCAGCGGCTTTTCAAGTTATTCATATGGATATAACTGGGA

AGCTGGGAACTCCAGATGATCAACAGTACGTTATAGTCACCATTGATGCTTTCTCAAAATATGTTCTGTTCTACTTCTCT

AGCAACAAGAATCCACACAGTACACTAGCCGCTCTGAAACGCACTGTACATCTATTCGGTGCACCAATTCAAATAATAGT

CGATGGAGGCAGAGAGTTCCTTGGTGAGTTTAAAGACTATTGTGACCACGCTGGAATAAATATTCACGCCATAGCCCCAG

GAGTTAGCCGAGCGAATGGTCAAGTAGAGAGGGTTGTTGCAACTCTCAAAAATGCATTGGTGATGATTAAAAACTACGAA

ACAGAGCAGTGGCACACTACACTTGAGGAACTACAACTAGCAATGAACTGTACAACTCATCGTGTTACTGGAGTTGCTCC

GTTGACACTTATTACACAACGGAAGCATTGTGTACCTTCTGAGCTCCTCAGCATAGTGAACATCGATACTGAAACCGTAG

ATATCGAGGCACTTTCTCAACAGGTATTACAGAAAATGTCTCGTTCAAGCGAACAAGACAGACAACGATTTAATCAACAT

AAAGCCAAAATTCATCATTTCCAAATAGGAGACTATGTGTTGATTAAGAATAATCCTCGCAATCAAACATCACTCGACCT

TAAATTTAGTGAACCTTATGAAGTGTCTAGAGTACTAGAAAATGACCGCTATTTGGTGAAGAAGGTGGTCGGCAGGGGGC

GACCTCGCAAGGTAGCTCATGATCAGCTGCGCCGCGCACCACAACCCGGTGAACAGTTGACCGTATCGGCGGGAAATGGA

TCACCAGGTCATCAGGAGTCTGCTGCATTTGAGCCCATCCCTTCGACTTCACGAGACCCAGAAAACCACGAGCTACAGGG

TGAAAACAATTCACCAGAATAAACTGTAAGTAACTAAATTCAATACCAGTAACTTCAAAGATTTCCATGAAACATATGTA

TCTACGAGAACCTACGAGAACCCACGAGAACCTTCGAGAACCTCCGAGACACTCCGAGAACCCTACGAGACCCTACTACC

TCATTAATACTGATATCACTAACCATAAAAGGCATTGTGTGTTGGTGTAATAACTAACATAAGCAACACAAGTTGCTCAC

AGATATCCGACTCAAAAGAAGTCTAATCTATTGTTGCAGGTACAAATAAGAACAACAATCAACGAGGCTGGTGCAGAGCT

CGCACCGCCTGTCAGTATGGCCGTATCGGCGCAATAACGATAATTAGTCAATTACAAGAATCAACAGTTAATTAATTAGT

TTAATAATTTGATTGTGCCAACACTAGTTCATAAGCAATTAATTAATATTGTTTGATTATTGCAATGTAGAAAGCTTGTA

ATGTAAATAATTCAATACTTAACCTAAAAATTATGTACACGTGCTTGCCACACTATAAATACAGCCGCACAAAGCGTGCT

AGGGCAGTCTTGCTCGAGCCGCCGAACTGTACGGACCTATGTAAATACGTCGTTACGCTGTCGGTTGAATAAACGAGACT

TGCAAGTATACTTGTGGTGTTTTACTAAGAAGATTGAGGATGAGTGATCACCGCGAAGATAGTGTCAGTGACGTCAGCTG

TGCTGACGTCACACTTTTTAAAAACGGAACCGAGACAGTTCCTCCGTCATCAGAAGTGGGATCACAGGAGATCTCTACTC

ACACCACTTCCTCATCAGCTCGTTTACCACCTGAGCAACTGGAACCAGCCAACAACCAGCGTCGGGAGCCTGTATTGGTC

TCCAACGCATCAACGGAAGCCATATCTCAATCAAGACCACATACATACCATGATCAAGGGATGCGGATGGATGGAATACC

ACCACAATTCCTGATGCAGATGATGGAGATGATGCAGAGAATGTCAGAGCGTTTGTGCGCTCCGCCTACTGAATCAAAAA

TAAGGATCAAAGACATTTACCTGCCATCATTCGACCCGGATACTCATGTCGGAGTACGTGAGTGGTGTCAACATATAGAT

CAAGCCATTGAAGCATATAAACTGAGTGATTTCGATGTTCGTATGAAAGTCTGCAGCCTGCTGAAGGGCCGTGCTAAAAT

GTGGGTCGACGATTGGATGGTGAACAGTAGTACCTGGGAAGAGTTGCGAAAGAACCTCATCACTACATTCGAACCTGAGA

ACAGATACTCCAGAGACATTGCTATGTTCAGGGAACATGTTTATGATTCCTCAAAGGACATTGCCGAGTTCTTGTCGCGG

GCATGGATCCTATGGAGAAGAGTCACCAAGGACAAGTTGGGAGATGAGCACGCAGTGGAGGCAGTGATTGGATGCGTTAA

TGATGAACGATTACGCATCGAGTTACTGAATGCCAGAGCAACCACTGTTCCTGAACTGATTTCAGTGGCAACATCTATAA

GGAAAAAACGTTCTCACCCAAGCTCAAATTCCACAATGCCTAACAAGCGTTCACGATTTGCAGACAGTCAACCATCAACA

TCTAGCTGCCGCATATGTAAAAAGACAAACCACTCAACTAACGATTGCAGATTTAAACCTCCGGAGGCTAAACCCGAAAC

TGGTAACAAGGACCCTAAAGCACAAACCATACCTACGTGCACCTATTGCAAGAAACCGGGCCACAGTTATGAAACTTGCT

TCAAACGGGAACGTTCGTTGACTTCAAATGTAAATTACGTAGGAAGCAATAAGCTGGCTCCTATTTTCATCAAAATAGGA

GGAAAGACATTGCAAGCTGTGTTCGATAGTGGGGCTGAATGTTCAATAATGCGAGAATCAGTTGCTATGGTTCTACCAGG

TCAAAGGAGCTCAGCAGTTAACTATTTGAAAGGAATTGGTCAATTTCCAGCGCTATCGTTAACCAAGCTTACAACTATAT

GCGTCATAGATACCATAAATATTGAGCTGGAGTTTCATGTGTTACCTGATTACGAAATGTCAACAGATATTTTAGTGGGA

ATGAACCTGATTCATGATACCAATCTGAATGTGATAATTACACCCCACGGAACGAAGCTAGTTCACAACACAATCATCAA

TCTGGTATGTACAAATTCTCCTATTTTTAATCATTTGGATTGTGATCTAACCAGCGAAGATGAAATCTCACAATTACGTG

GACTTCTGAACAAATACGAGCATTTATTTATCCGTGGATATCCTAAAACTCGGGTGAACACAGGAGAACTGGAAATCAGA

CTGAAAAACCCAGATAAATTTGTCGAAAGAAGACCATATCGCCTCAGTCCATTGGAAAGGGAGAAAGTTCGTACCATAAT

TCAAGAATTATTAGAACACAATATCATACGTGAAAGCAAATCACCTTATTCAAGCCCTATTATCTTGGTCAAAAAGAAGA

ATGGCGATGATCGTCTCTGCGTTGACTTTCGAGAACTTAACTCGAATACTCTACGTGATCACTATCCACTTCCATTGATT

TCCGACCAGATTGACCAGCTGGCAAATGGCCATTTTTACACAAGTCTGGATATGGCAGCTGGTTTTCATCAGATTCCTAT

CGCTGAAACCTCAATAGAGAAGACCGCCTTCGTTACACCAGATGGGCTATATGAATACCTTACCATGCCGTTTGGTTTGA

GTAATGCTTGTTCGGTTTACCAGCGATGCATCAACAGAGCCCTGACGTCATTGCTGGGGACAGCTGCGCAGGTCTACGTG

GATGATGTGCTGTCCAAATGCACAGATTTCCCAGAAGGAATATCACACCTGGAGAGGATTCTGATAGCGCTGCAAGAGGC

TGGCTTTTCTATAAATGCTGACAAATGCAGTTTTTTCAAACGATCCATAGAATACCTCGGAAACATAGTTTCTGACGGAC

AAGTACGACCAAGCCCACGAAAAGTGGAAGCTCTCGTCAAAGCTCCAGTACCTAAAACTGTAAAACAAGTGAGGCAGTTC

AATGGACTAGCGGGATACTTCAGGAAATATATCCCTGACTTTTCACGTATAATGTTACCATTGTATGAACTGACGAAACA

AGGTGCAAAATGGACATGGACAGACAATCACGAAAAAGCGCGTAACACAATCATCGAATACTTAACGTCTACCCCAGTGC

TGACATTATTCCAAGAAGATGCTCCCATACAACTGTATACGGATGCTAGCAGCCTTGGGTATGGTGCAGTACTGGTACAG

GTGATCAACGGACGTCAACATGCAGTTGCATTTATGAGCATGAGGACCACCGAAGCAGAAAGTCGTTACCACTCCTATGA

GTTGGAAACATTAGCAGTGGTGCGAGCTATAAAACATTTTCGCCAATATTTGTATGGACGAAAATTTACGGTGATAACGG

ACTGCAATGCATTGAAGGCGTCCAAACACAAGAAGGACCTTTTGCCGAGGATTCATCGTTGGTGGGCTTTCTTGCAAAAC

TACGACTTTGAAATTGAGTACCGCAAAGGTGAACGTCTCCAACACGCTGACTTTTTCAGCAGAAACCCGACTGAATTTGC

AGTCAATGTTATGACAAGAGACATGAATTGGCTCAAGATTGAACAGCGGCGTGATGATCAACTGCGACCTATAATGGACA

GTCTTAGCGGTGGCAATTCAGTGGAGGGCTACGTCTTGGAAGATAACGTCTTAAAGAAACAAGTTAACGATCCAGTTTTT

GGTCAACAGCTTAGCACAGTCGTCCCCAAGGCATTCCAATGGAGTATTATTAACTCTTTTCACACCGTACTGAAACATCC

TGGTTGGGAAAAGACATTACAGAAGATCAGAGAAACTTATTGGTTCGATAAAATGAGTTCTACTATTCGAAGATTTGTGG

ACAACTGTGTGATATGCCGAACATCAAA

>Spodo_Contig_20_Harbinger *°

GGGCGCGTTCCCACCGTGTCGTGACGACAGATAATCGTGTAGTTTTAAGTGTCGTGGCTTATATGTTTAAATGGAGGTGT

TCACATTTAGAACGACACAATTTACTGTCGTCTACGACAGTTTTTGCAGTGACGACACTTTGTCGTGACAGTAGGAACGG

CAAGCACGACAGTCGTAAAACGATAAAAAATCGTGGACGGCAACTGTGTTTTCCCTTCCCTCTCGCACCCGCGCGCCCCT

CGCGTCTGTACTCGGTTTGCTTTGTGCGTGTTAGAACTTGTCAGACTCCTGCGCATGGTGGACGTATAACAATGGCTAAT

AATATAACACCGGAAGTTTTAATTCCACTTGTGGAACAAAGGGCTGTCCTTTGGGACAAAACTTTAGACGTTTATAAAGA

CAAAGGTTTGAAATTAGCCGCGTGGAGAGAAATTTGTTGCGTTTTTGAACCTAATTTTGACAAGTTGGAGGAAAAGGAAA

GAAAAGATTTCGGTAAGTTTCTGAAACTTTTTATTTATTATGATACGTGTGCTGTAATGTTTTGCCAAGGTACGGAGCCT

ATTTCAGATACAAAATAGTCGGAAAATTGTGAGCGCACTGCGTTTCCACCATTTCCGTTTGAACGCAGCTGTGTTGCAGG

CATGTTTACCTGGGGCATAACGGTTGGGGTATTCTCCAAGTATATAGCGTCTTTTTTTATTAAAAAATTATGTAATACCG

TACAAGCCTTTACAACTTCAATAGCTGTTTCTGCACATACATCCAATGGTCTATGCAAGATCCTCCATTTATTGGATAAG

ATACCAAAAGTGCATTCAACATATCTTCTCGCGCGGCTTAGACGGTAATTAAATATCTTTTTCTTATTGTCTAAATGAGT

ACCGCCATAAGGTCGCAATAAATTTTGTGACAATGTAAAGGCTTCGTCTCCAACAAATACATATGGTATTTCTTCTTGCA

TATTTTCGTGCAATGGCGTGGGTCTTGGTATATTTAAAGTACCATCATTTAATTTCTTCCAAAAATTGGATTGTTTTAGG

ATAGATGAATCACACTCTTTTCCGTATGAACCAATACTTACGTAAATGAAGCGGTATTCTGAATCAACAATTGCAAATAA

AATAAAAGAAAAATAATCCTTATAGTTAAAAAACATAGAACCACTATTTATTGGCTTTGTAATCCTTATGTGCTTGCCGT

CCAGCGCACCGAGGCAATGAGGAAAATTTGCTTTCGTTTTAAAAGCTTCAGCGACCGTTTTCCATTCTTCTTCGGTATTG

GGCAACTTTAAATATTCTGCACTTAATACGCTCCAAATAACAGAACACGTTTCTTTGATTATTTTGCTGATGGTTGACAT

TCCCACTCTAAAATTATAATGCAGTTGTTTAAACGAACATCCCGACGCCAAATATCTGTAAATAAATACAACAGCTATTA

TAATACGCTTATATTCGAGGACCAATGCCCTAACATTNNNNNNNNNNNNNNNNNNNNNNNNNNNNNNNNNNNATAATTAT

GTATAAACATATAATCAGTCTAATAGGTAGTCCCTTACTTGTTCACGGAAACATCCGGGGATAATGGAAAAACTCGGGGG

AACCCGGGGATAATAAAAAAATGGAAAAACTCGATCTTATTATAGTTTTTTAAACTCTGAAAAACGTGCAGATTTTTGCG

TGAACAATAATTTGACTACGTATTAGACTGATTATATGTTAATACATATAATGTTAAATCTATACACAAACAAATTTTAA

TTTTAAGTCTATTTTACTTTTACAGCTACACACATATCTACAAAGTGGACACATATTCGAGATGCATTTATGAGATCCCT

GAAAAATGAAAAAGAAAAAAAGCGATCTGGTGCAGGCGCAAAAACAACTAGGCCGTATGTTTATAAGAATCAGCTGTCTT

TTTTATTAAAAGTTACTGAGCCCCGTATGACCTCTGATTCATCTAATGAGCCTAATAATTCTATCTCTGATACATTAAGT

GATGCAGCACCTTCTACTCAAAAGAAAACTAAGACTGACACATTAGATGATAAAATGTCACAATTTCTTGATTATAAATT

AAATAATCAAGAACACCCACATGTGTCCTTTATTAAAGGAATTTTACCATCTTTGGCATCATTCGACAATGATGACAATT

TTGAATTCCAAACGGGTATTATGCAACTGATCCAAAAAATTAAAAAGAAACGCCTTCGTAATAATTTATATGATACTTAT

GCAAACTATGGTGACTATCAAAACCATTTATCACAACCGACAGGATATTTTACACAAGATCCAGATGCATCAACTTCGAC

TAGCAATCAAGTTATGGAACCGGCTGCATCACCAAATTCAGTCTATACTAATTTGTCTGATAACACACAAGATTCTTTTA

ATTTTGACAATATATAATTTAAAGATGTCTTTACTTATAAAACGTGTAACTCTAAAATAAATATCTAAATAAATAAATGT

ATACATTAATAAAGAATTTTCATTTTATTTACTTACCTTAAAGTTATCACTAGTCTTTCTTCGGCACTTATACAATTTCT

GAAATATGTGTTTTCTCTTCTAAGATTGTCTGCAAGTCGTGCCAGTAGGCTATCAAAGGTGGAGACAGTCATTCTTAAAT

ACTCATAAAACTTTCCATCATCACTTTTTAATTCTTCTATATAACTCTGACATAATCCTAATGGAATTCTTTTCATTAAC

ATTGGATGTACCCAGTACTTTCTTTTCCTTGCTTGCTTCATTTTCCTTCTTTTCAAAAAGTAATACATTATTAAAATTTC

ATCTTCGTCCATTGAACTCAGGAGAGTAAACGCACTAGTGATCACCAAGCGAACGCAGAGGCGACTAGATTTGTAGCTTG

CTGATGATGTGTAAACGACAAAGCGACTGCCGGGAAACGATAAAATGTCGTCGCGTTCCAATGGGAACAGCTAGATACGA

CAGTCGCTAAATGATAAAAAATGTTGACGACAAACAATCGTTATAATGGGAACAGGTGGAGACGACAGTCGTCGAACGAT

AAATTAGTCGTCACGACACGGTGGGAACGCGCCC

>Spodo_Contig_11_piggyBac *°

GTGATTTAACACGTTCACGGACGCTACCACTTGAGTCGGTGTAACAGAATGACACAGTAAATGTGTATACAAAATTATAC

GACGTATCAGTACGTTGCGTCCCATGAAAAAATATTGTTTTAAATACATCGTGACAGAACCGTTTTATCACGTCATCGTA

TAAATACGTTGTGTCATTAAAAGTAATGTAAANNNNNNNNNNNNNNNNNNNNNNNNACTAGATGTATAGGGACACAACGT

GTAATAACGTCCAACGTGTAGATACGTTCTGGCACATCATATTATGTCAACGACAAGCAGTACCATCACAACGTACCTAT

GCGTTNACTAGATGTATAGGGACACAACGTGTAATAACGTCCAACGTGTAGATACGTTCTGGCACATCATATTATGTTAA

CGACAAGCAGTACCATCACAACGTACCTATGCGTCCAACGTGAAATAACGTTCTGTCACTTTTAGTGTTAAGATATCCAT

ATGATTGGGTCACAACGTATTATGCCGACGACGTTTCTATACGTGTTGTCGTGTCATWGTGAGATGAAAAATKATGCCTT

TAACWGAACKTCATTATACGTTCAAGGTATCTAAATGTWTTATCATAATGCACGTGAAAATAAATAAATAATAAATTAAA

TTCATWTAWGATCGATATAAAATAAAACTTAAAATATGAAAATCAACCATTTATTTCTTTAATAATAATTTTTCAGTCTT

AAGTAGAAACTATTGTTACTTAATTAATCACAACCATTCACTTGCATAATTATATTTATCACAAAAAAGCAACTTCTGCA

TAAAAATAAAATGTAATCAACAGAACAATCAAAATCTAAAACAACTAATAAAAAAAATCAGTCTGTCGTTTAGGAACTCG

TAACATTATCAGTCTCGTGCCATTCTTCAAAGCACAACGCACAAAGTGCAGGCTTATCTTTGCAGCCCTTACATCTTAAT

CCAGTTTCCTTGCGGCGTTTATTCTTTGCACACATTTTGCATTTAAGATAGTTAGTAGTTTTTTTGGAACCAGGAGGAAG

TGGAATCTTTTCAGGCCAATGTCCACCACTGCCATTTTCGGGTTGTGTTGGGGAAGGTCTTGGGTGTGCTGTCCTATCCA

CTCTTCTATTGTCGTTCTTGGAATGCTTCAATAAAAGTTCAGGTCTTTGAGAAAGGCTAATACCAATTAAAGTTCTGATT

AAATCTTCTCTAAAATGTAAAAATTCATATTTCTTACTGTTATTCATTTTGTATTTTCTATACAAATAAAAACTGTTCCA

TACAGTTAAATCAAGTAAATGAAAAAATACCTTTTTATACCATCTCAAACATTTTCGCGGAGACGAATAATAGGACACCA

TCTGATCAGCGCGGTCAATACCGGACATAAATGCATTGTAACTCACCACTTCTATAGGTTTTCTTTTTAATTGTCCGCGC

TTATTTGCAACGTTAATCATTTTGGGATGATCCTTTGTAGTAATCATGGTTACGGCTCGCTTATCAACCCACTTTGAAAC

ATATACGTTATTTTTCCGTACCCATACATGTTCGCCTTTCTTTATCTTTTTGTTCATAACGTTTTTCGGGTTTTCTTTAC

GGCTTTTTCTGAGGGTGCCCACAGTATGAGTTTTGAGATCAAGCAGTTTTTGAGACAAACCGTATGAATTATAATAATTG

TCCATAAACAATTCGTGGCCTTTTAAAACATAAGGTCTCATAAGCTTCATGACAGTTTTATCAGTTTTTTTTCCTTGTTC

CAAGTCAGTCGAGTCATCTTTGCCGGTATACATTAATATATTTAAAACATAGCCGTTTGCTTCCGTCAATTCGTAGAATT

TGATCCCATATTTTGCCTTTTTGGATTTTATATACTGTCTAAACGATAAACGCCCCCTAAATAATAAAAGTGACTCATCC

AAAGAAAGTTCTTTTTCAGGAGAGTAGCACGCTCGGAAATTTCTTGTAATGGCGTCCATGAACTCAGTTATTTTATTTTT

GCCCTTGGCACCTAAAGGTCCTGTGCACAGGCACCTTAACAGTTGTTCATAGCGTCGTCCTGACATAGTTGAGACAAAAA

CAGGGTGATAATATAAACAGTCCGACAAAGTAAAAAGTTTTCGCTTTTGGGGTATATTGACTTGGGCACCAAGCAAGCAA

AGTCCTAAAAACTTCAACATTTCATTCTTGTCTGTCAATTTAAATGTGTAAGACCGACTGTGCCTAGTTTTCGGTCGATT

AGTTTGACACATACTTGTACCGTATGCATTAGTGCATTCGACGAGATAATCTAGTATAAGAGGTGGAAACAGGCGGTTAA

ATACCTCAAACACGGTTGTGTTATTATCAATGGTGAACTTTACCCCCACAGCACCAGCGTCAAAATTGAAATCCGGTATG

GGTGCTGTAGTTTCTGACCAATCTTCTGTAGTTGAAGTTCTAGGATTAGGAGTATCCAACGCATTGGAGGGTTGGCCGGA

ACCTATTTGCTCCTCTTGAAGTATCTCGTCTTGGGGTTCATCGAATAATGGCGGTAGTATATCAAGGGACCTAAATTCAC

CTGTATTATCTAAGTTTTCAGCTAAGACATTAGAAATAAGTGATAAAGTCGACAAACCCACGGTCTCATTTTGAGAGATG

TTGTGTGAAGGTAAGATATCCTGAGTCGTCCCACGAGGTGATGTTTGAACCAGATTTTCAATAATTTCAGGTTCCAAAGG

TATATGTCCTTCTTCAATGACCTCATTGGGACCTTGATTCACAGTAGGTACGGTTGTAATTGGAGATTTAACCAATTTTA

AGCGTTTGGCTAGAGGGAAAATTTCTTCCGAAGAATTCGACTCATAGCCTGCAACCTCCTCTTGAAAACGTGATGTGGTA

GTAGGGGTTCGAGGTGGTGANNNNNNNNNNNNNNNNNNAGAAGTAACATTGGATTCGGGCTTTCGAGTCGAAATACCGAC

CAAATTTGATTGACCCTTAGTAGTTTTTGTCGATTTCTGTCGGTAATTTTTAGAATAAGAACTTTCACTACCTGATGTAC

TTCCTTGTGGATCAAAATCTTTATCCGAACCATATTCTCCATCATCATCTTGGTAAGGATCCGGCTCGTTGGAGGTGTCG

GAATCATCGAAAATGCTCAGCGGTCTTATTTTGCGCTTATCCATCTAAAACACATAAAATAACCCATTAAACATCAATTT

CACACAGTAAAATTATTAATTCACAGTAAAATAATATAGTTTCTAGCTAATGAATAATAAAATAATAACATAAACTAAAT

ACTTACGATGTTTTGTCGCGGCAAATATTACGATACGCCACGAAATGTCACTTGAACGCGAGCGCACTCCCACAGTGACG

CAGCCGCAATGAGTATAGGAGCGCGGTAGGAGCGCGGGACGTATAAANNNNNNNNNCACGTTGTGCGTATGTGAGTACCT

TAACCGCACACTATCAACGGCACTTATTTGAAATAGTTTAAAAATTAMCCTACGTGRCAGTACAGTGTTGGGTATTTTAC

ATGTTGTGACACAAGATACATTCAATAGGATTTTAACTTTAACTTTCATCGGAAATGAACGTTGYGCCGTATAATTTAAA

TGTTATTTGAACGTCATCGTACGTTGTGCCATATCAAAATCTCGAACGATGACGTA

>Spodo_Contig_15_Harbinger *°

AAGGGGCAACGCAAATAGAAGACGGCGAGACGAAGGTACGGTACAGACACGGCGCAGATACGGTGCAGGCGCGATATCTG

CGCAAACAGACGCGGGGACGATACGGCACAGAGTATTATTATACAAGGTGCTATTTAGTGGAAATAACATTTAATACACC

TCCTTTCGGTTTTTTTTCGAATACTAATTCCCTCCTTTTGTATCATTATAATTCCTATGTACAGTCGAGAGCACATCAAG

TGTAACATTGCGCTACGAAAGCCGCCACTATTACAAGTGCAATAAAGGCGTGCTCAAGGTCAAACTGTATAGCTTGATGT

GCTGTCGACTGTACTTATGTTTGCAATATGTAACCGCTATGTTTGCTATTTAACTACTTTAAAGTACGAATAACATCGAA

ATACATAAATCAAATCATAATAAGTTAAATTACATCGGATTTTCATTAAAATCATGGATTAAGGCTGATTTACATTGATA

CAGTAGCTGGCGCCAGCTACTGTATCAATGTAAATCAGCCTTTAAATATCCATATGGACGAAAATTTGAACAGTTTAAAA

TATATCGCTACGTATCTTTGTTCATACTGTATATAATGCGAGACGCCGCTTGCGCCTACATCTGCTGGCGACGTGGCGGG

GGGCGAGGAGCGGCGTGCCTGCGCCGTCCGCTTGCCGCCTTCAGTCACGAACAAAAGATAACGCAAGACGCACGTCGCTT

CGTATACAGGATGCGTGACGATATTGAGTTTAATATTGAGTTTTGCGAGTTAATTGAGCAATACCCGAATATCTATGACT

ACACTAAGACAGGGTACAGTAACAGAAATGTGCAAGACAAAATATGGCAAGAAATAGCAATAAAAGTCGGTGCAACAGGT

ATGTATTTTATTTAAATTTTAAACATTATGTCTAGTCTGAAACGGAATCGGAGATCGTTGTGTTAGATATGTTGTAAGAT

ATTCCCTGATATGGTAGGGATTTTCTCCCATTAATCGCATGTTAATGTTTGCTCCTATCAAATTATTTCTAAACCGTGGA

TTTGATTTTTTACCATCACATGTGCGAATTACGTTATGCAACACACACATTGCTTTTATAATATTATCAACATATTCTGG

TTTGCAGCAAATAGGCGTGTAAAGTCCACCAAACTTTGACGCTAACATACCATAGGCACATTCTACTGACTTACGTGCCC

TTGACAACCTGTTATTATAAGCTCTTCTCTCATTAGTCAATTGATTTCGACCATATGGTCTCATAATATTTCTTTTTAGT

GGAAAAGCTTCATCTGCCACAAAATAAAAGGGCACATTCTGTGTTTCTCCAGGCAAAGGTGTAGGGTTCGGTAAATCGAG

ATCGCCTAGATCCATAGCTTGTCCAAAAGAGCTAACTTGTAATACTCGCCCATCACTATTTCGGCCGTATTCACCAACAT

CTATAGTTAAAAAGAAACCATCTGCATCTACAACTGCTAACAATACAATAGAGAAATATCCCTTATAATTTATGAAAGTC

GAACCCGAGTTGATGGGCGCTGTAATTCTAATATGCTTTCCATCTAAACTCCCAACACAATTCGGTAAATTCCAGAGAGT

GTAAAACCGTTCAGCAACTTCTACCCATTTTTGTCGTGTAGGTACAGGCATATATTCCGGCTGTAGGCATTCCCACAAAA

CGCTAGAGGTTTCATGCACAATTTGCCGGACGGTACTGCCTCCTCTCAATACATATCCAGAAAGTGATGTAAACGAACAG

CCATCTGAGTAGTATCTGTAACAAAAATAAAATAGCAATAAGTATGTAACATTTGTGTGTTTAGATTGAGGTGCCACTTT

TCGCATCGTAAAGTGGCTAATGGAGTTATTTAGTAGTAGTTTAGAAAATTTTGTAGAACGGATATTGGATTTTGTATTAG

AATACTTCAAAAGTTAAATGAATATAAAGGTGGCACCTCAATCTAACTGTTTGCATTGAGGTGCCACTCAGAACTGGGAA

AATTATGGATGAAATTTTATCAAAATTTTGTATTATTTTGTAGTACTCCAGAATTCTGGAGTACTACGAAATTTTGATGA

AAACTACTAAATAACTCCATTAGCCACTTTACGATGCGAAAAGTGGCACCTCAATCTAAACACACATTGTAACTAATCTC

CTTAAATATTTTTCAGCCCAAGAATGTAAAGAACGGTGGAAGAATATTCGATGTAGTTACAGCAAAAACAAGAAAAGGCT

AGATTTGCCATCTGGATCAGGTGCAAAGACGATTAAAGAGTACTACCTTTCACCACATCTACATTTTTTAGACAATTACT

TGAAGTCCCGACCAACCAAAAGCAATCTAAGCAAATATGATTGCACAGACGGTAATGATAGTGAAGAAGTCGATGAGACA

CTGTTTACTGGTCAACTAGCTTCTGAAAACACAAATTCGGATTCCATCTCCAATCAATCCACGCCAACACCGCCACTGCT

ACCACTATTAAATAAATCTAGGGCAATCTCGATAAAAGATGTTAATAAATCAGCACATGAATATTTCAGTCAAAGAGCAT

TACAAATGAAGACCATACATAGAGCTGAGCCTGAAACAGACAGCGATCTAGCTTTTTTTCAAAGCATGCTTCCAGATATG

AAATCAATGACACCAGCCCAAAAACGAAGATTTAAAATGGGCGTTCTCAATTTATCAGAGCAAATTTTAAATGACCCAGT

ACCTAGTTATCCTAGGACACAGCCGTATTATCCACGAACACCTTCTCGAGAGACAGATTATGATCCTAATGAAGGTGCAA

CCCAGCAGACTCTTACATCTAGAGCTAATACAAGTCCAGCACCTTCGTTTACCTCGACTCAGCAGACAGCTAAACATACT

GCAGCAGCTAACAAAAATCCGGAAACTCAGCTGACTTTGATATCCGGAGAGTATATAAATCCAGCACCACCGGCGGCAGT

GGCTGTAATTGAACAAATAGCTGGGCCTTCAAGACACCGAACTTATGATGAGAAAGCTTATTATCAACTCAGCATTTCAG

AATATCAGCAAACGCGGACACCGGGAGCTTATGAAAGAGCACAATCCTTAGCACCTCCGGCAACTCCAACTACATTATGT

TTGCCAATATTTTCACCAATAACTCCAGCTACACCTCACCCGAATCCAGAGACATCTATCCAATCTCATGAAGACGAAAT

AAGTGATTTTAATGAGAACTATCCAATGCCTTGAACTTTATTTATAAATGGCTCTTAGTGCGACAGCTAGCTTGAGTAGT

CATGTGCTGTCCCTGTCCGCATGCGCTGATTCTGTATTGTGAGATAAGGACAGCACATGATGACAGCTTGACAGATACTC

AAATTAACTGTAACACTGAGAGCAATTTATAAATACTAATTGTAGCAAAGATGCTAAACCAAAAACATTTTCAATGAATA

TTATCTTTTGATTTGTCAATAAAAGTTTGATCTGTAAAAAATTGTTTCACTTACCTTAAAGTTATCAGTAGTCTTTCTTC

AGGGCTGATTGGTTTTCTCCAATTTGTAACTTGCTTCCGAAGTCCTTCTCTTACTTTTGACAATACGATATGGTAACATT

CCAGTGACATTTTATAAACTGTGCGAAAACGATCAGGATATTCATATAATGTTTCCATTATTTTATAGTACCTGTGCTCT

TCTGCATTTCTCCACACTGGATGTATCCAATACCTTCTTGGTCTTTGCTGTAAATTAATAATCTGTAGCAAAGCCAGTGC

ATCTTCATCTTCGTCTGAAGAGGAATACATGTCTTATCCCGTCAATATCACGCTCTATAATGACTTAACTTGCCGTATCG

TCCCCGCGTCTGTTTGCGCAGATATCGCGCCTGCACCGTATCTGCGCCGTGTCTGTACCGT

>Spodo_Contig_9_Harbinger *

TGGGCGCTCTACATTCACGCGCGATTCGCGGGCGCGAATCGCGGCGCGAATTGCGGCGCAAATTGCGCGTGAACATTTCT

TGGCTCCACATTTGCAGTGTTCACAGTTGTGTTCACAGTATAGTTCGCGCGCTCGACGAATATGGACGAAGTTGTTGCTT

CGACAGGCCTACTGCTGTGTGCATTTGCATATTATGATTATGTCTATATTAAAAGTAAAAAAACAAATAAAAAAAAAGTG

GAGGAAACGAAGATGGTGGATGACGAGTATTCATCGTAATAGAACAAGGTTTGTAATATTTTGGTCAGTATTGGGCTAAT

GTTTCGTAAAAACAGTGGTACATAAAATTTATTGTATTTAATACCTTTCTTTGTTACAGCCTAACTATGGAGCAGCAGTT

AAATGAATTAGTTGCTGAATCTTCTCGTGAATTTAAAAAGTTTACTCGTATGTCTAAAGCAGATTTTGAATACCTACTAA

ACAAGGTTTCACCACTGATTGCGAAACAAGATACCCAATTACGGAAAGCTGTTCCAGCAAAGATACGCCTTGCCATAACA

CTGAGATATTTAGCCTCTGGAGATGACTTCGAAAGCCTTCATTTTTTATTTAAAGTTTCCCCGCAACTGATTTCACAAAT

TATACCTGAAGTTTGTCGAGCTTTGAATAAAGTGTTAAAGGATAAAATAAAAGTAAGTACATAAAAAATCACTTATATTA

ATTATTATAATCAGTACGAAATTAATCTAATAATATTATTGAATATACAAATATCGTGAGCTTCAATTAACTTGACATAA

AAGCTTTTTGTAATATATCAATATTATCTTCCTCACCGATCATATCAATATTTGCGTCTTCAATGGTGTCTAGATCTGGA

ATGTGAACAACTGGTGGATGGATCACTTCATTCGATTGTATAATAATACTTGAGCCTATGTTTCTCCGATTATTGGTAAT

ACTATTTTGTATGGTCATCGGAGAATAATGAATGATTGGCTCGGAATGTGTTGATGATGAAGTGCCAGGTCTTGGTTGGC

ATTGAATGATTGGCTCGGAATGTGTTGATGATGAAGTGCCAGGTCTTGGTTGGTATTGAATAACATATTGTTGTTGTAAC

GGTGGTTGTATTGGTGATAGTGGTGGTATTGGCGATGAGGATGCTGATGATGCCGTATAATGAGAACTTAAAATCATGCC

ATCTATGTCATGCATCAATCTCAGTCTTTTCTCTTCTGACAATCTTCTCAACTTTTTGGCCAAAAGTCTCCCATACAAAT

CGCAGTCGTCTTCTTGAATTGTATTGATGGATTGATTGTCATGCCTTTTTATAGTAGATTTAAACGTGACCAGAGCTTCT

TTCATCATTTTTCCTGCCTCTGTGAGTTCGCTAGGATTTTTACGACGACGAACTGTTGGTGTAGCTGTAGTATTAGTATT

AACATCCTGACTTTGTTGTGAATCATTCTGACTTTGATTTTCCTGCTCTTCTTCAGGATTCTCTTGTTCTGTATCATCCA

TATTTTCTGGCTGTGTTACCTTAAATGAAAATAAATTTATTTGATTATTTTTGTTGTTACAGTTACCGTCATGTCCAGAG

GAATGGCTACAAATAGAAAGTGGATTTTCATCAAAATTTCCTCATGCTGTCGGATCCATAGATGGCAAGCATATTGTTTT

GGAAAGTCCAATGAACTCTGGATCAGAATATTATAATTATAAAAAAACATATAGTATAGTATTATTGGCGTTAGTTGACA

GCCAATACAATTTCATTTTTGCTGACATCGGGTGTCCAGGTAGCATAAGCGATGGTGGTGTTTTTATGAATACTCATTTA

TGGAAAAAAATGTGCAGAAATGATTTAAATTTACCAACACCACGGCCTCTACCAGGTTCAGATATCGAAATACCCTACGT

TTTTTTGGGAGATGGAGCTTTTGCCCTTTCTGAGTATATTATGAAACCATATCCAGGAAATTACGACATGGGATCTCCAA

AACGTAAATTTAACCAAACATTATCTAGAGCACGAGTCGTGGTAGAAAATACTTTTGGCATATTGGTGACAAAATTCCGT

GTGTTTAAGAAACCAATTCAATTACAGCCAGAAAAAGCAGCCATCGTAACAATGACATGTATATTACTACATAATTTCTT

AAGGCGAAGCAGTACATCCTTATATGTGTATACACCACACGGAACTATCGACATTTATGACAACAGCGATATGCTTGTGC

AACCTGGCTCGTGGAGGCAAGAAGTGGAGATGACATGTGCTGTTCGTGATTTGCGACATGTACCACGCAGACCACCTCTT

AACGCCACGGAAATTAGAAACCAATTCACCAATTATTTCAGCAGATAATTATCATCACTCATGTACTATCTGAATAAAGT

ATAATATACTTACCGTATTGATTGTTGTTTTATTTATTGTTGTATCACCAATCATACTGTCGATAATATTATATCCAAAC

CAAATTGGCTTATACACGTCATCAGCACCTGCTCCCGATTTTTCCGATTTTTTTATTTTACTTTTGTAATTTCTGTAGCT

GGAAAACAAATTAGTTTTTTTTGTTTTCAGTTCATCCACGGGTATTTCCATAATAGTGCTAATACGCTTCCATGCGTCAT

GATTTTTTAGTTTGTTTTTGTAGTCTTTCAAACTTTTATTCCATAAAACTGGTTCCATTTGTATCTGTTCCAGAAAAGAT

TTTTCTAATTCGTTTGTCCAATTAAAACTTTTATCAGACATTTTGCACCACTGGATTTTTTTTGAATAAACAAGCACAAC

CGTTCTCGTCCTCGAGTAGAGCGCGAATGTGGAGTCGTGTTGTGTTCACGCATCTTTCGCGCGTTCGCAGGCTCTTTGAC

GCGGGCCAGAAACCGACAAAAAGTGGTTAACAAGGCGCGAAAGTGGCGTGAACCCGCGAAAAATATGCGAATCGGCTCCA

CATTCACGCCTTCGCGCGCAATTTGCGCGTTTGCGCGCGAATGTGGAGCGCCCTNNNNNNNNNNNNNNNNNN

>Spodo_Contig_19_undetermined *°

GGGTCGATTTCAAATTTGGGGCCAAAAAAGTGTGAATTGCATGATATTTCAAAAATATTAAAAAAATATATGTTTATATA

TCTAGGTGCATTCATTAGTTAAAAAAACACATGCTAATCAAACAAACCTTTAATTTCTTCCAATAACAGGGAAAATACAC

ATTTTTGAGTGACACGAATGAGCAATATAATTTCTCTAATGATATTTTGTGATTTTCTAATGAGATCTCTGGTAACACTA

ATGAAAATTAATAAATAATCAACAAAACATAAAATATTTGTCATTAACATTATTAATAGTAGGTAGGGTAGTAACATTAT

TGCAGTGAAGGTGGATGTGCCGGTCACCGTACTACAGATATTAATTATTTTAAACTTAATTCCAAAACAATAACTTATTC

AACTAAGCTAATCAATGGTATACATTTCTGATTTCAGTCTTCAAGATTGGTTAACGCTACTCCTTTTTCATTGCGTTTAC

AATAACAATATCCATATTATTAATAGCTTCTTCATTAATTAATCTGATAAGGGATCGTCATCATCATCTTCACATTTTTT

TAAAAAATCAAGGAATTCTTTTTCGTGATCTGTCGCTTTTGTATAATTATTTATAAAACATTTATTGGCCCAGGAGTGTC

GAATACTGCGGAGACTTGAAGTTGATGGTGTGCTGTCATGTAACATTGGATCATCGGTATTAGCATAGCGAATGGTAACA

TCACTAAGTACCTTAATTCTTTTGTTTTCCGGGATGACCTTTACATTTTCCATTGTAGATCCAATTGGAGTATTATATAT

CTTAAAAAATCTCATATGTTTGCCATGTTTACAAGCGACACTTATACATTCGAAACAGCTCTGTTTTCTAAATTCTAGAT

AAGCAGAATCAGCTGCCCAAATGACCTGTAAGGAAAATATATGTATTATTATTACATTGTATATGGTAACATCAGTATAT

TTTTTATTTGTTGTATAAAATATACCTGATGAACTGTCATAGTCCCCTTGAAAGTGGCAATATCTTTAGGCAACATTAAT

TCCCTTAATTTAATTTCGTGCTCTTGAATAATACCCATTTCTACGTTTTTTATATTTCTTCGAATAATGTTAACAAAGGC

ATCAAAGTCTCCGACATCCTGATGACACTTTAATTCATAATCGGCAGTCCTTTTGATTACTGCTCCAATGCCATCAGGAG

CACCCTTGCCGTGCCCAGCTTCTTGATAATTCCACGTGACGAGATTTAGATCTGAGAACTCATTCCTTAATTGAGTTAAT

ATATAAAATATATATTTATTTCTGTACTGGCTTGTGGGACTATCTGTTAAAATGTGAATGCGATTAATTTGTGATGATCT

GTTAAAAACAAATTTAATTAAATAATTTAGATGAGCCCACACTGCCGCTGCATCATGACGGGTACATTCACTGGCTGTGC

AAACAGAGTAGGATTGAGTAGCATTGGAATCATTTTTTAAATAAACCACTGCTGTATGAAGTGAGACTTGACCACGGCTG

CCACCAAAATGCATGCTTTGTACTTCCTCCGAATATTTATAAGAATAATTTTCCGAAAAATCCATATGAACCAAGGCTTC

GGTAGTAGTTAGAGATTCTTTTAAACATTTAATAGTAGCATACTGGTTGACAATGTTACTCGTGTGGGACAAAAATTTTG

GCAGCATATTTTCCAGTTCAATTATCAGGTTACGAGGATCAGATCTCTTTGTTTTCTTCTTGATTATTTTTATTTCTTTC

GTGTCAACTTTTTTGGTGTCTCTTTCCCATACCATGTACTGTATTTCTTTATCGTTTCGGAATTCTTTATAAGGTACTGT

TTTATTTTTACATGAAGCGCATTTGCGATTAAGACAATCTAGCCTGTATTTATCACAACAGATTTCCTTTATCAAATTAT

GACTATCTTTTACATGAAGAATATTATTCTTATTAAGAGCCGCTATTAATAAGTTAAAATTTGTGTGCAACAAGCAGGCG

CACGTATCACGGTCGTCAGTCGGAAGAGAGACCCAAAATGGTCTGAGTCTTGTAAAAGTCGTGTAACTAATTTTAATGTT

AGTATTTTCAGCCAAAAATTTCTTGTGTAAGTTTTTTATAGAATCTAAAAGATATCTGACTTGTTTAACGATTCCATGTT

TCTTCTTATATTGTTTCTTGCCTGGAGTAATGCGACTGTTAATGTCGTCTTCGTAAAACTTTTCTACTAATTTTTGAATG

TTGATGTTATTACGTGCATTCTTCTTAAATCGTTCTCGAAGATGAACTTTCCGAAGGTCAGTAGCAACCTTAAATTTTTT

AAACTTCTCTTTATCAATTAACTTAGTGATAACGCGTTTGTCTTTCCTGTTTGCAGTTTTTGCAAATTTCTGTGTTATTT

CAGCACTTAGAAGTGTATCAAATAATTGCTTCTTGTCTTCATTTTTTGTTTTTTCTGCTTCATTACTTATTTCACCTGTT

AGTGTA

>SEUC37551_TC02_MuLE

GACCCGCCGGCCCCCGCGGTCGCGCGTATTTCCCTTTCCGTGTTCAACTGGCGACACCGGTCTCGCGAAATCCACACGCT

TTTGTTTTACTACGGAATCCTACGATCCCGTGTTCAATTCGCGTCAACTGAGCACGCTTTTGCGATCAAGCAACGTTAAA

AGAAGTTTTACAAAACAGTGTGTAATTGAATTGAACACGGTTTTTTAACCTGTCTATTCGAAACAAATCCGTACTGATTT

AGCGCGTTCGGAAATGTTCGGGATAAATCGTGCGGGCCGAAGCAGGCGAGAGCGCCCGAAGTTGGATTCGCGCGCTTCTC

ATTGCAATTTGTTATTTGTTTGTTACTGTGCGTTCAAGGTTATGTTTTGTTTACATAGTGCATAATGGAAGAGTTTAAAA

GGGTTTTCAGTGAAATCACTAAAGATTTTGAAATACTCAACTATGACGGACAGTCTAGTGACAGTTATAAGTGTAAAGTT

CAGTGCTTATTACATTATGACGGAGAATACGACGAAAAATGTGCTAGATTTGTGGAATGTTTTTCAAAACAAACAAATTC

TAACTGGGTTATGAAAAGACGGTTTGCAGAACCCATACGTTATGAATTCAGAAAATTGTTTGTATGCCATCTTTCGGAAA

GAAATAAATTGATAAATAAAACGCAAGGTGTTAACAGAAACTATAATTGCAAGTCATCTCTTAATATAAAATTTTTGAAA

ACTACACCAAGTACTCTGAGAAGTTCAGCCCTGCTGAGAAAAGGATATAATGTTATAGTTGACATCCACTTCCATCATTC

CCACAGAGTTAATGTAGCTCAATCGTTATCCTTGCTCCGATGTTCGAACGATACGAAACAAATATTTATAACTTATTTCG

ATAGAGGTATGACTGCCGCGGCCGCTAAGATGTACCATGAATTAAATATTATAAGTACTTATGAAAGCGATGCCTACAAA

ATCTTAGCTAACGCTCAGCATAACCCTCTAGATCGTCAAGTTGTGTATCTTTACGAACAATGGCGTAAACAAAATTATGG

AAATAGAAATGAACAGACTGTGTTACAAATTTTGAAAAGAAAACAAGACGAGTTAGGGAAATTGGGTATAGACTTGATCA

TACTGGAGAATCCTACTATTTGTGTAATTATTACACCAATAATGCAGCGAGTTTTCACTAAAGGATTAGCCGATGAAATA

GTTTTTATAGATACCAGCGGATCATGTGATCAATCAAACACATGTGTGACATTTATTTTTGCAGCTTCTAATATTGGTGC

GTTGCCTGTTGCAATAATTTTACACGCTAATCAGACAGAAGAACAATACAGTTTAGCGTTCAAAACTCTGAAAGAGTTCC

TCGAAAATAAATTCAACAAATTATTTCAGCCAAGTGTAGCCATGACCGATGACAGCAGAGCTGAAAGAAATGCGTTGAAG

TCAGTTTTACCTAACACCCGGCTTTTGCTTTGCATATTTCACGTTAATCAAGCAATATGGCGTTGGCTTTGGCAAACAGA

GCATAATGTTAATAAAGATGACAAACAAT

>SEUC27063_TC12_undetermined

GTACTTTGTGCACCGTGTTTTGAATAGTTTGCAGTTATATAAGGTACGTTTTGGTGCCCTTATCATATCAGTGAGATTAA

TGTCTACATATAATAAACACTGTATTATTGGAATCTTAACAATCAGTAGACAGGAAGAACTTCTGTAAACATGTCTTTCC

TAAACAAGAACTACAAATACGTCAGCCAAGAGAACTTTGATGGGTTCCTCAAGGCAGTTGGTGTGACTGGAGAAAAACTT

GAACAACTCATGAAATTCACACCTGATCAGAAGCTGACTAAGGATGGAGACACCTATACTTACTACACAGTATCACCTGA

TGGGCCAAAAGAAATCAAATTCAAGTCCGGAGAAGAGATCGACGAACTTCTTGGTTCTTCAAAAACGCCTGTTAAGAGCA

CATACGTAGTTGAAGGCAATACCTTGACACAAACTGTCAAGTCCGACAGAGGTGTTGGTGTATTCAAGAGAGAGTACAAT

GGGGATGATCTGATTGTGACCCTCACCGTTGACAAGTGGGATGGCGTTGCCAAGAGATTCTATAAGGCTGAATAACCAAT

ACTCCTTCATCATCATGACTTCCTTTAGGTGACCCCACGTCGTCGCATCAGAAGTGGCAAGCTTTACAATCAGACGCTAT

TCCTCGGGTGTTTTTATTTTTAAATTATTCTATTTTAGTAACTTATTTATTTCCATTATTGGGTGATTCTTTAAAACCGA

GGGAATTCGACTCATTGGTATGGCTTAATTTTTTTTTGTACAATATAATCTCAGTAAGGATATTAATTTGTCCGGGTAAC

TAAATGTAAAAACATATGTCAAAGGCACTCATTTGCACCTAGTGGCTTACGATTTAGAGCGTGCTGATTAATTGATCAGC

CAGATGAATTATGAAAAAGGCAGAATAATGAGATTTTGCGCCAGATTAATTTGTATCATGCAGATAAATGATAATGTCAC

TTAATTTTTAATAAGTAGGTTTTATTAATTAAACTTTCATAATCACAGTTGCAATTTAAAAATAACAAAAAAATAATTTA

AACATTAAGTAAGTTAAGTATTACCTAAATCACAATTTTGACAGTCGCCTTTCGTTTCGAAGAAAATTCTCACAATCACT

TTAAAATATAATCATGTAATCTTAGTCCTATTAATCTTTGTCATACGTCTGCGCGTCTAACGCGTAGTAATGCTCTTAAA

AAGGTAATAAATGCAAAAAAAATCAGCCTTCATTTTCAATTGAGGAAACTTAAAATAATCTTCATTATAGACATAATCTT

CATTATTAACAAAACTCTTAATAACATACAACAGAAACAGCAAAGATCTTGATTTTAAACTAAACCCCAAACTTAACCAC

TAATAATAAAAACAGTAAAATCTTCGTTGAAAAGTAAACACTTAATAATAATGATAGTCATGAGAATCAAAATCAGTCCT

AATTTAAAAGAGGTTTCACTAACATGAGATCAAAGAATCGAATCTAAGAATATTTCTACCTAGATATCAAAAAATATTTA

AATCTTCGTCATCAGAGTCTAATAGATACGGATTGACATTTTCTAAATCAATACTAAAATGTTGGTTTCCATCCCTTGAA

AAAGTTATCTCCGAAATATTATCGAATGCATCTCCTGTAGATTCAATAAATCGATCTGCTCTATCAATCCCTTCTCCTTT

AGTTTCATTTTTTTTCATCACTGTTGATTCTTTTCCAATGGCACCTTGTGTATTATGTATTCCTAATTTACTCTTACTAT

GTGAGCAGTTCCGTATTTTATCAATCTCTGACAAATTACTCTCTTCTTTATTTGCTGTTTTGTTAGGCGTCCCGCTAATA

TCTTGGTGTTCATTCGTTTTATTACTCATTGTCCAAAAAGATTCACCAATTTCTCTCAGTTGTCTTGTTGAATTAGCTAA

CGGTGTGACTGGCAAGTTATGAGGAACTTCTTCAAAGCGATAAAACCCAAGATGGTTCATATGGTTGCAACAAATGGTTT

TGTATAGGCATTCTGGATCAACGCAGCTCAATTTCCGAACGGCTATCACTGGTGATTCTAAGTTCCATACTATTTGGTGC

ATTTTCATGCACCCTGAAAAAGCTTTAACTTTTTGTGGAAAAAGTAATTCCTTCTCACTAACATTATACTCAGAAACAGT

TCGCAATATAGTTTTTTTTAAGTTATGCTTCATGATATTTAAAAAATCATCCAGATCTTTTATGTCTTGACCTTGAGAAA

CAGCTCGATCTGCTGTTCTTTTAATTATTGCTCCTACTCCATCGGGGGCACCTTTTCCGTGGCCAGACTCACTATAGTTC

CATGTAATGGTTCTGATTGAGCTCAAGTATATATGTAAATAAGAAATCAAATGAAAAACTTTTTTGTTTCGGTACTGGCT

AGATGGAGAATCAGATATAAAATGAACAGTATGAATGCCTGTCTTTGTTTCACCTATAAATTCAAGAATAGGTACTATGT

GGGCCCATACTGCTGATATGTCATGACGAAGGCACTCACTCACCGTACAAAATGATTGTACTTGCTGTATCTTCTCATCG

TCTAGGTAATATATAACCGAAGTGTGTAAAGTTAACTGTTTACGGCTACCACCAAAATGGTATGACTGAATTTCATTATT

ACATTTGACTGCATAATTCTCACTGTAATCAATATGGATAATACACTCACCCTCTTGAAGATGTTCTTTTAAATGTTTGA

AAGCCTTTTGCTGAGCAACTATATTTCCACAATGTATAAGATACTGATCCAGTGTTGATTCCAATTTTTCAATACACTTT

TTAGGCTCTTCTCGTTTTTTCAATTTCTCAGTTGTTCTTATTTTTCTAGCTGTCCCTTGTATTGTGTGTTCTTTAGTTTC

CCACTGCCAGTACGTAATTTCGATTGAATTATCAAATTCCTGATAACATACAGCCTTATTTTTGCATGCTGAACAGGTTC

TATGCAAGCAATTAACATTATACACGTCGCAACATATCAAAGATAGAAAATCTTGAGACGTTTTAGTTGTTAGTATCTTA

CGCTGATACAATCCTTGAATGATTAAAGCCATATTAGTGTGCAATTTGCATAAACAAGTGTCTCGTGATCGAATATCAGG

CAGAAGAACCCAAAAAGGCCTCATCTTACAGAATGCTGAATAGCTAATAGTATAAGAAGAAGTTTTTAGAAACTTTTCAT

GCAGGTTAACAAGAGTGTCGCTAATGTACCGCTTTTGCTTACGAACTTTGTTTCTTGTTATACATTCCTTTTTTCCAGAG

CAAAGCCGACTATTTTCGTCGTCTTCAAAAAATACTTCGATTTCTTTTCGCAGAACTTCAGGAATAGTTTTTAAGCTATG

GATTTGTGTTTGTTTTACCTTTTGTTTCATTCTACTATTTCTTCTATTTCGATCGGTCAGAACTGGGGATATTTCCTTTA

TTTTTGCTGGCATTATTCTTTGTATTTGTTTTTTATAACGTTCATTTTGTAGAAAATCGCCTGTCTGTTCTTCACCTGTC

TCTTCTGCCGTGAGACGTATCAGATACAATA

>SEUC42258_TC01_Pao

AAGAATGGCCACAAAATAAAAAGGTCATTGCCCTCACGGACACGGGCGAAGAACGCGTCAAATGTCATGTGATCAACAAC

ACGACAATGAACGATCACCTCCCCAATATTACTCGATTTTCCAAATGGACACGACTGATAAGAACAACTGCAAGACTACT

TCAGTTCATTGACCTGTGTAAACCAAGTAAACATACTGTCGAATATAAATGCACTAAGAAAAAGGCAACAAAAGATCCAA

CATGGAAAAGAACACACAAAAATAAACTCGTTAAAAAATTTACGCAAAATATGATAAATAATAAAACTGTAAACTACATA

CCTATTCCTGCTTCATATATACAACGCGCCGAGGAACAATGGATAAAGGGATCGCAAGCCGAAAGCTTCTCAAAAGAAAT

TGCCGCCATTAAGGAAAACAAACCGACGCCCGGCGACAGTCCGTTATCAGTACTAAGTACATACCTAGACAACGCAGGCG

TTATACGTTTGCGCGGGAGAATCACAGCGGTACAAGACGTAAATGAAGAAATTAAAAACCCTATTATACTCGAGGGTAAG

CACAGGTATACCAAGCTATACATAGCATACGTACATGAAAGTTTACATCATGGCGGGACCGAGATAACAGTAAATGAGAT

AAGACAGCGTCTTTGGATTACAAAACTACGTCCGTCGGTAAAAAATGTAATTAAGGGCTGTTTATATTGCAAAATAAAAA

AGGCGAAGCCTGCTAACCCGTCTACGGGTAACCTACCGTACCCCCGTTTAGCTCATCATGCACGTCCTTTCACTTTCACT

GGACTAGATTATTTTGGACCCATATCGGTTTCTGTAGGTAGGCATAAGGAAAAAAGATACGGAGCTTTATTTACCTGTTT

AACGTGTCGAGCAATACACATAGAAATAGCTAATTCGCTTAGTGCAGACTCAGCTATCGCAGCCCTTAGGCGATTTATAG

CTAGGAGAGGTTCTCCCAAAGAGATATGGAGCGACAACGCTACCTGTTTCAAAGCGGCAGATAGGGAGCTAGGTGAAAGC

GCCTTAAGCGCTTTAGGTAGTGAGTCCAATGCGCGGTCCATAAGTTGGAAGTACATACCTCCCGCGGCACCGTTTATGGG

AGGAGCGTGGGAAAGAATGGTGAGGGCTGTCAAGGAAGCGCTGTACGTCACACTGAAGGAAGTCCACCCGTCTGACGAGA

CCCTGGCCACGCTACTCGCAGAAGTGGAAAACACGGTGAACTCCAGGCCACTCACGCACGTCAACGTGTCACCGGATGAG

CCAGAAGCCCTCACGCCTAATCATATTTTGATCGGCCCTAACCCACATGTACCCTCACCGGGTAATTTCACTGCAGCGGA

CATTACAGCACGGCACCAATGGCGGCGCGCCCAGGCACTTGCAGACGTCTTTTGGCGAAGATGGGTACGTGAGTACCTGC

CGCTCCTTCAAAACCGGCGGGAGCCCTACGGTAGCGGTACCCCACCCAAAGTTGGAGACGTCGTCGTGATCTGCGACTCT

AACCTTCCACGCAACACCTGGCCTAAGGGAAGGGTGACGCAGGTGTTGCCAGGCGCGGATGGAGAAATACGAGTTGTAGA

CGTCATGACCAGAGGACTCGTACTACGAAGACCAACGAAGCGCATCGTCGTGCTCCCCACGGAGTCGCCTGAAGGCGACG

GCGGGAGAAGTGTGCACGACAGAATCATTACGTAGCCAGAGCGGCGCGCCACCGCGCCACGGAATCATAACACAGACGTA

GCGGCGCCGCACGCCGTTACTTACCTGTCTTTGTTTGGTTTAAACCTGTTTAAAACGACTATAAACCCGTTAGTAGTAGG

GCAACGGAGCCGCGGAGCCACGAGCCCAGACAAGTACAAACACGTTCTCGCGGCTCGGTCGCTCCACGCATCGCCATTGG

CCAAACCACGCCCCCCGCATGTCAGCGTCTATAAAACGCCAGACCATGGCGATGCGCATAGTCAGTCAGTCAAACAGTTT

TGGGTTTAAAAGTGTCACCACCTTGTTACGTATAAAAAACTCTAAAATTTCTTATGTTTTGTGTAAAAGTTTGTAAATTA

ATTAAAAATCCATAATCAAGCTATCGAGTCTCAATTTATAATATTTATACAGTCCATTCGCCACACAATCTGGTCCTTCG

AAGCCGAATAATCACTAGCAAGATGGTGTTAACACGTAGCCAAAGTGGTGACTCAAGAAGAGAACAGACCCAAGAAGAGC

CCTCAGTGACCGCCGGACCGGGTGCCAGCAGCTCGGCACCAGAATCGAGCTCGGTCGGCGTGTCAGGCACAGGCAGCGGC

TCTACGACGACTACCAACACCGACACCACGCGAACACATGGTACGCTCAAGCCTGTCGAAACCAGGTCACACCGTTCCAC

ATCTTCGAAAAAGAAGCGCCTCGCAGAGCTGGAGGCCAGAGAACGGCTGGCTGAAATGAAAGTCAAGCAAGCGCAGGCAG

AGAGAGAGCTGCAGGAAATACAGCTGCAGCGCATTAGAACCGAAGCTGAGTCGAGTGAAGACGAAGACTTGGAAGACGAT

CTTGCCTCCGTGAGGATTGGAACGTGGCTCGCGCGACCGGCGCCGCCACCCGGAGGTGAGACTCGGCCGCCACCACACGA

CGCCAGCGACGGGAAGAACGGCAGAGACGCAGCGACGGACACCGGCGGCGCCGCGACGGGCGCTCGGCCGCTGCGAGACG

GCGACGGCTACGGAGAAAGTGTGTGTGCGAAATCAAAGGGCAATCTCGCGAGTGAAGTGTTGCGCGGTAGTGATAAAGTG

ATAGACGTAGGGACACTGGCCGCGGCCCTAGCGCAGGTCGCGCGCAGCTCAAGAGAGCCAGCGAGGTACGTCCAGCAGCT

ACCGATCTTCGACGGAAAACCCGGTGAGTGGATAGCTTATAAGGCCGCGTATTGTGACTCACACACGTATTTTTCATATG

TAGAAAATGTTGCTAGGATTAGGATGAGCCTCAGAGGCGCCGCAAAGGAAGCTGTAGGTTGTCTATTGTTTAGTCAATCT

AACCCCCAGGTCATTCTAGACGCACTAGAAAGACGTTTCGGTAGACCCGAGGCCTTGATAATGACCGAGTTAGAAAATGT

AAAATCGTTACCTAAATTAAATGATAACCCTAGAGACATTTGTATTTTTGCTAGCCGTATTGCAAACACGGTGGGTACGG

TAGAAGCACTAAATAGAAATCAATATTTACATAGCCCTGAAATGTCGCGTTGTATCGTTGAGAAGTTGACGCCAATATTA

AAAAACAAATTTTATGATTACGCGTCTAGTAAGAAAAGTGAAGATATACCCGTATTAAAGCTATTATCTATGTTCATGAA

TGAAGAAGCAGACAAGTGTGGCGCGTATGCTCAGCCAGAACGCGGGAACGACGAGTCCAGTGATGTACGACAGCGGCGCA

GAGTCGAGCGCACGTTCAACGTAAATAAGGAAACACCGAATGACCAATGCCCGGCATGCAAGCAACACGGCCACAAATTA

CCCGACTGCAGTAAATATAAACAGCTAGACATTGATACTCGATGGGATATAGCTAAAAACTCGAACATGTGTTTTAGATG

CCTACGAAACAGACATAGAAGGTTTACGTGTAAAGCGCCTATTTGTGACGTAAAGGAATGCAACATGAAACATCACAAAC

TGTTGCACAAAGAGCGTAAAGTTGAGGCGTCACACAAGGTCGCGACGCCGCCGGCGGCGTCGCCGACGCCTGCTGTTATC

ACACACGAAACCGTCACGTCGGCGCGGGAAAATGTATCAACGACTACGCCTCGGAATCGCCGCGCGTATTTAAAGATAGT

ACCTGTCACGTTAAGCGGACCACATACAAGTGTAGACACATATGCTTTGTTAGACGATGGGAGTACAGTCACCTTGTTAG

ACGCGTCAGTAGCAGATATCTTAGGTGCAGATGGGCCGGCGAGCCCTATGTGGATACAAGGCATAGGCACGGAATTAAAG

CATGAGCAAAGCCGAACAGTGAGCCTTCACATTCGCGGTAAGCACCATAGTGAGCCACAGAAGATAGAGGCGCGCACCGT

GACCAGGCTCGAAATTATGGCGCAAACGGTACATGACCAGGACATAAATAATTGTAGGCATTTAGACGACATAAGTAGCA

CTCTTGTCTATGATCACGCGAAACCAATGATTTTAATAGGTCAAGATAACTGGCATTTATTAGTAGTCTCAGAAACACGG

ACAGGTGCGCGTGATCAGCCGGTAGCATCGTACACAAAACTCGGCTGGGTACTGCATGGGTGTCAGTACTCCGTAAATAA

AGTCATTGAAACATTTTGTGGTCACATTAAAATTGAAGAAAATCCGGAACCAATAGAAGAAGCTATGAGAAACTTTTTTA

AACTAGAGTCGCTAGGTATAGAACCTAAGTTACCTAGAGACGACCCGCAACAGCGAGCCTTAGACATCCTAGAACAGAAG

AGTCGACGATTACCCAATGGCAGCTTCGAGACCGGACTATTGTGGAAACAAGAGGAACCTAAAATACCAAACAACTACCT

AGGTGCCCTAAAACGCCTAGAAAATCTAGAACGAAAACTAGATAAGAACGAAAACCTTAAGACACAATACTTACAACGCC

TCGAAAATTTATTTAATTCAGGTTATGCTGAAAAGGCGCCCGACACATGTACGCCGAATAAAACCTGGTACCTACCTCAT

TTCCCAGTGGTCAATCCAGCCAAGCCACATAAGCCGCCACGACTGGTGCACGATGCAGCAGCTAAGACAGCGGGTCTGTG

CCTAAACGACCTACTGTTATCGGGACCCGACTTATTAAAATCATTGCCCGGTGTTTTAATGCGCTTCCGGCAAAATAAAA

TAGCTGTGTCAGCCGACATTAAAGAAATGTTTATGAGGATAAAAATAAGAGAAGAAGATCGCGATGCGTTGAGATTTTTA

TGGCGAGGCGATCAACGAAGTGGACCGCCGGGTGAATATCGCATGACGTCTGTTATATTTGGCGCGACGTCATCCCCATG

TACGGCACTATATATAAAGGATCGAAACGCAAGAGACTTTACTGATAAATACCCTGCCGCCGTGTCAGCGATAATAAATA

A

>SEUC40004_TC01_Sola *°

AGAAAATATCGATATTAATAACTTACCAATAGAAATAGTGTCCGAGCCTCCATCTCCGCCTTTGCTGGACCTATTGAGCC

CTCAAAACATTCAGGATGCCATACCTCTTTCTCCATCTGAACAAGTTACCATAATACATTTGCAGCCAAGTGAAGAATTG

CAGCCTCTGCCCTCCCCTGTTGCGATTTCACCGCCATTAGAAGCGACAAAACCACCTGAAAATGACATTGAAGGAACATT

CCTACCTTCAGCTCCATCAACCTTGGAGCAGCTACCGTCTGACAGTGAGATGGAACATATAAATTCTAATAGAAAAAGGA

AATCGAAGGGATTCGCACAACCTAAAAAATGGAATAAAAACACAACAAAACTGAAACGTATGCTAGGTGAAGAATATGTG

GGGTACCGTAGGGACAGGAAAGTTACAGGGCAAGAGAAATTTCAAGTACTCCATGATGTGCAACGTCCAGCACGTTCAAT

TGGTTCACGTTGCACTTCTTTGTTTTGTTCGAAGTCTAAGTTACGTGGCTGTTCTAGCTTGTCTGACATCGAAAGAGAGA

ACATATTTGGCAAGTTCTGGAAAAGCATGACTTGGGAGCAACGGAAACAGTATGTTGTATCTCACGTCTCCGTTTGTGAA

AAAAAGCAAATAAAAGTAAAATCGAATTCTCGTAGAAGTGGGAGTAAACAGTACTTTCTCAGTACATCAGAGGGACGTAC

CCAAGTTTGTCTGAAGACCTTTTTGAACACTCTGGGTTTGAAAGAGTCGACTATTAGGTGCTGGGTCGATAGATCTGAAC

ATGGCATTGCTGAAACAACACCACGACCAAACGACAACAATGATGAACGCGTTATTAAGAAAGAAGACATGACATTCCTC

AAACAGTTCTTCGCTTCGTTACCAAAAATGCCCTCGCACTATTGCAGGGCATCTACATCAAAACAGTATTTAGAGCCGAT

TGTTGAAAACAAGAACTGTCTCTTTAGGTTGTACATCCAAGAGTGCGAAAAAGAGTCTAAAAGTCCACTTAGTCGCTGGA

CTATGTGCAGAGTTTTTGATGAGTTGAATCTAAGCCTGTTCTCGCCTAAAAAAGATCAGTGCGATACTTGCTGTTCGTAC

AAGGTAGGCAACATAGCAGAGGAAGAATATAGATCACATATTAAGAAAAAAGATATGGCAAGAAGCGAGAAAGTGAGAGA

TAAAGAAAGGGCTCGTCAATTGGATGACGTACATGTTTTTACACAAGATTTACAGGCCGTAAAGTTATGTCCACAACTTC

AAGCCAGTGCTTTATACTTTAAAACTAAACTATGTGTGCATAATTTTACGATGTACAATCTTGCCAACAAAGAAGTTACC

TGCTACTGGTTTGACGAGAGTAACGCTGGTTTGACTGCTTCTGTGTTTATATCATGCATCATAGACTGTTTCAAAAAAAC

ACTACAGAAGAAATGCATGCCAATCATTTTATATTCTGATGGTTGTACGGCGCAAAATCGGAATGTTTATCTATCAAATG

CTCTGCTGCAACTATCCATGGAAAAAAACATCATTATCGAACAAAAATTTTTGGAGAAAGGTCACACACAAATGGAGTGT

GATGCGGTTCATAGTTCAATCGAGCAAAAATTAAAAAACCAGGAGATCTTTTTACCAAGTCAATTTGCGACGTTGTCAAA

ACAAGCTCGACCGCAAAAACCTTATTTAGTAGAATACTTAGACTACAGTTTTTTTGATGACTTCTCAGACAAGAATTCGT

TCATGTATGACTCCATACGGCCTGGTCGCTCCGTCAATGATCCTACAGTAACGGATATACGAGCACTGCAGTATAATCCA

AGTGGAGTCATCAAATATAAATTGAACTTTGAGGAAGATTATCAGGATCTGCCACGCAGAATTCGACGCAGAGTAGTAAT

GCCCGAAACTTTACCCCCTAGACCAAAACTCTACCAAAGTCGCATCAAAATATCAGTAACTAAATGGAAACATTTACAGG

AGTTAAAGACTGTTATTCCTTCTGATTGTCACGGGTACTATGATTCCCTACCCTATTGATCTCTTAAAAGTCTGATTATA

GTTAAGAAGTTGATTCGTTTTAAGCAAAACCATTTTTTATAATACAAAATATTTTAAATTTTTTATAAAATAAAGTTTTA

ACACTTTCAAGTTTTAACAATTTTATTTTATTTGCCTTCTCCAAATTATCTCCTTTAACTATGTTACTAAGATGAAACAT

GGCGTAAGTCAGTGGCAACTACTGGCAACACTGCGGAATTCACCAAATTACTTCAATAGAAAAGATTATTACAATTTTCA

CGAGTTCAAATCATCACAAAGTTCATTTGAATTAATAATTAAACACAATACGGCCATAAGCGTAAGTTACATGCATTTTA

AACAGTTTTTTCACTCTCCTGTAAAGTAGCCATTTATGACTTTCGTACTGTTAGAACTTCACTGACGATATGCCTTTAGT

TAATCACCCCAAATTAGTAAAGTTAGGCTGGTATACATTTTCAATTTTACTTTACAGGGTGTCTTTCATAAGGAATTAAC

ATACATGGTAAGCATTGTCGGACGTAGAACTGTCGGTCCCTGCAACTTGATATACTGGTGTGTTAATATCCTCCGCCCAC

GGATACTTCGCCCTTTGTTGTGCAGCCGATGCAAATTGTTGTTCACTCCTAGACACCACGGTTGTTTGATAAGGGTGCAC

TGTGCGCGGAGACAATTGCTGCTTAAACATTATTCTTTCTGAAATT

>SEUC40004_TC03_Sola *

AGAAAATATCGATATTAATAACTTACCAATAGAAATAGTGTCCGAGCCTCCATCTCCGCCTTTGCTGGACCTATTGAGCC

CTCAAAACATTCAGGATGCCATACCTCTTTCTCCATCTGAACAAGTTACCATAATACATTTGCAGCCAAGTGAAGAATTG

CAGCCTCTGCCCTCCCCTGTTGCGATTTCACCGCCATTAGAAGCGACAAAACCACCTGAAAATGACATTGAAGGAACATT

CCTACCTTCAGCTCCATCAACCTTGGAGCAGCTACCGTCTGACAGTGAGATGGAACATATAAATTCTAATAGAAAAAGGA

AATCGAAGGGATTCGCACAACCTAAAAAATGGAATAAAAACACAACAAAACTGAAACGTATGCTAGGTGAAGAATATGTG

GGGTACCGTAGGGACAGGAAAGTTACAGGGCAAGAGAAATTTCAAGTACTCCATGATGTGCAACGTCCAGCACGTTCAAT

TGGTTCACGTTGCACTTCTTTGTTTTGTTCGAAGTCTAAGTTACGTGGCTGTTCTAGCTTGTCTGACATCGAAAGAGAGA

ACATATTTGGCAAGTTCTGGAAAAGCATGACTTGGGAGCAACGGAAACAGTATGTTGTATCTCACGTCTCCGTTTGTGAA

AAAAAGCAAATAAAAGTAAAATCGAATTCTCGTAGAAGTGGGAGTAAACAGTACTTTCTCAGTACATCAGAGGGACGTAC

CCAAGTTTGTCTGAAGACCTTTTTGAACACTCTGGGTTTGAAAGAGTCGACTATTAGGTGCTGGGTCGATAGATCTGAAC

ATGGCATTGCTGAAACAACACCACGACCAAACGACAACAATGATGAACGCGTTATTAAGAAAGAAGACATGACATTCCTC

AAACAGTTCTTCGCTTCGTTACCAAAAATGCCCTCGCACTATTGCAGGGCATCTACATCAAAACAGTATTTAGAGCCGAT

TGTTGAAAACAAGAACTGTCTCTTTAGGTTGTACATCCAAGAGTGCGAAAAAGAGTCTAAAAGTCCACTTAGTCGCTGGA

CTATGTGCAGAGTTTTTGATGAGTTGAATCTAAGCCTGTTCTCGCCTAAAAAAGATCAGTGCGATACTTGCTGTTCGTAC

AAGGTAGGCAACATAGCAGAGGAAGAATATAGATCACATATTAAGAAAAAAGATATGGCAAGAAGCGAGAAAGTGAGAGA

TAAAGAAAGGGCTCGTCAATTGGATGACGTACATGTTTTTACACAAGATTTACAGGCCGTAAAGTTATGTCCACAACTTC

AAGCCAGTGCTTTATACTTTAAAACTAAACTATGTGTGCATAATTTTACGATGTACAATCTTGCCAACAAAGAAGTTACC

TGCTACTGGTTTGACGAGAGTAACGCTGGTTTGACTGCTTCTGTGTTTATATCATGCATCATAGACTGTTTCAAAAAAAC

ACTACAGAAGAAATGCATGCCAATCATTTTATATTCTGATGGTTGTACGGCGCAAAATCGGAATGTTTATCTATCAAATG

CTCTGCTGCAACTATCCATGGAAAAAAACATCATTATCGAACAAAAATTTTTGGAGAAAGGTCACACACAAATGGAGTGT

GATGCGGTTCATAGTTCAATCGAGCAAAAATTAAAAAACCAGGAGATCTTTTTACCAAGTCAATTTGCGACGTTGTCAAA

ACAAGCTCGACCGCAAAAACCTTATTTAGTAGAATACTTAGACTACAGTTTTTTTGATGACTTCTCAGACAAGAATTCGT

TCATGTATGACTCCATACGGCCTGGTCGCTCCGTCAATGATCCTACAGTAACGGATATACGAGCACTGCAGTATAATCCA

AGTGGAGTCATCAAATATAAATTGAACTTTGAGGAAGATTATCAGGATCTGCCACGCAGAATTCGACGCAGAGTAGTAAT

GCCCGAAACTTTACCCCCTAGACCAAAACTCTACCAAAGTCGCATCAAAATATCAGTAACTAAATGGAAACATTTACAGG

AGTTAAAGACTGTTATTCCTTCTGATTGTCACGGGTACTATGATTCCCTACCCTATTGATCTCTTAAAAGTCTGATTATA

GTTAAGAAGTTGATTCGTTTTAAGCAAAACCATTTTTTATAATACAAAATATTTTAAATTTTTTATAAAATAAAGTTTTA

ACACTTTCAAGTTTTAACAATTTTATTTTATTTGCCTTCTCCAAATTATCTCCTTTAACTATGTTACTAAGATGAAACAT

GGCGTAAGTCAGTGGCAACTACTGGCAACACTGCGGAATTCACCAAATTACTTCAATAGAAAAGATTATTACAATTTTCA

CGAGTTCAAATCATCACAAAGTTCATTTGAATTAATAATTAAACACAATACGGCCATAAGCGTAAGTTACATGCATTTTA

AACAGTTTTTTCACTCTCCTGTAAAGTAGCCATTTATGACTTTCGTACTGTTAGAACTTCACTGACGATAAGTATTGTTG

AATTAAGTAT

>SEUC30172_TC01_piggyBac

ATTGCCTATTTATATTCTCTTAACTCGTATCATCCCAACCATAGTAAAACAAATCCAATTTTTATAAAAATCGGATCAGG

GCACGTTTATAGATGTTTAATGAGACCTACATATAAAAAAAAAATATATCTCATTTAGATTTTTAGTTTTGTAACCGTTC

CAATAATGGAACACTAGGATTATACATGCATGTAAGATACTTTTTGCTATGGATTCCGTTTGGAAATAAAACTATCGTAT

TTACCAGTTATTAGTTATTAAATCTTAGAAAAATTAAATATTTATTAAATCTTATAATTATTACATAAAAAAATACAATT

AGGTATGTTATTCAGTATGAAAAATAATAGAACATTTGCTATGTAAGGCAACATTACATTTGATACACTTCATAGTAGTA

TTTTTGTGGCATAAACCACATCGAGTTTGTTTATTTTGTTTGTCAATCAGATGTCCTTTACCATCCAATCTAATAGCATC

AGGCACACGTTTTATTACTGCTAGTGAAGATTTAGGATTTGATATGGTAAAAGTACTAGGGGCTGAATTTTTCATTAGAA

AATATGTACAAACAATATCCCTCGTAAACGATAAAAGATCATGTGGCGTACCGTTACTTTTCTTTGCAGCTGGAGATATT

TTGTACAGCTGAAAAGCGTTATTGACAGACACATTCAGTGGAAACATAAGTAACTGCCAGTACCATCTTTTCAGCCGAAT

CGCGATTCGATAACAACCCACATTTTGATCAAGTCGGTCCACGCCGCCCATAAATTTATTATAGTTTACGAAGCAACGAG

GTTGGTCAACATCCTTCTTCTGTTTATCACAGTATCGTTTGACTTTGCTTATGGGTTCTGAACCACATTGAGTCGAGGCC

ACTGTGACAATGTTATTGTCGTTATATCTAACAAGAGTGATATTAGATAGTATATCAGTACATTGATGATGAGATCCTCT

ACTTGTTTTTTTCATTTCTTTCGGATCTTTGAGAGGTGCTTTCTCAGTTCTGTTAGCTCGTATAGTCCCTGTTCCATCGT

GACCCATTTTTTGAAGCTCTTCTAGCAGCGGTAATGACGTAAAAAAATTATCGAAATAAAAACTGTAGCTACCTTGCGGC

AGCTTTGATATTAGATCCAGAACAACTGAGGCACCAACACCTAACTCAGGATGGGTATTTCCGGTACTGGCTCCCTGATA

AAGCTCACCATAAATCAAGTAGCCAAGTCGCGTACAAATTGACCAACTTTTGTATCCAAATCTGATTGGCTTTCCATGGA

TATGCTGCTTGGTGCCATGCTTCCCATAATAGGGAATCATAGATTCGTCAATGCTCAAATTTTTATCTCCAGGATAATAC

TTTACCCATCTTGCATTGAGCAGTTTCCATAGAGGGCGCACTTTAGCGAACTTATCACTGGGGTCAAGATTATTATTGTC

GGCAACATGGAAATAACGCAACAAATCTTCAAATCGATTTCTCGACATAAGAGCTGCCACTGTTGGAAAACTGCAATCAA

CTCTTTGTTCCCAATACATTCTGTATCTCGATACACTGTTATATCCCGACAATAATAAAATGGCAATAAAATGTTTGATA

TCCGTCGTAGTAATCCGAAATGCATGTTTACCTTTCTGAATCGCATTTTTTTCTGTTTCTGAGCGCAGTAATTCGAACAC

TTCTTCATCAAAAAATAGTTCAAAAAACTGAACAGGGTTTTCAAGGTTTTCCAGGAAATCCGGTTCACAGTACTCCTTCT

CAGACTTTTCTGGAATATCTACAAACCTCCACTTTCTTGTGACTTCAACACGTCTTCTTTTTGGCGGTGGCTGTGTTACC

GTCAACGAGGAGATGGGCTGTGGAGGCTGCGACACTGACGATGTGGAAGGTATTGAGTGCACTGTTTCTTCATCCCCAAC

GAGCTCATTCACTATCCTAGACTCAGGTTGAGACGATCTGGTGACCCGGAGTTCAGCTTCTGCAAGTAACTGATGTCTTG

ACAAATTATTTATACTTGCAAACTCTTCGTCACCACTGTCTTCGTCGCTATTTTGCCAGTTATCAGGAGGTGTGATGAAA

ATATCAGCCGCGAACACGTCATCATCTTCTTCTAAGTGCGAGATTATTTGATTCACATTCAATCTACGATAAAATAATAT

CATAATTACATGTCAGTACTGATGAAATAAAAATCCTAATGTATAAAAATTACATGTGCGTATGTTCCTACTGCTCCAAT

AATGGAACAGTACATAAAACTTACACTTACCCACAAATTAGTAATGTTTTTTCTAAAAACATATAATGATATGATATAAC

CATATTATTACATGTGTATAAACCCTAAATTGTCAATAGTATAATATAAAACATCATAATTACTTACGTTTTTCTGTTCG

CCATAATTTTCACTAAAAACTTCCAAATAAAATATTTTTTATAGGTTGGAAAAACCGCGCGAAGTGAAATGACATTTATT

AAACTGACAGCTAGAAATGTTAGTAATACCAACATAATAAACATTTATTTAAAAATGCGTTCAAAAATTACTAAAAAA

>SEUC46157_TC01_Mariner *°

AGGTGTATGCCCACAGCAGTGAAGAAGCAAGCGCGCGTATTGCGAGGGTCCAGCGTGGGCATTACCCTTCCTCATTCATG

GTTTGGCTTGGAGTCTCATATTCAGGCGTAACTGAGGTGTTTTTCTGTGAAAAAGGTGTGAGAACTAGTGCCGAAGTTTA

TCAAAATACTGTCCTAACAAACATAGTGGAACCTCTTTCTCAGACAATGTTCAGGAACAGGCATTGGATATTCCAACAGG

ACTCTGCACCGGCTCATAAGGCACGAAGTACGCAAGACTGGTTGATCGCTCGTGGAATTGACTTCATCCGGCATGAAGAC

TGGCCCTCTTCTAGTCCAGATTTAAACCCACTGGACTACAAGATATGGCAATATTTGGAGGAAAAAGTCTGTGCTAAACC

TCACCCGAATTTGGAGTCTCTCCGAGCATCTCTGGTTAAAGCAGCGGCAGACATAGATATGAATGTGGTGCGTGCAGCGA

TCGATGACTGGCCGCGTAGATTAAGGGCATGTGTTCAAAATGGGGGAGGGCATTTCGAATAATGTAAATAAAATTATATT

GTACATTTAATGACCTTTCATTTCATATATAATTTTTAAATAATTGTCAAAAGGCGTATAAGTTATTACATCAAAAGTTT

GTGACAGAACTTATGACCCGACTAAGTAGTATAGTGTTGCCACTTTATAAACTAAATAAACTTACTACAAATAAGTTTGG

AACAATACGATAATTACAGTTTCTAAAAGGCCCAAGGTCAAATCATAGCCCATGGAATGTTCGAAATAAATTAATATTAG

TAACGATGGAGTTTCTCACTCGTTCTTCTACATTCGAACCTACTTTTTGAATTGAGCACCTGAGTTCACTGACGAACAGA

ATGACGGAGTAATACTACTATTACTAATTTATAACCTTGTATTGTTTTGTTATTTTTTAGTCAGTAAAAATCTGACACTT

CATTTCGGCACACCAAGGGCCGGAAAAGTCTTATAAATAAAATTTAGTCGCTGAGAATCATAGCGATAGTGACAGAACAA

CCTGAATCAACAGAAGCATTCAAATCAAAACGGAAAATAAAATTCTATTTTAACTTTATTTGTCGTGTTTTTTTAAGTAT

TTGGTAAGTCTAAAATATTAATCAACAACGTATTACTTTAAAGAAGTGAAAGATGTATGTAAACTATGTATTTAAAATTC

TTTTTATTTTAGCTAAATCTGTGATAAGTTATTAGAGTTTTAACATTTTCTAAATTTGTTCTGAGTCTTACGGTTATACT

GAGAGGCACTTAATGATTATTTAAATTATTTCTTAGTAACAATTTTGTTCCCAAAAAAAAACAATGGCTATAGATTGGCT

TATGTGCCCATTAAACAACTCTAGAGTATAACTTCAAAGGAAGGTATTATTTAGAGACACTATTGATTAAAATTTTTAAT

CTCGGATGAGATTTGTTTTTCAAATGAATCTTATTAGTTTTACTTTGTAGATTAGTTGCTTTATTTATTTTTCAACTTAT

TATTAACGCTGTTAAATTTTTAACGCAACTTGTAAAGAAAGTTTTCAACAAAGTTATCTAAATTAAAAATTTTAATTTGT

GTGGGGCTTTTAATTAGTTTGGTGAATTGTTGTTTAGTATTTAATTTTTTTTTATTAAATGTGTATTGGAAGCATAGTTT

CTTATACCATTGAAAACTTTTAAATTTTCTTTGCTAATTACAAAAGTTTTACAATTTATTTTAAATATTATCCTATGATA

CATACTCTACATACATAATATACATTTTAAATATTTATTAAAATTCCATTACAAAAACTCAACAATTACTTTTCTAGTCA

AATTACAAACATTTTTTCTAACACTAGTTATCTTGCTATAACTATGTGTATTGGTCACGTTTTGTATTTATATGTGACAT

GTATCCCTTTAAAAATTGTAAATACACCAAAATAAATAGGTAATCATTATCTAATATAGCAGTTAAATCGAAAAACTTTG

TTTTTACCTATTAGGAGGATTCTGTTTGACTTAGGGCTAATTTTTTAATGGTTAGATAAAAGTTACCTAC

>SEUC33946_TC05_piggyBac *°

AAAAAACTAGACAAGATATTAATATAATATCACACTTTTGGATTACTGATTAATTATTTTTAAGAATAATTAGTTTCAAT

TGTATCACAAAAAAATATTTAAATCGTCAATCCTAATTCCCATTTACTAAGTTCATAGTTGAACTTTAAATGCTCTCCAT

TACAATTACATTTATGGTAGACAAGATACTAATTTAATAACCGACTACGGCAAAGCAAAATGAGGGTTATGATTTTGACA

GGCTATTTAACCCTTAATAGCCTAGAGTTTCAATTTTGATACCAAGCTTTTAAACCGAATGTTCTCATTACTATTCGTTT

TTTTTTATATTTTGGCAGTACGTATTTTCTTGCTGTCTAATATTTTTTGCAACGAAATTAAAAAAAAAAACAAATGTCAT

TATAGTTTCATTTTATCTGTGACATATTACTTGCGCGGCAAAGTGAAGAAGTGAACACATTTTACTAAAAATTTTGTTGA

ATACAAATTCGATATATTTTATTTTTCTGAAGAATATAAATAGTTTTGAGGTAAGTGAATATATTATTTTCGATAATATA

TAATTATTTTTTGGGAGTGAGTGTTTTACTTGAAGAAACGTGGTATTTTTGTGAGATGTGAGTGATCTATCCACTAATTC

TATAGCTATAAAAACTTCTTGAATTGGCTTGGTGTTGGGATTACTTTCATTTTTATTTTATTATAGAAATTAATCAAAAA

TGCGACCTGAAGAGATTGAGAGCATATTGAAGCGGTTAGAAAATGGTGAAATTTCGGAAGACGACTCCACCGATAATGAA

GATGACATAGACTATTATTCCAGCCAAAGAGATCGTCTGATGGAGTTAGAAGAGGAAGAGGATGTAGGTAATTTTCCAAC

TGATCCAGACTTGGATGCAAATCCGCTTGCAGCTAACGTAGATACAGATATGCAACATAATTTAGAGACTGATGAACGAA

GCATAACTCCTACAACCAGCAGCCAGAACAACCTGCAAACTGCACCCTTCAACTCTAGAAATTTAGTGTGGAGAGTAAAG

AATATGGACATAGTCCAAAACGCTTTCCAGTTTGCTGGAAACACTGAGTATTCACAAGAAATTATGAATTTAGACACGCC

TTTTCAGTTTTTTTCCTATTTTTTCGATGAAGAAATGTTTACATTTTTAGTGGATGAGTCAAATAAATATGCCTTCCAAA

AGAATCCCAACTTCACAGAACCTGTAACTGTCTTAGAATTACGTAAATTTATTGGAATTCTTATTTTCAGTAGTGTATAT

CATTATCCTAGTGTTAGGTCATACTGGTCAAACATCGCGAAGTTTGAACCCATAGCACAAACAATGAATCGCAACAGATT

TGATAAAATTAGACAAATATTTCACATGAATGATGACTCTAAACATTTGCCCATTGATCATCCCCACCACGACCGACTGC

ATAAAGTAAGACCAGTTATAGATTATTTGAACAGGAAATTTATTACTGTCCCATTTGAGCATCGTCTATCACTAGACGAG

CAAATGTGTTCCACTAAAATAAAACATTTTATGAAGCAATGTTTACCCAACAAGCCCCATAAGTGGGGATTCAAATTATA

TGTTTTATGCTCTCTGTCTGGGTATGCCTACAGTTTTGAGATTTATTCAGGAGCTAAGGACATAGGCCGTTTACCTGGCG

AACCAGACCTCGGAGCTGTATCAAATACAGTTATTCGATTGTTACGACCAGTACCAAGACACGTCAATCATATAATTTAT

TTTGACAATTTTTATACAAATATTCCTCTGCTACACTTCTTGACCAACGAAGGTATTTATTGTCTTGGAACAGTACAAAG

AAACAGGCTTGGAAAGTCGTGCAAGTTGCCGGAGAAACGCGAAGTTATGAAATCTAACGTACCCAGAGGAACATATCACG

AAAATGTGGCCTCTCACGAAGGCCAAGAATTCTCGGCCACAAGTTGGAAAGACAACAAACAAGTGCTATTACTTTCCACG

TATGTTGGTGCTGAACCAGCGGATACCATAACCCGCTACGAAAAAAAACTGAAAGCTAATGTTCAAGTATCATGTCCTCG

AGTCATAAAAGAATACAATGCACACATGGGCGGCGTTGATTTGATGGATAGCTTCATTGGTCGATATCGCATCCGTATAA

AGTCGAGAAAGTGGACAACGAGGCTCTTCTACCACTTGTTAGACATGACTGTCACCAATGCTTGGGTACTGTACAAAAAG

GTAAACACAGCTAAAGGAAAACTTCAAAAGAATATTATGACGCTAGCAGATTTTAGAACTGAACTGGCCGATACTTTATG

TAAATATCAAAGTTACTCGAAAAATAAAAGAGGAAGACCCAGTACCAACTGTCGACAAGATACTACAGTGCCTTCTAAAA

AACCCCGGGCAGGTGTACAAGTACTACCCTCTAGTGAAGTGCGTTGCGATTGTATTGGTCATGAAAAAGTATTTATGGAT

TCTAGAAATAAGTGTAAATTTAATAATTGTAGGAAACTTACCTCATGGTTCTGTAAAAAATGTAAAGTGTCGTTATGCGA

TAATAAAAACAATCAATGTTTCACATTATTTCATGCATAGGTATATCCGATTTTTTATGACCTTAATGCCTGAAGTATCA

AAATTGATACCAGGTATTTTTTTCCTAAATAAATGTTTAAAAATAATATTGTGATTTTTTTACTATTTTTCCCTTCATCT

AGACCCTCAAGCAAATTCAATAAAATTAAAAAAGAAATCATTTTCGTAAATCAGGCTATTAAGGGTTAAGCATCAATTAA

TCAAATAAATTTACTTTACACAGTCTTATCAACTATATCAACCATTGGTATGTGAGATACAATAAGAAATATAATTTTTA

ACTCACATGTGGTATAAAACACACCTTGGTTATTTCCTCTTGCTGATTATGTATATTTGGAAAAACACTAACAAATGTAT

GACTTTCTTCCTGATATGGAAACTAAGAATAAATAGTTGCATATGACCTTTCTCTTTATACTAAACAGAACAAAAGTTAT

TGCTTCATATTAGAATACATTAATCATACTAAGACACATGGGTATATGAATGTCCAGCTTGCATTTGTGCAATATTGAGC

AATATTAACAACATTTTAACATCATATTCACAAAGCAATCTTGAACAATCAGCCAAGTATTCAGCATTAGGTTTGCTACC

TCTTCCTTTCACCTTTTTGTGTCTTCCGCTTTTCTTTTTTCTCCTCCTTCAGTTGCTTTTGCTTTTCTCTGTCTTCCTTA

TCTTTTTCTTTCTTTTTTTGGGCATCAAGCCGAGCCTGTTCACGGCGTTCACGAGCAGCAGCATGAACATCCGCTGCATT

TGTATTCTTAACTAGATCAGGACTAGCAGCTTCTTCATCGTTTGCAATTTCTTCCTCATTATCAGAAAGTTGACTATATT

GTGACAATATAGCCTCACGTATTTTTTTCTCTTCATCGGTGTACTCTCGTCGTGTCGTTGTGGATGGAGCTTGAGATTCT

AACAACTGAGCTAGTCTCACATCTACGTCTGTCACGGGTTTCGGGCTTTCTTCCTTTGGTCGACATGATTCCCACTTTTC

TATTATCTCGTCTATATGTGTTGAAATGTCTTTCTCCACTATCTCAGAAAGTATGCCTTCTAAAGCATCTTTCTTTTCAT

CCACACTGTCTTCGCTTTCTAAAATACCTGATATGTAGGATCCGAATACTCCTTCGTCAGTTTTTAACGATTTCAACTTT

TCACTTAGCCATGGCTCAAATTCACTCGCAGCCGTCGCCATATTGCTAGAGTTTTTTAAATTTCAATTTTCAAAATGTTT

TTGACATTTTTGAGGTTCCTTCAAAGTTGGTTAGATTTTTTTATAGGTAACATTGGCGCTTGGTGCAAAAGTGGACAGCA

GACAGATTACATCATACATAATTATTTAGCACGACATTTTCCACAGATTTAAATTTATTACTAGCTACCGTTTCTTAAAC

AAAATCGTTAATATCTATTAAACGTCTCATAAAATCATAAAGATGTATCACAAAATTCTATTATATTGATTTCCGAACGA

ATTATTATCGAAACACCGTTGTCATGCTGCAGCTAGTGATGTATTTCATATCGATATTTTTTTAAAATAATCTATAATAC

ATTATTCTTTAAACCCCATGATATTTTTTGGACTGAAACCATTGTTATAATAAATATTACATGTACACAATAGTAAGCAA

ATAAAAAACAGAAACATGCTTGTAAAGATTCTATTTTATTTTTATTTTAAATATCTCTTCGACATCTATCTGTACCTGTC

AAGTTTCACGTCCCTACACAGTTTGCGGCGAACGAATAGTTTACGGAGTGACTTGTAAAATATTTGTATTCTACAAAGTA

AGAAAATGTCTGCGAGATACGATAGAGCAATCACAGTATTTTCACCTGATGGTCATTTACTTCAGGTAGAATATGCTCAA

GAGGCTGTTCGTAAAGGATCCACTGCGGTCGGCGTTAGAGGCGCTGATGTAGTTGTACTAGGTGTTGAAAAGAAATCAGT

TGCAAAATTGCAGGAAGAAAGAACTGTGAGAAAGATCTGTCTCCTTGACGACCATGTCGTAATGGCTTTTGCTGGTTTGA

CAGCAGATGCTCGAATCCTCATCAACAGGGCTCAGATTGAGTGCCAATCGCATAAGTTGACGGTTGAGGACCCTGTGACT

TTGGAGTACATTACTAGATATATAGCTGGACTAAAACAAAAATACACCCAGAGCAATGGACGTCGACCTTTCGGTATTTC

TTGTTTGATTGGAGGTTTTGATTATGACGGTCATCCTCATCTGTTCCAAACAGAACCATCAGGTATCTACTATGAGTGGA

AGGCTAATGCTACAGGAAGGTCAGCTAAAACTGTTAGAGAATTTTTGGAAAAGAACTATACTGCTGATGAAGTTGCTACT

GAAAATGGTGCTGTAAAGCTAGCTATTCGTGCTCTTCTAGAAGTGGTCCAGTCTGGACAAAAGAACTTGGAGATAGCAGT

GATGAGACGTGGTCAACCTATGCAGATGCTGGATTCTGATACCATCAGCTCATATGTGTCTATTATAGAGAAAGAAAAGG

AAGAGGAAGCTGAGAAGAAGAAACAAAAGAAGTAATATCTTAATATGTTGCACACTTTTCTATATAACTAATACATGTTT

TTAAGGAAATGACAGAGTTGTTTATTTTTATTAAAAAGCTTAAGAATTAAGCAAGTCAATAGTTTTAGAGATT

>SEUC36321_TC05_hAT *°

TAAGTATCGATACTTACTCATTTCGATAATACTCAAATCTTTTTTCGATACTATACAAATAAGTACTCCTCAAGTATCGA

TACTTACTGATATCGATACTTTTTGGAACTTTTATTTTATTATTTTGAGGGTGCTATCAATGAACGTTGAAGGATAATAA

TTTAAAGAAAATTGAGACGAAAAAAACGATTTATTTATTATAAATGTTTATTAGGCTTATTATAATTTAAACAAAAAACT

CTTCAGCATTAAGATTGCCCAAAAACAGTAACTGTTCAAGGTATTTAGAAGACAATCTGTTTCTTGTTTGTGTCATTGTT

GCACCGGCTTTGGAAAAAAGGCACTCGCTAGGAACTGATGTTGCAACAACAATTAAATATTGCTGTGCATACTTGTACAG

ACAAGGAAACACCAACTTCATGTCATCCCATTCTGCAAATGGGTTGCTTTTAAGAGAAACTACTGGATTTGATAAATAAA

GAGAAACTTCATCTCCCTGATGAGATTTTTTTTTCTTCTTGTGCCCATGTGCTAACTCTTTATGATGTTTCCAAAAATCG

TATTCAGGTGGTGCAGTAGATGAGACATCATCTTCTGACTCACTTGGGCTACTTAGTTGTCCGACAACCATGGTTCTAAG

TTTTTGAATAGCTCTTCCGCATGCAGAGGGGTCTTGGAAATGCAGATTTTTAAACCTTGGGTCTAGAATTGTTGCAATGG

CTGCATGATCGTTTAATTCAATCATCCCATACCTCCTTTTCAGCTCTCTCAATAAAACATCCTTACATTCTTTAACGATA

GCGGAGTTTGGAGTGATACTATTCAGTTCTGTAGTTAAGCAATTAAGCATTGGTATAATTTTGGAGATTGTTACATACTT

TTGGCCCGATGATTCTCTTGTTACATATTCAAAAGGCTTTAATACAGTTGTAAGCTCATTCAAGGTTTCCATTTCCACTG

CACTTGGCAAATTTGGAGCCACTACACTCTCAATTACAATTTCTGATATTACTATTCTTAACTTTAGAAATCGCTCTAAC

ATAAAAAAAGTTGAGTTCCACCTAGTTCTTACATCTGCAATAAGTTTTAAAACTGATCCTGCCGAAACATTATTACGCAT

TTGGATTTTTCGTAGCTTATCCGACTGCACAACACTATTTTTTATCCAATTAACAATTTCTCGTACTTTTATAACAGCTG

ATTTAACACTGGGCAACTTTAGTGCCTCTTCAACTACAAGGTTAATAGTATGGGCAAAACACGGTAAATGTTTTTTTTCG

CCTAAAGACATTCGTATGGCAGCCAACATTGAACTGGCATTATCTGATACAGCAGCACGAATTTTGGTAAGTGGAATATT

CCATTTTTTCAAAATATCCAGCAGGTTTTCCGATATGTATTCAGCTGTATGTCGCTCTCTTAATGCACGAGTTGCAAGAT

AATACATACACATTTGACCATTTTCTACAAAATGTATGGTGACGCCCATGAATCCTACTTCATTCATTGTCTCTGTCCAT

ATGTCCAGCGTTAAACAGAAATTGTCTATCTTGGATAATTTGGACTTACAAATAGTTATACAAGCTTCGTACTTTTCGGT

AGTCATTTTCTTTAAATACTTTGCGCCAGGAACTTTAAAAGTAGGCTCCAGTTCTTTTACAAGTCGTAAAAAGCCTTCAC

CTTCAACTATATTAAATGGACGCATATCTTGACAGATAAAAAATATCAATGCTTGAACAATTCTGTTATACTTCGTACCA

CCAGCTTTATTAGATGAAATTCGTTCAAACGCATTCTTTATTGAATTGCTCAGACATAGCTCCCTTGCAGTCTCCGAGTC

CATACTTGTTAATGTGGTTACACTAGAGCAATCATCACGATCTTCTGAAATTTTCGATGTTGATGCTTGGGGTTGATCAT

CTTCCATTAAATGCCTTGAGTTGGCCAACACTATTGTATTTTTTGAACTTTCGTTGGACTTATTATTTTCTTTTTTCTGT

AATTTGCCTTTGTCCACAGAAATTCCATGAGTTTTAGAATGCTTGAATAAATTCGACGTATTCCCACAATACTTCACAGA

CTTAAAACACTGTTTACATTTTGCAATTGTTTCGTTTGTTTTGGTAAAATGTGTCCATATTATAGATTTTGGTGGTGCCA

TGATAACATTGGAATAAACCGATGAATTTGATGATTACAATTAATAAAATTACTTGTAAACAATCACTTAACACTTTCAC

AATGGCGGAATGGCGAGATGATTTGCGAGCGGGTGCGGGTGTTTTCTTCTCTCGCTTTCGGCTCGGGCGGGCTCGGCTGA

AGTCGGACGAATTCGGCTAGCCCAGCGCGAGACAACGCGTCACTCACGCCGCCACGCGACGAATACCGGTAACTCGGTAA

CCTGATTAGCTGACTGTTTTTGCCTCGTTTTTGCTCTCTTAGTTCTGTATGATGCGTTCTCGGTTTTTTTTATTGTATTC

GGTTGTTTTGCTGATTCGATTCGATACCAAGTATTTTTGTTTCGATACCGATTATGTATCGCAAAATTGATTCGATACAA

TAAATACTTGGTATCGAATCAAAAGTATCGAGTACCTACTAGTATTTATTAGTATCGGAATATCCCTAGTCGGTCGGTGT

TCGTCGGTGATGATTTCGTTTCGTATTCCGTAATTTCGCTATTTTCTACCCGTTACACATTATTTATTTCTCATGAATGT

TTTCAACCGTGTGATTGCTAAACTTGTTCTTCTTTTAATTTGAAAATTGTAATTTTTATTCTGCGATAATACATACAAAA

TTGCCTTCAGGTTATTATGTTACTCAACAAAACCAGGCTGGTGGGTTTTACAAATAGATCTCGCAAGAGCAATATGGAGG

GTAACTCCACGTCAGATATTGTTATCATAAGCGGTAACTCACACCCTGAACTAGCGGAGTTAATTGCTAATCGTCTTGGT

GTAAGGAAGGGAGGATGCTCAGTATATCACAAAACAAACAGGGAAACTATTGTTGAGATTGCTGATTCCATTCGTGGAAA

AAATATTTACATAGTCCAGACTGGCACTAAGGATGTAAACAACAACATAATGGAGTTACTTATAATGGCATATGCTTGTA

AGACTTCCTCCGCAAGGTCTATTGTGGGAGTGATCCCATACCTGCCATACAGCAAACAATGTAAGATGAGGAAGCGTGGA

TGCATTGTCACCAAACTTCTAGCCAAAATGATGTGCAAGTCAGGCTTGACACACATCATTACAATGGACCTTCATCAAAA

GGAAATTCAAGGTTTCTATGAATGTCCAGTTGATAATTTGAGGGCTTCTCCTTTCCTACTGCAGTACATTCAAGAAAGTA

TTCCAGATTACCGGAATTCGGTCATAGTGGCTCGTAATCCAGGGTCGGCTAAAAAAGCAACTTCATATGCTGAACGTTTA

CGATTAGCCATTGCTGTAATCCACGGTGAACAGAAGGAAGCAGAAAGTGATGAAGTTGATGGGCGCTACTCACCTCCTTG

CTTACCTAACCGATCTCGCACCATGGATGTATCTGTTGGAGTTCCTGTACATCCAGCTAAAGAAAAGCCACCTATCAACG

TTGTTGGTGATGTGGGAGGCAGAATAGCTATTATGGTGGACGACATGATTGATGATGTCCAATCATTCGTTGCCGCTGCA

GAGGTGTTGAAAGAATGCGGAGCGTACAAAATTTACGTACTGGCAACCCATGGGCTTTTGTCGTCTGATGCGCCGCGTCT

TATAGAAGACTCGCCTATTGATGAAGTCGTGGTCACAAACACCGTACCGCACGAGCTTCAAAAGATGCAATGTAACAAGA

TCAAAACCATCGACATATCAGTGCTGCTCAGCGAGGCAATCAGACGCATACATAACAAGGAATCCATGTCTTACCTGTTC

AAAAACGTAACCCTCGAAGATTAGTCACACCTGATGTAGCTTTCCATGTTTTAATGTTAAGTATTAATTAAGTATTATAT

TCCTAATTGAGTTTTATAGTCACTGTTTTCACTGTATTATTATTTGATTAATTTTATAAAATTGTCATTGATAACATTGA

TGTTGATCTAGAATGAAGTATTTCATAAAGTATTATATCTGTTACCTAACTGGTCAAGTGGTTGTATGAGCGTCCGCTGA

GTAAAGAATTGATGCCTGGATTGG

>SEUC41948_TC13_Gypsy *

ATGCAGTCTGTGTTCAACTTTGTTATGAGCTTGCTCTTAGGCTTGGTGGGTCGTCGTAACATGGTTCTGGTGGTGACCAC

ACTATGCGGTGTAAGTGGTATCCTGGTCAACCTGGTGCCCAATGCCATCGCCAGTGCCATACTCTTCGCTATACTACTTC

AAGGAGTCGTCGTCATTGGGTTGTACACTGCGATTGTGGTCGCCATATTCCCCACAAACTTGAGATCGATTGACATCGCT

TGAGGAAGACCGGCGTGTGTCACGTGCAGCATCGCTGCGACAACCGTCCAACCAGCAGCAGCAGCTCGTGGAAGAGACTG

CGACTACGGCACGAGATGGAGCAGGTTTTCGTGACTCGGCAGCTCCGTCTTCACCTGGACGTTCCGTGAGTGCAGCCGCC

GATCGCGATGCTACCGTGACCGATCGTTTGATTGACGCCATACGATCCATCAACACTACGGTAAGATCCAACCAAGCTTA

TTTTATTTCTAATTTCGATCCTAGTGTCAATGATATAGACACTTGGTGCGAGGAGGTAGACCGCGCGAAGGTTTCAAATA

ATTGGACTGATAATGAATGCTTGTCCCGCATTGGGAATTGCCTCAAAGGAGATTCGAAGACGTGGCTCAATGAGTGGGTC

ACTAGTGACCGCACATGGAGCAACTTTATAAGGGAATTCAAGCCCTTATGTCCTAGGAAGCCAGATGTAGCGAATATCCT

ATACGAGGTTATGAGCACAAACTCTGATAGGTACCCAACATATGCCGATTATGCCCGTCGCTCTTTATTGAGACTCCGTA

TAGTCAAGGGCTTAAGTGATGAACTCATTTCTGCAATTGTGATTCGAGGCGTAATGGATCCTCAAATCCGCGCCGCTGCC

ACAAATGCAAAGCTAATGCCTAACGAATTAGTAGAATACCTCTCCATCTACGTTAAATCGAACTTAAACTCAAAAAAGTA

TACCAACCCTTCTAATAATGATCGTTCTTTTAAATCTACCCTCGAAACAAGTCAGCGTAAGCGAAAATTCGAAGATGGCA

CATGCTTTTTGTGTGGGAAATATGGTCATAAGTCTTCGAATTGTCACAAACGCCAGGCTGCTAACAGTTCTAATAATAGC

AATGGAAATGTACAAAGAAATTATCCAGTCTCCAGCTCAAGTAAAACAGAGCCATGTAGTTTTTGCAAAAAACCTGGTCA

TAGGGTAGAAACGTGTTTCGCTAAACAAAAATCAGAGTCGAATAACGCGGAAAATGTTAACTTTTGTCGTAAGAATCTTG

ACAGCGGGCGTAACGATATTGTTGTAGCGGTCATTCAAGGCATACCTGTCGATGTCCTCATCGACAGCGGGTCGAGCATA

TCACTGATTTCATCTTCGTTACTGAAATATTTTAAATGCGCGCGCAAGCCTGCCTTCAGAATTTTAAGAGGTATCGGTAG

TCAAGACGTTGAATCTACTTACTATGTCACGTTGCCGATAGAATTCGATGAAATAACCTTGGAGGTAGACTTGCATGCAG

TATCGCCAGAGTTTCTAAACACGCCTGTTATTGTTGGGACTGATGTGTTAAATCGTGACGGAGTGACCTACGTTCGTACA

CGTGATCGACAACATCTTGCTTATAATAATAGGTCGCCATATAACGTTATGTGCGCTGTGACAATACATGATACCCCCAT

TAAGACACCTTTGACCGGTCGCGACCTAGAAAGGCTGCTTGAGTTGATCAACGAATTTTCAAAATTCTTTATTTCCGGGA

CTGCTGCCACCACAGTCAATACTGGCAGCATGTCTATCAAATTAAATAATCATACGCCTGTTAATTATAGACCATACAGA

CTTTCGTTGCCGGAAATTTTGAAAGTTCGTGGAATTATTCAAGAACTCCTAGACAACGGTATTATCCGAGATTCCGAATC

TGAATATGCGAGCCCAATCCTCCTAGTAAAGAAAAAGGATGGATCAGACCGCATGTGCGTAGATTATCGCAGGCTTAACG

GGATTACTGTCAAAGACCGTTTTCCTTTGCCGTTGATTGACGACCACATTGACCGGTTAGGGAAACACAAATTTTTTAGC

AGCCTTGATATGGCAACAGGGTTCCATCAAATCCCATTGGACGAAGAGTCGATTCCGCTCACGGGATTCGTCACACCAGA

AGGGCATTATGAGTATTTAAAAATGCCCTATGGTTTGGCGAACGCCCCCGTCGTTTATCAGCGTATTATATCGAAAACAC

TTCGCCGTCAGCTTGAGGCTGGTAAGACCCTTGTGTACATTGACGATGTGCTGATTCTTAGCCATACGATAGATGAGGGC

CTTATTCTTCTCCGCGACGTACTTCAGACACTGACTGCGGCAGGTTTTTCTATCAATTTAAAGAAATGCTCATTCTTATC

GACTCAAATTGAATATTTAGGCCGCACTATAAGTCAGGGTCAGGTACGGCCGAGTGAGCTAAAGGTCCAGGCACTCGTTG

ACGCCCCCACTCCAAAAACGGTCAAGCAAGTTCGTCAGTTGCTAGGTTTGGCAAGTTATTTTCGCCGATATATATCGGGT

TTCGCTGCTAGAACGGCCCCGATTACTAAACTCACGAGAAAGGGTATACCGTTTCTTTGGGGAAGAGAACAAGAAGAAGC

ACGCCAAGAAATTATCTCACAATTAACCAGTGAGCCTGTGTTGGCAATCTACGACCCTAACTTGCCTATTGAAGTCCATA

CGGACGCAAGTTCTATTGGTTTTGGCGCTGTGCTTCTTCAGGTTCATGAGAACGGATGCAAGCAAGCGGTAGGTTATTTT

AGCAAGCGAACGCAGGGTGCCGAGCCCAGATATCATTCGTACGAGCTCGAGACGCTAGCCGTTGTCAAGGCCCTGCAACA

CTACAGACACTACCTGGTCGGCGTTAACTTTAAAGTAGTGACCGATTGTAATGCACTGAAATCAACGCAGCAAAAGAAAG

ATCTCTTGCCCCGTGTTGCCAGGTGGTGGATGTACCTACAGGACTTCGACTTTACCCTAGAGTATCGTAAGGGTACGTCT

ATGTCGCACGCAGACTACTTGAGCCGAAATCCTGTTAATGTTTGCGAAGTACGCAGACAACAGAATTGGGCTCGAATTGC

GCAGGCGGCTGACGAAGAGACACAACAGCTTATCCAAAGACTGACAGACGGACAGCTAGACACTACTCATTACGTTCACA

AAAATGACCTGCTTTTTTATAAGTGTTCTCCAGTGGGAGAAGAGCCCAGGTACTTATGCTATGTACCTAAAGGTCATAGG

CTTAGTCTGCTTAGGATATTTCATGATGAACATGATCATATTGGCGCTGATAAGACTTTAGAGCTTATTAGAAAACATTT

TTGGTTTCCTTCACTAAAGTCATTTGTCAAAAAATACATAAGCCATTGTATCATTTGTTTAACCCATAAAAAGATTCCTA

GAGACCCACAGCAGCCAATACATTCTTGGGAAAAACCCTGCACCCCTTTTGAAGTAATTCATACAGACACATTAGGTCCT

CTACCTCAATCAAATGGTTATCGCTATGTACTCATTATAATCGATGCTTTTTCAAAATTTTGTCTTTTGTATCCTATGTT

TGGTCAAGACGCTAACGAACTGAAACGACTTTTTACAAATGTCATTTCTCTATTTGGTACACCAAAATTAATAGTAGCAG

ACCGAGGCAGAATGTTCCAGAGCTCAGAGTTTACAAAACTCGTAACTGAGTTGGGCATTGATTTGCATCTGATCACTCCG

GAGATGCATCATTCAAACGGGCAGGTGGAGCGTTACTGTCGAACATTGTTAAACATGATTCGCATACAATGCAATCATCG

TCAAGATGAATGGGCAGACTTGATGTGGCGATCTCAGCTAGTTCTTAACATAACAAAGCAGAAAACCACTCAGCTTTCAC

CATTGAACTTGTTGGTTGGTATTGAAGCTGCTACGCCCCTCATACGTCACCTGGTGCGCGACGTGGCCTTGGATAGTTCG

CATCCAAATCGTGAAGCGCATAGAGAAATGCACAGACAACGAGCTTCGGAGCGTTTAGCTCGGAACCAACGTCAACAAGA

CGAGACTGTGAACGCTGGTCGAAAATCCCCCCGATCATTTGAATGTAACTCACTTGTTTTTGTTATTAAGCAGGCGCAAT

CGACTGGCAAGCTTGACTCCGGCATGCGCGGACCCTATCGCGTGGTCAAAGCGCTTCCCCATGGTCGATACGAGCTGCAA

CTACTGGCAGGTTCCTATGGCAAATCGACGCAAGCTGCGGCCCAGTTTATGAAGCCGTGGCGCGGGGAGTGGACTCCAGA

GACATGCGCTGCATACTTTGATGGCGCAGATAGCAATGATGATAATGCGGAGTCTACGCCTGAGCAAGCTGCCAATGTCG

ATACGCAACCCACCATCGATACTTCGCCTGAACCGATGCCTAGCACATCCAGAGCTGCAGAAGCCCTGCAGCCCCAGCCG

CAGGAGAACGGGGACGTCCTCCAGTCAGGAGAGGCCGTATTGGCGGAAGAGTGACGTCTTGGCTGTCATCGGAGGGGATG

TTGGTGGGAGAAGCTACGGTTGCCTATAAAAGGGCCTGCGCGCTCAGTGGAGCCCTCTTTCTGCGTTGAGTACACATGGT

ACAAGGGAACCTCTGCTCGTTTGAAGGGTTAAACTGTCTTGTCTGCTATTAATTTTGCGTGTCTTTAGTTTAGATTGATC

TATGTTTGTAATTTCAAAATTCGTTATTTAATTAAAATTTAAATTGTTGTTTATTGTAATTGTGTTATTAATAAATAAGA

TTGTGATTTGTATTTTTTTTAATATTTTCTTCCTGAAGTTGCCAAAATCAGAAGTGGGATACGCCTCGCGTTCCATTTCG

TTTTGTTCGCCTTGAATCAAGACAGTTTAAAACTTCGTAACGTATTTTTTTTTCTTTTTCTTAAGTGGGTACATTTCAAC

ATTTAAATTAACTGTTAAGCATTTGTAACGTCCGCGTAATTAATAAGGTTCAAGTGTAAGTGTGTATTAGTGGTTTTGGT

GAGTACGTTCCAGTTTTTCTTGATATTTAGAAAGCAAATTCTTTTGTTTACGTATAGTTGTAGAATTTTACAATCATATG

TTTTGATTTGTTGCTATTCATACAATTGCTTTGATTTATTTAAATTTTCTACCGATTCCTTTTATTTGATTTGAAACAAG

TTAGAAAGAAACAAGATGCCTAATAGCCATAGAAGAAGAGGCCGTTCAGTGAGTCGAGAATCGCGTCATCATCGTCGTGA

TGTGCGCTCTAGGAGCCGTGAATCTCGCTCTCGGGGTCGCATCTCACGTTCTAGGGGTCGTGAATCGCACAATAGTCGAC

TTGATTTGCAAGGGAATAGAATTCCTAGGCAACCTGATATTGCTTCTAACAGTCTATTAACTCAACAAATGCAACAAATT

TT

>Tni_Contig_14_Sola *°

CGACCCATCCGAAGAACAATGTTACATTTGAAATTTACTAGGTTTTCAGGAGAGCCATTCAAAGTTCACCCCATACTTAA

ATGGTAATAACTTTTTTAATTATTGAAATAAAGTGATGAAATTTCACATATACTTTAATTACTTATTTAGACAACATTTT

AATAGAAAAGTATCGTATTTATGCTATTATTTTCTAAAATAAGGGGTATTATAATCACATATTACTTTGTTCCGCGTGAA

GTCAATATAACTAAGTTAACATAAAACAGTGTTTTAGGGAATTGGGTGAAGATTTTGAGATATATTTGTATATAGTTCAC

TTTTCTTTGGAGTTCCAGTAATGTGTGACATTCAAGATGTAACATTTGCAAAATTAAAACGAATTATAATATCGACTCAG

TGAGTTTTATTATGAAAAATGTAATAATTATTGTAACAGCTTATTTTAAAACTATTTCAGTGTAGGTAATAATTGACTGA

TAGGCATAATTTTACCTATTTAGGTACCTATACTTAATTATAAAAATAGTTGGCTAGGAAGTAAAACTAATTACGAATAA

TAAAGTTACATAAAACTTTGTACTTTTATATCACAGTTAATTAACATTAATATTTATTGACCAGTACGCATTAAATGGTA

AGTATTGAAAATCACATTTTTGGTAAGAATGTCATATGAAATTCTGAATGAAATTAAATTCTCACATAAAAACTTTATAT

AAACTAAACAAAACTTAAAACAAACATAAACAAWTGCAATTCTTAATTTGAAAATAAAATTTCTAATAATCAGTCTTTTT

ACTYCAAGTGACAAATTAGAAACCAATAAAGCAACTCTAAATCAACAAAGAAACTCTNNAAAAAACAACRTAATATTATA

AGTTTAAAAGGAAAAATAATCAAAAATCAAAAAAATACTGTAATCATCGTGCCTACTAATTTTCTTCATCATCACTTGTG

TTGGCATCACCGTTGGGCAATGTTTTGAAATAATCATGATATTCAACGGGTATAATATTTGTATTGCATAAACTAAGAAG

ATCTTTGTATTTATCCCGCGAAATAGGCAAAAGCTGATTATAGGCCTTTTTTATTTGCATATTTTGATTGCCTCTTGAGT

TTCTAGTTGTATTACCGATAGTTATAGCATCATACATTTCAGCGCTTAAATCGTATTTATAATATAGYTTGTTTGAATCA

TTGCAATCTACGTGTACTTCTTTAATATTGTTCCAATTAACTTTCTGACCACGACAATTTTTACTCCATACTTTATCAAC

AACCCGACTTTTAAAATCGTAAAAATGCTCTTGAGTCATATTTTTAACTTCATACGGATAATCATTATTTTTACTCCATC

TCACTAATAATTGCCACTCTTGAGGTGTATAGATTATTTTGGCACGGGAGGCATTTTCAATACATGCATGAACGCTATCA

CCTTCGTTCTGCGTGTGACCTTTCTCCATAAAGCGATGTGTGATGGTCACTTTGTACTTCTTAGAAGCGTAAATGTAGGA

TGAGTACACGATTCGGTTTCTATTTTGACCGGCGCAGTTGTCAGACCAAAAAAGGAACTCGCGAACTCCTTTTTGCACGT

TACAATCAATAAAATCAATCAAACAACTGCTTACATCGTTTGCTCCACGCTTAGCTATCGTTTCGTCCCACATATAACAC

GTTCCATGTTTATTACCCATATCAAAAATTGTAAAATTTAAGCAGCTTAATTTTCTTTTGTAATAAAAAATACTAACTTG

TCCGTGAGGACAAGTCAATACCTTTTCAAAATCGAACACMGCACAGACTATTTCACCATTAGAATTTTTGGCGCTCTCTT

TATCTGCAGTTTTCATAGCTCTTGCAATCTTTTTTCTATCCTGATGTTTAGCAAAAGTATCTTGTTCCACTTCAGAAGGA

TTTTTCAAATTTTTAAATATGTGACATTGGTCACATTGATCTTTTTTAGGAACAAAAAATGCCAAGTTAAAGTGGTCATT

GACAATATCACGGTATTGCCTTTCTGTCCGTGCTTTGATTGAGTACTGTTCGTCGTTGAACCACTCCTTGTATAAAGTAA

ACATGCGGGCTATACTTAAACTACCGTCCAGATATAATTTAGTRCTTTTCTCCCTGAGATAATGGGATTCCACAGGTTGG

AAAGACTTGACGTGGTCACAAACGCTACGAATCATTTCATCGACAAGTACTATTTTGTGACTCTTGTGACGACCTCGTTG

ATCCTCTTCAACGACTGCAGTACCATCATACTTTGACCAAGCAGTTTGTAATACTCTGCCACTTATGCCAAGCGTATTTA

AAAACATTGTCTTACAGACATCTATTTTAGCGCAAACAGGKTCGGACGTTAYAACAGGAAGAAAATATTTATAAGTAAAC

TTACGATTATTTGGCTCGTCACTATTAATACAGCGTTTTTTTTTAATTTTCAGTGTAAACTGTATTACGTAAGCCCACTG

TTTCTGTTTATCAGCCAATGACCAAAATTTCCTGAAAATCTCTAGGCGTTGATCAGGTGTTATTTTGTCACTGCATTTAT

AACGACATTGGCATGCTGGCCTAATTAACTTCTCATCTACTAATTTTCCTCTTTTCGATATATATGATTTACCCATATTA

GTGCGACATTTCCGTTTAACATCATACCAGTTTTCCACGTTCCGGGCACGTTTTCTAGATTTTCTATTTGTAGTTGAAGG

GTTATCAGCTGAATCTAACCAACATTCATGTTCCGTACAAAATACATCTTGACATTTAGACTTGGAGTGATGGTTTAGAG

GAGATGGCATTGGTGTAGGGATATTCAAACAGTCTACCAATATACTGTTATGATTTACTGGCATCGGGGAATTTTCAAAA

CTTGTTGTGGTTTCTATATTAAATGGAGAYGGATCTATCAAAGTGGGACTGGAAATATCATATATTATCATCGGAGCTGG

TTTTGGGCTTACATTTTCGGGGACTGTATTTARTAAGTGAACAGTGTCATTTACAGCATTTTGATCTTCTTGGATTGTGT

TATTAACGATAACTTCGTCCGTATCATTGAAAACGTCGCGTAAAAGGATGTAAGTTTCTTCGACCCCGTCCGGTAAAGGT

GATGAAAGCTCCCGATCATCTAAAACAAATTGACTAATAAGTCTCCTGTATCATAATAATTTATGCAACCTGTCCACAGG

GCACTGTTATTACCTACTACTTATATAGTTTATRATTCCYAAWGTGAAAAGATCTAGCGCAGATCAATAGTAGAAAAATC

ATATTTTATTTTACATAAATAAATAGTAATTTTGTAAATAAGCCATAATAATGCTCTTAAACAATTCTGGTATAAAAAAA

AATGTAAATATTTAAATACATAAAAAGATTTTATTCAAACAAAGGAACATTTGATTTGACTTCCATCGAACAACGTAACA

TATGCGGATAGTACATTATTTTACTAATGAATCTCCAAAGAACACAGTTACATCTGCACTCATTGTATATAATAATGATG

ATATTTCCGAAGAATAACGTTACATGTTACCTTGAAATACAAAAAAATATAATAATATTAATTTATATAAATTTAAATAT

AATTACTTTTACCATGACAACAACTATTAGTTTTCATATTAAAACAACATTTTAAAACATTGTATAGTTTTCAAAGTAAA

CTGTTTCACGTGCATATGTATCTGTTTTGTTCGGAGGTCACAAAATTTTGCGGTTTAACCTTCTTTAAGGATATTTTTAT

TAACGAACAGTAAACAATCATAATTTCATTACATAATTAATAAAAATAAACACTTACCATTATCTTTTTGGATAAAGTTT

CGTTGTATCTGTTGACTAGAACTTGTATTATCTATACTTTCATCGGGCAAAATTAAAGACAATATTTTCTTAGAACGTTT

CATATTGAATAATAATCTCAAATATCAAARAAATATGTAAATTCAATTAGAAAAACCAATAAACARCATGTAATTTAACC

GTCACCCATGCACAAGCAAACACATGCACACCTCCCGCCAACACGACCGCGAATGACGAAGTAACCAGCGCGCGGGCAGC

GGGAGCGCGGGGTGAGGCCCGCGTACATCGATCTATCTATTCATGAATTGTTATTCTTCGAAACACTACTACGTGTTACG

TTCTTGGCACATTATAACTTCGAAAATAGGCTTCATAAAAAGAAAATAAATACACCATTCGATAGCTCTTGTTATTCTAA

ATTAAATACGAAAAACAAAAAAAATTGATACATTTTTACATGGAACTATGTTCTTCGGATGGGTCG

>Tni_Contig_13_Harbinger *°

AACTTTATTATACGAATCACAATAAAAGTGTATCTCACTATGTGTAATTTACGATTTTAGATTTAATGGATAATACATGG

AACTACTTAATGATTAAAAATAATATTGATTATATGAGTTTTAATAGCTTAGCCACGAGAGGAGCTCGTGGCTAAGCTAT

AACCGCATAAGTGATTCGATCACAGGTTAAGCTATGCTTGACGCGGTTGGTCCGTAGATGGGTGACCATCTTTGTCATAA

CGAGTTCCTCCGTGTTTCGGAAGGCACGTTAAATTGTGGGTCCCGGCTGTTATTCCTACATCTTTGACAGTCGTTACAGG

TAGTCAGAAGCTTGAAAAAGTCTGACAGCCAGCCTAACCAAGGGGTATCGTGTTGCCCAGGTAACTGGGTTGAGGAGGTC

AGATAGGCAGTCGCTCCTTGTAAAACACTGGTACTTAGCTGAAACCGGTTGGACTGGTAGCCGACCCCAACATAGTTGGG

AAAAAGGCTAGGCCAATGATGATGAGTTTTAATTTTATCGACTAATGGGCGTTTATTGGTTATATTGCCATTCTAATCCT

CCGACACTAGAAAGAAAATAATGAGTCAACTCCACTCTTGTATCCTCAGCGGTCACTGTAGTATCCGAGGTCTGATTGTC

TTCTCCTGACATATTATCTAAGCAACTGTCGTTTTCAAGATGTCTACGCCATTCACCGGGAAAGACTTCTCCTTGATTAT

TTTCAGTATCGTATTCGTTTTGCTGAGTTGCAGCGGTTCGTTTGATATAATTAAATAAATGTATACACGCCAAAGTTACA

GTAGTAGCTTTTTCTGGGATTAGCTGAATAGGTCTATGAAAAATCTTGAATTTGTGTGTTAACTGCCCAAACACGTTTTC

AACTACTCTTCGCCCTCGACATACGCGATAGTTAAAAACCCTCTTAGATGATCCCATTGTGTGATTACCATCATAAGCCT

TTATAATATGGGGTTGCAGAGGAAACGCGTCATCACCCAAAAAAAAATACGATACTGGTAGATTCCTTCCGGGTAAAGGG

GTTTTATGTGGTATATTCAATTCATTATCATTTAACAATCGACATAATGCTGAATTATTAAAAACCCCACCATCTGAAAT

CCGACCCTGACATCCCACATTAGCGTACAAAAAGTTGTAATCAGCATCTACTAAAGCAAAGAGCACAATACTGAAATATT

TTTTATAGTTTATATAATCACTTCCACTTTTTGGGGGACATTGAAGTTTTATGTGTTTCCCATCCATTGACCCAAGACAA

TTGGGTATTTGCCATCTTTTTAAATATTCATCTGCAATTTCTCTCCATCCTTCTTGACTCCTTGGAATTTTCACATAATC

TGAAAGTCCTTCAACTAAAGCATCACATACTTCAGGAATAATGCTTGAAATTAGTTGAGGAGATACTTTAAAGACGTCGC

ACAGGCTACTAAAAGAATTTCCTGAGCTTAGGTATTTTAACGTTATTGACAATCTTTCTACTGGTGAAACTGCTTTTCTG

TAATTTGTGTCTTGTTTAGCTATTTTGTGTCCAACAATATTCAGTAAATATTCAAAGTCGGTACTGGTTAATGACAGGAA

AGTCTTAAAAGAAATGGGTTTGTATAGAACCAAGTCGTCTTCCTTTAAATCACTTAATCTTTGACAAGTACTGTATTTAT

TGCGACTCATTAAACTTGGCCTAACCCAACACTTTCGTGTGTTGAGGGATCCACCGATAATAACTCCACAAACTGCTATT

ATTAAATCACTGTTATCGTCCTCCTCCATGTCAATTAGGTACTGAAAATACTACTTCTGAATTATTCGAAACACACACGT

CCAATCTCTACGAGTATTTGGCAATTACTAGAACACCACATGCCGCGGCTGTCGTGCCGCCGGCATTGCCGCCGGCAAAG

TCGTTCGGCTTGGACATTCTGAAGCCGCCGGGTTAACCCGCGGGCACTAAGTCGCCGGGCTGTGCCGCCGGGTCAGCGGC

TTTGGTGTGTAGACCGCTTWATGCCTGCTTAGTCGATACAAATTTTATAATATTTTTGATCATTTTAATTTTTTCAGGCT

CGGTTAAT

>Tni_Contig_24_P *°

AGTCGGAAGAGGTAGAACCAAAGAGAATCTAACCACTTGAGAGCCGATAGGCTTATTTCTTCTAAGAACGCGACTCACAA

CAGCGGGTCGGGGAGTGAAGAAAAATGCGCGCTTGTGATTGGCTAATGCAACAATGTCAGTACAAATATTTCTGCAAAGT

GCCGTTCAATAATTTTTTTCCAAATTAATCGATATCGATAAATCGACTTTATGATTCTTTTAATTCGACTATTAATGTGT

CATACAAATAGTCGTAGAAATACCCTATTTAAATGAAAGGTAATGGTGGTAAAATAAAATAATGTATAAATGTGATTTTG

TTAAAAACTTCATATAATCTATAATTGCCATGTGCAAAATGTACTAGAACGGCCCTTATTATTGAGGTTGTGAAATAGAT

ATGCAACTTGATGTGGTTGTAGGTGGTGGTAGGTAATGTGCACAGTAGGTATGTAAAGTAGTACCATTAGGATAAAAAAT

GATACACCAAACAAAAATAAAATGTTTAATCAACTTTATCCAACCGTATTTTTTTTAATTTTAAAGACACAAACAAACAC

AAACCTTCAAAACTTTCAAAGCCAAAGAGATTTAATTAAGAGTTCTTAAAAAAACACTCACAATTCTGACGCTCTCAAAA

TGATATTATAGTAAAGGTGGTGGTAACGAAAAACTAGAATTTAAACAAACCGAAATAAGTTATTACTGATTTTTAAAATG

AATAAGTTTAGTGTAAGTTTTGCGTATAAAAGCTTTAGGGTCCTGATTGTGCTTAGTTAAAAATGACAATCGAGCGTCTA

CGTACTTTTGGATTATGCATTTTGTTAAATCAATCAAGTGATTAGAGCTTTCTACTTCAAATTGATGAAATAATATTTCC

GGGAATAATTTTTTCCCGATGAACGATTTCATAACTAATCGGATGAAGTAATCTGTATTACATTTTGAGGGCAGTACTTT

GGATCCGTTTTCTTTGATCGATCTCTTTATGTACATTTCACAACGGCGACAAATTTCTATAACGGTGGTGGAACCATAAA

GTAACTTGTTCTTATTTAGATCTTTTTGGTACTGTAAGGAGTTAACATTTTCTAAGCCAATCAAAGCTCCAATACACGTT

TGACATTTTAATTTCTTTATCAATGCATGGACGGTGTAGCCGGCCATGTATTCAAGAACCCTTTCACCAAATTCTGAAAC

GTGTGTAAGATCTTCAAGTTCAGGAAAATCTACATTTTCGCTTTCAATTGACTTTTCATGTGTGTCTGTAAGGTCTCTGT

ATCGTGTTGTGACATTGATACGATCCACTGGTGATGTACATTGCAAAATAGACATTTGATGTAAAGCAGAGCAATTTCCA

CTGCCGCTATTTCTTATTTGTGCATAGGTGAGAAGCTTTTTATAAATACTCTCGAACTGTCGAGCGCTGGGATTATTGTT

TGAACCCCCATGACATCTTACACTTCCAAAAAAATGCTCCAAATGATCCTGGCTGATTTTGTGGAGTGGTAAGTATTTTA

ATGCGTAGTTTGGATTTACAACGAGGTCCTGGATAAGGTATATCGCACTTTCGATACATACACAAAATCCTAGAAATCCA

GTATATCTTGGTGATTTTATGAGTAGAACAGTTTGATCAGTATTTTTTATTTTTATCTGAGTAGACAAGTTTAATAAAGT

ATCTTTTGAAGCCCTAAGTTTTACTAGCACATTGGTTGCATTTTCAACATTTACCGCCCTTTTCAGGCCGTAACCGTGGG

TATGCGAGTCTAGGATATCAAATAGGTCATTTATCAAGTCCAAAAAACGTGCTGTCGGTGTAACCTGCTCAAATTCCCGT

AAATTGAGTTGTTCCTTGCAAAACATGAGTGAATCTGACACACTCTTGCTAAAAATTTGGGTAGCCAAGCTCACTTTCAT

TATTTTACTTTTAAAAAATATGTGAGCTGCTCTGATGCGATTAGCAAGATGAAAATTTTCTGCTTCTTGCAAATTATGTA

AATTTACAAGGTGTTGCCAGGAAATGGTCTCACCATGTTTGTCTTGAAAATTTTTATAGCACTCGAACGCATTTCTTATT

AGCTTTATCATGTGCGCCGGATCTAGAAATACTGCAATTTTATCATCATTGCTGGGGTTTTTAAAGGTAGTATTGAAATT

ACTACCTTCAAAAGAACAGCCTAACTTTTTGGCCATCGTCAAATTCGCTGGACAACCGTCAAATGTGACAGCTTTAATTC

CAACACCAGCTTCTTCTACTAAATGAATGGCTTCCGTTAAAAGAGCCACTTTCTGCTCAGAGTTAACGCCATTTATTAAA

AAATATCCAATTGGAATTTTCCAGGAAGCGTTGATCGGTACCAACAAAAATACTAGTGCTTCCTTGGCTTCATCTTGGCA

ATCTGTTTCCAAGCTGGTACCGAAACTAACGTGGCCATAATACCTCCGGCCTTGGGGGTGATATTCGACTCCGCTTCTTA

TAGCCATTTCATCAAATATAATAGTAGCGTAAAGTTTTTGTTTTGATTCTGTCGCTTTGATTTTAATGGCATCAATCGCC

TCTTTCGTAAACCCTGGTTTACCATCAACCGACTGATACCACTTTCTGAGCGTGCTCGTGGCTGGTAAACATGTACTAAA

TGTTTTTCTTACAAAATTATATGCCTTTGGCGAATAGAAATGCAGTGTCAAAGCGAACGCTCGTAATTCTGGAGAGTATT

TTAGTTTTGGCATATGTCCTGTTTTAGATTTAAGTATTTGACGTTTAACTAGTTCTCGATTTTGTACACCTATGCAGGAT

AAAATATTTTCATTTTCTTCGGTTATATAAGATTTTTGAGACAATGTATTTAAAATTTCTTTTAGATTCGCATTTTTTTT

TAACAACACTCTATTTTTGTCTTGAAGCCTCTTTACATGTTTTTTCTTGTTATTTAGAGAATATATTAGATGGTCTATTT

GTCGTTTCATTTTGCGTTTTCGTGGACTGAGCTCACCAATTTCTGGGTCTACTATTACTTTGGTATTCATTGTTGAACTC

TGAAAATATAATAAGAAATATTAATAGTGGACTTAAAAGAACTACTTAGTTATAAAGTAATATGTGAAATATTATAAAAT

TAAGCTTACGGGACTTTCATAAACATGGGAAGGCCTAAGAATGTGACTATCATCTAGTAGACCGCACGGCACAAGAGTCT

GGTCTTCAATTATTGTATTAACTTCAGAATGCACTTCTGTTTTTGATGATAAACTCTAAAATTGAAAATATCGGGTACAA

CAACCGCAGTTACATTAAATTTAATCCACTGACCTATCATAAAAAAATCAACTTACTTGGCCGGTATTGTGATGCGAAGG

CCCAGGTACGTTAGTATTAGACTCAGTCAAACTATAAGGTGAAGCCTGTAATAAAATAATGTTTTATATTTATGTTTTGA

CTTTTCTTAGCATTTTACCAATAAAACAAATCGTACCAAAATTGGTAGAAACATTGTTGGTATTGCTGAACTAAATAAAC

GGCGACGTTGTTTTAAATACTCAAAACAATCAGGTGTGAAGTGCCTTGAACAGACAGCACTGTATTTCGTTGGGAACCAA

TTTTCCCTATTAATTTTATTCATCCACTTTTCTTTTAGTTCCGGAATCTTCGGAAACCTAAAATGAAAACATGTACTTAA

TTAATTGAGCAAAATAGTAATGAAATTCTTCATATAATTTTTCTTAAATTTTATTTTGTGATTATCGATATTTGTTTGAA

TTGACTTCAGCTAAAAGTAACAGCACTATTTTTTACGATAAACCTACAAATTGGAAGAAAGAGATACCCGCATTAGAATT

GACTAATGAAAGAAAGAGACTTTTCCAAAAGACATAAACGCGCGTTTCTGCACCTTTGCATCTGTCACCCGCCATCCATT

TTATCTTGACACATACAGATCTCGTATAACCTACAAAAATAAATAAAATAATCGTTCTGCTTACTTACCGGTGAAAAGTT

ATACCATCCTTTTTGTAACTCGATGTCTTGGAATGTTTCCCACACCACTTCACCGCACAAGAAGGCATTTTAATGCGTTT

TAATTGCAAAATAATTTTAACAATTTACGATAATTTAGCGTGCGCACAGGTCTAACACTTGGACACAAACTAAAATGGTC

ACTCATACAGATAGCTTATATAGGCGACAAAATACTAATAGTTTTCGAACGTCAAAGCACTGGATGCGCCGTCAAGAGGT

TTTATAGTCTTTGCTTTGTA

>Tni_Contig_27_Sola

CCCTTTGTCTAGTAGAGGCATTATATTTGCCGACATGTTTTCATTGCTCCACAAAAAGAAGATTTGTATTAAAGAAACGT

TTTATCGTTTCGGGTAATAGAGAACAAACTAGTACGACGAAAAAAAATATAAAAATTGGTAACATTTATATGACATGTGT

CAAATAAATGTCAAGTAAGTTTGAATTTACAGCTTTTTCTGTTCTCAAGAGTAATCCGGCAAAATCTCGAAAAAAAACTG

TTATTTTGGTAAGTTTTCTTTTATAATCGTAAGATTTGTGTTTTTATGACAATCTTAGATAATTATTTTAGATCAATATG

TAATATTATTGGCGAAATCTGGAAAAATCACGTCGGCAGTATATTTGCCACTACCAGTTAGTACATTACCCGTTGTTGGC

AATATATATGCCAGTACCAGTGAGATTTATTTTTATTTACAGAATAAACAATGGAGCACCGTCGGTCGATAAACGACCGC

GAAATTGAACAGCAGTTGGAAGCCATGTTTGGTCTTCCAGATGGTACAGATTCAGAAGACAGTTTGGAAGAATCCGATAC

TGATGACCTTGTACAAATGCTTTCGGCTAACGATTCCTCTTTTCAACCTTGCATCGAACCAGACTTTGCAGACTCGTTGT

TGACGGCTAGAAGTAGTAGAAGACACGGAAAACATGGGCCAGAACAGGCAACGGATGAATGGGAAGAAGTTGACGACGTT

GAGAGTGACGACGAAATAAGTACAGGAAGCAAAAACAGAAGAATGGAGAATGGTAATATACGGACTGATAGTGAAGACCA

AATAGATACAGAAAGCAACATCATTCGGAATGAGACAAGTGAAGGGACAACAGATTGTGAAAGTGATAATGAAGTGGGAG

GAGGAGGAAACGTTAGAGTACTCCGTGCTTCAACATCTAGCGCCACCACTGCACGGCCCTTAGATTCTACATCTAATGTG

GTGACACGAGATGCAGAGGTGACTGCACCCACTTCCTTAGTTTCAGAAGAATCAGATTCTGATGAAGATGAAATAGAATG

GAAGAAAGTTGATTGGCTAAATGATCCGAATGTGCCTTCCTTCGACGAAAACGAACTTCAATCGCAGAACCATTTTCCGA

GTAGATCTAGACCAATCGCTTACTTCGAAGTATTTTTTGATAATGAAGTGATTGAAAACCTACTTGTTCAATCAAACTTG

TTTGCTAGTCAATCCAATGTTAGAAATTTTACTCAAATTAGTAAAGACGAACTCAAAGCATATCTTGGAATGCTAATACA

AATGGGGATACACAAACTACCGTCAATTGAAGATTACTGGTCTAGCAATCCAGCATTATGTGTCCCCGAGATTGCTGAAA

CCATGACACTCCAAAGATTTCAAAAGATTTCTAGATGTCTACATGTAAATGACAACGAACAGATGCMCAGAAGAGGTGAA

ACAGGTTTCGACAAACTTTATAAAATTCGCCCACTACTAGATCAAATAAACCAGAGATGTCAAAATAATGCCAGAAATAC

AAAATCTCAGTCCATTGATGAATCAATGGTGAAATTCAAGGGACGCAGTGCGCTAAAACAGTATATGCCCCTAAAACCTA

TCAAGCGCGGTTATAAAATTTGGGCTAGAGCAGATAGCAAGACTGGTTATTTGTTTCACTTCCAAGTCTATACTGGTAAA

AGTGACAATGTGGAAACTGGTCTAGGAAGTAGTGTGGTAAAAACTTTAGCCCAACCACTTATTGATGAAGGTTGCTCAGC

ACATATTAGCTTCGACAACTTTTTTAGTAGCTATGACCTTCTTCAGTATTTATATGACCACGGCATATACAGTACAGCAA

CTGCGARAAATGATAGATTGGGAATGCCTGTTTTAGTAAAGAAACCTACAGGTCTAAGAAATTGCGAMGAAATCATGAAG

AGACAAAATAGAAAACTGAAACAGTTACAAAAAGGGCAGTACAAATGGCGAGTTCGGAACAACGTAGGGTTTTCAATCTG

GAAAGACACAAAATTAGTGACCATTTTGAGTACAGCCTTTCATCCAAAAGAAGAGGCTACATGTCAACGTACACAAAAGG

ATGGAAACAAAAGMCCCTTTTCCTGTCCACGTGCAGTTGTAGAGTATTCGAAGCGAATGGGCGGGGTCGATAGATTCGAT

CAGCAAAAAGCCATTTATGATGTCGCTCGTCGAAGCAAGAAATGGTGGAAGCGGTTGTTTTATTTTTTGTTCGATGTAGC

AATCACAAATGCGTATATATTGTACTCAAAGAATTCAAGAGTGCACAACCCCATGAGCCAAAAGATGTTCAGGCTTACTC

TTGCCAGAGAACTGGTCAACAATTTGACATTTCGGAAACGAAAATTTTCATCTGCTCCCAAATTCTTAGCAAAGAAAAGG

AAGAATAGTGCAGAGCCAGTTACTCGCCAGAAGAAACTTTTTGGTGTTCCGGAAGATATTCGTTTCACTAATGTTGGTGA

GCACTTACCAGAAGGCATAGAAACGTACAGGCGCTGCCGAGTATGCAGCAGCGCAACTAAGAATAAAAGATCAAACATAC

AATGCGGCAAATGTGAAGTGCCACTTTGTGCTGTTCCTTGTTTTAAACAATTTCACATGTGTGCTGACCAGTAGTGGCAA

TTATATGGCCAACATATAAAAAAAATAAAAAATAAACAAAAAAATAAAATATTCTTCTTTATAATACATAATTACATAAC

ATATCTGAGTTTCATTACATTTGCACGGCTAGTTTCCTCTACCAGTTAAAGGG

>Tni_Contig_25_Transib

CACACTGGGGCAAGCTGTATGAAGGCGATGCCCAAAATTGAATTTCTAAAATGAGGGTATTTCAAGAAACTTATGACAAA

ACATCAAAGTTCGATATATTTCGCATCACATAGCATTGATATTATCCATCCAAAGGTTATCAGTTCGCTAATATATTGCC

ACAAAGTTGATAGAATTGATAGTTGTTGCATTTGCACTATTTCGTCTGCGTGATTCAAGTGTACATAACTTCACTGTTAT

TGAAGTTATCCAAAAACTAAAATATGTCCAGTGAAGATCAAGGTCAGTACTATTATAAATAAAAGTCAAATAGGGCAAAA

GTATTATATAATATTAGTAAATAACAATATATTTATTTTATTGTATTTTATAATTAAAGTAATGATATTTTAATTTTGCG

CGAAGTTCTTTTGAAATGAATTAGTGTTATGTATAATACAAACCTTCATTTTTAAGAAACCAAACGCAATAGTGAAAGAA

CTTTGCGCAAATTCAAAATATTGCTTTCAATGCTATAACAGGCGCATAAGTATTTTTTTAATTATTTTCAGATATGTCGA

AAAACGTCATTATCAGTAGAAAAACTTTAGTAGAAGTATTTTTTAAGAGTAAATATAAATCATTTGATAGTAAATTTAAA

GAAATAATAGATTTTGTTGCTTCCCAAACGAATTGTCCTACGCAATCTCGTACAGATTTAACTTTACAACTGCGTAACTT

CAAGCGACAATTTAACACGAAATGGTTACTACACGGTAAACATAGAGAAAGACTATTCGCTAATGAAAAGACTTGGTTGG

ATGGAAGTTGTAAATTTACACGTTTTGTAGTCATAAACTCAGGAAGGCCCACTTCAAATTTTAGTGACTCTAGCGAAAAT

TCAAAACGCAGAAAAACATCTAGTCTGCGTGCTGAGATGGATGCAGATTTTTTAACTTATGCAGCACAAATGAAGCTAAG

GTCCTCAGGTAGAACCACAGAAGCACAAGTTATTAAAAAAATGTCAGTATGTCCCAAATACGCAAAAGACTGTTTTGACA

TATCTACCGAAATAAAGGTCAAGAAAATATCACCGCGGGAAGCACTTTCATTACTAATTGAGGCTCAACTATCTCGTAAA

CAATATGAAATTATCAGAAACTACGCTAAAGATATTTTTCCATCTTACAAGTTGGTGCAGGCAGAAAAGGCTCTTTGCTA

TCCGAAAGACGTTCAGGTTACAGAAACTGAAGCTGTCGTGCCCCTTCAGTCGTTGCTTGATCACACTGCCACTCGTCTGA

TACATTCACAAAAAAGTGTTCTTCTTACGCAGTCAATAAGCTCTTCGGATAAAATTGTATTGCATACGAAATGGGGTTTT

GACGGAAGCTCCAGTCATACCCAATACAAACAAAAGTTTATTTCGGAGACTGCTGACGACAAATATATGTTTTTAAGTAG

TCTCGTTCCATTAAGACTTTCGTATGAGAAAAATGATAGCACAGTTGTTTTGTGGCAAAATCCAAAGCCTTCTTCATCAC

GATTTTGTCGGCCGATAAGTCTCCAGTATCGTAAAGAAACTGATGAATTAGTCAAAGCAAAAAAACAAGACATTGATCAA

CAAG

>Tni_Contig_16_piggyBac *°

CCTTTTAACCGCCTACGTCGGATTAATCCGACAAATATTTTCGGGCCCATTTCGCCAGAGTCGTATATTTACGACCAAAA

TATCACTGCTCGTTTAATCAGCTTTTTTATTTAATTACGCTGTATTCTATCAAAAATATGTTTATATTAGGCAAATAGTT

AACTAAGTAATCATTTACAGTTTAATACACTTTAGTCTCTTAATTAGAAGTCCTAAAAATAATGTCCAAAGTTAGATGAA

TTACTAACATTATTTTTAGAAAATTGTATTGATGTGCAACGCCCATGACGACAAAATCCGACATGAGGGACGGGACATAT

GCCAAGATTTACCGATACAAAAATAAACATAAATCAAAATATTTCTCGTTATACTGACTAATTAACTTTAAAACAAATAC

AGCAATTGTTAATAAAACATTTGTTTACAAATATTTGTGAGTATTTCATTTAATTCCTTTAAAATTAAGTAAATTTACAT

ATGAAATGTCAGCGATTCAATTGATGGTTCCCATCCCTAGCCTACATGTTCCGAAACTTACTTCGTTAGTACGCCCTGCG

CTACGGTCGGATAAAGACGACCGCTTCTTACGTCATAAACGAGTGCCGACTTCTGGAATTTTCCATCAGTGTTGTGCTTG

TGCATGAAGTTTTACGTTCGTATGTCGATAAATGTACATGATAGAAATGTATCATGACGACTACTGAAGAGTTAATACGA

ATTCTAGAAGGCGATGAAAATGAAGAATTTTTATCAGACGAAGAGTTTGACATTGATGTAGAGGTAATTATTTTTTTTAT

TACATATCTAGATAAATGTATTTTAATTTATTGTGTAGTATGGTGGTGATGTCTGAATGGAAATTTTAAACTTAGAGTGA

GTGAGATGGCGAGAGCGCGCCGTGCGATGAGAATGCGCGCGCGGCCACTCTGGAACGGTGATTCTGATTGGTTGGAGCGC

GAGTCACGTGTGGGCGCACTGACTCGCTCGTAGATACATAGGTAGAGTTTATTTTGTGCGTGCTGCCGCGCTGCCACACA

CCGCACTGAATCGCACTGCTAATTTTGCTTTTGTAGGCTATTCAAATTTCCTACCTTGGAGACCGATGTATTTTTATTAC

TCTACAATTATTTTTTGTATTATTTCAGATTCAAGGAAACAATGTCACAACCACTAATTGGACAACGCAAATTGCTACCC

CAATATTAGAAGAATTCGACGACTTTGTTTGTGGACTGTCACCTGCTGTCAAATTAGATGATACCTCCACTGAATTCGAA

TTTTTCGAACATTTTTGTAGCAAAGACTTGTTACAGAAAATAGCCAATTGTACGAATAAATATCATCAACACTTCGTAAG

ACATACTGATCTCAGAATATATTCAAGACTGCAACAATGGCGCGACGTGACTATTGACGACCTTTACATTTTCCTAGCAG

TAACAATGCTTTTCACTAGAAACAAAAGAATAACAATTGAAGAACACTGGTCTACAGACCCGTTATTACATAGTACGGTG

TTCAGTAATACTATGTTTCGAAATAGATATTGCTCAATTCTTGCAATGTTATCTTTTTGTGAAGCACCTGCAGGGACAAG

TAGTGTACCGAGATTGCACAAAATAAAAATGATGATTGACCACGCTCGTGAAACTTTCAAAACAACATTTATCCCGGGTA

AAAAAATGTGCATTGATGAAAGTATTGTGCCTTTTAAAGGTCGTCTAATTATAAAACAATATTTACCTAAAAAACGTAAT

AGATTTGGCATTAAGTTATTTGTTTTATGTGATGTCGAAACCGGTTATATCGTGGATTTTATAGTGTACTGTGGATCGGA

AACAGAAATAGAGACCGTACCAAATTTAGGCTTATCTGGATCAGTTGTGACTGAACTTTTGAAAGACTACTATTTTTGCA

ACCGAGAGTTGTATGTAGATAACTGGTATTCCAGTCCTCAGCTTTTTATATATTTAAAAGAAAGGGCGACTTATGCATGT

GGTACCGTAAGATCAAATAGGAAAGGGATGCCGAAATTCCAAAAACTGAAACGTGGACAAATAGCCGCTTACTCGTCTCC

TCCCCTCTTAGCCCTAAAATGGCAAGATAAGAAACCGGTCTTAATGTTGTCTACAATGCACGATGACAAAATGATACCAT

CAGACAATGTAGACTACTCAACTGGGCTACCCAAAATGAAACCACAGTGTGTCGTAGACTACACAAAAAATATGGGGTCG

GTTGACACTTCTGATATGATGACTAGCTCATTGGGCTGTATAAGAAAATCAAAAAAATGGCATAAAAAGCTTGGGTTTCA

CATATTTGACATGTTTTTACTTAATGCATTCTATTTGATTAAAGTGATTAAAAATAAACAAAATTATAGTTTAGCTGATT

TCCAATTAAACGTTATCAGACAAATRATAGAAAAATACAAACAATGTTCTTTAACTCAACAGTCAACTTCGCGCGGCGTC

CTTGACCCTAAAGGTTTTTTACATAATAGTGCCTTAGAGCATTTTCCTGTTAGAATACTAACCGGCAAAAGTCAGCGATG

CAAAATGTGCGCAGCAAAAAGAAAACGGTCTGAGACTCGTTTTATGTGCCAAAAATGCAAAGTTTATCTCTGCATAGATC

CATGTGCTGTTGAGTACCACAATTCTACAAAACAATCGGTATCGTAATCCCACATCACTATTTTTGCGTTTAGTCTATTA

TGTCTATGGTCAGCTAGACGTTGTCGATACTCGACAATATCCATTATTCCGTATTCGACAGTGACGACTGTTAGTGGCAC

GACGTTTGTAAAATCAAGTGAACATTTTACAATTTTTTAATATCAAGATGGGGTCCAAAAGTAAGTGGTCTTCGAAACGT

ATTGCATGTAAATGGTGATATCATTACAAAAACTCATGTTTGATTTGTTTTATAGGGAAGTTTGAAGAGAGCCAGTCAAT

TTTGGCTCCCTTTGACTATAGCCAAGATAAAGTAGACATAGACTGTAGCCAAGATGTTAACCTTCCATCAACGCTGACCA

ACGACGAGTATATGGTTTATTTAAATGAGGGTCAGTCGCATTTAAGAAGTTCTGATAGTACGCCAGATTTATATAGATCT

ACGGCGACACAAACTTTAGTCAAAACAACAAATGCAAGTACACAATACGAGCAAATTGATAATCCAATCAAAGAAAGTGC

CAAAATTTTAGCGGACAGTTATTTTGTTGGCGATGAAACTCGTAAAAATAAGTTTATCGAATGTTACACAAATGTGGTCT

CGTCAATAATTCGCCTCGTCGATAGCGAGCATAAATAATTTTTCGACGGATCATACTATTATTGAAAAATCTTACCTCTG

ATTGTTAGACTGATTTTAGTAATAAGAAATTATATCTGTGATTATTTGACTGATTTTATTGAAATTAGTGTTATACAAAT

TTTATTGTGATTGTGATA

>Tni_Contig_9_Mariner *°

ATGTACAGTCTGGGGCCATAAGTCCAGTAGCACATCAAAGATCGTATTCGCCTGCATGCTACGGCGCATACATGTGTGCG

TGATGTCAACGCAAACATTCCCCGTCATACAGTATAACTACAAGATGATAGTGTGTGTGGGTAATACTAGACAAAGATAA

CAATATATTTGTTTTCTTTCCTATTAGTGTGGTATGCACCTTTATCAACAAGTTAATTTTCAAATCTACCTGCAACATAT

TCACAATTTTATTATTTAACTAAAACTACCTCATAATTTCTGCGGTCTTTTAATGGACCAACTATTATGTCAATTTAAAC

TTCAAGAGTTCATATGGTTAAGTGCAACAATGTGTGACTTGGGAATGAGTGGATGTCAGTTCGAATTTCAATTGATTCGT

CAGATCGCCCTTTTTTACTTTACTCTCTTATTAGAAGCAACATTATTGTATATTTATTTATAGATTTTATATTATAAATA

TATAAAGGAATATTATATTATATTTAAATATATCAATGATTATATTATAATTAATAATAACTTTAGGTTCAAATAAAATA

ATATTTGGTAATGTGAAGAAACATTTATTTATATTATTGTTAATAAAGTTAAAAACATAATTTATCATTTTGTATTATAT

TAAAATTAAAATAGAAGTGTTATTATACTTCTGACTTTATGAGATTATAGCTTAATATATTCATTTTCTCAAAGAGAAAA

GAAGATTGAAGCTTAATCCCAATCTTAAAATCAAAATAAATTACGTATGACTTTATATTATGACCAGCACGCCCACTAAA

AAACAGCGTAATCAATATTTAGTATGGCCTCCATTATTTTCAATGCAGCTAAGCAGACGTCGCCGCATGGAGGCCACAAT

GTTTGCACATATATCTGTGCCGCGTAAATTCTCCCACAGTTGAGTGCAATGGGATTTAAGCTGCTCAGGACATCTCTCAT

CTCGCGCTTCCCATCTTTGAACCATTAAACCCCATATATTTTCAATGGGGTTTAGATCAGGGGAACGTGCTGGCCACGGA

ATAACTTTAATATCATTATGCTTGCTAAACCACTCTTTTACAATACTGGCACGATGTATCGGACAATTATCTTGAACAAA

ATTTAGTTCACTACATTCTTCTATGGGGTAAACATTACGCACAGTAGGTAGCATTATGTCATTTAAAACTTCAACGTAAT

TAGAGCTGTTCGCACGCGTAGGTATGAATTCCAGTTCACCCGGCCCTTGAGCACTAATCCACCCCCACATGTTCACACTT

ATTCTACCGGATTCTGTATTCGGAATAATGTTTTGCTCAGACCAGCGAGTATTATCTTTTCGCCATAAATGAAGTCTTCC

TTTCTGGCAAGATTTGAAAGTCTTTTCATCGGTAAATATGGTTGTATCCCAGTTAAAATCTAAATATTCTCGAGCAAAAC

GTAGTCGTTCCTCCTTGTGTTTTTGTGTCAAAAACGGTTTTTTAGCTGGTTTCCGGTGATGTAAACCTTCAGCATGGAGT

GCACGGCGAACAGTTCGTACTGACGTATCAAATTGATCAGCAAATATTTTTGTTGGAATGAAACCATTTGTTTCATATTC

TTCCACCATGGCACGTCGCTGAGTCGGGTTTATAAGCGGTGGCCTTCCACTTCTTACACGACACTGCAGACTACCTTCTT

CCTCGAACCTCTTTATCCAACGGCTAACAGTATTTCTCTGAAAAACGAAAATTTTATTATAAACTTGCAACTAGTGATTA

TATAAAAAAATATAGACCACAACAACCATAATTACAATTTTAAAAAAATACTTACACATAAGCCGGTTTGATTTGAAATT

TGAGAAATATTCAACCCATTTTGATGTAAAGCGACAATATTTGCCCTGGCATCAATCGTAATATTTGCTTGCATTTTGAA

TAAAGATAATAAAAATACACTAATCGCAATGATAATACAAACGCTCCGAACAAAGTTCACCAACAAACAACGTCGTAACT

AATTTATTAAATTAAAATCTAGTAGTTAGTTAATTATTTTTTTTACATTTACCCTCATCTTCATTCTTTTTTGTAAATAA

GTTCGCGACACATTTCATCGCATATAACTAACTATAGAATTAGTGTAGCCATTCTTAATTCATAATTAAAACACAAAAAG

TGTATACTGCAGTGGGGTTCGATTGCAGGTCTCAATGAGCGTTTCAGTTAGACCAATGCGTTATCCGCTAAGCCAAACAG

ACACTTATCTACATTATTGAAATTAAATAACAATATCTCAATACGTCCGTCATCGCATAACTGGTTTGTCACCAAGGCAA

ATTATTATATCGCACTCTTTCTGACTAGATAATGATGAAAGAGTGCGAGACCGCGTATGTGTGCAAAATGTTTGTTATAG

AAAAAGGTCGTAAGTGCAACTTGTTTTAATGCGATAGTTTTGTGAAACTGCTACTGCACTTATGACCGCTACC

>Tni_Contig_28_piggyBac

CTTTAACTTATTTTCTGGGGCATTATGCAGGTAATCATTTATTGACTTATTAAGATGAGCAGTTTCTGGAAAAGTGGTTA

CTGGAAAAGTTGCAGAAGTCGACGGCTGGCACAAATCTGAACAACTTTTTGTAGGAAACGTTGTTATTTTTAGTGTGGTT

TTATTAGCATAGTGTTTTTCAGTATCGGTTGATTCCTTCTGTGGGCAATCACTTTGAAAGAAGGACCAAAGTTTAACCCT

AGAAAGATAATCATATTGTGACGTACGTTAAAGATAATCATGCGTAAAATTGACGCATGTGTTTTATCGGTCTGTATATC

GAGGTTTATTTATTAATTTGAATAGATATTAAGTTTTATTATATTTACACTTACATACTAATAATAAATTCAACAAACAA

TTTATTTATGTTTATTTATTTATTAAAAAAAAACAAAAACTCAAAATTTCTTCTATAAAGTAACAAAACTTTTAAACATT

CTCTCTTTTACAAAAATAAACTTATTTTGTACTTTAAAAACAGTCATGTTGTATTATAAAATAAGTAATTAGCTTAACTT

ATACATAATAGAAACAAATTATACTTATTAGTCAGTCAGAAACAACTTTGGCACATATCAATATTATGCTCTCGACAAAT

AACTTTTTTGCATTTTTTGCACGATGCATTTGCCTTTCGCCTTATTTTAGAGGGGCAGTAAGTACAGTAAGTACGTTTTT

TCATTACTGGCTCTTCAGTACTGTCATCTGATGTACCAGGCACTTCATTTGGCAAAATATTAGAGATATTATCGCGCAAA

TATCTCTTCAAAGTAGGAGCTTCTAAACGCTTACGCATAAACGATGACGTCAGGCTCATGTAAAGGTTTCTCATAAATTT

TTTGCGACTTTGAACCTTTTCTCCCTTGCTACTGACATTATGGCTGTATATAATAAAAGAATTTATGCAGGCAATGTTTA

TCATTCCGTACAATAATGCCATAGGCCACCTATTCGTCTTCCTACTGCAGGTCATCACAGAACACATTTGGTCTAGCGTG

TCCACTCCGCCTTTAGTTTGATTATAATACATAACCATTTGCGGTTTACCGGTACTTTCGTTGATAGAAGCATCCTCATC

ACAAGATGATAATAAGTATACCATCTTAGCTGGCTTCGGTTTATATGAGACGAGAGTAAGGGGTCCGTCAAAACAAAACA

TCGATGTTCCCACTGGCCTGGAGCGACTGTTTTTCAGTACTTCCGGTATCTCGCGTTTGTTTGATCGCACGGTTCCCACA

ATGGTTAACTTATACGGTTCTTGTAGTAAGTTTTTTGCCAAAGGGATTGAGGTGAACCAATTGTCACACGTAATATTACG

ACAACTACCGTGCACAGGCTTTGATAACTCCTTCACGTAGTATTCACCGAGTGGTACTCCGTTGGTCTGTGTTCCTCTTC

CCAAATAAGGCATTCCATTTATCATATACTTCGTACCACTGTCACACATCATGAGGATTTTTATTCCATACTTACTTGGC

TTGTTTGGGATATACATCCTAAACGGACACCGTCCTCTAAAACCAAGTAACTGTTCATCTATGGTCAAATGAGCCCCTGG

AGTGTAATTTTGTATGCACTGATGGATAAAGAGATCCCATATTTTTCTAACAGGAGTAAATACATCGTTTTCTCGAAGTG

TGGGCCGTATACTTTTGTCATCCATTCTAAGACATCGTATCAAAAAATCAAAACGATCACGACTCATTACAGAGACGTAC

ACCATTGACAAAGATCGATCAAAGAGGTCATCTGTGGACATGTGGTTATCTTTTCTCACTGCTGTCATTACCAGAATACC

AAAGAAAGCATAGATTTCATCTTCATTCGTGTCACGAAATGTAGCACCTGTCATAGATTCCCGACGTTTCAATGATATCT

CAGCATTTGTCCATTTTACAATTTCCGAAATTATCTCATCAGTAAAAAATAGTTTGAAGCATAAAAGTGGGTCATATATA

TTGCGGCACATACGCGTCGGACCTCTTTGAGATCTGACAATGTTCAGTGCAGAGACTCGGCTACGCCTCGTGGACTTTGA

AGTTGACCAACAATGTTTATTCTTACCTCTAATAGTCCTCTGTGGCAAGGTCAAGATTCTGTTAGAAGCCAATGAAGAAC

CTGGTTGTTCAATAACATTTTGTTCGTCTAATATTTCACTACCGCTTGACGTTGGCTGCACTTCATGTACCTCATCTATA

AACGCTTCTTCTGTATCGCTCTGGACGTCATCTTCACTTACGTGATCTGATATTTCACTGTCAGAATCCTCACCAACAAG

CTCGTCATCGCTTTGCAGAAGAGCAGAGAGGATATGCTCATCGTCTAAAGAACTACCCATTTTATTATATATTAGTCACG

ATATCTATAACAAGAAAATATATATATAATAAGTTATCACGTAAGTAGAACATGAAATAACAATATAATTATCGTATGAG

TTAAATCTTAAAAGTCACGTAAAAGATAATCATGCGTCATTTTGACTCACGCGGTCGTTATAGTTCAAAATCAGTGACAC

TTACCGCATTGACAAGCACGCCTCACGGGAGCTCCAAGCGGCGACTGAGATGTCCTAAATGCACAGCGACGGATTCGCGC

TATTTAGAAAGAGAGAGCAATATTTCAAGAATGCATGCGTCAATTTTACGCAGACTATCTTTCTAGGG

>Tni_Contig_8_Mariner *°

ATCCCGGGTAAAAAAATGTGCATTGATGAAAGTATTGTGCCTTTTAAAGGTCGTCTAATTATAAAACAATACTTAGTCTG

GCCATAAATACTGTTACACTTAATTATAAAAAAATATTACATTTGAATTTCGAATCTGTCATTTTTATACGATTGTTCAT

TGTGTTTTCTCATTTTGGCGCCAATACATTGTAAAATATTTTGCGATATTAAAATGGTGTGGGGTGATAAAGAGAACCGA

ATCGCTGTGATAGCATTACACAAAGTAGGTATGGAGCCAAATGCAATTTTTAAAACTCTCCATACACTTGGTATTAGTAA

AATGTTTGTGTACCGGGCTATTAATAGGTACAATGAGACCTCCTCTGTTTGTGACAGAAAAAGATCTGGCCGTCCACGTA

GTGTTCGTACGAAAAAGGTGGTCAAAGCAGTAAGGGAAAGAATTCGAAGAAATCCTGTCCGAAAGCAAAAGATTTTATCT

CGGGAAATGAAGATAGCACCTAGAACCATGTCGCGTATTTTAAAAGATGACTTAGGACTTGCAGCCTATAAGAGACGCAC

TGGCCATTTCTTAACTGATAATTTAAAGAAGAATAGGGTGGTAAAATCGAAACAACTACTGAAGCGGTACGCAAAGGGAG

GTCACAGAAAAATTTTGTTTACGGATGAGAAAATTTTTACAATTGAGCAACATTTTAACAAACAAAATGACCGTATTTAT

GCTCAAAGCTCTAAGGAAGCTTCCCAATTAGTCGACAGAGTGCAACGTGGACATTATCCGACTTCAGTGATGGTTTGGTG

GGGTGTTAGCTATGAAGGAGTGACTGAGCCATATTTTTGTGAAAAAGGTATCAAAACATCGGCACAAGTGTATCAAGATA

CCATTCTTGAGAAGGTAGTTAAGCCCCTTAACATCACCATGTTCAATAACCAAGTATGGTCCTTCCAGCAAGACTCGGCG

CCGGGTCATAAAGCTCGGTCCACGCAGTCTTGGTTGGAATCGAACGTTTCGGACTTCATCAGAGCTGAAGACTGGCCGTC

GTCTAGTCCCGATCTTAATCCGCTGGATTATGATTTGTGGTCAGTTTTAGAGAGTACAGCTTGCTCTAAACGCCATGATA

ATTTGGAGTCCCTAAAACAATCTATACGATTGGCAGTGAAGAATTTTCCCATGGAAAGAGTGCGTGCTTCTATTGATAAC

TGGCCTCATCGTTTAAAGGACTGTATTGCAGCCAATGGAGACCACTTCGAATAAGCTTTTTATATTTTTAATTGTTTTAT

ATTTATGTATTAAACTGACACACTGTAAAAGTAATAAATGTTATTTGCAGTTAACAATTTTCTTTTTTCTTTATTACAAT

ATTTATGGCAAGACTAGGTATTTACCTAAAAAACGTAATAGATTTGGCATTAAGTTATTTGTTTTATGTGATGTCGAAAC

CGGTTATAT

>Tni_Contig_19_hAT *°

ACCAGGGTTTGAATACCGGTTCCAATTTTGGTTCTGATCCTTCATTTAGGTTCATAAAAAGGTTCCTCTTTTCGGTACGG

TTCCAGTTCCGGTATCGGTTCCGGTTTCGGTTCTGATGTACCGGTTCCTAGAACCTTTTTTTTTAATTATATTATACCGG

TTCCTTGCCACCTATTTTAAAGGTTCCTTCCGAAAGAGGGGATGCGTTCGTACAGAGTGTTTTGCAGACTATGCCATTCT

TTCAGTTCGTCTTCGTTCGTTGTGTTGAGTTGAGGGGTACTATATTATAATATAATATATGATATACGAGTAAATAATTG

TCATTGTTACCAGTTGGGCTATATTAAAATGGATAAAGGTAAGTGGCATTTCAATGTTTCGTATTCATATTCCTGTCGTG

ATTTGTGTCTCTGTTTTCTTTATATACCAATTTGTTTTTTAACTCTCAGGAATGAAACGTACCTGTACCAGTCCCGATAT

AGACGACAAATTAAAGCATTTGATTAAAAAAAAACTTCAGGAGAAATGCCAACTGAATGAAGACACGGAAGAATATGAAT

GTAATGCATGTCTATTGCACCGCAACGATGTTGATGACAGAAACCATACATTTTCTCAACTGATTGAACAGGAACTAAAT

AATATAAAACCATGCTTGAGAACTAGGTGTTACATCGACATGCTTGAGTATTTACTGTCAGCAAAAGGAATGCATACTAA

AGACGTAGAGAAGTCATAATCGGCATCTAATTACTACAAAAAGCGTGGGTTAATTCTTTTAAAAAAGATGAATGAGAATG

AATCTTATATGAACGAAAGGTGATTCTAAAGTGTGATTATTGATCATTAAATAGTGGAATTAGTATGTTTCTATATTNNN

NNNNNNNNNNNNNNNNNNNNNNNNNNNNNNNNNNNNNNNNNNNNNNNNNNNAACATAAAACGAAATTATTATTAAATCTA

GATAATTATTAGCACGCGACGTAGGCCCTGATATACTAGGTATACATATTATAACTTATACAAAAAGTAACAAAAACAAA

AAATAACAGCACTTTGCAAATAACTTAATTTTCAATTTGGCGACGAATTTAAAACTAAGTTTGAAATTCTCAATTACATA

ACGCCTACTCTGTTGCGCTTCAATTACGAAGCTCAGTTAGATTATTTCTTAGCTTAGATACTAGGTAGGCAAAAATTTTA

CGTCAAGTCTTCTTATATCCTGATCCGTTTTTTATTGGTACCTAAATATAAGAGAGAAATAATAATAACTATTCTAAGAT

TTTTTAATGTTGCGCAACAGAATCGGGCATCTGGAAATAAGATCATAGTAAATAATCATAGTTATATAGGTTTAAAAACT

TCAAACCTAATAATAAAAAGATCTATGCTTCGATGAAAATAATTATCAAAAATTTTCATTACCTACTTATCTAAATTATT

CCTTAAGAATAAGATATCATCAATTAACTCACTTTTTAAGTGCGTTCGAAGTTTGTTAAATACAAAATTCAAAGAGGAAA

ATAACCTCTCTACACTGACTTGTGTACAAGGCGCAGCCAGTACTATCTTTGATAAAATATGCAAATCAGGCAATATATGT

TTTTTGCTTTCCCAAAATAACAAAATATTTTTGTTCGGAGGAAGGCGCACCTGTTTGTTAAAAAAATCTTTTAATTGGTG

TTTGATATCTAAGTCGTTTCCAGCGCAACCATAGTTTGGATCTCCTCGCTGGGTATTATACGACTGATCTAAGAAAACTT

CGAGCTGAGATTTTTCATTCTGCTGCTGCGTCGAAGTAGAGGCGACAGGAGAGCTAAATAGTGTATTGTCACGGTCTTCT

TTATTTAACTTGCGCCATCTCATCCAAGTACTGACCAAAACCTGTTCTGCGGTTTCTACTTGATTAGTGGTCAGCAAGAA

GTTAATTCGTGGATCCAAATATGCAGCTGCGACAAATGCTTTAGATATAAACAGTACTTTTTCCCTAATCTCCATGTTGC

GATGTAATTCACATGCGAGGGTTGTGTTTATGGCCTTGAGCTCTATTTTGCAATTTATCCAATCAAGGTAAAAGTCACCC

ATAGACAGTTGTTCCTTTTGTAATTTGTTAGTCAAGATTTTGGTTGGTTTTAAAGACGCTATAGTATCCTCTATAGAATT

CCACGTCGTTTCAGATAAATAGAGCTCATAATTATCTGTACACATTGGTCGAAGTTCTAGAAGCCGAGTGAGCATATCAT

AAGTCGAACTCCAACGTGTGGGGCAGTCCACCACGGGTTTTTTAGCCCCCATACTTTTTAGAAGGTGTTGGTACACTGAG

GTGCGTAACTTTTTTATAACCTCACGAGCATTTGAAAGCATCGTTAGAATACATGAAGATTCACTCCCTAAAGCGTCTTG

TACCGCTAGCTGCAACGTATGAGCAGCGCATCTAACTGCCGCAACCATGTAATGTTCACTGTCAAGATAATCTTCGAGTA

TAAAGTTTACTTCGTCACTCATCATCTCCATAATATCATTTTCGTCTTCATCTGTGTCATCTTCATTTTCTTGAATGCTA

GTTTTTATTGCTTGTTCCACTAACCTGACCATTTTCAGCATATTTGCTCCATTATCAGTGGTTATGGAATAAATTTGTGA

GATGGGTATCTTGAAGCGATTTAAAACATAAATTAATTGGTCTTTTAAAAACTCAGCTGTATGTCGTTGGGTGAGTTCAG

TCACACTTAATGTTTTAATAATTATTTGGTTATGTTCGATATACTGCAGGTTAATACCAAAAAAACTTCTCAAATGGCAT

GATGCTACATCTAATTTAAGGCTTATAAATCTTTTCCTGATCTTTGCAGAAATCTCATCTCGCAAATTGTCCGCTTTTTT

TTGGGTAGCGTCACGAATTTTATGTGAGTTTATAGAATTGTGTGAGCATCCTGCCATTGCCATATCCAATATGTTTTGAA

ACGGTTTGTCATCAATTAATGTAAGCGGTCTCCCACATGTCGTCACCAAATTTGTGCATTCTTCCAGTAGTTTAGCTTCT

AAATCTTGTTTGTTTTTTTTTCTACTGTTTCCTTCGTCGAGTTCTTGTCGTTCTGCGCTAT

>Tni_Contig_22_Sola *°

TCGCTAACAGTTTAAAATATCGCCTCTTTACAAGAGCAACGTGACATCTCATAAAAACGTTTTTTTTTTTTATTTGATAT

TCAAAACCGCGATGTCGAATTTTATTTTCTACAAAAACGCGGTATTTTAAAAGTGAATGTTCGAGGAGCTATGAGTGAAA

GAGACGACATGACCGTGGCAAATGGCGTTATCACGTTGTCTCTTTCGGTGTGGCGCACGACTTCGACAACGCGACGCGTT

AGTTTGCCCCGCACGCGCAACGAGTGAAGTGCTCCTGCTTTTCACAAAAAACGTGGCATCACGAGTATACCACGTTTTTA

GTGCAAAGGTATGTTTCTTGTTGTTTTTTACTTTAACTTGATTAATGTGATATTAGTTATTTAACTCGTTTTTGTTGTAA

AACTCATGAGTATTGAGAATGAGATTATGAATATTGTCACATTATTGATGAAAAAGTTACAAGTTTTCCTTGTGATATCT

TTGAATGTAACCATGTTGTTGATGAATCGTTTTCAATTACTGTGGGTTATTTTATTTTACATCACTATTTTTGAAAACCT

TTAAGTTTTGGATTATATCATGCTAAAATGTTACATTATACATGCTAAAATAAAGTAACTTTTACGAATTTGTTTTTCAG

ATGGCATCACGATCTAGGATGTTAGTTCAATTAGCTACAGGTGTTAAAAATAATTTAAGTGTACAACAAAGTGATTCAAA

CTCTCAGATAGATACTTTAACACAAGATGAGAATGAAAATGCCAAAAGCTCGACCAATACATTGCAGACATCAACAGAAG

GTATTTTTTTACTTGTACTGATAATATATTTTTTTATTCTAGTTATGTTCCTATAATTTCATTATTTCATTCCTTTTTGT

AGACAAAGACATCGCATTAGATTCCATCCGACCTCGTAGTAATTCGACTAGTAGCAGCGGTTCCTCTAGTTCTAGCAGTT

CTTCGAGTTCTAGCAGTTCTTCAAGCAGTGGCCCATCTATGTATCAAGGTAAGTTAATTTATATCCCACTTTTCTCACAA

AATTCGAGGGTAATTGATCAAATCAATACCACACCACTACACTTCAAGAATTTTGATTGCATTTATAACGTTTCAGACAG

CGACGACTCAGTAAAGGATCCTGATTATGAAGCCCAACCAAAAAAACGTGCTGAGTATTCTTCTGATACTGACGTGGAAC

AAAATATCACTACTAACTATATAAACATTTCGGATGATCAACATTTGGTAACATCTAATATAGTTTCAACAAACATTGAA

AACTCCGAGGAACCACTTTCGTCTACAAACGAACATGCTATGGTATCCTACGATCTCGGTGTCATAGAGAACAGTGAATC

TTCTGCGATTGAAAACGTAAGTAATGGAAATGCTGTAGGTCTAATAGTGTATAATTCTAGTCCAACTAAAACTGGTAAAA

AAAGAAGACTAAATGTAGCAAACTGGAAAGTCAATGTAGCTAAACGACTGAGAAATAGTGGGAAGCCTTATCAATCTACC

AAATCAAAAAAAAACATGCCAAGTCGTGAATTGAAACCTTCTTGTAAAGATAAATGTAAATTTAAATGTGGGTTTAAATT

TACAGAGGAGCAAAGACAGACCATTTTAAATGATTATTGGAGCTTAGGTGCAATAGAAAAACAATGGACGTTTATTGCTA

ATAGCGTAGACATAGTCATCCCAAAACAACGTTACGTTAAAGTTGATTCGGATGGAAATGTTGCAACTATTCGAAACAAC

AACAACGCTTTCTTTTTGACGATTTCTGGTGAAAAAACAAGAGTTTGTAAATTGTTTTTCAAAAATACTTTGGGGATTAA

TAATCGACCCATAGAGACTGCTTTGAAAAAGAAAAATAGCAACACTAATATTTCTTCCATGAAAGATAACCGAGGTACTC

ATTCAAACCATTATGCAGTGGACGAAAGCATTAAAAATGGCATCAAATTGTTTATTGAGTCTATACCGAAGATTGAGTCA

CATTATATCCGGGCTAACTCAAAACGAAATTATATTGATGGTAGCAAAGCTGTCACAGATCTTCATAGAGATTATGTAGA

AAAATGCAAATCCCAAAACGTCCCCTATGGAACCTACATTACATTTTATCGTATTTTCACACAAGATTTTAACATTTCGT

TTTTCACTCCTAAAAAGGATTTATGCGATATTTGCGAAGCCTATAAAAACATCACAGATGAAGAGAAACAATCTAAGAAA

CAAGATTATGAATTACATCTTAAGGAAAAGAATTTGTCTCGTATTGAAAAAGAAAAAGATAAGAACAACAAAAATATTTT

AGTAGCGGTCTATGATCTTCAAGCAGTGTTCCAATGCCCCAAAGGAGACATCTCAGTTTTTTACTACAAGTGCAAACTAA

ATGTACTTAATTTAACAATTTATAACTTACAAAATAATTTAGTTGAGAGTTATGTATGGGATGAGTCTAACGCCAATAGA

GGTGTCAACGAAATAGCTACCTGTGTTTACAAATACCTGGAGAAAATTTCGGCAACGGCTGAAGACCTCGATGTTGTCTT

TTATTCAGACAATTGTGCCGGACAGCAAAAAAATAAGTTCATGATCTCAATGTACACTTACGCAGTCAAAACTCTGCCAA

ATCTCAGATCAATTACACACAAATACCTCGTTAAAGGACATACGCAGAACGAGGGCGATTCAGCTCACTCTCAAATAGAG

AGAGAAGTTAAAAGGCAATTGAGATCAGGACCAATGTATACACCTGACGCATTTATCGGGGCTATAAAGGCGGCACGGAA

AAAAAGCGAGCCATTTCATGTTAATGAAATGTGCTTCGATGATTTTTTTGATTGGAAAAGTATATGCACTCAAATGAACT

TTGCAATAATGAAAGATGAAGACAATAATCCTGTGAAACTGGCTGGACTGAAGATGATAAAAGTTCAAAGCTCTGATCCC

GATGCCATATTTTTCAAAGAATCGTACGCTGACGAATTATTTAAGAAAGCTATAGTGGTGAAAAAAAAGAGAAATCATAA

TAGTCAAAGCATTAATTTAGATTTGCAAAAGGCCTATCTTCAAAAACCTGGACTAGCAGAGCGAAAAAAAGCTGATCTCA

TGGATTTGGTCAATAAAAACCTCATTCCTAGATACCACAAGCCATTTTACGAATCGTTGTAATAAGACTGATTAAGTACA

AAGATTTTTTGAATTGTATTTTTTAGAAATATTTAATTGCTACATTCAAAATGATAGTGTTTTTATTAGTATTAAGAGAA

TTTGTGATTCTATGTTTATGAAGGTTGATAGCAATGTTCCTATTTTGAATTTCTAAGTACTATAAAACTGGGTGCCATAA

TTGTGTTAGAACTTAGAAGACGTTAATTACCATTAGATAACTTTAAGTACAGTTATCTTTTTGATTTTTTATTTGTATGA

AAGTAATTTTTTAGTTACCTTATAATTTTAATTAAAGCTAGTACTAAGTACTATGTGATTGATTGATCTCTTGTTGATTA

TTGTGCCTTAAACTAATTTTATGGTGTAAGTAAGCCATTGTTTTCGTAAATTTGATTTTTTTTAAATACAAGATTTTTAT

TTTGATAGTGTGTTTTATTCAACTTATCACACAGCGTTACACCTAAATTTAACACCTTCAATGTGATACATCAATATGTT

ACAAGCATTTTTTGTCATATTTTCATTAAAAACGTGACATGTGTGTCTGTGTTTTTTTAAATCAGTTTTTAACTTGTTTC

ATATTGAACTGAGTTTTTTTTTGCATATTTTCTGAAAGCACTTTAATTACCGCAACAATTTAATTGTTTTCCGACAGTAA

AATTGTCAATATTTTAGAATTTATCAACGCTTAAATGTTTAAAATTGCTCTCCTGTAAAGTTGTGGTTTATAAAATATCA

CGTTGTTCTTGTAAAGAGGCG

>Tni_Contig_10_Sola *°

AAATCGCACGCTTAATCATAAAGAGTAATAACTCAAAAATCATGTTTTTTGAGATTTAATCATAAAACGTAATAACCCAA

TAGTATAAATACGTACGCTCCGACTGCCGCTCGATTACAATAAGTGTGAGTGAAATAAAGGTTCGTCCCTCTCTTTTTGA

TTTACTCGCGCGGACAAGTTGCAACCAATGAAATGCTACAATCAGTATTGCGTCAGCGCTTACGCTAGGAGGACGATCCG

AACAACGCAATAAATTGAGTTAGATCTGTTTATTATTAACAGGTAAGTCCCTTTTTATGTTACTATTATATTATAAACTG

ATTTTTTTAGTTTTATTACGTTATATTACAAAAATATGTTCATGGATTGTTGACATATTTCGACTTAATGCAGTTTAGTT

TTAATTTTTTGACCGCTGTTTGAGTATCAATTTGAGTTAATGCAGTTTACTCTTAAAACACATTTCAATTGTATGGAGTA

TTTTGAATTATTTCAGTTTATTATTAAATAATACTTTAACTTCATACATCAGTTAGATTTATTTCTATTTATGATTAAAA

TTATACTAAGTTTGATGGCACATTAGGGCTTAATTCTGTTTTCTTTAAATAACCTAACTCATCGAGCTTGATAGTTTTAT

AATAAACTGGAGTATTTTAGAAATAAGGCTAATTTTTCGGTTGTCTGTATCGCTAGTGCTAATTAAAATAATAGTTATTA

AGTTCAGTTCGTTACTTAATTTTTTGACTGTTTTGCCTATTTTGAGCCAAACGCTTACACAGATTTAAAGTTAGCGAGAG

GTGCCAAGGGTGGGAGTGCCGCTTGATATTTTACCTACCGTAATTTTACGTAAAAAATAAATCTAATATTTTTTTTTAGT

ATGGCGTCTTCAAGAGCATGTCGAATTATGCAACTGCTATCGATCGAAAATAAAACAATAGAAGCCAGAAGTGAAGATGA

AGACGAGACACTGAAAAGCAACATCGACAATGTTCCCTCCGAAAAAATAAGATCTCAGTACAGTGAAATGTTACAAGACA

AAGAAAACTTAGAAGAATCTTTAGATAGCTGTGATGACATTCTATCGGATTACGATAGTAATATAAAATATGATCACCGA

TTGATCACTTCACGTAAAGTACAACCAAGCTGCAAAAATAAAGAACATGAATTGAAAATTTCCCAAGTTAGTCTTGTGCC

GGCTTATGATAGTGATGATTCTTCTGAAGATAACAGTTACATAACTCGTGCTGCAACAGTCACTGAAATCAGACAAAGTT

TGCCAAAACCAAATTCACCCTCTTCGAGCTCAAGTTCATCGTCATCTAGTTCATCTTCGTCCAGTACTTCGTCGTCAGAT

TCCGACAGTTCTTTATCGTCTGAAGGACCAAAAAGATGTTCTCCGGAATTAACTACTAATACAAATGAGTATAGGATTAT

TGACTCGCAGGTAATTGAACAAAATCGTGGGACATCATCTATAGAAACGGCACAAAGCGATTCGAGCAAAATGATTTTAA

GACAGCAAAACTTTAATAACACATCCAATAATATTTCACCCATGTTTACTGACGAAAGTGATATTGACTTAAGTGATGAT

GACCCAAGTTTTGATTACGAAAAATTGATTCATAATAAAAAGAAAAAGAACTACATTTTTGAGTCTCACACTACAGAGTC

TGACAGTGATGGTAGTTCCCAGATCAATATTAATAAAAAAGGCCGTAAGCGCACAAGGAACCCTACAAAATGGAAACAAA

ACCAAATAAAAAAACTACGAAATACTGGAGAATCATATGTATCAACAGCGAAAACCCATAAAACTATTCCTAGCCGGTGT

CTTAAAATCCCGTGCACAGAAAAGTGTAAATTGAAATGCACTGAAAACATATCTACTCCTGACAGATATGATTTATTCAA

AGAATTCTGGGATCTTGGAGATCTTAATAAGCAGAGAGCTTACATATCCACTTGCATGGTTGATATAGTACCTAAATACA

AGTATACAAATGCCGAAAAACCTCGGCGTCCTAATAAGGCTTATTATTTTACAGTCAATAATATAAAAATTAGAGTTTGT

AAAACATTTTTTAAATCTACCCTGGATATTACCGACCGCACAATTTTTACTGTGCAAACCAGAGTAAGTGAACGTGGCTT

TATGCTTGAAGACATGAGAGGCCGACACAATAAACACAGGACTCTTAGTTCGGAACTAACTAGTGACATAAGAAAACATA

TTCAAAGTATCCCAAAAATGGAATCCCACTACGTACGAGCTAGTACTTCCAGACAATTTATTGATGGTGGAAAAACAATC

AAAGACTTATACAAGGACTTTGTAGAAGACCAAAAGAAAGATTCCAAAGAGTTTGGCAATTATATAGCATATTATACAAT

ATTCACAAAGGAATTTAATTTAAGTTTTTTTCAACCAAAAAAAGATCAATGTGATCTATGTTTATCATACAAAAATTCTG

TAGAAGAGAAAAAGAAAGAGCTAGAGGAAAAATTTAACACACATTTGGAAGAAAAAGCACTTAGTAGGCATGAGAAGCAG

GAAGATCGAAGAGTGATAAATAAAAACAATAAGGTCGTTATATATGATTTACAGGCAGTATTACAGTGCCCAAGAGGCGA

TAGCTCATCGTTCTATTATAAGTCTAAACTAAATGGCTACAATTTAACTTTAACCGAATTGACAGCAGCTACATCAAAAA

CGGCTTATGACAATGTACATTGTTATTTTTGGACAGAATCAGACGCGAAACGTGGTGCAGTTGAAATAGGAACTTGTGTT

TTGAAATATCTTGAAAGACTTCGTGAGGAAGATACAGAAGAAAAAAATATCATATTTTATTCTGATAACTGTTGTGGTCA

GAATAAAAATAAATATATCGCGACATTATATTTATTTGCCACACAAAATTTTAATATAAACACAATTACACACAAATTCT

TAATTACTGGACATACACAGAACGAAGCAGACAGTGTGCATAGTTTAATTGAGAAAGAAATTAAAAAGAATCTTAAATCA

GGGCCCATTTACAGCCCTGATCAGTACATAGCATTAATAAAAAATGCAAAGAAATCCAAACCAGCCATAAATGTCCATGA

ATTAACATTCGAGTCTTTTATGGATATGAAACTCTTGCAGGAAGATTGGGGTTATAACTATAACATAGATATTGAGGGGC

AAACGGTAAACTGGAATAATATTAAAGTGTTGTTTATGAAAAAGGAATGCCCGTTTAGTATATTTTTTAAAACCTCTTAC

AAAGACAATAATTACCGAGAAATAAATGTCCGAAATAAAAGAAAGAAAATGAGTATATTAACACAAATAACGCTGCATAA

AGCGTACACGCAAAGACAAGACATAAGTGTGAATAAAAAGAAAGATTTAAAAGATCTTATAAATAAAGGTTTGATCCCAC

CTTTTTATACTAATTTTTATAATTCTATTATTAATTGATTGCTTTCATTCAATTCAGATCTCATTTATGTGTGATTATTA

TGATTTAATTGTGATATTATTTGTTTAAAGTACTACTTACTTCAAATCGTTATTGTACTAAGTAGGTACTGTTTTATATT

TGGTATAAAAGACTGATTACCTATTGCCAATTGTTTTAATTACTGATAGAAAGATAAAACTGTTCCTTTTGTTATTGATG

TTAAGTTGCTTTTATTTTATTATTTTAATGAAAGACCTATTTGCTTCTTATAATTTATGATAGAAATAAAAATATTTTTG

TTTTCATTGATTTTGAGTTGATTTTTTATACCTATTATTTTAATAAATATTTTTCTTTGATAATTCATGGTAAAAGTTAT

AAACATTTCGTTTTTATTGATTTAAGTTGATAAGGCATGTTAGTTTTAAGTTCACAATTTTGTACCTATTTTTAATTAAA

GAAAATATTATGTTGTACAAACAATCGTTTCTTTTATTTTAAGTTCATTTATTTATGCCTATTTTTATGATTTAAGTCAG

GAATATACCATTTGTAAGGGATGTAAATAAATACACAATTTCGTAAACTCTGATTATTGCGAAAGTAATCCATAAATTAA

TACAAAATCATGAATATAAATACGATGTTACGTTTTATTATTAAATGTTTTAAATATGTGAGACACGGTTCTATTCATAA

AACGCATTATTGTAATTTATTGTTAGAAATATCTACGTTGATAAATCATAAACTGAACTAACAGTGTATACTAATACAAT

ATGATATTTTATTTTATTCATAAACTGGAATATTTCCATATACGTTGGTTATAATTTGACTTCCAAATATAAAATTTAAT

AATAGACTGAAATAAGTCGAAACTTAATTTTCTCTAACGATTTTCTTGGTCAAATTTTTATGAAATTCATTTTTCCTGGT

TTCATTAAATAGCTTGATTATCAAACTATAACCTAAAACTTATTTTTTTATT

>Tni_Contig_21_Mariner

CGTAAAACGGGGTGAATAGAATTCACGGGGTGAATAGAAATATTCTTGGGAAAAAGTCTAAAAGCCTGTGATTCGAAAAC

TACTATAAATATCGCAATAGTGGTTTTTTTTTTTACAATGGCAACATTTTTTTTTTAGTCTGTCAACATTTGAATAGTTT

GAAGTTGACAATTAATGTGAAAAAAGTGAGTTCAAAGCACAACGGTTGTGTGTATGTCTTTTGCGGTCTTATTAACTTTT

TCCTAGTTATTATTTAAATTGTGTTTTCTATAGTACAAATTAGTTAATACGACGGTGAAGTGTATCCAGAATAAATTGAA

AGGGTAAGTTTTTCTGATTAGTTTGATTTATATTGTTAAAATAGTCAAAATTTAGGACCCCTAAATGTTTCTCGATTACG

AGGTGAATAGAGACGACTGAGGGGTCAATAGAAACGCCATATGAGGTGAATAGAAACGGGAAAAGGGTAAATAGGAACGA

GAAAGGGTTTAATAGAAACGCTAAAGGGGTTTTTAGAAACGCTTTTGATAGGGTTGGCACTTTATGCAGGTTGCTGAATT

CTTACTTAATTACCTTTTTAAAAAGTTGAGTTGTTTATGCTTTAAATTAAAACATTCATTTTGTTATGACGAAATCAATA

TTTTTCAAATATTTATTATTATTTTTGCATACGCAGGTATAGTAGCAATATGCCACGCATCTACAAACCAGACCATCGAG

GTAAGAAGTATTTAAAATATGACGTCAACTTAATACAACAGGCTGTTAATGAATATTCTGCGAGTAATAGTTCTCTTGAT

GCTATCTCAAAAAAATATAATATACACAGATCTGTATTATACAGACACTGCACAAAACATATGAAAAGTCAAGGAGGHCA

AACTGTTCTATCGAAAGAAACTGAAGAAGAATTTATTAAATACATAAATGTATGTGCTGACTGGGGCTATCCTTTGGAGG

CATATGATTTAAGAATGCTTGTTAAGGTCTATCTCGATAAATTGGGTGTCAATGAAAAGAGATTTAAAAATAATATGCCT

GGACCAGATTTTGTATCCTCTTTTATGAAAAGACACAAAGATTCAATTACTCAGAGATTATCGCAAAATATAAAGAGAAA

CCGCGCTGTTGTTTCCCCTGAAATAATAAAAAAATACTTTGAAGAATTAGAAATTTCATTATCAGGCGTATCTATGTGCA

ACATTATNNATTACGACGAGACGAATCTTTCGGACGATCCTGGGCGGAAGAAAATAATTACAAAAAAAGGCGTGAAATAT

CCAGAGCGGGTGATGAACCACTCGAAGTCTGCTGTTTCTATTATGATTGCGTGCACAGCTGAGGGAGAATTACTGCCTCC

TTATGTTGTCTATAAGGCACAACATTTGTATGATACTTGGACCGTTAAGGGACCAAAAGGGACTCGTTACAATAGATCAC

AGTCAGGTTGGTTTGATGGAAACATTTTTGAAGACTGGATTAAATCGGTAATCATACCCTATTTTCAAGGAGTACCCGGA

AGAAAAATCTTGATCGGTGATAATTTATCGTCGCACCTTTCATTACATTTAATTAAACTATGCCATGAAGAAAACATAAG

TTTCGTATTCTTGCCAGCGAATTCAACCCACATCACACAGCCATTGGATGTGGCCTTTTTCAGGCCCATGAAAATAGCTT

GGCGAAATATATTGTTTCAATGGAAAAAAGCCGACGGTCGCCAACAAGCATCGGTTCCAAAAGGATGTTTTCCACGTTTA

CTGACTATGCTTTTAGACGCCCTAAAAGACAATGCTAAAGAAAATATTTTATCAGGCTTTAAAAAAGCAGGAATAAACCC

ACTTGACCCTACTCAAGTACTCAAAAGATTACCAGGTTATGAAACAGTTAGCCATCAAACGGAAACAATAGATGAATCCG

TTCTCACGATCTTAAAAGAAATGAGATTTGGTACGATAAATGTGACTGAACCTAAAAGAAAAAGAAAAATAGAAGTAGAA

CCAGGAAAGAGTGTTAGTGTTGAAGAAAGTTATCCTGAAACAGATATAGAGAAAGAAAACCCAAAGATAAAAAATAAAAA

GACAAACAAAGAAAGTAAAGAGAATAAAAACCAAAAAGAACCAATAAAGAAAGGAAAGGGAATTGGAAAAAAGTCAAAAA

TTCAACCACATTCAAATGAGATCAAAGATTTCGTTTCATCTGTAGATGAAACGAAATCTTTCGCCTCTAACTTTTTACAA

CATGATCCTGATCAGATTACTATTGAACCAAGCACAAGCTTCTGTAATGAAGTAGGAAAAACAATCTTAGTATCACAGTC

AATGAGTACAGGTGAGGCTTATGATTACTTAGATAATATAGAAACGGTACCAATTATTTTTGGTGATGATGTTGTCATTG

ATCATGTTGAAACGATTGATAATTTAAATGTTTCTGATGCCACGTTTTCGAGTCATGAAAAAAAAAACAATAAGAAAATT

GAAATTATATCTGATATTTCAGTTACTGATCCTAAGCTATTAGCAAAAATATTATATGATAATAAAATGAAAATAAATAA

ACCAAAACATCGAAATTATTACAGAAATGATGAGKAAATATTATCCGATTTAATGTCTGATACTGATTAAATTTTTGAGA

CCCTAATTATTTTGAACTGATTATTGTACTGATTTGTTGATTATTGTCAGAATATTATTGTTTCCGACTGGGTTGGTAAT

ATAATTTTAATACGTTTAATAATTATTAATTAAACTTAATTGTTTTGTAGTCTTAATTCTGAGGCTAAAATTATTTTTGA

ACTGATTATTCTGTTGTGTTGATTATTGTCAGAAGATTATTATGTTTCACACTGAGTAGGYAATATAATTTTATTACGTT

TCATAATTATTAATTAAGCTTCATTTTTTTGTGATCTTAAGAGCGCGTATGCCAAAAGTTGGCAACGTCGGAAATAGCTG

TCAAATATTTACATGTTGTAATTAATTTGGCAATTTTACTGTATGGAAATAAGAAATAATGCTCTGTAGTGCACTACAAA

AATGATTAGTGAATGCAATAATTAAGATCGTAACACCGAATATATAGTTTTAGATGTAAAAAAAGTATTTGGGAACTTGC

CAAATGCGTTTTTTACACCTGAAACGTTTACATTATCGAAATTTAGGGTTTTAATTGCAATTCATATTCAATTTTTTAGT

GCACTACAGCCATATTTGATTGACATGCACGACTGATTATTTAATAAACATTGTTAATGGTTGCGATATATTCTATTTAA

TTTTAATAAAAAATGTTTTCTAATATATTATGTACACTCTTTCATTCCATTATCCATGTACCTAATTATTTATATTAAAA

CAAGAACAATGAAATGACGACGTAATTAACATAAAATGTTTTGACAACCCTATCACTTTTCTCTATTAACCCCATACGTC

GTATCTATTGACCCCGTAAGCCGGACAAATAGAAATTGACCAGATTTTTGTCTTTTTCTCCATAATAGATAATAATTTGG

CATTAAAATTGTAATTAGGTTGTAAAGTAACTTGAGACTCCCACGATGAAAGCAGCTTTATTGCCCGAAACTTAGAATTT

ATATTATTAAAGTCAGTTTGAAAGTCCAGATTGTTTCTATTAACCCCGTTTTACGGTATT

>Tni_Contig_17_Mariner *°

GGGTAAACCACCCAATTATTGGCACTTTAAGCAGCTACTTCACAAACCCAGTAAAATTTGCATGTAAAAATATGTTTTAA

CAAAGAGTAAAGCATTTATATTGGTCTTCGAATCTCTGCAGAATATTATAAAGAACTTATCATTAAGAAATTATATCGTT

TTCTTCAGTATAGTGTACCTTTTAAAAAAATACCTCAAAATACCAGTTGCTGGCATAGCCAAACCTTTTATTGGCAGTGA

TTATTACCTATTATTGTCACTGTTCTCCCAAGACTGAAAAATTGCTGCTAGCTTTTATGTAGGGGTTTTAAAATGTATTC

TACATCGTCTATGTACCTACCTATTTTAAGCTAATTCATAATAGACATAAACAAAATAAAATCCTTTGTTAGAAGAAAAG

TAATATTTATTTGTACAGGAACATACCAAATCAGTTGAAACGCTTATAGTTAAATAAACTTTCTATAAAGTACCTACCTA

TTAAAACAAAACCAAAAAAACCTTCTACCTAGGCTTGAACAAAAAATAGAACAATTTTAGCTAAAGACTATTTAACTTCC

TTAATTATATTTAATTAAATCACAGCTCTAATTGATTAAAATCATAAGTAATTCTTAAGTCACATTAGTCATCTGAATCA

GTCTCAGTCAACATTTTTTCTATCTCAGCAGCACTGTAATTTTTGCTAGTACTTGGTTTTTGGTTTTCGTTGTTATCATT

TTTAAGTACAGCTTCCAATTTGCCATTGTCAAGTTTATAGAAATCTTCCGAAAACAAAATTTTGGACACGTCTTTGGCTT

TAGTTTGTTTTTTGTTTTTCTTTACCATTTTCTTTTCTTTTTCCAATTTCTTTTGTTCTCTTTCTTCTTTTCTCTTTTTA

ACACCTTCTGCTTTCTCTTCTTTAATTTTAATTTTTTCATTTTCCTGAGCTTTCCATTCGGCAGACGTTAGTACAAAAGG

CGTCTTCTTGGTATTTTTTATCCCTTTTCTTTGTGGCGTTTTAGGAAGTACCAAAATATCTTGTAATTTTACTATAGTAT

TGTCTTCCTTAACCAGTGGTACTTTCAGTTGCATAAACTTATTTTGATCTGCACCTATTTTGACATCTCCAGTTACTCTG

ATAGACGGTTTTTGGGGAGTTGAATAAATTGATGATTGCTGCTGACTGCTAACGGACGTTTGGTAGATAGGTGTTTGTTG

AACAAATGTTTGCTGTGTGGATTGTTGTTTGTTGCTTGGTACGTAAGTGAATGATGGCTGTAAGATCGAAGTATGCTGTA

AAGAAGTAGAGGCTTTTTGAGAGGATACTTGCTCTATGGATTCTGGCTGATTGAATGCTTGTTGAGTGGGTGAAAGACGA

TTGTGTGGTTGTTGGCTAACTGTTTCTGGAATAGGTTCTTGTAGGGTTGATAAATCTTCTATAAAATAATTTAATTCAGA

ACATTGGACTATTTCCTCAACAACAACATCTTGTGAAACAAAATCTAAACTTTGCTCAGCAACATTTTTGTTTGGAGAGT

TATTGATAATTATCTCCATATTTTCAATATCAAAATTCCCTATATTGTCATTCTGACGCTCTGTGTCTAATATTGAGCTA

TTCTGCAAATCATTTTGAGACGTGGTAAAGAAATTCCAAAGGAGAACCCCATTTCTGGGCCTAGCCGTTACACGAATGAG

ATATGTACCAAAAATATCTACTATTTTCCTTTATTAACCCATTTAAATCTTAGTCTCAATTGCAATGGCCAAGTGTAGGA

AATAATTACATTTATATTTTTTTGTTTTAAACAACCTTTGTAAACCGATATAGAACTTATTTCATTATACGTCACTTACT

TCGCAAACGCTAGTATCAAACAATAGAGCGTACCCTAAGAATATTATCGATTAAGTGAATAATATAAATAATTATAAATT

ATAATTGAATAATACTCCATTATTTTAGTATAATTGTTTTTTAGATATCTAAATCATTATAAAATGAAAAAAAAACGGGA

TTCTAATTACTTTTTAACGTAAACACCATTAAAAATCATTGAAGGAAATAAAAAATCGATAAAATCGATTGCATCTCGGA

TTGCACATGGATTGCACTAGTAAACGGCAAATGTCGATGTCAACGTCTGCCAATTGTCATCGTTATAATATAATTAGTGT

CATTCGCTTTTCGTATCTTTTCGCAAGTGCATGTGTTTTTCTGTTTCTCGAGATTTATTACAATATTAAACCTCTGCTCG

AATATTGGACTCCGAAATTGATTGCCTTCTGACTGACCGTCGCTTGAATATTGGACGCTGTAGCCTGCCATCCCTGCTGA

GTTTTGCTTTAAAACCGGATCTGGATATTGATTTTTTGACGACTGGTTAATACGGTATCATGAGTTTGTGCGTTAATTGT

GGAATAGCAACGGACCGCTGCAACACAATAGGAAGGCGAAGATTGGAAGGTGAAGATTGGAAGAAGAAATCAATCCTCCT

GCACAATTCCTAAATAATGTACTATTATATTATGATAATCCTAACGAAGAAGGTGATTAATAAAACACTATCTCAATAAT

GCGGTATTTTATTTTAATAAACCTACTGTTGCAATCTCAGTTTACTGTAATCTGAGATAGCATTAAATCCCCTAGTTGTA

AATACAATGTTAAAAATACAAGCCAGTAGTGATATAAGTGGGCCTTATCTAATAAACATAGTTTTAAGTAAGGTTAGTCT

GAATTCACCACTTTTTATGTAAGTACCGAAACATCATCAAGATAGGGTGCAGGCGCTACAGATCGGCGCATGTGCGTTCC

ATATTAACGTAGGAAACAGCCTCAAAAATGCATTTTTTTATTTAAATATCGCAGTGGCTGAGCGTTGACTGCTGTGCAGC

ATGTAGCGGGTTCGATTCCCACACGGAACAAATATTTGTATGAGCTACATGTAGTTGTTCAGGGTCTGGGTGTTTGCGTA

TATGAGTTTAAACGTTTGTAAGCACACCCACGACACAGGAGAAATTCCTAGAATAGGACAGTTTTAGAAAAAAAATATAT

AACCTAACATGTAATCAGTCTGATTTTACCAATTTGGCCGTTGCTGGGAGACGTGAAAATTGGGCTCATAAAATCTTCCT

AAATGCTCTAATTTATGGCGAAATTCCAGCCCTGTAACGTCAGGCCCAAGTTTCTATGGAGTTTGGGTTTCTCCTTTCAA

TAAACACTCACTTGACTCCTCTGGCCTCAATTCTCTTTTATCAATTTTATGTATTGCACTTTCTCCCAACAACTTCATAA

AGTCATCTTTGGTGAGCACCCGATTGTCTGTTACATGCTTGGGTTGCGCTATGGTAATGTCATTTTTATTTGAAGTTCCT

AAGCACTTGGAATAGTCCACTGCATCTTCATTAAAAGGACAAAGTCCAGTTGCTCTAAAACCATTTATAATTGTTGACGG

GTTAAGACTTTTTTCTATAGTATCTTTAAGTAATGGCACAAATTGAGTTTTCGTTAACGCTTGAGATGGATTATTTCTTC

GCCATTCTAGGACAGTTTTCTGCCATCGTGTTTTTAATGGTTTAAACGCGGCGACGTCTGCAGGTTGAAGGAGCCTAGTG

CAGTTAGGATATAACGCTATGAGAATTATACCTAAATTAGTACATAATTCACTGGTAGCATACGTCAAGTGCGATTTGTG

GCCATCCACAAAAAGAACTACAGGGAATTGTGTGCCAGTTTTTACGAGGTGAGGATGTAAAACGTTTTTGATATACTCAA

AAAATACATCAGAATTCATCCATCCGTTGTCGCTTAAGCCCACGCCCCAATCTTCAGGAATCTTAGACGTAATCTCGCCA

GGTAATCTTTTGTATGGAAAAATTACCATTGGCGGAGTAACTTGACCATCTGCACCAAAAGTAAACATGACGGTTAGTGA

AGTTTTGGCTTGGGCGTGGTCAATTTCGTAAACGTTTTTTTCTCCCTTAGAGCTTAAAACAATACCTGTTTTTGGACATA

GAACAAAATTCGTTTCATCGCCATTGAATACTCTTGATGGGTCTGCTAAGATGTCATAGTAGCTGTTTTCAGTCATGTAA

ATCCTTATTTGTTCAAACCAGCCTCTTACATCAGATGCAGAGACCGTGGAGCTGGCAGCTGTTACGGCTTCTGGTGTTCG

AAATGTAACTGTTGGATGTCTAGCTAAAAACAATTTGAACCACTTCTCACCATTCTTAAAACTGCTGGCTCTCCCCGATT

TATTGATAAAGTCTGAAACACTTTTTATCAAATCTTCTTTCCTCTTCGGAAACCCTTTTTTGCTACTGATTTTTATCCAG

TCAACAAAAAGCTGTTCTTCTTCGTCAGTTAAAATTGTGGACGGACCGGGCTTAGAGCGATGATCAGGATGTTTCAATCT

AAATTGTATAGTAGACCGAGGTACGCTGAAGGTTTTTGCAGCAAGTTTTTTGCTCATGCCTTTTGATATTTCGTGGAGAG

CTTTTTGTAGGTTCTCTTCTGTATAAATTTTCCGTTTAAAACATTTTTTACTATTTTTTTCTCTAGACTTCTTCTGACAA

GGCTTCATTTTGAAAGTGGCTGAAAAGACAATATTTTTATTGGCACGTTTTTACCCAATGTTTGACACCCATGCCAATAA

ATGGATTTTATATAATTGGCAGTGGGCCAACTTTTGGATTGGTGTATCCAAAACATTAGAAAATATTTTCAATGCAATCT

AAACTCAATTTTGAGACAAACAAGGTATCATTTTTAATAAACTCAAATTATTTTAAATGCCCTTTTATTGGCATGTGTGC

CAATAAAATGGATAAACCGATTTTACACATACCAAATGTTGGCCCCCCACAAATAACTAAAAATTGGCGGTTAAATTATA

AAATAATGTTTTTAAAAGGCAGTTAATATATTCAATAATAAGGCAAAATGTTTATGTATAAGAAAAACAAAATAGATTTG

CAATTTTTAAAAGTTTATGATAGCTCTTACCTTAGTAAAAAACGAATATTTTGATTGTTTTTGTCGCACAGGTCCCGCCT

CGTCAAACAGATGGTAAATACGTCGCCATTTTTGAAAATAAATAAAACGTGTGTTGCCAGTTGAGTAATAAATGATTACT

ACTGTTTAATTTCTACTAAAATATATAAGGTCTGGCTACAAAAAATCTTAATTTTCTTAAAAAACTAGAGCATGCCAATA

AATTGGTGGAGTGCCAATGATAGGGTGGTCTACC

>GBKU01035396_Harbinger *°

TTTTTTTTTTGTATTAAGATATGTTTATTGATTGTTTGTGTCGTTTTCATAGTTTTTTAAGCAAACATTAATTACTTCTT

CAACTATTTGTGCTCTTTCTTTAGCAATTTTATGCGCTTTTCGTTCACATTCTGTTGATAAATTGCTGCCACCAAATTTT

TGAGAGCCACAAATGAATCCTTTATTGTAGCATATAATTAAATTGGCACCAAGCCCTCCACACATATCCTCTTCATAAGA

TGATGGGTCGGCCAGAATAATATTCCTTTGCTCTTGTGTATTCATTGCAAAACTTGTAGCAACTGGCAGTCCATGTACAT

TTAGTTTTGATCTCACGCTTGTATCTACAGCAATCTCTTCAGTTTCAGAGTCATAGGTTACTGTTGGAAGAGTCAATGTT

TTCAAACTGGCCATGAGCGCAATAAGACAGGCATCCACTAAACTGCCATCATTGTCCATGCAAACCATATCACAATACAG

TACCCATGCTAATTTATCTGATGCAATGCACAGATCTTTAAGGTCAATACATTTTGAATTAATGATGATATCTGATACCA

AATTACTGGTGACTTGTGCATTATCTGAAGGAGGGCCAGGCCTGTACTTTGACGAACATAAAGGTGGAAGTTCTATGTTT

GTCACTACAAAACCGAAATCTGGCTCTTCGGCTTTTGGTGTAGCTAGTTCCAACTTGATACCACAGACTACAGTGGTGTT

TCCACATTTAACTACAGCTGAGGCATCCGCAGATTTAATTGAATTTACATTTAATTTTATATTTCGTTGTTCATGAAATT

CTCTTCCATCTGGACGAACTTCTCTTGATATGTAGTCATTGAAGTACTTAACTGGATGTATAAGTTTGTATATATCGGCC

ATTTTCTTACTATTTCAGTATCTTGCAGGCTATACATAAAAGATGCTTCATACAAGAATAAATATACTTATAAGTTAAGT

TTTACTAATTTACTACAGTACCTTGCGTTCTCAACAATAATACAAAGTACTTAACCTCCAAAGTTGTGATTGTTTTTGTA

TCATTGACATTACATTGACAGTTGAGTTGAGCATGCAACTTTTTCTGAAGCCTTTGCAGCTCTGATATTCCAGTTTATGG

ATTACGTATTGACATAGCGAAAGAATCTTAAAGCCATGTATTCACTGAACAACAGTGTTTCGGATACACAGTTTCTGAAA

CATCACGTAAATGTTTCCAGGAACAATGTTTCGGAAACTCTGCCGTCCACACAGTCAGTTGCTTAGGTCATGTCGCCCGA

GGAAGTAGTGTTGTTATGTGGTGCCTACATTTTTTTGCATAAGTCCATTTCTAGTAAAAAGAAACGTAAAAGATGGTGGG

TCAGGAATTACTTGCTAAGAAGACAAGACGTATTAAGTGATTTATGCATGTTTGATGGATCATTTATAAACTTTCTACGA

ATGTCAAAATCAGATTTTGAATATCTTTTGCAAAATGTTGGACCATTTATAGAGAAACAAGATACAAACTTACGAAGTGC

AGTTACCGCTGAAACAAGACTAGCCATTACTTTAAGATATTTAGCTACTGGAGATTCATATTCTTCACTCTCATACACTT

TTAGAGTTTCCAAACAATTAATCAGCCGGATCATACCCGAAGTTTGTAAGAATATAAAACGTGTGTTGAAGAACTACATC

CAGGTAAGAAAAACTAAGTTATGAATCAATATTTTTTTTATTAATTATACAAAATTAGGCGAACTATCTACTGGGTACTA

CTATCGGTTTCTTCTACTGATGCGATTTGTGAAACTATCTCTTCAATGGTTTCAAGTTCTTTTTGTGAGGTTGAACTTGC

TGGTGTCGACGCAGTTTCATATCCGGGATGGCTATGATATCCGTAACCATAATTGTATTTGCCCATTCGCATGTCTATTA

AAATATTATTAATATAAGCCTTGGCTTGTATTACGGCAGTCTCGTTTTGTACCGCTCTTAATTCTGAAGCGACGTATTGC

CCATAAATATCATATTCATCCTTAGATTCATCCATTTTGCGTTTTGCAGATTTGACTATTTCAAATACTTCACTTAGATT

GTCATCCACTTTTCTTTTAGATGCTTTTTTGCTACTTGTAGATGGTAGTGGGGATGGTGGAAGTGGAGATTGTGGTTTCT

GCTGCTCCGATTGCTGTTCTGGCTCCTCAGTTGCCAGGAGGTCTTCACGGTTTGCCTGCAATTTTAAACACTTTTTTTTT

AAGGTTCCGAGTTCCGAAGCGCACTGGAAAGAAAAGGCCCGTGAGTGGAACGAATTATGGAATTTTCCACACTGTGTCGG

AGCTATAGATGGGAAGCATGTGGTTATTGAAGCGCCTAGCAATAGTAACAGTGATTACTACAACTACAAAGATCAGTTAA

GTATTGTACTACTGGCTATTGTAGATGCATCTTATAATGTTATATATGCAAACTGTGGTGCGAAAGGAAGAGCATCTGAC

AGTGGCATCTTTCAAGAAACGAGTTTCTATCAAAGAATGGTGGAACACCGTCTTAATTTTCCAAACCAAGAAACGATATC

ACCAGATGGACCTGACCTTCCTTATGTAATACTGGGCGATAGTGCATTCCCATTATCGGAAAACTTGATGAGGCCGTACC

CGGGTATTCATAACCGTGGCACAATGAAACGAATTTTCAACTATAGACTCTGTAGGGCCAGGAGGGTTGTAGAAAATGTT

TTTGGAATTTTATGCGTGGTTTTTCGTGTATTTCGTAAACCAATTCCTCTCAAGCCTGAAAAGTGTGAACTTATTGTAAT

GGCATGTCTTTACATGCATAACTTTTTAAGAAGGAATAAACAATCTAGAGCCTTGTATACGCCCCCGATGACTTTTGATT

TTGAAGACAGTGACCATAATGTCATAGAAGGTGCTTGGCGTAGAGAGTATACAGTTGAAGGAACTTCAATTTTGGAATTG

GAAAGGCGACCTAGAAATTCAGCACGTACTGCTACAATCATAAGGGATCAATTTGGTGAATATTTCATGAGCGAAAGAGG

AAGCTTGCCGTAACAAAACAATGTAGCTTAATGCTATGGCGGTAGTGTTTTCGTATATGTTAGATAGAATATTTACATGC

CTACAGCTGATAACATAAAAATAAAATATGAATAATGCGCTCACTGTTTCATTTAAAGTATCCGCACCACCGACCCTTGT

CGTCGTGCATTGTAAGAAAATTAATTCTTCATAAAATTTCCATTTTGATTTCCTTATATCTGCAGCACCAGCACCTGTGG

TTTTTGTAGACAACATTTTCTTCCTCTCTCTTGCAAATTGCGACCGAATTTTATTCTCAACATCTGTTTTTGGAATTGCC

AAAGCATTCGAAATTTCTTCCCAGGCGTCATTCTTTTTTATTTTATTTTTATAGTCCTTTGTCGAGCAATCCCATAATAT

TCGTTTTTTCTCAAATAGTTCTATCAATTTAAAGACAGTTTCATCGTCCCACACTACAGCCATGTTTATAAACAAATGTT

TAGACGCGTCCACACTCGCATGCACCTGTTGCGCAACTGATTTGCTTCTGTGTTCTCCGTCACAAGTGGGGAGCACCGAA

ACATACCGATAAACACGTAGTGTTTACAGAAACAAAATTATATGTTTCTGAAACAAAATGATTTGTTTCAGAAACACGTA

GATTTTGTTTCTGAAAC

>GBKU01001550_Gypsy *

GAACAGTTAGGCCTTGAGATAATATTTCACGTCCAAGCATTATGTCATATCGAAGGCAAAAGTCGGGTAAAACATGAAAT

AGGACTTCTAAAGTTATGTCGTCAATCTTTATAATAGTCAAGATCTGTAACATTGACTTAACAGATGTCTGCCCTATGCC

AGTCATATTAACAATACTATTTAACCTTCTACCACTAAATTTAAGAGATGCCGATTCTTTAATTAATGAGCATTCCGCTC

CCGAATCAAAATAAAATGGAAAACGCTCACCTTTGTGGTAGAGGTGTCCAGCGGGTACATCTATATTGCACAGGTCGACC

CGCTTCTCCGTAGTTGAAGCTCCGCCGCTGCTGGTGCCTCCATCCTTGCTCCTCCGTTTCGGACAGCTGGATGCGTAGTG

ACCTTGTCCATGACAAGAGTAGCAGGTAATGGAGAGTTGTTCCGCAGCAGGCTTCTTATGGTTGTTGGAGAGAGTCTGGC

CTTTGTCTCCATTTGGCTTCAAATTCCTCCTCAAGAAGCACTGAGTGGACTTATGGCCGAGTTTCCCACAGTGAAAGCAC

TTGACGCTAACATGGTTTGTACCTGGGCGAATCCTCTTAAAATCTGGTGCGTCATTGTTTAGTGGGGCTTTTCTCTTTAA

AAACGACACCGCTGTAAGTTCTCGTTGTAGTCGTTTTCTATTATCGATGTCGGTGGTACAAGACAACCGTTGAATGCGAG

GCTCGAACTGCGAAATATGTGCCAAAACTGTGGCTACCGCAATCTGTTCTGTCGTCAAGTTTTTCCACCTTGACATAATA

GAAGTCATCAACGCAGCGCCATATGCAGCAAGACATTCATTTTCTTTTGGCCTGCTTCCAGCCATATTTATAAGGTAAGA

GGCCACAGTCTCAGGGCACGCATACCTTGCAGTGAAAAGCTCCTTAAAATTTTCCCAAGTCATCCCCGGAAACGACACAG

TAGACAGCCACGTCGACGCGTCTCCCTTCATTGCACGACTCAACGCAATCATTAATGGGGCGCCTTGTTGATATCCATCA

GTAATGCACGTGTCCGCGGTTGCAATCCAAGCACGTGCGTCCACATTGCGTCGGTCCGGATCAAAATCAGGAAGACGAAG

TTCGTTCGGAGATCGCGACTGCTTCACGGCCTCTAGCATGGCCAAAAAGTTTTTATTTTGTTGTTCTAATAACAACAATA

ACTTTTCTTCACTCTCACATTTATTTTCAACACGTGGGGCGTTCCGGCTACATCTCACTTCTGATGACGGGAGTTCAGGA

GTGGATTCGTTATCAGACATGATGATCGTTGAAATAAAAGCGTAGTTATTCGCTTTGCCGTTGTATTTTCAATATTAAAT

TACAAAAGTAATAAAAGAATTAAAGATCAAATCGATGGACCACGATTTAGTTGGCAACGCAGCGAGTTCATCCACTCGAA

TGTCGGCGCACGCGGTCCGCCTGTCATCCGGCGTTCCTTGGCATCTGTCAACGTCATCCGACACCGGGGTGACCACGCTC

AAGCTACCTGCTTGACATTGCCAGCTAATTCGTAATAATTAAAGAAATGAAAATAAAATCACAATTAATTCTGACGCTCC

GACACGGCCATCGTGACATCTCACACGTCCTCGTGTGATGTACCTGAAATGAAGAAAAAGTTCTGCAGTATTCAAGTTTC

CACTCGGCCAGCCTGGCCTAAATAAGAACTCAGAATATTCTTTATTATTTTATTTCATACAGAAACAACTAAACTTCTCG

TGTAAATTAAAATTATAACAGCAATCAGAATACAAGCAATCAAATTCAAAATACCTACAGTTCAGAGTACAAATAACAAA

ACAATAACCGCAATTTAATTTTGATGATAGTACACATGGACATTTAATTCTGGCGTCCGAACAAGACATGGACAACCTCA

CACAAAGTTGCTACACCAGGATAACTGAACCGGTGTCCGAACAAGCCATGGACAACCTCACAGCACCCGACAATACCAGG

ATAACCAGATCAGTGTCCGAACAAGACATGGACAACATTCTTCCAGCATCCGAAAACACATGAATAACCGTGCTAGCGTC

CGAACATGCCCTGGACAACCATTCCACGGTATAACTTATCACTAACTAACAACTTTATCAAGAGCTAGTAGATGGCACAG

CACAGTCATCGTCAGAGTCATGATCGTCTGTAGGCCCAGGCATCCTACGAAGTCTATCATGAGCATACTTGTATTTCCTA

TTTGAATTCAAACCTTGTAATACGTAACGGTCACCTTCCAGAACCTCTATTACTTTGAAGGGGCCCTTATACTTAGGATC

TAACTTGGTTTGGTTTCTTTCCTCATTTTGGAGTAAAACGTAATCACCGATAGGGAACCTGTTGACCTTGGCCTTAGTAC

TGTCAAACCGTGTCTTATCATATTGAGCATTTTTAGAAATATTCTCTATCGCGTGTTCACGTACGGCGTTGAGATCCACT

TCTGGCTCTATATCACTAGCTAACAAAATGTCGACAGGCCTAGCAACTTTACCAATGAGCAACTCGAGAGGACTCGACCC

TGTGACCCTATGTTGTGAACAGTTTAATGCTAGTTGTACATCTGGCAAGGCTTCCTGCCACGACCGCTTACTGGTCTCAG

CGGCAGTAAGCATGGCCTTTAGAGTGCTCATTGTACGTTCGACCTGCCCGTTTGCTCGAGAGCTACCTGTTGCAATTAGG

TGTAGTTTAATGTTCGCAGAATCGCAATACTCTTTAAAATCTTTGCTGGCGAAGCATCTGCCCTGGTCGGCGATTATTCG

TGTAGGTACGCCGAATAAGGAGATGCTAGCTTTAACCGCCTTTATACTACTAGCCGAATCGATGTGTAACGTATGGTGGA

GTAAGACAAACTTAGTGAAGGCGTCGATAACAACAAAAACATACTCCTTACTATCACTTTTTCCACTAAGCTTTCCCGTG

GCATCTATGTGTAAAGTATGCCAAGGGATCGCTACCTTTGGGATGGGGTGCAACTCGGCTTGAATTTTGCCAGAATGCGA

CTTCGAAATTCTGCATGTAATGCAATTGTTAACAAACTTTCTCACGTACTTGGTCATTTTATCAAACCAGTAGAGCTCGT

ACACTTTCTCTAAGGTCTTCTCCCATCCCAAATGCATTAATCCCTCATGCACACTATTAATAACTGACCACCTCAAAGAC

CGGGGTATGATGGGTAAGAAGCGAGACTTACCTCGACGCTGTATTTTACGATATAAGACACCCGATCTGAACTCGTAAGT

CTTTGCAATATCGTCAGGCAACTTCTTGTCTTTAAGGTCACGCACTAACTTGCTGATTTCTTCGTCACGCTGTTGTTCTG

CTAACAGCCAATTACTAGTCAGCTCAGTAATATTAATTTGCTTTGACTGTACCTGTGGCTGATGCATGTGTGCTTCTTGG

GCTGGAAGAGGGTTTCGTGAGAAGAAGTCCGCATGTGCCATACGTCTACCATCAACGTGTACTACATCAAAGTCGAAACC

CTGAAGGAATGCCCACCATCTGTGGACTCGAGGCGTCAGATCCAACTTTTTGCGAGTTGACTGAAGGGAGTTGCAGTCGG

TAACTACAGTAAACTTACGGCCATGTAGGTAATGGCGGAAATGCTTAACCGCGTTAACAACCGCTAGAGTCTCCAATTCG

TATGAATGATATTTAGACTCTGCGGCTGTCGTACGCTTACTAAAATAAGCAACTGCATGCAGCTTACCTGCTTTTCTCTG

CATGAGCACCGCGCCGTACCCAACCGCACTTGCGTCAGTATGCAGTTCCGTTGGTAGATCTGGATCGTAAATCGTAAGAA

CTGGCTCCCGAGTTAGCTTGGAAATTATTTGTTGTCTAACCGCCTCATGCTCCGACTTCCAATCTAACTTACCATTAGAG

CTCGAGGTCAACGCGTACAACGGTGCCATTGTCTGTGAGAATCCGGCGACAAATTGCCGAAAATAGGAAGCCAATCCAAT

AAATTGACGAAGCTGCGTTACCGTCTCTGGTGGTGACAGTGCCGTGAGAGCATCAATCTTACGTGGGTTTGGGCGAATCT

CTCCCTGACATACCTCGAAGCCTAGGTACTCCACTTTTTGTTTTAAGAAGGCACATTTTTTAATGTTCAATGAAAACCCT

TGATCTGTGAGGACTTTGAGTACGACTTTTAAACGCTCGAGAGCTTCCTCTACCGTACTCGCTACTATAAGAACATCGTC

CATATAAACCACTACGTACGTATTAACTAGTTCGCCAAGTGCTTTAATAATTGCGCGTTGAAAAACTGCTGGGGCATTTT

TTAATCCGAACGGCATGGTTAAGTATTCGTATTGGCCCTCGGTAGTAACAAACGCAGTACGCTCAATAGAGTCAGGCTCT

ATGGGGATCTGATGGAACCCTGACGCCAAGTCGCAAATTGTGAAAAAGTTGCCTTTATTCAGTCTTGCTATTTGATCCGA

GATCAGTGGTAGCGGATATCTATCAGGAACAGTATTGTCATTCAGTTCCCGATAGTCGACACACATGCGATCAGTTCCAT

CCTTCTTTTTAACAAGAAGGATAGGACTGGCAAAAGGTGA

>TRINITY_DN12763_c0_g1_i2_BEL13_AG#LTR/Pao429-5717[Anophelesgenus]

GCTACGACCGCGTGGAACAGATGCGGCAGCAGTTTTGGCAGAGGTGGTCAAAGGAGTACATCGCGGAGCTTCAGACAAGA

ACGAAGTGGAAGACACGCAGAGAAGAATTGGTACCTAATACTCTTGTACTAATCAAGGAGGACAATCTGCCGCCTCTCAA

GTGGCGCTTGGGAAGAATCCTTCATACTTTTCCAGGGAAGGATGGCCTGTCACGAGTCGCTGACATCAAAACGGCGACAG

GGACGGTACGAAGGGCATTCTCTAAAATATGTCCTTTATTATCGCAAGAAGAAGAAAAAACTACGAGTTCACAATAATCA

TGCAGTTATCAGTACTGTACTTCGTTGGAAGCAGGAGCTTCCAAGGCCGGGGGCATATGTTAACGCTACTGCTAACGCCA

TCTAGTGGAAAAGGATTAAGATACGAGACACCCTCTATCGTCCGGCGGACGGAGACGCGGCGAATGATTGTGAGCGGCGA

ACGAGACCGGCGTGCAGCGAGCAGCGAGCTCTGATTGGTCGCCGAGGAACTTCAATTATTCGCTCCTATCGATCACTTCT

TTTTGACATCCTTCAATCAGCAACTGCGGTGGTAGTCGGTACACCACCGTAACATATTATTCGCCTTACATAATATTTTT

CTGAATGTACTGTACTCATCATAATAAAGTATTTTTATAAGAAGCCTAATAAAGAAAAATTCATTGAAGAGTGACCTGAT

CCTCACACACAACACACAACATAAATTGGACCTTCGAACCGGATCGGAGCGGCTTCAAGAAGAACACGTGGAAGAAGCCG

GCAACCTGCATACGAAGAAAACGGTCAGGCCATTCAACGTTCCTTTCCAAACCCTTCCCTTAGCATCATTTGAGCTAATA

TCGTGTTTAAATTTCTAGTAACCGAGGCGCGGCGCGTCGTCGCGAGACCGCGTCTTGAAGTACCTACTTGCCGCCATTTT

GTGCAGCGTGATTTTCAAGCTCGACCGGGTTAAAAACTGTAAGTACTTTTCTTTTGTTTCTTGCAGTCTCCCAAAATGCC

AATAGACGATTATAAAGATTTGGTTAGGAAACGGGGTGTAGTTAAAGGAAAGTTTACTGGATTCGTTAAATATGTAGAAT

CTTTACAAAACTTAGAATTAACTGAATCTCATAAGATTGCTCTAGAAGATCGATGTAAGAATGCTGTTTCACTGTTAGAA

CAATTCTCAGGCATTCAAGATGACATAGAATTTCACCTACAGGATTCTGAATTAGAAAGTGAAAAACAATTAATTGAGCG

AGAAACTTTTGAAAATTTATTTTACGATTTCACATCTAAAGCGAAATGCATGCTAAAGAGTCAAGAAATTATTGATAACG

ATGTTAAAGTATGTAATAAGACGTCTAGTATTAAATTACCCACTATTACATTGCCTTCCTTTGACGGTTCATTTGAACAG

TGGTTAGAGTTTAGAGATACCTACATTTCCTTGATTCATAATTCTAAAACTATAGATAATATTCAAAAATTTCATTACTT

ACGTTCCGCGCTCACTGGTTCTGCTAGTCAAATAATAAAATCGTTGCAGTTTACCGCTGAAAATTACGCGATAGCATGGA

GTTTATTGGAGAACAGATTTAATAATAATAGATTACTCGTTCAAAACCATGTTAAAGCTCTGTTCTCATTACAGTCATTA

CACAAAGAATCTGCATCTCATATTCGTAAATTGATAGATAACGTACTAAAGAATATTCGCGCTCTTAAAGGTCTAGATGA

GCCCACGGATCAATGGGATACGTTAATCATATTCCTTATAGTTACTAAGATGGACTCAGCCACCGAGAAGGAGTGGGAGA

ACCATAAAAGTGCACTCTCTAAGTTACCAAGTCACGCTAACAAAAAATTAACATTAAACGATATGATTACATTTCTTACC

GATAGGGCGGACATGTTAGAATCCATAAAAAGTACACATGAGTCTAGTAAGCAAACGTCAGAGGGATTCGTAAAGAAAAC

ATTATCCCATCAATCTAAGCATGTTAGCTCACATAGCTACACCGCAACGCGTAATAATAATAATAATTCGTATAATAAAT

TATCGCAAAAATATAATTCTTACAGTGATTCCAAACGTCAGCATAGGTGTAATATGTGCAAAGAAAATCATCCACTTTAT

TCGTGCAGTAAGTTCAGTGACCTTCCTATGAAGGACAAGTTATTATTCGTAGACGAAAACAAGTTGTGTAAAAATTGTTT

GCGTGGTGGACATGCAGTGTCTCATTGCTGGTTCGGCCCTTGCAAGCTATGCAATCGTAAACATAATTCGCTTCTTCATG

GTGAGTGCTGTGATAACAGCAATACGTCAAAGTCTGTAGGTCCGCAGGACGCGCAAGCGCTAGCGTCCGCAACGTGTGGC

GAGTCCGAAGCGACGACAACGTCCAGTGTACACAGTACCGCGCTACACAGTGCGTCAACTTCAACGCGTTATCAAGTTGA

AAATGGGTATTTATTCCAAGAGCCCGTAATTCTTTCGACTGCATTATTAGATATTTTAGACAGTGATAACAATTATCACA

CTATTCGTGCAATTTTAGATAATGGCAGTCAACATTCGTTCATTAAAAAATCGTTATGCGATAAATTAAAAATTCGTTTG

ATACAGTCCACAGTACAAATCGCAGGGGTAGGTAATTCGGTAACCCAATCTACACAATCTTGCAAAGTGCAGGTGCGATC

TAAAAATCAAGAATTTAATACGCATTTAAATTGTCTCGTCCTTCCTTGTATTACTGCAACGTTGCCTTCTGTTGCTCATA

ATGTCGCAAAATTGATGATTCCGAGTAATGTATCACTCGCCGATCCTACATTTTATATGTCAAAGAATATCGATTTATTA

ATCGGCGCTGATAGATTTTGGGATCTTTTAAGTGAAGGATTAATTCGCCTTACAAACGGCCCATACCTACAAAATACTAA

GTTAGGTTGGGTTGTATCTGGTGTCGTACACACAAAAGGTTCATCCGTTAATCGAGTTCAATGCAATTTTACGCACACGT

ACGACAACGCAGATATTCAATTAACGAAGTTTTGGGAATTAGAGGAGATAAGTTCACAAACTTCGAACATGTCTAAAGAA

GATAGTATTTGTGAAAATTTATTTATAAAAACAACTACACGTGATAATGACGGTCGTTTCTCTGTGCGGATCCCATTAAG

CGAATCAGCTGATCAACTAGGCGACTCGTACTCGCAAGCCGAGAGTCGGTTCCTCGCGCTTGAACGTAAGTTAGGTCGCT

GCACACCGAGTTATAAGAAATTATACTTTGATTTTATGCGTGAATATTTAGAACTCGGTCATATGACAAAAATAGATACT

TATCCGTCTCCACATTATTTCTTACCGCATCACGGTGTATTTAGAGAAAATAGTACGACTACAAAATTACGAGTTGTGTT

TGACGCTAGTGCTAAAACTACTTCCGGTCAATCGCTAAATGATATCCAAATGATTGGTCCGTCTTTACAAAATGATATTT

TTTCCATTCTTTTAAGATTTCGTCAGTACACATATATTGCCTGTGCAGATGTCGAAAAAATGTACAGACAAATACTAATT

CAACCCGATCAACAATATTTACAATTAATTTTATGGAGAGAAAAACCGCTCGATGCACTCGGTGTATACCGCCTTAACAC

AGTTACCTACGGCACGGCGTCCGCGCCATACCTAAGCATGCGCTGTTTGCAACAACTTGCGTCGGAATGTACTGATAACG

ACATCGCTACTATTATTTACAAAGATTTTTACGTAGACGATTTAATTACGGGTTGTGATGATAAAAGGGAATTATTAGAC

ATTTGTAACAAAGTTTCTAATGTGCTAAAATCGGGTTGTTTCTATTTACGAAAATGGTTATTCAATTTCGACTTGTCAAC

TTTGAATTTAAACGCATCAAAGAATTTATCTCTAGGCGAGTCTTGTCAAAGTAAAACTTTAGGTTTAGGTTGGTTTGCCA

ACACCGATGAATTATATTATACGACTAAATTTAAATTTACCGAGAATCACGTTACTAAGCGCATTATGTTATCGGCAATC

TCACAAGTTTACGATCCATTAGGTTTATTAGCACCTATTGTAATACTTGCAAAAATATTATTACAAAAATTATGGACTTA

TAAAATCGGATGGGACGATCCGGTACCTCACGATATTTTAATTAGTTGGAAAAAATTCGTTGATACTCTTGATTATTTGG

ATAAATTACGTATACCACGTCAGGTTATAAATAGTAAAATGCAATATATAGAATTGCACATATTTACGGACGCCTCTCAG

AATGCGTACGGCGCCTGTGCCTACGTCCGTATGTACTCGGGTAACAATAACAAGGTGTCCGTAGCCTTATTATGCGCTAA

GTCGAAGGTCGCGCCCGTCAAACCTATAACTATACCGCGTTTAGAATTGTGCGGAGCATTATTAGGCGCAAAGTTGTACA

CTAAAATTATTAATTCACTTCGTTCGAATTTCACTAATGTTTATTTTTGGTCGGATTCCACAATTGTGCTAGGGTGGCTA

AGAATGTCACCTAATACTCTTAAAACATTCGTACAAAATAGGGTAGCTCAAATTAATGAGCTCACGGGTAGTTTACCATG

GTTACACGTAGGAAGTAAATCCAACCCAGCTGACTTGGTGTCACGCGGGGTCACGCTGGACGCACTGCGCACAGCTGATA

TTTGGTGGCGCGGCCCGGCATTTTTACACGAACGATTGTGTAATTTTAGTTATGATTATAGTTGTCATAATGTTAATTTT

AATGAGTTGCCCGATACTAAATTAACGACCACACTGTTAGTACATCAAGTTAATAAGAATACATTTCCATTTGAGAGATT

TTCTTCCTTTATTCGTATGAAACGTGTATTTGCTTACATGTTACGTTTCATTGATAACGTTCGTCATAAAATAAAGTTAA

ATCGTAAATATGGCCCGCTTCAAGTAGATGAGTTAAACAAATCTATTATTAAATTAACTCGAATGTCACAAATTCAGTCG

TTTCCTGATCTTGATATATCTTCAAAGGGGATCTTAAAGGGTAATCGTAATATATCTGGTCTTAATATATTTGTTGACAA

TAATAACATTATTAGAGTAGGAGGTAGGCTAGATAATTCTTTAAGTTTTTCATACAATAAAAAGCACCCCATTTTGTTAT

GTAGTAAACATACTTTTACACGTTTATTATTTCAATATGAGCATATACGCCAACTACATGTAGGTCCTCAACAACTGTTA

GCTGGTATACGAGAAGAATGGTGGCCCCTCAGAGGTCGTAATCTGGCCAGAAATGTTGTACATACATGTGTGACATGTAC

ACGTATTAAAGGTAAAACTATGTCAGTACAAATGGGTAACTTACCTATAGAAAGGCTAGAACCAGGTTATCCATTTATAA

GGTGCGGTGTAGATTATGCAGGACCCATGTTTATTTTGAACCGTAAAGGAAGGGGCGCTAAATTAGAAAAATGTTATATT

TGTTTATTTATATGTTTCTCTACGCGTGCAGTTCACTTGGAGTTGGTGACTGCTTTGACAACTGATGCATATATATTAGC

ATTAAAACGCTTTATGTCTAGACGGGGTAAACCCTCTGAAATATTCTCAGACAACGGTAAAAATTTTGTTGGAGCTATGA

AAGAACTTACACATTTTTTAAGGAATAATTGTGATAATATAATTGAAGTTTTAGCTAATGATGATATAAAATTTAATTTC

ATACCACCATATACACCGCATTTTGGGGGATTGTGGGAGGCAGGAGTTAAGTCTTGCAAGTACCACATACGTCGTGTTAT

AGGCAACGCGAACTTAACTTATGAAGAGTTTAGCACGATATTGGCTCAAATCGAAGCCGTCCTAAACTCTCGACCCATGT

ATCCACTGTCCGCAGACCCAGCTGACCTTCTTCCCCTCAGCCCTTCTCTGTTTCTGATTGGCCGACCGCTCACCGCACCG

GCCTGCCCAGACCTGACGACTACAGCTTCACATCGCCTGTCTTGCTACGACCGCGTGGAACAGATGCGGCAGCAGTTTTG

GCAGAGGTGGTCAAAGGAGTACATCGCGGAGCTTCAGACAAGAACGAAGTGGAAGACACGCAGAGAAGAATTGGTACCTA

ATACTCTTGTACTAATCAAGGAGGACAATCTGCCGCCTCTCAAGTGGCGCTTGGGAAGAATCCTTCATACTTTTCCAGGG

AAGGATGGCCTGTCACGAGTCGCTGACATCAAAACGGCGACAGGGACGGTACGAAGGGCATTCTCTAAAATATGTCCTTT

ATTATCGCAAGAAGAAGAAAAAACTACGAGTTCACAATAATCATGCAGTTATCAGTACT

>TRINITY_DN12705_c0_g2_i1_CR1-5_NVi_pol#LINE/L21509-4384viatfastywithCR1-73_HM_pol,1frameshift.[Nasoniavitripennis]

TGTGTGTGTGTGTGTGTGGTGTGTGTGTGTGTGTGTGTGTGTTAGTTTATACAGTTATTTTGGTATGGTTAAAAGGTTTT

CTGTGTCATCGTAGTTACATGATAATAGATAAGAAGTGAGAGTTTGCTTGCACTTTAATTTAGTCATAGGGTATATATTT

AATATTGAGTTCATTATGTTATACAAATATGATCCAAGAAAAATGTGATGGCGATGCGAAAGTTCATACTTGCATAGTTT

AGTTGTACATACTTTTGATTTTTTACGTTTATGCGTATTTAAGTTAGGGTTGTAAGTCTGAGATGAGTGTTTTCGTAACA

CAGTATGCAAGATAAAAAGTTGACGGACTGTTAGAACTTTGCACTTTTCGTATAATTTTTCAGTGGGGTATAGAAACGGT

AAGGAATAAGCTGTTTTAAGTATAGCTCTTTGTGCGCGTTCAACTTTGATCATAGATGTCTTGTGTGATCCACCCCACGC

CGTAATGCAATATGTTATAACTGATTGGCATAGCGCAAATTATACCGATTTGACAACCTTCGCATCAGCAACATGTTGTA

GGTTCTTAAATATATAAATAAGCTTTCTTATTCTCTTAGCTAGCAGTTCTATATGCGCACTAAAGTTTAGGTTTTTGTCA

ATTATAATACCAAGGTATTTAATGGAATCGACCTTTTGGATATTTGGACAAGAGCAGGAAGCGTGGGTGTGGTTGCATGA

GTGGTGTATTATGTTGAGAGATGTGATGTTAGTATGGTGTGAGTTTGCTGGAGAAAAAGTAATGTATTTAGTTTTGTCTG

TGTTCAGAGTGAGTAAGTTATTATAGAGCCAAGTTGTTACTTTAGTGAATCCTATCTGTGCATAATTGTAGACCTCTTCC

CAAGTATCACCGTAGAAAAGAAGTGCAGTGTCGTCCGCAAAGGTATATATACGGCCATTTCTGAGAGAGATTTTGCAGAG

GTCGTTTATAAAGATTAAGAAGAGGGTAGGACCCAAAACACTACCCTGCGGAACGCCGATATTAACCGCACAATCATCAC

TCATCCATTTATCGACTACAACACGTTGAACTCTACCATTTAGGTAACAGTGAAATAACTTAAGTTGAGTACCACGAATT

CCAAGGTGTTCCAGTTTATTCAGCAAGAGTGGTGCAGAGACTGTGTCAAATGCTTTGGCTAAATCAAGAAATATTGTTAT

AACTTTTTTCTTTTCCTCAAGTTTATGTACTATTGTGTCCGTGAGTTGATTTACGGCGTCGTTAGTCGATCTACCACTCC

GAAATCCATATTGATTGTTTGATAGTAATTTGTTTGATTCTAGATATTTAATAAGTTGTTTGTTAATCACTTTTTCTAAT

ATTTTGGATAATGTCGGCAATATTGAAATTGGGCGGTAGTTATTGACACAATCCCTACTACCACTCTTGTGAATCGGATG

GATGAGTGATTTTTTCAAACTATTGGGAAACTCACCTGTATACAGACAACGGTTACATATATGTGCAATAGGATCTGATA

ATTGTTTGGCATATCTTTTTATTACATTACTAGGAATATCGTCACATCTCATTGAAGAGGAAGGTTTAAGACCAGTTATA

ATAGTTATGATCTCGTGAGCATCAGTCGGTAGTAGAGCTAGCGACGAGAGGCAAGGTTGAAAGGGAGTTTGGTTAGTGAT

AGGGTCAGAGTTAGGACGCAATATATTATCTGCTAATGACTTGCCTATACTAACAAAGAATGTGTTTATGTCGTTAATAG

ATTTAACTGGATTTTTTGTGTCTATTAACTCTATGGGCGACGTATTTACTTTGGTAGTGGAGGTGATAGTTTTTATGGTA

TTCCATAGTCTTTTTGTGTTGCCTTCACACTTTGCAAGCTCAGTTTTGTCATATTCTTGTTTAACCTTTTTTAGTATGTC

ATTACAGAAATTACGGTAACGCTTGTAGATGACTTGAGCATTAGTATCCTTTGGATGTTTTTTCGATTTGAGGTGCAAAT

TGTCTCGATTTCGCATGCAGCGCATTAAACCTGGCGTAATCCATGGCTTAATTATTCGCTTTCGCCTCGAAACATTAATG

ATTTTACTACTACTACTAATCGCAGTATTTAGCGTATTGAGAAAACAGCATGTGGCATCATTAGGGTTGTCTAACAGATA

TATACTATTAAAATCTATATTTCTAATTTTGACATCTAGAGCATTGTAATCAATGCGTTTGATTGTTTCTTTACGCCGAT

GTGGGGCTGGTTTAGTTAGGAGACTTAATATTACACTATAGTGGTCAGTTAATGTTGTGTGCGCTACTAAACATTTTACA

GAAAGTTTAGTTTTTGTAATGACATGGTCAAGACATGTTAAGGTATGTGTAGGTATGTAATGTGCTGGAAGCAAGCCATG

TGATGCTAAAAATTTTAGGAAAGGTTCCGTTCTACTATCTGAGGTGGATGAAGAAATATCTATATTTATGTCACCAGTAA

GAAAAATATTTTTGTAGTGTTTAAGGCTAAGTAAAATATTATTCAAGGAATCCAAAAATTTAGAGATATTCCTTGTACTT

GGGGGGCGATAAATGCCAAGCATAACTGTTTGAGTATTAATTTTAATTATTAAACAATTTGCATCCTCTATGGTGGGTTC

GGTTATACTGAAACTGTGGGAAAGCTTGTAATAAAATAAAAACAATTAGAACATTCCCCCTCTTTGCAAGGGCTGTTTGA

AGTGTTGTCTGAAGGTCTCGTGTATAATGTCATCTGTGCTACATTTGACGTACAGCCCACTTGACGTAGACCTATAGTTA

GGGGGCGCATGGTTATGTTCCAGTTTAAAACTCGTAATTCTCCCATTATCATCAAACTTGACACTGGACTTACAACTCTT

GTACTTTCTCGA

>TRINITY_DN12709_c1_g3_i1_Proto2-1_BM#LINE/Proto217-1009Fragment.[Bombyxmori]

AGCAACACCAAAGTCGGATGTCATATTGATGGAATTTGTATAAATAACATTAGCTATGCAGACGACATGGTGTTGCTGAG

CCCGTCGATCGGCGCACTGAGGCGACTGCTCGCCATTTGTGAGTCATACGCATTGAAACATGGTCTCAAATATAATGGTA

TGAAGAGTGAATTCATGGTGTTTAAGGCAGGAAATGTAACGTACTCTGATGTTCCTGATGTGTTGCTTTCTGGCTCACCA

CTGCGTAGGGTGAACAAATTTAAATACTTGGGGCACTGGGTCACAGAAGATTTAAAAGATAACTGTGACATTGACAGGGA

ACGCAGGTCGCTGGCTGTTCGCTGTAACATGTTGGCTCGCAGGTTTGCAAGGTGTACAAAACCAGTTAAAATAACGCTCT

TCAAAGCCTACTGCCAATCTTTTTACACATGCAGCCTGTGGGCCAATTACACTCAGCGAGCATACAGTGACCTGCGTGTC

CAATATAATAATGCACTGAGAGTACTGTTGGGGCTACCATGGCGTTGTAGTGCGTCAGGGATGTTCGCAGAAACAGGTGT

TGACGGCTTCACAGCCATCATGAGAAAACGTTGTGCTTCCCTGATGGCTCGTATGGGTGCTAGTGCCAACACTATACTCA

GTGTATTTGCTGAGCGGTGGGACTCCCCAATGTTAAAACGCTGGGTCCACCTACATGTTCAATAAATGAATGGCTTATGT

ATTGTGACTTATATATTATGATTATTATTGTGTTTAAATTAATGTAATATAACGAATGTAATGTACCTATATTTTTTATA

ATGATTTTAATATGTACCTACTAACACTAGATATTAAGTTAACATTGTTACTAACACTATGGACATGGTCTGAAATAAAT

GATTTGATTGATTGATTGATTGATTGAATATACTTTACATATACATACTAAACACTATCTATATAATAATATCACTAAAA

GAAACAGACACTTAGTAAATCTGACAATGATAACCGAGTTTAG

>TRINITY_DN12726_c0_g2_i1_PiggyBac-5_HM#DNA/PiggyBac484-2212viatfastywithPiggyBac-5_SM,1frameshift.[Hydramagnipapillata]

TTTTTTTTTTCATATAAACATCTTTTTTTATTCCAACTTATAACCATACATAATATACACAGTACAAAAAAAAACTTCCT

AATACAGCAATAAGGACAAAAAGAAATTGTCACTATCGTTTTGTAACATAACCAAATTGAGAAATCTAGTAATAGGATAA

ACTTATCTACATGCCGTGTTGTAATGTACAATGCTTCGAGCGAATACGTTTATTTATTTCAATACAATAACTTAAAAGTA

TACAATTCTTAATATGTACTCGATGGAATTCGAATTAAAATTTACACAATACATACATATCACTATAGCTACAATTAAGC

TGTAAATAACCTGAAGAGTTTTATTTGTATTTCAATTAAGGAACATAAAACATATCCACATCTCAACGATATCACCATAA

TCAGACTAATTTACATTGCACATACTCCGATCGCGACCGTCGGACTAAATCACATTTCGAGTACACTTACACACATAACT

GGTACACAATCGACTCGGACGCTTAACAATGGCATCTTATACACGAAACGGCGCACAGCGCGTATCATACTCGCTCCCAA

TCTTACGTTAAATGCGGAACTTAGCAATTAATGTTACGTTCGAGGTTGAGATCAAATGCGGGAGGGCCGCTCGTCTTCAC

TTGGCTTCGGCGAGCGCCTCCTCTAGGCAGCACAGAGAATCCTTTACCGCGTGGTACACAATATTCTTAACCCTTAATTC

GACGGAGTTTTTTGTAGTTAAAAAAGCCGGTTTTTTTAGTAAAGTTATATTTATGGACATGAAATAAGTTCAGGAAAAAT

GCTAAAAAAAATTACTGCTGAACAGTTTTTGATAAAACACATGTCCGTCACAGCGGACATTGTCAAGTGTGTAACGAATC

ACTTGCGAAACAACATGTCCGTTGGGGCGGACTCTATCAAATGACTAACAGAAATGATAAATAAAACAAATACACATCAA

AAGCAAAAAAAAATGTAATTCTTATCAAATATTACTTTAACCATGCAATTCTAATCAAATATTAATCAAATAATTCTAAT

CAAATTTCACACTAAATAATCATTATTATTTTGTATGAAATTTTAAATGGCAATTACGAGGGTTCTTTCCCGTAACGAAA

CATACCCTAACGTCACATTTAATGCAGAAGACAGTAGTCTTTTTAGTGCACTCCATGCAACGACCTTCTTCTTTGGTCGA

TAAAAAATGACCAATATTGTCAAATCGCACACAATCTACTGGTCTGGGCTTACAAGGCTTCCTTATCGTGGTCTCTTCTT

GGTTAGTGCGCTTAGCTGACTTGTTCATTTTGATCAAAGCAGAAAATATCTCGTATCGGAAAAGTTTTAATTTAAGCCTT

GCTTCAGATTTTCTTTGGGAGTGGTGCTTCCTGTACAAAATCCAGGCATTATTGATACAGATATCAATTAATTGAGCAAA

TATGCCTATGTACCAACGACGTGTCTTGAACGGCGTTTTATAAAGCGAGACCAGCATGTCGGCTAGGTCGACACCACCCA

TATGCTTGTTATAGTCCTTTACTACGTTAGGACAATCCACGTCCACTTTCCGCTTAGCATCCTTGGAGTACCGTTTTATC

TTCTGAACAGGTTCGGAGTCGGTATAGGAACTGATCAATGTGACACATTTATTGTCATTCCACCTTACCACGGCAAGCTT

GTTGTTATTGCAAACAACTTGTGAATAGCTACCTCGCTCCTTTTTTTTCAGCATTTTTTCACTGGTAAGTACATTCTCAG

CTCCTCTCAATCTGTTATTGCGTACAGTTCCTAATGCAAAAATACCATAATTTTGGCGCAGTATATAAAGCACTTCTGGC

GATGTAAAAAAGTTGTCGAAGAAAACCATAGCGGGCCTCCTTTTAATACTTTGGCATAAAGCTAATACTATTTGAGCACC

AAAACCCAAGGTCTCTTCTTCTTCAGAAAATCTGTGAAACCTAAATGTGTCTTCCCCGCCATACAAGACGAAATCATATA

TCATTCCTGATATACCAGCACGGACATAGTTTTTAAAGCCCCACTTATTGGGCTTGTCCTTCATATATTGGCGCCTTTTG

CCAGCTTTTGTGCCTTTGTATGCAATCATCATTTCATCAATGCTAAAACGTCTTTCGGTCTCTTGAGCAAGGCAATTATT

TCGTATTTTCTCTACTATTGGTCGAATCTTATAATACCTGTCCCCGTCGTCATAATTGTTGTCCGCAAAATGAATATAAC

GTCGTATTTTTTCATATCGTTTCAGACTCATTAGATTAGCTATAAGTGGATATCTAAATTTCGTGCTCCAGTAATCTATA

TAAGAAGGCATACCAACAATACCCATCAAAATATGTATAGCTAAAAAATCGCAAAATTCTTCCTCAGTAAGCTGAATAGA

TTTGCCTGTACATTGGGTGGAATAAATATTTGTTTCACTCACAATATGTTCAATGATATCACGGGAAAAGAATTTGTAGA

AATATTCAAGGGGTGATTCAATTTCTTGTATGTTAAGATCTTCAAAGTCGTCCTCTAAGGTCTCCACTCTGTGTTGAAAT

GGCACCTTCTTCCATTTGTATGTCATACTGAACTTCTTCTTTGGTTTCTTGGCCTTTTTGGCTACTACAACAGGCACTTT

AGATCTTCTCTTTCTTCTAACTGTAACTGCTACTGATGGAGGAGTTGGTAACGATGGTAGAGATGACATTGAAGGGAGAT

TACTGTAGACTGGTTCTTGCGATGGACTAGGGAAAACTTCTTGCAATATTGAACTAAAACCGGCAGGTAAGGGTTCATCA

TCTCTTTCACGTTCATTTTCGTCACTGTCTAATAAATTAAGAAGTTCCAAAGAAGAAGCTATTGAAGGTGCAGGAGATGA

AAAATATGAATCTGGTGAAGGTGGAACATGCAGTTCTTCATCAGAATCTGATTTATTACTAGCTTCAGATGGGGCACGAT

GTCTTGGTACTAAAGCTAATATCTTGTTAATGCGCCCATGCAAATGCTTCTGTAAAAGCACAAAACAAAAAAACTAAGCA

TACACACCCACACCAAGCATTTATAGAAGAAATGTATTCAACCATATGCGATATTATGCAAAAAAAACAATAAATGTTAC

AATAAAGTGTTAATCGTAGTATTTGGCAATGTTCGTCACACCGGACAACTAGTTAAACATATACTATACGTAGTAATCGA

CAGTGTCCGGTGTGGCGGACAAGAGATTTAGAGCGAGATAAAATAGTGAAAATAAGCGTAGAAGCTCGTGATTATCTTAT

TTTCCAGATGTATATAATATTATAAACATAAATTAAAACAGAAATCGGGTAGAAGTTAGTAAAAACCAATAAAAATTAAA

TAAAATACCTTGAGCAAAACAGAAGACTTCGGCGTCGACGCCATTTTCGACGAAAACTGACATTTTGGGTGTTGCCATTA

TTCAAATTATATTTTTTACTAAAATTTTTATATATTACCAACACACTAAAATCAAAAGCTCACGTATTTTTCTCTAAACT

AGTTCAGTAGATTTTTTTAAAGTAGTTGTTCGTCACACTGGACACTATCGAATTAAGGGTTAATCTGTAGCTTATCTTCT

TT

>TRINITY_DN12707_c0_g2_i2_Harbinger-1_NV_tp#DNA/PIF-Harbinger203-1563,ViagenewisewithHarbinger2_DR_tp,1intron(753:1081)[Nematostellavectensis]

CAAATAAGGAAAATAATGTAACTAATTTAAATAAATATTTTTTGTAAATCTAAATGATTAATAGAAATAAAAGCTTTGAT

TTTCATTATCTATTTATTGTATTATGAATACATACAACCTCTTACACCCTAACACTATCTCTATTCACTATGGGACAATA

CAAACATGTCATATCTAATTTAAATACCTAGTCATATTTACTTATTTTCATACAAGAATAAAGTAAAAAAATAAATTCAA

ACAATAATTGTAATAAGATATTAGACCAATAATCAAAAATTCTTCATTTGTTACATATTTCCTGGTTCATACTCTCTCTA

TTGTGTGCAGGCCTGTGGCATCTCATATCAAGTTGTTAAAATATTGTGTGATTAGATTATCTCTTTCTGTAGTACCAGTC

TGATTCGAACTTTCATTATAACTTGGTGAAGGTACATCTGCATCTAGAATTTCCACTTCTGGTGGTATGTCACTGAGGCT

TCTCAACCGGCAAATATTGTGGAGTACTGCTGTTGCTATGATCACTGCATTGGCTCTTGAGAGGCTTAGGCGCATTGTCA

AGGCTACAATAGGAAATCTTCGTTTCCAAACTCCAAAACATCTTTCAATACAGTTCCTAGTACGAATATGGGCTTCATTA

TAAAGTTGTTGACTCTGTGTTTGGGGATTTAACAAAGGTGTTAAAAGGTATGATTTTAGTGGATATGCACTATCTCCAAG

TAACCATTTATTGCCAAATAAGCCACTTTCACATTGAGCTCGCAGTTCAGAATTATTAAATATTGTTGCATCATGTGCTG

AACCAGGCCAACGTGCCACAACATTCATAAACAATAAATTAGCATCACATACAGCTTGCACATTAATGCTAAAGTAGCCT

TTTCTGTTTCTGAATTCTTCTCCAATTTGAGAACATGGTGATTGAATTCTAATATGTGTACCATCAAGAGCTCCAAGAAC

ACGTGGAAAGCGTGATATATTATAAAAATCCTGCTCAGTGTTTGGGAGCATGCGAATATATGTGTTATATAGCCGTGCTA

TAGCACAGCTAACATCAAATACGATACGACAAGCTGATGTTTTCGACACACCTATAAAATCAGCTACTGATAATAACATT

GTTCCTAGTGCATAAAAACGTAAAGTAAGCAGCAACTGATGTATTGGTGGTATTCCATGGTTTCGTCTTCCTTTCACTCT

TAAATTAGGATAAATTTCACTGAGTAATTGTTCCACAGCGGGTTTGTCGAGCCGAAATCTCAGCTGAAATTCTTGGTTAT

CAAGCACGGAAAAATAATCAATCCTTTGTTTATAAATTCTTAATCCCACTTCTTGATCCGTATCTTCGTCTG

>TRINITY_DN12734_c1_g2_i1_45238703_Tni#DNA/hAT-Tol2gi|45238703|emb|CAE54614.1|putativetransposase[Tetraodonnigroviridis]

GTCATTTATAAAAAAAGGGGTAACAAAAAGTAAGGATTGTACAATTATGAAATTTTACCAGAAAATGGTTTTACCTCAGC

GTAAAGCCAGTGTGAATTTAATCTGAACTGACGACACTTTCCAACCTGAGCAACTGCATACTCTGGAAATTATATTTCAA

AAATATTCTCTGTAGCTTATTTGGTTCGTCATCACCAAAGTCAGGCATCAAATTTACTATGTCTTGGAAATTGAAGAGCT

CATTCAAGGGCTTAGCTTTCGCCAAAAAGGTATTGTATTCAAAGAATATTTTTTTTATTTCTGGGTAACTATTTAAGGTG

GTTATATCTTCCCGTCCGTCCCTTAAATAATTTTCCATTATTGACTTAGCTTCTGTTAAGAGGGAGTTTGCCGATGCCGA

TTGTTCCCTACTTAGTTTATTAGAAATTAGAATCGCAAATCTTTGGTGTATTACTCGCCTATACGACTCAGGGTAAAAGT

CCATCCAATTATCTTTATACATAGGACACGAAATTGCAGCTATGGCAGCGTGTTGTGCGATGGGTCCCTTCCCTGAGATA

ATGGAATCAAATCTGCTTAATATACTCCTTCTAAATAATGCTGCCAAATGTTTCCCGTAGAGTAAATCACTTTGTTCTAA

ATCTGCTAAAGTTGTTATCAAGGCTGTAAGCCGTGGCAGAACATTGCCGTAATAATTATTATTTGCACCTAATAATTCAA

CTGTTTCCATTATTGGCATAGCGCATTCTAAATGTTCTTCTATGTATTGGAAATTTTTATCGGTCATGACTTCATGCTGA

AATCCTAAGGCATTGTGCAATTCTAGGTACTTTCCTCTGATGGATAATATTTGCATTAATGCTTCATAAAGGCTAAACAA

GCTGTATGAATTTGGCCGCATCAAATTTTGGCCTAAAACCCGATGGAATATTTCAGTATTTTTATTACACCCTGCTGCAC

GCCACAGGTGGCAACACTTATCCATGACCGTAGTATGATGCTCAATAATTACACAAGGGGCATTTTTCTTAATATCATGG

GTTAAAGTCAATCTTAATTTACGGCTACTGCAGGTAACATCGTCAAGCCGGGCATAAGAATTATATTCAGTAAGGTATTC

GTCCCGGACCCACATCTGCATGTTGCCGATCGACTCTGTGTCATCAGGTTGTTCTAGGATGTAATACATCAACGCAGCCT

CTCCACATTCATAGGAATCAATAGAAAGGCCGTAAGCCTGATATCCCCGTACAATACTTCTGAAGTCGTCGAGAATTGGT

TCAGCATTATTTTTATCAGCATTCAACAATCCAAATTGTGTATATACCTGTTTTGCATACGCTCTCAAATTATTCACGTC

GCAATCGGACTCTCCAACATCCTTTAAAGCCAATGCTACTGAAACCCGATGAAAATCTTCCGTTATCCAATGCGCAGTCA

CGCATAGAAAATAATATCTGGCATTTTTCCACAAGTCCATAGACGTACATACATAGTCAGTATTGATTATTATATTTTTT

AATTCTTTTAGTTGACGTTCATATTCTTTATTTAAATCACGTTTTATATGCCGATATTCTAGCTGATACAAATATGGTTT

AGGTATGTTCATATAATGCATGAAATTTACAAACTGTGGGTCGATAACATATTGCAAAGGTATCAGAGTATTGATGATAT

AATTTAAGCAAAGGTCATTAAATTCTTCCTTAGTAATATTCTGCATTTCATGCGAATTCTTAACCTCGATGTGGTACTTT

TTCTTTTTGTGTTGTTGATACTGATAGGCATAATCATCAACATCATAATCATCTGCATGTTCTTTTTTCAAGCAGTTTGC

ACATTGCACCGTGACCTCATGCGCAGTACCCTCTTGTGGCAACTCGTGACGCGGAATTATAGCCACCGTCTTCGAACTAC

TGCAGCCTGGGGTAGCCAGAGTTGTTTTTGTGCGTTTTGCTTTACTTGAGGTACTACAGCCTGGGATTGCCGGCGACTTT

CCCTTGTCAATTCGATTGGATGAGGTACTACGGCCTGAAATTGCCGGAGATTTTTCTGTATCCATTTGATTGGCCGATGT

GCTCCTGCCTGCAAATTCTAGAGCTTTGCCTTTGTGCCTTTGATTGACCGAGGTTACATTGCTTTCATAGGCCCAGGAAC

TGCAACCTGGAATTGCCAGAGATTTTTTCGAGTCACCGTCCGTGTTCCAGGTGCTGCAGCCTGCAATTGCCAGAGATTGA

TCCGAGTCACCGTCTTGTGCCCAGTTGCTGCAGCCTGGAATGGCCACAGATGTTCCTACTTGCCTTTGATTGCCAGAGGT

GGTGCAGCCCGGAATTGACAGAGATGTTTCTGTCGCTATTTGATTGGCCGACGTTCGAATTGTCACAGATTGTTTTTTGT

CCCATTGACTGGCTGTAACACTGACGCCTGGGATTGCCAGAGTTTCTTCTTTGTGCAATTGATCTACCAAGATAGTGCTG

TCTGGGATCGCCAACCCCTCCTCTGGGAGAACATCAGGTACAAGAGGACCACAATATTCTGATTCCAATATTTCAACATA

TGCCCCCACATTATCATCATCGTCCATCTTATTATATTGACGCACTAGTTTTATAATATTAAATCACTTAATTGATAATT

ATACGCAACACTTACTTCCGCTTTGTTGTCGAATGTCACCACACAAAACTTTATTCTTGGTTCTACTAACAACTACGCTA

CTGCGCGGCCTACGCCTTCCACTGATACTGGTGCATTAACGTCGCCACCGGCCGATAGCTTACAGATGCGGGGCGTGCGG

GAGGCTGCGGCGGACTGCGTATTACTTACTGCGCAGGCTGCACCCCCGTCCGCACCTGCGCGCTCGATGCACCTTGTGCC

TATGTTCACACTAATACAGATACTTAGCCGTGGTATGAGTTGACCTTCTTTTTTAAATAATTTTAACTTTGTAAGTAGGT

ACCTTTAACGGTAATTTTAGTCAATTCTTAA

>TRINITY_DN12755_c0_g2_i2_PiggyBac-1_SM#DNA/PiggyBac476-2245[Schmidtea]

TTTTTCTGCTATTATTTATTCGTATAATAGGACACTTATTAACATTATTAATATTAATTAAAAAATATGTAATCATAAGT

TACGCGAAATTCGGTGGTAATCATTTTACCATTGCCTGTTTACGTTTATGTCTTGGTGGTAATTAATTGACCTTAGCCCG

TTAAACTACTAGAAGCAGAGCTGCGACAGTCCATAACTTCTTCTTCATCTGGAGCAATGTGAAAGCTTTTGAAGCATGGT

GTGGTACATAAAGCTACTTTACAGACACCGCAAATGATCCGTGATCGTTTTTGAGTATTTTTTGTGCTGCAAAATCGGCA

TCTAGTAAATTTACCTTGATCTTCTGGGTAGTGTGGCACAGAAGAGCACCTGATTTCAGCGGGTACCCCGAAATTTCCGC

CTTGTTTGTTCCTAAAAATAATACTAGTGGCTCTTTTCTTTACTGTGAATCCATCTATAAGACTGCGAGCCAAACGCAGT

CTGAAATCACGATGTGTATGAACTTTTTGCTTGTGTATTTGAGTGTAGGCTATGTACGCGTTTATGACAGCTGCATCGAA

CATGAAATAAAATAGGCGCAACCAGTTGCGTCTCGATTTTCGGCCGATTGGATACGAGGCTCGGAAGTGATCAAAATGAT

CCACACCTCCCATGTTCACAGTATATTCAGCAATAGCAGTGGGGATAGTAATATCCTTCTTGGAACCATCTTTCTGTCTT

CGTTTGACGGTAGACACAGATTTAGGATTGTGTGCTGTTGTCAAAATACTCACTAGTTGAGTATCCATCCACTGTATAGC

AGAAACTGGACTTTTGGTTTTATACATAAACTCGTGCTTTTCAAGTTTTTCCTTTTTGAGTTTATTTTTGGGCAACAACT

TTTCTGGTAAGCCTTTTCTGTTCAAGCGCACGGTACCAACGGCATATATTCCTTTCTTTATCAGCAAATCATTCATAAGC

GGAAGGCCAGTAAAGAAGTTGTCGAAAGCTACAAGAGTATTGCGTGGAACACCTTGACATAGTTTCATTATGACATTATA

TCCTAAGCCTTCCTCACTTGATGCATTTTCGTCTGCTTTACCAACATATATATCGAAAAAATACATGTATCCAGTCTTAG

CATCACACCGAGCCCATACTTTGTAGCCCCTTTTGATGGGCTTTTTGGGCATGTACTGTTTGAGTGTGCTTTTACCCTTG

AATTTTACCATACTTTCGTCTACCGATTGATTGCTTGATTGGGAACATTGAGTCTGGAATATTTCGTTTAGTTTCGTTAT

CATAGGCCGTATTTTATAAAGCTTATCATAATCTGGCGTATCTCTTGCGGGTTCTTTAGTATTATCATTCAAGTGAATGT

TTTCAGTCAGTTTTTTGAATCTTTTTAGAGTGAAAGTTTGGGCAATATCAGGATTTCTGTAAAAAGGATCCGTTGACCAA

TACAGATTGACATCAGGTAAGGGGTGAAGACCCATCATGATTAGGACACCAAGATATGCACGCATTTCGGCTGGAAATGT

TGGATTCCAGTGAAGTGTTTGCCTTTGTGCTGCATAAAGGTTTGTTTGTTCTACTAGCAACTCTACCAGAGCATGAGGGA

AGTAATTGTCGAAATATTCAATAGGCGATGTTTTATGTGTGACGCGCATGTTTACAGTCAGCTTTCTATCAAACTTTGGT

ATGCTATGGTTTGGAATCTTATCTGACCAGTTACGAGCCACACAAATAGGTCTTCTAGATGTTATGTCTGCGCTTACCGC

CTCATTAGGCTGCGCTAAGTTAGACTGAGCTGAAGTTGAGCAACTTGGTTCAGAGAGAGCATCCATAACGACGTTTGAAT

CATAGTCAATTAGCGTACTTACTTCCCTCACCGGTGTTTCAGTTACTGGTTCAAAGCTTTCCAGAAATTCTACAGAAACA

CGGGGTCCTGGAGGATCTTCAAAATTCTGCAATATTCTCGGCTCCAACTCAGGCTCTTCACCAAATATTCTTGAAAGACC

TCGCGAAACTTCATCGTCGTAATCCAGTACGCTATTGGCTAAAATTGGCTCCTCCGAGTCACTTTCGTCACTACTTTCAA

TATCGGAGAGATCATCTTCTTGAATACTGGCGATAATCTCAGACTCAGATAACTTCTTTTTGTTTGACATTCTCCGGAAT

CTTTTTAGTACAAAATTTCAATTTGTTGGCGCCAAACACCGGCGATTGTCAAAATATTATGATTTTTTTTTCTGAATATT

TGTATCTTGCAAACTTTGACAGTTAGCGTAGTTGCCAGTACTAATAAAAGTTTTATAGATGTTTTCGCATTAAAATTATA

GAATTTACGAAAGTGTGCCATAAACGACCCTTGCTCTGTGGTCAAAAAGCTAAGGTGGTAAGCAATTGTCCTTTGCCCTA

TTAAGGGATAAATTACATGTGATTTAGGACTTTTCCGGACAACCTGCTTATGAGCACATATATTATTAAGATTTCTTATA

ATATATAATAGGTAGAAATAAGGAATAAGTGTAGAGGAAACCCATCTGCCTATCACTGATTGTCAGTTAAAAGAATCATG

TATTGTCCCGAATTGGACACCTGAGGCAGGATGGGGATCTCTATGTTACGTACTATTAATGTAAACTGCGTTCTTAATTA

TTGACTGTTTTATTAGATTAGGCGACTGCCTCGTTTCCGATACCTTGTTGTTAGAAAATGTAACAAATATGTTTTCCGTA

TGACTTGATGGTAAAAGTACGAGTCAATAAATAAAGAGTGTGTGGCAGACCTACAGCTGAAGCCGTACCGCACGGAGGAG

TTCGTGCACAAGCTGTACTACGAGACATCGCGGTTCTCCGCCTTCGGTCACCAGTGGGTGGTGAAGGCCTTCGTCAACAA

GAACCAGCGCGACCCCACGCAAAGCTCGCAGCGAGAGATCTCTTACCAGTTGATCCTGAAGAGTAAGCCGGCGCAGGCGC

TGGGCGTGCAGTGGCTGGTGCTGCG

>TRINITY_DN12755_c0_g2_i3_PiggyBac-1_SM#DNA/PiggyBac476-2245[Schmidtea]

TTTTTCTGCTATTATTTATTCGTATAATAGGACACTTATTAACATTATTAATATTAATTAAAAAATATGTAATCATAAGT

TACGCGAAATTCGGTGGTAATCATTTTACCATTGCCTGTTTACGTTTATGTCTTGGTGGTAATTAATTGACCTTAGCCCG

TTAAACTACTAGAAGCAGAGCTGCGACAGTCCATAACTTCTTCTTCATCTGGAGCAATGTGAAAGCTTTTGAAGCATGGT

GTGGTACATAAAGCTACTTTACAGACACCGCAAATGATCCGTGATCGTTTTTGAGTATTTTTTGTGCTGCAAAATCGGCA

TCTAGTAAATTTACCTTGATCTTCTGGGTAGTGTGGCACAGAAGAGCACCTGATTTCAGCGGGTACCCCGAAATTTCCGC

CTTGTTTGTTCCTAAAAATAATACTAGTGGCTCTTTTCTTTACTGTGAATCCATCTATAAGACTGCGAGCCAAACGCAGT

CTGAAATCACGATGTGTATGAACTTTTTGCTTGTGTATTTGAGTGTAGGCTATGTACGCGTTTATGACAGCTGCATCGAA

CATGAAATAAAATAGGCGCAACCAGTTGCGTCTCGATTTTCGGCCGATTGGATACGAGGCTCGGAAGTGATCAAAATGAT

CCACACCTCCCATGTTCACAGTATATTCAGCAATAGCAGTGGGGATAGTAATATCCTTCTTGGAACCATCTTTCTGTCTT

CGTTTGACGGTAGACACAGATTTAGGATTGTGTGCTGTTGTCAAAATACTCACTAGTTGAGTATCCATCCACTGTATAGC

AGAAACTGGACTTTTGGTTTTATACATAAACTCGTGCTTTTCAAGTTTTTCCTTTTTGAGTTTATTTTTGGGCAACAACT

TTTCTGGTAAGCCTTTTCTGTTCAAGCGCACGGTACCAACGGCATATATTCCTTTCTTTATCAGCAAATCATTCATAAGC

GGAAGGCCAGTAAAGAAGTTGTCGAAAGCTACAAGAGTATTGCGTGGAACACCTTGACATAGTTTCATTATGACATTATA

TCCTAAGCCTTCCTCACTTGATGCATTTTCGTCTGCTTTACCAACATATATATCGAAAAAATACATGTATCCAGTCTTAG

CATCACACCGAGCCCATACTTTGTAGCCCCTTTTGATGGGCTTTTTGGGCATGTACTGTTTGAGTGTGCTTTTACCCTTG

AATTTTACCATACTTTCGTCTACCGATTGATTGCTTGATTGGGAACATTGAGTCTGGAATATTTCGTTTAGTTTCGTTAT

CATAGGCCGTATTTTATAAAGCTTATCATAATCTGGCGTATCTCTTGCGGGTTCTTTAGTATTATCATTCAAGTGAATGT

TTTCAGTCAGTTTTTTGAATCTTTTTAGAGTGAAAGTTTGGGCAATATCAGGATTTCTGTAAAAAGGATCCGTTGACCAA

TACAGATTGACATCAGGTAAGGGGTGAAGACCCATCATGATTAGGACACCAAGATATGCACGCATTTCGGCTGGAAATGT

TGGATTCCAGTGAAGTGTTTGCCTTTGTGCTGCATAAAGGTTTGTTTGTTCTACTAGCAACTCTACCAGAGCATGAGGGA

AGTAATTGTCGAAATATTCAATAGGCGATGTTTTATGTGTGACGCGCATGTTTACAGTCAGCTTTCTATCAAACTTTGGT

ATGCTATGGTTTGGAATCTTATCTGACCAGTTACGAGCCACACAAATAGGTCTTCTAGATGTTATGTCTGCGCTTACCGC

CTCATTAGGCTGCGCTAAGTTAGACTGAGCTGAAGTTGAGCAACTTGGTTCAGAGAGAGCATCCATAACGACGTTTGAAT

CATAGTCAATTAGCGTACTTACTTCCCTCACCGGTGTTTCAGTTACTGGTTCAAAGCTTTCCAGAAATTCTACAGAAACA

CGGGGTCCTGGAGGATCTTCAAAATTCTGCAATATTCTCGGCTCCAACTCAGGCTCTTCACCAAATATTCTTGAAAGACC

TCGCGAAACTTCATCGTCGTAATCCAGTACGCTATTGGCTAAAATTGGCTCCTCCGAGTCACTTTCGTCACTACTTTCAA

TATCGGAGAGATCATCTTCTTGAATACTGGCGATAATCTCAGACTCAGATAACTTCTTTTTGTTTGACATTCTCCGGAAT

CTTTTTAGTACAAAATTTCAATTTGTTGGCGCCAAACACCGGCGATTGTCAAAATATTATGATTTTTTTTTCTGAATATT

TGTATCTTGCAAACTTTGACAGTTAGCGTAGTTGCCAGTACTAATAAAAGTTTTATAGATGTTTTCGCATTAAAATTATA

GAATTTACGAAAGTGTGCCATAAACGACCCTTGCTCTGTGGTCAAAAAGCTAAGGTGGTAAGCAATTGTCCTTTGCCCTA

TTAAGGGATAAATTACATGTGATTTAGGACTTTTCCGGACAACCTGCTTATGAGCACATATATTATTAAGATTTCTTATA

ATATATAATAGGTAGAAATAAGGAATAAGTGTAGAGGAAACCCATCTGCCTATCACTGATTGTCAGTTAAAAGAATCATG

TATTGTCCCGAATTGGACACCTGAGGCAGGATGGGGATCTCTATGTTACGTACTATTAATGTAAACTGCGTTCTTAATTA

TTGACTGTTTTATTAGATTAGGCGACTGCCTCGTTTCCGATACCTTGTTGTTAGAAAATGTAACAAATATGTTTTCCGTA

TGACTTGATGGTAAAAGTACGAGTCAATAAATAAAGAGTGTGTGGCAGACCTACAGCTGAAGCCGTACCGCACGGAGGAG

TTCGTGCACAAGCTGTACTACGAGACATCGCGGTTCTCCGCCTTCGGTCACCAGTGGGTGGTGAAGGCCTTCGTCAACAA

GAACCAGCGCGACCCCACGCAAAGCTCGCAGCGAGAGATCTCTTACCAGCTCGCCCTGCAGTTGATCCTGAAGAGTAAGC

CGGCGCAGGCGCTGGGCGTGCAGTGGCTGGTGCTGCG

>TRINITY_DN26391_c0_g1_i1_DIRS-1_BM_pol#LTR/DIRS1522-3390viatfastywithDIRS-7_NV_pol,2frameshiftsand1stopcodon.[Bombyxmori]

ACATTCTCTGAACGATAGGATCACTGCTTATGATTCGTCCGTTAGGACAAGCCAAAGTGACAATTGTTGATTTATAAACT

AAAATAGTTTTGTAAGACAGTTTAAGAGTTAAGTGCAAGTATGCTAGGTACTTAGCGACTTGGGCACCAGATGGATTTGT

ATGGTTGAAATTATTATCAGTACACCATTTTAACCATTTTTTCCAAATAGCAGCATACATTTTCTTCGTAGAGGGTCGCC

AGCTAGAATTAAATAGGTCTATTTCGTTTTGAGACCAATTAGTTAATTCGGCACACCACCCTTCACTAGCCATGCCTCTA

GATACATCTGGTCCACGTGAACCGGAGGCAGATATGTCCGTGTATCTAGTAACTGCTGCTGAAGATTGAATATGGGTAAT

GGAGCGCTCACCGCTCTCCTCTTCAGGTCCGGCCGCCAAAACACTTTGGACCAGCGCGGTACTATTAGGATGTAGCGACC

CTTGCACAGGTTGAGGTGTGACAGAATTCGTGGAATTAGGTGTGGTGGCGGGAATACCCATGCTCGACGGTATGACCACC

TTCTGCTGAAGGCGTCGTGAAACTCTGCGTTCGAGTCTTTGCAATCTCGTGAGACGTAGCGAGCAAGCACGTGCGCGGTT

TTCGAGGCGAACAAGTCGATTTCGGGCATTCCGAACTTGCGAAATACCTCTTGACATGCTTCTTCCGTTAAGGTCCACTC

CCCGGGTAATTTGAGCCTTGACAGCATGTCCGCGTCTGTGTTTACCTGACCGGGCAGGTACTGAGAGACCAGGTGAATCT

TGTTCTCGTCCGCAATCTCGAATAACATGCACGTTAGCCGATAGAGGTTCTGTGATCGGAGACCCCCCTCGTTCCGCATG

TAACTCACCACACTCTTGTTGTCTGATTGGACTAGCACGGTTGAATGACGTAGCGATTGCTGACGATTGGTTAAGGCTTG

TATAACTGCCCACATTTCCTTTAAATTGCTGTGCCATTTCTTCTGTTCCGGGGACCATAATCCGTTCAGAGAAGCGTCGT

CTACTATGGCACCCCAGCCTCTGGCAGATGCGTCTGTCGTTACATAGTGTGAGACAGGATCTGGATGTATCGCTGTTCCG

GTTGATAGAGCTCGTAACCACCACATTAAGGTCGTGGACACTTCCGACTCGAGTGGGTGTTTTACGTATGCTTGGTGCTT

TGGAAGCCGATTGCAAGCTACTTGTAATGGGCGGCAATGAAGCCGACCTCTGGGTACAACGAAGGCTGCA

>TRINITY_DN5302_c0_g1_i1_L2-1a_Cis_gag#LINE/L2100-795[Cionasavignyi]

GATCTCAGCTGTCAGTTCTATGTCAATAACAATATTGAATAGGTTACCGACTAATTTATAATAATAATGTAATAAAAATG

AAGGTATCAACGAAAGGAAATAAAATAAGTGCAAGACGGTGGTTGAAACAAAAAGTTATATAAGAGTAAGCTATGATGCA

GCAGTAAAAAGAGGTGCAAAGCAGCAAAGTCAGAACGGATACTATAGATAAACATGCATGATTATAAAACTACTTAATTG

CGACGCTTTAACCTTCTTTGGAGCTTGTTGACGGGTGTAGCGGGCTCGGAAGACAATGGAGCAGGCTGAGGCGAAGTTGG

AACTGCCGGCTGCGCAAGGACATCGTTGCTCAGGGGATATTTAGAAAGAAGTTTTTGAAGTTCATCCATGCTTTCTAATT

TGTGACGAGACTTGTCAGGAGTGATGATTAATATCTTGCCCTCACTCGACCAGCAATTAGACATTCCAAAATGTTTGCGT

GCGGCAACGAAGGTTTGGTGTCGCATACGAGTCAAGAATTCAGATATAGTGTAACCGGTACCTTTAAGTGATTTCTTGGC

ATCCCAAACAAGTTGGCGATGCTCGGTGGTGAAAAACCTGACAAGTATTGGCCTGGAACGCTTGCCTGATCCTAACCGGT

GGCACACATGGATGTTCTCCCTGGAGAGCTCGTTCAACTTCAAGCTGTCATGGAATACTTGAGAGACAACATCAGGGACC

TTCTCATCTTTAGCTTCAGGGACTCCATGGATTAAAAGCACCTTGCGCCGCATAATAGTTTCATGCCTGTCAAAACCTTT

TGAAAGTAACTCCAATTGAGTTTTGAACTTTGAAAGAGCTTGCATAACAAAGCTTTTGAAGTCACTAAATTCTTGCGAGA

GTGAGGTTAGACTTGCAACTGGTGTTGAAGCAGCAGAGGAGGAGGAGCCAGCGGTAGCGGATGCCAACTTTGCTTCATAA

GCAGCCATCTTTGTAGTGAAGAGGGATTCCAACTCTTCCAGGTTCAGTGCTAGTTTAGCATCCATGGTGATGAAGTGCGG

TGTCAAAGGTGAAAGTTAAAGAGTAGCGTAAATGACTTGAAAACAGTTATTAGTGAGTAAAGGGGGGTCTATAAAGCAAC

TGGGCCGATCAAAACATTAAAATATTCTAGAATAAGATTAATAAAGAATAATAAATAGGAGCAAAAAAATAAAAAAAAAA

>TRINITY_DN13316_c0_g1_i1_CR1-1_BM_pol#LINE/CR1274-3162[Bombyxmori]

CTTACTGTTACAATATAATGTCTAGTCTGTTTCTGGATAAATTAACCATTTAAAATTATCAAAAAAGATAGAAAATACTT

AATTTTCTCAATATACTTTAAGTCCGATATTAATTATAGTATGTCAGCGTGACAGCCGAAATTATTTTATTTATTTTATT

TTATTTGGGGAGCTTACGGCTATATTTACACATTAGATCAACAACATAATACTAGCATATGCCAATTACAAGCTTCCACA

GATGGTAAGAAAACATTATAGGTGTTGTAAAAATTATACAGGTCACACGAAAAATTACTTACTTATAAAATTATAACAGT

ATTTTGGGTTCCTTAGATAATAGTCTAGTGACATAGTGGGGTTTACTACAAAACAGGTCTATAGTAGGGTTGCTTTGGTT

TAACTTATTAAATATGTGTATAGCTCTTGGAAAGTAACTATTTTTTCTGTAATTGGTGTGGCAGAATGGGGTATGGAGTA

AATTATGAGAGCGCAATCTCCTAGAAGGTGTGTTCAAATATAAGTTACTGAGGAGATCAGAACAATCAAGACCATTTTGA

GCGATTTTCAGAAGGAAACGGATATCAGCTATTTGTCTACGAAGATGAAGAGGCAAAAGATGGAGTCTAGTGCATTGTTG

GTCGTAATCTAAATATTGTAATTTAAATTTAAAGCTTAGATATCTTAAAAATCTTTTTTGGATTCGTTCAATAATATTGT

TATATATATAGTAACCTGGGTTCCAGACTTGACTAGCATACTCAAGGTGGCTACGCACATAAGCACAGTAAATTACTTTA

AGTGACTTCATATTTTTGAATGGCGAAGAGGCACGAAGGATGAAACCAAGCGTTTTGTGTGCTTTATCAACAATGTGATT

AACATGGTGATCAAATAGAAGTTTACTGTCGACTAGAACACCAAGATCTCTGACGACATTTACACGCTCAAGACATGCAC

CTTTCAATGTGTATCTAGAAAGGAAATTATGTCGCTTTCTCGTGAAGCTAGTGACGCTGCATTTAGTGACATTAAGATCC

AGTTTGTTTAGATTGCAGTAAGCATCTAACCGATTTAAATCTTCTTGAAGACATAAAACATCAGATACCTTATTAACCTC

TCGAAAAATTTTCATGTCATCTGCATACAAAAGGAAGTGAGAGTTCAAAAAGCACGAGCCAATGTCGTTCATGAATAAAA

CAAAGAGAAGAGGTCCTAGTAAAGAACCTTGGGGTACCCCGGAAGTAATGCTAACCCAATCAGATGTACATCCGTTCATA

GCAACGGCCTGTGTCCTGGCTGTAACATAGGAAACAAACCATCTGTAAAGATCACCATGTATACCCAAGGCAGATAGTTT

TCTAAGTAGTATATTATGATCTAATCTATCAAATGCCTTACTGTAATCTGTGTACACAGCGTCAATTAGATTGCCTCTGT

CCATATTAACAGTTATATAATCCAAGAAAGTCACTAAATTAGTTGATGTTGATCTGGCTCTAACAAAACCATGCTGCTGA

GGAATTATTATATTTTTGAAGGATGCATACATTTGATCAAAGACTAGACGCTCAAAAATTTTAGCCAAAAGACAGAGTTT

TGAAATAGGCCTATAGTTGGTAACTTCATTTCTTGCCCCTTTTTTATGGACTGGTGTTATCAAGGCTGATTTCCATAACT

TAGGCATCACTCCATCATTCAAGGAACGCCTAAAAAGTATGGTAACCGGATGTGAAAGCTCGCGAGCACATTTAGCCAAA

AACAATGCCGGAAGAGTATCAGGACCAGCAGATTTATTAAGGTCAAGGGCTGAAAGCAATTTGGATACCCGATCCTTACT

TACCTCAATGTTAGATAAATCACAAGGAGTATCGAGTATCTTAACAGAGTGAGAACTCGGTGAGAATGTATCAGTAGCAG

GTAGGAAAGTAGAACTAAAATAAGCAGAAAAGAGTTGAGTGGTACCGCCACACGAATTGCTAGTACGAGATAAATAGGTC

ATAGTCGACGGATAACAGCTACTACCTCGCTTCTTCTTGATGTAAGACCAAAAAAGTTTGGGATTTTTGGAGATCGAGTC

TTCAACACTTATAATGTATTCCTTGTAACATTTATTTTCAGCTTTTTTAACCCTATCGCGGAGTAACCGGAAACTATAGT

CATCAGATATGTTACCATACGTTTTATATTTCTTTAAAAATTTAAATTTTTCTTTAATTATTTTGACAAGATAAGGCTTA

TACCAGGGAGGATATCTGTGACTAGGCGTAATACGTGTTGGTACAAATGTGTCCCTTAGTTTGAGTATAAGTTCATAAAA

AATCGAGACTGCTTCCTCCATCGACTTAGTGTTTGATAATATTGACCAATCGGTAGTAGATAGGTGGTTGCAGATTAGCT

CATAGTCAGCATGACGATACATGAATATTTTACGTCTAGGTATTTCCAATGAGTCTAATTCAATAAAAGTGGGCTTGATA

ATAAGAGATTTATGATATGCATCTTCAGGCACCAGCGGATCGGGGCATATCGACACCGACACAGTATTACTACATAAAAC

CAAATCCAAGATACCACCTGAATGATTAGTGTGGTTATTGTATTGCTTTAAGTTACAGTCATAGATGGTATCAACCAGAT

TATTAGCAGGTAGGCCAATAACATTTATCGGTTGCAGACCCATATCAGAAGGAATCCAGAAGATACTGCTCATATTAAAA

TCTCCGAGTACCAAAAATTCATCATGTTTAAAATTTTCAGTCGCGTATTGAAGTTTAGTTAAAAAGTTAGCCAACTGTAC

AGAAAAACTATTACCCATGTTTTGATTGCATAAGTATATGGTACAAATATGAAGTTTAATCGGTTTTTTACTACGCGCAT

TACGTAATGTTAAAGTGATCCAGATATCTTCAGCTGAAGAATTCCAACTAAAGTTTGGAGTGCATGCAAAATTACGGTGT

ACTGCAATTAAAACCCCACCTCCCCTGGTCTGTTGAGTCATCGCGTAATCTCTGTCGCGACGGAATACTAGGTACTTGTC

ATTAAAAAGTTCACTATCCGATATATCACTCAACAACCAAGTTTCTGTCAGCGAAATAATATCATAATTGGACATACATA

AGTTCCGATAAAAGTCATTGGTCTTAGTCCTGAGTCCTCTGCAATTCTGATAGTAGATACTAGCCATTGGTGTAAAATAA

GTTACTGTATCTAAAGTAGGTATGTAAGTATAAAAGGTTGACGGTAAAAGACATAAGTGTAGTCAAACCATAGCAAAACC

CCACAAACACATAAATATTGAAAACTTTACAAATAAGTCCAGCCAAAAATTGCATAATTCTTAGAAAATAAAGTATAGTA

CTAGAATATTTATTAAGATTAACAAGCATATCACATACGCAAATCGAAGTAAGCAATATGACTATCTTAATAATGACGGG

TAACCGAAGAAAAAAGGTGTAAACAATAATATAATTTTGTAGTAAACAACAAGTAGCGAGCTCGCCGCAAACAAAAGCAT

TCCATTGATGAGTACAAAGGATCACTAATGAGCTGTAAGTGATTCTCAAAGTTTTATGATAACAGTCGTGAATATTATAA

TAGGTAAATAGTAATGACGAACTGTATGCGAAAATTACATTATTTTGCCTAAGTCTAAATCGGTTTTTATTATGAAAGAT

TTTGTTTGAGTATTTTTGCGAACAAATATTTTGCAGTTTTTTACCCATACATATTCGTAGTCATGTTCCTTCGCCAGGTT

TTTTGCCTTGGTGAGAAGAATTTTATTTTGTACCGTTAAATGATCATTGATATAGATGCGCCCCTTATCTTCAAAGCCTA

TATCAGCCGAGCAAAGAGTTTTGAAGGATTTTGCAGCTGCAACGAAATCTTCTTTGACGTAACGATTCAGAAAGGATACG

ATTATAGGTTTCGTCCGGTTATCGCGCGCAGGAACTCTAGCAATGAAATTGATATTTTGTTTCACTATGGGATACATTAT

TTTGGATCCAATTTTAGAGACAATTTCAATCAAGTTTTCGCGATCTTTCATAGGAACACCTTTGATTTCAATGTTGTTTA

ATCGCGTCCATTGATTCTTTTCATTCAGGTCTTCTTCCAAAGTAGTAACTTGATTCTTCATTAACACAGCATCCTCTTGG

GCCTTCTCCACTTTGAGTAATCTTTGGTCAACGACTTGAAATTTCTCAGAATAGGAAACTAGCGTGGTCTTAATGTCAAC

TATTTCCTTTTTAATATCTTGGACACCAATGGTTAAGTCTTGAATCGGGGCAATAGTAAGTTTGATATTTTGTAGTTCTT

GCATTATCAACTCAAGTGACACAGGCTTCGGTGATTTGCATCCAGGACATTTCCAGTTAGCCTGGCGCTCAGCACCCAAC

CGGCGATATCCACTCTCAGTGACACCCGAACAGGCATAGTCGTAATATTTTTTACAGCCACTACACAAGACGGCGTTACT

TTCGGACACTTCGTCGTTGCATCTACCGCAACTAACCATTTTTAGCAGTAGCTATAAACAGGAAGTGTGACGGAGCAATT

AACTTTGAGAAACGCTGTTTCCTAGCAGAGCGAGACACGGGATACGTGTGACCGATAAGCGACGCAAACGCGAACTGATA

GATACAATCATAGATACAAACCTTCTAACGACATCCGTGGTCGAGTGGCG

>TRINITY_DN13302_c0_g2_i1_R1_pol#LINE/R11830-4985[Bombyxmori]

CTCTTCCGATCTTGGTTATCTGTCGTTCCGCACATTTCGTGGAAGAAGACGCTTCGTAGTCTTTGTCCAGAAATAATTTT

TATTTTATTTTTTATTTGTAACAGCGTGTTTGAAAATTTATAAGTGAACTTTTTATTCTCTACTATTACAAGATAATAGT

AGATTAAACTTGGTTTAATAGTGACAAATTGTGTTTGAACGCTCAACGAAATTGTGTCTGCCATTACTTATCCGTAACGG

GATAACAGTTTGGTTCTACGCCCTTTGTTCTAAACAAGTGTTAAATTAGCCAAGTGCATGCATCTTTCCCTGCAATACAC

AGGATTTTTGAACGAGGAGTGTGATGATACTATTATCGAATTATCCAAACTCTCGATCGCTGATCAGTCGCACGAGGTCA

CTGTATTAGACCCCTTCCATGATACTTCGAGCCGTTTAGCTACACCAAGGGATTACGTGTTGCGTCGATGCGGCCAGGCG

GACGCGATACTATTTGATGAATGCTATCCGGACACGGTGTTGAAAAACTGCCACAAAATCGGCGAAGGGGTGTACGGCGA

AGTGTTTTTGTGGCGTGCCAGAGATGGCCGTGCTCGCGTGCTGAAGATAGTGCCAATCGCCGGCACTCTCAAGGTCAATG

GGGAGAGTCAGAAGGACTTCCACGAAATCATATCTGAGATTGTGATTGCTATGGAATTAAGCGCACTACGCGCTCCCATA

GCCGAAATAAATCGCCACTTTGATGAAGGGAAGGACGTCGAGGCACTCAATCTACACGCTATAGAAAATGCAACTGATGT

ATTCAATGAGGTGCTAGCTGTGAAGTGCGTATACGGCAGCTACCCAGCTCGCTTGCTCGACCTGTGGGACCTTTATGACG

AGTGCAAAGGCTCTGAGAACGACAACCCAGCCATCCTACCAGCGGACCAGCAGTTCATAGTGTTGGAACTAGCCAACGCC

GGGCAAGACTTGGAGAGCTACCAGTTCAATAACGCTGAGCAGGCGTATGCTTTGTTCCTGCAGGTGGCGTATGGTCTGGC

AGTTGCCGAGGAAGCCTTCCAGTTTGAGCATAGAGATCTCCACTGGGGTAACGTGCTGATCGCACCAACTGATCAGAAGT

ACGCGACGTTCGTCCTGCGCGGTCGCGCGCTGCGCGTGGCCCGCTGCGGCGTGGCGGGCACGGTGATCGACTACTCGCTG

TCGCGCGTGTCGCTGCCGCTGGCGGCCGGCGAGTGCGCCGCGCTCTACAACGACCTGGCTGCCGACGACTCGCTCTTCGA

CGCAGTCGGCGACTACCAGTTCGAGGTCTACCGCCTCATGAGAGATAAGTTGGGGTGAGCTCGACTAATATTATTACACT

AAAAGCGAGGACTATTGAAAGCCTATCTTGGTTTTTTTAAATTGTACCAGCCGACGGAGAAATCAATGAACTATGTCTTC

AACATTACATTGACATCACTGTATTGTAATGATTTGCTCTAATCATATGCAAAGATTGTCCATAAAGTTGCATGAAGCTC

ATGGAAGGATTCACCAGCACCGGAGAGGAGACCAGGATTGGACTCCTCAGGACTCTGTGCCCCGACGATGACCCCACCAA

GGATTCAGCATACCACCGGCAGGTCAGAATAGCAGCGGCGCTCGCCCCTACAGGCAGGGACTCGGAGGCACCCAGTCGCG

AGGCGCTGGAGCGGATAATCAGGACACTGCCGAACACTGCACCGGGACTCGATGGACTCACTGCCCGAATCATCAAACAT

GCGTGGAAGGTCAGCGGAAGGGAGATGCTGTTTATGTACAGCGCGTGTTTGAGCGAGGGCGTGTTTCCGGACATTTGGAA

GATAGGGAGGCTAGTAGTACTGCCAAAGGGCAACGACAGACCGCTCTCGGACCCCAAGGCGTATAGACCGGTTACGTTAC

TTCCAATTTTGGGGAAGATTCTGGTACGCATAATTATATCGTGCGCTCCTTGCTTGTATAGGAACATCTCAGCAGCACAG

CATGGTTTTTCGCGTGGGAAATCTACAGTCACGGCGCTTAACGCGATACTGGAACGAGTTACGAGCACCGCGGAGAATTA

TGTTCAGTTAGTATTGTTAGACATCTCGGGGGCCTTCGACAACGCGTGGTGGCCCATGATTCTGGTCAAGGCTAAGCAAG

GCGGATGCCCGCCAAACATTTACAAAATTCTGGTAAGTTACTTTACCAACAGACGCACTGGAATGTTCATGGGCGGCCAG

GCGGTGTGGAAGATTTCCACTATGGGATGCCCCCAGGGATCCGTACTTGGCCCGACGCTCTGGAACCTCCTGCTGGATGA

CATCCTCAAACTACCGATGCCACTAGGCGTCAGTATGGTAGCATACGCTGACGACATTACGATCGTTATTGAAGCCCCAT

CACGGGCAGCGATTGAGAGAAATTCTCAGTCGGCCCTGGACGCGGTTTCCGCTTGGGGAAGGCGGAACCGCCTTAGCTTT

TCACCAGCTAAGTCTCAGACCCTTACCATGAAGGGTAAGTTCAAACGTCCCCCGACAGTACGCATGGAGGGTGCTTCGAT

CGCGCATGTCACGCACACCAGACTGTTAGGGGTTATTATTGACGATGCTAGCTCCTACGTGCAGCATGCTAATTTTACCG

GCCAAAAAGCCGCGAACTGCTTTGGGAAGGTGTCGAGAGTATCGGCATCGACCTGGGGCATCAGATACCGCGCCCTACGA

GTCCTGTTTTCAGGGACTTATGTGGCGACGCTCACTTATGCTGCTGCCGTATGGTGGCGACGCTCGTCCCATTATGCTGT

ACGTAGCGCATTATTGCGCACGCAGCGCCCGGCACTGGTGTTGCTCACGAAGGCCTATAGGTCGGTTAGCACGGCGGCAT

TACCCGTCCTTGCGGGGGTGCTTCCAGCTGACCTAGAGGTAACGCGTGCAGGACGAATCGAGCAGGAGGGTTTCCGGTTG

GGAAGGAAGGAGCGCAACGAGCTGAAACGCAGGGTGAGATCGGAGGTCGTTTCCCAATGGCAGGAGCGATGGGACTCCAG

CACGAACGGGAGGGAGCTTTACAACTTCCTTCCGGACGTGTCAGTGCGACTGTCTTGCGGCTGGGTGGAACCTGATTACG

TCACGTCGCAACTTTTGACCGGACATGGTTGTTTCCGAAAAAGACTCCACGCGATGTCACTATGTGACACGCCGACTTGT

TACTGTGGAAGTCCGGAGGAGACCAGGGATCACGTGCTGTGGCACTGCGAGCTTTACGCAGAAGAGAGGAGGCAGATGCT

CAATGACTGGTGCAGAGAAGAGATTGGACCAGTTTACCATCAAGAGATGGTGACCAGCAAGGAAGGCTTTAGAAGGCTGA

GCGCATTCGCTCATAAATGGCATAAGAAGCGAAAGGAACTGGAATCATAAAGAAGGAACTGTTCGAGCGCGAAGTACTGT

GGTTTGTCGTAACACGCCCCAGAATGGGGAGAAGTGAAATCAAGAAGGGACGGAAATAGAGAATTTGTAGGAGTCAAGAA

CCGCACCCAGGGAAAGAGCCAGTGGCAAGTACCGGGGTAGCGTCTCCGAGATTCGGTCCGTATGCCCAGGAGGGTAGGAA

GGACCATGAGGTGGAGGTGGGTTTGTCCCTATCTACTATCTAGCGAAACCACAGCCAAGGGAACGGGCTTGGGAGAATCA

GC

>TRINITY_DN13370_c1_g1_i1_TRAS3_BM_gag#LINE/R12207-3673[Bombyxmori]

AGAAAATCCGCACATTCCGCCCTCATATGGGGACCGCCGCAATGACTGCACAGATCCGCCGGGTCAGCGCAGAAGCGCTT

GCCGTGGCCGTAGCCTAGGCAGCGCGTGCACTGAACCAGCGGAGTCTGGTCCTCCACCCTTATGGGCTGCAGGTCAATCT

GCACCAACTTCCTCGCAATTGCCTTCTGCCAGATGATCGGAGACACGCTTATAACTGCATGGCACGTGTGGGGGTTTCGC

GCTCTTCGCCTGTAGCGGAATTCGACCCTGTCTTCGCTTTTGTCTAGGCCTTGGAAGAGGTCCTTGTTCTGATTCCTTAG

GGCCTTCAACATGTCAGCGTCTGTGTGACTGTTGAGCACTCCCCTCAGAACCAGAAGTGGGTCTCGGTTCTGGACCTCTT

CCACTGTTAGATGTTCTTTAACCCCTTGCAGCTTTTCTTTTAGTTTTTCTCGCTCTTCCTTGGAGCTCAGTCCGATAATT

ATTTTTCGGTCGCGGGCCTTCCTTACATTACGGACTTCAATCCACCCCTCTTTGGCTTCTACGGCGTCCCTTATTTTATC

CAGAACTTGTTCTCCAGAATCCTGGTCATCTGTAGAGGTGACCACAACTGAGTGGAGCGTTTCCCTTACTTTCGGCACTG

TGCCAATCTGGGCCGAAGTGTTTGCGGTGACGCTCGCGTACGTGCTTGTGGTCACCTTTTCCAGGAGTTCTTTCTGCGTC

TGTATGTCCTGCCGTAGTTCTGACATCATCTGAGTATTTTCCCGGAGTAGCTTCGCATGCTCCTCGATTTGAGTAGCCTG

CAGAGCCACCCCCGGAGTCTCCCCAGATGTTACCAATTTCGATGGTCT

>TRINITY_DN13341_c0_g1_i2_P-28_HM#DNA/P705-3561viatfastywithP1_Cis_tpandgenewisewithP-13_HM,4frameshifts,1stopcodonand1intron(1076:1142).[Hydramagnipapillata]

TTTTTTTTTTACTTGAAAAAGTGTATAATTAATAGCTCTAGGGAGATCAATACAAAATTGTTTGCAGTTGATTATATTTA

TCACAAATATAGCGACAACAATGTTTTATGACAATTCTAATATCATAAAATTAGTAATATACCTTTTATAATTTTGGTTG

TACAATGAAATTGATCTCATTGTTTTAAAACAATCTCACAGCACTTACTTTACATAAAGAACATACAGAAAAATATTCAT

ATAATTTTCACTTCTACATTTGTTGGAACAGAACATAAAAAAATAGATCGAAAATACCTCCACTGAAGCTATTATTACAA

ACCTATTCTTAATAACACAAGAAGCAATTTTAAATAACAACTTAACATACCTACAACTGTGTATACTTCATGGTACTTGA

ATGTGTGAAATACACTTAAACACATAAGTGAGTTTAAAGAACTTCATGTAGATTATTTGCTATTATGTAGGTGTAGAAAT

GTATACAGCTATCAAACAGATTTCAATTGCTCTCATTCGTAAAGGTATTGCCAATCAGTTATGCTATAAAACAACAACAT

AAGGCCCCTTGAATGGCTGGATCTTAGATTATATACCTATAGATGTAACCATGGCGTGACCGCACTTGAAACAGAAATAG

ATTTTTATGGTGTTGCCTAATAAGTTGGCAATTCCCACATATTAGGTAAAAAATATTATGAATTCTACTTGATCGCGAAC

AGACATTATAAGTACCTGTAAGTACTTCGCAAACTAAAAATTCTGAATTAGCACATTTTATATGCTAGCTTCAATAATGT

AACTCTTATGTCGTAGTCGTACGAAATACTTTAATAAAACCTCCTTGGTCTAATATTTCGTTTTTACAAGGGCGGTAAGG

AGAATCGTATGGACATCCATCGACCGACACGACATAAAACACACACGACACGTTCAATGATGAAATAAAACCAGTCTTTA

TCATAAAAATATTTATTACAAACCATAAGGAAATACCTACATTCATAATTATTGACCCTTAAACAATATTAACTTAGTGT

GCATATTTCTGATTCTTTCATCTCGATGAGATAATGACGTCATTATATAATGAATCCTGACTTCAAAATATCTAAAAGCG

ATAGCTTTAATTAATAATACAAAATGATTTTCAAAAGGACTTTGTTCATTAATATGATTCTTTATTTCTGCAAAGACTTC

TGTACCAATAAATTTTTTCAAAACACTGCAAGTCATTTTTGGTAAAGTGCCTTTTTGTGAAAAATAGGAATTCCCGTGTA

TTGATGTGGTTTGTCTTAGAATTTTTTCGGCAAACACACAAATTTTGATAACATCTTGTGATGGATAATGTAATCCGCCA

ACATCCTTTACAGCAATAAAACTCGGAAACTTTTCAATTGCAAATATTGCTCCTAAACATTCATCACATTTTATTATTTT

TTTCAAATTGCGCACTACATATCCAGAAATATAAGTTATGGCTTCCTTTGAAAATTCTGATAATTCTAAGTTTCCTATAT

AGTCATGACCCTCGGGCAAATCAACAATTGAATCTTCATCGAGACTTTCGTCATTCATAGCCAATATTCTTTCTCTTCTA

CTGTTTATTTTACGACAAATGGAATTTGAATTGCACGTCAAAATATTAATATTATCTAATGGTATGCAATTACCTGTGGT

AATATTTTTTAATTCACCATGGATCAGCAGTCGCTTATACGCCGATACGAACTGCAGAGCAGTAGGGTTATTGTTAAACC

CACCTTTAGAGCGAATGGCACTAAAGAAAACCTCCAAATGGTCTTGGCTCATTTTATAAGTACATAAATATCGAAGAGGA

GCATTTCCAATATGTATGTACTTGTAAAAAATAATTCTTAGCGCCTTAATAGAAATTAAAAACCCCAGAAACCCTGTTTT

TCTTCCCGAATTAATGACTAAATCACCTTCAATGAACCTGAGCGTTTTGAGATAAGTTTCTAACTCTTCTAATTTTGCAT

TCATCATATTTTCTTTACTGATAGGTTTTTTAAAACCATATGAATATAAACTTCGGCTATTTAGAATATCAAACAAATCA

TTAAAGTTTGTGATAAATTCAATAGTAGCTCCTGAATGTACAAAGTCTTCTATGGCTAGATAACCCTTGCATAATATAAG

AGCCTCGGCGACAGATTTGCTGAAAGTTTGCGCAGCGAGTCTAACTTTCATTATTTGTTTCTTGTAATTAATATGAGCAG

CTCTGAGCTTATTCCCTAAATGCAGTTCTTCCAGTTTTTGTAAATTATGCAATTTTTCAATATAATTCCAGGATATTATT

TCGCCATTAGCATTCAAAATCTCTTTTTTGTCGGCTAGTGTATTTCTAACAAGTTTTAACATGTGACATGCATCTGGTAT

TATTACTATGATTTGACCTTCAATAGTTATTGTTGGATTGATATTGTTAATGTCATTAAATTGAGCGCCTAGATTTTTTG

TCATGGCGAAATTTGCAGGACATCCGTCAAATGTCACCGATACTATTTGTACCCCACATTCTATTAACAAATGTACACAA

TTTTTTAATAATTCACTACGTTGGTCCCCGTTGACACCATTTGTCAGAAAGTACCCAACTGGTATTTTCCAACTGGAGTT

TAACGCCGTAACCATAAAGACCAAAGCATCCTTAGCTAGCGGCATACTATCGGTGTCTAGATTATGTCCTACATTCACGT

ATCCGACGGTGCGTTTGCCATCCCACTGTAGGTGTTGTCGGATTGACATTTCGTCAAGACTAACGCAGCATAATATTTTT

TCATTAGACTTACTAACTAATAATTTGATAGCTTCAAAAGCTTCACTTGTGAATCCAGGTTCACAATCCACACATTTATA

CCACCTATATAATGTTTTAGGATGGGGCAAGCATGTGTTAAATTGCTTTCTCACAAATGCATAGGCCTCAGGGCTTAAAA

AATGCAAACACAATGCAAATTTTCGTAACGATGGCGAATATTTTCGCGGACAGTTCGTTTTATCGAGGTATCTTTTAAGA

AAATCTGCATTTTCAGATTCAACATTTGACAGTAAATCTAATTCTTCAGATTGCATTTGTAGTTTGCATTTTAATTCTGT

TATAATATCTTGTAAATGTGCTGTCTTTTTAACTAATCGTCGTTTAGATTCACGTACAGCACGCAATTTTTTGCGACAAT

TATTAGATTTTGTCTTCTCTTTCTGCAGCAGATTTTCTAGTCGTTTTTCCTTCTTAAGAGTCGCTGGCTGGACAACAGGT

TCTACATCAACTTCATAGCTGGGACCGGCCTAAAATGAAATTAAGTAGATTTTATAGTTCTTCTATCAAATATTTGTAAG

TTTTACAGAGAATCAGTACGTCAGGTAAATATTGTTTCATTTTGCGCAGGTATGCTTACCACAGTTAACTTCTCTGGTTG

AATATTTTGCACTTCTCGTAAAATAACCAACTTTCTTCGTTTATTAGGAGGTGATAAAGATGAATTAAAATTCTCTTTGC

TCTCCTGGCTCGTAGATGGTAATGGAGTATTATCCTGTTGTGGACACGGATACGTTGAGGCTGGACTATGTATATATTTC

GTTGGTACTGAGTGAGGTTTTACTTGTACTCGTTTATTTTTATATAGTAATGAATCATCATC

>TRINITY_DN13341_c0_g1_i3_P-28_HM#DNA/P705-3561viatfastywithP1_Cis_tpandgenewisewithP-13_HM,4frameshifts,1stopcodonand1intron(1076:1142).[Hydramagnipapillata]

TTTTTTTTTTACTTGAAAAAGTGTATAATTAATAGCTCTAGGGAGATCAATACAAAATTGTTTGCAGTTGATTATATTTA

TCACAAATATAGCGACAACAATGTTTTATGACAATTCTAATATCATAAAATTAGTAATATACCTTTTATAATTTTGGTTG

TACAATGAAATTGATCTCATTGTTTTAAAACAATCTCACAGCACTTACTTTACATAAAGAACATACAGAAAAATATTCAT

ATAATTTTCACTTCTACATTTGTTGGAACAGAACATAAAAAAATAGATCGAAAATACCTCCACTGAAGCTATTATTACAA

ACCTATTCTTAATAACACAAGAAGCAATTTTAAATAACAACTTAACATACCTACAACTGTGTATACTTCATGGTACTTGA

ATGTGTGAAATACACTTAAACACATAAGTGAGTTTAAAGAACTTCATGTAGATTATTTGCTATTATGTAGGTGTAGAAAT

GTATACAGCTATCAAACAGATTTCAATTGCTCTCATTCGTAAAGGTATTGCCAATCAGTTATGCTATAAAACAACAACAT

AAGGCCCCTTGAATGGCTGGATCTTAGATTATATACCTATAGATGTAACCATGGCGTGACCGCACTTGAAACAGAAATAG

ATTTTTATGGTGTTGCCTAATAAGTTGGCAATTCCCACATATTAGGTAAAAAATATTATGAATTCTACTTGATCGCGAAC

AGACATTATAAGTACCTGTAAGTACTTCGCAAACTAAAAATTCTGAATTAGCACATTTTATATGCTAGCTTCAATAATGT

AACTCTTATGTCGTAGTCGTACGAAATACTTTAATAAAACCTCCTTGGTCTAATATTTCGTTTTTACAAGGGCGGTAAGG

AGAATCGTATGGACATCCATCGACCGACACGACATAAAACACACACGACACGTTCAATGATGAAATAAAACCAGTCTTTA

TCATAAAAATATTTATTACAAACCATAAGGAAATACCTACATTCATAATTATTGACCCTTAAACAATATTAACTTAGTGT

GCATATTTCTGATTCTTTCATCTCGATGAGATAATGACGTCATTATATAATGAATCCTGACTTCAAAATATCTAAAAGCG

ATAGCTTTAATTAATAATACAAAATGATTTTCAAAAGGACTTTGTTCATTAATATGATTCTTTATTTCTGCAAAGACTTC

TGTACCAATAAATTTTTTCAAAACACTGCAAGTCATTTTTGGTAAAGTGCCTTTTTGTGAAAAATAGGAATTCCCGTGTA

TTGATGTGGTTTGTCTTAGAATTTTTTCGGCAAACACACAAATTTTGATAACATCTTGTGATGGATAATGTAATCCGCCA

ACATCCTTTACAGCAATAAAACTCGGAAACTTTTCAATTGCAAATATTGCTCCTAAACATTCATCACATTTTATTATTTT

TTTCAAATTGCGCACTACATATCCAGAAATATAAGTTATGGCTTCCTTTGAAAATTCTGATAATTCTAAGTTTCCTATAT

AGTCATGACCCTCGGGCAAATCAACAATTGAATCTTCATCGAGACTTTCGTCATTCATAGCCAATATTCTTTCTCTTCTA

CTGTTTATTTTACGACAAATGGAATTTGAATTGCACGTCAAAATATTAATATTATCTAATGGTATGCAATTACCTGTGGT

AATATTTTTTAATTCACCATGGATCAGCAGTCGCTTATACGCCGATACGAACTGCAGAGCAGTAGGGTTATTGTTAAACC

CACCTTTAGAGCGAATGGCACTAAAGAAAACCTCCAAATGGTCTTGGCTCATTTTATAAGTACATAAATATCGAAGAGGA

GCATTTCCAATATGTATGTACTTGTAAAAAATAATTCTTAGCGCCTTAATAGAAATTAAAAACCCCAGAAACCCTGTTTT

TCTTCCCGAATTAATGACTAAATCACCTTCAATGAACCTGAGCGTTTTGAGATAAGTTTCTAACTCTTCTAATTTTGCAT

TCATCATATTTTCTTTACTGATAGGTTTTTTAAAACCATATGAATATAAACTTCGGCTATTTAGAATATCAAACAAATCA

TTAAAGTTTGTGATAAATTCAATAGTAGCTCCTGAATGTACAAAGTCTTCTATGGCTAGATAACCCTTGCATAATATAAG

AGCCTCGGCGACAGATTTGCTGAAAGTTTGCGCAGCGAGTCTAACTTTCATTATTTGTTTCTTGTAATTAATATGAGCAG

CTCTGAGCTTATTCCCTAAATGCAGTTCTTCCAGTTTTTGTAAATTATGCAATTTTTCAATATAATTCCAGGATATTATT

TCGCCATTAGCATTCAAAATCTCTTTTTTGTCGGCTAGTGTATTTCTAACAAGTTTTAACATGTGACATGCATCTGGTAT

TATTACTATGATTTGACCTTCAATAGTTATTGTTGGATTGATATTGTTAATGTCATTAAATTGAGCGCCTAGATTTTTTG

TCATGGCGAAATTTGCAGGACATCCGTCAAATGTCACCGATACTATTTGTACCCCACATTCTATTAACAAATGTACACAA

TTTTTTAATAATTCACTACGTTGGTCCCCGTTGACACCATTTGTCAGAAAGTACCCAACTGGTATTTTCCAACTGGAGTT

TAACGCCGTAACCATAAAGACCAAAGCATCCTTAGCTAGCGGCATACTATCGGTGTCTAGATTATGTCCTACATTCACGT

ATCCGACGGTGCGTTTGCCATCCCACTGTAGGTGTTGTCGGATTGACATTTCGTCAAGACTAACGCAGCATAATATTTTT

TCATTAGACTTACTAACTAATAATTTGATAGCTTCAAAAGCTTCACTTGTGAATCCAGGTTCACAATCCACACATTTATA

CCACCTATATAATGTTTTAGGATGGGGCAAGCATGTGTTAAATTGCTTTCTCACAAATGCATAGGCCTCAGGGCTTAAAA

AATGCAAACACAATGCAAATTTTCGTAACGATGGCGAATATTTTCGCGGACAGTTCGTTTTATCGAGGTATCTTTTAAGA

AAATCTGCATTTTCAGATTCAACATTTGACAGTAAATCTAATTCTTCAGATTGCATTTGTAGTTTGCATTTTAATTCTGT

TATAATATCTTGTAAATGTGCTGTCTTTTTAACTAATCGTCGTTTAGATTCACGTACAGCACGCAATTTTTTGCGACAAT

TATTAGATTTTGTCTTCTCTTTCTGCAGCAGATTTTCTAGTCGTTTTTCCTTCTTAAGAGTCGCTGGCTGGACAACAGGT

TCTACATCAACTTCATAGCTGGGACCGGCCACAGTTAACTTCTCTGGTTGAATATTTTGCACTTCTCGTAAAATAACCAA

CTTTCTTCGTTTATTAGGAGGTGATAAAGATGAATTAAAATTCTCTTTGCTCTCCTGGCTCGTAGATGGTAATGGAGTAT

TATCCTGTTGTGGACACGGATACGTTGAGGCTGGACTATGTATATATTTCGTTGGTACTGAGTGAGGTTTTACTTGTACT

CGTTTATTTTTATATAGTAATGAATCATCATC

>TRINITY_DN17279_c0_g1_i1_Crack-1_HM_gag#LINE/L286-808[Hydramagnipapillata]

GTGTGAACCGCTCGATTATGGCGGGGGGTCGCAACACCTGGGATGATTCATCGTTACTGCCAAATCGGCGAACACGGTGA

ATGGTGTCGACGTCACTCTCCAACAGCGCAAACCCAACGCGCAAACATAAGTCGCGGAGGATCGAGTATAGGTTTTCTCC

CTTTGTAAATGGTATGCCAGAAATCTCCAGGTTGTTAAGCATTGAAAATTGTTTGGCGTTATTACATTCCTTGATCAGGG

ACTTGATAGACTTGTCAGCATCTTTGTAGACCTTAGAAGTGTCGGACAGGACGAGGACTTCCTTTTCGGCAGTGTTCAAT

CTATCCTTAAGGGCTGACAAATCTGCCTTTATTATATTTTGTTCTTCAACCAAACTCTCGGACAGCGATGCGACCCTATT

TATCTGTTCCTTGATATCAGAGGTTATATCTTGACGCATTTTCTGGAGGTTGTCGTTGTGTGTCGTAGTGAACGTGGACA

GAAGAGACACAATATCCTTACGGAAATCAGATAATTGTTGACCAATTTCACAACTGTGATCTTGTATTTTACGCTTCCTT

AATGTCACCTGGGGATCATTGGTAAAAATAGTCGATAGATCCGGTTGAGATCCACCCATTGGTATCCGATCTTCAATAGA

TGAGGAGTCCATTTTTATATTTTCAGTTTGTGGGTAAAATAAACTAAGTTGGTTCCATATAGACCGGTTGGCAGCGGCGC

GGACACGGTGGCGTGATATACGCGGGAGCGTGACAGAGCTATTCAATTGTGGTTTATCCTTTTTTCTCAGCAATGACAAC

ACAAGACTGTCAGGAAGCAAAGGAGCGTAAGCACACGATCCACGTCAACTAAAAGTAAGCTCAGAGACGTAGTGATAGGT

GTTTCTTTTGCTTGCAAGTGTCTAAGAATAACTCGTAAGCACCATGCGTACGCGCGATCAGAACGGCTGCTCGATCGAGA

CTGAATGCTCGTGCTGTGAAAAATAACAGCGCTAGCAGAAAAAAG

>TRINITY_DN12843_c0_g2_i1_Gypsy-8_HMM_gag#LTR/Gypsy48-908[Heliconiusmelpomenemelpomene]

AGAAATTACTGTCTTGATAACTTTGACTTTGGTTTTACGCTTCAGCATCCTTTCAGGTGTTGTGATCCAAGCAACCGGTG

GGATAACTACGTCACCACAGCGCGCGAAGTTGAAAAGCGAGCTCATCAAAAGGCTTTCGGCTTCGCGTGAGAAGAAAGTA

AAGCAACTTCTCATGCACGAAGAACTAGGCGACCGGAAGCCATCCCAGTTTTACCGACACCTTCTTAACTTGGCCGCCAG

GGCCGGCCGTCAGTCGTCAGGCCCCGAAGAGTTCGTGCGCACCATCTGGACGAGTCGCCTTCCCAGCAGCATGCAGGCGC

TCGTCACTTCGCAGTCCAAGACCGACCTGGCGGAGTTAGTAGAGTTCGCCGACAGAATCCACGATATTGTGGGTATCCAG

GTAGCATCAAATTCTGCCATTACAACACCGACTTCATCAGGTCATGCAGGTAACCCAGTTAACCCTGAGATCGCAGCACT

TAGCAAACAAATAGAAATGCTTGCTAATAAAGTGGACAGATTGTCACGAGCTAGGGACCACTCCAGTAGTCGTACGCGTT

ACCGACATCGATCCGGTTCGAACCGGTCTGACGCTAGCTATAAACGTTTCCCACTGTGCTGGTACCACCAAAAGTTTGGG

GAACGGGCGAGGAAGTGCGTTAAGCCGTGCGATTTTAAGGCGGGAAACGAGCGGGGCAATCTGTAATGGCGACCACTGAT

TGTCCAAGCACAGGTCGCTTA

>TRINITY_DN12843_c0_g2_i2_Gypsy-8_HMM_gag#LTR/Gypsy48-908[Heliconiusmelpomenemelpomene]

AGAAATTACTGTCTTGATAACTTTGACTTTGGTTTTACGCTTCAGCATCCTTTCAGGTGTTGTGATCCAAGCAACCGGTG

GGATAACTACGTCACCACAGCGCGCGAAGTTGAAAAGCGAGCTCATCAAAAGGCTTTCGGCTTCGCGTGAGAAGAAGGCG

AAGCAACTCCTCATGTATGAAGAACTAGGCGACCGGAAGCCATCCCAGTTTTACCGACACCTTCTTAACTTGGCCGGCCC

TGGAGTCCCGGAAGAGTTCTTGCGCACCATCTGGACGAGTCGCCTTCCCAGCAGCATGCAGGCGCTCGTCACTTCGCAGT

CCAAGACCGACCTGGCGGAGTTAGTAGAGTTCGCCGACAGAATCCACGATATTGTGGGTATCCAGGTAGCATCAAATTCT

GCCATTACAACACCGACTTCATCAGGTCATGCAGGTAACCCAGTTAACCCTGAGATCGCAGCACTTAGCAAACAAATAGA

AATGCTTGCTAATAAAGTGGACAGATTGTCACGAGCTAGGGACCACTCCAGTAGTCGTACGCGTTACCGACATCGATCCG

GTTCGAACCGGTCTGACGCTAGCTATAAACGTTTCCCACTGTGCTGGTACCACCAAAAGTTTGGGGAACGGGCGAGGAAG

TGCGTTAAGCCGTGCGATTTTAAGGCGGGAAACGAGCGGGGCAATCTGTAATGGCGACCACTGATTGTCCAAGCACAGGT

CGCTTA

>TRINITY_DN12843_c0_g2_i3_Gypsy-8_HMM_gag#LTR/Gypsy48-908[Heliconiusmelpomenemelpomene]

AGAAATTACTGTCTTGATAACTTTGACTTTGGTTTTACGCTTCAGCATCCTTTCAGGTGTTGTGATCCAAGCAACCGGTG

GGATAACTACGTCACCACAGCGCGCGAAGTTGAAAAGCGAGCTCATCAAAAGGCTTTCGGCTTCGCGTGAGAAGAAAGTA

AAGCAACTTCTCATGCACGAAGAACTAGGCGACCGGAAGCCATCCCAGTTTTACCGACACCTTCTTAACTTGGCCGGCCC

TGGAGTCCCGGAAGAGTTCTTGCGCACCATCTGGACGAGTCGCCTTCCCAGCAGCATGCAGGCGCTCGTCACTTCGCAGT

CCAAGACCGACCTGGCAGAGTTAGCAGAGTTCGCCGACAGGATCCATGATATTGTGGGTATCCAGGTAGCATCAAATTCT

GCCATTACAACACCGACTTCACCAGGTCATGCAGGTAACCTAGTTAACCCTGAGATAGCAGCACTTACCAAGCAAGTAGA

AATGCTTGCTAATAAAGTGGACAGATTGTCACGAGCTAGGGACCACTCCAGTAGTCGTACGCGTTACCGACATCGATCCG

GTTCGAACCGGTCTGACGCTAGCTATAAACGTTTCCCACTGTGCTGGTACCACCAAAAGTTTGGGGAACGGGCGAGGAAG

TGCGTTAAGCCGTGCGATTTTAAGGCGGGAAACGAGCGGGGCAATCTGTAATGGCGACCACTGATTGTCCAAGCACAGGT

CGCTTA

>TRINITY_DN12843_c0_g2_i4_Gypsy-8_HMM_gag#LTR/Gypsy48-908[Heliconiusmelpomenemelpomene]

AGAAATTACTGTCTTGATAACTTTGACTTTGGTTTTACGCTTCAGCATCCTTTCAGGTGTTGTGATCCAAGCAACCGGTG

GGATAACTACGTCACCACAGCGCGCGAAGTTGAAAAGCGAGCTCATCAAAAGGCTTTCGGCTTCGCGTGAGAAGAAGGCG

AAGCAACTCCTCATGTATGAAGAACTAGGCGACCGGAAGCCATCCCAGTTTTACCGACACCTTCTTAACTTGGCCGCCAG

GGCCGGCCGTCAGTCGTCAGGCCCCGAAGAGTTCGTGCGCACCATCTGGACGAGTCGCCTTCCCAGCAGCATGCAGGCGC

TCGTCACTTCGCAGTCCAAGACCGACCTGGCAGAGTTAGCAGAGTTCGCCGACAGGATCCATGATATTGTGGGTATCCAG

GTAGCATCAAATTCTGCCATTACAACACCGACTTCACCAGGTCATGCAGGTAACCTAGTTAACCCTGAGATAGCAGCACT

TACCAAGCAAGTAGAAATGCTTGCTAATAAAGTGGACAGATTGTCACGAGCTAGGGACCACTCCAGTAGTCGTACGCGTT

ACCGACATCGATCCGGTTCGAACCGGTCTGACGCTAGCTATAAACGTTTCCCACTGTGCTGGTACCACCAAAAGTTTGGG

GAACGGGCGAGGAAGTGCGTTAAGCCGTGCGATTTTAAGGCGGGAAACGAGCGGGGCAATCTGTAATGGCGACCACTGAT

TGTCCAAGCACAGGTCGCTTA

>TRINITY_DN13327_c0_g1_i1_Harbinger3_DR_tp#DNA/PIF-Harbinger362-1480GenewisedwithHarbinger-5_XT_tp,1intron(1038:1118)[Danio]

CCGGTCTATAAAATTTATTGCAGTTAAAATAAAGACATCATAACAACTAATAATTATAAATCAATTATGAGATGCATTTA

AATGTAAACTTTTAAAATAATTATTTATTAGCTCTCTTCGCTTAGGATTTATATCTCTCATAACTCCATTATTTCTTTGT

TCCCTGATATGGCCCTGACGGAACAAATCTTCCCATGGCATAATTAACTCAAGTTCAGGATCATCTGGTGGTAACTCTTC

CTTTCCCTTTATTGCAATATTGTGCAGTATTCCCGTTGCAACAATAACAGCAAGACATTTTTTCACTTGTATCTGCATCC

CCAAGGCAAGTACAGGGAATCTTCGTTTCCACACCCCAAACATGCGTTCAACAGGATTTCTGCTTCTGATTTGAGCCTCA

TTATATAGTATATCCTCTGGGGTTTGTGGATTGTCCAGTGGCATCATGATATAAGGCCGATTCATGTACCCACTATCAGC

CAATAATACAGCATCCCTATACTTGCCGCTTTCGAACAAAGCATTCCTGTAGGAAGCGTTCCAAATAGTTTGGTCTTGGC

TACTGCCAGGCCATCTTGCCACCAAATCTAAAATTTCTAAATTTGCACTGCAAATCGCTTGCACATTTATGGACATGTAG

CCTTTCCGGTTCCTAAAATATTCTGCATTTACCCCACCAGGGGAACGAATCCGTACATGTGTTCAATCTAGAGCACCCAA

AGCTTTTGGAAATTTTGCAATATTATAAAAATTCAACTGAGTTTTCCTTATCTCTTCCACTGTGTCTGGGAACTTGATAC

AATTGCGGCTTAATGAGGCTATTGCAGCAGTGACTCTGTGTATGATTCTGTGAGATGACGATTTACTTATACCGCAATAA

TCACCAACTGATATTTGTTGGGAGCCAGTCGCATAGTATCTCAAAGTCAAAAGAAGCTGATTTATGGGTGAAATTGATTC

GTTTCGATCTGATGGATATTCTAATTTATCTTCGATCAACTGGAGAATATCAAGGGTCATATCTTTTGATAAACGAAACC

TGGTACAAAAATCGACATCATCGTATTCTTCGAAATAGCAATGACGATCTTTAAAAATTCTGGGACGTCGTACAATTCCA

TCTAAGCAATCTAAATTTTCTAAAATGTTATTTGCAAAAATAAAATTATATGCAGCGTCCATTTTGACGTGACTTTGACA

ATCTGTTAGTGTCTAACAGACGTCTTGACCGGTGATTAGATTTAATGCCTTGTTAAGCGAAGTTAACAGACGTTTAGTCG

TGGTGGGACACTCGATAACTTTAACAGTCCGTTATCTGTCTTAACAGACCGTTAAATATTTAACAGAGTATGGTGAAACT

GGACCTTAAGACGTTTCAAAAAAGACAATTGTTTAAAATATATATATTCTTTTCTGCCTGATGCTCCAGATCCGCTTTTG

TTTTGCTTCCCAATTTCTCTGTTGAAACTATCGCGCAGACTCTTCCACTTTCGTTGTAATGTGGCAGCTGCGGTGCTTTT

TTCTTTTCTATTTTTGTCCTTGAAATTGTTAATAAATAAATAACAAATCTCTTCCCAGGCTTTTATTTTCTCATTTCTAT

TACTGTACAGATCAGAATGTACATCCCAAATAGCGGGACGTGCATGGATTGCCGAAATAAATGTATCAACATCCATTGAG

GCGTCGTGCTCCATGGTGACGCGTGCGATACGTAGAACGTCTAACGGTTAACTGCGCGGGGGGTGGAGCG

>TRINITY_DN13327_c0_g1_i2_Harbinger2_DR_tp#DNA/PIF-Harbinger2140-3243[Danio]

CCGGTCTATAAAATTTATTGCAGTTAAAATAAAGACATCATAACAACTAATAATTATAAATCAATTATGAGATGCATTTA

AATGTAAACTTTTAAAATAATTATTTATTAGCTCTCTTCGCTTAGGATTTATATCTCTCATAACTCCATTATTTCTTTGT

TCCCTGATATGGCCCTGACGGAACAAATCTTCCCATGGCATAATTAACTCAAGTTCAGGATCATCTGGTGGTAACTCTTC

CTTTCCCTTTATTGCAATATTGTGCAGTATTCCCGTTGCAACAATAACAGCAAGACATTTTTTCACTTGTATCTGCATCC

CCAAGGCAAGTACAGGGAATCTTCGTTTCCACACCCCAAACATGCGTTCAACAGGATTTCTGCTTCTGATTTGAGCCTCA

TTATATAGTATATCCTCTGGGGTTTGTGGATTGTCCAGTGGCATCATGATATAAGGCCGATTCATGTACCCACTATCAGC

CAATAATACAGCATCCCTATACTTGCCGCTTTCGAACAAAGCATTCCTGTAGGAAGCGTTCCAAATAGTTTGGTCTTGGC

TACTGCCAGGCCATCTTGCCACCAAATCTAAAATTTCTAAATTTGCACTGCAAATCGCTTGCACATTTATGGACATGTAG

CCTTTCCGGTTCCTAAAATATTCTGCATTTACCCCACCAGGGGAACGAATCCGTACATGTGTTCAATCTAGAGCACCCAA

AGCTTTTGGAAATTTTGCAATATTATAAAAATTCAACTGAGTTTTCCTTATCTCTTCCACTGTGTCTGGGAACTTGATAC

AATTGCGGCTTAATGAGGCTATTGCAGCAGTGACTCTGTGTATGATTCTGTGAGATGACGATTTACTTATACCGCAATAA

TCACCAACTGATATTTGTTGGGAGCCAGTCGCATAGTATCTCAAAGTCAAAAGAAGCTGATTTATGGGTGAAATTGATTC

GTTTCTGAAACAAAATAAATGATTAATTTTATAAAACATTCATGTATTGAATAAGCCTTATAAATCTTACCTGTATTATG

TAAATCTGTGTTCGTAAAGTTATAAGGTTGTAGGTGGATATAATCAAAATATCACACATGATAAGATGGGTTGTATAAGT

GCATAAAAATACTACTCAATGATTTCTTGCATTTCATAACGTACATGATGGTCTTAATTAATTAATAGACGACTTACCGA

TCTGATGGATATTCTAATTTATCTTCGATCAACTGGAGAATATCAAGGGTCATATCTTTTGATAAACGAAACCTGGTACA

AAAATCGACATCATCGTATTCTTCGAAATAGCAATGACGATCTTTAAAAATTCTGGGACGTCGTACAATTCCATCTAAGC

AATCTAAATTTTCTAAAATGTTATTTGCAAAAATAAAATTATATGCAGCGTCCATTTTGACGTGACTTTGACAATCTGTT

AGTGTCTAACAGACGTCTTGACCGGTGATTAGATTTAATGCCTTGTTAAGCGAAGTTAACAGACGTTTAGTCGTGGTGGG

ACACTCGATAACTTTAACAGTCCGTTATCTGTCTTAACAGACCGTTAAATATTTAACAGAGTATGGTGAAACTGGACCTT

AAGACGTTTCAAAAAAGACAATTGTTTAAAATATATATATTCTTTTCTGCCTGATGCTCCAGATCCGCTTTTGTTTTGCT

TCCCAATTTCTCTGTTGAAACTATCGCGCAGACTCTTCCACTTTCGTTGTAATGTGGCAGCTGCGGTGCTTTTTTCTTTT

CTATTTTTGTCCTTGAAATTGTTAATAAATAAATAACAAATCTCTTCCCAGGCTTTTATTTTCTCATTTCTATTACTGTA

CAGATCAGAATGTACATCCCAAATAGCGGGACGTGCATGGATTGCCGAAATAAATGTATCAACATCCATTGAGGCGTCGT

GCTCCATGGTGACGCGTGCGATACGTAGAACGTCTAACGGTTAACTGCGCGGGGGGTGGAGCG

>TRINITY_DN9777_c0_g1_i1_CR1-4_BF_gag#LINE/CR1-Zenon234-935[Branchiostomafloridae]

AAAGCCCCCCCGCCGGTTCTGTGTTACAATTCTAGTGAAATATGTACATATCAATAGTTTAAATTAAAAAAGAGGGTAGG

ACTGCCTCACGGCAACACGAAACTGTTAAACTTAATATGTAAAATATAATGACTATGGAACGCAACTGCAGCTCAAAAGC

CGTTAGGCCAAGATTGACAAAATAACTGTCATAGACATTACATAGGTGACAGTTGATACGGGAAAATAGTGCTTGTCATA

AACTAATTGAAAAACGTAATAACGGTTAAAATCGTTCAACGATAAGTTTTAAAGCATAAAAATAAAGGTAAATGAGAAAA

CTTTGTCAAATTTTACGTGTTTTGCCATGAAATAAAATCTAATATGAATGAATTACTAAAGTTATGGAATACGGACAAAG

TAAAAGGTTACCGTGAAGCAGCCATGCGGACTCGGAATACACCTGCAACCACTATTTCCTAACTGCCACAGCCCGCTTCT

TTCGTGGTACAGCAGCAGCGACTATGGCGGGCGATACAGAAGCTGGAGATGTTGCGACGTGCTTGACAGGGCCCTCTGGA

GAATCAGCTATCGCAATAACATCTCGTGTGCTGAGAACCCGGCGCTTGGTACCGTCGGCACCCAGTACAAACACCTCGCC

TCGCTGTGTCCAGCAGTTGGAGATGCCGAACTTCTCACGTGCCATCATGAAAGCGTCGTGCCGCGTCTTCGTCAGAAATT

CTGACAAAGTGATGCCGGAACCCTTCAACTTCGTCTTCTCGAACCAAATCCTATCTCTAACTTCCACGTCCCTTAACTTG

AGGAGTATTGGTCGTGGTTTGCTCCCGTTGTTATTGCGGCCCATCCTATGGCACCTCCGTACGGCAGCAACAGAGAAGTC

TGCAATTTTTACGTGCTCCAGCATCACGCCAGCGACCACCTGGGCAGTATCCTCTTTAGAGTGTTCAGGAACACCATGCA

GGAGGAGTATTTTTCGGCGACCCCTCATCTCTAACGAGTCAACGTCTCGAGCGAGTGTCTCCATTTGTCCTTGGAGGCAA

CTGAGGACTTCTGTTACCATCATCCTGAATGCTGCGAAGTCTGCCGCCAAAGTGGCGGCTGAATTTGGGGACTGCTTCAA

CTCCGCCTCGAACTTGGACATGCGGTCATGAAAACTTTTCATCATGTCGGACATTGACCCCTGTAGAGCTTCCATGATGA

TAATTGCTTTTAAACTATTGTGGTTTTAAACTATTAATTGGATACCACAGAAACGGCAGGCAGTGAGTGTTAAATGTGTA

ATTTCATTTAAAACTTTGTGAATAAATACTTTTAACTATTGTTTCTAATTATTTAACACAGAGATCAAATTTCGCGACCA

CTCTCAGTGCTACCTCCGAGAGAACATTTATTGTCTTTCTATTTTCCACACCTTGCGTTACCCTGGACTATCTGCTTAAC

TGGACATTCGCAGAGCTTTTCTGGTTAGATCCGTCCGAATCCTCGTGGATTCGCTCTCCTTCGGTGATTGTAGTGTTAGA

CTTACTGACTGAACTTAAACAGCCGAGTGATGACTAAAAAAAAATGTGACTCTCACACTAAGACATTTAATGTTTATGTA

GCGGGGGTTTCAAAAAGTTACAAGATTCATGCTTAAAAGCACCCAGCAGAACAAGCGTTATGGATAACACAAACGCTTAT

CAGGAGCTGGGATTGACCCGCGGCACGTCGTACACAGTGGGTTTGGCGTAGTCACCTATACGACTGCCCTCCATGTAGTC

TACTTTGTTTACTTTACCCATAAAATTACATATTTCAAATTAAGCTAGCAGCTCCAGTAAGCCTTTAACTGTCAGGTTTT

ATTTCAGTTAAATTGGAGTTACCAGACTCGGCATCGTCCATGAGAACTTCTCGGCAAACCTTCACTCCTAGCAGTCAAAG

CACCCCGCGGTCACCAAACTCAGCTGCCAGTAGTCCCGGCACGCGTACCCCTCGCCGTCAGCCCCCCCGTCAATCTCATC

AATCCCGTCCTCCACTTCGCAAGATCACTACGCCAAAGCCAGCGAACCCTCCTCATACGTATAAAAAGAAACGTGTCACC

GTAGTATCAGCGACGAACAAAGTCAAGAAGAATGAAGTTATTGTCACCAGACGATAGAAAAAATTACCCTTGACGATTTT

ATTTTCTGAAAGTTTGGAGAAATAAATCTTTGATACGATAAAAAAA

>TRINITY_DN9796_c0_g1_i1_CR1-5_NVi_pol#LINE/L21509-4384viatfastywithCR1-73_HM_pol,1frameshift.[Nasoniavitripennis]

TCTTGTGCTTTATAAGCTCGATACAGAAAGTTGCTTGAACAGTTTCTTTACTAGGATATTTTTGTATGAGACTTTTCACA

CAAAAAACTATCTAATATATACACAATCACACTTAATTTGTACTAAATACCCAACCCACAACTTCTGCATGGACACTTTT

TGTCCTCAATACACATATCAAAATACACACATACAAAATGAACACGCGCGCCTATACGAGTATTACTGTCTTATTTTATT

GATAAAAGCCGATAACTGTATTTTAAAACATTTGACTGTAGAGATACCCCTAATAGCGTTTTTACTTATGTATGGATAGT

AATCTGATAACCTTGGGCGAGTTGGACAGTGTGGAAATTGCCAAGTCAGAATTAATTGACGTCACTAACATTCCTGGGGA

GGTGCGAAATTCTGGAATGAAGATTTTAACGCAGAATATAAGAAGTGTATACCGTAATTTTGACGACCTGCAAATCAATC

TGCAACAAATTGCTTTGGACCTCGATATACTGCTGCTCACTGAATGTAGACTCTCAAATACGAAAATTTTACCATTGCTC

CCCAATTATACATCGTACTGCACTTCAAATCATCTAAACCAAAACGATGGCGTCGTAGCGTATGTTAAAAATAACCATTT

GCCCATAATAACAGAATTAAATATAGTACATGCCTCAGCTTTACAAATAGTTTTCTCGGGTTTTACTATAATAGGAATTT

ATCGTTCGCCATCCAATAACTCTGCCGAAAATTTTATCAACTCAGTTAACAGTCATCTGGAAGCATGTGGTTCTCATAAA

AATATAGTGGTGATTGGTGACATTAATATAAACATAATTGAAAAACCTACAGAAACACCACAAAAAAGATCCAATAGGCT

TAACTATCTGAATATGCTATCTGTACATGGTTTACTTCCGGGACACTGTCTCCCTACAAGAGAGGAGAACTGTCTCGATC

ATATCATGATTAGACTTGATAAAACTAGATACTCAGCCTTCGTGTCTGTACTAAATACGACCATAACAGACCATGCCATG

GTAGTCCTCGGTGTAACCAATTTACATACTATAAGAAAAACAACACGTAAAACTGTGACTGTAACAGACTATGAAAGCGC

TCGAAATACCCTTTGCAACATAGACATTGCCCTCTTTGATACATATAAACTCCCAGATACTTTTGCCATAAAACTAATGG

AAGTGATAAAATCATCACTCCTCGAAAACACAAAAATCAAATTGGTTTCCTGTAATAAGAAAACTAAAAAACCCTGGATC

ACAGCGGGTGCACTAAAATGTATTAGGCTAAGAAACAAAATGCACCACCAAACAAGAATTGACTGTTTTAATTTAATCCT

GGTAATAACCTACAAACGATTCCGAAACTTCTGTTCCAATCTAATCAAAAAACTCAAAAGACAGTATCATAAAAAACAAA

TAGAGGAATCATCCAAAAACCCGAGAAAACTGTGGACTAAAATAAATGAGATAACGCAGTTCAGGACACCTAAAAACTCA

AACGCTCACTTACTTAACGTAGCACCCCAACCACAGGACTCTTTAAACCGAGTTAATACGTTTTTTAGAACAATAGGTAA

ACAACTAGCCGATCGCATTGGCACCCAAGGCCAGGTGGATATACCGGCAACCACAAACAGTTACACTTTTTCTAATTCAT

TTGTTCTAGAGGATACTGACCCATTGGAAGTGAGTAGTATCCTTATGAGTCTAGACTCAAAGAGTGCACCTGGCTGGGAT

GGGATCCCCACTACATTTCTAAAATTATCACGGGATGTAGTGGTCCCACTGATATGCCGACTGGCTAACCTCTGCTTTAA

AACAGGAACTTTCCCTAAAATTTTTAAACGTTCAGTGATCACACCAGTGTATAAGGGTGGGGACGTAAACGACATAAACA

ATTACAGGCCTATTGCGGTACTACCATGTATATCTAAAATACTAGAGAAGCTATTAAACAAAAGACTAATCAGCTATTTG

CAGAAATATAAAATCATATCAGATTCACAATTCGGGTTCAGACAAGGGCTCTCAACACAAGACGCCATAAAAGCCCTAAC

ATCTGAAATTATTGACAAGGTAGATAAAGGTCAAAAGGTTCTAACAGTGTTTATAGACATAAAAAAAGCCTTTGATACCG

TTTCCGTCCCTATCCTTGTACGTAGGCTTGAAAGCATAGGTATAAGAGGCACAGCTCTCAACTTATTCGAGAGCTACCTC

CAAAATAGATCACAAATGGTGAAAATTGATAATTTAAACAGTGACTTAGGAGAAATTGAGTATGGCGTTCCACAAGGGAG

TGTGCTCGGCCCAACACTCTTTTTGATTTATATTAATAGCTTATGTAATTTAAAAAGTAACGGTGGCCGAATCTTCTCTT

ACGCAGACGATACCGCAGTTGTTTATTCGGGAATTACATGGAGTCACGTAAGAATTGCTTCTGAAAACGGTCTTGCAGAA

ATAGCAAAATGGCTCACAGAAAATCTTTTAAGTCTAAACACCAACAAAACTAAATTTATTTGCTTTTCTGCAGACCAGAG

AACACAACCAGATATAGGATTTGGGGTCACAATTCATGTTTGTAATAGGAGTGTCTTGAACTGTGACTGTCAGGCTATAG

AAAAGGTAAGCTATACAAAATATCTAGGTATTATAATTGACCAAAGATTGTCTTGGCACTCTCACCTAGAAATTCTAATG

AAAAGAATCAGAAAGTTTATCTGGATATTTAAAACACTGCGCCATTTAATGACTGCACAGCTTTTAAATAGAATATACGT

TTCTCTGGTTCAGTCAGTAATTGCTTACTGCATTCCAGTATGGGGAGGAGCCTCCAAGATGAAGGTCCTTGATCTTGAGA

GGGCGCAGCGTTCATTAGTTAAGGTGATGTACTTCAAACCCTATAGGTTTCCTACAACTGACCTCTATAAGGTCAGTCAG

TTGCTATCTGTTCGGAAATTATATATCCTAAATTTAACTCTGTGTCTACACAAATCACTTCCCTTTGATGTATCCACTCA

GAGGAAGAGACGAAGAGATATAATGGCTTACTCATCTATGGTTCGCACAGTTTATGCGCAGAGGCAATATATGACCCAAT

CTACATACGTTTACAACAAAATAAACTCAAAAATTCAGATATACCCTCTCATGCTACATGAATGTAAAAAACGAGTACTA

AAATGGCTAAATACACTAACGTACGAAGATACTGAAAACATCCTTATCAGAATGAGCTAAAGCATAGAACAAATCGCGCG

CACACTCGCACACTCACACACACAACACACACACACACACACACACACACACACA

>TRINITY_DN9771_c1_g1_i1_Harbinger1_DR_tp#DNA/PIF-Harbinger977-2128[Danio]

TTTTTTTTTTTTTTAAGATTTTACTTATTTAATACTTGTTGTCTAGTATCTTTTACTAAATATTTCTTTAAGCAGTTCAC

ATTTTTCTCTTTCTATTTGTAATGTATCTCGATATTCTTTTTCTTTTAGCTGAATCTTTTTTAACTCTAGAGTTTCTTTA

ATCTCCAACTCTTTTTTACGTAGTTCCAGTTCTTCTTCACGCAATTTCAAATTTCTTTCGTAAATTTCATCCCTCCACTG

CTTCTGTTCTTCTTGCCTCCTTAAATATTCTAACCATTGCTTTTCCAATTGTAATTTTCCCTGAGACCTATCACACTCTA

TTCTGTTGGCTAGTCTTATCTTCCTCATTTCTATAACAGCTTTGCTTTCTTGAAATGGTTTAATATTGTGATCATTTATA

TTCAGGTCAACTTTATTACTATCTTTCTTCCTGTGTAGGAGTTCTGTTAATGTGTAAGAATCACAATCTACATGGTATTG

TGCATAAATATCATTCATTATTTTAAAATATTTAAACTTGTCATGATGCCCGGTATCAACACATTGCTTATACTTTTTTG

TTAATGCATTTATTTTCCACCTTACTTGATCTGGTGTCACCTCTATGCCATATTCCATTAGGCTTTGTGATATTGCCAAC

CAGATTCTTGTTTTCTTTTTTGGTGTTTCTAGCATTCCTAGTTTGTTTTCATATAGTTTCAGTAGTGTTGTTGTAGCATT

AGTAGTCCACACTGCACAGGGTTTCTCGGAACCATCAATTGGCTTTGATTCTGAATTAACTGTATGAACTTCATCAGTGC

TTTCACAGATTTCTAAATTGTCTTGGGAAGCAAATCGATTCCAGGCGAGATCTGATTGTCAAAAAAACATTTAAAAACTG

GACAAGAACTTAAGTTGCATGTATGACCCAAAGACATGTATGACCAGAATTTTTGAAGTTAGTATGCTTAGTAATTTTTG

TAACTGTCATTGCCACCACTATCTCCAGCTACACTATGTCCACGGAACCTATAGGTATACCCCTAGAGGGACTGTCTTGT

TTGAAGTTTAGGAAAAACGTCAATAATTATTGTTTGTTTATTTTTGTCAGTCACAACGTAACGTACTTACGAAAATATTA

TTTGGGCCTAAATATGCGTTATTGGCAAAACTTATATTCTGGATGTGTACTAGAATAACCACTTCAAATGAAACAATAAA

ATTAGTATTTCAGTTACAATGGACAAACAAAAATTAATCTTGTGGTTAAGCGAAACAGCTCAAAGTACTGACGAAAGTGA

TTCAAGCGAGTGGAGTGATGTATGTACGGTGAAACAAGATGTTTCTGAAGAAGAGGACTTTAGTGACAATGATGACGACT

CACTATTTTTTCCGTTAATGCAATATTTAGTAAGATTGCGAAGAAAACGTGTTGATGATTATCTGCATATAATAGATTCT

TGGACAGATTCCGAATTTAAAAATCGATTAAGATTATCACGGAAGACTGCTTACCGGCTTATTGGTAAAACGAAAACTCC

AATTAACTTTTCATTAACCAACTCTTCTAATACTATCCACATTACTTATTTGTTTCAGATGAATTAGAAAAATCTGGGTT

CATAGCATCGCATAAGTTTGGATTAAAACCTTTGGAGCCTAAACTTTGTATTTACATATTCCTCTCTTTTATTGCAAACA

CTGAACCACTGACACCAATAGCAACACGATTTGACATTTCCATTTCATCAACATTTAGAGTTATTAGAAGAGTGGTGGCT

TGGATCCTTACAAAATTGGATGATGCTATAAAGTGGCCTCAGAGTTATGAAGAAATAAGCTACTTATGTGATTCATTTAA

CACCAAGACTGGTATTACTCACATGATTGGAATTATTGACTGTACCCACATTAAAATTGAGAAACCCAAGAATGCAAGAG

AGTATTGTAACCCTAAAGGATACTTCTCAATTATTCTTAAAGCCACTATTGATGCTAATTTGAGGTTTACAAATGTTTTC

TGTGGTGAACCAGGCTCATCTAACTGTGCTAGAGTTCTCAAGAAATCTCCTTTGTATAATACTGCAACACAAAATAGGAA

TTCTCTATTTCCACATAATACCTTTTTAGTTGGACATTCAGGATATCCATCTTTACCTTGGCTCATGCCTCCATTTAGAG

AAAACAAAAGACTGACACCTCAGCAAAGAGAATTTAATGCCTTACACACATCAACAAGGAAGTTGAGTGATAAAGCTTTC

AGTTTATTGAAGGGGAGGTTTCGTAGAATAAAACTATTCACTGTGTATAGAAATATAGCCTTTATAACTGATACAATTGT

GGCTGCATGTATCTTGCACAACTATTGTCTCAATGAAAATGATCATTTGGAAGAACATGAATGATTTATAAATATTTTGC

TCTAAGCAATAAGTATTTTCTCATGGACAATATTTGTGGTTCCTTCTTTCAGCATGTAAACATGTTGCAGATTTTAAATA

AGTCTTTTGTATAAAATGTAGATTTCACAGCATCATGTTAAACAATATTTATAAGTATAAAAGTGCAATTGCTATGAATG

GTTACTATTGCCTCATTAAATCCTGATCAGCCAAAAGCTTTCTCAAAAGTGCACATTTTTCTCTTTCAATTTTAAGCATT

TCTTCTTGTTTTTTCTCTTTGAGTTGTAATTTTTTTAATGCTAATGATTGTTTAATTTCTAATTCTTTTTTTCTTAACTG

TAACTCCTCCTGTCTTAGCCTCAAATTTCTTTCATACCTCTCATCTCTCAATTGCTTTTGATCCTCTTGTTTTCTTAAAT

ATTCTAACCATTGTTTATCCAGCTCTATTTTAGCTCTTCTTTCTGTCCTCAATCTTCTGAAAGGTGTTGAGCCTTTGAGG

GTCAAATTATTTCTTTTACTTTTATCTTTATCTAAGTCTTCTGAACCACCTGGCATTACACCAGATGCTAGTCTGTAGGT

TTCAGAATTATCTGAGTAGCGTCCCAAAATTTGGTGCATTTCATTAAAATATTTAAAACTCATGGCCCCTTGTCCATTGT

CTATGCAGTCTTTATACTTTTTTGTAAGAGCATTTATTTTCCATCTGACTTGATCTGGGGTCACCTCAACACTCAATGAT

TTTAATTCCTCAGCAATAGAGAGCCACATTTTAGATTTCTTCTTTGGGTTGTCCAGCATATGGATTTTAGTTTGATATAA

ACTTAACAACAAATTAGTTGCATGTTTAGTCCAAATGACATTTTGCTTATTGCTCTCATTAACATCTGTTTCTTCGTGTG

TCACAGTTATTGTGTGACTAAAGTTATAGTTATTCATATTCATATTCGGTTTATATTGGTTTGCTGAAATACATAAAACA

ATACATTGAAATCTATCCTTGGTTATAAACAAATTTTCAAATTTGACATGTATTTGTTTATTAGCTTACACTGGTTATCT

AAAATAGCCAAAACGGCAACATTACTATCAATGTTTTCATCCATTATGTGTCGACAACTTGGGCGTTTTCCAACAAGTAC

AATTACAGTGCACGGTAACTTTTATACTAGCCGCAGATTGACCGTGTAGACCAACCTCTAACTTCAAAGCTTTATAATAT

TAAAAATCGAGTAACAACATGTATGATACTGCAACATGTCGTGTAATTTGTTATTGCATTATATTGATTTCATGAAATAA

ATACAGCGTTCTCACACAGATATTAAACACAAAGGGCACACAGACGTCAAATTGACATTTGGCGAAACACAGTGCTGTTC

TTGTCATTTCTATTGAGCCTATAGGGTTAGACTTCGGTTTCTTATTCCAAAGTATTATAAACTAGTTTTTCAATAATATT

TACTTGGAGATGCATTGTTTCTTAGCCTAACATAACCTAAACAAACTATGTGCAACTTTTGTGCACGATTAAAGGTTTCC

CTTTCTGTAGAAAATTGAAGAGTACTTGTTTAAATGTAATTTACAAAAAGACTTAAAAGAGACACTTCTAATGGAAAAGC

ATTTCGATATTAACACTGAACTATGTTTACTTGGCATTACATAAATGCTAGCATATTTTATTTTTGTCACTCAAAATCTT

GCAATGTATCTTCATCAATCACTTTTGTTTGTAGGTGAAGCTAATAATCAGCCGTAGACAGCATTTAGAAAAAAATCAAA

CTTGCTGATTCAGGAGAACTTAGCAAATGTGACGAAATCTCGTGTTGTTTTACCTCCTTTGAAATAATGATCTATTATAT

ATAAATATAAATTTTTAATGGCAATATTTACAATATCTATCAAGTAATTTGACAGATTTGATTTGACATTCGTTTGCTCG

TTGACGTGACGTGACATCGGGCGATTAGTGCATTTACTTTAAATTAAATGAATAAATGTTACTTATTATAATCAAATTTT

GTTAACCTTAATAACACAAAATGGCTCAATGTGGTGACTTTATTAAAAGTCAATTCCCACTCATAGATGACGAATTGATG

AAATATGTTGAAGACATTTTGGATAGCAGTGCCGGTGAATTTGAAGACACGGAAGAAGTGTATGAGGCAGTTGGAGAAGT

GCTTCAAGGAATCTCTGAGAAATCTGAACAAGATATAAGGGATATATGTGAGAAGCTATTACACATGCTCCAGCCTGACA

AGCCGGGTAGTAGCAATGGACCAAGGAGAGTATTAGATGCCCCAGTACACTTGGCGTCCATGACTACTACTGTGACTGAA

ACTGAAGATCTCAGAAGTATATGGATCAATACTAGAGATGATAACTTGAAAGTGGACGCAAAGAAATTAGAAAAGGCTGA

AGCTAAGTTACAACAGAAACAGCAGAAACAAAAGGATTCAAAGTTACCAGTGACAGCCCCAGTTCTTCAGACAGCGACGG

CATCACAAGTTACGTCTAAGAAGGACAGTAAACTCGAAGCTAAGGGCACGAACAGGACACAGGACATTAGGATAGAGAAC

TTTGATATTGCTTATGGTGATAGAGTATTATTACAAGGCGCCGACCTAGTTCTAGCGTGCGGCCGGCGATATGGTCTCGT

TGGGCGCAATGGGCTCGGCAAGACGACACTCCTTAGAATGATTTCATCAAAACAACTGAAGATTCCGTCCCATATATCAA

TATTACATGTAGAACAAGAGGTCGTGGGCGATGACACTGTGGCCCTACAAAGCGTGTTAGAATGTGATACTGTTAGAGAA

ACATTGTTGAAACGAGAGAAAGAAGTGACTGCCGCTATTAATAATGGATCGACTGATACAACACTATCGACGGAGCTGAG

CGAGATCTACGCGCAGCTGGAGAACATCGAGGCGGACAAGGCGCCGGCGCGCGCGTCCATCATCCTGAGCGGGCTGGGCT

TCACGCCCGACATGCAGGCGCGCGCCACCAGGACCTTCTCCGGAGGCTGGCGGATGAGGCTCGCACTGGCGAGGGCGCTG

TTCTCAAAACCCGACCTCCTACTACTCGACGAGCCGACGAACATGTTGGACATCAAAGCGATCATCTGGTTAGAGAACTA

CCTCCAGAACTGGCCGACCACCCTGCTGGTCGTGTCTCACGACCGCAACTTCCTCGACACTGTGCCCACAGACATCATGC

ATTTACACACACAAAGGATAGATACTTACAGAGGTAACTACGACCAGTTCCACAAGACGAAGACGGAGAAGCACAAGAAC

CAGCAGCGCGAGTACGAGGCGCAGCAGCAGCACCGCGCGCACACGCAGGAGTTCATCGACCGCTTCCGGTACAACGCGAA

CAGGGCCTCCTCCGTGCAGAGCAAGATCAAGATGCTGGACAAACTGCCTGAACTAAAACCAGTAGAGAAAGAGATAGAAG

TGGTCCTCCGGTTCCCCGAGACGGAGCCGCTCTCGCCGCCCATCCTACAGCTGAACGAGGTCGGCTTCTACTACTCCAAG

GACAGAGTCATCTTCTCCAATGTCAACCTCGGAGCGACGCTAGAGTCCCGGATATGTATCGTGGGAGACAACGGAGCTGG

TAAAACGACGTTATTAAAAATAATCATGGGTATCCTGTCACCTACCAGTGGTATCCGCAGCGTACACAGAGGACTGAAAT

TCGGTTACTTTTCGCAACATCACGTTGATCAACTAGAAATGAACGTCAATTCTGTAGAACTGTTACAAAGGAGCTATCCA

GGTAAAACAATAGAAGAATACAGAAGACAACTAGGTAGTTTCGGTGTCAGCGGCGACCTTGCTCTCCAAACGATCGGCAG

TCTCTCTGGGGGTCAGAAGTCGCGAGTTGCCTTCGCGAGGATGTGTATGGGCAACCCCAACTTCCTCGTACTCGACGAGC

CGACTAACCACTTGGACATCGAGACCATTGAGGCGTTAGGAAAGGCGATAACTAAATATACGGGAGGTGTCATCCTCGTT

TCTCACGACGAAAGATTAATACGAATGGTTTGCAAAGAACTGTGGGTTTGCGGCGGTGGCTCCGTCACCAGTATAGAGGG

CGGCTTCGACGAGTACCGCAAAATCGTCGAGAGAGAACTCGAGGCACAGAACAAGTAGACAATGTAGCTGGAATCGTGTT

ATTGTGTCGTGTCTCATCGGCGTGCCGTCACCATTCTATTCTAATTCTATAGGCTAATTCACTGAGACAATCTTCGATGC

TGTTCTCCAAAAAATTAAATTTATGCTCAATTTTTTGGGGTGAAGCAAAATTCTTGTTTATGTATTATTCAATTAGATAA

ATGAATATAGGTAATTAAAATGCATTGCACCGTTACAGGTAACGGGTAATGCTGCATAAATAATGTAGAAGCTTCTTCTT

AAAAAAATGAGCATGAATTGAGAAGTTTTTGTTTTTGGGAGAATTGAAGTTATCTGTCTCAACGTGTGTTGTGTGTGAAG

CGATCTCGCTTTGACGCTTTTTGATTGGTTGATATTGTGCGAGACGACGGCGCGTCGACCCGACACCACACAATACTACG

GTTCTCGCTTATCGCAAATACACACCCACATGTATACAATATATCCATGTAAAAAGTAACCTTAAATTATATTGTTGTAA

TATACACGCAGTGTTTTATTACTTGGTTGAATACTACATACTTACTTTATTATAACTTAGTGAAAAGGGAAATGAAAAAC

TTATTAATATCAAAAAAAAAA

>TRINITY_DN9771_c1_g1_i2_Harbinger1_DR_tp#DNA/PIF-Harbinger977-2128[Danio]

TTTTTTTTTTTTTTAAGATTTTACTTATTTAATACTTGTTGTCTAGTATCTTTTACTAAATATTTCTTTAAGCAGTTCAC

ATTTTTCTCTTTCTATTTGTAATGTATCTCGATATTCTTTTTCTTTTAGCTGAATCTTTTTTAACTCTAGAGTTTCTTTA

ATCTCCAACTCTTTTTTACGTAGTTCCAGTTCTTCTTCACGCAATTTCAAATTTCTTTCGTAAATTTCATCCCTCCACTG

CTTCTGTTCTTCTTGCCTCCTTAAATATTCTAACCATTGCTTTTCCAATTGTAATTTTCCCTGAGACCTATCACACTCTA

TTCTGTTGGCTAGTCTTATCTTCCTCATTTCTATAACAGCTTTGCTTTCTTGAAATGGTTTAATATTGTGATCATTTATA

TTCAGGTCAACTTTATTACTATCTTTCTTCCTGTGTAGGAGTTCTGTTAATGTGTAAGAATCACAATCTACATGGTATTG

TGCATAAATATCATTCATTATTTTAAAATATTTAAACTTGTCATGATGCCCGGTATCAACACATTGCTTATACTTTTTTG

TTAATGCATTTATTTTCCACCTTACTTGATCTGGTGTCACCTCTATGCCATATTCCATTAGGCTTTGTGATATTGCCAAC

CAGATTCTTGTTTTCTTTTTTGGTGTTTCTAGCATTCCTAGTTTGTTTTCATATAGTTTCAGTAGTGTTGTTGTAGCATT

AGTAGTCCACACTGCACAGGGTTTCTCGGAACCATCAATTGGCTTTGATTCTGAATTAACTGTATGAACTTCATCAGTGC

TTTCACAGATTTCTAAATTGTCTTGGGAAGCAAATCGATTCCAGGCGAGATCTGATTGTCAAAAAAACATTTAAAAACTG

GACAAGAACTTAAGTTGCATGTATGACCCAAAGACATGTATGACCAGAATTTTTGAAGTTAGTATGCTTAGTAATTTTTG

TAACTGTCATTGCCACCACTATCTCCAGCTACACTATGTCCACGGAACCTATAGGTATACCCCTAGAGGGACTGTCTTGT

TTGAAGTTTAGGAAAAACGTCAATAATTATTGTTTGTTTATTTTTGTCAGTCACAACGTAACGTACTTACGAAAATATTA

TTTGGGCCTAAATATGCGTTATTGGCAAAACTTATATTCTGGATGTGTACTAGAATAACCACTTCAAATGAAACAATAAA

ATTAGTATTTCAGTTACAATGGACAAACAAAAATTAATCTTGTGGTTAAGCGAAACAGCTCAAAGTACTGACGAAAGTGA

TTCAAGCGAGTGGAGTGATGTATGTACGGTGAAACAAGATGTTTCTGAAGAAGAGGACTTTAGTGACAATGATGACGACT

CACTATTTTTTCCGTTAATGCAATATTTAGTAAGATTGCGAAGAAAACGTGTTGATGATTATCTGCATATAATAGATTCT

TGGACAGATTCCGAATTTAAAAATCGATTAAGATTATCACGGAAGACTGCTTACCGGCTTATTGGTAAAACGAAAACTCC

AATTAACTTTTCATTAACCAACTCTTCTAATACTATCCACATTACTTATTTGTTTCAGATGAATTAGAAAAATCTGGGTT

CATAGCATCGCATAAGTTTGGATTAAAACCTTTGGAGCCTAAACTTTGTATTTACATATTCCTCTCTTTTATTGCAAACA

CTGAACCACTGACACCAATAGCAACACGATTTGACATTTCCATTTCATCAACATTTAGAGTTATTAGAAGAGTGGTGGCT

TGGATCCTTACAAAATTGGATGATGCTATAAAGTGGCCTCAGAGTTATGAAGAAATAAGCTACTTATGTGATTCATTTAA

CACCAAGACTGGTATTACTCACATGATTGGAATTATTGACTGTACCCACATTAAAATTGAGAAACCCAAGAATGCAAGAG

AGTATTGTAACCCTAAAGGATACTTCTCAATTATTCTTAAAGCCACTATTGATGCTAATTTGAGGTTTACAAATGTTTTC

TGTGGTGAACCAGGCTCATCTAACTGTGCTAGAGTTCTCAAGAAATCTCCTTTGTATAATACTGCAACACAAAATAGGAA

TTCTCTATTTCCACATAATACCTTTTTAGTTGGACATTCAGGATATCCATCTTTACCTTGGCTCATGCCTCCATTTAGAG

AAAACAAAAGACTGACACCTCAGCAAAGAGAATTTAATGCCTTACACACATCAACAAGGAAGTTGAGTGATAAAGCTTTC

AGTTTATTGAAGGGGAGGTTTCGTAGAATAAAACTATTCACTGTGTATAGAAATATAGCCTTTATAACTGATACAATTGT

GGCTGCATGTATCTTGCACAACTATTGTCTCAATGAAAATGATCATTTGGAAGAACATGAATGATTTATAAATATTTTGC

TCTAAGCAATAAGTATTTTCTCATGGACAATATTTGTGGTTCCTTCTTTCAGCATGTAAACATGTTGCAGATTTTAAATA

AGTCTTTTGTATAAAATGTAGATTTCACAGCATCATGTTAAACAATATTTATAAGTATAAAAGTGCAATTGCTATGAATG

GTTACTATTGCCTCATTAAATCCTGATCAGCCAAAAGCTTTCTCAAAAGTGCACATTTTTCTCTTTCAATTTTAAGCATT

TCTTCTTGTTTTTTCTCTTTGAGTTGTAATTTTTTTAATGCTAATGATTGTTTAATTTCTAATTCTTTTTTTCTTAACTG

TAACTCCTCCTGTCTTAGCCTCAAATTTCTTTCATACCTCTCATCTCTCAATTGCTTTTGATCCTCTTGTTTTCTTAAAT

ATTCTAACCATTGTTTATCCAGCTCTATTTTAGCTCTTCTTTCTGTCCTCAATCTTCTGAAAGGTGTTGAGCCTTTGAGG

GTCAAATTATTTCTTTTACTTTTATCTTTATCTAAGTCTTCTGAACCACCTGGCATTACACCAGATGCTAGTCTGTAGGT

TTCAGAATTATCTGAGTAGCGTCCCAAAATTTGGTGCATTTCATTAAAATATTTAAAACTCATGGCCCCTTGTCCATTGT

CTATGCAGTCTTTATACTTTTTTGTAAGAGCATTTATTTTCCATCTGACTTGATCTGGGGTCACCTCAACACTCAATGAT

TTTAATTCCTCAGCAATAGAGAGCCACATTTTAGATTTCTTCTTTGGGTTGTCCAGCATATGGATTTTAGTTTGATATAA

ACTTAACAACAAATTAGTTGCATGTTTAGTCCAAATGACATTTTGCTTATTGCTCTCATTAACATCTGTTTCTTCGTGTG

TCACAGTTATTGTGTGACTAAAGTTATAGTTATTCATATTCATATTCGGTTTATATTGGTTTGACTGGTTATCTAAAATA

GCCAAAACGGCAACATTACTATCAATGTTTTCATCCATTATGTGTCGACAACTTGGGCGTTTTCCAACAAGTACAATTAC

AGTGCACGGTAACTTTTATACTAGCCGCAGATTGACCGTGTAGACCAACCTCTAACTTCAAAGCTTTATAATATTAAAAA

TCGAGTAACAACATGTATGATACTGCAACATGTCGTGTAATTTGTTATTGCATTATATTGATTTCATGAAATAAATACAG

CGTTCTCACACAGATATTAAACACAAAGGGCACACAGACGTCAAATTGACATTTGGCGAAACACAGTGCTGTTCTTGTCA

TTTCTATTGAGCCTATAGGGTTAGACTTCGGTTTCTTATTCCAAAGTATTATAAACTAGTTTTTCAATAATATTTACTTG

GAGATGCATTGTTTCTTAGCCTAACATAACCTAAACAAACTATGTGCAACTTTTGTGCACGATTAAAGGTTTCCCTTTCT

GTAGAAAATTGAAGAGTACTTGTTTAAATGTAATTTACAAAAAGACTTAAAAGAGACACTTCTAATGGAAAAGCATTTCG

ATATTAACACTGAACTATGTTTACTTGGCATTACATAAATGCTAGCATATTTTATTTTTGTCACTCAAAATCTTGCAATG

TATCTTCATCAATCACTTTTGTTTGTAGGTGAAGCTAATAATCAGCCGTAGACAGCATTTAGAAAAAAATCAAACTTGCT

GATTCAGGAGAACTTAGCAAATGTGACGAAATCTCGTGTTGTTTTACCTCCTTTGAAATAATGATCTATTATATATAAAT

ATAAATTTTTAATGGCAATATTTACAATATCTATCAAGTAATTTGACAGATTTGATTTGACATTCGTTTGCTCGTTGACG

TGACGTGACATCGGGCGATTAGTGCATTTACTTTAAATTAAATGAATAAATGTTACTTATTATAATCAAATTTTGTTAAC

CTTAATAACACAAAATGGCTCAATGTGGTGACTTTATTAAAAGTCAATTCCCACTCATAGATGACGAATTGATGAAATAT

GTTGAAGACATTTTGGATAGCAGTGCCGGTGAATTTGAAGACACGGAAGAAGTGTATGAGGCAGTTGGAGAAGTGCTTCA

AGGAATCTCTGAGAAATCTGAACAAGATATAAGGGATATATGTGAGAAGCTATTACACATGCTCCAGCCTGACAAGCCGG

GTAGTAGCAATGGACCAAGGAGAGTATTAGATGCCCCAGTACACTTGGCGTCCATGACTACTACTGTGACTGAAACTGAA

GATCTCAGAAGTATATGGATCAATACTAGAGATGATAACTTGAAAGTGGACGCAAAGAAATTAGAAAAGGCTGAAGCTAA

GTTACAACAGAAACAGCAGAAACAAAAGGATTCAAAGTTACCAGTGACAGCCCCAGTTCTTCAGACAGCGACGGCATCAC

AAGTTACGTCTAAGAAGGACAGTAAACTCGAAGCTAAGGGCACGAACAGGACACAGGACATTAGGATAGAGAACTTTGAT

ATTGCTTATGGTGATAGAGTATTATTACAAGGCGCCGACCTAGTTCTAGCGTGCGGCCGGCGATATGGTCTCGTTGGGCG

CAATGGGCTCGGCAAGACGACACTCCTTAGAATGATTTCATCAAAACAACTGAAGATTCCGTCCCATATATCAATATTAC

ATGTAGAACAAGAGGTCGTGGGCGATGACACTGTGGCCCTACAAAGCGTGTTAGAATGTGATACTGTTAGAGAAACATTG

TTGAAACGAGAGAAAGAAGTGACTGCCGCTATTAATAATGGATCGACTGATACAACACTATCGACGGAGCTGAGCGAGAT

CTACGCGCAGCTGGAGAACATCGAGGCGGACAAGGCGCCGGCGCGCGCGTCCATCATCCTGAGCGGGCTGGGCTTCACGC

CCGACATGCAGGCGCGCGCCACCAGGACCTTCTCCGGAGGCTGGCGGATGAGGCTCGCACTGGCGAGGGCGCTGTTCTCA

AAACCCGACCTCCTACTACTCGACGAGCCGACGAACATGTTGGACATCAAAGCGATCATCTGGTTAGAGAACTACCTCCA

GAACTGGCCGACCACCCTGCTGGTCGTGTCTCACGACCGCAACTTCCTCGACACTGTGCCCACAGACATCATGCATTTAC

ACACACAAAGGATAGATACTTACAGAGGTAACTACGACCAGTTCCACAAGACGAAGACGGAGAAGCACAAGAACCAGCAG

CGCGAGTACGAGGCGCAGCAGCAGCACCGCGCGCACACGCAGGAGTTCATCGACCGCTTCCGGTACAACGCGAACAGGGC

CTCCTCCGTGCAGAGCAAGATCAAGATGCTGGACAAACTGCCTGAACTAAAACCAGTAGAGAAAGAGATAGAAGTGGTCC

TCCGGTTCCCCGAGACGGAGCCGCTCTCGCCGCCCATCCTACAGCTGAACGAGGTCGGCTTCTACTACTCCAAGGACAGA

GTCATCTTCTCCAATGTCAACCTCGGAGCGACGCTAGAGTCCCGGATATGTATCGTGGGAGACAACGGAGCTGGTAAAAC

GACGTTATTAAAAATAATCATGGGTATCCTGTCACCTACCAGTGGTATCCGCAGCGTACACAGAGGACTGAAATTCGGTT

ACTTTTCGCAACATCACGTTGATCAACTAGAAATGAACGTCAATTCTGTAGAACTGTTACAAAGGAGCTATCCAGGTAAA

ACAATAGAAGAATACAGAAGACAACTAGGTAGTTTCGGTGTCAGCGGCGACCTTGCTCTCCAAACGATCGGCAGTCTCTC

TGGGGGTCAGAAGTCGCGAGTTGCCTTCGCGAGGATGTGTATGGGCAACCCCAACTTCCTCGTACTCGACGAGCCGACTA

ACCACTTGGACATCGAGACCATTGAGGCGTTAGGAAAGGCGATAACTAAATATACGGGAGGTGTCATCCTCGTTTCTCAC

GACGAAAGATTAATACGAATGGTTTGCAAAGAACTGTGGGTTTGCGGCGGTGGCTCCGTCACCAGTATAGAGGGCGGCTT

CGACGAGTACCGCAAAATCGTCGAGAGAGAACTCGAGGCACAGAACAAGTAGACAATGTAGCTGGAATCGTGTTATTGTG

TCGTGTCTCATCGGCGTGCCGTCACCATTCTATTCTAATTCTATAGGCTAATTCACTGAGACAATCTTCGATGCTGTTCT

CCAAAAAATTAAATTTATGCTCAATTTTTTGGGGTGAAGCAAAATTCTTGTTTATGTATTATTCAATTAGATAAATGAAT

ATAGGTAATTAAAATGCATTGCACCGTTACAGGTAACGGGTAATGCTGCATAAATAATGTAGAAGCTTCTTCTTAAAAAA

ATGAGCATGAATTGAGAAGTTTTTGTTTTTGGGAGAATTGAAGTTATCTGTCTCAACGTGTGTTGTGTGTGAAGCGATCT

CGCTTTGACGCTTTTTGATTGGTTGATATTGTGCGAGACGACGGCGCGTCGACCCGACACCACACAATACTACGGTTCTC

GCTTATCGCAAATACACACCCACATGTATACAATATATCCATGTAAAAAGTAACCTTAAATTATATTGTTGTAATATACA

CGCAGTGTTTTATTACTTGGTTGAATACTACATACTTACTTTATTATAACTTAGTGAAAAGGGAAATGAAAAACTTATTA

ATATCAAAAAAAAAA

>TRINITY_DN11134_c0_g1_i1_13928075_Cpa#DNA/MuLE-MuDRgi|13928075|emb|CAC37681.1|putativerecombinase[Chironomuspallidivittatus]

GTTAGATAGAAAAAATAAGAAAAAAGTCGTGATAAGCATGGCATTAATAACTCATTTTACCTAAAAGTGTCACTTAGTTA

CTTTTCGCTCTACGAGGACGATATACATACATACATATCTATGTTTTTACTCATCACACATTTTCAATGCTCATCGTTTA

TTATTTTTATTTAAGAATATTAAAAAAATGGATGGTGCACAGGCTTCCACGTCACAATGTGTGTCAACACGTGAAGGTAC

ATCGAGAAAAGGTGGTCGCCTTTTATTTAGAGGAGGGTATGAATATAACTGGAAAAGGCAGAACAAAGACAGCACAGAAC

TTTGGCGTTGTGCAAAGAAAAATACTTGCAGTGCTTCTATAAAAACAAAAAGGGACCCTCTTATAGTTTTACATGAAACA

TCACATAACCATGAACCGAAAAACTTAGAAACAAGGGAAATAAAACAACAGATGGAAAAGTGTACAAATACAGTTCAACA

CGATGTAAGCATTCCAATTCCTCAAATATTTAGTGAGCACATGGAAATACTTAAACAAAAAGGAATACATAATTTGCCGA

AGTTTGAAAATGTAAGTAAACATCTGTATCAGAAACGAAACAACTCCATTGGAGCCAGAAGACTTTGCTTTAGTAAAGCA

GCAGAGCTTATTATACCAGAAAAATATTTATTTTTGCTGTTTGCTGATTATCAAGACAGAGATAAACGTATATTAATATT

TGGACATAAGGATTTTATTGGCCATTTGAGGCAGTCAAGACGATTTTTCTTTGATGGAACGTTTAAGATCTGCCCGAAGG

CATTTTATCAACTTTTTACCATTCATGCTGACATCGGAAGCACAGAAGAATTATTTTGTCAATGTGATTCCTATTTTGTA

TGCGTTACTGCCTGATAAGAAGCTCGACACATATCAGATATTATTTGAAATAATAAAAAGTCAGGTACCTGAATGGGAAC

CAACTTATGCCACTATGGATTTTGAAGTAGCCATTATAGTAGCATTACAGAATATTTTTCCTGACGTTTAAATAACAGGG

TGTAATTTTCACTTTAACCAGTGCTTGTGGCGTAGAGCAAAGACTTTAAATTTAGATAAGACACAAACAGGGAAGGCTCA

TATAAAGTTGTGTGCAGCATTATCACACATTCCTAAACATTTCGTCGAAGATGGATGGCTTTACATTATGGCAGAAAGTC

CAAGCAGTGAAAATTTTACGAAATTCAATGATTATTTTGTTAATACGTGGCTAGAAGACAAGGTGCTTTCGAATATTTGG

TGTACGTATAATGAATATCACAAAACGAACAACATTGTAGAGTCATGGAACCATAAAATTAAAAAAATCATAAAAGTCAA

ACCAAACATTGGACAACTTCTGCAAGGATTAAATAAGGATGTCCAATTTTATTCGGAACAGTTGAGACAACCCGGTGGAA

TCAAAATTTCAAAAAGGAAACCGGATACTTTAGTAAGAAATTGGAAAATACAAACTACAGTGCAGGAACTATTGAATGGG

CAGATAAGTATAGGACATTGTTTAGAAAAACTAAAATTCTAAGGCATAAAAAACAATAACCTATACTAACAATATCTCTG

GTTTAACTCATAGTTTTATGCTGATTTATTTGATTCAATAAGACTGATTTGTACGTATTATAAGATTTTTTATTGTTCAA

AAGTTAATTATTTATATTTTCACTTTTTATTTGATATAAG

>TRINITY_DN11115_c0_g1_i1_Copia-6_DWil#LTR/Copia265-4233viatfastywithCopia1_DM,2stopcodons.[Drosophilawillistoni]

GAGTATGGCACTCATGAATAAAGGCTTATCTGAAAGAGAATGGAATCAAACATCACACAACGGTCCCGTATTCTCCACAA

CAAAATGGTGTGGCAGAAAGGGCAAACAGGACCATTGTTGAGAAGGCCAGGTGTATGCTAAAGGATGCCGGCCTGGACAA

CAAATTCTGGGCAGAAGCTGTCAATACTGCTGTATATCTCAAAAACCGGACACCAACGAAGGCTCTAATTGGACAAGTAC

CTGAGGAAAAATGGAGGAATAAAAAAGTAGATGTTAGTCATTTACGTATTTTTGGCTGTATTGCCTATGCTGTTACCCCG

ATTAGAAACAAATTGGATTCAAAATCCAAGCAGTATGTTTTTGTTGGGTACTGTGAAAGTACAAAAGGTTTTCGTCTGTT

GGATCCTGATAAATCATACAAGTGTGTCAAAGCACGAGATGTGATTTTCTTGGAGAATAAATTTTTCAATAATAGGTCAC

ATGATGATAATTTTGACTCTGTGTTCATCGAATTTCCTCAGAATGGTGCTCCGGGGAAACCTGTGAATGTTGTTCATGAC

CCAGAATCAATCCTGGATAATGACCAATGCTCACAGCATATTGAACGATCTGAGCCAAGCCAGTCTGAACCATCCACCGT

AAGACCAGAGTTTTCTGATGACCAAAGGAGTAGAACACGGTCGAGCATAATTACAGTGCATGATTCAGACAGTGAGGTTG

ACACCACTTTAGAATCAAATTTTTCTGCGGATCCCACATACGTTCCAGGTAGCTCAACATTGAATGAGACGACATCAGAT

TCATCTTTATATGAAGATCTCGATGAGACTGGGATGACTGTCCTGTTGGTTAAAGAACTATGCAACGACGATGATGTACC

AGGAACGGTGCAAGAAGCTTTGGCAGGCACGGAATCAATTGAGTGGAAAACAGCTATGACAGATGAGTATAAATCTTTTA

ATGACAACAAATGTTGGACTCTAGTAAAGAAACCAAATAATCAGAAACCAATAAAGTGCAGATGGGTTTTTAAAAAAAAG

AAAGGTCTGAATGGTGAACTCTTGAGCTACAAAGCACGGCTGGTGGCTAAAGGTTACTCGCAGAAATTTGGAGTTGATTA

TGAAGAGACTTTCTCTCCGGTGGTAAGACACTCAACTATTAGAACTTTACTTGCCATTGCTGCTGAATTTAATATGGACA

TTGATCATCTTGATGTTAAGACTGCCTTTTTGAATGGCAACCTGAATGAAACCGTCTATATGGAACAACCAGAAGGTTTC

ATAGTAAAAGGTAAAGAAAACTTTGTGTACAAACTACATAAAGCTATTTATGGCTTAAAGCAAGCTTCAAAAATGTGGTA

TGAGAGAATAAACGAAGTATTATTACAGAAAATGCACTTCAAAAGGATAACTTCAGAACCATGTGTATTTTACATGAGAA

GTAATAGTGATTTAATAATAATTGCACTCTATGTCGATGACATTTTATTGTTTTCTAGCAATTCAACTTTGAAAGAAAAG

GTGAAATGTGAACTTATGAACACTTTTAAAATGAAGGATTTTGGACCTGCACACCACGTTCTTGGAATGAGAGTTAATAA

AAGTCAAAATAAAGTTACACTTGATCAAACTGGATACATAAAAAGGGTATTGGAGAAGTTTAACATGACGGATTGCAAAC

CTGCAAAAACTCCACTAGAAAAAGGAATCAAATTACCTAAAGGTGATAATAAAAGTACAAATTCTCATTACAGGAATTTG

CTTGGCTGTCTCATGTATATAGCGGTGTGCACCCGACCAGATATCGCTCATGCTGTGAGTGTGTTCAGTCAATTTAACGA

GTGTCACACTGAACATCATTGGAAAGCCTTGAAGCGTGTCCTTCGCTATCTTAAAGGTACTGCTAACTATGGATTAGTTT

TTCAGAAGAGTGGTATGGATGTAACAGCTTATGTAGATGCTGACTGGGGTGGAAATGAACTTGATCGTAAGTCTTTCACT

GGTTTTATTTTTAAGCTTGGTAATTCATTGATCTCTTGGGAAAGTCGCAAACAAAAGACTGTTGCCCTCTCCAGCACAGA

GGCTGAATATATGGCACTTTCAGATTGTTGTAAGGAAGCTCTTTTTGTAAGAAGTTTTCTTAATGAATTGTTGAACAGTA

ATTGTAAAGTAACATTGTATAATGATAACCAGTCTGCTCAAAAGTTAACTACTAATTGTATGTATCATAATAGAACTAAG

CACATAGATGTGAGGCATCATTTCATTAGAGAAAATATAAAGAAAAACATTGTTAATGTTAAGTATTTGTCTACAGATTT

AATGATTGCAGATGTGTTGACAAAACCTTTGACAAAAG

>TRINITY_DN11115_c0_g1_i2_Copia-1_BM#LTR/Copia159-4034[Bombyxmori]

ATTTTCTGATATTCATTGACGATTTCAGTAGGAAAACGTTTGTTTATTTTTTGCATAATAAAGATGAAGTTTTTGAACAT

TTTAGGAATTTTAAAAACTTAGTGGAAAATGAAACTAACCACAAGATTAAAATTATACGCAGTGACAATGGTGGTGAATT

TGTTAACTGTAAATTACAGGCTTATCTGAAAGAGAATGGAATCAAACATCACACAACGGTCCCGTATTCTCCACAACAAA

ATGGTGTGGCAGAAAGGGCAAACAGGACCATTGTTGAGAAGGCCAGGTGTATGCTAAAGGATGCCGGCCTGGACAACAAA

TTCTGGGCAGAAGCTGTCAATACTGCTGTATATCTCAAAAACCGGACACCAACGAAGGCTCTAATTGGACAAGTACCTGA

GGAAAAATGGAGGAATAAAAAAGTAGATGTTAGTCATTTACGTATTTTTGGCTGTATTGCCTATGCTGTTACCCCGATTA

GAAACAAATTGGATTCAAAATCCAAGCAGTATGTTTTTGTTGGGTACTGTGAAAGTACAAAAGGTTTTCGTCTGTTGGAT

CCTGATAAATCATACAAGTGTGTCAAAGCACGAGATGTGATTTTCTTGGAGAATAAATTTTTCAATAATAGGTCACATGA

TGATAATTTTGACTCTGTGTTCATCGAATTTCCTCAGAATGGTGCTCCGGGGAAACCTGTGAATGTTGTTCATGACCCAG

AATCAATCCTGGATAATGACCAATGCTCACAGCATATTGAACGATCTGAGCCAAGCCAGTCTGAACCATCCACCGTAAGA

CCAGAGTTTTCTGATGACCAAAGGAGTAGAACACGGTCGAGCATAATTACAGTGCATGATTCAGACAGTGAGGTTGACAC

CACTTTAGAATCAAATTTTTCTGCGGATCCCACATACGTTCCAGGTAGCTCAACATTGAATGAGACGACATCAGATTCAT

CTTTATATGAAGATCTCGATGAGACTGGGATGACTGTCCTGTTGGTTAAAGAACTATGCAACGACGATGATGTACCAGGA

ACGGTGCAAGAAGCTTTGGCAGGCACGGAATCAATTGAGTGGAAAACAGCTATGACAGATGAGTATAAATCTTTTAATGA

CAACAAATGTTGGACTCTAGTAAAGAAACCAAATAATCAGAAACCAATAAAGTGCAGATGGGTTTTTAAAAAAAAGAAAG

GTCTGAATGGTGAACTCTTGAGCTACAAAGCACGGCTGGTGGCTAAAGGTTACTCGCAGAAATTTGGAGTTGATTATGAA

GAGACTTTCTCTCCGGTGGTAAGACACTCAACTATTAGAACTTTACTTGCCATTGCTGCTGAATTTAATATGGACATTGA

TCATCTTGATGTTAAGACTGCCTTTTTGAATGGCAACCTGAATGAAACCGTCTATATGGAACAACCAGAAGGTTTCATAG

TAAAAGGTAAAGAAAACTTTGTGTACAAACTACATAAAGCTATTTATGGCTTAAAGCAAGCTTCAAAAATGTGGTATGAG

AGAATAAACGAAGTATTATTACAGAAAATGCACTTCAAAAGGATAACTTCAGAACCATGTGTATTTTACATGAGAAGTAA

TAGTGATTTAATAATAATTGCACTCTATGTCGATGACATTTTATTGTTTTCTAGCAATTCAACTTTGAAAGAAAAGGTGA

AATGTGAACTTATGAACACTTTTAAAATGAAGGATTTTGGACCTGCACACCACGTTCTTGGAATGAGAGTTAATAAAAGT

CAAAATAAAGTTACACTTGATCAAACTGGATACATAAAAAGGGTATTGGAGAAGTTTAACATGACGGATTGCAAACCTGC

AAAAACTCCACTAGAAAAAGGAATCAAATTACCTAAAGGTGATAATAAAAGTACAAATTCTCATTACAGGAATTTGCTTG

GCTGTCTCATGTATATAGCGGTGTGCACCCGACCAGATATCGCTCATGCTGTGAGTGTGTTCAGTCAATTTAACGAGTGT

CACACTGAACATCATTGGAAAGCCTTGAAGCGTGTCCTTCGCTATCTTAAAGGTACTGCTAACTATGGATTAGTTTTTCA

GAAGAGTGGTATGGATGTAACAGCTTATGTAGATGCTGACTGGGGTGGAAATGAACTTGATCGTAAGTCTTTCACTGGTT

TTATTTTTAAGCTTGGTAATTCATTGATCTCTTGGGAAAGTCGCAAACAAAAGACTGTTGCCCTCTCCAGCACAGAGGCT

GAATATATGGCACTTTCAGATTGTTGTAAGGAAGCTCTTTTTGTAAGAAGTTTTCTTAATGAATTGTTGAACAGTAATTG

TAAAGTAACATTGTATAATGATAACCAGTCTGCTCAAAAGTTAACTACTAATTGTATGTATCATAATAGAACTAAGCACA

TAGATGTGAGGCATCATTTCATTAGAGAAAATATAAAGAAAAACATTGTTAATGTTAAGTATTTGTCTACAGATTTAATG

ATTGCAGATGTGTTGACAAAACCTTTGACAAAAG

>TRINITY_DN11132_c0_g1_i1_Gypsy1_AG_pol#LTR/Gypsy783-3872[Anophelesgenus]

ACACAAGTAGTACCCATATCTGGTGACCCCGACGTGATCATTAATTAGTACCGTCGCATTATTTAATTAGTCATGGGTGA

ATTTGACGACGCTCTAACAGGACAAGAGAAAGAAGTTTCCGCCATTTCGCTGCAAGCGAAGATCCCGAAATTCTGGCGAG

CACAACCCAGGCTGTGGTTTGCTCAGTTCGAAGCAGTGATATCACCATACAAGACCAGCGATGAACGAAAATACAACTTG

GTGGTTGCAGTATTGGAGAGGCAAGACATCGAACAAGTTAGTGATATAATCTGTAAACCACCAGAGTCAGGAAGATACAA

CGCATTGAAAAGTCGTTTGCTATCAGTGTACGAAGAGTCCGAGTCCCGTCAATTCCAGAAGCTTTTGAGCGGACTAGAGT

TAGGAGATCAGAAGCCTAGTCAACTCCTGAGGGTGATGCGAGAGCTGGCTGGAGAAAAGGTACAAGATAACGCCCTCAAA

ATATTATGGATGGGTCACTTACCTTCCCAAGTTCGTGCCGTACTATCCGTGAACACGGAATCTGCATTAGATACCCAAGC

GTTGATGGCGGATAAAATGATGGAGCACACGGAACATACGATCGCAGAGGTCAACCAATCTGTATGTGCACCTACTCCAA

GTACCAGTACTGGTCCAGACTTGCAGTTCCAAGTTTTAACAAAACAACTTGAAAAGCTGACTCTTGAAATTGCCGCACTG

AGGAAGGAGAATGTGAACCACCGACGCCCATTTCGCAGAGACCACAGCCGTCCGAGGTCCCAGTCCCGCACCCGAACTGG

ACCAGGAAAGAAACCAGGAGATCCAACCTGGGAATGTTACTATCACCATCGGTTCGGACACAAGGCAAAAAAGTGTGAAC

CACCATGTGCAAGGAGGCCGTCGGAAAACTGAAAACCACATCGGCTATGGTGGGTGTCGGTGTGCCGAAAATTAACAACC

GCCTATGTATAACAGACCGCAACTCAAGAGAACGATTTTTGGTGGATACTGGTGCAGACTTATCGGTTCTAGCTGTAAAA

ACTAAGCGAACACCGATAAATACACAGTTTAAATTATTCGCCGCAAACAATACACCTATTAATACATATGGAATGAAGAC

ATTACAATTAAATCTTGGACTTCGACGCAACTACAAGTGGACTTTTATTATGGCAGATGTGAAGACGTCAATTCTTGGTG

CTGATTTTCTCAAGTACCACAGACTTCTGGTGGACCTACACAGGAAAAAGCTCGTAGACAGTCTGACCGAACTTTCAGTA

GACACGATGACCATGAACACTACGCAAGATACTACACTGTACGTAGTTGAACCAAGTCATCGTTATCATGCACTTCTACA

AGAATTTACTGACATCTTGAAACCTATGTCGCCTATGAAAGAAATCAAGCATAATGTGAAGCACCATATTAAGACCACCG

GCCCTCCACTCTTCGCAAGACCTCGCTCACTGCCGCCGATAAAGTACAATGCAGCAAAAGCCGAGTTTGAAGCAATGATG

TCAATGGGAATATGTCAACCATCAAAAAGTCCGTGGGCCAGTCCCATGCACGTGGTAACAAAAAAGGATGGAACTCTTCG

CATATGTGGTGATTACCGTCGCCTAAATTCAGTAACACTTCCAGACCGCTACCCAATACCGAGGCTGCAGGAATTCACTT

ACCAATTACATGGCATGAAGTTATTTTCGAAGATAGATCTGAAGACAGCATTCTTCTGGATTCCAATTTCTGAAAATGAC

GTAGAGAAGACAGCAATAACGACACCTTTCGGACTATTTGAGTTTCGTAATATGCCATTTGGTCTGCGTAACGCGCCACA

GACATTTCAACGATTTATGCATGAAGTCTTGAGAGGCCTAGATTACTGCTGCTACTGCTTTATCGATGACGTGCTTCTGT

ACTCAAAAACACCACAGGAGCATGAAGACCACTTGCGACTTGTTTTGAAACGTCTGTCTGACTACGGAATCGGTATCAAC

CTAGAGAAGTGTATGTTTGGTCAAAAGAAGCTAAACTTCCTGGGCTACGAGGTTACAGAAGATGGCATCACGCCTACTGC

TGAGCGCATTCAAATAATCCTAAACTACCCGAAACCAGCGAATGTTCAAGATCTCCGACGTTTTCTCGGAATGGTAAATT

TTTACCACGACTGTTTACCTAAGCAAGCCGAAAAACAAATAGAACTGAATAAACTACTTCACAATAAAAAGAAGAATGAC

AAGACGCCTATCACCTGGAACCAGAAGTTAGAAGACGCTTTCGATAACTGCCGTCAAAGCATATCGAAAGCAGCACAATT

AAGTCATCCTGTCCAAGGTACCCCATTATGCATCATGACAGACGCCTCGGACTTGAGTGTTGGAGCAGTCCTGCAACAGA

AAATAAACCACACATGGAAGCCGTTATCTTTTTTCTCGAAGAAGCTCAGTGACACGCAATCAAGATACAGCGTATACGAT

CGCGAACTATTAGCTGTCTATATGGCTGTCAAACATTTTAGAAGACTGATTGAAGGAAACGATGTTGTGGTGTACACGGA

CCATAAACCCCTTACTTACGCCATGACCAAATCACCAAGCGCAAGCGACACACCAAAACGGGAACGGCAACTTCATTTTA

TAAGCCAATTCTGCAGCAAGATTGAGTACATACACGGAAAAGACAACGTGGTTGCCGACGCACTATCTCGTATAACTGCC

ATCGACTGTCCTTGCGTTATCGATTTCAATAATCTGGCCGACGAACAAATAAAAGACGAAGATTTAATTTCCTTGTTGAA

GCAACAAAATCTTCACTTCATCAAGATATCCCTGCCTGGCGTAAAGAAACAAATCTACTGCGAAAATTCAACAAAGTCAC

CACGTCCTTATTTACCGAAATCTTTTCGTGAGATTGCCTTCAAAGCACTCCATGAAATGAGTCATCCCGGTATCCGGACA

ACTAGAAAAATAATTACTGAGAAGTATTTTTGGCCGGCAATGAATAGAGACGTATCTGAATGGACACGATGCTGCATTAC

ATGTCAACGTGCTAAAGTTCACCGGCACACGAAAACACCGCTTGTACAGTTCCCCGAGTCGAGGAGGTTTGAGCATATAC

ATGTCGACATTGTTGGACCCCTCGCCCCATCAGAAGGATACCGATACTTGGTCACAATCATTGATCGATGCACAAGATGG

CCAGAAGCAGTGCCAGTCAGCGACATAACCGCGGAGACCGTCGCCCGAGTCATATATGATACATGGATTACTCGCTTCGG

TTGCCCACTGCGTATTTCAACGGACCAAGGTCGACAGTTCGAGTCATCATTATTTCAAAAACTAATGAATAAACTCGGTG

TATCAAGAATCCGCACAACAGCCTACCATCCTCAATCAAACGGGCTCGTCGAAAGATTTCATCGTACGCTCAAAACAGCA

TTAATAGCCAGAGGAAATAAACAACAATGGGTACAAGAATTATCTACCGTATTATTTGGATTACGTACGGTGGTCCATAA

AGAAACAAACTACAGTCCCGCTACGATGATTTATGGCACCTCATTAAGATTACCGGCAGATGTTTTCACACCTGCGAAGA

TAGATAAGGAAGACCCGTTATTGATACGTCACTTAACGGAATCAATGGCAATGCTTAATGAGTCATGCGCTCGTGCACCC

GCTCGACAACAACGCACGACATTCATACCGAAACAACTGGAAACATGCACTCATGTATTTATGCGGGTAGATGGTGGAAA

AAAATCATTATCACCGCCGTATGAAGGCCCGTACGAAGTTGTTGAGAAACATGACAAGTTCTATAAGATTCGCTTGCCCA

ACCGAGAAGCGGTGGTATCATTGGATCGATTGAAGCCTGCCTACGTACTGCCAGCAGCCACAGAAGAAGAAGTGAAAGAA

AACCAATATGTTACCCGTTCCGGCAGAACTGTTAAACCTACAAAATTTGTACGGTTTTCTTGAGGGGGGATACTGTGGGA

GTCCGTCATACAACACCGATCATAACGACCGCTCCGCGCGGCGCGCGGACGGACACTGAACGCTGCCGGCCCGAACGGCC

GTATTTATACCTCGAGTTCTCGGCGACGCGTCATCAACGGACATACTCCTGCGTCGATCGCCTTCAACTAACTGAGCGGC

CGATGCAGCGCAACTAGTTTTTATTAAGCGTGCCGAACCCTGATTGGCTGCACACGTTGTGGACATATCGACAGCCAATG

GGATGTTAGAGACTCTTAGGATGTTGGTATAAATATTGGTTTCGAAATAAATTTCACAGCATTCTCAAAAAAAAAA

>TRINITY_DN11156_c0_g1_i1_PiggyBac-14_SM#DNA/PiggyBac379-2016[Schmidtea]

TTTTTTTTTTCTTATTAAAACAATATATTTATTTACAATGACACATGTTCTTCAGGTACAAATAAAATAAAAAAAACAAT

CACAACATGAAGACATTGTAATAGCTACAGACTGTTATTTATATCATAATTATGTCGGCGACTTGTGTCAGGTGTCAGGA

TAGCCGACATCGGGATAAATTAAACACATATTCAAGCCTCATTATCTCATTATCGTTTCTTCCTAGCCTAATCTGGACAT

ATTACGAACAACGCCCGCCCGTCACGTACCGCTTGCTAAGAGTTCTAGAGAGAACAAACTCCCCAGCCTCTCGCTGTTCA

ATCATTACACATTATTTAGTAACTTTACACTATCTTATTAACACCACAATCGCTTACACGTTTCAATAATTACTAACTTT

AATATAACGCAATAATTAATTAGTCTCGTGACATCTAATCCACTCTTAAAAATTCAAATATCCCCCGCTTCTACTTTCCA

ACCTGTCATTTTTGGCGCGAATAGTATAATAAATAACTATATAGGTTTCATGAATGCGGTTGCGGACTTTTTATCACAAA

TGAGGTTTGAAATAAGAGAGATTTAGCTATTTATTCTTATGACCATCTCCGACATAAATAACGTTTTTACACACATCATT

CATCACCTTGCACTCTTTTTTCAGAACATACCGATCACAAGAGTTATTTATGCATAATTGGGAAGAATTGAAATGGAAAG

GAAAGCATAATATATAAATACTATAGTTTTTGTGATGATTGTGTATGGTATGCTTTGAAACAATCCTTTATACACAGTGG

CACCTTACAGGACTCGCAACAATATGGTGTCTTTTGTGATTTAAGGCCATTCTGAAGGCAGACTACGCAACGCTTCGCGA

AGTTGCGATTGCGCGCTATGCTGCTCTCCGTCGGTTCCAGTAAAACGGGCCAATGTGTAGAACGAATAAATCTATCGGGT

TGTTGGTGCAAAGATCTACTGATGGAAGCAGTATGACTACGGCTTAGAGGACAATGAGAAAGATGTTTTTGGAGGATGTA

ATCTACAAGGTGAAGGCGGAAAGCTAGATGATCGTGATGTTTTTTTGTGGATGACTGCAGAAGAGTGCGAGAGTTGAGGA

TAGAAACATTGATAAGACGTTTAAAAAGTTTCATGTACCATTTAGCACAACGTTTCCTTTCTAGTAAATAAGGCTCGAGC

ATTTGGTCCTTCATATCTACCCCCCCCATGAACTTATTGTAATCCAGTACCACTTGCGGTTTAAATGGCACTGGCCGAGA

TGGTGGTCGTGTTGGAACTGTAGGCAAGGCAGTATATGCACCATGACAAGTAGAAATCATGGTTACTCGTTTCTTGTCTT

GCCACGCTAATATTGAGACATCCCCGGAATGTCTCGCAATGAATTCTCCCCTTTTAAGCGGAGCCTTATTTACTATTAAG

GGAACGTCTCTACGTGAAGGTCTCAAAGTACCGACGCAGTCGGTGCCATTGAGCTTCAAAAACCTAGCTAACAAGGGAGA

ATTATACCAATTATCCATAAATAATCTATAACCTTTGTTTAGTAGGGGACCAATGAGCTTCATCACTACAGCTGTGCTTT

TAAGCACCCCGGGCGACTGCTCCAACTCCGTCGCGGATTGTTTACCGGTGTAAACAATAAAAGACCACAGGTAGCCGGTG

GACGCCTCGCAAAGCTCAAAGGTTTTGATACCAAACTTAGAAGCTTTGGTCCGAATATACTGTTTAAAGTTTAATCTTCC

TTTCCATAGGGTTAGACTTTCGTCTATGCAAATATTTTTATCTAAAACATAATTTGATTGAAAACGCGAATTTAAATGTG

TTATTATAGGTCTCAAACGGTGTAATCGGTCATAGCACCTAGCGCCAGTATTGGAGGGATTTGAATTGTCGACAACGGGG

TTGGAATCTACAAAATGTAACGCTCTAATAAGCATTTCATACCTCCGTCGTGTAAAGATGCGCCTGAAAACTTCTGTTTT

CAGAAGAGGATCGACAGAAAAACAACTGACTATAGTTGGCATTTTGACGATCCCCAACATCATCCAGAACGAAAAGAGGC

ACAAAATTTCGTGCAAGTTAGTGGGGTACCATTCAGACTGGAAACCAGTAGATGCAATTGTCGTAGCATACTGGTTCGTT

TCAGTCACAATTAGGTTTAGGACATCATCGTCCCAATAGGAACGAAAAGCGTCATAAGCTGAATTGAACACACGGACCGA

ACCAGTACGCTCTACTTTAAAATCCTCTTCGTGACCTTGAAAGGTATCCATAGGAAACCATTCAAAGAGAAGGTTCTGGC

CTTCCAGTCGTGGGAGATCATCTGCTCCATTACTAGCGTCGAAAAATTCATCATCACTATTGCTCTCGCTAGCACTCTCT

AATTCAGCGTTTCTCTCAGCATCTTGCATACTCGTCTGTCGTTTCCTCAATACATCCGTCGCCAACCTTAACAATTCGGG

GGGTACGTCTGCATCGTCTACCCATATTTCGTTAAGTGGGTATCCCAGGACCCACTGACGAGCCCTTCTTCGACCCTGGC

TTGGATACCCACGCGCACTCCGTCCTCGCACACTACCTGTTCGTCCTCCTCTCCGCCCAGATCTACTTCCATGTCTAAGC

TCATTCTCTCTCCTTTCATTTCTATCACGCTCCACAGGGGACACATACAATGCATGGGTATCCGGGTCTAAAAAGACGTC

ATCGCCGCCGTCAGCGCCCAGTTGCGTACCATTTTCTTCGTAAAGCATGACGACAACAGGCATAGTCGCGATATCCGACG

AAAATATAGGAGACCGGGACCTGTCTCGGGACGCTATGCGCGATGGCGGTTGTTCATACATTGACGGCAACCTGTCGCGG

CGCTGTGGGGATGGGAGAGGAGAGTACGGTGGAGCCTCCGGCGAGTGGCACTCATAAACCTCCTCTTCAATGATTTCTGA

TTCATCACAGATCTCTTCATATCCACTCGGCTCCTCTTCATTGCCACTTTCATCTTCAATGTCATAAAGAACCATGTTGA

AATCCTGAGGAGCCTCCTCACTGGGACGCCAAGTTGACATTCTCTGGAAAATTTATGAAATCCACAAAAAAAAACAAAAT

TTACCACAACACCGCGCAGCGGTCCGCCAAACCATAGAGATATATTATACATGGAAACTGGAGTGAGTAAATTCTACTGT

ATAAATAATACCTATACAAATGTATAACATATCGACGCGCATATGACAAAAAGCGTTTTGTCTCTTCTTAGCTAACCAAG

AGTTGCATATGTAACGAGAGATATCTAAATGTCTGACTCGGTCCAAAATTCCAAATTCGG

>TRINITY_DN9677_c0_g1_i1_Sola1-3_AA#DNA/Sola400-2851ViagenewisewithSola1-2_AA,2introns.[Aedesaegypti]

ACTTTGTAAGTCTTAAAATGTATTAATTTAAATGCAAGTTCAGTCTTTACTCATCACAATCATCTACATCTAAAATATTA

ACTAGATCAATTGAATTGTCATTGGTATGCAAGTTTTGCCAAAATGGTAGTCTATTTTCTGGAATAACTCTAGTAAGCGT

TGAAATAATTGCAGATTTTCTAGCTTCAGTGATACCCCTAGGACTTGAATTTTGTATGGGATCGGGAATTGAATGAGACT

TTATGTGTTTCATTTTTAGGAACCCTATTTCTCGGAGTTCACCATCATGTTCGGTCTTTACAAATAAATTAAAATTATTC

CTACGCACCTCGACACTAACAATATCTTTCAAATATATCCGATTTGTGCTCTTTTTCAATTTATACTGCGATGAATGATC

AATATACTTGTAAAAATCAGTAACTTCCATAGGTTTCACTGTTACTTTTCCGGAATTAGCTAACTGAATGCAATCAATGA

AGTCTGAGAAATCATAAACTTTTTTTGCTCTTTTTAATTGTTGTTCAACCTGGTGGTGAAAGTTATCGGCTGACATAAAA

GTATGTCCTGATTCAAGATATTTAATGGTAATCGTGTTGGCACATATCAAAGAGCTGTTAATTATCTGAATAATAAATGA

GAAAAATGCCCAATTCTTATTCTGGCTGGAACAGTTGTCGGCCCATACTACTATGTCAGTACTGTCGCGTTTGCAGATGA

AAAACGCTCTAAAAGCACTTATTATATCTTCTTGTTTTCGTCCTGATATGGCTTCATGCCATATTGCGGCGAATGGCTGA

GAGGTTGACTTAGTAGGCCCCAAAGGTACAAAACTCTCGTTGAAAGCTATTATTCTGGGACAAAAAATAACAGCTTTAAA

TATATCAATACGTGGCAACATGATGACTTTTTGCAAGTCTGCTGAATAAAAAGCTTTTTCTGTGAATTTGGTCGATAAAT

CAACATCTTCTTGATAACATTTCCGTGACTTCTCGTATTTTTGCTTGTGTAGTTTATATGATATACAGGTATCACAGGAT

GCGGAATCTTGACATGTGGGCTGGTGTAACTTAAAACTTTCACAGTCCTCGCATTCTTCATTGCCCAAAAGAGTAAAAGA

AATATTCATGTCCTTTTTTAAAAAATGTCTGTATAATTCATAAGAAACCTTTATATCAGGGAATTTGTCTTGGAAGTCTT

TAAACATTAAAGTCGCATTTAAATCACGTAGTGCATGCTGGTTTAACACAGAACTTTTCATTTTTTTTTTGTTTTTTTGC

TTCTTTTCTAGTGTTTGTAGATTCTGTATACAGTTTCCTAATTCTTGGTGTTCCTTTTTTTGTTGTGCCCTTTAAAGCAT

CACACACTAATCTGTTTGCTGCTTCATTATCTTCATTTTGTATAACTGAATCAGTTACTAGAATTTGATTATTCGATGTT

GTTGGTGCATTATCTACGTCCGGACAGCTTTCGTTACTTCTCTGGTTTTCGATCTCTTGAAGACCTTGCGAGGTGGTAGG

TTCATCTTCTGTGCCAAAATCAGCAACTGGACTGCCCATAAGAGAATGTGTCAGTGTCTCATTACACAACAAACCAAAGC

TTTCATCTTTGGAAAAAGAATAATCTGAAACTCGTATATCTCCCAATGCAGCTGATGACAAGTTTAATGAGAAATCTGCA

TTAACCACGTCCTGTGATGAAGCGATATCCTGACGATCCGTAAAAATTTCATCCTCAGATTTTTTAGTATCCTCAGGTTC

GATTTCAATAGTTAACGTACTTGTGCTCAGCTCAGTATTCCGCACAGAATTATCGTGGCAGACGTCAGTGTTTACTACCA

TATGTACTAATTTTCTAGCTCGGAAGTTGTTCGAAGCCATTTTACCAAGTAAAACGATAACAAGCCAATTGAGATCTGCA

A

>TRINITY_DN16677_c0_g1_i1_Yabusame_BM_tp#DNA/PiggyBacgi|41016738|dbj|BAD07480.1|putativetransposaseyabusame-W[Bombyxmori]

GGGGCTCACGCCAAGGAGTGATAAGAAATGTGTGGATCCACGAATGGATTGGCATCGGGTTCAGGCAATTTTGGACATGG

TTCAAGACAATGAAGTCATTGATTCTCTTCAGACAAAGGAAGAATGTGACGTTTTGCATCAATCTGATCATGACTCTGAT

ACAGAGGCTGAACTCCAACCAGACATTGATGATGACTTTGTTGACTGCAAGAATGGTTACATTTGCAAGAATAATAAAGT

TATATCAAAAAGTCCGAAACTGTCAACATCTGTGAATAATGCTCGAATAAAAGAAGCTTTTAAGATGAGGAAGCGCAAAG

CCGATTTTACTAAACATTGTGCTCGACCTAAAAATGCGTGGCAGTTGTTATTCACTGATGACCTACTAGAATTGATTGTC

GCTTCGACTAATGAAAATATTATGACGAATGGGAGAGGTTCCGAGTTAACTGGAGTCAGCGAAATAAAGGCGCTTATCGG

TATTCTATATCTGCATGGTATAATGCGACCTACACATGAGAAATGTAGTGACCTTTGGAACAGCGACTGTGGTGTACCTT

GCGTCAGAAATGTAATGAAATATGAGCGATTTAAATTTTTACTTCAAAACATCAGTTTCGATAAAGAGGACGACGACAGC

ATTATTCAGTTCGACATAATGAAGCGTATGCGTAAAGTGTTCGAGATCTTCGCCATGAATTGCAGAACGTCTCACGAGAT

CGAGAATATTGCGGTTATTGATGAGATCATTGTGCCTGTTTACGGACCTTGCCCATTTCGATATGATATTGATAAGAAAC

CGCTGAAGCGAGGTATTAAAATGGTATTGCTAGTCGATTCATCCACGTTCTATATGAGCAATTTAGACGTCATTACGGAT

CCCTACTTTGGCGCCGAAGAGATAGCGAAAAAGATGGTCCAGCATTTAGCTGGGACTGGTAGAACGATTATTATGGACAG

TTGGTACACGTCTTCTACATTGATGGAAAGTCTTAAAAATGAGTATCAGCTATTTTCAATTGCAGCTTTGAAACCGAATA

GTGATATAATCCCACCTTTATTCTTATCTCAGTATAGGAAATGTCGAACATACATGTCTGGTTTTATCGATCACGAAGTA

TCACTCACATCATATGTCAATAATGAAGGAAAATCCATCAATGTGTTAACCAACGAGCCAAAATACTACAGGAAAGGCCA

TATCAACCATACTACAGTTGTGTCTGTGTATAAGAAGAATCAGTCAGCTGTGCAAGTGGTTGATGTTGTTATGAATTACT

ACACTACAATGCAGCACACAAATGATTGGACACTGTCCCTATTCTTCGCACTGTTAAACATTGCTTCCATAAACGCTCAA

GTCATGTGGTGCTCTCAGAACTCGAATGTGACAAACCGACGAATATTCATTAAAGACTTAGCTCTGAGCTTAATGGAACA

GGACCATGAAAGAATTTCAATATCTCCTATAAATGATCTTGAAGAGAAGAAGAATAAGTATGCTCTTTTACAGATGCAAA

ATTATTACAAAAACAGGCGAAGATGCAAAATTTGTGTGAAGACAACGAAACGCGATCGGAGAACTAAACAGTTCTGTATG

AAGTGCGGCCAGTACATGTGTAAAGAGCATTCTGTCAACATCTGTACTATGTGTGCTCATTGATATATTTTTAAATGTTA

TCTGAAAGTCAAAGTTATTTCGTTATATTATGCTTAACGCGTTTTTTCTTTGGTGAAGTGTTTTATATATATTAGTCATT

GATTATTTTATTGTTTTAAAAACAATTTAAGTTGCTTAATCAATAAGTGCGATTGGTAATTGTAGTTTAT

>TRINITY_DN9626_c0_g1_i1_Copia-1_BM#LTR/Copia159-4034[Bombyxmori]

TGCAGAACGCGGATCTCGTGGGTACCGGAGCGGGCCTTCGCAGGCTCCTCGGTCCAGGATACGATGAGTGGGCATTCGCG

GCCGAGAATTTCTTGATTTTGGAAGGCGTCGACATCATCAAGCAAGAAGCCGGCAATGGCGGCAATGCAGTCACCGTCGA

CGAACAAAAGGCAAAAGCAAAATTAGTGATGACAATAGATTCGTCGTTATTCGTACATATCAAGAATGAAAAAACTGCTT

TGGACATATGGAAAAAACTCAAGACGCTATTCGACGATTCTGGTTTCACCCGAAGAATTAGCTTGTTACGAAATTTGATT

TCCATACGCCTCGAGAGCAGCGAATCAATGACGGCATACATTACGCAGATCATAGATACGGCGCAAAAATTAAGAGGAAC

GGGTTTCGACATTAATGAAGAATGGATAGGATCGCTATTGTTGGCAGGATTGCCGGAAAAATTTTCGCCCATGATTATGG

CGATCGAACATTCTGGGTTAGCCATATCTGCTGACGTCATAAAAACAAAATTGTTGGACATGAGCGATAATGTTGGCAGC

AGTGAGTCCGAAAGCGCATTTTTAGCGTCTAAAGGTTGGCAGCGCCGTAAAGGAAATAAAGTTGTCAAAACTGTTGATAC

GTCAAACCGACCTGTCAAAGTGATTAAATGCTATAAATGTAAACAAACCGGTCACTTTAAAAACCAATGTCCGCAAACAA

AGGAAAAACAAGTAAATGCGTTCAGTGCAGTATTTCTTAATGGCAGTTTTGATAAGCATGAATGGTACATTGACTCTGGT

GCCAGTAGTCACATGATGATCAGCCAAGACAACATAAAAAATGTGTCATGTAACCTCACTACAAAGGAGATTATAGTTGC

AAACAGATCAACTATGCCTGTATTGTGCTCTGGAGATACACAAATTACGACTGTTGTGAACAACAAGGAATTTGATATTG

TTGTCAAAGATGTGTTGTGTATTCCCACACTCACCACCAACTTATTGTCTATCAGTCAGCTGATAAAGCATGGTAACAAA

GTCAGTTTTCATCAGGAATGTTGTTACATCAGAAATCAGCAGAATGAACTGATTGGCATAGCACAGTTGGTAAATGGGGT

CTACAAGTTAAACACCAGGTCAGTATGTTTGTTCACGGCTTCAGCAACAACAACCTCAAACGTAATTTGGCACAGAAGAC

TTGGACATATAAATAGCAAAGACATGAATGACATGAGAGAGGGTGCTGTTGATGGCTTAGAATTTGACAAAAAAGCTGAG

ATCAGCAAGTTCAATTGCACAGTATGCTGCGAAGCCAAACAAACCAGACTGCCATTTCCACATAGCAGTAATAGAAGTAT

GCATGTCCTCGATTTGATTCATGCAGATGTGTGTGGGCCGATGGAAACCAAATCACTCGGCTCATCCAGATATTTCTTGC

TATTTGTGGATGATTATAGTAGAATGTCATTTGTCTATTTTTTGCAGAATAAAAGTGGAGTCCACTCCTGTTTTAAAGAA

TTCAAGATTATGGTTGAAAATCAGATGAATTGCAGTATAAAAGTTCTAAGGACAGACAATGGCACAGAATTTTGCTCAAA

TGAAATGAAGAACTATTTAAAACAACATGGCATAATACATCAGCGCACCAACCCATATACCCCAGAACAAAATGGGATGT

GTGAAAGATTTAACCGCACTATTGTGGAGAGAGCCAGATGCTTACTTTATGATGCCAAGTTTGAGAAGAAGTTTTGGGCA

GAAGCAGTTCACACAGCTGTGTACTTAAAAAATAGAACAGTTGCTTCTGGGTTGAATCAAAAAACTCCCTATGAACTTTG

GACTGGCTGCAAGCCAAATGTCAGTCATCTAAGGTTGTTTGGAAGCACAGTTATGGCTCACATCCCTAAACAAAAAAGGC

TGAAATGGGATAAAAAGGCAGAAAAGCATTACTTAGTTGGGTATGCTGATAATATAAAGGGGTACAGGCTGTACAATCCC

AGTACCAAAAAGGTGATAACATGCAGGGATGTCACTGTCATGGAGCAGGATAATTCAGAAATAGTGCAAGCAACAATAAT

TGAAAGCAATGTGTCCTCATCAGACAGTTTTGGTCAGAATAAAGATCCTGATGGCTCAGACTCTGAAGTGTCAACTGGTA

CAGTGAAAAGTAACAATAATGACAGTACTTACATAGAAGAATCTCACTCACACAGTAGTTCAGATGAATTTTTTGACAGT

ATACCAACAAAGGAGTTAAAGGATTTAATTGAGTGTGATAAACCAGATAAAGCTGTTAGAAAAAGGAAAAAACCTGACAG

ATATGGTTATAGTAGTATGTGTGTTGAAACAGGATTAAATTTATGTGCAGACCAGCTGATAACATATGAAGAGGCTGTAA

ATGGACCAGAATCAGAGGAATGGTGTAAAGCTATGCAGGAGGAACTAAAATCATTCGAAGATAATGAAGCTTGGGATGTG

GTGAACAGGCCAGAGCAAGCTACAGTGGTTGAATGTAAGTGGGTGTTTAAGAAAAAAGTAGATTGCGATAATAATGTGAG

ATATCGTGCTCGTTTAGTCGCCAAAGGATTCTCCCAAAAGGCAGGAATAGACTATACAGAAACATTTTCCCCAGTTGTCC

GTCATTCAACATTAAGATTGTTGTTTGCATTAAGTGTCAAATATAACTTAGATATTACTCATTTCGATGTAACCACAGCA

TTTTTAAATGGATATTTAAAAGAAAATGTATACATGAGCTTACCTCAGAACCTTGAATGTAGTAATAAAGAAAACAAAGT

GTTAAAGTTGAAACGTGCTATCTATGGGCTAAAACAGTCTGCTAGGGCATGGTATAAACGCGTAGAAGATTGTCTACATG

ATTTAGATTATAAAAAATCAGAATATGAACCCTGTTTATTCATGAAACTTAATAATAATGCTAGGATATATGTGGCTTTG

TTTGTAGATGACTTCTTTGTTTTTTCTGATAGTCAAAAGGAAACTGAATCTCTTAAAAAACAATTACAAAGTAAATTTAA

GTTGAAAGACTTGGGAAAGATAAGACAATGTTTAGGTATGAGGGTTAGAAGGGAAAACAATGAAATTTTTGTAGACCAGG

AACAATTTGTTGATCATTTGTTGAATAAGTTTAATATGAGAAATTGTAATCCAGCCAGTACACCGATGGAAGTAAATTTG

AAGCTTGGAAAGGGTGATAATAATTGTTCTAAACAATACCCCTACCAGCAGTTAATAGGAAGTTTGCTCTATTTGTCCAT

GTTAACCCGACCTGATATATTTTTTGCTGTTTGTTACCTTAGCCAGTTTAATAACAATCATAGTGAAACTCATTGGAAGC

ATGTAAAGCGTATTTTAAAGTATTTGCAAAAAACTAAACATTTTGGTTTGAAATATGTAAAAGATGATTTAGATCTGATA

GGTTATGCCAATGCAGATTGGGCATCAGATTGTGTTGACCGCAAATCCTATACAGGTTTCATGTTTAAAATGTCTGGTAG

TGTAGTTTCATTTGAATGCAAAAAGCAGTCAACAATAGCGCTGTCAAGCACAGAAGCTGAGTATATGGCCATATGTGAAG

CAAGTAAGGAAGCCATATACCTTAAGAACTTGTTATTTGAGTTGAAATGTAGAAATGATTTGCCTGTATTGTTATATAAT

GACAACCAGGGTGCGCAATTGTTAACTAAACAATCTGTATTCCATAAAAGAAGCAAACATATAGATATTAGATTTCATTT

TGTCAGAACAGCAGTTGAAAATAATTGTATTAAAATAGAGTACTTAAATACCAATTCTATGCCTGCAGATATATTTACAA

AAAGTTTGAGTTGTCAAAAGCATAATAATTTTGTTGGACAGTTAGGTATAACTTCCATGTAAATTTGTTTCATTTATGTT

TATTTTTTGTATTTTTTCAATATCCGTGTTCAAATTATTTTGATATGGGAGCATGTTAAATTTGTATCAAAATAATATTA

CTTGTATACTATAGTGTCATTAGTACTGAAATGAAACGTTAAATATATTTTTAATCTGTGCCGAGCACTCTGTAGTTTTA

TTAGGTTATGGGCCCAGACCCGCTTAAAATAGTAATTTGTTGTGGAAATACTGTGAAAAATGGCTGGCAGTTATATTATT

AATGTTCCGAAGCTCCGAGGTCGAGATAATTACGATGAGTGGGCATTCGCGGCCGAGAATTTCTTGATTTTGGAAGGCGT

CGACATCATCAAGCAAGAAGCCGGCAATGGCGGCAATGCAGTCACCGTCGACGAACAAAAGGCAAAAGCAAAATTAGTGA

TGACAATAGATTCGTCGTTATTCGTACATATCAAGAATGAAAAAACTGCTTTGGACATATGGAAAAAACTCAAGACGCTA

TTCGACGATTCTGGTTTCACCCGAAGAATTAGCTTGTTACGAAATTTGATTTCCATACGCCTCGAGAGCAGCGAATCAAT

GACGGCATACATTACGCAGATCATAGATACGGCGCAAAAATTAAGAGGAACGGGTTTCGACATTAATGAAGAATGGATAG

GATCGCTATTGTTGGCAGGATTGCCGGAAAAATTTTCGCCCATGATTATGGCGATCGAACATTCTGGGTTAGCCATATCT

GCTGACGTCATAAAAACAAAATTGTTGGACATGAGCGATAATGTTGGCAGCAGTGAGTCCGAAAGCGCATTTTTAGCGTC

TAAAGGTTGGCAGCGCCGTAAAGGAAATAAAGTTGTCAAAACTGTTGATACGTCAAACCGACCTGTCAAAGTGATTAAAT

GCTATAAATGTAAACAAACCGGTCACTTTAAAAACCAATGTCCGCAAACAAAGGAAAAACAAGTAAATGCGTTCAGTGCA

GTATTTCTTAATGGCAGTTTTGATAAGCATGAATGGTACATTGACTCTGGTGCCAGTAGTCACATGATGATCAGCCAAGA

CAACATAAAAAATGTGTCATGTAACCTCACTACAAAGGAGATTATAGTTGCAAACAGATCAACTATGCCTGTATTGTGCT

CTGGAGATACACAAATTACGACTGTTGTGAACAACAAGGAATTTGATATTGTTGTCAAAGATGTGTTGTGTATTCCCACA

CTCACCACCAACTTATTGTCTATCAGTCAGCTGATAAAGCATGGTAACAAAGTCAGTTTTCATCAGGAATGTTGTTACAT

CAGAAATCAGCAGAATGAACTGATTGGCATAGCACAGTTGGTAAATGGGGTCTACAAGTTAAACACCAGGTCAGTATGTT

TGTTCACGGCTTCAGCAACAACAACCTCAAACGTAATTTGGCACAGAAGACTTGGACATATAAATAGCAAAGACATGAAT

GACATGAGAGAGGGTGCTGTTGATGGCTTAGAATTTGACAAAAAAGCTGAGATCAGCAAGTTCAATTGCACAGTATGCTG

CGAAGCCAAACAAACCAGACTGCCATTTCCACATAGCAGTAATAGAAGTATGCATGTCCTCGATTTGATTCATGCAGATG

TGTGTGGGCCGATGGAAACCAAATCACTCGGCTCATCCAGATATTTCTTGCTATTTGTGGATGATTATAGTAGAATGTCA

TTTGTCTATTTTTTGCAGAATAAAAGTGGAGTCCACTCCTGTTTTAAAGAATTCAAGATTATGGTTGAAAATCAGATGAA

TTGCAGTATAAAAGTTCTAAGGACAGACAATGGCACAGAATTTTGCTCAAATGAAATGAAGAACTATTTAAAACAACATG

GCATAATACATCAGCGCACCAACCCATATACCCCAGAACAAAATGGGATGTGTGAAAGATTTAACCGCACTATTGTGGAG

AGAGCCAGATGCTTACTTTATGATGCCAAGTTTGAGAAGAAGTTTTGGGCAGAAGCAGTTCACACAGCTGTGTACTTAAA

AAATAGAACAGTTGCTTCTGGGTTGAATCAAAAAACTCCCTATGAACTTTGGACTGGCTGCAAGCCAAATGTCAGTCATC

TAAGGTTGTTTGGAAGCACAGTTATGGCTCACATCCCTAAACAAAAAAGGCTGAAATGGGATAAAAAGGCAGAAAAGCAT

TACTTAGTTGGGTATGCTGATAATATAAAGGGGTACAGGCTGTACAATCCCAGTACCAAAAAGGTGATAACATGCAGGGA

TGTCACTGTCATGGAGCAGGATAATTCAGAAATAGTGCAAGCAACAATAATTGAAAGCAATGTGTCCTCATCAGACAGTT

TTGGTCAGAATAAAGATCCTGATGGCTCAGACTCTGAAGTGTCAACTGGTACAGTGAAAAGTAACAATAATGACAGTACT

TACATAGAAGAATCTCACTCACACAGTAGTTCAGATGAATTTTTTGACAGTATACCAACAAAGGAGTTAAAGGATTTAAT

TGAGTGTGATAAACCAGATAAAGCTGTTAGAAAAAGGAAAAAACCTGACAGATATGGTTATAGTAGTATGTGTGTTGAAA

CAGGATTAAATTTATGTGCAGACCAGCTGATAACATATGAAGAGGCTGTAAATGGACCAGAATCAGAGGAATGGTGTAAA

GCTATGCAGGAGGAACTAAAATCATTCGAAGATAATGAAGCTTGGGATGTGGTGAACAGGCCAGAGCAAGCTACAGTGGT

TGAATGTAAGTGGGTGTTTAAGAAAAAAGTAGATTGCGATAATAATGTGAGATATCGTGCTCGTTTAGTCGCCAAAGGAT

TCTCCCAAAAGGCAGGAATAGACTATACAGAAACATTTTCCCCAGTTGTCCGTCATTCAACATTAAGATTGTTGTTTGCA

TTAAGTGTCAAATATAACTTAGATATTACTCATTTCGATGTAACCACAGCATTTTTAAATGGATATTTAAAAGAAAATGT

ATACATGAGCTTACCTCAGAACCTTGAATGTAGTAATAAAGAAAACAAAGTGTTAAAGTTGAAACGTGCTATCTATGGGC

TAAAACAGTCTGCTAGGGCATGGTATAAACGCGTAGAAGATTGTCTACATGATTTAGATTATAAAAAATCAGAATATGAA

CCCTGTTTATTCATGAAACTTAATAATAATGCTAGGATATATGTGGCTTTGTTTGTAGATGACTTCTTTGTTTTTTCTGA

TAGTCAAAAGGAAACTGAATCTCTTAAAAAACAATTACAAAGTAAATTTAAGTTGAAAGACTTGGGAAAGATAAGACAAT

GTTTAGGTATGAGGGTTAGAAGGGAAAACAATGAAATTTTTGTAGACCAGGAACAATTTGTTGATCATTTGTTGAATAAG

TTTAATATGAGAAATTGTAATCCAGCCAGTACACCGATGGAAGTAAATTTGAAGCTTGGAAAGGGTGATAATAATTGTTC

TAAACAATACCCCTACCAGCAGTTAATAGGAAGTTTGCTCTATTTGTCCATGTTAACCCGACCTGATATATTTTTTGCTG

TTTGTTACCTTAGCCAGTTTAATAACAATCATAGTGAAACTCATTGGAAGCATGTAAAGCGTATTTTAAAGTATTTGCAA

AAAACTAAACATTTTGGTTTGAAATATGTAAAAGATGATTTAGATCTGATAGGTTATGCCAATGCAGATTGGGCATCAGA

TTGTGTTGACCGCAAATCCTATACAGGTTTCATGTTTAAAATGTCTGGTAGTGTAGTTTCATTTGAATGCAAAAAGCAGT

CAACAATAGCGCTGTCAAGCACAGAAGCTGAGTATATGGCCATATGTGAAGCAAGTAAGGAAGCCATATACCTTAAGAAC

TTGTTATTTGAGTTGAAATGTAGAAATGATTTGCCTGTATTGTTATATAATGACAACCAGGGTGCGCAATTGTTAACTAA

ACAATCTGTATTCCATAAAAGAAGCAAACATATAGATATTAGATTTCATTTTGTCAGAACAGCAGTTGAAAATAATTGTA

TTAAAATAGAGTACTTAAATACCAATTCTATGCCTGCAGATATATTTACAAAAAGTTTGAGTTGTCAAAAGCATAATAAT

TTTGTTGGACAGTTAGGTATAACTTCCATGTAAATTTGTTTCATTTATGTTTATTTTTTGTATTTTTTCAATATCCGTGT

TCAAATTATTTTGATATGGGAGCATGTTAAATTTGTATCAAAATAATATTACTTGTATACTATAGTGTCATTAGTACTGA

AATGAAACGTTAAATATATTTTTAATCTGTGCCGAGCA

>TRINITY_DN9626_c0_g2_i1_Copia-1_BM#LTR/Copia159-4034[Bombyxmori]

TACGATGAGTGGGCATTCGCGGCCGAGAATTTCTTGATTTTGGAAGGCGTCGACATCATCAAGCAAGAAGCCGGCAATGG

CGGCAATGCAGTCACCGTCGACGAACAAAAGGCAAAAGCAAAATTAGTGATGACAATAGATTCGTCGTTATTCGTACATA

TCAAGAATGAAAAAACTGCTTTGGACATATGGAAAAAACTCAAGACGCTATTCGACGATTCTGGTTTCACCCGAAGAATT

AGCTTGTTACGAAATTTGATTTCCATACGCCTCGAGAGCAGCGAATCAATGACGGCATACATTACGCAGATCATAGATAC

GGCGCAAAAATTAAGAGGAACGGGTTTCGACATTAATGAAGAATGGATAGGATCGCTATTGTTGGCAGGATTGCCGGAAA

AATTTTCGCCCATGATTATGGCGATCGAACATTCTGGGTTAGCCATATCTGCTGACGTCATAAAAACAAAATTGTTGGAC

ATGAGCGATAATGTTGGCAGCAGTGAGTCCGAAAGCGCATTTTTAGCGTCTAAAGGTTGGCAGCGCCGTAAAGGAAATAA

AGTTGTCAAAACTGTTGATACGTCAAACCGACCTGTCAAAGTGATTAAATGCTATAAATGTAAACAAACCGGTCACTTTA

AAAACCAATGTCCGCAAACAAAGGAAAAACAAGTAAATGCGTTCAGTGCAGTATTTCTTAATGGCAGTTTTGATAAGCAT

GAATGGTACATTGACTCTGGTGCCAGTAGTCACATGATGATCAGCCAAGACAACATAAAAAATGTGTCATGTAACCTCAC

TACAAAGGAGATTATAGTTGCAAACAGATCAACTATGCCTGTATTGTGCTCTGGAGATACACAAATTACGACTGTTGTGA

ACAACAAGGAATTTGATATTGTTGTCAAAGATGTGTTGTGTATTCCCACACTCACCACCAACTTATTGTCTATCAGTCAG

CTGATAAAGCATGGTAACAAAGTCAGTTTTCATCAGGAATGTTGTTACATCAGAAATCAGCAGAATGAACTGATTGGCAT

AGCACAGTTGGTAAATGGGGTCTACAAGTTAAACACCAGGTCAGTATGTTTGTTCACGGCTTCAGCAACAACAACCTCAA

ACGTAATTTGGCACAGAAGACTTGGACATATAAATAGCAAAGACATGAATGACATGAGAGAGGGTGCTGTTGATGGCTTA

GAATTTGACAAAAAAGCTGAGATCAGCAAGTTCAATTGCACAGTATGCTGCGAAGCCAAACAAACCAGACTGCCATTTCC

ACATAGCAGTAATAGAAGTATGCATGTCCTCGATTTGATTCATGCAGATGTGTGTGGGCCGATGGAAACCAAATCACTCG

GCTCATCCAGATATTTCTTGCTATTTGTGGATGATTATAGTAGAATGTCATTTGTCTATTTTTTGCAGAATAAAAGTGGA

GTCCACTCCTGTTTTAAAGAATTCAAGATTATGGTTGAAAATCAGATGAATTGCAGTATAAAAGTTCTAAGGACAGACAA

TGGCACAGAATTTTGCTCAAATGAAATGAAGAACTATTTAAAACAACATGGCATAATACATCAGCGCACCAACCCATATA

CCCCAGAACAAAATGGGATGTGTGAAAGATTTAACCGCACTATTGTGGAGAGAGCCAGATGCTTACTTTATGATGCCAAG

TTTGAGAAGAAGTTTTGGGCAGAAGCAGTTCACACAGCTGTGTACTTAAAAAATAGAACAGTTGCTTCTGGGTTGAATCA

AAAAACTCCCTATGAACTTTGGACTGGCTGCAAGCCAAATGTCAGTCATCTAAGGTTGTTTGGAAGCACAGTTATGGCTC

ACATCCCTAAACAAAAAAGGCTGAAATGGGATAAAAAGGCAGAAAAGCATTACTTAGTTGGGTATGCTGATAATATAAAG

GGGTACAGGCTGTACAATCCCAGTACCAAAAAGGTGATAACATGCAGGGATGTCACTGTCATGGAGCAGGATAATTCAGA

AATAGTGCAAGCAACAATAATTGAAAGCAATGTGTCCTCATCAGACAGTTTTGGTCAGAATAAAGATCCTGATGGCTCAG

ACTCTGAAGTGTCAACTGGTACAGTGAAAAGTAACAATAATGACAGTACTTACATAGAAGAATCTCACTCACACAGTAGT

TCAGATGAATTTTTTGACAGTATACCAACAAAGGAGTTAAAGGATTTAATTGAGTGTGATAAACCAGATAAAGCTGTTAG

AAAAAGGAAAAAACCTGACAGATATGGTTATAGTAGTATGTGTGTTGAAACAGGATTAAATTTATGTGCAGACCAGCTGA

TAACATATGAAGAGGCTGTAAATGGACCAGAATCAGAGGAATGGTGTAAAGCTATGCAGGAGGAACTAAAATCATTCGAA

GATAATGAAGCTTGGGATGTGGTGAACAGGCCAGAGCAAGCTACAGTGGTTGAATGTAAGTGGGTGTTTAAGAAAAAAGT

AGATTGCGATAATAATGTGAGATATCGTGCTCGTTTAGTCGCCAAAGGATTCTCCCAAAAGGCAGGAATAGACTATACAG

AAACATTTTCCCCAGTTGTCCGTCATTCAACATTAAGATTGTTGTTTGCATTAAGTGTCAAATATAACTTAGATATTACT

CATTTCGATGTAACCACAGCATTTTTAAATGGATATTTAAAAGAAAATGTATACATGAGCTTACCTCAGAACCTTGAATG

TAGTAATAAAGAAAACAAAGTGTTAAAGTTGAAACGTGCTATCTATGGGCTAAAACAGTCTGCTAGGGCATGGTATAAAC

GCGTAGAAGATTGTCTACATGATTTAGATTATAAAAAATCAGAATATGAACCCTGTTTATTCATGAAACTTAATAATAAT

GCTAGGATATATGTGGCTTTGTTTGTAGATGACTTCTTTGTTTTTTCTGATAGTCAAAAGGAAACTGAATCTCTTAAAAA

ACAATTACAAAGTAAATTTAAGTTGAAAGACTTGGGAAAGATAAGACAATGTTTAGGTATGAGGGTTAGAAGGGAAAACA

ATGAAATTTTTGTAGACCAGGAACAATTTGTTGATCATTTGTTGAATAAGTTTAATATGAGAAATTGTAATCCAGCCAGT

ACACCGATGGAAGTAAATTTGAAGCTTGGAAAGGGTGATAATAATTGTTCTAAACAATACCCCTACCAGCAGTTAATAGG

AAGTTTGCTCTATTTGTCCATGTTAACCCGACCTGATATATTTTTTGCTGTTTGTTACCTTAGCCAGTTTAATAACAATC

ATAGTGAAACTCATTGGAAGCATGTAAAGCGTATTTTAAAGTATTTGCAAAAAACTAAACATTTTGGTTTGAAATATGTA

AAAGATGATTTAGATCTGATAGGTTATGCCAATGCAGATTGGGCATCAGATTGTGTTGACCGCAAATCCTATACAGGTTT

CATGTTTAAAATGTCTGGTAGTGTAGTTTCATTTGAATGCAAAAAGCAGTCAACAATAGCGCTGTCAAGCACAGAAGCTG

AGTATATGGCCATATGTGAAGCAAGTAAGGAAGCCATATACCTTAAGAACTTGTTATTTGAGTTGAAATGTAGAAATGAT

TTGCCTGTATTGTTATATAATGACAACCAGGGTGCGCAATTGTTAACTAAACAATCTGTATTCCATAAAAGAAGCAAACA

TATAGATATTAGATTTCATTTTGTCAGAACAGCAGTTGAAAATAATTGTATTAAAATAGAGTACTTAAATACCAATTCTA

TGCCTGCAGATATATTTACAAAAAGTTTGAGTTGTCAAAAGCATAATAATTTTGTTGGACAGTTAGGTATAACTTCCATG

TAAATTTGTTTCATTTATGTTTATTTTTTGTATTTTTTCAATATCCGTGTTCAAATTATTTTGATATGGGAGCATGTTAA

ATTTGTATCAAAATAATATTACTTGTATACTATAGTGTCATTAGTACTGAAATGAAACGTTAAATATATTTTTAATCTGT

GCCGAGCAAAAAAAAAA

>TRINITY_DN9433_c0_g1_i1_Harbinger-1_DW_tp#DNA/PIF-Harbinger964-2779viagenewisewithHarb_Cis1_tp,1intron(1248:1902)[Drosophilawillistoni]

TCTATTTAATGCAAAGGCTTCGTCTCCGACCAGAAAGTGAGGGAGGATTACATTTGTTCCTAGTTAAGCTTTTGGTGGCG

GGATATTAAAATTATTGGTGTCTATTTGCCTCCCCATCAATGATTTTTGAAATATACCATTGTCAGATTCTTTTCCGTAA

GCACCAACATCAACTGCTACAAACTTATAATTAGCATCCACGATTGCTAATAGCACAATTGAAAAGTAGTTTTTGTAATT

AAAAAACAATGATCCACTCTTTTTAGGTGCCCTGATTCGCACATGTTTCCCGTCCATTCCTCCTACGCAATTTGGTATAT

TCCATTTTTCCCAAAATTCACGTTCTATGCGTTTGAAATCGTCTTGTAATGCAGGTTTCATTAGTATAGGCATTAATTTT

TGTCTCATAACTTTAAGAACACTCTTCACAATTTGGGATATGTAGCACTCTGATATGCGGTATGCAGTTGACAAAGATTT

ATAGGTTTCCCCTGTGGCCAAAAATCTTAATGTTAAAAATAATTTTCGCTCAGCATTTATGGCATTTATTTTCATTGGCT

CGATTTCTTCTCTGATTAAACTTAGTATAAAATCGAACTGTTTTTCATTTAAACGACAATACTCTCTGAATTCTTTCATA

TCGCTTTTCAAATGGTTTTTGATCAGTATTGAATAAGTTCCCTCACTTGCTCTGCGTTTAATCATAGGACATGTGGCTTG

TCGTCTATTTCTGTATAATAAAAGCAAGTCATCATCATCATCTTCCTCTTCTAATAAAAGACGTAAAAAATTGCGATCAC

TATTCTCAAACACGTCAGACATCTTATTTCACGGACCGCTCGGAATCAACTGGATGCCATCTGCAGTTGTCGTCTGCAGT

CGGGATCGAAATCATGACTGCAGCGACAGGACGCAACGACGTGTCGCGACGACATGAAGCGACGACATGTCGTCTGCAGT

TGTCGCCTGCGGTCGGCTGCCAACTTGTACCTTAATCCTTGATATCAACGCTGTCTATTTGAGAACTGTTTTCAAATAAA

ACGAATAAACTTACATTCAATTAAGATTTATTAAATGTTGTTTTCTTGCAAAAGTATTTTATTTTTAAAAAGTATTTTAA

AGATAGGTATTTTGTATTTATATATTTCAGATACCTTTTTAGAAAAGTATTTATATCTGGTATCTGAAATGCTTTTTTTA

AAGTATCTTGCATCTAGTATTTTAGATACTTTGTATAAGGTATCTTTTACATCAATTTTGTACTTTATATAATTACTAAT

TATATAAAGTACAAAATTTACGTTTGTTTTTTTTTTTCTGAATTTACCTACGTACACTATAAATTACAAGCAAAAACCAG

TGTCTATATGTTAAGTTACCGTTCAAACATAAGAATTAAAAAAACCCTTATAGTAAGTAACATACTAAGAATTGATGAGC

GCAATTGTACACATGCGCCGACTACCCCCACTATTTTCGTCGAGCGATCAAGAACCGCCGCGCGACCTATACCATCATAT

TTTGGCTTATTAGGAATGTGATTGGTGCAAGTAACAGTTATTGTTGGGGGCGGCTGACCTATGGCCACAAGGTAGCGGTT

CGGCTACGGTCTGAATGACGAATAGTTCATCTAAATCTATACCTCAGATGTTAAAGTAATCAATAAAAAAATTTAAGATA

TCAAGAAAAATATTCAATTAGGTAGTGAAGTCAAGTCGAACTAGAATAAACAAGCTTATAGAACTGAATATTCCAAAGAT

TTACAATCAACAACCTTAATAGATTACATGACCAAAAACCAAGACAAACAACAATCAAAGAAACAACAAAAACAGGCGAC

AAACTTTTTAAATGTTTGAGACACACTTAATTGTATCTCAATAACTGACATAGATGGCGACTGTTGTACCACAGTAGTCA

ATTTTGAATCCCAACAAAACAATATCTAATAAAGACTGAATTTTTGACTCATGCTTAACTTAGCTAAGTTAGATTGCAGA

CTGGTTTTTCCTTTATTGTGAAAGCACATTTTTCCACAAACCAAGTGACTAGACTTGATTTTCTCAACTGTAGCTTGTTG

TCACCAATAAGTATTCAGGTTCAACAAAGGTTAAAACCAGCGTTCACATCTTATTTATACACTATCAGCAGCACAGAACA

ATAATACTCTCTAACGAGATCTAACGAGAGGGTCAGCAGGCTGCAACAGTGCAAGTATTATGTATTTTAAGTACAACTCA

GCACAAGGTTCCAAAGATTT

>TRINITY_DN5858_c0_g1_i1_CR1-5_NVi_pol#LINE/L21509-4384viatfastywithCR1-73_HM_pol,1frameshift.[Nasoniavitripennis]

TGGGGATGGTATGTATGAAAGTAAGTGAGTAAATGTATGTGGGGTGTGTGAATGTGTGTGTGTGAGTGTGTGAGCGTGTC

TGTAAGTGTAATGCGGACGTGATAATCTTGGTCAAGAGAAAGGTGCTTTATTGTTGTAATTGTGTAGCTAAACAAATCGA

AAAGCAACAGTACGGTAAGTATGTCCACCTACTGAGGACGGTGAATCAATTCCTCTGTTTGGGCGTAGTTCAGTTCTTGT

AGCCAATCTTGTACTTTAATCTTCACCTCATGATAATTAAGGTCATTTATTTGGAGAATTTTGCAAATTCGTTTATATAT

AAAAGAACTGATAGTGTAAAACTGGCGTCCAGCGAATGCGGTTCTGTGGCTTATTACTGGACACATAACTTTCCCTTTGC

GTCCGCTTGATGTTGGTGGGGGAATTTGTCTATGTTTTCTGAGAATGGTTCGAAGCACAAAGAGCTGTCTCACTGTGAGT

AATTTACAGTCAACATATAACTGTGAGGTGGGAAATCTGTACGGTCTACCAAGCATTACTTTAAGCACAGCTCTTTGTGC

TCGTTCCAACTCAATAAACTTAGTCTTGGCGGCCCCTCCCCACACCGGGATGCAATAAGTGATAACGGACTGACATAGTG

ATAGATAAACCATTTTTAGAATTTTAGTATCAGCACAATATCTAAGAGTTTTAAAAATATAAATAAGTTTCCTAGCTTTC

GAGATAGTATTGTCTATTTGATTGTACCATTTCAGTCCGTCATCAATCACTACACCCAGGTACTTTGTGTGGGTGACACG

GGTAAGAGACACGCAAGAACACGAACTGGGATCGGGACATGTGTGTGCCGTAATCTTAAATGCGGTTGGTGGCTTTGTTC

TGGAGCTCAAGGAGAATGGGATATAATTAGTTTTAGTCAAGTTAAGGGTTAATAGGTTAAGTTGCAACCATTTCACTACT

GTTTTTAAAGATTGTTCCGCATTGGTCTTCACTTCGCTCCAAGTGTTACCGTAGACCACGATCGCAGTATCGTCAGCGTA

AGTGAAGATTCTCGATTTATGTAGGGGCAGCGTGCAAAGGTCATTTATATAAATCTGAAACAATGTAGGTCCAATCACGC

TGCCTTGCGGAACTCCGTATGTAACAGTTTCCTCATCACTTATGTGTGTATTGATCTTCACAGCTAGTTTTCTATTCTGA

AGATAACTTTTAAAAAGTTCCAGTGGTTTGCCACGAATTCCTATAGACTCCAATTTAAGGAGAAGGGTGGGAACAGAGAC

TGTGTCAAACGCTTTCGTTAAATCTAGGAAGATACAAGCGCATTTTTGTTGTTCGTCGAGTTTGGTAGCAACAAACTGAG

TTAGGTCAATAACTGCGTCTTCACAAGAAACTCCTTTTTTGAATCCATATTGGTTTGTTGCTATAATATTGAATTTGTTA

AGGTAATTTACTAATTGGCTATTTATTAACTTTTCCATGACTTTAGATATTGCCGAGAGGACTGAAATGGGTCTATAATT

GTTGACACAGTCTCTGGTCCCGCTCTTGTGTATGGGATAAACTAAAGCCTTCTTAAAAGGTTCTGGGAATATTCCTTTTG

CTATGGAGATGTTGATAATATGTGTAATTATTGGTACAAGAGCATTCCGACAAATTTTCAACAGGGTGGATGATATTCCA

TCCCAGCCAGTGGCACTATCCTGTCTTAAGTTCATGATAGTGGATTCCACCTCGGAGTCATCTACATCAAGAATTACAAA

GGAGTTGGTGAAAGTATTTGAGATGGTAAACGGGTGTGAGGTAGTAGGGATATTAGAAGCTAGATCAGCCCCAATCGTGG

CAAAGAAGTGACATATGCTGTTAATTGCTAAATGAGGGTTAGATTTGGTATTCAATAGTTCGGTAGGCGGTGAACTTATC

TTTTTGGTATTGGTGATGTCTTTTATTTTATTCCATAATGTTTTAGGGTTGTGTTTGGCTTTTTCCAACTCAGATTTCTC

ATATAATGTTTTTAGTTTCCTTAGAAGGCTATTGCAGTAATTACGGTATCGTTTGTAAGTAATTTGGACAGTTTGGTTAT

CTGGTGATTTTCGAGCTTTTTTGTGAAGTCTATCGCGGTGTCGAATACACCTTAGTAGACCGGGTGTAATCCATGGTTTT

ATGTTTCGCTCTTTTTTAGGGACATTAATCATTTTAGTGTGTGTGTTAAGAACCGTTTTTATTGTGTTTATCAAGGTGAT

AGAGGCCAGGTTAGGGTCGTCTGTATTTATGATTCGAGAAAAATCTAGAGATTCTATGTCTGTTTTAACATTAGCGTAGT

CTATTTTAGGAATTTTGAATAGCGGGACTGTCTGTTTTAGAGGTTTTGTGTCCTCTATTTCTACAAGCAACGGGAGATGA

TCAGTAATGTGGGATTCTAGAACCAGTACAGTTGTGGTTTTGTTCGTTTTTAAGATAACGTGGTCAATACAGGAATTTAT

TCTGGTTGGTAAATAATGTGCTGGTAGTAAGCCATGGGTAGCGTTAAGGGTTAAATAATTGCTTGAGCGTTCATCTACTC

TTATATTAGAAGGTTTTATATCAATATTGACATCACCTATAAGACATATATTTGTGTATTTACTAAATGAGGTTAATATG

TTATCCAAACTACTTAGAAAATTTTCAAATCCTTCTTTTGTTTTGTATGACGGAGATCGATAAAGTGAAATTATTGCTAA

TTTATTATTTATTTCACAAGTGAGACAATTCCCATTTTTAAATTCTGGTTCATTAATAGTGTACTTTATCTGATTTTTTA

TGTAAATAACTATGCCGTCATTTTGATTACTTGTTTGTTTCGAATAATAGGAACTGTACCCATCTAGTATGGGTAAGTTT

ACTATTTTACTCAGCCAACATTCCGTTAGTATAAGGACGTCACAACTGACTTTGATCAATGGTAATAATGTTAATAATTC

GTCAAAATTTCTTTTAATGCTGCGAATATTTATATGAAGTATAGTAATAGATCCGTTCAAACCGGTAACATATTTATTAC

AGTCTTCTGGAATACATAAATAAGCCTTCGCGATTTCAATTTCATCTAAGTCATTGCTTATATTTTCAACTAGATTAGCC

ATTTATAAAATAAATAAAACCCCAATA

>TRINITY_DN7853_c0_g1_i1_Crack-6_CQ_pol#LINE/L23-1592Fragment[Culexquinquefasciatus]

GTACCGGGCTTGACGGAATAAGCGCAAAAGCTATAAAATGTTTAAAAAATATAATACTAAACAGATTAACCAACTGCATA

AACAAATGTTTACAAAATGGCACTTTTCCCGACACACTCAAGGTAGCCAAGGTCAGTCCTATCTATAAGGCCGGCAGCAG

ATCAGATCCAGGCAATTACAGGCCCGTCTCTGTACTACCCATACTTTCCAAAATATTTGAGCGTGTGATATACACTCGGC

TAAATAACTACCTAAATGAGAAAAAGTTTTTAATTGACGAACAATATGGTTTCCGATCCAAATCCAGTACACTATCAGCT

GCGATAGATTTAATAACCAAAATTAAAATTAATATTGACCAAAAAAAATTTGCTGTAGGTATATTTATCGATTTAAAAAA

ATGTTTTGACACCGTTAGTCCTCAAAAATTACTCGATAAATTAGAGAATATAGGCGTGAGGGGTACAGCGCTAAAAATTT

TTAATTCATACATGCAAAATAGGAGACAGGTTGTCAAAATGGGTAATTTTGTTAGTTCGCCACAGAACTTGGATTACGGC

TGTCCTCAAGGCTCGATCATAAGTCCATTACTATTCCTAATTTATGTGAATAATATTAATAAAATAGGCCTCAGTGGCCA

TTTAACACTTTACGCGGATGATACCTGTTTATTTTATTTTGACAAGTCAATCAAAGAAATCATGACTGATGCACAGAAAG

ACCTGAATTTGCTTGCTGAGTGGCTAAAATATAACTTGTTAACAGTGAACGCCTCAAAAACATCCTATATGATTTTTACT

GCAAAAAATAAACAGATACCCACATTCACACCGCTAACTATTAATGACGAACCAATAAAACGCTCAAACGCGGAAAAATA

TTTGGGCCTGTGGTTAGATGACAAACTAACGTGGAACCTCCATATCAATCACATCCGCACAAAGTTAACATCACTACTAG

GAAGCCTTCGCAGAATAGCACATTGCATACCATATCAAGTGCGCAATGTTATCTATAACTCGTTAGTAAAATCAAACCTT

GAGTACATGATTGAAATATGGGGATCAGCCGCCGCCACTCATTTGAAACAATTACAAACTACCCAAAATAAAATTATTAA

GGTGCTTCACCACTATAACTACTTGACTCCAACAAAAACACTATACGAAAAGACTTACGTACTAAATCTTACGCAACTCT

ACACATATTGTACTTGTATCCTAGTTAGAAATATAAGTAACCTAACAATACAAACAAACATTAAACTACACAAAAAAGAA

ACTACATATAATCTAAGACATAATAAAAACAAAATTCAACTAAGGAAGCCACGCACTAACTTCGGAAAAAAAACAATAGT

ATTTGAGGGAGCACAACTGTATAACAACTTACCAAAAGAAGTGAAAGAAAGCAGCAGTATAAATGTATTTAAAATGAAAC

TAAAAAGATATCTCAGCACACTGTAAGCGACTCGTTATCAATAATTTTTGGTTAAGAATATATATATATAAAATAATAGC

AAATAATAT

>TRINITY_DN14441_c0_g1_i1_Harb_Cis1_tp#DNA/PIF-Harbinger283-1581[Cionasavignyi]

GGAGAGACTAGATTTATAACACATAAAAACTATAGTATTATCTTTAACAATGTCTTTGTTAAGCCATGTATTCACTGAGC

AACAGTGTTTCGGATACACAGTTTCTGAAACATCACGTAAATGTTTCCAGGAACAATGTTTCGGAAACTCTGCCGTCCAC

ACAGTCAGTTGCTTAGGTCATGTCGCCCGAGGAAGTAGTGTTGTTATGTGGTGCCTACATTTTTTTGCATAAGTCCATTT

CTAGTAAAAAGAAACGTAAAAGATGGTGGGTCAGGAATTACTTGCTAAGAAGACAAGACGTATTAAGTGATTTATGCATG

TTTGATGGATCATTTATAAACTTTCTACGAATGTCAAAATCAGATTTTGAATATCTTTTGCAAAATGTTGGACCATTTAT

AGAGAAACAAGATACAAACTTACGAAGTGCAGTTACCGCTGAAACAAGACTAGCCATTACTTTAAGATATTTAGCTACTG

GAGATTCATATTCTTCACTCTCATACACTTTTAGAGTTTCCAAACAATTAATCAGCCGGATCATACCCGAAGTTTGTAAG

AATATAAAACGTGTGTTGAAGAACTACATCCAGGTAAGAAAAACTAAGTTATGAATCAATATTTTTTTTATTAATTATAC

AAAATTAGGCGAACTATCTACTGGGTACTACTATCGGTTTCTTCTACTGATGCGATTTGTGAAACTATCTCTTCAATGGT

TTCAAGTTCTTTTTGTGAGGTTGAACTTGCTGGTGTCGACGCAGTTTCATATCCGGGATGGCTATGATATCCGTAACCAT

AATTGTATTTGCCCATTCGCATGTCTATTAAAATATTATTAATATAAGCCTTGGCTTGTATTACGGCAGTCTCGTTTTGT

ACCGCTCTTAATTCTGAAGCGACGTATTGCCCATAAATATCATATTCATCCTTAGATTCATCCATTTTGCGTTTTGCAGA

TTTGACTATTTCAAATACTTCACTTAGATTGTCATCCACTTTTCTTTTAGATGCTTTTTTGCTACTTGTAGATGGTAGTG

GGGATGGTGGAAGTGGAGATTGTGGTTTCTGCTGCTCCGATTGCTGTTCTGGCTCCTCAGTTGCCAGGAGGTCTTCACGG

TTTGCCTGCAATTTTAAACACTTTTTTTTTAAGGTTCCGAGTTCCGAAGCGCACTGGAAAGAAAAGGCCCGTGAGTGGAA

CGAATTATGGAATTTTCCACACTGTGTCGGAGCTATAGATGGGAAGCATGTGGTTATTGAAGCGCCTAGCAATAGTAACA

GTGATTACTACAACTACAAAGATCAGTTAAGTATTGTACTACTGGCTATTGTAGATGCATCTTATAATGTTATATATGCA

AACTGTGGTGCGAAAGGAAGAGCATCTGACAGTGGCATCTTTCAAGAAACGAGTTTCTATCAAAGAATGGTGGAACACCG

TCTTAATTTTCCAAACCAAGAAACGATATCACCAGATGGACCTGACCTTCCTTATGTAATACTGGGCGATAGTGCATTCC

CATTATCGGAAAACTTGATGAGGCCGTACCCGGGTATTCATAACCGTGGCACAATGAAACGAATTTTCAACTATAGACTC

TGTAGGGCCAGGAGGGTTGTAGAAAATGTTTTTGGAATTTTATGCGTGGTTTTTCGTGTATTTCGTAAACCAATTCCTCT

CAAGCCTGAAAAGTGTGAACTTATTGTAATGGCATGTCTTTACATGCATAACTTTTTAAGAAGGAATAAACAATCTAGAG

CCTTGTATACGCCCCCGATGACTTTTGATTTTGAAGACAGTGACCATAATGTCATAGAAGGTGCTTGGCGTAGAGAGTAT

ACAGTTGAAGGAACTTCAATTTTGGAATTGGAAAGGCGACCTAGAAATTCAGCACGTACTGCTACAATCATAAGGGATCA

ATTTGGTGAATATTTCATGAGCGAAAGAGGAAGCTTGCCGTAACAAAACAATGTAGCTTAATGCTATGGCGGTAGTGTTT

TCGTATATGTTAGGTAGAATATTTACATGCCTACAGCTGATAACATAAAAATAAAATATGAATAATGCGCTCACTGTTTC

ATTTAAAGTATCCGCACCACCGACCCTTGTCGTCGTGCATTGTAAGAAAATTAATTCTTCATAAAATTTCCATTTTGATT

TCTTAATATCTGCAGCACCAGCACCTGTGGTTTTTGTAGACAACATTTTCTTCCTCTCTCATGCAAATTGCGACCGCAAT

ATATGCATTTTATTCTCAACATCTGTTTTTGGAATTGCCAAAGCATTCGAAATTTCTTCCCAGGCGTCATTCTTTTTTAT

TTTATTTTTATAGTCCTTTGTCGAGCAATCCCATAATATTCGTTTTTTCTCAAACAGTTCTATCAATTTAAAGACAGTTT

CATCGTCCCACACTAAAGCCATGTTTATAAACAAATGTTTAGACGCGTCCACACTCGCATGCACCTGTTGCGCAACTGAT

TTGCTTCTGTGTTCTCCGTCACAAGTGGGGAGCACCGAAACATACCGATAAACACGTAGTGTTTACAGAAACAAAATTAT

ATGTTTCTGAAACAAAATGATTTGTTTCAGAAACACGTAGATTTTGTTTCTGAAACAGAGTTTATAAACAATTGTTGCTC

AGTGAATACGTGGCTTAACTAAACAATCCACTTACGGTAGATTACCTACCTTAAATATTAAATACATTATAACGTCAATT

GTTTACAATCAAACAGCAGTGCTACATATATTTTCATGTTAAAATATTTTGTTTTACCCAAAAGATAAATTTAATACCAA

TGTATGGTTTAAAAAATAATTAATGGGATATATCATTCCTATATATTTTGGTATGAAGTACCGATCTAAATATTTTAGAT

ACTTAGAAGTGGTTGTTTTGTTACAGTATTAAAATAAATATAATTTAAGGAAAAAAAAAAA

>TRINITY_DN14441_c0_g1_i2_Harb_Cis1_tp#DNA/PIF-Harbinger283-1581[Cionasavignyi]

GGAGAGACTAGATTTATAACACATAAAAACTATAGTATTATCTTTAACAATGTCTTTGTTAAGCCATGTATTCACTGAGC

AACAGTGTTTCGGATACACAGTTTCTGAAACATCACGTAAATGTTTCCAGGAACAATGTTTCGGAAACTCTGCCGTCCAC

ACAGTCAGTTGCTTAGGTCATGTCGCCCGAGGAAGTAGTGTTGTTATGTGGTGCCTACATTTTTTTGCATAAGTCCATTT

CTAGTAAAAAGAAACGTAAAAGATGGTGGGTCAGGAATTACTTGCTAAGAAGACAAGACGTATTAAGTGATTTATGCATG

TTTGATGGATCATTTATAAACTTTCTACGAATGTCAAAATCAGATTTTGAATATCTTTTGCAAAATGTTGGACCATTTAT

AGAGAAACAAGATACAAACTTACGAAGTGCAGTTACCGCTGAAACAAGACTAGCCATTACTTTAAGATATTTAGCTACTG

GAGATTCATATTCTTCACTCTCATACACTTTTAGAGTTTCCAAACAATTAATCAGCCGGATCATACCCGAAGTTTGTAAG

AATATAAAACGTGTGTTGAAGAACTACATCCAGGTAAGAAAAACTAAGTTATGAATCAATATTTTTTTTATTAATTATAC

AAAATTAGGCGAACTATCTACTGGGTACTACTATCGGTTTCTTCTACTGATGCGATTTGTGAAACTATCTCTTCAATGGT

TTCAAGTTCTTTTTGTGAGGTTGAACTTGCTGGTGTCGACGCAGTTTCATATCCGGGATGGCTATGATATCCGTAACCAT

AATTGTATTTGCCCATTCGCATGTCTATTAAAATATTATTAATATAAGCCTTGGCTTGTATTACGGCAGTCTCGTTTTGT

ACCGCTCTTAATTCTGAAGCGACGTATTGCCCATAAATATCATATTCATCCTTAGATTCATCCATTTTGCGTTTTGCAGA

TTTGACTATTTCAAATACTTCACTTAGATTGTCATCCACTTTTCTTTTAGATGCTTTTTTGCTACTTGTAGATGGTAGTG

GGGATGGTGGAAGTGGAGATTGTGGTTTCTGCTGCTCCGATTGCTGTTCTGGCTCCTCAGTTGCCAGGAGGTCTTCACGG

TTTGCCTGCAATTTTAAACACTTTTTTTTTAAGGTTCCGAGTTCCGAAGCGCACTGGAAAGAAAAGGCCCGTGAGTGGAA

CGAATTATGGAATTTTCCACACTGTGTCGGAGCTATAGATGGGAAGCATGTGGTTATTGAAGCGCCTAGCAATAGTAACA

GTGATTACTACAACTACAAAGATCAGTTAAGTATTGTACTACTGGCTATTGTAGATGCATCTTATAATGTTATATATGCA

AACTGTGGTGCGAAAGGAAGAGCATCTGACAGTGGCATCTTTCAAGAAACGAGTTTCTATCAAAGAATGGTGGAACACCG

TCTTAATTTTCCAAACCAAGAAACGATATCACCAGATGGACCTGACCTTCCTTATGTAATACTGGGCGATAGTGCATTCC

CATTATCGGAAAACTTGATGAGGCCGTACCCGGGTATTCATAACCGTGGCACAATGAAACGAATTTTCAACTATAGACTC

TGTAGGGCCAGGAGGGTTGTAGAAAATGTTTTTGGAATTTTATGCGTGGTTTTTCGTGTATTTCGTAAACCAATTCCTCT

CAAGCCTGAAAAGTGTGAACTTATTGTAATGGCATGTCTTTACATGCATAACTTTTTAAGAAGGAATAAACAATCTAGAG

CCTTGTATACGCCCCCGATGACTTTTGATTTTGAAGACAGTGACCATAATGTCATAGAAGGTGCTTGGCGTAGAGAGTAT

ACAGTTGAAGGAACTTCAATTTTGGAATTGGAAAGGCGACCTAGAAATTCAGCACGTACTGCTACAATCATAAGGGATCA

ATTTGGTGAATATTTCATGAGCGAAAGAGGAAGCTTGCCGTAACAAAACAATGTAGCTTAATGCTATGGCGGTAGTGTTT

TCGTATATGTTAGGTAGAATATTTACATGCCTACAGCTGATAACATAAAAATAAAATATGAATAATGCGCTCACTGTTTC

ATTTAAAGTATCCGCACCACCGACCCTTGTCGTCGTGCATTGTAAGAAAATTAATTCTTCATAAAATTTCCATTTTGATT

TCTTAATATCTGCAGCACCAGCACCTGTGGTTTTTGTAGACAACATTTTCTTCCTCTCTCATGCAAATTGCGACCGCAAT

ATATGCATTTTATTCTCAACATCTGTTTTTGGAATTGCCAAAGCATTCGAAATTTCTTCCCAGGCGTCATTCTTTTTTAT

TTTATTTTTATAGTCCTTTGTCGAGCAATCCCATAATATTCGTTTTTTCTCAAACAGTTCTATCAATTTAAAGACAGTTT

CATCGTCCCACACTAAAGCCATGTTTATAAACAAATGTTTAGACGCGTCCACACTCGCATGCACCTGTTGCGCAACTGAT

TTGCTTCTGTGTTCTCCGTCACAAGTGGGGAGCACCGAAACATACCGATAAACACGTAGTGTTTACAGAAACAAAATTAT

ATGTTTCTGAAACAAAATGATTTGTTTCAGAAACACGTAGATTTTGTTTCTGAAACAGAGTTTATAAACAATTGTTGCTC

AGTGAATACGTGGCTTAAGGCGCAACGCTTCTAAACAAATTGCTGG

>TRINITY_DN14441_c0_g1_i3_Harb_Cis1_tp#DNA/PIF-Harbinger283-1581[Cionasavignyi]

TATAAGTGAAATCTGAAGTTTAAAGCCATGTATTCACTGAGCAACAGTGTTTCGGATACACAGTTTCTGAAACATCACGT

AAATGTTTCCAGGAACAATGTTTCGGAAACTCTGCCGTCCACACAGTCAGTTGCTTAGGTCATGTCGCCCGAGGAAGTAG

TGTTGTTATGTGGTGCCTACATTTTTTTGCATAAGTCCATTTCTAGTAAAAAGAAACGTAAAAGATGGTGGGTCAGGAAT

TACTTGCTAAGAAGACAAGACGTATTAAGTGATTTATGCATGTTTGATGGATCATTTATAAACTTTCTACGAATGTCAAA

ATCAGATTTTGAATATCTTTTGCAAAATGTTGGACCATTTATAGAGAAACAAGATACAAACTTACGAAGTGCAGTTACCG

CTGAAACAAGACTAGCCATTACTTTAAGATATTTAGCTACTGGAGATTCATATTCTTCACTCTCATACACTTTTAGAGTT

TCCAAACAATTAATCAGCCGGATCATACCCGAAGTTTGTAAGAATATAAAACGTGTGTTGAAGAACTACATCCAGGTAAG

AAAAACTAAGTTATGAATCAATATTTTTTTTATTAATTATACAAAATTAGGCGAACTATCTACTGGGTACTACTATCGGT

TTCTTCTACTGATGCGATTTGTGAAACTATCTCTTCAATGGTTTCAAGTTCTTTTTGTGAGGTTGAACTTGCTGGTGTCG

ACGCAGTTTCATATCCGGGATGGCTATGATATCCGTAACCATAATTGTATTTGCCCATTCGCATGTCTATTAAAATATTA

TTAATATAAGCCTTGGCTTGTATTACGGCAGTCTCGTTTTGTACCGCTCTTAATTCTGAAGCGACGTATTGCCCATAAAT

ATCATATTCATCCTTAGATTCATCCATTTTGCGTTTTGCAGATTTGACTATTTCAAATACTTCACTTAGATTGTCATCCA

CTTTTCTTTTAGATGCTTTTTTGCTACTTGTAGATGGTAGTGGGGATGGTGGAAGTGGAGATTGTGGTTTCTGCTGCTCC

GATTGCTGTTCTGGCTCCTCAGTTGCCAGGAGGTCTTCACGGTTTGCCTGCAATTTTAAACACTTTTTTTTTAAGGTTCC

GAGTTCCGAAGCGCACTGGAAAGAAAAGGCCCGTGAGTGGAACGAATTATGGAATTTTCCACACTGTGTCGGAGCTATAG

ATGGGAAGCATGTGGTTATTGAAGCGCCTAGCAATAGTAACAGTGATTACTACAACTACAAAGATCAGTTAAGTATTGTA

CTACTGGCTATTGTAGATGCATCTTATAATGTTATATATGCAAACTGTGGTGCGAAAGGAAGAGCATCTGACAGTGGCAT

CTTTCAAGAAACGAGTTTCTATCAAAGAATGGTGGAACACCGTCTTAATTTTCCAAACCAAGAAACGATATCACCAGATG

GACCTGACCTTCCTTATGTAATACTGGGCGATAGTGCATTCCCATTATCGGAAAACTTGATGAGGCCGTACCCGGGTATT

CATAACCGTGGCACAATGAAACGAATTTTCAACTATAGACTCTGTAGGGCCAGGAGGGTTGTAGAAAATGTTTTTGGAAT

TTTATGCGTGGTTTTTCGTGTATTTCGTAAACCAATTCCTCTCAAGCCTGAAAAGTGTGAACTTATTGTAATGGCATGTC

TTTACATGCATAACTTTTTAAGAAGGAATAAACAATCTAGAGCCTTGTATACGCCCCCGATGACTTTTGATTTTGAAGAC

AGTGACCATAATGTCATAGAAGGTGCTTGGCGTAGAGAGTATACAGTTGAAGGAACTTCAATTTTGGAATTGGAAAGGCG

ACCTAGAAATTCAGCACGTACTGCTACAATCATAAGGGATCAATTTGGTGAATATTTCATGAGCGAAAGAGGAAGCTTGC

CGTAACAAAACAATGTAGCTTAATGCTATGGCGGTAGTGTTTTCGTATATGTTAGGTAGAATATTTACATGCCTACAGCT

GATAACATAAAAATAAAATATGAATAATGCGCTCACTGTTTCATTTAAAGTATCCGCACCACCGACCCTTGTCGTCGTGC

ATTGTAAGAAAATTAATTCTTCATAAAATTTCCATTTTGATTTCTTAATATCTGCAGCACCAGCACCTGTGGTTTTTGTA

GACAACATTTTCTTCCTCTCTCATGCAAATTGCGACCGCAATATATGCATTTTATTCTCAACATCTGTTTTTGGAATTGC

CAAAGCATTCGAAATTTCTTCCCAGGCGTCATTCTTTTTTATTTTATTTTTATAGTCCTTTGTCGAGCAATCCCATAATA

TTCGTTTTTTCTCAAACAGTTCTATCAATTTAAAGACAGTTTCATCGTCCCACACTAAAGCCATGTTTATAAACAAATGT

TTAGACGCGTCCACACTCGCATGCACCTGTTGCGCAACTGATTTGCTTCTGTGTTCTCCGTCACAAGTGGGGAGCACCGA

AACATACCGATAAACACGTAGTGTTTACAGAAACAAAATTATATGTTTCTGAAACAAAATGATTTGTTTCAGAAACACGT

AGATTTTGTTTCTGAAACAGAGTTTATAAACAATTGTTGCTCAGTGAATACGTGGCTTAACTAAACAATCCACTTACGGT

AGATTACCTACCTTAAATATTAAATACATTATAACGTCAATTGTTTACAATCAAACAGCAGTGCTACATATATTTTCATG

TTAAAATATTTTGTTTTACCCAAAAGATAAATTTAATACCAATGTATGGTTTAAAAAATAATTAATGGGATATATCATTC

CTATATATTTTGGTATGAAGTACCGATCTAAATATTTTAGATACTTAGAAGTGGTTGTTTTGTTACAGTATTAAAATAAA

TATAATTTAAGGAAAAAAAAAAA

>TRINITY_DN14441_c0_g1_i4_Harb_Cis1_tp#DNA/PIF-Harbinger283-1581[Cionasavignyi]

TATAAGTGAAATCTGAAGTTTAAAGCCATGTATTCACTGAGCAACAGTGTTTCGGATACACAGTTTCTGAAACATCACGT

AAATGTTTCCAGGAACAATGTTTCGGAAACTCTGCCGTCCACACAGTCAGTTGCTTAGGTCATGTCGCCCGAGGAAGTAG

TGTTGTTATGTGGTGCCTACATTTTTTTGCATAAGTCCATTTCTAGTAAAAAGAAACGTAAAAGATGGTGGGTCAGGAAT

TACTTGCTAAGAAGACAAGACGTATTAAGTGATTTATGCATGTTTGATGGATCATTTATAAACTTTCTACGAATGTCAAA

ATCAGATTTTGAATATCTTTTGCAAAATGTTGGACCATTTATAGAGAAACAAGATACAAACTTACGAAGTGCAGTTACCG

CTGAAACAAGACTAGCCATTACTTTAAGATATTTAGCTACTGGAGATTCATATTCTTCACTCTCATACACTTTTAGAGTT

TCCAAACAATTAATCAGCCGGATCATACCCGAAGTTTGTAAGAATATAAAACGTGTGTTGAAGAACTACATCCAGGTTCC

GAGTTCCGAAGCGCACTGGAAAGAAAAGGCCCGTGAGTGGAACGAATTATGGAATTTTCCACACTGTGTCGGAGCTATAG

ATGGGAAGCATGTGGTTATTGAAGCGCCTAGCAATAGTAACAGTGATTACTACAACTACAAAGATCAGTTAAGTATTGTA

CTACTGGCTATTGTAGATGCATCTTATAATGTTATATATGCAAACTGTGGTGCGAAAGGAAGAGCATCTGACAGTGGCAT

CTTTCAAGAAACGAGTTTCTATCAAAGAATGGTGGAACACCGTCTTAATTTTCCAAACCAAGAAACGATATCACCAGATG

GACCTGACCTTCCTTATGTAATACTGGGCGATAGTGCATTCCCATTATCGGAAAACTTGATGAGGCCGTACCCGGGTATT

CATAACCGTGGCACAATGAAACGAATTTTCAACTATAGACTCTGTAGGGCCAGGAGGGTTGTAGAAAATGTTTTTGGAAT

TTTATGCGTGGTTTTTCGTGTATTTCGTAAACCAATTCCTCTCAAGCCTGAAAAGTGTGAACTTATTGTAATGGCATGTC

TTTACATGCATAACTTTTTAAGAAGGAATAAACAATCTAGAGCCTTGTATACGCCCCCGATGACTTTTGATTTTGAAGAC

AGTGACCATAATGTCATAGAAGGTGCTTGGCGTAGAGAGTATACAGTTGAAGGAACTTCAATTTTGGAATTGGAAAGGCG

ACCTAGAAATTCAGCACGTACTGCTACAATCATAAGGGATCAATTTGGTGAATATTTCATGAGCGAAAGAGGAAGCTTGC

CGTAACAAAACAATGTAGCTTAATGCTATGGCGGTAGTGTTTTCGTATATGTTAGGTAGAATATTTACATGCCTACAGCT

GATAACATAAAAATAAAATATGAATAATGCGCTCACTGTTTCATTTAAAGTATCCGCACCACCGACCCTTGTCGTCGTGC

ATTGTAAGAAAATTAATTCTTCATAAAATTTCCATTTTGATTTCTTAATATCTGCAGCACCAGCACCTGTGGTTTTTGTA

GACAACATTTTCTTCCTCTCTCATGCAAATTGCGACCGCAATATATGCATTTTATTCTCAACATCTGTTTTTGGAATTGC

CAAAGCATTCGAAATTTCTTCCCAGGCGTCATTCTTTTTTATTTTATTTTTATAGTCCTTTGTCGAGCAATCCCATAATA

TTCGTTTTTTCTCAAA

>TRINITY_DN14441_c0_g1_i5_Harb_Cis1_tp#DNA/PIF-Harbinger283-1581[Cionasavignyi]

TATAAGTGAAATCTGAAGTTTAAAGCCATGTATTCACTGAGCAACAGTGTTTCGGATACACAGTTTCTGAAACATCACGT

AAATGTTTCCAGGAACAATGTTTCGGAAACTCTGCCGTCCACACAGTCAGTTGCTTAGGTCATGTCGCCCGAGGAAGTAG

TGTTGTTATGTGGTGCCTACATTTTTTTGCATAAGTCCATTTCTAGTAAAAAGAAACGTAAAAGATGGTGGGTCAGGAAT

TACTTGCTAAGAAGACAAGACGTATTAAGTGATTTATGCATGTTTGATGGATCATTTATAAACTTTCTACGAATGTCAAA

ATCAGATTTTGAATATCTTTTGCAAAATGTTGGACCATTTATAGAGAAACAAGATACAAACTTACGAAGTGCAGTTACCG

CTGAAACAAGACTAGCCATTACTTTAAGATATTTAGCTACTGGAGATTCATATTCTTCACTCTCATACACTTTTAGAGTT

TCCAAACAATTAATCAGCCGGATCATACCCGAAGTTTGTAAGAATATAAAACGTGTGTTGAAGAACTACATCCAGGTAAG

AAAAACTAAGTTATGAATCAATATTTTTTTTATTAATTATACAAAATTAGGCGAACTATCTACTGGGTACTACTATCGGT

TTCTTCTACTGATGCGATTTGTGAAACTATCTCTTCAATGGTTTCAAGTTCTTTTTGTGAGGTTGAACTTGCTGGTGTCG

ACGCAGTTTCATATCCGGGATGGCTATGATATCCGTAACCATAATTGTATTTGCCCATTCGCATGTCTATTAAAATATTA

TTAATATAAGCCTTGGCTTGTATTACGGCAGTCTCGTTTTGTACCGCTCTTAATTCTGAAGCGACGTATTGCCCATAAAT

ATCATATTCATCCTTAGATTCATCCATTTTGCGTTTTGCAGATTTGACTATTTCAAATACTTCACTTAGATTGTCATCCA

CTTTTCTTTTAGATGCTTTTTTGCTACTTGTAGATGGTAGTGGGGATGGTGGAAGTGGAGATTGTGGTTTCTGCTGCTCC

GATTGCTGTTCTGGCTCCTCAGTTGCCAGGAGGTCTTCACGGTTTGCCTGCAATTTTAAACACTTTTTTTTTAAGGTTCC

GAGTTCCGAAGCGCACTGGAAAGAAAAGGCCCGTGAGTGGAACGAATTATGGAATTTTCCACACTGTGTCGGAGCTATAG

ATGGGAAGCATGTGGTTATTGAAGCGCCTAGCAATAGTAACAGTGATTACTACAACTACAAAGATCAGTTAAGTATTGTA

CTACTGGCTATTGTAGATGCATCTTATAATGTTATATATGCAAACTGTGGTGCGAAAGGAAGAGCATCTGACAGTGGCAT

CTTTCAAGAAACGAGTTTCTATCAAAGAATGGTGGAACACCGTCTTAATTTTCCAAACCAAGAAACGATATCACCAGATG

GACCTGACCTTCCTTATGTAATACTGGGCGATAGTGCATTCCCATTATCGGAAAACTTGATGAGGCCGTACCCGGGTATT

CATAACCGTGGCACAATGAAACGAATTTTCAACTATAGACTCTGTAGGGCCAGGAGGGTTGTAGAAAATGTTTTTGGAAT

TTTATGCGTGGTTTTTCGTGTATTTCGTAAACCAATTCCTCTCAAGCCTGAAAAGTGTGAACTTATTGTAATGGCATGTC

TTTACATGCATAACTTTTTAAGAAGGAATAAACAATCTAGAGCCTTGTATACGCCCCCGATGACTTTTGATTTTGAAGAC

AGTGACCATAATGTCATAGAAGGTGCTTGGCGTAGAGAGTATACAGTTGAAGGAACTTCAATTTTGGAATTGGAAAGGCG

ACCTAGAAATTCAGCACGTACTGCTACAATCATAAGGGATCAATTTGGTGAATATTTCATGAGCGAAAGAGGAAGCTTGC

CGTGGCAAAACAATGTAGCTTAATGCTATGGCGGTAGTGTTTTCGTATATGTTAGGTAGAATATTTACATGCCTACAGCT

GATAACATAAAAATAAAATATGAATAATGCGCTCACTGTTTCATTTAAAGTATCCGCACCACCGACCCTTGTCGTCGTGC

ATTGTAAGAAAATTAATTCTTCATAAAATTTCCATTTTGATTTCTTAATATCTGCAGCACCAGCACCTGTGGTTTTTGTA

GACAACATTTTCTTCCTCTCTCATGCAAATTGCGACCGCAATATATGCATTTTATTCTCAACATCTGTTTTTGGAATTGC

CAAAGCATTCGAAATTTCTTCCCAGGCGTCATTCTTTTTTATTTTATTTTTATAGTCCTTTGTCGAGCAATCCCATAATA

TTCGTTTTTTCTCAAACAGTTCTATCAATTTAAAGACAGTTTCATCGTCCCACACTAAAGCCATGTTTATAAACAAATGT

TTAGACGCGTCCACACTCGCATGCACCTGTTGCGCAACTGATTTGCTTCTGTGTTCTCCGTCACAAGTGGGGAGCACCGA

AACATACCGATAAACACGTAGTGTTTACAGAAACAAAATTATATGTTTCTGAAACAAAATGATTTGTTTCAGAAACACGT

AGATTTTGTTTCTGAAACAGAGTTTATAAACAATTGTTGCTCAGTGAATACGTGGCTTAAGGCGCAACGCTTCTAAACAA

ATTGCTGG

>TRINITY_DN14441_c0_g1_i6_Harb_Cis1_tp#DNA/PIF-Harbinger283-1581[Cionasavignyi]

CCAAACCGATCCGCGAAGTAGGCGGAGGGGATCGGCGTCAACGCTGAGCACAGGATCATAAAGCCATGTATTCACTGAGC

AACAGTGTTTCGGATACACAGTTTCTGAAACATCACGTAAATGTTTCCAGGAACAATGTTTCGGAAACTCTGCCGTCCAC

ACAGTCAGTTGCTTAGGTCATGTCGCCCGAGGAAGTAGTGTTGTTATGTGGTGCCTACATTTTTTTGCATAAGTCCATTT

CTAGTAAAAAGAAACGTAAAAGATGGTGGGTCAGGAATTACTTGCTAAGAAGACAAGACGTATTAAGTGATTTATGCATG

TTTGATGGATCATTTATAAACTTTCTACGAATGTCAAAATCAGATTTTGAATATCTTTTGCAAAATGTTGGACCATTTAT

AGAGAAACAAGATACAAACTTACGAAGTGCAGTTACCGCTGAAACAAGACTAGCCATTACTTTAAGATATTTAGCTACTG

GAGATTCATATTCTTCACTCTCATACACTTTTAGAGTTTCCAAACAATTAATCAGCCGGATCATACCCGAAGTTTGTAAG

AATATAAAACGTGTGTTGAAGAACTACATCCAGGTAAGAAAAACTAAGTTATGAATCAATATTTTTTTTATTAATTATAC

AAAATTAGGCGAACTATCTACTGGGTACTACTATCGGTTTCTTCTACTGATGCGATTTGTGAAACTATCTCTTCAATGGT

TTCAAGTTCTTTTTGTGAGGTTGAACTTGCTGGTGTCGACGCAGTTTCATATCCGGGATGGCTATGATATCCGTAACCAT

AATTGTATTTGCCCATTCGCATGTCTATTAAAATATTATTAATATAAGCCTTGGCTTGTATTACGGCAGTCTCGTTTTGT

ACCGCTCTTAATTCTGAAGCGACGTATTGCCCATAAATATCATATTCATCCTTAGATTCATCCATTTTGCGTTTTGCAGA

TTTGACTATTTCAAATACTTCACTTAGATTGTCATCCACTTTTCTTTTAGATGCTTTTTTGCTACTTGTAGATGGTAGTG

GGGATGGTGGAAGTGGAGATTGTGGTTTCTGCTGCTCCGATTGCTGTTCTGGCTCCTCAGTTGCCAGGAGGTCTTCACGG

TTTGCCTGCAATTTTAAACACTTTTTTTTTAAGGTTCCGAGTTCCGAAGCGCACTGGAAAGAAAAGGCCCGTGAGTGGAA

CGAATTATGGAATTTTCCACACTGTGTCGGAGCTATAGATGGGAAGCATGTGGTTATTGAAGCGCCTAGCAATAGTAACA

GTGATTACTACAACTACAAAGATCAGTTAAGTATTGTACTACTGGCTATTGTAGATGCATCTTATAATGTTATATATGCA

AACTGTGGTGCGAAAGGAAGAGCATCTGACAGTGGCATCTTTCAAGAAACGAGTTTCTATCAAAGAATGGTGGAACACCG

TCTTAATTTTCCAAACCAAGAAACGATATCACCAGATGGACCTGACCTTCCTTATGTAATACTGGGCGATAGTGCATTCC

CATTATCGGAAAACTTGATGAGGCCGTACCCGGGTATTCATAACCGTGGCACAATGAAACGAATTTTCAACTATAGACTC

TGTAGGGCCAGGAGGGTTGTAGAAAATGTTTTTGGAATTTTATGCGTGGTTTTTCGTGTATTTCGTAAACCAATTCCTCT

CAAGCCTGAAAAGTGTGAACTTATTGTAATGGCATGTCTTTACATGCATAACTTTTTAAGAAGGAATAAACAATCTAGAG

CCTTGTATACGCCCCCGATGACTTTTGATTTTGAAGACAGTGACCATAATGTCATAGAAGGTGCTTGGCGTAGAGAGTAT

ACAGTTGAAGGAACTTCAATTTTGGAATTGGAAAGGCGACCTAGAAATTCAGCACGTACTGCTACAATCATAAGGGATCA

ATTTGGTGAATATTTCATGAGCGAAAGAGGAAGCTTGCCGTAACAAAACAATGTAGCTTAATGCTATGGCGGTAGTGTTT

TCGTATATGTTAGGTAGAATATTTACATGCCTACAGCTGATAACATAAAAATAAAATATGAATAATGCGCTCACTGTTTC

ATTTAAAGTATCCGCACCACCGACCCTTGTCGTCGTGCATTGTAAGAAAATTAATTCTTCATAAAATTTCCATTTTGATT

TCTTAATATCTGCAGCACCAGCACCTGTGGTTTTTGTAGACAACATTTTCTTCCTCTCTCATGCAAATTGCGACCGCAAT

ATATGCATTTTATTCTCAACATCTGTTTTTGGAATTGCCAAAGCATTCGAAATTTCTTCCCAGGCGTCATTCTTTTTTAT

TTTATTTTTATAGTCCTTTGTCGAGCAATCCCATAATATTCGTTTTTTCTCAAACAGTTCTATCAATTTAAAGACAGTTT

CATCGTCCCACACTAAAGCCATGTTTATAAACAAATGTTTAGACGCGTCCACACTCGCATGCACCTGTTGCGCAACTGAT

TTGCTTCTGTGTTCTCCGTCACAAGTGGGGAGCACCGAAACATACCGATAAACACGTAGTGTTTACAGAAACAAAATTAT

ATGTTTCTGAAACAAAATGATTTGTTTCAGAAACACGTAGATTTTGTTTCTGAAACAGAGTTTATAAACAATTGTTGCTC

AGTGAATACGTGGCTTAAGGCGCAACGCTTCTAAACAAATTGCTGG

>TRINITY_DN14441_c0_g1_i7_Harb_Cis1_tp#DNA/PIF-Harbinger283-1581[Cionasavignyi]

GGAGAGACTAGATTTATAACACATAAAAACTATAGTATTATCTTTAACAATGTCTTTGTTAAGCCATGTATTCACTGAGC

AACAGTGTTTCGGATACACAGTTTCTGAAACATCACGTAAATGTTTCCAGGAACAATGTTTCGGAAACTCTGCCGTCCAC

ACAGTCAGTTGCTTAGGTCATGTCGCCCGAGGAAGTAGTGTTGTTATGTGGTGCCTACATTTTTTTGCATAAGTCCATTT

CTAGTAAAAAGAAACGTAAAAGATGGTGGGTCAGGAATTACTTGCTAAGAAGACAAGACGTATTAAGTGATTTATGCATG

TTTGATGGATCATTTATAAACTTTCTACGAATGTCAAAATCAGATTTTGAATATCTTTTGCAAAATGTTGGACCATTTAT

AGAGAAACAAGATACAAACTTACGAAGTGCAGTTACCGCTGAAACAAGACTAGCCATTACTTTAAGATATTTAGCTACTG

GAGATTCATATTCTTCACTCTCATACACTTTTAGAGTTTCCAAACAATTAATCAGCCGGATCATACCCGAAGTTTGTAAG

AATATAAAACGTGTGTTGAAGAACTACATCCAGGTTCCGAGTTCCGAAGCGCACTGGAAAGAAAAGGCCCGTGAGTGGAA

CGAATTATGGAATTTTCCACACTGTGTCGGAGCTATAGATGGGAAGCATGTGGTTATTGAAGCGCCTAGCAATAGTAACA

GTGATTACTACAACTACAAAGATCAGTTAAGTATTGTACTACTGGCTATTGTAGATGCATCTTATAATGTTATATATGCA

AACTGTGGTGCGAAAGGAAGAGCATCTGACAGTGGCATCTTTCAAGAAACGAGTTTCTATCAAAGAATGGTGGAACACCG

TCTTAATTTTCCAAACCAAGAAACGATATCACCAGATGGACCTGACCTTCCTTATGTAATACTGGGCGATAGTGCATTCC

CATTATCGGAAAACTTGATGAGGCCGTACCCGGGTATTCATAACCGTGGCACAATGAAACGAATTTTCAACTATAGACTC

TGTAGGGCCAGGAGGGTTGTAGAAAATGTTTTTGGAATTTTATGCGTGGTTTTTCGTGTATTTCGTAAACCAATTCCTCT

CAAGCCTGAAAAGTGTGAACTTATTGTAATGGCATGTCTTTACATGCATAACTTTTTAAGAAGGAATAAACAATCTAGAG

CCTTGTATACGCCCCCGATGACTTTTGATTTTGAAGACAGTGACCATAATGTCATAGAAGGTGCTTGGCGTAGAGAGTAT

ACAGTTGAAGGAACTTCAATTTTGGAATTGGAAAGGCGACCTAGAAATTCAGCACGTACTGCTACAATCATAAGGGATCA

ATTTGGTGAATATTTCATGAGCGAAAGAGGAAGCTTGCCGTAACAAAACAATGTAGCTTAATGCTATGGCGGTAGTGTTT

TCGTATATGTTAGGTAGAATATTTACATGCCTACAGCTGATAACATAAAAATAAAATATGAATAATGCGCTCACTGTTTC

ATTTAAAGTATCCGCACCACCGACCCTTGTCGTCGTGCATTGTAAGAAAATTAATTCTTCATAAAATTTCCATTTTGATT

TCTTAATATCTGCAGCACCAGCACCTGTGGTTTTTGTAGACAACATTTTCTTCCTCTCTCATGCAAATTGCGACCGCAAT

ATATGCATTTTATTCTCAACATCTGTTTTTGGAATTGCCAAAGCATTCGAAATTTCTTCCCAGGCGTCATTCTTTTTTAT

TTTATTTTTATAGTCCTTTGTCGAGCAATCCCATAATATTCGTTTTTTCTCAAA

>TRINITY_DN14408_c0_g1_i2_L2-1_BM_pol#LINE/L2257-3202[Bombyxmori]

TAATTTATTCCAAGTTGAGAGGTTCTTAGTAAAATATTTGTTACATTTTTCTTTATTTATAGTTTATGAATAGAAATTTA

GAATGAATTTTAAGGTGTAAATTAAGATGCTCTGTTTAGGTGTTAGTGATCTTAAGGAATAAATTATACTTGTAAATTCG

CGATGGTTGAATGTTTTTATTGAGGAGAATACAAATAAGAACTCTTTCAATGGTATGTGCTTTTTTAATCAAGAAAAGAT

TGTGTTATTCTTGAATCTGAGACTAACAATAATTGTAGTAAGAGTCGTAAGGTCTAAATCGAGTAAGCGCTGCTCTATTT

ATTTTCCTTTTTTTTTGTAAGAAAAGAGCCTTTAATAAGTGGACCAATTTCTGGTCCTATTACATTAAAAAAAAAAACAG

AGTACTTTTTTTCATTTGCACTAAAACTTTATATAACTTACTATATTTAAATACGAAAAACAAAATTCCAATTGACACTA

CTGCAGGCGACCTCCAAAAAGTTGTAAAAAGTATGCAATCAAAAGAGTACATTTCAGTTCGGTCTTCCGTTGAATGATTT

CCATCAAATATCCAATTAATTAGTCCGCTCCAAAAAAACTTCATTTTCAGCTTGAAACAAATTTGTTTGTGTCTCTCGTA

CCGTTTTCCAGAAACTAAAAAAACGCGACTGGTTAGGGTCCGAGTTATGCTCGAAAGGTTCAGTACCCAACTTCCAATAT

TAATCATAATGGGCGACAATTTGTATATTGAAAAGTTGATGTTCCTGAGAAAAAAGACATTCCACCAAGACAACTGAAAT

TTGAACCTATAAACAACACAAGTCCAAATCAAGCAACAACTTCCTTAAAAAAATTTTGAAAAATTGTATTGCTGTTGTAG

ACATGCCACTGAATATGTCATCTCTTCTATCACTAATCTCTCTATGGGACAAACAAACGCGCTGTTGAAGAGTGCGCGAA

TCAGGGAGCTTTTGACATCCGAAAAGCACTCTGTGGTAACCATGTTTGTAGAAATGGTTTCTAAATTTAAATTTGACGAA

CCCTGAAAATAGGAGAAATAGGAAAGAAAATTACAGGCACAAAGCTTTTGAAAGCTCGATATGAAATGAATGCCGTTTGG

TGCGGTTCTAATGTCACGTTGCAGATGAAGATAGAAGGTTTTTTTTTCCTCTGTGTGTAGTCACAGTGAACAGACGTGTT

TTTGTGAGCTCCTCAATTTAAAAAAAAGTTTTGCTATTATATTTAAAATTATTAAATGAAAGACACTTAGCAAGTACACC

ACCCTCTTGCTAGTTAAAATAATTTATTTTCAACACAATGGACAACAAATTTTCAAGCAGTTTGGCAGATCTGGAGCAGC

TGTTCCATTCCCGTATGGCTGAATATGAAGATAAACTGGTCAAAGCTTCTGCAGGAGCAGGTCAACCATCAGACAGCATA

ACCTCCATCAGCAATGACTTCAGGGACTTCAAGACCTTTGTTTGGCAAGCACTGTCTAAGTTAAAATCCCAAGTTGAGTT

GCTTGGCCATGGTTTTGACAGGCATGAGACCCTCATGCGAAGAAAGGTGCTGCTGCTTCATGGGGTGCCTTGCATGTTTG

TCACCGCTTAGGCCCTTCCAGGCAGAAGACTCGACCCATCCTAGTGAGGTTTTTCAAGGTAGAGCATCGGGAGCTTGTTT

GGGACGGCAAGAAGCAGTTAAAGGGCTCGGGCATCACCATTTCTGAGTTTCTGACTCAGACTCGACATCAAGCCTTCCTT

GCCGCCAGGAAACATTTCAGCGTCACGAAGTGCTGGACCACTGAGGGCAGGATAGTGATCCTTTGCCCTGACAATATTCG

GCGGAAGATTGAGACGGTAAGTGAGCTCAACGAGTTGACAACCCGTTTTCCTACTGGCGCCATTGAGACCTCCGGCTCCG

ACCCTGGTCCTCTTAAAGACGCCCCAGAGACCTCCAAGGCTCAGCGAAAGGCGAGGCGTCGGAACTGAAGACATCATGCA

CTTTCACTAATTCACTTCTAACTTCAATCTCTTTTGCTTTCGTCGGTACTTCAATTTAAATTTGCGTTAACTATAATTCC

GTTACTTTTAAAATAGTTCTATAACTTCTTGAAGGTATAACTGTATTTTGTTGTGCACAGTTCTATATTTATTGTTGCCA

CTTTATTACTTTTAATATTTAATTTAAAGGTCAGTGCACCTATAATTGTGTAATACATACTTTGCAGAATCTGTGGTTAT

TTCCTAGTGACATCTGTCAAATTGTGTCTAACTGACATCTTCTTGCTGCGTTCATTAATACTTGCGCGTGAGTGTTTTAC

CTGATTTGGCTTCATGTCTTTTAATTTGTTTCATTTTATTTTTTTATTTTTTTTTGTTTCTGTTTGTCGTTCTTTGTGTT

GGAATGTACTTATTTCTTATTAATTCACCGGCGAGAAGGGGTGGTCGTTGCTATTGCTACGTGCTTATTAGTTATTAATT

TAGTGTAAAATATTATTATACATATATTAATTTATTTATTTTTAATTATTTAATGGATGCTCTCGACGAGCTTAATGATA

GTTTTTTTTCCTGCTCTTCCGAATCTTGCGGTTATGTTAGCGCGGACGACTTAAATTCTACTCCCTCTTTACACGAACAA

ATTTCTTCTCTTTTCTTTGAACATCGTAACAATTTTAACGTCGTGCATATTAATGCCCAGAGTGTTCCGAGTCACCATTC

CGACCTTCTAGCATCCTTTAGCAACACAAACGTCGATGCCATTTTGATTTCGGAAACCTTCCTCAAACCTTCACTTCTCT

CTACTCAATTTTCTCTTCCCGGCTTCGTTCTCATTCGTAATGATAGAACTGGGAAAGGTGGTGGGGGTGTTGCCATTTAC

TTGCGAGCCCATTTACCTCATAAAATTGTTCTGGCCTCACCCTCATTGTACTCCGAGTCATCCGAATACCTATTTCTTGA

AATTTCTTTAAATTCTTCCAAAATTCTCCTTGCTGTCCTCTATTCCCCCAACTTACGCATCGACTATTTCCGCTCTCTTG

ATTCCACTTTGAGTGAACTTTGCCCTATTTACAAACATATCATCATCATGGGCGACTTTAACACATGTCTTCTTAAAAAT

GATTCACGTTCCACCAGACTTACTTCTCTCCTTTCTTCTTATGATCTGCATGTCTTACCTCTCTCTGCTACTCACTTTGC

TCCCAACTGTTCTCCTTCTCTATTAGACCTTGCGATTGTGTCTTCCCTTGACAATGTCGCACGGCACGGTCAACTTACGG

CAACTTTCTCTTACCACGATCTAATATTTCTATCGTATCGTGTTCGCACTCCTAAGCCTAAGCCACAGTATTTGTATTTG

CGTAATTACAATGCGATAGACATTGAAGCTCTGCAGGCCGACGCTGCCGCACTTGACTGGTCACCTGTCTCTGATAGCCC

CGGTACGGAACAAAAAGTGTCCTGCTTTAATTCGATGGTCCTTGGTTTAATGGACAAGCATGCACCTATCCGCCGTATTA

AAATAAAGCATAAGCCAGCTCCGTGGATAACAGCTGAAATTAAAGTTCTTATGGTCCGCAGAGACAGAGCGAAGAATAAA

CTCAAGGGCAGGCCGTCTGATGTGAACCTCGAATACTATAGGGCTCTGCGCAATCGCTGCAGCCGTCTGTGTAGAGATGC

TAAAAGGCGCTACTTCCACGAATCACTTATCAACCGTAGTTCCTCCGAGGCTTGGAAATTTCTCAAATCCGTTGGTATCG

GCAAATCTCCCATACACACCTGTACAGACATGGATCTAAATGAATTAAATCTCTCTCTTTCTCAGCCGCCTATCCATTTA

GATCCAAATGCCAAAACTGTCACTCTCACTGAGCTTCAGAACTTGCCGGCACCAAACTGTCCCTCCTTTTCATTTAGTCC

TGTTTCTGAGGTTGACGTTGAAAAATCGCTTCTTGCAATTACCTCCAATGCAATTGGCTGTGATAATGTGTGCTCGAGAA

TGATCCGTCTCATCCTTCCCCACCTACTCCCGATCCTAACTCACATTTTCAATCATTCCTTATATACTTGTAATTTCCCA

TCCGCCTGGAAGCAGGCACATATCTTACCTTTACCCAAGATCCCTAACCCCTCTGTATCATCTCATTTCAGACCCATTTC

CATTCTCCCTTACCTCTCCAAAGTTCTCGAGCATATCGTTCACAGGCAGATAACCGGTTTTTTAACGTCAAATTCTCTTA

TTTCTTCTTTCCAATCTGGTTTCCGTTCTGGCCATAGTACCGTCACTGCCTTGTTGAAGGTGACTGACGACATCCGCTGG

GCAATGGAGAAAAAGATGATGACGGTCCTTGTCCTGCTTGACTTCAGCAGTGCCTTCAACTCGGTAGATTTTGATGTGCT

ACTTGGCATCCTAAAATCTCTAAATTTCTCTTCTACATCCCTAGCATGGTTAGATTCTTACCTTCGGGGTCGCTCGCAGT

GTGTTCGCCTTGAAGAGTCTCACTCTGATTGGTGCGAGCTCACGGCGGGGGTTCCCCAGGGGGGCGGCCTT

>TRINITY_DN14408_c0_g1_i3_L2-1_BM_pol#LINE/L2257-3202[Bombyxmori]

TTTTTTTTTTTTAATGTAATAAATCGTTTTATTGCATCCTATTACACAATATTATAACACTTTATTATGTAAAAATGGAA

TAACCGCTATTACAAAAACGATTTGCTCGTACATTATAAGTATGTAGCAAAAAACGAGCTATGTATATTCATTGTAATGC

TAAAGACTATGCGGACATACTGAATCATTTGTAATTGCACGATGCATTTCATATCTAACAACACATTCAGACCTGGAAAT

AATAGCAGTATCGTATACCTAAGGCCGAGAATGGCTGTTTTAACAAACATACTGCCAACCATTCGATCGAAGAAATATAA

CCATAATAATCAATTTTAAAACAATCTGTAAATAAAAACCATTTAATATCATTGTATATTAATCACATTTATTATAAAGT

AATATAAAAACATCTAAAATAATAAACAATTATTCAACATAAACATGTATAAAACGCATATCTGCCTATATTAACAAAGC

CTCTTTCAACCGCATGACTCGGAACTGCATATGATACTTACAGATAACGAATTGAATCTCGCAATAAGTGGATAAGTGTG

AAATATTGGACGCGCCGATGTTAATACTTCAATGTTTACGTTGAGTCTCATCTCGTACGAGTACACGAGGACGTTTGTGA

CGTGAGTCATAATTAGGGGCTCTGTTCGTGATGATCCCGTATAGATAAATTTCCACGTCCTGTTGGAGTTTGGTACTGTA

CTGGATGTACTGTGTTGAGGTCACGCTGGCGGTGACTGATGTAAACAATCATGAGAAGACGTAGAACATTGAGAGTGTTT

ATTTGACGTTCGCGTTTGTCGCCGTCTTGCCGACGTCCTCGTCGTCCGAGCAGTCCTCGTCGTGCGTGTGCGTGAGCTGG

CGCGGGGTCTCGGGACGGACGTGCGGGACTTGATGCTTAGTGTCGGGATTGTCAGTATGGCTGGGGGCGGCATCCGAGCT

GTCCTCGTTGAGGCCGAGCTGCAGGCTGAAACTGGGCTGACGCGGCGCGCTCGTTTGTCTAGATCTGTCGATAGTGGGCG

CGTCTACGGTGAACCTCTGTTCGTGTGCCACCGTAGACTCGTGGGGCATAAGCATCGCCGGGGCGCGGAACATGTAGCAG

AACGGGTAGTACATCAAGTTCAGGAACGAAGCCGCGTACAAGTCGGCGTACCTTACAACCTGTGACGAGAAGAAAGTCTG

CCTAGATCCGGAGCGGAACAGCGACCCCAGCATACCGTAGGCGAGGTCCATCTTGTGTGTGACGTCACGGATGGACATGC

GCAGCTTAGAGATATCGGGCTTCTCCTTTGTACTTGAATCTAAGTCTTTGTACATATCGCCTAGTTGCACATCAAGGTTC

TGTAGCTGAGCAAACAACTGACATTTGTCGGTCCACACGTGTAGCTCTTGAACTAATTCAGGTACGATGAGGAAGGTGCG

CCAGCCTCCGATCTTCTTGGACTTGAGAATATCACCGAATATGTGGTCACCGATGTATAGAACATCTTTACCCTTAGCAC

TGATCAATTCAGTGAATACGTCGCAGGAACCTCCAGAATACACAAGTCCTTCGTGCAGCGGGCCGACGTGGTGGCCCATC

TTGAGCGCGCCGGTCTGAGTGTCGACCTGCCTGAGTGTTGTTCCCTCGCCGAAGAACAACGGCTTCTTCGCGTCTACCAC

GATCCAGTCGAAGTAAGTCCTCCAGTTCCTGTGGGGCTCACCAGGCTTAGCGCCATATGGAAAATCGAATAGGTAGTTCA

TGATTTGGTCAGTGAAATTGTAGTCGCTGTTGGTCAATATGAAGATCTTGGCGCCGCTGTCCCGTATCCTCGAGAGGAAC

ATGGGCAGCCTGTCGTCTTTCTTCAAATACAAATCCATATTTTCTATAGTCTTGCTCTTTAAGTCCCCGTGTATGTGAAC

GTAGTCGACCGCATTTCTGACGTCTTGGAAGATTGATTTGAATGACATTGCAAGATCTCCGGCTCTTACGCCAGTTTTTT

CTCTTGTGTATTGAGGAGAATTTGTGAAGAAATCTATCAAACAAGCTATGAGGTATGTTTCTGGCAAGTTGAATAGTGTG

TTCAACACATACACTCTAGACTCATCCAATGTTAAGAACTTGTTTGGATATAATTCGTATACTTGTGAATGTTTTAAGAA

TTCGAATCCATGTACGCATACTAAAATATTACCATAGGCGTCTACTTTCAATAGGTTTCCATACAAGGTGTCGAACCACA

GACCTCGAACTGGGAATGAGGGATCATATTCAAATTCTAATATTTCTTGTGGATATCCCTTGGACACTAATCTCTCTTTG

GTTAAATTAAATCCTAAATTTTCATACTGTGGCGATTTGTACTCAGCCAGTGTGTAGTCCATATCAAACCCATAGAACTG

GACATTTTCCAAATGTAGCGATCTGTTTACAAAGATTCTGTGTTCTGCACGTCGATAATACTTTTTCGACGTCAGCTTTT

TTTTTTCTTGGCTCATTACACATTCCTTGTTAATGCAGGATTCCATGTTTGAAATAGTTGTGTGTGTCTCTAGTTTAAAT

AAAGGAGCACTTAAATTTATAAATCTTCGGTTTCTAGATGAGCTTAAGATTATTCTTGGTAACCTGAACATGCATAGGCA

ATGTTCTGTTACGTTTATTTTTAATTACCTAAAAGAAAACGTCATAAATTAATATGTATAATTATGTTTTGAATTTAACT

ATGTATTGTTTTCGTTTTTATCAGACGGTCATTACATTAGATATGTGATGGATGGCAAGACAACAACAACAAAACCAACT

AAAGAATATTGTGTTAGCCATTGATTTTCAATCAGAAGTTTATTAAATGCTTTTTTACATAATACATGTCTCACAAATAT

AATTAAAATTTTCACATCTTAAATCACACCCATACAGTGGGAAATCCAGGTTTTTCTCTTGTCTATTAACAAATTAATAT

TTATCCTAGTAGATTACAGGAAAAAATGCAAAAATCTTATGTTAAAAAGCTGTAATTATGTCATTACAGCTACTAATATC

AGCCTAGCATATTAACTCTTCAAATTATTTTTCACAAAAACTCAGCTGCATATGGGGGCATCAACCTCTAGCTAACCTGG

CTTGATCAGCCACTGATGGGATCAAGCCAAACTAACTCAAAGCAGGAATAGTTACTTGATATTTTCTCATCCAAAATAAA

GTTTCTTTTCTTTTTTTTCCTCTGTGTGTAGTCACAGTGAACAGACGTGTTTTTGTGAGCTCCTCAATTTAAAAAAAAGT

TTTGCTATTATATTTAAAATTATTAAATGAAAGACACTTAGCAAGTACACCACCCTCTTGCTAGTTAAAATAATTTATTT

TCAACACAATGGACAACAAATTTTCAAGCAGTTTGGCAGATCTGGAGCAGCTGTTCCATTCCCGTATGGCTGAATATGAA

GATAAACTGGTCAAAGCTTCTGCAGGAGCAGGTCAACCATCAGACAGCATAACCTCCATCAGCAATGACTTCAGGGACTT

CAAGACCTTTGTTTGGCAAGCACTGTCTAAGTTAAAATCCCAAGTTGAGTTGCTTGGCCATGGTTTTGACAGGCATGAGA

CCCTCATGCGAAGAAAGGTGCTGCTGCTTCATGGGGTGCCTTGCATGTTTGTCACCGCTTAGGCCCTTCCAGGCAGAAGA

CTCGACCCATCCTAGTGAGGTTTTTCAAGGTAGAGCATCGGGAGCTTGTTTGGGACGGCAAGAAGCAGTTAAAGGGCTCG

GGCATCACCATTTCTGAGTTTCTGACTCAGACTCGACATCAAGCCTTCCTTGCCGCCAGGAAACATTTCAGCGTCACGAA

GTGCTGGACCACTGAGGGCAGGATAGTGATCCTTTGCCCTGACAATATTCGGCGGAAGATTGAGACGGTAAGTGAGCTCA

ACGAGTTGACAACCCGTTTTCCTACTGGCGCCATTGAGACCTCCGGCTCCGACCCTGGTCCTCTTAAAGACGCCCCAGAG

ACCTCCAAGGCTCAGCGAAAGGCGAGGCGTCGGAACTGAAGACATCATGCACTTTCACTAATTCACTTCTAACTTCAATC

TCTTTTGCTTTCGTCGGTACTTCAATTTAAATTTGCGTTAACTATAATTCCGTTACTTTTAAAATAGTTCTATAACTTCT

TGAAGGTATAACTGTATTTTGTTGTGCACAGTTCTATATTTATTGTTGCCACTTTATTACTTTTAATATTTAATTTAAAG

GTCAGTGCACCTATAATTGTGTAATACATACTTTGCAGAATCTGTGGTTATTTCCTAGTGACATCTGTCAAATTGTGTCT

AACTGACATCTTCTTGCTGCGTTCATTAATACTTGCGCGTGAGTGTTTTACCTGATTTGGCTTCATGTCTTTTAATTTGT

TTCATTTTATTTTTTTATTTTTTTTTGTTTCTGTTTGTCGTTCTTTGTGTTGGAATGTACTTATTTCTTATTAATTCACC

GGCGAGAAGGGGTGGTCGTTGCTATTGCTACGTGCTTATTAGTTATTAATTTAGTGTAAAATATTATTATACATATATTA

ATTTATTTATTTTTAATTATTTAATGGATGCTCTCGACGAGCTTAATGATAGTTTTTTTTCCTGCTCTTCCGAATCTTGC

GGTTATGTTAGCGCGGACGACTTAAATTCTACTCCCTCTTTACACGAACAAATTTCTTCTCTTTTCTTTGAACATCGTAA

CAATTTTAACGTCGTGCATATTAATGCCCAGAGTGTTCCGAGTCACCATTCCGACCTTCTAGCATCCTTTAGCAACACAA

ACGTCGATGCCATTTTGATTTCGGAAACCTTCCTCAAACCTTCACTTCTCTCTACTCAATTTTCTCTTCCCGGCTTCGTT

CTCATTCGTAATGATAGAACTGGGAAAGGTGGTGGGGGTGTTGCCATTTACTTGCGAGCCCATTTACCTCATAAAATTGT

TCTGGCCTCACCCTCATTGTACTCCGAGTCATCCGAATACCTATTTCTTGAAATTTCTTTAAATTCTTCCAAAATTCTCC

TTGCTGTCCTCTATTCCCCCAACTTACGCATCGACTATTTCCGCTCTCTTGATTCCACTTTGAGTGAACTTTGCCCTATT

TACAAACATATCATCATCATGGGCGACTTTAACACATGTCTTCTTAAAAATGATTCACGTTCCACCAGACTTACTTCTCT

CCTTTCTTCTTATGATCTGCATGTCTTACCTCTCTCTGCTACTCACTTTGCTCCCAACTGTTCTCCTTCTCTATTAGACC

TTGCGATTGTGTCTTCCCTTGACAATGTCGCACGGCACGGTCAACTTACGGCAACTTTCTCTTACCACGATCTAATATTT

CTATCGTATCGTGTTCGCACTCCTAAGCCTAAGCCACAGTATTTGTATTTGCGTAATTACAATGCGATAGACATTGAAGC

TCTGCAGGCCGACGCTGCCGCACTTGACTGGTCACCTGTCTCTGATAGCCCCGGTACGGAACAAAAAGTGTCCTGCTTTA

ATTCGATGGTCCTTGGTTTAATGGACAAGCATGCACCTATCCGCCGTATTAAAATAAAGCATAAGCCAGCTCCGTGGATA

ACAGCTGAAATTAAAGTTCTTATGGTCCGCAGAGACAGAGCGAAGAATAAACTCAAGGGCAGGCCGTCTGATGTGAACCT

CGAATACTATAGGGCTCTGCGCAATCGCTGCAGCCGTCTGTGTAGAGATGCTAAAAGGCGCTACTTCCACGAATCACTTA

TCAACCGTAGTTCCTCCGAGGCTTGGAAATTTCTCAAATCCGTTGGTATCGGCAAATCTCCCATACACACCTGTACAGAC

ATGGATCTAAATGAATTAAATCTCTCTCTTTCTCAGCCGCCTATCCATTTAGATCCAAATGCCAAAACTGTCACTCTCAC

TGAGCTTCAGAACTTGCCGGCACCAAACTGTCCCTCCTTTTCATTTAGTCCTGTTTCTGAGGTTGACGTTGAAAAATCGC

TTCTTGCAATTACCTCCAATGCAATTGGCTGTGATAATGTGTGCTCGAGAATGATCCGTCTCATCCTTCCCCACCTACTC

CCGATCCTAACTCACATTTTCAATCATTCCTTATATACTTGTAATTTCCCATCCGCCTGGAAGCAGGCACATATCTTACC

TTTACCCAAGATCCCTAACCCCTCTGTATCATCTCATTTCAGACCCATTTCCATTCTCCCTTACCTCTCCAAAGTTCTCG

AGCATATCGTTCACAGGCAGATAACCGGTTTTTTAACGTCAAATTCTCTTATTTCTTCTTTCCAATCTGGTTTCCGTTCT

GGCCATAGTACCGTCACTGCCTTGTTGAAGGTGACTGACGACATCCGCTGGGCAATGGAGAAAAAGATGATGACGGTCCT

TGTCCTGCTTGACTTCAGCAGTGCCTTCAACTCGGTAGATTTTGATGTGCTACTTGGCATCCTAAAATCTCTAAATTTCT

CTTCTACATCCCTAGCATGGTTAGATTCTTACCTTCGGGGTCGCTCGCAGTGTGTTCGCCTTGAAGAGTCTCACTCTGAT

TGGTGCGAGCTCACGGCGGGGGTTCCCCAGGGGGGCGGCCTT

>TRINITY_DN14494_c0_g4_i1_Invader6_gagpol#LTR/Gypsy64-3168viatfastywithBLASTOPIA_gagpol,2frameshiftsand3stopcodons.[Drosophilafruitflygenus]

CCGTTATGTCCCCGTAAACTTGACTATGCCAACATTTTGTTCGAAGTCATGAATTCTACTTCAGATAAATACCCAACTTA

CGCCGAATATGCGCGACGCTCACTGTTACGGCTACGTATTGTTAAGGGTCTGAGTAACGAACTCGTAGTGCAGATTGTAG

TGCGTGGTATTATGGACCCACACGTTCGTGCAGCTGCCGCGAATGCTGATCTGACAATCGAAAATCTGGTATCGTTTTTA

GCAATCTATGTCAAGCCTGGCCGTGCTAAAAATGAGACGCGTGTACCCCCCTCTAATGCAATAAAAAGACGTCACGTTCC

CACGAATAACAAATGCTTTTCATGTGGTCAGACTGGACACATGAGCTACAAATGTTTTAAAAATCAAAAACCATCTGAAA

ACGTTGCAAAACCTTCAGGAAGTTTCTCAAAATTAACGTGCTCGTTCTGTAAGAAGATCGGGCATAAGGAAGCAGACTGT

TTTGCTAAACAGCGCTCAGAGGCACAAAACGGTAATCAAAGGAAAGTTAACCTATGTAGGGAACTCGCGAGTAGTTGTAG

CAATAGTGACGTGTCTCCTGCTGTTGTCCAAGGCATACCGGTAGATGTTTTGATAGATAGCGGCGCGATTAACATTTCAT

TAGTCTCTTCAGATGTTGCCAAATTATTTTCTGGTCCGCGAAAATCAACGCTTTGCGTTATTAAGGGGGTGAGCGATAGC

GAAATAGTTTCTCGAGAATACGTTACAGTCACCGTCGAGTTTGAACACGTTTCCCTCGAAGTAGACCTGGTAGTCGTACC

CGCATCATGTATGAACACTCCAATTATTATCGGTACAGATGTTCTTAACAGAGACGGCATAGTTTACATTAGAACAAAAG

ATAGACAATACTTAAGCCGATCCTCAAACGAAGTTCTTAAAGTAAACGCAACTCGAATTGATATGCAAAGTATAGTGAAC

ACTCCTCTACAGGGAGCTGATTTTACTGCATTAATGGTCGTTATTGATGAGTTCTCTGAGTTTTTAATATCTGGCACCGC

TACTACTACCGTGAAAACAGGACAAATGCATATCAGTTTGACAAGCGAAACGCCCATAGTTTACCACCCTTATCGTCTTT

CCTATAAAGAAAAACTAAAAGTTAGAGAGATTATTAAAGATTTATTAGATAAAAACATAATTAGAGAATCAGAATCAGAG

TATGCTAGTCCTGTACTATTGGTCAAAAAGAGGGACGGTTCTGATAGGCTATGTGTAGATTTTCGAGCCCTTAATCGGAT

CACGGTTAAAGACAGATATCCCCTCCCTCTCATAGATGACCACATCGATCGCCTAGGATCCTCTAAATACTTTTCGTGTT

TGGATATGGCTTCAGGGTTCCACCAGATCCCTATTCATGAGGACTCTATCCACAAAACTGGTTTCGTGACCCCTGAAGGC

CATTTTGAATATTTAAAGATGCCGTTCGGTTTATGCAATTCCCCCACTGTCTATCAGAGGATAATAAACGGTACTCTTCG

AAGGTTGATTGAGACTGGAAACGTTTTGGTTTACGTCGATGACGTCCTTCTCGTGAGTGAAACCATTGCTGATGGCATAA

AGCTATTACGCGAAGTGCTTAAAACGCTCACCGAAGCCGGCTTCTCAGTCAACCTCCGGAAGTGTTCATTTTTAACCAGC

GAAGTAGAATACCTCGGACGCATTATATCTCATGGTCAGGTTAGGCCGAGCCAGCATAAAATTGTAGCTCTAGTTAATTC

CCCGGCCCCGGGTAATGTAAAACAAGTCCGGCAGTTCTTGGGGTTGGCTGGATACTTTCGACGTTACATCAAAGGTTATG

CGACGAAAACAGCAAGTATTTCACGTTTGACAAAAAAGGATGTAAGGTTTGAATGGGGTCCTGAACAGGAGACTGTTCGC

CAAGATCTAATTAAACAACTAACGAGTGAACCTATACTTGCTATTTTCGACCCTAGCCTGCTTACTGAGCTCCACACTGA

TGCTAGCAGTGCTGGATATGGAGCCGTCCTAATGCAGGTTCATACCGATGGTACAAAACGAGTGGTTGCCTATTTCAGTA

AAGTAACACAGGGCGCTGAGAGTCGGTATCACTCGTACGAGTTGGAGACGCTGGCGGTCGTCAGGGCACTGCAGCACTTT

AGACATTATTTAATTGGGTTGAAATTTAAAATTATAACTGACTGCAATGCCCTGAAGTCAACCGAGCGCAAAAAGGACTT

GTTACCTCGAGTAGCGCGCTGGTGGATTTATCTGCAAGATTTTGATTTCACCATCGAGTATCGAAAAGGCACACTAATGT

CACATGCCGACTATTTGAGCAGGAATCCATTAGCGGCGCAAGTAAATCAGGTTGAAAGGCCTCGCAACTGGGCTCAGATT

GCGCAACACGCGGATAATGAGACCCAAGGGCTCATCCAAAAGTTGAACGAGGGTAATCTGGACCCGCGGCGTTACGTCTA

TCAACATGATGTGTTGTACTATCGTTTTTCACCTACCGGCGAGGAATCTCGCCTATTGTGTTATATCCCCAAGGGTCACA

GGTTAAGCTTATTACGAATATATCATGATGAGCATGAACATATCGGGGTAGAAAAGACACTAGATCTTATCCTTAGACAT

TTCTGGTTTCCTGGACTTAAAAGCTTTGTATCAAAATACGTCTCTCACTGTCTTACTTGTATATCAAAAAAACGCGTACC

TCGAGGCAAGACAACCCAGGCTGCGGCCCAATACATGGTGCCCTGGAAAGGAGAGTGGTGTCCTGAAACCTGTTCAGCGT

TTTTTGAAT

>TRINITY_DN14494_c0_g4_i2_Gypsy-30_DWil#LTR/Gypsy97-4039viatfastywithGypsy9_Dya,1frameshift.[Drosophilawillistoni]

TCCCCTCCCTCTCATAGATGACCCCATCGATCGCCTAGGATCCTCTAAATACTTTTCGTGTTTGGATATGGCTTCAGGGT

TCCACCAGATCCCTATTCATGAGGACTCTATCCACAAAACTGGTTTCGTGACCCCTGAAGGCCATTTTGAATATTTAAAG

ATGCCGTTCGGTTTATGCAATTCCCCCACTGTCTATCAGAGGATAATAAACGGTACTCTTCGAAGGTTGATTGAGACTGG

AAACGTTTTGGTTTACGTCGATGACGTCCTTCTCGTGAGTGAAACCATTGCTGATGGCATAAAGCTATTACGCGAAGTGC

TTAAAACGCTCACCGAAGCCGGCTTCTCAGTCAACCTCCGGAAGTGTTCATTTTTAACCAGCGAAGTAGAATACCTCGGA

CGCATTATATCTCATGGTCAGGTTAGGCCGAGCCAGCATAAAATTGTAGCTCTAGTTAATTCCCCGGCCCCGGGTAATGT

AAAACAAGTCCGGCAGTTCTTGGGGTTGGCTGGATACTTTCGACGTTACATCAAAGGTTATGCGACGAAAACAGCAAGTA

TTTCACGTTTGACAAAAAAGGATGTAAGGTTTGAATGGGGTCCTGAACAGGAGACTGTTCGCCAAGATCTAATTAAACAA

CTAACGAGTGAACCTATACTTGCTATTTTCGACCCTAGCCTGCTTACTGAGCTCCACACTGATGCTAGCAGTGCTGGATA

TGGAGCCGTCCTAATGCAGGTTCATACCGATGGTACAAAACGAGTGGTTGCCTATTTCAGTAAAGTAACACAGGGCGCTG

AGAGTCGGTATCACTCGTACGAGTTGGAGACGCTGGCGGTCGTCAGGGCACTGCAGCACTTTAGACATTATTTAATTGGG

TTGAAATTTAAAATTATAACTGACTGCAATGCCCTGAAGTCAACCGAGCGCAAAAAGGACTTGTTACCTCGAGTAGCGCG

CTGGTGGATTTATCTGCAAGATTTTGATTTCACCATCGAGTATCGAAAAGGCACACTAATGTCACATGCCGACTATTTGA

GCAGGAATCCATTAGCGGCGCAAGTAAATCAGGTTGAAAGGCCTCGCAACTGGGCTCAGATTGCGCAACACGCGGATAAT

GAGACCCAAGGGCTCATCCAAAAGTTGAACGAGGGTAATCTGGACCCGCGGCGTTACGTCTATCAACATGATGTGTTGTA

CTATCGTTTTTCACCTACCGGCGAGGAATCTCGCCTATTGTGTTATATCCCCAAGGGTCACAGGTTAAGCTTATTACGAA

TATATCATGATGAGCATGAACATATCGGGGTAGAAAAGACACTAGATCTTATCCTTAGACATTTCTGGTTTCCTGGACTT

AAAAGCTTTGTATCAAAATACGTCTCTCACTGTCTTACTTGTATATCAAAAAAACGCGTACCTCGAGCCCCACGACAACC

TATAACATCCTGGGAAAAATCCGACACTCCATTTAGTACTATCCACGTCGACGCTTTAGGCCCTTTGCCCGAATCTAATG

GGTACAAGTTTGTCCTGATTATGATTGACCCCTTCACAAAATATTGTCTCCTTTACTCCATGTACCGCCAAGAAGCAGAT

GAGTTAAAGCGCGTTTTTAACAACGCAATATCATTGTTCGGTGTTCCAAAATTAATAGTCTGCGACCGTGGTCGTATGTT

TGAAGCATCTAGTTTTGTGACTTTGATTACCGGAATGGGCTGCAACATTCACTATATTACACCAGAGATGCATCAAGCAA

ACGGCCAGGTAGAGCGTTATATACGCACCGTTCTAAACATGATTCGCATTGAGACGAGTAACAAAAATAGCCTTTGGTCT

GACCTGTTGTGGAAACTTCAATTAGTTCTGAATATCACTAAACAAAAAACCACAAAATCATCCGCTTTAAACTTGTTAAT

TGGTACTGAGGCGACTACACCGGTAATCCGTTCGTTAATCCGAGACTTGGCAGTGGAAAACGCTAACCCAAATCGCGAGG

CTTGGCGTGAGATATGCCGGAGCCGTGCAGGTGAGCTCCTTCGAAGGAACCAAGATCAACAAGATGCTCGCGTAAATCGA

CACCGTGAATCTCCAAAGGTTTTTAAATTAAATGACTTGGTGTTTGTGATTAAGTATTCACAGAGTACAGGGAAGTTGGA

CTCTGGAATGAGAGGCCCGTACAAAGTTACGAAAATCTTGCCAAGTGACCGATATGAGCTCCAGTTGCTGACCGGTGCTC

GAGGCAAGACAACCCAGGCTGCGGCCCAATACATGGTGCCCTGGAAAGGAGAGTGGTGTCCTGAAACCTGTTCAGCGTTT

TTTGAATGCGAAGATAATGATGATGACGCGCACGCAGGTCCCCCAGCCGAGCCACCTGCGATTCAAGATTGGGATCCAGC

TCAGGATACTGTCGGAGATGTGCCCGAGGACGGCGCACAGTCAGGAGAGGCCGTATTAGAGTAATAATGTTTTTGTACAG

CTGTTATCTGTGACGTCACATCCACGTCTATAAAACTAGTTGTCACTCGTACAGCGCCCCTTTTTGCCGGTAGACGCTCA

AGCGGACAGATCGTTGGATTTCGTCAGAGATAA

>TRINITY_DN14494_c0_g4_i4_Gypsy-8_DVir#LTR/Gypsy106-4068[Drosophilavirilis]

CCGTTATGTCCCCGTAAACTTGACTATGCCAACATTTTGTTCGAAGTCATGAATTCTACTTCAGATAAATACCCAACTTA

CGCCGAATATGCGCGACGCTCACTGTTACGGCTACGTATTGTTAAGGGTCTGAGTAACGAACTCGTAGTGCAGATTGTAG

TGCGTGGTATTATGGACCCACACGTTCGTGCAGCTGCCGCGAATGCTGATCTGACAATCGAAAATCTGGTATCGTTTTTA

GCAATCTATGTCAAGCCTGGCCGTGCTAAAAATGAGACGCGTGTACCCCCCTCTAATGCAATAAAAAGACGTCACGTTCC

CACGAATAACAAATGCTTTTCATGTGGTCAGACTGGACACATGAGCTACAAATGTTTTAAAAATCAAAAACCATCTGAAA

ACGTTGCAAAACCTTCAGGAAGTTTCTCAAAATTAACGTGCTCGTTCTGTAAGAAGATCGGGCATAAGGAAGCAGACTGT

TTTGCTAAACAGCGCTCAGAGGCACAAAACGGTAATCAAAGGAAAGTTAACCTATGTAGGGAACTCGCGAGTAGTTGTAG

CAATAGTGACGTGTCTCCTGCTGTTGTCCAAGGCATACCGGTAGATGTTTTGATAGATAGCGGCGCGATTAACATTTCAT

TAGTCTCTTCAGATGTTGCCAAATTATTTTCTGGTCCGCGAAAATCAACGCTTTGCGTTATTAAGGGGGTGAGCGATAGC

GAAATAGTTTCTCGAGAATACGTTACAGTCACCGTCGAGTTTGAACACGTTTCCCTCGAAGTAGACCTGGTAGTCGTACC

CGCATCATGTATGAACACTCCAATTATTATCGGTACAGATGTTCTTAACAGAGACGGCATAGTTTACATTAGAACAAAAG

ATAGACAATACTTAAGCCGATCCTCAAACGAAGTTCTTAAAGTAAACGCAACTCGAATTGATATGCAAAGTATAGTGAAC

ACTCCTCTACAGGGAGCTGATTTTACTGCATTAATGGTCGTTATTGATGAGTTCTCTGAGTTTTTAATATCTGGCACCGC

TACTACTACCGTGAAAACAGGACAAATGCATATCAGTTTGACAAGCGAAACGCCCATAGTTTACCACCCTTATCGTCTTT

CCTATAAAGAAAAACTAAAAGTTAGAGAGATTATTAAAGATTTATTAGATAAAAACATAATTAGAGAATCAGAATCAGAG

TATGCTAGTCCTGTACTATTGGTCAAAAAGAGGGACGGTTCTGATAGGCTATGTGTAGATTTTCGAGCCCTTAATCGGAT

CACGGTTAAAGACAGATATCCCCTCCCTCTCATAGATGACCACATCGATCGCCTAGGATCCTCTAAATACTTTTCGTGTT

TGGATATGGCTTCAGGGTTCCACCAGATCCCTATTCATGAGGACTCTATCCACAAAACTGGTTTCGTGACCCCTGAAGGC

CATTTTGAATATTTAAAGATGCCGTTCGGTTTATGCAATTCCCCCACTGTCTATCAGAGGATAATAAACGGTACTCTTCG

AAGGTTGATTGAGACTGGAAACGTTTTGGTTTACGTCGATGACGTCCTTCTCGTGAGTGAAACCATTGCTGATGGCATAA

AGCTATTACGCGAAGTGCTTAAAACGCTCACCGAAGCCGGCTTCTCAGTCAACCTCCGGAAGTGTTCATTTTTAACCAGC

GAAGTAGAATACCTCGGACGCATTATATCTCATGGTCAGGTTAGGCCGAGCCAGCATAAAATTGTAGCTCTAGTTAATTC

CCCGGCCCCGGGTAATGTAAAACAAGTCCGGCAGTTCTTGGGGTTGGCTGGATACTTTCGACGTTACATCAAAGGTTATG

CGACGAAAACAGCAAGTATTTCACGTTTGACAAAAAAGGATGTAAGGTTTGAATGGGGTCCTGAACAGGAGACTGTTCGC

CAAGATCTAATTAAACAACTAACGAGTGAACCTATACTTGCTATTTTCGACCCTAGCCTGCTTACTGAGCTCCACACTGA

TGCTAGCAGTGCTGGATATGGAGCCGTCCTAATGCAGGTTCATACCGATGGTACAAAACGAGTGGTTGCCTATTTCAGTA

AAGTAACACAGGGCGCTGAGAGTCGGTATCACTCGTACGAGTTGGAGACGCTGGCGGTCGTCAGGGCACTGCAGCACTTT

AGACATTATTTAATTGGGTTGAAATTTAAAATTATAACTGACTGCAATGCCCTGAAGTCAACCGAGCGCAAAAAGGACTT

GTTACCTCGAGTAGCGCGCTGGTGGATTTATCTGCAAGATTTTGATTTCACCATCGAGTATCGAAAAGGCACACTAATGT

CACATGCCGACTATTTGAGCAGGAATCCATTAGCGGCGCAAGTAAATCAGGTTGAAAGGCCTCGCAACTGGGCTCAGATT

GCGCAACACGCGGATAATGAGACCCAAGGGCTCATCCAAAAGTTGAACGAGGGTAATCTGGACCCGCGGCGTTACGTCTA

TCAACATGATGTGTTGTACTATCGTTTTTCACCTACCGGCGAGGAATCTCGCCTATTGTGTTATATCCCCAAGGGTCACA

GGTTAAGCTTATTACGAATATATCATGATGAGCATGAACATATCGGGGTAGAAAAGACACTAGATCTTATCCTTAGACAT

TTCTGGTTTCCTGGACTTAAAAGCTTTGTATCAAAATACGTCTCTCACTGTCTTACTTGTATATCAAAAAAACGCGTACC

TCGAGCCCCACGACAACCTATAACATCCTGGGAAAAATCCGACACTCCATTTAGTACTATCCACGTCGACGCTTTAGGCC

CTTTGCCCGAATCTAATGGGTACAAGTTTGTCCTGATTATGATTGACCCCTTCACAAAATATTGTCTCCTTTACTCCATG

TACCGCCAAGAAGCAGATGAGTTAAAGCGCGTTTTTAACAACGCAATATCATTGTTCGGTGTTCCAAAATTAATAGTCTG

CGACCGTGGTCGTATGTTTGAAGCATCTAGTTTTGTGACTTTGATTACCGGAATGGGCTGCAACATTCACTATATTACAC

CAGAGATGCATCAAGCAAACGGCCAGGTAGAGCGTTATATACGCACCGTTCTAAACATGATTCGCATTGAGACGAGTAAC

AAAAATAGCCTTTGGTCTGACCTGTTGTGGAAACTTCAATTAGTTCTGAATATCACTAAACAAAAAACCACAAAATCATC

CGCTTTAAACTTGTTAATTGGTACTGAGGCGACTACACCGGTAATCCGTTCGTTAATCCGAGACTTGGCAGTGGAAAACG

CTAACCCAAATCGCGAGGCTTGGCGTGAGATATGCCGGAGCCGTGCAGGTGAGCTCCTTCGAAGGAACCAAGATCAACAA

GATGCTCGCGTAAATCGACACCGTGAATCTCCAAAGGTTTTTAAATTAAATGACTTGGTGTTTGTGATTAAGTATTCACA

GAGTACAGGGAAGTTGGACTCTGGAATGAGAGGCCCGTACAAAGTTACGAAAATCTTGCCAAGTGACCGATATGAGCTCC

AGTTGCTGACCGGTGCTCGAGGCAAGACAACCCAGGCTGCGGCCCAATACATGGTGCCCTGGAAAGGAGAGTGGTGTCCT

GAAACCTGTTCAGCGTTTTTTGAATGTGAGTAATATCTTTGAGTATGTCACAGATTGGACTGCATAAATGTAGGTGTGTG

AAGCCCCTGTTACAGCTGCCCGAGCTGACCCGCGACGAGACGAGAACGGAGCTGGCAAGCTGCTGGCTGGATACGCGCCG

CGGTGCGACCTTCCAGACTCATTGTCATGTGTAATGACCAGCGCTGACTGCTATTCCATCAGTTTTGGACATGCGCTGCG

GTGCCTCTGTCTTGCAGACACGGCTTGTACGTGTAGTATCGGACTAGTGCTGCGGTACTCTTCCATTTGTTTTGGACATG

CGCTGCGGTGCCTCTGTCTTGCAGACACGGCTTGTGTAGTATGGGATTAGTGCTGCGGTACTTTTCCATTTGTTTCGGAC

ATGTGCTGCGGTATCATGATCCTGATAGTGTGCGTAAATGCGCTGCGGTGCATTTGGAATGGGAATGTGTGGTACCGGTC

GAAAGAGTAACTGTGTGAAAGCGATAGATATCTTAGCTGGTTTGTTTCTATAAAAGAGGGCCTTGAATGTGTGATGAACG

ATTGAGTAGGCAAACATAAGCCTCTTTGCTCCGTACTCATATTTACTTCATAATTTCAGGCGAAGATAATGATGATGACG

CGCACGCAGGTCCCCCAGCCGAGCCACCTGCGATTCAAGATTGGGATCCAGCTCAGGATACTGTCGGAGATGTGCCCGAG

GACGGCGCACAGTCAGGAGAGGCCGTATTAGAGTAATAATGTTTTTGTACAGCTGTTATCTGTGACGTCACATCCACGTC

TATAAAACTAGTTGTCACTCGTACAGCGCCCCTTTTTGCCGGTAGACGCTCAAGCGGACAGATCGTTGGATTTCGTCAGA

GATAA

>TRINITY_DN14420_c0_g3_i1_BEL-6_AA#LTR/Pao387-5549[Aedesaegypti]

AACCCTAACTTCTCCTAACTGCTGCTTCTACGTTGGAGGTTGTCCCTCCAAGGCGGGCGGCATGTCAACGCGTCGTCATC

AGCTGAGCATGTACCTGCGACTCTTGCGAGCCCACTACCTCTAGACGACGTCACCAGCGTAGTTTTCAAACGCCACCGCG

CGCCGCGCTTCATTCACCTTTTTTCGCTCGACACACGCGCACTATGGTGTGCATAAAATCGACATAAAAATAACAGTGTA

ATAAAACTGATTAAACGCCTAATTGCTATCCAAGAAGATTTTCATTCATCTCCAAGACATAATTTGGACCTCCGAAGCCG

GATACGGGAGAAGAAATCGTTGCGACCAGCATAAATCGGACGCACGAGGCCACCAGCGAAGTCATCTACGGACCCACGTC

ACGGCACCAGCATAAAACGGTCAGGCTATCATCCTTCTTAATCTTTAGTCCTTTTCTTTATTTGTGCTTCATTTGTGTTG

ATATTGTGTGACTTGCTTTATTCTCGTTTAATAATTTGAAACGCGTATAAGTTTTCGTTTGTCCTCTACCCTCAATTTAA

AAATATAAAATATATTTTGCAAATATCTTAACATAACTGTTTGGACTGCCTTTCACAAACTCCAAAATGACTGGAACTCC

ATCTAAAGAAAGTCGTAGCAGTGGTGAGGATTTACTAAAAGATTTAAAAAAAAGAAGAGGTGTTGTTCGTGGTAAATTTA

GACTATTCATGAAGTTTGTGGATTCGATAGAAAAAATAACTATTACTGAGACCATGAAAATAGAATTACAAACACGTTTA

CAAAACGTAGATTTATTACTACAGCAGTTTACAGAAATTCAGGACGAAATAGACTTAAAAATACCTGATAGTCAAGTAGA

AAAGGAATTATCTGAAAGGGAGTCTTTTGAAGCCAATTATTATGCTTATGTGACTAAAGCTAAGTGTGTTATTAACGTAG

ATGATGTTCAGAACAGTTCCAAAGTTTTTTGTAAAGTTAAATTACCTACAATTTCACTTCCTACCTTTGACGGATCGTAT

GATCATTGGCTAGAATACCGCGATACTTACCTATCTCTCGTCCATAACTCTAAAGAAATTGATAATGTGCAAAAATTCCA

TTACTTACGGTCTTCTCTTTCTGGCAGTGCATTATTGGTTATAAAATCATTAGAATTCTCCTCTGAGAATTACAGCGTAG

CATGGGAGTTATTAGAAAACAGATTCAATAGTAAACGTTTACTTGTGCAAAACCACGTCAAAGCTTTATTTTCTATGACG

TCACTTCATAAGGAATCATCCAGTCTTATTCGTAAACTTATTGATACCGTTCTCAAAAATCTACGTTCCCTCAAAAATCT

CGGCGAACCAACTGAGTCTTGGGACACGTTGATTATTTATTTAATTACATCTAAACTAGACTCTGTTACCGAACGAGAAT

GGGAGACTCATAAAAGTTCTATTTCGAATAGTCATAATCAGTCAAGTTCACGCATAACTGTTGATGATTTAATTACCTTT

CTTAAAAATAGGGCTGACGTGTTAGAAACCATTAACTTAGCACATAGTAAATCTTACACAGACAATAACACAAAGAAACC

ATCACATTCACAACAATCAAACAAATCACATAGTTACACATCAACACAAAATAAACAACATTATAATAAAAATCCGAAAC

GTTCTCGTATTTGCGCGATGTGTCAAGCTAATCATCCGCTTTATTTATGCGAATCATTCTTAAAATTAACTATTAACGAT

AGAATTAAATTCATTAATGAAAAGAAATTATGTTTTAATTGTTTACGCGCTAATCACTCGGTAGAAGAGTGTTGGCTCGG

TCCGTGTAAGCAGTGCAACAAAAAACACAACAGTCTAATTCATGATAAATGTGACGAAAGTGCGAGTGTATCCACATTGC

CAACAGTTGCGCATAATTCTCAGGCATCGTCACAGGATACAACTAATGGTAAGTCTTTAAATACTGCCACATTACATTCT

GCATCTCACACGACACATCAAAGTACTCATTCATTATTAGATACTGTACTGCTGTCTACAGCTTTAGTTGAAGTTGCAGA

TAAACGCGATAATTATCACACTACCCGCGCTTTACTGGATAACGGTAGTCAGCATTGTTTTGTAGCAGAATCGTTATGTA

AACGACTCAAACTTAATTTGGTACAGTCCACTGTGCAAGTCTCCGGAGTAGGTAACTCGGTGACTAATTCAACGCAATCT

TGCGAAATTAATTTGAGGTCTAAAACTACACAATACAATACACGCATGAAATGTTTAGTTTTAAATCAAATAACAGCTCA

GCTGCCATCTTTAGGTACAACATCATACATAAATATCCCTGATCATGTACAGTTAGCTGATCCCGAATTTTATTCGCCAT

CAGGAATCGAATTATTAATTGGGGCAGATAAATTTTGGGAGCTATTGAATGACGGACTAATTCGCTTAAGTAGTGGTCCA

TATCTCCAAAATACTAAGTTGGGATGGGTGATATCTGGCTCATTGTTGAATAATAAACCTTCGCGCAACAATCGAGTTCA

ATGCAATTACTCACAATCATTAGACGCTCAACTTAGGCAATTCTGGGAGTTAGAGGAATTAACTCCGCAAAATAAATTAA

CTAATGACGAGTCCTTCTGTGAAAACTTGTTTTTGCAAACTACGTACCGCGATGATAAGACGGGTCGGTTTTTTGTGCGC

ATTCCATTAGCCGAGTCTAGTGACACACTGGGAGATACGTACACGCTCGCGCTAAACAGGTTTCATGCACTTGAACGAAA

ATTAGATAAATCAACACCTGAATATAAAAAATTATATTGTGATTTCATGAATGAATATTTGGATTTAGGTCACATGAGTC

GAGTCAGTCACTATCCATCACCAAATTATTTTCTACCGCATCACGGTGTTTTAAGAATTCACAGTAGCACTACTAAACTA

AGGGTGGTTTTTGATGGAAGTGCAAAGTCAACTTCGGGCAAATCGCTTAACGATATCCAGCATATTGGCCCTAGTTTACA

TAATGACGTTTTTTCAATTTTGTTAAGATTTCGACAATTTAGATTCGTTGCATGCGCTGACGTCGAAAAAATGTATCGAC

AAATTAATATTCAACCGGATCAAAGAAATCTTCAATTAATTCTTTGGCGAGAAAATAAGTCTCACGAACTCGGTGTGTAC

CAGCTTAACACGGTGACATACGGTACCGCGTCCGCTCCTTATCTAAGCATTCGTTGCATCAAACAGCTGGCTTCGGAATG

TAAGGATGACGTCATTAAACAAGTTATCGACGAAGATCTGTTTGTAGATGACTTAATTACTGGTCATGATAATAAACAAT

GTTTATTAGACATTTGTACCAAAGTAAATAATGTTTTAAAATCTGGTTGCTTCCATTTACGTAAATGGTTGTTTAATTCG

GAACCATCTACAACAGATAAACCAAAAGAATTATCTTTAGGCGAAAATTGTTTAACTAAAACTCTCGGTCTCGGTTGGTT

AAGTTATTTAGATATTTTATATTTTACGACAAAATTTGATCAAAGTTTTACTCTTGTGACTAAGCGCGTTATCTTGTCCG

TTGTTTCACAAGTGTATGACCCATTAGGCTTGTTGTCGCCCTCAATTATAATTGCGAAAATACTTTTGCAAAAACTATGG

CTTTGTAAGTTAAGTTGGGACGATCCAGTACCTAATGATATTCTGTTAATGTGGAAAACCTTTATTGCCACTCTTCAACA

CTTACAAAACATAAAAATTCCTCGTCATGTTAGGGAAATAAATACAACTTATACAGATTTACACATATTTTCAGATGCGT

CACAGGATGCATATGGTGCATGTGCCTTTATAAGTACATACAACAAAACCTCACCAGCCACAGTTAGGATACTTTGTGCT

AAGACGAAAGTCGCGCCAATTAAACCAGTTACAATACCACGTCTTGAGCTGTGCGGAGCTTTAATTGGTGCCAAGTTATA

TCAGAAAATTGTTCAGTCGTGGAGACTAAAATTTGATAACATATATTTTTGGTCGGACTCCATGATTATCATAAATTGGA

TTAAAATGTCACCTAACCTGCTTAAAACATTCGTACAGAACAGAGTAGTGCAAATCAATGAATTGACTGGTGAGTTGCCA

TGGTTTCATATCGCTGGTAAAGACAACCCGGCTGATCTGCTATCTCGCGGGCTTACGCTCGACGCGCTTCAAAGTTCAGA

TATCTGGTGGTATGGGCCAGCATTCTTAAGAGAAATACATATCAATTTTGTACGTGATAACCCCAATGAAATTATTGATT

TTAACGATTTACCAGAAATGAAATCAAAGTCTTTATTTACACAATCAACTTTTACTAGTATTTTTATTTTTGAACGTTTT

TCAAATTTTAATCGCATGAGACGTGCAGCCGCTTACGTGTTACGATTCATTAATAATGCTCGACTTAAAGTTAAAACTGA

ACGTAACATTGGTCCGTTCACGGCTGATGAATTAAAGAAATCAACTTTAGTTCTAGTACGCTTAGCACAACATACATCAT

TTGCAGACATTTTTAATGCATTAACAAATAACTTACCTATTAAGTCGATGAGAAACATCTCTAGTCTTGATGTTTTCTTA

GATTCTGATTGCATTATAAGAGTGGGAGGTAGATTAGTAAATTCACATGCCTTTTCATACTCCAAAAAACACCCTATATT

ATTATGCTCAAAACATATCTTCACGCGATTACTTTTTCAATTTGAACACAAACGTCTACTCCATGCTGGTCCACAACTGC

TCTTGGCCACTATCAGAGAAAGTTGGTGGCCACTCAAGGGTCGTGATTTAGCTAAGCAGACGGTACATAAGTGCATTACA

TGTACACGCCAGAAAGGGAAGGCACTGTCAGTAAAAATGGGTAACTTACCTTCTGAGAGACTAGAGCCGGGGTATCCTTT

CTTGCGCTGCGGTGTAGATTATGCGGGACCTATGTTCATATTGAATCGGAAGGGAAGGGGTTCAAAACTTGAAAAATGTT

ATGTCTGCCTATTCATATGTTTTACCACTCGAGCGATACACTTAGAATTAGTCACAAGCTTAACTTCAGAGGCGTACTTA

TTGGCTTTGAAAAGATTCCTATCTCGTCGTGGAAAACCCGCTCAAATATTTTCCGATAACGGAAAAACGTTCGTAGGCGC

ACTCAAGGAATTTTCAAATTTTTTAAATAGCAATGCAAATGATATTGTAAATTTCGCATCAAGCGAAAATATTAAATTTA

GCTTTATACCTCCGTACTCACCACATTTTGGGGGTTTGTGGGAGGCAGGGGTCAAATCTTTTAAATATCATCTCCGTCGT

GTAGGAAACGTTAATCTCACGTATGAGGAATTTTCAACATTATTGACACAAATTGAAGCCCTCCTTAACTCCAGACCTAT

GTATCCCATGTCTTCAGATCCAAACGACCTACTTCCTCTTACACCAGCCCATTTTCTGATTGGCCGACCGCTCGTTGCAC

CTGCCTATGATGACCTCACAACTGCGAGCACATCACGCCTAGTCCGCTACGAGCGCATAGAACAGATGAAGCAAAACTTT

TGGAAACGCTGGTCGCAGGAGTACGTTTCGGAGCTGCAGACAAGGAGTAAATGGCAGTCCAACGCCAGCACGATAGAACC

CAATACCTTGGTGCTCATAAAAGAAGACAATCTGCCACCCTTAAAATGGCGTCTTGGACGAGTCCTAAGCACTTCACCTG

GGAGGGATGGACTAGCACGTGTAGCTGACATCAAAACAGCTAACGGAGTAATACGAAGAGCATATCCAAGATTGTGTCCG

CTGCTACAGGAGGAGGAACCCTAACTTCTCCTAACTGCTACTTCTACGTTGGAGGTTGTCACTCCAAGGCGGGCGGCATG

TCAACGCGTCGTCATCAGCTGAGCATGTACCTGCGACTCTTGCGAGCCCACTACCTCTAGACGACGTCACCAGCGTAGTT

TTCAAACGCCACCGCGCGCCGCGCTTCATTCACCTTTTTTCGCTCGACACACGCGCACTATGGTGTGCATAAAATCGACA

TAAAAATAACAGTGTAATAAAACTGATTAAACGCCTAATTGCTATCCAAGAAGATTTTCATTCATCTCCAAGACA

>TRINITY_DN14400_c0_g2_i1_Tc1_Ele13#DNA/TcMar-Tc1393-1480,1intron(964:1028)[Aedesaegypti]

GCGTTATCCTGCTGGAATTTGACACCATCCATATTCGTGTCGCCAAAAAGTTGCGTGAATGAAGGCAGTAATGCAGTTTC

TAGTACATTTATGTACTTAGTAGAGTCCATGCGACCCTCACAAACGAACAATTCGCCAACTCCAGAATCGGTCATGCAAC

CCCAGACCATTATGCTGCCTCCACCGTGTTTAACGGTTGGGATCACACACTCTGGCTTAAATTCTTCGCCCACACGGCGA

CGCACAAAGGTCACACCAGGTGTACCAAAAATCTGCAATAAAGAGAAAAATATTGAGTTCTGTAAAACTAAAATAAATTC

AACTATGCGTTTGGTTTCTAAAACATTTTATAAAATTATATCAGGGCACTAAATTGACTTACTCACCTCAAAGTTTGATT

CGTCTGACCACACAACATTTGACCAATCTTCAGCAGTGAAAGTTCGGTGTTTTAAAGCCCATTTCAATCGAGCTTTTTTG

TTGTTATCGGAGAGCCATGGCTTCTTCCTAGCTTTACAGCCTTTCAGACCAGCTTCTTGAAGTCTTCTGCGAGTAGTTCG

AGCAGAAATTGGCTTCTTAATTTGTTCCGACAACTCAGCAGCGAGCTCTGAAGATGTTTTTTTCCTATTTCTTAACGACT

CTCTCAGTAACTGACGATCTTGACGGTCTGTGGTGATGCGTTTGCGCCCGGTTCTCGGTCTATTTTCATGTGATCCGGTA

GTAGAGAATCGCTGAATAGCATATTGCACGGATCGGCGCGAACATTTCACAGTTTTAGAAATCTCTACTTGTGATTTCCC

TTGCTCATACAGAAGCTGTATCTGCACACGTTTTTCTAAAGAAAGTTCGTTTTGTTTTGGCATATTGCAACGATAACACT

TAAAACTTCTGAAATAAAATACAAAAGGCGCACTAGATCACTGGATGTGTTCACAACGAGCGATGGTAGCGAACTGATAA

ATAAACACAAAAATTGTAAAAACACATTTTATTGCACCCAGGTGTTTTATTGCTAGTTGTGCGGCGTGGGTCATTGTTTA

AACCTTTGTATATGGCCGACAAATGAATTCTTCAACAATAGATTTGGTTTACTCTCATTCTTTCCGTGAATGAGCGAAAT

TTGTAAGGTGCTCAATACTTTTGGCCAAGGCTGTATACATTTGGGTTTGGTCGTAAACCGTGTACCTAGTCAATAATGTA

ACGCTTTAAAATTGTCTGGAAAACGGTGTATTTGAAATAAGAACTAGATTTTGAACAAAAAGTACTGTGTTAAATGAATA

TTTCGTTAGTTCAATATCTCATGTAGTCTTATAATGATGAAAAACATGGCTAGAGTCCTGAAAACACAGTGTTGAGAAAC

CCTTTCTCGTTCATTTACTTCTGATGATACTCTTTAACAATGCTAACATAACAATGTTTACACACGTGTTCTAATAAATT

GTGTTTGTTCAAGGTATTTGTGAAGCTGATGAGACGTTACAAGTACTTGGAAAAAATGTTTGTGG

>TRINITY_DN14400_c0_g2_i2_Tc1_Ele13#DNA/TcMar-Tc1393-1480,1intron(964:1028)[Aedesaegypti]

GCGTTATCCTGCTGGAATTTGACACCATCCATATTCGTGTCGCCAAAAAGTTGCGTGAATGAAGGCAGTAATGCAGTTTC

TAGTACATTTATGTACTTAGTAGAGTCCATGCGACCCTCACAAACGAACAATTCGCCAACTCCAGAATCGGTCATGCAAC

CCCAGACCATTATGCTGCCTCCACCGTGTTTAACGGTTGGGATCACACACTCTGGCTTAAATTCTTCGCCCACACGGCGA

CGCACAAAGGTCACACCAGGTGTACCAAAAATCTCAAAGTTTGATTCGTCTGACCACACAACATTTGACCAATCTTCAGC

AGTGAAAGTTCGGTGTTTTAAAGCCCATTTCAATCGAGCTTTTTTGTTGTTATCGGAGAGCCATGGCTTCTTCCTAGCTT

TACAGCCTTTCAGACCAGCTTCTTGAAGTCTTCTGCGAGTAGTTCGAGCAGAAATTGGCTTCTTAATTTGTTCCGACAAC

TCAGCAGCGAGCTCTGAAGATGTTTTTTTCCTATTTCTTAACGACTCTCTCAGTAACTGACGATCTTGACGGTCTGTGGT

GATGCGTTTGCGCCCGGTTCTCGGTCTATTTTCATGTGATCCGGTAGTAGAGAATCGCTGAATAGCATATTGCACGGATC

GGCGCGAACATTTCACAGTTTTAGAAATCTCTACTTGTGATTTCCCTTGCTCATACAGAAGCTGTATCTGCACACGTTTT

TCTAAAGAAAGTTCGTTTTGTTTTGGCATATTGCAACGATAACACTTAAAACTTCTGAAATAAAATACAAAAGGCGCACT

AGATCACTGGATGTGTTCACAACGAGCGATGGTAGCGAACTGATAAATAAACACAAAAATTGTAAAAACACATTTTATTG

CACCCAGGTGTTTTATTGCTAGTTGTGCGGCGTGGGTCATTGTTTAAACCTTTGTATATGGCCGACAAATGAATTCTTCA

ACAATAGATTTGGTTTACTCTCATTCTTTCCGTGAATGAGCGAAATTTGTAAGGTGCTCAATACTTTTGGCCAAGGCTGT

ATACATTTGGGTTTGGTCGTAAACCGTGTACCTAGTCAATAATGTAACGCTTTAAAATTGTCTGGAAAACGGTGTATTTG

AAATAAGAACTAGATTTTGAACAAAAAGTACTGTGTTAAATGAATATTTCGTTAGTTCAATATCTCATGTAGTCTTATAA

TGATGAAAAACATGGCTAGAGTCCTGAAAACACAGTGTTGAGAAACCCTTTCTCGTTCATTTACTTCTGATGATACTCTT

TAACAATGCTAACATAACAATGTTTACACACGTGTTCTAATAAATTGTGTTTGTTCAAGGTATTTGTGAAGCTGATGAGA

CGTTACAAGTACTTGGAAAAAATGTTTGTGG

>TRINITY_DN14452_c0_g1_i2_Gypsy-22_DWil_gag#LTR/Gypsy1422-2393[Drosophilawillistoni]

AGAATGGACACTTGCTTGGGGCTGGTTACCCGTGTGGTCCTTGGATTTAGTAGAATGGACACTTGCTTGGGGCTGGTTAC

CCGTGTGGTCCTTGGATTTAGGTAAATTGGTGCCGTTGACATGGAAGGATTTTGTGAGAGGTCGTATTTTGGGCCTCACG

AGGCACCTCCGTGAGCCCACTATTCGTTTAAATCGTGGGTTTCGTCATATTGGGTATCATTTGTGCTCGTGGATGGTTGT

GGTTCCGGATTATAATAGTCATCTTCACTGATTACACAATTAACGCGAGCAGGTTTATAGTTCTCCTGATAAGGTTGCTT

TTGATATTTGGGAGATGGATTAGCCTTGAACCGATTATTGTTGTACTCGCGTTTTCTACATGTTTCAATGGTATGACCTT

GATTTTTACAATACCTACAGAATAGATTTTTATTATCTCGGTCATATTGTTTAGCCATTTGCGGCTTAGAATTGACATCA

TGGTTTGAAGGACGAGAAGGATATTGTGGTTTAAATGCAATACGATGTAATGTTGAACTTTGAGGATTACGCTTATTATA

CGAATTTAGGATCTTCTCCTCTGAGACGGCAAAATTGATTGCATCGTTGAGAGTGGATGGGTTTCTACTGCGGACTAGAT

TAGAAATTTCGGGCTTAAGGCCAATTAAAAACGTGTGGAGTGCTAAGTCTTCCATAGCCGCTATACGACCAACTAATTCG

CTTTTCTTTTTATTGGATAATGTTACTTCTGTTAAGAGCTTCGAAAGGCAAGTTTCGAGGCGTAATGCGAATTGGTTAAC

CGGTTCATTTAGTTGTTGGCATTCCCGTAATTCAGTCATAAGATGGGCATAGTGTTTTCTTTCACCGAACTGTGTCTTAA

GGAAACTATTTAATTGGTCCCAAGAGTCGAATTCTTTAATGGAGCAAGCTACTTCGGCTTTGCCTTCTAATTGTGATAAT

ATATATTTAAATAATATTGGTTTTTGGGTATCAGAAGCTAAATCAATTGCGTTGTCACAATTAGTTAGAAATGAATTCAA

TTTTTCACGTGTGCCATCGAATGTTTTTATAAATTTAAATAGAGTTCCAATTTGAATATTTTCATTTTTTTCTAGTTTAA

GAGACATTTTATTAAACAAATTTTATAACGCGTCCACTAAAGATGTAGTTCGGTGTAATAGGCGGCAACTGTAATGTGAG

GTTTAGATAAGAGCGAGTGCGGTATCGAATGTCTTGATAGTGATAATTCCGAGTAATGGTTAACAATAACAGTGAGACAT

TTAGAATATAAAGTTTGCAAACAAAAAATAAATTAGAATTAGTAACGCGCACAAGAAAATAAACCTTTATGTTTAGTTCA

CTGGGTTTACGCACTTTCACGCACTTGTTTTCGAATTGTTAGAGATGAGTTGCACTTTCGCTGGGAAATATAATGTTTTC

AAGACTTACAGGAACACGTAGAAAATGTACTGGAACATGACCATTTCTTTGGTCTGTTGAAGTTGAAGAGGCTTTCCCTC

TCAGGAATCAGGCACGAAACAGGAACACAATCACAATACCCACGGGAAAAGTTATTCAGATTTTAGGATGTCCGAAAATA

GATATGAATATTGAATTATTATTATTTTATTTGAGTTCGAGTTAATTTTGGCACTTCGACACTTTTATGCTTTAAAATAG

TTTAGAATACGAGTCCAGAAGGAGACGTCCCAGGGATACCTTCACCATCGGTATCCCACCGCTGCCACCAAATTGTTATG

AGGATTCCCGAATTTCGGTTTTTAAAAAAAACAAAGAAATAAAACTTAGATGAATATTGAAGGACACTTTAATTACTTTA

GGACTCGGTAAATAGGGCAAGTGTCACGCGCGGATGATGGAATCGAACTGTCGCAAAGAGAGCGTCTTATTATTATGAAC

TATCAAAAGTACTGATAAGGGATATAGAAAGAGCCGTTGGGCACTATCATATACATTACATATATATAACAGTACGCACC

CCCAGCGCAAGGCTATTACGGATGGGTGCCACCGTACACGACGTTCCCTAACCAACCCCCA

>TRINITY_DN14442_c9_g2_i2_BEL-2_HMM#LTR/Pao231-5441[Heliconiusmelpomenemelpomene]

AGAGAAACGTTAGTAATATGGATATAACAAAATCCGAACGCTAAGTCACGTGGTCAAATCAAACGAATAGAAAAATCGTA

ACAATCAATACATCGATCGCTAAATCCCATACAAATGATACAAAACGGTAGCCAACATGCAATTTTGACAAGCGTTTACG

AATAGAAAACTAAACGATCGCCGGGACCCTGACGTTTATGACAAAAAATGGCGTCATTCTACAATCAGCTGTTTGGATGT

TTTTGAGAGTTTTGTTTTGTTTTTGTATTATTTAAATATAACTATTAACTATGCATTTACTTAATATGTTGCGGTGCTTT

CAAAATGAAGAAAGGTGACTCAAACGTCGGGAAAGAGACCTTAATCGCTAGTGCATAGGAACGTCTCAAGATATATTATG

CCAGATACTGTAGTTGTGCAACATTTTAGATTGAATAAATCTATGATTATCAGAACTTTGGATTAAAATGTCCTTGACAG

GAACAAAGGAGATTACGCTACTAATAAATGTTCAATATTTGCACAACAACGCCGTCTAGTGGCACCCAGCGGAAGCAACT

GAACGGCGCGAACATCGTGCGATCGGGACTGTTTCCGGCGGGTGGCGGGCACGCGGGCGGCAGCACTAGCGACAACTTCA

TACTGCATCGTGTGCACACTACGCTGTGTGGCCGGCAACGTTCTTCTTATTCGAAAAAACACTTTTGTTGTTTTTTATTA

AGTTAAATATACGTTTATATTTAATCTTAAATAAATACCTTCATTGAAGACATCAGTGCCTCCTGAATCGGATAGAAGAC

AGGAATCAGTCTATCCAGTAAAGTGACAACACGTGTCAAATCATCTTTGCCCGGATGTAGCTGCAAAATTCTACCATATA

ACCACTTGGCTGGAGGTAAGTTATCTTCCTTTACCAAAACTACATCCCCTATCTTACAGTCAGTGTCACGACTATTCCAT

TTATGCCTTTGGAAGAAGTTTGTCAAATATTCCTGTTTCCATCTACGCCAGAAGTGCTGCACCATACGTTGTACTAATTT

CCACCTGCGTAAAGTACTAATGGTGGCACTTTCATAATTGTAGTCAGGAGCCACTACCAATGGATGACCCACCAAGAAAT

GTCCAGGAGTTAGTGGTACAGGATCATCTGGATCGCTACTACCCTGAGATAAGGGCCTAGAATTTAGGCATGCCTCAATT

TGGGAAAGGACTGTGGACAGCTCTTCGAACGTCAAAGTTGAGTCACCTATGACGCGCTTCAGATGGTACTTTACACTTTT

TACCCCTGCTTCCCACAGTCCACCAAAATTGGGAGCATGGGGAGGGATAAAATGCCATTCCGTTTGAGAGGATGCAAGAG

CAGCTGCAATGTCTTTAAACATTGCATTCTCTTGGTTGAAAAGAACCTGTAACTCCTTGGCAGCGCCCACGAAATTGGTG

CCATTATCGCTCCATGTATGACGACACTGGCCGCGACGGGAAACGAACCGTTTGAACGCTGCTAGGAAACCCTCAGTAGT

TAAATCTGAAACTGCCTCGATGTGTACTGCGCGCGTGGCCATACATATAAATAAGAAGATGTATCCTTTATAGGACCGCT

GACCACGCCCTTTAGAGACTCTGATGTTAATAGGGCCCGCATAATCGACACCAGAGTTAAGGAATGGTTTGCTGGGTGTC

ACTCTACAAGCTGGAAGCTCTCCCATAAGCTGATGCTTTGTGGTCGCTGCATACCGAATACAGGGAACGCAACGGCGTAT

ATGAGCTCTTACCATATTTTTTGCGTGGCTGATCCAGTACTTGGTACATATAAAATTAAGTACCAGTTGCTTGCCCCCAT

GAAACATCTTATCGTGTGCGTCGGCTACAATTAACTTGGCCAAATTTGAATGATGTGGAATAACTATTGGATGCTTTGTG

TCATCGAGTAGTGAAGAATATCGCAGCCTTCCCCCAATGCGCAATATCTCAGATTGATCAATAAATACATTTAATTTATA

CAACGAACTCCTTTTATCTAATGAAACCTTATTTTTCAGGGCCGTTAATTCTGCATGAAACCATTGCTGTTGGCTTAGTC

TGATACACACATTCAAGGCTTCGTGAATCTCCGAAGCTGTCAGTTGCAGCAGGAGGTGACATCTTCACAGAATAATCAAA

CTCATCCGTGTTCCGGTTCCAGATTCGCATCCGGTCATCAGATCGTCCATGTAGAAGTCGGACTTCACCCTTTCAGCAGC

CAATGGATACGTACTACCTTCATCGCATGCAACCTGGTGCAATGACTTGACAGCTAAGTAAGGGGCGCTTGACGTGCCAA

ATGTGACAGTCAGCAGTCGGAAGTCCTCTATCTCTTTGTCCGGATCCTCTCTCCATACAATTCTTTGGAAATCTGCATCC

GATCGAGCTACTTTGATTTGGCGATACATTTTTACTATGTCTGCTGCCAAACAGATCGGATGTTGTCGCCATCTCATGAC

AATGTGTCTCAAATCTGCCTGAAGAGTGGGTCCTACCATTAAGGTGTCATTTAGGGAAACTCCGTTTGTCCCCTTACATG

ACGCGTCATAAACCACTCTTACTTTAGTGGTCAGTTTATCCTTTCTAACCACTGCGTGATGGGGCAAATAAACCGCATCA

CTTTTATTTCTTTCAGGTTCGTCGAGGATTTTCTCCATGTGGCCCAGCAATAAATACTCGTTTATCACCTCAGTAAAAAA

AA

>TRINITY_DN14442_c9_g2_i4_BEL-2_HMM#LTR/Pao231-5441[Heliconiusmelpomenemelpomene]

TCAGTCTATTTTGACTTTTTGAATGGCACCCAGCGGAAGCAACTGAACGGCGCGAACATCGTGCGATCGGGACTGTTTCC

GGCGGGTGGCGGGCACGCGGGCGGCAGCACTAGCGACAACTTCATACTGCATCGTGTGCACACTACGCTGTGTGGCCGGC

AACGTTCTTCTTATTCGAAAAAACACTTTTGTTGTTTTTTATTAAGTTAAATATACGTTTATATTTAATCTTAAATAAAT

ACCTTCATTGAAGACATCAGTGCCTCCTGAATCGGATAGAAGACAGGAATCAGTCTATCCAGTAAAGTGACAACACGTGT

CAAATCATCTTTGCCCGGATGTAGCTGCAAAATTCTACCATATAACCACTTGGCTGGAGGTAAGTTATCTTCCTTTACCA

AAACTACATCCCCTATCTTACAGTCAGTGTCACGACTATTCCATTTATGCCTTTGGAAGAAGTTTGTCAAATATTCCTGT

TTCCATCTACGCCAGAAGTGCTGCACCATACGTTGTACTAATTTCCACCTGCGTAAAGTACTAATGGTGGCACTTTCATA

ATTGTAGTCAGGAGCCACTACCAATGGATGACCCACCAAGAAATGTCCAGGAGTTAGTGGTACAGGATCATCTGGATCGC

TACTACCCTGAGATAAGGGCCTAGAATTTAGGCATGCCTCAATTTGGGAAAGGACTGTGGACAGCTCTTCGAACGTCAAA

GTTGAGTCACCTATGACGCGCTTCAGATGGTACTTTACACTTTTTACCCCTGCTTCCCACAGTCCACCAAAATTGGGAGC

ATGGGGAGGGATAAAATGCCATTCCGTTTGAGAGGATGCAAGAGCAGCTGCAATGTCTTTAAACATTGCATTCTCTTGGT

TGAAAAGAACCTGTAACTCCTTGGCAGCGCCCACGAAATTGGTGCCATTATCGCTCCATGTATGACGACACTGGCCGCGA

CGGGAAACGAACCGTTTGAACGCTGCTAGGAAACCCTCAGTAGTTAAATCTGAAACTGCCTCGATGTGTACTGCGCGCGT

GGCCATACATATAAATAAGAAGATGTATCCTTTATAGGACCGCTGACCACGCCCTTTAGAGACTCTGATGTTAATAGGGC

CCGCATAATCGACACCAGAGTTAAGGAATGGTTTGCTGGGTGTCACTCTACAAGCTGGAAGCTCTCCCATAAGCTGATGC

TTTGTGGTCGCTGCATACCGAATACAGGGAACGCAACGGCGTATATGAGCTCTTACCATATTTTTTGCGTGGCTGATCCA

GTACTTGGTACATATAAAATTAAGTACCAGTTGCTTGCCCCCATGAAACATCTTATCGTGTGCGTCGGCTACAATTAACT

TGGCCAAATTTGAATGATGTGGAATAACTATTGGATGCTTTGTGTCATCGAGTAGTGAAGAATATCGCAGCCTTCCCCCA

ATGCGCAATATCTCAGATTGATCAATAAATACATTTAATTTATACAACGAACTCCTTTTATCTAATGAAACCTTATTTTT

CAGGGCCGTTAATTCTGCATGAAACCATTGCTGTTGGCTTAGTCTGATACACACATTCAAGGCTTCGTGAATCTCCGAAG

CTGTCAGTTGCAGCAGGAGGTGACATCTTCACAGAATAATCAAACTCATCCGTGTTCCGGTTCCAGATTCGCATCCGGTC

ATCAGATCGTCCATGTAGAAGTCGGACTTCACCCTTTCAGCAGCCAATGGATACGTACTACCTTCATCGCATGCAACCTG

GTGCAATGACTTGACAGCTAAGTAAGGGGCGCTTGACGTGCCAAATGTGACAGTCAGCAGTCGGAAGTCCTCTATCTCTT

TGTCCGGATCCTCTCTCCATACAATTCTTTGGAAATCTGCATCCGATCGAGCTACTTTGATTTGGCGATACATTTTTACT

ATGTCTGCTGCCAAACAGATCGGATGTTGTCGCCATCTCATGACAATGTGTCTCAAATCTGCCTGAAGAGTGGGTCCTAC

CATTAAGGTGTCATTTAGGGAAACTCCGTTTGTCCCCTTACATGACGCGTCATAAACCACTCTTACTTTAGTGGTCAGTT

TATCCTTTCTAACCACTGCGTGATGGGGCAAATAAACCGCATCACTTTTATTTCTTTCAGGTTCGTCGAGGATTTTCTCC

ATGTGGCCCAGCAATAAATACTCGTTTATCACCTCAGTAAAAAAAA

>TRINITY_DN14442_c9_g2_i5_BEL-2_HMM#LTR/Pao231-5441[Heliconiusmelpomenemelpomene]

TCGATCCCAACGCCATCTATCGAGCATGCAGGTACTATTTTATACAAAATATTGATTTTGGTTATGGAATGGCGTTGGAA

TCGATTACATCTTTATTATTAATATTTCTACATTGAACATTCCGCCCACCAAAAGGAATATGTTCCTTTTCGTACCGCCC

ACCATGAAGAAACTTAGCAACAATAAGAACAATGGACTGTATGGTAATTAAATACAATTTTTAATACTAAATTGTGGACG

GCTTCTTTACACTTAATTACAATATAATGGACAGATATGGTTTCAATCAAACATAACAACTACAAATTTTTTTTCCGTTT

GACAAAGTACTGAAAATAATATCATAAAACTAAACATTTTTATAAAAATATAGAGTTCGTGTGAACTCGATAAAAACAAA

AAAAAAACAAGGTAAAAATACATGGATGATGCCCAAGGTAGAACACCCTTTTTTTAAACAATAAACATAAAAGCTACAAC

CTTAACACTCGCTAAGCTTTAAGACACACAACAACACTTTAAGCTAAGCTCACAATTTCTAAATTTCTATTTTTAACATA

AATTGAGTTATTCGGTGATAGGAAGTAAACACAATTTTGAAATTGGACGTTTAATCGTAGTGCCTTTGCAACCAACAGTG

ACAACACGTGTCAAATCATCTTTGCCCGGATGTAGCTGCAAAATTCTACCATATAACCACTTGGCTGGAGGTAAGTTATC

TTCCTTTACCAAAACTACATCCCCTATCTTACAGTCAGTGTCACGACTATTCCATTTATGCCTTTGGAAGAAGTTTGTCA

AATATTCCTGTTTCCATCTACGCCAGAAGTGCTGCACCATACGTTGTACTAATTTCCACCTGCGTAAAGTACTAATGGTG

GCACTTTCATAATTGTAGTCAGGAGCCACTACCAATGGATGACCCACCAAGAAATGTCCAGGAGTTAGTGGTACAGGATC

ATCTGGATCGCTACTACCCTGAGATAAGGGCCTAGAATTTAGGCATGCCTCAATTTGGGAAAGGACTGTGGACAGCTCTT

CGAACGTCAAAGTTGAGTCACCTATGACGCGCTTCAGATGGTACTTTACACTTTTTACCCCTGCTTCCCACAGTCCACCA

AAATTGGGAGCATGGGGAGGGATAAAATGCCATTCCGTTTGAGAGGATGCAAGAGCAGCTGCAATGTCTTTAAACATTGC

ATTCTCTTGGTTGAAAAGAACCTGTAACTCCTTGGCAGCGCCCACGAAATTGGTGCCATTATCGCTCCATGTATGACGAC

ACTGGCCGCGACGGGAAACGAACCGTTTGAACGCTGCTAGGAAACCCTCAGTAGTTAAATCTGAAACTGCCTCGATGTGT

ACTGCGCGCGTGGCCATACATATAAATAAGAAGATGTATCCTTTATAGGACCGCTGACCACGCCCTTTAGAGACTCTGAT

GTTAATAGGGCCCGCATAATCGACACCAGAGTTAAGGAATGGTTTGCTGGGTGTCACTCTACAAGCTGGAAGCTCTCCCA

TAAGCTGATGCTTTGTGGTCGCTGCATACCGAATACAGGGAACGCAACGGCGTATATGAGCTCTTACCATATTTTTTGCG

TGGCTGATCCAGTACTTGGTACATATAAAATTAAGTACCAGTTGCTTGCCCCCATGAAACATCTTATCGTGTGCGTCGGC

TACAATTAACTTGGCCAAATTTGAATGATGTGGAATAACTATTGGATGCTTTGTGTCATCGAGTAGTGAAGAATATCGCA

GCCTTCCCCCAATGCGCAATATCTCAGATTGATCAATAAATACATTTAATTTATACAACGAACTCCTTTTATCTAATGAA

ACCTTATTTTTCAGGGCCGTTAATTCTGCATGAAACCATTGCTGTTGGCTTAGTCTGATACACACATTCAAGGCTTCGTG

AATCTCCGAAGCTGTCAGTTGCAGCAGGAGGTGACATCTTCACAGAATAATCAAACTCATCCGTGTTCCGGTTCCAGATT

CGCATCCGGTCATCAGATCGTCCATGTAGAAGTCGGACTTCACCCTTTCAGCAGCCAATGGATACGTACTACCTTCATCG

CATGCAACCTGGTGCAATGACTTGACAGCTAAGTAAGGGGCGCTTGACGTGCCAAATGTGACAGTCAGCAGTCGGAAGTC

CTCTATCTCTTTGTCCGGATCCTCTCTCCATACAATTCTTTGGAAATCTGCATCCGATCGAGCTACTTTGATTTGGCGAT

ACATTTTTACTATGTCTGCTGCCAAACAGATCGGATGTTGTCGCCATCTCATGACAATGTGTCTCAAATCTGCCTGAAGA

GTGGGTCCTACCATTAAGGTGTCATTTAGGGAAACTCCGTTTGTCCCCTTACATGACGCGTCATAAACCACTCTTACTTT

AGTGGTCAGTTTATCCTTTCTAACCACTGCGTGATGGGGCAAATAAACCGCATCACTTTTATTTCTTTCAGGTTCGTCGA

GGATTTTCTCCATGTGGCCCAGCAATAAATACTCGTTTATCACCTCAGTAAAAAAAA

>TRINITY_DN14405_c0_g1_i1_Mariner-36_HM_tp#DNA/TcMar-Fot11004-2788[Hydramagnipapillata]

CCATTCGCGGCGCGAGTATTTCGGGCGGCACTCGACGCGCCTCGGGTTTCACGCCGCCCGCAGATCCCACTGACACACAA

ACACTGTATTTTCGGCTCTGATAACGCTCCCGATGGTTTAGATGCCCTAATTCCATTGCCCCACTGTACACACGGTGCTG

GCGAAATTGCTTTAATGCGATAATGTTTTATCTGACAGATGTTTGTTAACCATCGTTAGAATGAACTGGTTTACTTTCGT

AAGTAATAACATGACTTAATCGCAGCGTAATAATGTAGCACGCTAGCCATGACTCTTTTCATTTCATTATAGGTGTCCTG

TTGATTAGTAGGGTAAACTTTCCTAAATCGTACTTTTTCCTAAAACATACCCCATCGAGTTCTTGTTTCGTGAAACTGCA

GTGGCGACGCAAGTGCTAGAAAATGAAACTACTAGTTGGCATTGTTCCGTGATCCGAGCAACGACTCCATGCTGCCGCTC

CTTTTAAAATTATTCAAAAAGTAAGTAATTCTGCTGTTTTCAAGTAATATTTTTGGTTCATTCATAAGGTTTTCAATTTT

CTTCACGCTGGGTTATGTAACTTCGAAGCATCTTTTTTGATTCTATACATAATTGCGTTTTAAATTACTGTACATTTATG

TTTAAGCCTGACAGCCATTTTGTTACGCGCCAAATTTGAAATCCTTAGTTAACTTGTGCCTAATTCGTACTCATCACTTT

GTTCTATTTCGTACCGGTACAAATTAGGAATAATTGTTTCTTTTTTTTGTTTAAGATCGACATGGCTAAGCGACGATACA

AAACTTGGTCAAGTGACGATATGGAAAAAGCCCTCAGTGCTTATAAAGATGGAAGTTTAAAATTTAATGAAACTTGTCGT

GTATTTAATATTCCAAAACCGACATTTCGGCGTCATTTAAAAGGATTGAATATGCACGAAGGAATTGGACGTCCGAAATA

TTTAACAAAAGCAATGGAAGACGAACTAGTTAACCATATATTGGAATTAGAATCCAGATTTTTTGGCGTTACCATACGTG

ATTTACGCCACTTAGCATACCAATTAGCGGAAAAATATGGTTTGCCTCACAAATTTAATCCGGAAACTAAACTAGCTGGG

AGGAAATGGTACTATAAATTTTTAAAAACCCATCCTCAAATATCTTTAAGGACTCCAGAACCGACATGCATGGCTCGCTG

CAAAGGCTTTAACAAAAAAACCGTTATGGAGTTTTTTGATAAATACGAAGCTCTTCTTGATGAAGGAAAATTTACGGCCC

AGCAAATTTACAACGTGGACGAGACTGGCTTGAGTACAGTGCATAAGCCTCAAAAAGTTCTAGCTCTTAAGGGTAAACAT

CAAGTTGGTGCCGTTACTAGTGGTGATCGGGGCTTGAACACAACATGTATCTGCTGTATGAACGCTGCCGGAGAATTCAT

TCCACCTATGCTAATATTCAAACGTAAAAGAATGACGGACGATCTTAAAAGAGGAGGACCACCAAATACAGTATACACTT

GCTCTGAGAGTGGCTGGATAACTTCTGAGTTATTCGTCGATTGGTTAAAGCATTTCATAAAGTCTGTAAAGCTGCAGATA

TCCAAAGAAAAACAGGTTCTTCTCATTTTAGATGGGCATTCCACGCATACTAAAAACTTGGATGCCATTAACCTTGCACG

AGACTATGGTATTGTAATGCTGTCCTTGCCACCGCACACCACACATAAACTTCAACCATTAGACCGGTCATTTTTTAAGC

CACTGAAGCAAAATTTCAATGCTGCTTGTTCATCATGGATGAGAAATCACCCAGACTCCGTAATTAAGCAGGCAAACATT

TTAAAAAATTTAGGGATTTCTTATCCTCGAGCTGTCTGTATGGAGACAGCCATACACGGATTTGAATCTTGTGGGTTATG

GCCTTGCAATCGATTGAAGATCAGAGATGACGAATATGTTATACTGGAAGATAATTATGAAGAGCTACTAGATGTAGGGC

CATCAGTTAGCTCCCATTCGACTGTGGCAGCGGAATCTGTAATTGAAGTACAGAATCAGCCAGCGGCATTGCCATCTGAT

ATTACACCAACGAAGACCCCTGCAGCATTTACCTCAGATAGAGAGGAGATTTCAAAGTTAGGTTCTCCGTCAGCGTCATC

TATTAGAAGTTTAACTAATACTCTGCAAAATCACGAACTTGAAGAAAATATTATCGTAGACATGCAACCCAACAACAGCG

AGAGTCCGAAATCAAACGCAATTCGAGCAACCATACAAAGCCTTTCTCCATTGATTAGTCTACATAAGCCAAAAAGGGTA

GTAAAAAATAAAACAACATCTCAAGTACTTACAGAAAGTCCTTATAAAAAAGAATTAGAAAATAAGTTAATAGATTCAAA

AAATAAAAAACCTAACGAGTCAAAAAAGAAGGCTGTATGTAAAAATTTAAGAGGACAAGAAGTCGTCAAGAGGAAGAAAT

CCGAAGAAAGAAAGGTTACGAAGAAAGCAAAAAAAGAAAAGGAGAACGAAGAACAAAGCTGGTATTGTTTCTTCTGTAAA

GAGAATAAAAAAGAAGACATGATACAGTGCACAGAGTGTGAAACCTGGATTCACGAACTTTGTGCCGGAGTTGACAAAAA

TGCACGTAATTTTATTTGTGATGATTGCAAAGAATTAGAAACAACAATAAGATATGCTATGCATACTTAAAATGTTAAAT

TTATATTAGAAATTATACCACAGATCTCGACAAACTAATCAGATTAGTTAAGCAATATAGTTACTATTACTTATGTATGT

CAATTACTGTGATTATGAGACTGATATTAATCATAGGATCTTAAGTTTGTTATAGAGATATACGGGTACGATTAAGGAAT

ATACAAGTACGTTTTAGGAAGATTTAAGCCTAAATCGTACCTTTTCCACTTTTTTTTTATTTAATTTTTTCTAAGAAGTT

ATCTAAGTTTGAGTTCCATTTGTTATTTTTGTTGATTACTTAATAAATAAAGATTCTTGAAACACCAAAAAAA

>TRINITY_DN14491_c0_g1_i2_L2-1_BM_pol#LINE/L2257-3202[Bombyxmori]

TTTTTTTTTTAGCTCTGTATAATCTTTTTTAATTAAAATTAAATTAATAAGTATTCTATACTGGATTAATGGTACTAATA

ATAGCTTAGACAAAGCTACCTGCCGGTGAAGTGTTTCCTTTCAATCACTGTCACAGTTTTCACTCCACATCAACAAACTG

TAATTTATCACAAATGGATCACTTAAACAGCACTGTGACTGGCCTGATGGATATCTTCCAAAAGCGGATGGCAGAATTTG

AAGAAAAGCTGCGGAAGGCGCCTGTGGACTCAAGCAACATCTCGACATTGGCCTCCGACTTTGCTGTCTTCAGGGATTTC

ATCACTGAGGCGGTCAATGGCCTGCAACAGCAGATAGAGTTGTTGGCCAAGACTGTAGACTCCATGGAGATGCGTGGGCG

ACGAAGTATCTTGCTGCTGCATGGCATCCCTGAGAGGAAAGATGAAGATACGGCGCAGGTGGTGGTCGACACAGTCAAAG

TACGCTTGAGGCTGGATGGGTTCACAACAGATGACATAAAGCGCTGTCACAGAATGGGGCGTTCCACCTCCTCTTCAAGA

TTACGTCCTATTTTAGTTAAGCTACGAGATGTGACGGTTCGTAATAATATATGGTTTGACAAAACCAAATTAAAGAGTAC

TGGAATCACTATCTCGGAGTTTTTGACAAAGTCAAGGCATGACCTGTTCATGGCTGCACGGAAAAGGTTTGGGGTAACCA

GGTGCTGGACAAGAGATGGATGTATTTATGTTCTGGCTCCCGATGGCTCGCGGCATCACATATCATCTGTTGCGGAGCTA

AACGCATTACAGCCGCAAGATACCACCGAGTCGCCGACGAATTCTGCTGCTAGTGCCACACCAAAGAAGCCTATAACCAT

GAGCAGGCCCAAGCGCGCTGCTGCCAATAAGAAGTAGTATCATTCGTTACAGTGCTGTGTCCGGGTAATACAATTTTTTC

TTTCTTCAATTCTTCACTACTCATGTCTGCCTACCCACTATGTTTCCTTTTGTAGTTTTGTCCATGTTACCCATTCCGTT

ATAAGCTTCTGTTCTTTTTATATGTAATACTTTCTAATTTTATTAAACAAGTGGGTATAAGTTTTCATTGCTGTGACGAA

TATGACATTGACAATGGACCAGCTGTCACTATGTCACGGATGCGTCAAAGACAATAAATGGGACTGGTATTCTCGTCATG

TTTGTTATTGCCTGTTACTGTCAGAGTGCTATTTTCGAATATCCTACCGTTTTGCCCGGACCACCACTATTAGTGTTAAA

TTATACTATTGTAATCTTTAAACATATCTTTCTTGAACAACCGGCGGGTGGATTTTGGCTGTCCCCTTTTAATATATTTT

TATTTATTTATAACTTAACACAACATACTTATTTATTTTATCACATTATACACTCTTTATGCCTTCTTCGTGTGATGATA

GTAGCGAACCAGATGTCTACTATTCAGCTAGTAACAGTGACGACAGCTTCACAAGTCTACCCTCTCTAGCTGATACACTA

ACTGCGCATTTTTCTGATGTTCCTGGGAATTTTAATGTAGTTCACATAAACGCACAGAGCATTCCGGCTCATTTTCCTGA

TATGCTGGCAAGTTTTGACTGTAAGAATGTTCACGCAATTTTAGTTTCTGAGTCGTGGCTCAAACCTTGCCTCCCATCTA

GTATCTACACGTTGCCAGGATTCCAGTTAGTTAGAAACGACCGCGCAAGTGGTGGCGGTGGTGGTGTTGCCATATATTTG

CGGACTTATATTTCCTTTTCAATTCTAAATGTGTCAGCGCAGCCTCCGCCACCGAATGCCGGGGAACATCTCTTCCTGGA

ACTTACAATGACTCACACTAAAATTCTACTTGGAGTTTATTACTGTCCGTCTTTGCGTACTAACTTTTTTTCTTCCTTAG

AAAACATTTTAGAAAACGTGGTAACTTCTTACAGTCACATTATAATCATGGGTGATTTTAACACCTGTCTTCTTAAAAAT

GATTACCGATCTGTGTCTCTTAACTCTATTGTCAATTCTTGCAATCTTTCAATACTTCCTCTTAACGCAACTCATCATTT

TCCTCATTCTACTCCCTCACTTCTTGATTTAATTTTTGTTTCTTCCTGTGATCACGTGGCCAAACACGGCCAATGTACGG

CTGATGCATTTTCCTATCATGACCTTATATTTCTTTCATATAAAATCCGTCCTCCTAAAGCAAAGTCAAGAATTCTTCTG

CAGCGTAATTTTGGTGGCATGGATCTTGGGAGACTTCGTGAGGATGCTGCTAATATTGACTGGTCGCCATTACTTTATTC

CCCAGGCATGGATGAAAAGGTAGAGATTTTTTCACGAAATCTAATCGCTCTGTACGACGTCCATGCTCCAGTTCGTTCAG

TTAAGGTGAGGCATCTACCTGCGCCTTGGCTCACCGAGGAAATAAAGCTGCTAATGGGCAAGAAGGTTGCAGCCAAGTCG

AGGTACAAGGCCTGCTCTGATCGTGCGAACTGGGAGAAATATGTTAATGTACGAAATCACTGCAACAAGGTGTGTAGGAA

TGCTCAACGCCATCACATTCACGCATCAGTTGAAAACGAAGATCCGGCTAAGGTCTGGAGATTTCTAAGATCAATGGGGA

TTGGAAAATCACGCTATGAAAATATTTCTCAAGATGTTAATATCGACTCATTGAACCAACATTTCTCTTCGTCTCCTAAT

TTTGATAGGATGACAAAACTAAATACTTTAAACACTCTTTCTAGTGTTCCAACCCCAGATTATTCTTCCTTCGTCTTTAG

TCAATTTGCCGCTTGTGATGTTAGGAAGAGCATTGCATCCATTACCTCTGATGCAGTTGGAAGCGACTGTGTCAGCCGTA

AAATGATCCTCCCTATCCTTGATGAAATCCTTCCTGTCATATGCCATTTCCTGAACTATTCCATATCCAGCGGTGTTTTT

CCGGACGCTTGGAAAGAAGCCCAAATTACTCCTTTACCAAAGAAAGCCAATCCTAAATCCTTTTCCGAGTATCGTCCCAT

ATCCATATTACCATTCCTATCCAAAGTCCTAGAACGTCTTATTCACAATCAGTTAAGTTCATTCCTATCCAAGAATAGAC

TTCTTAATCCCTTCCAATCCGGTTTCCGCCCTGGTCACAGTACCCTGACAGCCTTGGTTAACATTACGGATGACATCAGG

CTGGGCATGGAAAATGGGAAATTAACGGTTCTTGCTCTCCTAGACTTTAGTAATGCCTTTAACACCGTTGATTTCGACAT

TTTGCTTGGTATGCTTAGCTCTCTTAATGTATCTCCAACGGCCATTGACTGGTTTCACAGTTACCTGTACGGGCGCCGGC

AACGTGTTCGTATTGACGATAAATGTTCGTCATGGTGCGATACGACCGCTGGTGTGCCGCAGGGTGGCGTTTTATCTCCA

TTACTTTTCGCTATTTTTATAAATTCAATCACCAACAATCTCCTTTCTTCCTATCACCTTTATGCAGATGATCTTCAAAT

CTACTCACAGGCTCCTTTGTGTCTACTATCACAAGCCATTGGAAACATCAATAAAGACCTATCACATATAACTGAGTGGA

GTAGGTGTTATGGGCTTAAAATAAACCCTTTAAAAACTCAGCTTATCATCATAGGTAGCTCACGAATGGTCTCGAAAATA

GACTGGTTCCAGCTTCCCCAAGTTATCTTCGACGGAATCCAGATTCCCTTTAGTCCCGCAGTGAAGAACCTTGGGCTACA

TATGGATAGTAGGATGTCTTGGGATCTGCAGATGCAGGAAACCAGTAGGCGGTTATTTGCGTCTGCGGGATCCTTGAGAC

GGTTGCGGAATTTCCTACCCACTGCTACAAAAATTGCGCTTGCACAATCTCTTCTTCTCCCTGTTCTTGACTATGCTGAT

GCCAGCTATCTTGATCTCAGAGAGGACCAGCTGAATAAACTTGAACGTCTTCAGAATGTCTGTATAAGATTCATATTTGG

TTTGCGCAAGTATGATCATGTTTCAGAATTTCGCATAAAACTCAAGTGGCTTCCAATAAGACTTCGTCGGAATGCTCATA

TACTTTCCTTGCTTTATAATATTCTTTTCCACCCTACAACCCCTTTTTATCTCAAAAATCGTTTTGAGTTTATTTGTGAT

ACACATGCAAAATCTCTCAGGTCATCGGAAAATCTAAAACTGAAAATGCCAATGCACTCGACATCATTTTATGACAAATC

CTTCACGGTCCAAGCTACACGTCTGTGGAATGCATTGCCCGGAGGACCTGTTCATTACCTCGAAGCTGTGGAACACCTTC

CACCGTCCGGACCTGGTGAAGGGCGCGCTCCTCAAGTCGCTGGAGAACCTCAACATCAAGTACTTGGACCTCTACCTGAT

ACACTGGCCTCAGGCCTACAAGGAGGACGGCCCCCTATTCCCCACCGACGAGGCTGGTAAGATCCAGTTCTCGGAGGTGG

ACTACGTGGACACGTGGAAGGCGCTGGAGCCGCTGGTGGGCGAGGGCCTGGTCAGGAGCATCGGCGTCTCCAACTTCAAC

AGCAAGCAGCTCGCGAGGCTGCTGCAAGTGGCTACCATCAAGCCTGTTACCAACCAGGTCGAATGCCACCCCTACCTGAA

CCAGCGTCGTCTCAAAGACTTCTGCGAATCCCACGACATTAAGATCACAGCGTACTCCCCGCTCGGCTCGCCCGACAGAC

CCTGGGCCAAGCCTGATGACCCTCAGCTGATGGAGGACCCCAAGCTCAAGGCAATCGCTGATAGATTGGGGAAGACTGTC

GCGCAGGTGTTGATCAGATACCAGATTGACCGCGGCAACATCGTGATCCCGAAGTCCGTGACCAAGTCGCGCATCGCGAG

CAACTTCCAAGTGCTGGACTTCAAGCTGTCCCACGAGGATATGGCTCTCATCGACTCCTTCGACTGCAACGGACGGCTTG

TGCCTATG

>TRINITY_DN14405_c0_g1_i3_Mariner-36_HM_tp#DNA/TcMar-Fot11004-2788[Hydramagnipapillata]

CCATTCGCGGCGCGAGTATTTCGGGCGGCACTCGACGCGCCTCGGGTTTCACGCCGCCCGCAGATCCCACTGACACACAA

ACACTGTATTTTCGGCTCTGATAACGCTCCCGATGGTTTAGATGCCCTAATTCCATTGCCCCACTGTACACACGGTGCTG

GCGAAATTGCTTTAATGCGATAATGTTTTATCTGACAGATGTTTGTTAACCATCGTTAGAATGAACTGGTTTACTTTCGT

AAGTAATAACATGACTTAATCGCAGCGTAATAATGTAGCACGCTAGCCATGACTCTTTTCATTTCATTATAGGTGTCCTG

TTGATTAGTAGGGTAAACTTTCCTAAATCGTACTTTTTCCTAAAACATACCCCATCGAGTTCTTGTTTCGTGAAACTGCA

GTGGCGACGCAAGTGCTAGAAAATGAAACTACTAGTTGGCATTGTTCCGTGATCCGAGCAACGACTCCATGCTGCCGCTC

CTTTTAAAATTATTCAAAAAATCGACATGGCTAAGCGACGATACAAAACTTGGTCAAGTGACGATATGGAAAAAGCCCTC

AGTGCTTATAAAGATGGAAGTTTAAAATTTAATGAAACTTGTCGTGTATTTAATATTCCAAAACCGACATTTCGGCGTCA

TTTAAAAGGATTGAATATGCACGAAGGAATTGGACGTCCGAAATATTTAACAAAAGCAATGGAAGACGAACTAGTTAACC

ATATATTGGAATTAGAATCCAGATTTTTTGGCGTTACCATACGTGATTTACGCCACTTAGCATACCAATTAGCGGAAAAA

TATGGTTTGCCTCACAAATTTAATCCGGAAACTAAACTAGCTGGGAGGAAATGGTACTATAAATTTTTAAAAACCCATCC

TCAAATATCTTTAAGGACTCCAGAACCGACATGCATGGCTCGCTGCAAAGGCTTTAACAAAAAAACCGTTATGGAGTTTT

TTGATAAATACGAAGCTCTTCTTGATGAAGGAAAATTTACGGCCCAGCAAATTTACAACGTGGACGAGACTGGCTTGAGT

ACAGTGCATAAGCCTCAAAAAGTTCTAGCTCTTAAGGGTAAACATCAAGTTGGTGCCGTTACTAGTGGTGATCGGGGCTT

GAACACAACATGTATCTGCTGTATGAACGCTGCCGGAGAATTCATTCCACCTATGCTAATATTCAAACGTAAAAGAATGA

CGGACGATCTTAAAAGAGGAGGACCACCAAATACAGTATACACTTGCTCTGAGAGTGGCTGGATAACTTCTGAGTTATTC

GTCGATTGGTTAAAGCATTTCATAAAGTCTGTAAAGCTGCAGATATCCAAAGAAAAACAGGTTCTTCTCATTTTAGATGG

GCATTCCACGCATACTAAAAACTTGGATGCCATTAACCTTGCACGAGACTATGGTATTGTAATGCTGTCCTTGCCACCGC

ACACCACACATAAACTTCAACCATTAGACCGGTCATTTTTTAAGCCACTGAAGCAAAATTTCAATGCTGCTTGTTCATCA

TGGATGAGAAATCACCCAGACTCCGTAATTAAGCAGGCAAACATTTTAAAAAATTTAGGGATTTCTTATCCTCGAGCTGT

CTGTATGGAGACAGCCATACACGGATTTGAATCTTGTGGGTTATGGCCTTGCAATCGATTGAAGATCAGAGATGACGAAT

ATGTTATACTGGAAGATAATTATGAAGAGCTACTAGATGTAGGGCCATCAGTTAGCTCCCATTCGACTGTGGCAGCGGAA

TCTGTAATTGAAGTACAGAATCAGCCAGCGGCATTGCCATCTGATATTACACCAACGAAGACCCCTGCAGCATTTACCTC

AGATAGAGAGGAGATTTCAAAGTTAGGTTCTCCGTCAGCGTCATCTATTAGAAGTTTAACTAATACTCTGCAAAATCACG

AACTTGAAGAAAATATTATCGTAGACATGCAACCCAACAACAGCGAGAGTCCGAAATCAAACGCAATTCGAGCAACCATA

CAAAGCCTTTCTCCATTGATTAGTCTACATAAGCCAAAAAGGGTAGTAAAAAATAAAACAACATCTCAAGTACTTACAGA

AAGTCCTTATAAAAAAGAATTAGAAAATAAGTTAATAGATTCAAAAAATAAAAAACCTAACGAGTCAAAAAAGAAGGCTG

TATGTAAAAATTTAAGTGGACAAGAAGTCGTCAAGAGAAAGAAATCCGAAGAAAGAAAGGTTACGAAGAAAGCAAAAAAA

GAAAAGGAGAACGAAGAACAAAGCTGGTATTGTTTCTTCTGTAAAGAGAATAAAAAAGAAGACATGATACAGTGCACAGA

GTGTGAAACCTGGATTCACGAACTTTGTGCCGGAGTTGACAAAAATGCACGTAATTTTATTTGTGATGATTGCAAAGAAT

TAGAAACAACAATAAGATATGCTATGCATACTTAAAATGTTAAATTTATATTAGAAATTATACCACAGATCTCGACAAAC

TAATCAGATTAGTTAAGCAATATAGTTACTATTACTTATGTATGTCAATTACTGTGATTATGAGACTGATATTAATCATA

GGATCTTAAGTTTGTTATAGAGATATACGGGTACGATTAAGGAATATACAAGTACGTTTTAGGAAGATTTAAGCCTAAAT

CGTACCTTTTCCACTTTTTTTTTATTTAATTTTTTCTAAGAAGTTATCTAAGTTTGAGTTCCATTTGTTATTTTTGTTGA

TTACTTAATAAATAAAGATTCTTGAAACACCAAAAAAA

>TRINITY_DN14403_c6_g2_i1_Proto2-1_BM#LINE/Proto217-1009Fragment.[Bombyxmori]

TTCCGATCTTCGCAGAAGCAGGTAGTCGCGCGGTCCGCTGTCGTGTAGCACCACGCCGAGTGTTGCGAACTAACTTGAAT

CATAGTCGTCTGAGTCAGTGTGGCGCGCGTGTTCAGTGTGATGGTGAACAGTATCTTAGTGCATTCCTAGTCGACGTGTA

ATGCACGCCATGAGTGACCCGGCTGCTTTGGCAGGCATGTTGCCTTTTGACTCCATTGGACTGTATGAACAGCCTAAACC

ACGTTTCATTTTCAAAATGCCTCGGGTAGTTCCTGATCAGAAAGCTAAATTCGAATCTGATGATCTCTTTAAAAGGCTAA

GCCGCGAAAGTGAGGTGCGGTACACTGGGTACCGGGACAGGCCGCCGGAGGAGCGCCAGATGCGCTTCCAGAGCGGCTGC

CGTGAGGGTCACACCGAGATCGCATTCACCGCCACCGGCACCAACCTGCAACTCGTGTTCGACCACTCGCCATACAACAA

CCGTGGCTGCGACTTTCAGAAAGAGAGCGGAAAGTTGGGACCATCGGGGGAGATGCTCAATGCTGAGAAACCTGAAGGAA

AGGTGGGCTTACGTTTTAGGGCCAAGGACGTGGAGAACGTGATCAGACACATGTCGCGAGGTAAGTCCCCTGGCCATGAT

GGTCTGAGCATTGAGCACCTTCAGTACGGTGGTGCTCATATTTCGCGAGTGTTATCACTATTCTGTAACCTATGTATGAG

TCATAGTTATTTACCACCCGATTTAATAAAAACCATAGTGATACCAGTTGTTAAAAATAAATCTGGGGACATGTCGGATC

TGGGCAACTATAGGCCGATATCCTTGGCCACCATTATAGCTAAAGTACTTGATAGATTGCTTGAGATACAGTTAAATAAA

CATCTTCAAATACATGACAATCAGTTTGGGTTTAGGCCCAAACTCTCAACCGAATGTGCAATTATGTGTCTCAAGCAAAC

TGTCAGATATTATACAGATCGGGGAACGCCAATATATGCGTGTTTTTTAGACCTTTCTAAGGCTTTTGACCTGGTTTCCT

ACGACATACTCTGGAAAAAGTTAAAAGAGGAGAATACGCCTCCAGAAGTCATAAATATCTTTAGGTATTGGTATGGAAAC

CAGGTCAACAATGTAAGGTGGGCAGGGCAACTGTCTGATTCGTATAAAATGGAGTGTGGTGTGAGGCAGGGAGGGTTGAC

CTCTCCTGTACTCTTTAATTTATATATTAATGCACTGATTGTCACGCTCAGCAACGAACATGTCGGATGCCATATTGATG

ACGTATGCGTCAATAATCTCAGCTATGCTGACGATATGGTGCTGTTGAGCGCCTCAGTCTGTGGTCTAAGGAGGCTGCTT

TGTTTATGTGAGGGGTATGCACAAACCCATGGACTTAAATATAATGAAAAAAAGAGCCAGGTTATGGTCTTTAAATCCGG

AACTAAATATCCCACCAATATTCCCCCGCTAAAAATAAATGGAACCTCCTTAGAAAGAGTAGATAGTTTTAAATACCTAG

GTCATGTGGTTGCCTCAGATTTACGTGACGACATGGACATGGAACGGGAACGAAGGGCTTTATCGGTAAGAGCTAATATG

GTATCTCGTAGGTTTGTACGTTGCTCCACTGGTGCTAAAACAACCTTGTTTAGAGCGTATTGCACAACATTCTACACGTG

CAGCCTATGGATTACATATACTCGAAGATCGTACGAATCCCTTCGGATCCAGTATAATAATGCGTTCAGGATGCTGTTGG

GGTTGCCGCGTTATTGTAGTGCCTCCGGCATGTTCGCGGACGCGCATGTTGACGGTTTTAGCGCGATAATGCGAAAACGC

ATCGCTTCAATGGTCAGCAGAGTACGCGCCAGCACCAACAGCATCCTGAACATGATAGCTTATAGGGTAGATTCCCCGTA

CATGAATAATGTTATAAGACTTCATGTCTAATCTATTCTGTATGTTGCTGTATTTTTATATGTTTATATTGTGATTGTGA

CTTACTAACCATATTATTCATGTATTACTAACAAGTGGATTATGTTATCTTAATAAATAAATTAAATTAAATTAAATTAA

ATTAAATTAA

>TRINITY_DN14403_c6_g2_i2_Proto2-1_BM#LINE/Proto217-1009Fragment.[Bombyxmori]

TTTTTTTTTTTATTCTAAATTTAGGCACTTTATTTTCTTCACTTCGTCTTATTTACATATTTACAAATGATTTGATTAAG

GCTAGGACATAACATTTAGCTTTCTTTCTCAAGATTCAAAAACATTATTGGGTACATGTGTCCAAGTTTTGATATTCTCT

TCTGTATTATCAATCTTAAAGTGTCCGTGTACGTGGCGGGTGGTTTGCACGTTGAGGGATCGCGCCAATGAAGACCAGTG

GAGTCACAAGCAAAAAGCGCCGGCGAAGGCACGTGATCTCTCGTATAGTTCCATAGACAATCACGTAATACTTGCACTAT

CTCGCTAGCTGTTAGTTCAGGCGCTAGTTCGAGCATGTGGTTGAGTGGGTGCGGCAGTAACTGTCCCAGTGTCATAGTGG

ATAGTAGCTCGGTAAGCGCAGCGCGTTCCTTTGCCATCTCAGCAGACTGTGTGTGACGATCTAGGACAATATTTTTTTAA

ATTTAATAATCTCTTTCTGGGCCCTGGAGTTCTAAATGGAAATATTATTTACACAGTCGCGCGCACGCATCGCTTTGTGA

CGGGTGTGTGATCGGTACCGTCAATATGTCGAGTACGTTAAAGTGCAGTGTTTGTAACATTGTGATAGACGAGTTGCTCT

CGTATATACAAAATAAAATATCGGTGATAGACGAAATTACATTAATGAGAATATGTACAACGTCTTTCTCAAGCGAAGAA

ATACAGAAATCAAAATCGCTGTTGTTTGATTCTCTGTCAACGGATGTACGTAAAATATCTAGAAAACGTAAAGGAAAGGA

AGTTAGAGATTTGGAAGACATCATTAGTCTTTTCAAAGTAACAGATCCTGATGCAATTCCAATATTTGTGGCGAGGCAAT

TAGAAAAATTGCCGCCTATTACATTTGATCATCTTGATTGTACGAAACTTCTCAAGGATTTGCTGCTCGTAAAAGAGGAA

ATTGAGCAAGTTAAGACGACATATGCTTCGGTCAAGCAGCTTGAAGACCTAAGAAACGAAGTTATCGGATTAAAAAACGA

CAGAATAATGTATACCCCAGCACAATACATTAATAACAAAAGTGGCGCATGGATTGATAATAATGAAGCTAATGCTATTA

ATGACTCAACGATAGAAGTAAATAGTGTGTGTGATAAATTACAAAAAGAGGAACTTGTTGCAAACAGTAAGTTGCAGAAT

AATTCCAATTGTAGGTATCCGAAAATAAGCGACAAAGAAATCGTGAACTGCGTTAACGAGGAGCCGAGTGTCGTAGTGAC

TCATCGTGATATGCAAAAATCGCCTCGGACGCCGCCGCCGCTCGCGCCGGTAACTGAGAACAAAACTAAACAACAAAAGG

ACGATCAGCTGACTGTGTTGATAACGAGTGCGCCCGTATCACCTAATGCAGCGGAATCGGAATGCGAGTCGCGAAATAAT

ACTGAGTGGAGAACTATATCTTACAAGAAAAGATCGAATTATAGGTATGCAGGCAAAGCGGGAACTGCGCGTGACGTTCA

AGGAAGTTTCAAGGCGGCTGAAAGGAAAGTTCCTATTTTCATTTCTAATGTCCATAAAGATACGATGGCTAAGGACATAG

TGTCTTACATACAAAGTAAAACACAGGATGTTGTATCATTAGAAAAGATAAACACTAAGAAACAAAAAGAACACAACGCA

TACAAGTTCTTTGTATCAGAAAGTAAATTATCTTTATTCTTAGATGAAAATATATGGCCTGAAGGCATTATATTTAGGCG

TTTCGTGCATTTTAAAAGCAAATATGTAAGAGATATGTCAACAAAAACGGCCACAAATCAAACATATAATGCATAGCACC

TTTACGTTAACTAGTTTTAACTGTAAAAACGTTAAACGTTCCGTCGATAACATACGAGAAATGTGCCGTTTTTCTGATAT

CATTGCGCTTCAGGAGACGTGGCTGTTACCGGATGAGATCCAGTACCTGAGCACTATTGACAGAAAATTTAGTTCAACTG

GTACTTCTGCCGTTGACACAGCAGCTGGCTTGCTGCGTGGCCGCCCGTACGGAGGTGTTGCGCTGTTATGGCGCAGTGAC

GTGTTTCAAAACGTATCTGTTATACAGTGTAATAATCCGCGCGTGTGTGCCATTAAAATCGTTACAAACGACAGACCTAT

TGTGGTAATTAGTGTCTACATGCCCACAGACTCGCCGGATAACCTGTCGGACTTTACGGACTGTCTGGGTACGGTGAGTG

CTTTAACGGATGACTGTATGATAGAATCTGTATATGTGATGGGTGATTTTAATGCCCACCCTAATGAATTGTTTTTTAGA

GAACTCTCTGCTTTTACTAGTGAACAAAATTTGTTATGTATTGATGTTGAAAGGTTGGGTATAAATAGTGATACTTATAC

ATTTGTGAGTGAGGCGCATGGATCCAGGAGATGGTTGGATCATTGTTTGATTACACAGGCTACTGTGCCGACTATTAATA

ATGTATACGTGAAATATGATGTGCTATGGTCTGACCACTTTCCCCTCATACTAGAATGTAATTTTAACCTTTTAACACCA

AAGGTATGTCATAAGTCAGTAACAGTAAATAATGCAGTATGGGGAGAGAGAAGTCCGGAGCAGATTGATTCCTATCGCAG

TGTCTGTCATGAAAACTTGCGTTTAATTGATTTTCCATCGGAGTTTAGACATTGTTGTGATAGTTTTTGTAACAATATTT

CTCATAAACCGGTCATTGACCGATTATATCGTAATATTGTAACGGCCCTCACAGATGCGGCAATATTAAGTAAACAAAGT

AGGAAACAAAAAAAAATGTGTATTAATAGGGTGGAATAGGCATGTCAGAGATGCTCACAGGGAGGCCAGGTTAAACTTTC

AGTGCTGGGTCTTAAGTGGAAAACCGTATTGTGGTCGTGAATATGAAAAAATGTGTGCTAGTAGGAAGATATTCAAATCC

CGTTTAAAATGGTGCCAAAATAATCAGCACCAAATAAAAATGGATATCATTGCCGATAAACATGCATCGAAAGATTTTCG

TGGCTTCTGGAAATCCACAAGTAAATTGAATAGTGGGCCTGGTCTGCCTGTGAGTGTCAATGGCTGCAGTGATACGAGAG

ATATTGCTAATTTATTCAAGGATCACTTCTATGTGCGTTCACAGTTGGGACCATCGGGGGAGATGCTCAATGCTGAGAAA

CCTGAAGGAAAGGTGGGCTTACGTTTTAGGGCCAAGGACGTGGAGAACGTGATCAGACACATGTCGCGAGGTAAGTCCCC

TGGCCATGATGGTCTGAGCATTGAGCACCTTCAGTACGGTGGTGCTCATATTTCGCGAGTGTTATCACTATTCTGTAACC

TATGTATGAGTCATAGTTATTTACCACCCGATTTAATAAAAACCATAGTGATACCAGTTGTTAAAAATAAATCTGGGGAC

ATGTCGGATCTGGGCAACTATAGGCCGATATCCTTGGCCACCATTATAGCTAAAGTACTTGATAGATTGCTTGAGATACA

GTTAAATAAACATCTTCAAATACATGACAATCAGTTTGGGTTTAGGCCCAAACTCTCAACCGAATGTGCAATTATGTGTC

TCAAGCAAACTGTCAGATATTATACAGATCGGGGAACGCCAATATATGCGTGTTTTTTAGACCTTTCTAAGGCTTTTGAC

CTGGTTTCCTACGACATACTCTGGAAAAAGTTAAAAGAGGAGAATACGCCTCCAGAAGTCATAAATATCTTTAGGTATTG

GTATGGAAACCAGGTCAACAATGTAAGGTGGGCAGGGCAACTGTCTGATTCGTATAAAATGGAGTGTGGTGTGAGGCAGG

GAGGGTTGACCTCTCCTGTACTCTTTAATTTATATATTAATGCACTGATTGTCACGCTCAGCAACGAACATGTCGGATGC

CATATTGATGACGTATGCGTCAATAATCTCAGCTATGCTGACGATATGGTGCTGTTGAGCGCCTCAGTCTGTGGTCTAAG

GAGGCTGCTTTGTTTATGTGAGGGGTATGCACAAACCCATGGACTTAAATATAATGAAAAAAAGAGCCAGGTTATGGTCT

TTAAATCCGGAACTAAATATCCCACCAATATTCCCCCGCTAAAAATAAATGGAACCTCCTTAGAAAGAGTAGATAGTTTT

AAATACCTAGGTCATGTGGTTGCCTCAGATTTACGTGACGACATGGACATGGAACGGGAACGAAGGGCTTTATCGGTAAG

AGCTAATATGGTATCTCGTAGGTTTGTACGTTGCTCCACTGGTGCTAAAACAACCTTGTTTAGAGCGTATTGCACAACAT

TCTACACGTGCAGCCTATGGATTACATATACTCGAAGATCGTACGAATCCCTTCGGATCCAGTATAATAATGCGTTCAGG

ATGCTGTTGGGGTTGCCGCGTTATTGTAGTGCCTCCGGCATGTTCGCGGACGCGCATGTTGACGGTTTTAGCGCGATAAT

GCGAAAACGCATCGCTTCAATGGTCAGCAGAGTACGCGCCAGCACCAACAGCATCCTGAACATGATAGCTTATAGGGTAG

ATTCCCCGTACATGAATAATGTTATAAGACTTCATGTCTAATCTATTCTGTATGTTGCTGTATTTTTATATGTTTATATT

GTGATTGTGACTTACTAACCATATTATTCATGTATTACTAACAAGTGGATTATGTTATCTTAATAAATAAATTAAATTAA

ATTAAATTAAATTAAATTAA

>TRINITY_DN14440_c0_g1_i1_Tc1-1_Xt_tp#DNA/TcMar-Tc1394-1416[Xenopussubgenus]

ATAAAATAATATTTGGTAATGTGAAGAAACATTTATTTATATTATTGTTAATAAAGTTAAAAACATAATTTATCATTTTG

TATTATATTAAAATTAAAATAGAAGTGTTATTATACTTCTGACTTTATGAGATTATAGCTTAATATATTCATTTTCTCAA

AGAGAAAAGAAGATTGAAGCTTAATCCCAATCTTAAAATCAAAATAAATTACGTATGACTTTATATTATGACCAGCACGC

CCACTGTAAAAAACAGCGTAATCAATATTTAGTATGGCCTCCATTATTTTCAATGCAGCTAAGCAGACGTCGCCGCATGG

AGGCCACAATGTTTGCACATATATCTGTGCCGCGTAAATTCTCCCACAGTTGAGTGCAATGGGATTTAAGCTGCTCAGGA

CATCTCTCATCTCGCGCTTCCCATCTTTGAACCATTAAACCCCATATATTTTCAATGGGGTTTAGATCAGGGGAACGTGC

TGGCCACGGAATAACTTTAATATCATTATGCTTGCTAAACCACTCTTTTACAATACTGGCACGATGTATCGGACAATTAT

CTTGAACAAAATTTAGTTCACTACATTCTTCTATGGGGTAAACATTACGCACAGTAGGTAGCATTATGTCATTTAAAACT

TCAACGTAATTAGAGCTGTTCGCACGCGTAGGTATGAATTCCAGTTCACCCGGCCCTTGAGCACTAATCCACCCCCACAT

GTTCACACTTATTCTACCGGATTCTGTATTCGGAATAATGTTTTGCTCAGACCAGCGAGTATTATCTTTTCGCCATAAAT

GAAGTCTTCCTTTCTGGCAAGATTTGAAAGTCTTTTCATCGGTAAATATGGTTGTATCCCAGTTAAAATCTAAATATTCT

CGAGCAAAACGTAGTCGTTCCTCCTTGTGTTTTTGTGTCAAAAACGGTTTTTTAGCTGGTTTCCGGTGATGTAAACCTTC

AGCATGGAGTGCACGGCGAACAGTTCGTACTGACGTATCAAATTGATCAGCAAATATTTTTGTTGGAATGAAACCATTTG

TTTCATATTCTTCCACCATGGCACGTCGCTGAGTCGGGTTTATAAGCGGTGGCCTTCCACTTCTTACACGACACTGCAGA

CTACCTTCTTCCTCGAACCTCTTTATCCAACGGCTCACAGTATTTCTCTTCCTCTGGGGTGAGATCCGCAAACTTGATAC

CACCACTTCCATCCTAGA

>TRINITY_DN14440_c0_g1_i2_Tc1-1_Xt_tp#DNA/TcMar-Tc1394-1416[Xenopussubgenus]

ATAAAATAATATTTGGTAATGTGAAGAAACATTTATTTATATTATTGTTAATAAAGTTAAAAACATAATTTATCATTTTG

TATTATATTAAAATTAAAATAGAAGTGTTATTATACTTCTGACTTTATGAGATTATAGCTTAATATATTCATTTTCTCAA

AGAGAAAAGAAGATTGAAGCTTAATCCCAATCTTAAAATCAAAATAAATTACGTATGACTTTATATTATGACCAGCACGC

CCACTGTAAAAAACAGCGTAATCAATATTTAGTATGGCCTCCATTATTTTCAATGCAGCTAAGCAGACGTCGCCGCATGG

AGGCCACAATGTTTGCACATATATCTGTGCCGCGTAAATTCTCCCACAGTTGAGTGCAATGGGATTTAAGCTGCTCAGGA

CATCTCTCATCTCGCGCTTCCCATCTTTGAACCATTAAACCCCATATATTTTCAATGGGGTTTAGATCAGGGGAACGTGC

TGGCCACGGAATAACTTTAATATCATTATGCTTGCTAAACCACTCTTTTACAATACTGGCACGATGTATCGGACAATTAT

CTTGAACAAAATTTAGTTCACTACATTCTTCTATGGGGTAAACATTACGCACAGTAGGTAGCATTATGTCATTTAAAACT

TCAACGTAATTAGAGCTGTTCGCACGCGTAGGTATGAATTCCAGTTCACCCGGCCCTTGAGCACTAATCCACCCCCACAT

GTTCACACTTATTCTACCGGATTCTGTATTCGGAATAATGTTTTGCTCAGACCAGCGAGTATTATCTTTTCGCCATAAAT

GAAGTCTTCCTTTCTGGCAAGATTTGAAAGTCTTTTCATCGGTAAATATGGTTGTATCCCAGTTAAAATCTAAATATTCT

CGAGCAAAACGTAGTCGTTCCTCCTTGTGTTTTTGTGTCAAAAACGGTTTTTTAGCTGGTTTCCGGTGATGTAAACCTTC

AGCATGGAGTGCACGGCGAACAGTTCGTACTGACGTATCAAATTGATCAGCAAATATTTTTGTTGGAATGAAACCATTTG

TTTCATATTCTTCCACCATGGCACGTCGCTGAGTCGGGTTTATAAGCGGTGGCCTTCCACTTCTTACACGACACTGCAGA

CTACCTTCTTCCTCGAACCTCTTTATCCAACGGCTAACAGTATTTCTACATAAGCCGGTTTGATTTGAAATTTGAGAAAT

ATTCAACCCATTTTGATGTAAAGCGACAATATTTGCCCTGGCATCAATCGTAATATTTGCTTGCATTTTGAATAAAGATA

ATAAAAATACACTAATCGCAATGATAATACAAACGCTCCGAACAAAGTTCACCAACAAACAACGTCGTAACTAATTTATT

AAATTAAAATCTAGTAGTTAGTTAATTATTTTTTTTACATTTACCCTCATCTTCATTCTTTTTTGTAAATAAGTTCGCGA

CACATTTCATCGCATATAACTAACTATAGAATTAGTGTAGCCATTCTTAATTCATGATTAAAACACAAAAAGTGTATACT

GCAGTGGGGTTCGATTGCAGGTCTCAATGAGCGTTTCAGTTAGACCAATGCGTTATCCGCTAAGCCAAACAGACACTTAT

CTACATTATTGAAATTAAATAACAATATCTCAATACGTCCGTCATCGCATAACTGGTTTGTCACCAAGGCAAATTATTAT

ATCGCACTCTTTCTGACTAGATAATGATGAAAGAGTGCGAGACCGCGTATGTGTGCAAAATGTTTGTTATAGAAAAAGGT

CGTAAGTGCAACTTGTTTTAATGCGATAGTTTTGTGAAACTGCTACTGCACTTATGACCGCTACCGTACACAAATGTTGG

GTCACATCACTGTAATCACTCGCCGGGGATGACGTAACTGCTCGACAATCATTTA

>TRINITY_DN14440_c0_g1_i3_Tc1_XL_tp#DNA/TcMar-Tc1199-1185(missesNterm)[Xenopussubgenus]

ATAAAATAATATTTGGTAATGTGAAGAAACATTTATTTATATTATTGTTAATAAAGTTAAAAACATAATTTATCATTTTG

TATTATATTAAAATTAAAATAGAAGTGTTATTATACTTCTGACTTTATGAGATTATAGCTTAATATATTCATTTTCTCAA

AGAGAAAAGAAGATTGAAGCTTAATCCCAATCTTAAAATCAAAATAAATTACGTATGACTTTATATTATGACCAGCACGC

CCACTGTAAAAAACAGCGTAATCAATATTTAGTATGGCCTCCATTATTTTCAATGCAGCTAAGCAGACGTCGCCGCATGG

AGGCCACAATGTTTGCACATATATCTGTGCCGCGTAAATTCTCCCACAGTTGAGTGCAATGGGATTTAAGCTGCTCAGGA

CATCTCTCATCTCGCGCTTCCCATCTTTGAACCATTAAACCCCATATATTTTCAATGGGGTTTAGATCAGGGGAACGTGC

TGGCCACGGAATAACTTTAATATCATTATGCTTGCTAAACCACTCTTTTACAATACTGGCACGATGTATCGGACAATTAT

CTTGAACAAAATTTAGTTCACTACATTCTTCTATGGGGTAAACATTACGCACAGTAGGTAGCATTATGTCATTTAAAACT

TCAACGTAATTAGAGCTGTTCGCACGCGTAGGTATGAATTCCAGTTCACCCGGCCCTTGAGCACTAATCCACCCCCACAT

GTTCACACTTATTCTACCGGATTCTGTATTCGGAATAATGTTTTGCTCAGACCAGCGAGTATTATCTTTTCGCCATAAAT

GAAGTCTTCCTTTCTGGCAAGATTTGAAAGTCTTTTCATCGGTAAATATGGTTGTATCCCAGTTAAAATCTAAATATTCT

CGAGCAAAACGTAGTCGTTCCTCCTTGTGTTTTTGTGTCAAAAACGGTTTTTTAGCTGGTTTCCGGTGATGTAAACCTTC

AGCATGGAGTGCACGGCGAACAGTTCGTACTGACGTATCAAATTGATCAGCAAATATTTTTGTTGGAATGAAACCATTTG

TTTCATATTCTTCCACCATGGCACGTCGCTGAGTCGGGTTTATAAGCGGTGGCCTTCCACTTCTTACACGACACTGCAGA

CTACCTTCTTCCTCGAACCTCTTTATCCAACGGCTAACAGTATTTCTCTGAAAAACGAAAATTTTATTATAAACTTGCAA

CTAGTGATTATATAAAAAAATATAGACCACAACAACCATAATTACAATTTTAAAAAAATACTTACACATAAGCCGGTTTG

ATTTGAAATTTGAGAAATATTCAACCCATTTTGATGTAAAGCGACAATATTTGCCCTGGCATCAATCGTAATATTTGCTT

GCATTTTGAATAAAGATAATAAAAATACACTAATCGCAATGATAATACAAACGCTCCGAACAAAGTTCACCAACAAACAA

CGTCGTAACTAATTTATTAAATTAAAATCTAGTAGTTAGTTAATTATTTTTTTTACATTTACCCTCATCTTCATTCTTTT

TTGTAAATAAGTTCGCGACACATTTCATCGCATATAACTAACTATAGAATTAGTGTAGCCATTCTTAATTCATGATTAAA

ACACAAAAAGTGTATACTGCAGTGGGGTTCGATTGCAGGTCTCAATGAGCGTTTCAGTTAGACCAATGCGTTATCCGCTA

AGCCAAACAGACACTTATCTACATTATTGAAATTAAATAACAATATCTCAATACGTCCGTCATCGCATAACTGGTTTGTC

ACCAAGGCAAATTATTATATCGCACTCTTTCTGACTAGATAATGATGAAAGAGTGCGAGACCGCGTATGTGTGCAAAATG

TTTGTTATAGAAAAAGGTCGTAAGTGCAACTTGTTTTAATGCGATAGTTTTGTGAAACTGCTACTGCACTTATGACCGCT

ACCGTACACAAATGTTGGGTCACATCACTGTAATCACTCGCCGGGGATGACGTAACTGCTCGACAATCATTTA

>TRINITY_DN14418_c0_g1_i1_Mariner-10_BM#DNA/TcMar-Tc1420-1484[Bombyxmori]

GGAATCAACAGTGTCTGCAACCTACAATCAGACTAACATTTTCACCACAAAATATACAACGTGTCCCAAAATTCAACGAT

AAGCCGGCGCCAAAAGGTAGACCTGCTCATGAGTACATAAGGAAAAATAATAAAAAAAATATAGCTAGTTTTTTTTTTTA

GTTACAAGGAAAATTTAGAATTCCTTTGAAATTTTACACCCATCATGATATTTTGAACGACCACGATGACAATTTCTCAA

ATATGCTTAAGATTTTTTTTTTATCTTATATCTAAATAGTGCTATTATACACCACAACTCTTAAAAACACCAAATTGCCC

GCAGTTTTTACACAAAAAACGTTCAAAATCATTTTTTTCCCTAAAATTATGAAATATTTTTACTCTTAGTGTACTTTGAC

TCATGGTCTTGTAAAAAAAAAGTATGGACACCACTATGGCAAGTTGTTTTTAAAATTGGCGCAACTAATCAAGATTCTAA

AAAGGTATAATACTCCAAAATAACTGGCCACAAAAAAAAAATATGTCTACCATTTGTAAACAATACCCAAATTCATTAAT

TGTTCATGTTGTTAGTAATAAATATTATAATGTTCCAAAAATGAATAAAACAATGGAAACTATTTTTGTAATTAATTTAT

TAAATAATTAACATTACAGTAAATGTTCAAACTGTTTTCCTCCGGCGTTAATACAGGCACGACATCGCCTTAAAAAGGAC

CTTTTTATCTTTCTTGCATATCTTCTTGCATTGATATGTGCCGCAGTCTGAGTTATTTCATGCCGAAGCTCATCTAATGT

CGCAATCGGTTTCGCGTAGACCCTGTCTTTGAGACAACCCCAATAAAAAAAGTCCAGGGGGTTTAGGTCGGGTGATCGGG

GTGGCCATAAGATAGGACCAAGTCGCCCGATCCAACGCCCCGGGAACTCTTGGTCTAGGTATTGTCTCACAGGACTAGCG

TAATGAGCTGGGCGACCATCATTTTGAAACCACATATTTTGAAGGTCACTTAAGGGGACGTCTTCTAGTAATTCTGGCAA

ATTATTTTGTAGAAAGTTCAAATAACTGTCCCCATCCAAGTTTCCTTGTAATTCAAAAGGCCCAATCACTTTTCCATTTA

ATATTCCCGTCCATAAATTAACCTTAAATTGATATTGGGATTTCTCTTGTCTCATCACATGTGGATTCTCATTGCTCCAG

CTGTGTAGGTTGTGAAGATTTAAATAACCATCTTTCTTGCAGGTTGATTCATCCGACCATAATATTTTTCCCAGGAACTG

AGGGTCATCCCGGTGCTTTTGAAGCATCACACGACAAAATGCGATTCGCAGTGGATAGTCCCGAGGTAGCAA

>TRINITY_DN14418_c0_g1_i2_Mariner-10_BM#DNA/TcMar-Tc1420-1484[Bombyxmori]

AAAAATGGAGGTACGTTTGGGCATTGACGAGTATTCGTGATATTATACAACGTGTCCCAAAATTCAACGATAAGCCGGCG

CCAAAAGGTAGACCTGCTCATGAGTACATAAGGAAAAATAATAAAAAAAATATAGCTAGTTTTTTTTTTTAGTTACAAGG

AAAATTTAGAATTCCTTTGAAATTTTACACCCATCATGATATTTTGAACGACCACGATGACAATTTCTCAAATATGCTTA

AGATTTTTTTTTTATCTTATATCTAAATAGTGCTATTATACACCACAACTCTTAAAAACACCAAATTGCCCGCAGTTTTT

ACACAAAAAACGTTCAAAATCATTTTTTTCCCTAAAATTATGAAATATTTTTACTCTTAGTGTACTTTGACTCATGGTCT

TGTAAAAAAAAAGTATGGACACCACTATGGCAAGTTGTTTTTAAAATTGGCGCAACTAATCAAGATTCTAAAAAGGTATA

ATACTCCAAAATAACTGGCCACAAAAAAAAAATATGTCTACCATTTGTAAACAATACCCAAATTCATTAATTGTTCATGT

TGTTAGTAATAAATATTATAATGTTCCAAAAATGAATAAAACAATGGAAACTATTTTTGTAATTAATTTATTAAATAATT

AACATTACAGTAAATGTTCAAACTGTTTTCCTCCGGCGTTAATACAGGCACGACATCGCCTTAAAAAGGACCTTTTTATC

TTTCTTGCATATCTTCTTGCATTGATATGTGCCGCAGTCTGAGTTATTTCATGCCGAAGCTCATCTAATGTCGCAATCGG

TTTCGCGTAGACCCTGTCTTTGAGACAACCCCAATAAAAAAAGTCCAGGGGGTTTAGGTCGGGTGATCGGGGTGGCCATA

AGATAGGACCAAGTCGCCCGATCCAACGCCCCGGGAACTCTTGGTCTAGGTATTGTCTCACAGGACTAGCGTAATGAGCT

GGGCGACCATCATTTTGAAACCACATATTTTGAAGGTCACTTAAGGGGACGTCTTCTAGTAATTCTGGCAAATTATTTTG

TAGAAAGTTCAAATAACTGTCCCCATCCAAGTTTCCTTGTAATTCAAAAGGCCCAATCACTTTTCCATTTAATATTCCCG

TCCATAAATTAACCTTAAATTGATATTGGGATTTCTCTTGTCTCATCACATGTGGATTCTCATTGCTCCAGCTGTGTAGG

TTGTGAAGATTTAAATAACCATCTTTCTTGCAGGTTGATTCATCCGACCATAATATTTTTCCCAGGAACTGAGGGTCTTC

CCGGTGCTTTTGAAGCATCACACGACAAAATGCGATTCGCAGTGGATAGTCCCGAGGTAGCAA

>TRINITY_DN14418_c0_g2_i1_MAG_pol#LTR/Gypsy1045-4368[Bombyxmori]

CCCCTCTATACAATTTGCATTATTGATTGTGGCTATGGGGGGTACACTAATTTTTAATTCTGACAACCACTGTCTTCCTA

AAAGTGTAGCGCTCCCCGTTTTAAACACATATAAATCTAATTTCTTTTTTTGGCTTTTGTAAGTAACAGTTACTTCTAGA

TACCCCTCGGGTTGAACTTTACTACCATTATAAAATTTCACCGCCAGATTACATGGCAATATTGGTACATCAGAAAAACA

TTCATTATAAGTTCTACTAGAGATACATGATAAGGGAGACCCGGTATCAATTTCCATTGTTAATAACACATTGTTCACTA

AAATACTCATGCTCACCGGCCTATATTGACTTAACGACATTTGATAAATTGGTTCTTCTTCCAAAATAACTTCTTCCGCT

TGTTCCTCCCCTTGCGTCTCGAAAACGTGCATTGTCGGTTGATAGGCTCCCGATCGCGCGTGCCAGGACCGCCCCGTGCC

CCGGCTAGCGCCACGGTAACCCCCAACACTCACTCTGGCGCCCCGCCGCGTCATGCCTGCCCCGCCGCCATCTTTCCGTG

CTTGAGCGCGGTCTCGTTGTTTTACGCTATCGCCATTTTCCTGACACATTCGACGCAAATGTCCCACTGTTTTACATTTA

CTACACTGGTACTTTGAAAATCGGCATTCCATTTTCGTATGTCTGTAGTCCCCGCATGCCGTACAACCCCCGAGTTGCAT

GCGATGTAAATCTCCCCTTAAATCCACTATCTCCGAACTACGCCGCGATACTGTGTTTCTAGAAACGTCAGGCCCCGCCG

CAGCCGCGAAGGTGCTCGCCCCGACGCTGCCGCGCCCATCCACAGCAGCTGCATCCCGCTCCGCCGCTTCCAGCGATGTC

GCCACCCTAACCGCATCCGTATAAGTGACTGAGTCGGCTTCCGCGAATAACCGTTGACGAATGACCTCGCTTCGTAACCC

GCACACGAATTGGTCCCGTAAATTTTCGTTTAAATTGTCTTTGAATTTACAATATCTTGCGAGTTTCTTGAGCTCGGCTA

CGTACATGGCCACGTTTTCCTCGTTGGCCTGCCTTCGTTGTCGAAACCGATACCGTTCCGCTAGCACTGATGGCGTTGGC

TCCAAATGTTGATGCAATAACTCCACCACTTTGAGATACGTCAAATCCGCCGGTTTTTTAGGACTAGCCAGGTCACTTAA

TAGTTCATACGCCTCATCTCCCATTACTGCTATTAATGTTGGTAATTTTGTGGCATCGTCAATATTATTTACTTTAAAGT

ACATGTCCAACCGTTCCACATATACTGACCACGTGCCGGACTTCACGTCAAATTCTTTTATTTTACCGATCGACATCTTG

ATCACTTTTTTGTTTTTTCCTTTCTTATTCGCACTCCTCCGGTCACGCTGACACGCGTTTTTTTATTTTCCTTGTCCAAT

TTATGCCATTCTCGTCGCCACTGTGGTATTTCGGAATAAGACACGTCGTTCGGTGGGTTAGCCATTTATTCTCTGCAAGT

CGACAGGTGGGTTCACAGATAATAAAACGCATACAGATAACCTATATTACACCTCCACCCTTATTTACTAACTTAAAATT

ATCATACATCCCTAGTACTTATGTTTTATTTCCTTACTGACTACTGCCTTAATTATTCGTTTACAAACTTAAAACTAGTA

AGCCCTTAATCTACGAACCCATTACAGCTGATATCTTTGGGGTAGTCTCCTATTCCTAATGGGACGCGTTTCACTGACGG

CCGACGCCACACTGTTGGACTCTGACGATTCCGTTACCAACGTCGGTTCCATACGGGTCTCAACACTCTTCATCCTACCC

TTTCCTGAACCTTCGACACCCCCACCCGAGCACCCCTCGACGGGTGTGATCGCCACTGGTATTGTCGGAAAAACCAAGGC

ATTACCTACCGACTGGTTAGTGTTCTTGGGCGTTACGTTCGTGCTGACCATTTCGTTATCTGTCGGTAACGACGGTGTCG

GTCGTAATGGATTTTCTTTACCTTTTAGCTGATCAATATGCCTATGTATCTCTGTGCCATCTAATAACCTTATTCTATAA

TCTGTAGTACTCAGACGCTCAATTACCGTACCACTTGCCCATTTCCTTAATCCTTGGTACACCCGGCACCAAACCCCGTC

CTGGGGCGCAAACTCGCGCTGCGTGCCGCCTGCACCGTGTTGCTGGCGGCGCTGCGCGTCCCTCACTTGCTTGTCGCGCG

CCGGTTTCAACCGGTCTAACCGAGTTCTTAATGTCCTGCCTTGTAATATCTGTGACGGACTTTCTTTCGTTGTGCTATGT

TCAGTGGTTCTATACACTAGTAAAAACCGTTGAAGGGCTAGTTCTACATCTATTTTTTGGATAATAGCTTTTTTTATCAC

TCTTTTGCACTGTTTCACCGCATTTTCCGCCAGACCATTGGAAGCTGGATGATATGGTGCGGAATATACATGTTCTACTC

CGTTATATTCTAAAAATGAATTAAATTCTTCGCTACTAAAAGGAGGTCCGTTATCCGAGACTAATTGTTTTGGTATACCA

AAACGTGACCAAATTTCTCTAATTTTACCTATTATTGCTTTGGCCGTAGTCGAAACGACCGAACATGCTTCAATCCACTT

TGAGGTTGCATCGACCATTATTAAATACTTGTGTCCCTGTATAGGCCCTAAGAAATCGACATGTAATCTGGTCCATGGAC

GAGAAGGCCAGTCCCACGGCTGCGGTGTGTGGTGTACCGGCGCTTCGCCCATTTCTGCGCAGATTGTGCAATTTCGACAT

TGACTTTCTAACATTTCATCAATCCCCGGCCACCATACATAGCTTCTAGCTATACATTTGGTCTTAATTATACCCATATG

CGTGTCATGTAGCTCCCTCAAGACCCTGTCCTGACACGCTTTTGGCACTATTAACCTATGTCCCCCCATAAGACAACCTA

ACTCTGTATATATTTCATTACGTCTATTCGAATAAGGTTTAAGTTCCCTCATCTCCACTTCCTTAGGCCAACCATCCCTA

ACATAGCTCAATACACGCATTAATATTGGGTCTTTTTTTGTCTCTTTTCGAATATCTTCATGTTTAAGTAACAAAGCATC

CGACGCAAAATGCAGGTACGTTTGTTCCGGAACTTCCTCTCTGTCTGTTTCACTAATAGCTTTATAACTTTCTACTAATC

GAGACAAAGCGTCAGCAGTGTTTGAATGTGTATTCACGTATTCAATTTTGAAATCATAAGCTGATAATATTAACGCCCAT

CTCTGCAACCTGCTAGCTGTCATGCTCGGAATTCCTAACTCTGCTCCAAATATACTAACTAAGGGTTTATGATCTGTTCT

TAAAACAAATGGTCTGCCATACAAATATTGGTGAAATTTCTTCACTGCGAAGACTATAGCTAAAGCCTCCTTATGTATTT

GGCTATAATGATATTCCGCGGCGGTAAGTGTTCTTGACGCGTACGCTATGGGACACTCAAGGCCCTCGGGCGTGCTCTGC

GCCAGCACCGCGCCGACGCCGCGCGAGCTCGCGTCGCACGTCACTACCACGGGCCGTGACTCATCATAGTGCGATAATAT

TTCTGAACTAATTAATAATTGTTTTATTTTCTTAAATGCTAGCGAATGAGTAGTGCTCCATGCCCAATGTTTACCCTTTT

TCAATAGTTCGTATAATGGTGATAAATAGAAACTTAAATTTCTTATGAATTTTCCATAAAAGTTCACCATACCTAAAAAC

GATTTTAGTTCCGATACGTTAGT

>TRINITY_DN14413_c3_g3_i10_Harbinger-1_DW_tp#DNA/PIF-Harbinger964-2779viagenewisewithHarb_Cis1_tp,1intron(1248:1902)[Drosophilawillistoni]

GCTGTAAGAAAGGACAATTCGTCAACATATACGCCGATCTAAGAAGATATCCGGATAAGTTTTTTAATTATGTGAGAATG

TCCGTGAGGTCCTTTGATGAGTTACTGACTTTGTGTGAAAATGATTTGTTAAAGCAAGACACAATATTAAGAAAATCAAT

CAGTCCTGAAGAAAAGTTGTTTGTAACATTAAGGTAAGTTAAATCAAACTATGAACATTTTTAAAGTAAGTTTTATTAAA

AAATGTAGACAAACAAACAAGCATGAACGTTTAAGTGCTATATAGATCCATAAGTGGTTCTGAGTCCTGTGAATCATTTG

TGTCGCTTGGAGAACTTGCATATGTCATTGTATTCATAGAATTATATGGGGAATTAGGACTACTTGTGATAGGTTCATTC

TGATAATTCGTGTTTGTCCTATAACTTGGTCCAGGATATTCAGTAGCAATCACTTGCCGTTGCGTCGAATAACCAGGATG

GTAGGTGTCATGACTCATCATCCTGCCACCTGGATTAATTTCTGGAGAATTCCGAAAAGACGGACCAGTATAATCAAACG

TCCTGTTACCAGAAAAACCTGACGCATCGTTCAACAAAGTTAGCATCTTTATTTTGGTAGCCATTTTAATTTGAGGAGGT

AGATTTTTTAATGTGCTCAACAATGAAAGCAAAAATAGCCGATCTTCGTCAACCTCAGCTGTCTCGCGTAGTCTCTGCTC

TGTTGTCTCACGGCGTCTTTGCTCAATTGAATTATTTAATGCTTGAATAAAAGGATCATCGTCTACTGCTAGTTTACGTT

TGTTTGTAGCTTTTTTTGGTCGCTCGCCTACTTCAGTGCTCTCCGAATTTTTTTCTTCTTCACCTTCTTCTCTGATAGCT

ACTACTTTTTGTAGAAACTGTAACTGCTTAAAATATATATATTCGCTTTTGCGGTTAGTGGCTCCAGACCCGGACTTAAC

ATTTCTTTGACGTTTGACTTCCCGCGCATAGCATGATCTGATGCTTTTCCACCTTTTTTGCAACACTAACCCTGGAAATA

AAATTATAACACTTATTACTTTTTGTTAATTACAGGTACCTCGCAAGTGGATGTACGATGAGAGAATTGCATTATAGCTT

TCGTCTAGGACAAACAACATTAAGCACAATAATAAAAGAAGTGTGTGCTGTGATTTGGGATAAGTTGCAAACATGTTTAT

CACTTCCGGCTACTGCAAATGACTGGATGAAAATAGCAGATGGTTTCGAAAAACATGCCAACTTTCCACACTGCATCGGT

AGTATCGATGGAAAGCACATAAGACTGATTCAACCTGCTGATTCTGGTTCGATGTATTATAACTATAAACACTTTTTTTC

ATTGGTGCTGATGGCTGCATGTGACGCTAATTATAATTTCATTTATATAGACGTCGGTGCCTATGGGAAAAGTAGCGATT

CAGCAATTTTCCAAGAAACTGAATTGTATAAGAAACTCATAAGTAATACTTTAAATATTCCAGAGCCCCTACAAATTTCA

GAAAATAACCCAACGCTTTTCCCTTACGTATTTATAGGCGATGAAGCATTCGGGTTGAGCACAAATATTATGCGTCCATA

CGGAGGAAACAACCTCAGTGCCGAAAAGAAAATTTTTAACTACAGACTATCGAGAGCTCGCCGTTACATCGAGTGCACAT

TTGGCATCTTAACAAATAAATGGAGAATTTTCCACAGACCATTAAATGTCCACACAGAATTAGCAAAATCCATTGTGAGA

ACATGTTGTGTGTTACACAATTTTGTACGTTCTAGAGATGGATATTCATATGATGATACTCTGACAATAATGGGCTTTCA

AACAACAACACAGCAAAATTTCAATAGAGGAGGGCGTGCTGCTACTACCACTCGAGATAATTTTACACATTATTTTGTAA

ACGAAAACCCACTTTCATGGCAAAATAATTATATTCATTAATAAATACATACACAACTTACAACGAGCTTTTGGCTTTCC

ATGTTACACCAGAAGCATCATGAACATTTATTGATTGACAAATTCATTCAGAAAAATAAATGTTGTTTACTTACCAAAGC

TCTTTTTTTGTTCAACGGTTTGCTCCTGTTCTCCAAATAAATCTACAACTTCCTCCCAACATTTCTTTTTCAAATCTCTA

TCGGAATAATCTGACGAGGAATTATCCCAAAGTGATGGTCTACTTTGAATTTCGATAATAAACCTTTCTGTATCGAAATT

ATCCATCGTATGCAGTTAACAAACTACTCAGATATTAAAATAAGCAGTTAAAAAATAAAACATAACCGCGACACGTGCTG

CATGCAATGAGGTGGGAGCGTCACTGGCGGCCGGACTGGCGGCCGAGTGAGTGAGACACCATATCGCGCTATAGTAGTTA

GTGGTGGCGCGACCAGCGTGGCGGCGCAACCAGTAACATTCTATGCACGCGTCCGAGTTGGTCGCGCCGCCAAATTGGTT

GCGCAACTAAACGGACACCAGGTGAGTAACTCTCTATAGAGCTGTATACATTTTGTTGGTAGCGGCGACTGGTTGCGCCG

CCATGGTGTCCCGGTGAAATATAGCCCTTACTGGCCGGCTGCTCGACGATCTCTACGTAGGGCTGGTCGCGGCGCGCCAT

GCTATCGTCTATGCCGGCGCCTGCGCCCGCGCCGAACGTCGGGTCCGCCAATGTGATCGCCTCAAAAACATCACTAATAT

TCAGTTGTTCCTCATTCTCGGCAGTGCCTATAGTTATGGCGCTTTCTCCGACGTTGTCCATGGCACTGTACGAATAGGCC

GCGGGCCGCGGACACCTCACATCGGTCCTTCCGCACGCGACAATATCAAAACGCACATTTATATAAACTTCGTCGTAACT

CGTTTTGGTTTAAAACTTAACACTTGCCACTTTTATAGTGTACTTTGATAACTTTTATATGCACTATAGCTCACTTTTTA

ACTTAATTCTGAGAGACTGATTTAATAACACACACGATATACTTCACAAATTGTAAACTATAGACGTACCTCCAGCATGA

ATGATCCTATAATAATAACTGTGAACTGTTTCTTCGCTCTCGCTCGTCCAAGGAACTAGTTATCAAGGTCTCTCGTCTCG

ACGTAGTGTATTTTTCAAGTTCCAAGAATTGTCACAGATAATGTCTCGAACTCTGACACTATATTGCAAACGCGCGGCAC

AAAATTTGATAAATCCGGCTAATTAAATAAGCAAAGAATATTCAGGTAACAAACGTATAGTCGCGAAGTAACACTACTCC

GGCTAGTGAACGTCAAAAACTCTAATGATAAATCTCAATAGTGTGCATGAGCACCCGACATCACACAAGCAACAACTGCC

GGCGATTGGCGGCGCTAAGTGTCACATGGCGGCTGAGTGACGTACGCGGAATTGCGAACATAGCGCATGCGTACAGTGTA

TTCAAAACTTTATTCCATAAGTGCGAGTAGAAACACAATAGCGGTAGTACTGTTCTAGCCGTCTATAACCGTACCGGTGC

TTTGCTACGGTTTCCCGCCAAAATTAAACAACATTTTTCTTTAGAATTTAATATGTATATTTAGTTATTGTGTTTTTAGT

TGTTGACAGATGTTAAATGTGATTTATTAAACAAATATCATTGGTTTTAAAAAAAA

>TRINITY_DN14413_c3_g3_i12_Harbinger-1_DW_tp#DNA/PIF-Harbinger964-2779viagenewisewithHarb_Cis1_tp,1intron(1248:1902)[Drosophilawillistoni]

GCTGTAAGAAAGGACAATTCGTCAACATATACGCCGATCTAAGAAGATATCCGGATAAGTTTTTTAATTATGTGAGAATG

TCCGTGAGGTCCTTTGATGAGTTACTGACTTTGTGTGAAAATGATTTGTTAAAGCAAGACACAATATTAAGAAAATCAAT

CAGTCCTGAAGAAAAGTTGTTTGTAACATTAAGGTAAGTTAAATCAAACTATGAACATTTTTAAAGTAAGTTTTATTAAA

AAATGTAGACAAACAAACAAGCATGAACGTTTAAGTGCTATATAGATCCATAAGTGGTTCTGAGTCCTGTGAATCATTTG

TGTCGCTTGGAGAACTTGCATATGTCATTGTATTCATAGAATTATATGGGGAATTAGGACTACTTGTGATAGGTTCATTC

TGATAATTCGTGTTTGTCCTATAACTTGGTCCAGGATATTCAGTAGCAATCACTTGCCGTTGCGTCGAATAACCAGGATG

GTAGGTGTCATGACTCATCATCCTGCCACCTGGATTAATTTCTGGAGAATTCCGAAAAGACGGACCAGTATAATCAAACG

TCCTGTTACCAGAAAAACCTGACGCATCGTTCAACAAAGTTAGCATCTTTATTTTGGTAGCCATTTTAATTTGAGGAGGT

AGATTTTTTAATGTGCTCAACAATGAAAGCAAAAATAGCCGATCTTCGTCAACCTCAGCTGTCTCGCGTAGTCTCTGCTC

TGTTGTCTCACGGCGTCTTTGCTCAATTGAATTATTTAATGCTTGAATAAAAGGATCATCGTCTACTGCTAGTTTACGTT

TGTTTGTAGCTTTTTTTGGTCGCTCGCCTACTTCAGTGCTCTCCGAATTTTTTTCTTCTTCACCTTCTTCTCTGATAGCT

ACTACTTTTTGTAGAAACTGTAACTGCTTAAAATATATATATTCGCTTTTGCGGTTAGTGGCTCCAGACCCGGACTTAAC

ATTTCTTTGACGTTTGACTTCCCGCGCATAGCATGATCTGATGCTTTTCCACCTTTTTTGCAACACTAACCCTGGAAATA

AAATTATAACACTTATTACTTTTTGTTAATTACAGGTACCTCGCAAGTGGATGTACGATGAGAGAATTGCATTATAGCTT

TCGTCTAGGACAAACAACATTAAGCACAATAATAAAAGAAGTGTGTGCTGTGATTTGGGATAAGTTGCAAACATGTTTAT

CACTTCCGGCTACTGCAAATGACTGGATGAAAATAGCAGATGGTTTCGAAAAACATGCCAACTTTCCACACTGCATCGGT

AGTATCGATGGAAAGCACATAAGACTGATTCAACCTGCTGATTCTGGTTCGATGTATTATAACTATAAACACTTTTTTTC

ATTGGTGCTGATGGCTGCATGTGACGCTAATTATAATTTCATTTATATAGACGTCGGTGCCTATGGGAAAAGTAGCGATT

CAGCAATTTTCCAAGAAACTGAATTGTATAAGAAACTCATAAGTAATACTTTAAATATTCCAGAGCCCCTACAAATTTCA

GAAAATAACCCAACGCTTTTCCCTTACGTATTTATAGGCGATGAAGCATTCGGGTTGAGCACAAATATTATGCGTCCATA

CGGAGGAAACAACCTCAGTGCCGAAAAGAAAATTTTTAACTACAGACTATCGAGAGCTCGCCGTTACATCGAGTGCACAT

TTGGCATCTTAACAAATAAATGGAGAATTTTCCACAGACCATTAAATGTCCACACAGAATTAGCAAAATCCATTGTGAGA

ACATGTTGTGTGTTACACAATTTTGTACGTTCTAGAGATGGATATTCATATGATGATACTCTGACAATAATGGGCTTTCA

AACAACAACACAGCAAAATTTCAATAGAGGAGGGCGTGCTGCTACTACCACTCGAGATAATTTTACACATTATTTTGTAA

ACGAAAACCCACTTTCATGGCAAAATAATTATATTCATTAATAAATACATACACAACTTACAACGAGCTTTTGGCTTTCC

ATGTTACACCAGAAGCATCATGAACATTTATTGATTGACAAATTCATTCAGAAAAATAAATGTTGTTTACTTACCAAAGC

TCTTTTTTTGTTCAACGGTTTGCTCCTGTTCTCCAAATAAATCTACAACTTCCTCCCAACATTTCTTTTTCAAATCTCTA

TCGGAATAATCTGACGAGGAATTATCCCAAAGTGATGGTCTACTTTGAATTTCGATAATAAACCTTTCTGTATCGAAATT

ATCCATCGTATGCAGTTAACAAACTACTCAGATATTAAAATAAGCAGTTAAAAAATAAAACATAACCGCGACACGTGCTG

CATGCAATGAGGTGGGAGCGTCACTGGCGGCCGGACTGGCGGCCGAGTGAGTGAGACACCATATCGCGCTATAGTAGTTA

GTGGTGGCGCGACCAGCGTGGCGGCGCAACCAGTAACATTCTATGCACGCGTCCGAGTTGGTCGCGCCGCCAAATTGGTT

GCGCAACTAAACGGACACCAGGTGAGTAACTCTCTATAGAGCTGTATACATTTTGTTGGTAGCGGCGACTGGTTGCGCCG

CCATGGTGTCCCGGTGAAATATAGCCCTTACTGGCCGGCTGCTCGACGATCTCTACGTAGGGCTGGTCGCGGCGCGCCAT

GCTATCGTCTATGCCGGCGCCTGCGCCCGCGCCGAACGTCGGGTCCGCCAATGTGATCGCCTCAAAAACATCACTAATAT

TCAGTTGTTCCTCATTCTCGGCAGTGCCTATAGTTATGGCGCTTTCTCCAGCATGAATGATCCTATAATAATAACTGTGA

ACTGTTTCTTCGCTCTCGCTCGTCCAAGGAACTAGTTATCAAGGTCTCTCGTCTCGACGTAGTGTATTTTTCAAGTTCCA

AGAATTGTCACAGATAATGTCTCGAACTCTGACACTATATTGCAAACGCGCGGCACAAAATTTGATAAATCCGGCTAATT

AAATAAGCAAAGAATATTCAGGTAACAAACGTATAGTCGCGAAGTAACACTACTCCGGCTAGTGAACGTCAAAAACTCTA

ATGATAAATCTCAATAGTGTGCATGAGCACCCGACATCACACAAGCAACAACTGCCGGCGATTGGCGGCGCTAAGTGTCA

CATGGCGGCTGAGTGACGTACGCGGAATTGCGAACATAGCGCATGCGTACAGTGTATTCAAAACTTTATTCCATAAGTGC

GAGTAGAAACACAATAGCGGTAGTACTGTTCTAGCCGTCTATAACCGTACCGGTGCTTTGCTACGGTTTCCCGCCAAAAT

TAAACAACATTTTTCTTTAGAATTTAATATGTATATTTAGTTATTGTGTTTTTAGTTGTTGACAGATGTTAAATGTGATT

TATTAAACAAATATCATTGGTTTTAAAAAAAA

>TRINITY_DN14413_c3_g3_i14_Harbinger-1_DW_tp#DNA/PIF-Harbinger964-2779viagenewisewithHarb_Cis1_tp,1intron(1248:1902)[Drosophilawillistoni]

GCTGTAAGAAAGGACAATTCGTCAACATATACGCCGATCTAAGAAGATATCCGGATAAGTTTTTTAATTATGTGAGAATG

TCCGTGAGGTCCTTTGATGAGTTACTGACTTTGTGTGAAAATGATTTGTTAAAGCAAGACACAATATTAAGAAAATCAAT

CAGTCCTGAAGAAAAGTTGTTTGTAACATTAAGGTACCTCGCAAGTGGATGTACGATGAGAGAATTGCATTATAGCTTTC

GTCTAGGACAAACAACATTAAGCACAATAATAAAAGAAGTGTGTGCTGTGATTTGGGATAAGTTGCAAACATGTTTATCA

CTTCCGGCTACTGCAAATGACTGGATGAAAATAGCAGATGGTTTCGAAAAACATGCCAACTTTCCACACTGCATCGGTAG

TATCGATGGAAAGCACATAAGACTGATTCAACCTGCTGATTCTGGTTCGATGTATTATAACTATAAACACTTTTTTTCAT

TGGTGCTGATGGCTGCATGTGACGCTAATTATAATTTCATTTATATAGACGTCGGTGCCTATGGGAAAAGTAGCGATTCA

GCAATTTTCCAAGAAACTGAATTGTATAAGAAACTCATAAGTAATACTTTAAATATTCCAGAGCCCCTACAAATTTCAGA

AAATAACCCAACGCTTTTCCCTTACGTATTTATAGGCGATGAAGCATTCGGGTTGAGCACAAATATTATGCGTCCATACG

GAGGAAACAACCTCAGTGCCGAAAAGAAAATTTTTAACTACAGACTATCGAGAGCTCGCCGTTACATCGAGTGCACATTT

GGCATCTTAACAAATAAATGGAGAATTTTCCACAGACCATTAAATGTCCACACAGAATTAGCAAAATCCATTGTGAGAAC

ATGTTGTGTGTTACACAATTTTGTACGTTCTAGAGATGGATATTCATATGATGATACTCTGACAATAATGGGCTTTCAAA

CAACAACACAGCAAAATTTCAATAGAGGAGGGCGTGCTGCTACTACCACTCGAGATAATTTTACACATTATTTTGTAAAC

GAAAACCCACTTTCATGGCAAAATAATTATATTCATTAATAAATACATACACAACTTACAACGAGCTTTTGGCTTTCCAT

GTTACACCAGAAGCATCATGAACATTTATTGATTGACAAATTCATTCAGAAAAATAAATGTTGTTTACTTACCAAAGCTC

TTTTTTTGTTCAACGGTTTGCTCCTGTTCTCCAAATAAATCTACAACTTCCTCCCAACATTTCTTTTTCAAATCTCTATC

GGAATAATCTGACGAGGAATTATCCCAAAGTGATGGTCTACTTTGAATTTCGATAATAAACCTTTCTGTATCGAAATTAT

CCATCGTATGCAGTTAACAAACTACTCAGATATTAAAATAAGCAGTTAAAAAATAAAACATAACCGCGACACGTGCTGCA

TGCAATGAGGTGGGAGCGTCACTGGCGGCCGGACTGGCGGCCGAGTGAGTGAGACACCATATCGCGCTATAGTAGTTAGT

GGTGGCGCGACCAGCGTGGCGGCGCAACCAGTAACATTCTATGCACGCGTCCGAGTTGGTCGCGCCGCCAAATTGGTTGC

GCAACTAAACGGACACCAGGTGAGTAACTCTCTATAGAGCTGTATACATTTTGTTGGTAGCGGCGACTGGTTGCGCCGCC

ATGGTGTCCCGGTGAAATATAGCCCTTACTGGCCGGCTGCTCGACGATCTCTACGTAGGGCTGGTCGCGGCGCGCCATGC

TATCGTCTATGCCGGCGCCTGCGCCCGCGCCGAACGTCGGGTCCGCCAATGTGATCGCCTCAAAAACATCACTAATATTC

AGTTGTTCCTCATTCTCGGCAGTGCCTATAGTTATGGCGCTTTCTCCAGCATGAATGATCCTATAATAATAACTGTGAAC

TGTTTCTTCGCTCTCGCTCGTCCAAGGAACTAGTTATCAAGGTCTCTCGTCTCGACGTAGTGTATTTTTCAAGTTCCAAG

AATTGTCACAGATAATGTCTCGAACTCTGACACTATATTGCAAACGCGCGGCACAAAATTTGATAAATCCGGCTAATTAA

ATAAGCAAAGAATATTCAGGTAACAAACGTATAGTCGCGAAGTAACACTACTCCGGCTAGTGAACGTCAAAAACTCTAAT

GATAAATCTCAATAGTGTGCATGAGCACCCGACATCACACAAGCAACAACTGCCGGCGATTGGCGGCGCTAAGTGTCACA

TGGCGGCTGAGTGACGTACGCGGAATTGCGAACATAGCGCATGCGTACAGTGTATTCAAAACTTTATTCCATAAGTGCGA

GTAGAAACACAATAGCGGTAGTACTGTTCTAGCCGTCTATAACCGTACCGGTGCTTTGCTACGGTTTCCCGCCAAAATTA

AACAACATTTTTCTTTAGAATTTAATATGTATATTTAGTTATTGTGTTTTTAGTTGTTGACAGATGTTAAATGTGATTTA

TTAAACAAATATCATTGGTTTTAAAAAAAA

>TRINITY_DN14413_c3_g3_i8_Harbinger-1_DW_tp#DNA/PIF-Harbinger964-2779viagenewisewithHarb_Cis1_tp,1intron(1248:1902)[Drosophilawillistoni]

GCTGTAAGAAAGGACAATTCGTCAACATATACGCCGATCTAAGAAGATATCCGGATAAGTTTTTTAATTATGTGAGAATG

TCCGTGAGGTCCTTTGATGAGTTACTGACTTTGTGTGAAAATGATTTGTTAAAGCAAGACACAATATTAAGAAAATCAAT

CAGTCCTGAAGAAAAGTTGTTTGTAACATTAAGGTACCTCGCAAGTGGATGTACGATGAGAGAATTGCATTATAGCTTTC

GTCTAGGACAAACAACATTAAGCACAATAATAAAAGAAGTGTGTGCTGTGATTTGGGATAAGTTGCAAACATGTTTATCA

CTTCCGGCTACTGCAAATGACTGGATGAAAATAGCAGATGGTTTCGAAAAACATGCCAACTTTCCACACTGCATCGGTAG

TATCGATGGAAAGCACATAAGACTGATTCAACCTGCTGATTCTGGTTCGATGTATTATAACTATAAACACTTTTTTTCAT

TGGTGCTGATGGCTGCATGTGACGCTAATTATAATTTCATTTATATAGACGTCGGTGCCTATGGGAAAAGTAGCGATTCA

GCAATTTTCCAAGAAACTGAATTGTATAAGAAACTCATAAGTAATACTTTAAATATTCCAGAGCCCCTACAAATTTCAGA

AAATAACCCAACGCTTTTCCCTTACGTATTTATAGGCGATGAAGCATTCGGGTTGAGCACAAATATTATGCGTCCATACG

GAGGAAACAACCTCAGTGCCGAAAAGAAAATTTTTAACTACAGACTATCGAGAGCTCGCCGTTACATCGAGTGCACATTT

GGCATCTTAACAAATAAATGGAGAATTTTCCACAGACCATTAAATGTCCACACAGAATTAGCAAAATCCATTGTGAGAAC

ATGTTGTGTGTTACACAATTTTGTACGTTCTAGAGATGGATATTCATATGATGATACTCTGACAATAATGGGCTTTCAAA

CAACAACACAGCAAAATTTCAATAGAGGAGGGCGTGCTGCTACTACCACTCGAGATAATTTTACACATTATTTTGTAAAC

GAAAACCCACTTTCATGGCAAAATAATTATATTCATTAATAAATACATACACAACTTACAACGAGCTTTTGGCTTTCCAT

GTTACACCAGAAGCATCATGAACATTTATTGATTGACAAATTCATTCAGAAAAATAAATGTTGTTTACTTACCAAAGCTC

TTTTTTTGTTCAACGGTTTGCTCCTGTTCTCCAAATAAATCTACAACTTCCTCCCAACATTTCTTTTTCAAATCTCTATC

GGAATAATCTGACGAGGAATTATCCCAAAGTGATGGTCTACTTTGAATTTCGATAATAAACCTTTCTGTATCGAAATTAT

CCATCGTATGCAGTTAACAAACTACTCAGATATTAAAATAAGCAGTTAAAAAATAAAACATAACCGCGACACGTGCTGCA

TGCAATGAGGTGGGAGCGTCACTGGCGGCCGGACTGGCGGCCGAGTGAGTGAGACACCATATCGCGCTATAGTAGTTAGT

GGTGGCGCGACCAGCGTGGCGGCGCAACCAGTAACATTCTATGCACGCGTCCGAGTTGGTCGCGCCGCCAAATTGGTTGC

GCAACTAAACGGACACCAGGTGAGTAACTCTCTATAGAGCTGTATACATTTTGTTGGTAGCGGCGACTGGTTGCGCCGCC

ATGGTGTCCCGGTGAAATATAGCCCTTACTGGCCGGCTGCTCGACGATCTCTACGTAGGGCTGGTCGCGGCGCGCCATGC

TATCGTCTATGCCGGCGCCTGCGCCCGCGCCGAACGTCGGGTCCGCCAATGTGATCGCCTCAAAAACATCACTAATATTC

AGTTGTTCCTCATTCTCGGCAGTGCCTATAGTTATGGCGCTTTCTCCGACGTTGTCCATGGCACTGTACGAATAGGCCGC

GGGCCGCGGACACCTCACATCGGTCCTTCCGCACGCGACAATATCAAAACGCACATTTATATAAACTTCGTCGTAACTCG

TTTTGGTTTAAAACTTAACACTTGCCACTTTTATAGTGTACTTTGATAACTTTTATATGCACTATAGCTCACTTTTTAAC

TTAATTCTGAGAGACTGATTTAATAACACACACGATATACTTCACAAATTGTAAACTATAGACGTACCTCCAGCATGAAT

GATCCTATAATAATAACTGTGAACTGTTTCTTCGCTCTCGCTCGTCCAAGGAACTAGTTATCAAGGTCTCTCGTCTCGAC

GTAGTGTATTTTTCAAGTTCCAAGAATTGTCACAGATAATGTCTCGAACTCTGACACTATATTGCAAACGCGCGGCACAA

AATTTGATAAATCCGGCTAATTAAATAAGCAAAGAATATTCAGGTAACAAACGTATAGTCGCGAAGTAACACTACTCCGG

CTAGTGAACGTCAAAAACTCTAATGATAAATCTCAATAGTGTGCATGAGCACCCGACATCACACAAGCAACAACTGCCGG

CGATTGGCGGCGCTAAGTGTCACATGGCGGCTGAGTGACGTACGCGGAATTGCGAACATAGCGCATGCGTACAGTGTATT

CAAAACTTTATTCCATAAGTGCGAGTAGAAACACAATAGCGGTAGTACTGTTCTAGCCGTCTATAACCGTACCGGTGCTT

TGCTACGGTTTCCCGCCAAAATTAAACAACATTTTTCTTTAGAATTTAATATGTATATTTAGTTATTGTGTTTTTAGTTG

TTGACAGATGTTAAATGTGATTTATTAAACAAATATCATTGGTTTTAAAAAAAA

>TRINITY_DN7875_c0_g1_i1_CR1-7_NVi_pol#LINE/L21297-4189viatfastywithCR1-5_NVi_pol,3frameshifts.[Nasoniavitripennis]

AAACGATTCGAGCTTATTCAGAAGCATGGGGACAGAGACCGTGTCAAAAGCTTTGGCCAAGTCCAGAAAAATAGCCAAGC

ACTTTCTTCTGTCATCTAAATTTATAGCTATATATTCCGTGACAGCATGGACTGCATCAGAGGTGGACTTAGAGCGACGA

AAGCCATACTGATTTTTAGATAGCAGATTGTGGGATTCCAAATAATTTACTAACCTAGTATTAATAAGTTTTTCTAGAAT

TTTGGCAATAGAAGAGAGCAGGGAAATTGGCCTGTAGTTAGTGACACGATCCCTGTCACCACCCTTGTGAATGGGAACAA

TTAAAGACTTCTTGAGTTTATTTGGGAAAACTCCCTCGCTAAAACAATGATTAAATAAAAATGTCAAAGGGGCTACTAAA

AAGTCCCTAAACTGTTTGAGGAATCTGTTCGAGATCCTATCCCATCCAACAGCGCAATCGTTTTTTAGACTTGAAATTAT

ATTAATGACTTCATTATGCTCAATTTCCGTCAGTACAAAAGAATATGATGTCCTAGAAAAAATATTGGAAGCGTTATTAG

TGAGCCTGCTACTCCTGGAAATATTTTCTGCCAATTTTTTGCCAACATTGACGAAGTAACTGTTAACTAACTGTACAGAC

TCTTGTGGAGTAGGAGCCAATTTTAATAAATCGATTGGAGCCGGGTTTGTTTTAGCGGTGTTTGTAATTAATTTGATTGC

CTGCCATATTTTTTTATTATCATTTCCTGCATTAGATATTTGATCTTTTTCATATGCTCTTTTAACTTTCCTTAATATTA

AATTACAGTAATTTCTGTAGCGTGTATACGTGATTCTGAGGATTTCGTTATTAGGAGATTTCTTAAGTTTGTAATGTAAA

CGGTCCCTGTTTCGCATGCAACGCATCAAACCCGGAGTTATCCACGGCTTTATAATTTTCTTTCGCCCTGCTAAGGATAT

TGATTGAGTATTCTTGTCTATAGCCGTTGTCACAGAATCAATAAAGTAATTTAAAGAATAATCTGGATCTGAAGATTCGT

ATATGGGAGTAAAGTCAATTTTAGCTATGTCAAGTTTAACTTGTTCGAAATTTATTTTATTTATAGTTTTTACAGAATAA

TTACGTGTTTGTTTTTTGTATAGGGATAACAACACCGGTTTGTGATCAGTCAGTGTGGTATGAACAACTAACGTAGTTGC

CAGATATTTTGTTTTTAATAACACATGATCAAGACAAGTTCCACTGGCTTCCCGAGTGACATAGGAGTGGGCTGGTAGCA

GTGAGTGATAGGCCGCCAGCGTCAAGTATTGATCTATATTACGGTGGGGTTTGTCCGAATTTATAGCAATATTTATATCC

CCTATTACAGCGATAGTTTTAAACTGTTTTATTTGTTCCAAAGTATTATTCAAAGAGTTACAAAAATGGTCTAGATTTGT

ATAGGAAGGGGATCGGTATATAGCGATGATGGCAATATCGCTTCCTATCCTAATCAGTAGGCCATTAGCTTCTGCAATGG

AAGGTTCTTCCACCGTGATCGCAAGTCCTTCCCTACTGTACACAACTACACCGTCATTTTGTAACACGTTATTTTTCGTA

GAAAATACCTGGTAACCTTGTAGGCTCGGAATGATGGGCGCACAAGATAACCAACATTCGGTTAATATAAGTATATCTAA

ATCTAGTTTAATTCGAGTCAGTAAAACTTCAAATCCTGGTAAATTCGAATTAATACTTCTTATGTTCTGTGTAAGTATAG

TAAGACCGGATACAGTATTGGGCATCAGTCGACTACAGTCCTCTAAGTTGCACTCGAAACTTTTTGAGACGTTGTAATTA

TCCAGATCATCAGGTAAATCACCTTGACTGTTGAATTTTAAATAAAAAGGAAATATATTCGTCTATAAAAGAGGACAATA

AATCAAAATATTTTAGTAGTTTTGGGCTGGAAAATGTTTTACGTGAGTTAAGAGAAATAAGAAAAATATAGCATTCATAA

GTAAGCGAATTGATGTAATATGATAGTGTATAAATGTATTGCGTGCAATTTAAACTAGAGTTGTAGTAATTATATATTAG

TGGCAAATAAACATAAAAATAAAAAAGAGCGCATGCAAAAGTTACTATGTGTGAAACACTTCCTTTACAGAAAGTCCAAA

AAATATACTTAGAAACTAGTCATTTGACATTTTTTAACTTTTCCAAATCCGCTTCAGTTTCAATTTTAATCTGTGCCCGG

CCGGTGCTTTTACGAAGGTAAACTATTCCTCTCGAAGTCCAGCAAAAATCATACCCATATTCCTTTTGAAAAGCCCGAGC

CAAATAGAAAATTTTCTGTGTTTTTTGCGTCAGAGTTTCAGCAACGAAGACAGGTTTCAAGGTATCAGTTTTAATGAAAT

GTGTTGTATTTAATTTTTCACCCTTTTGCTTGGTTTTATTGAAATCTTTTACTGCAGACAGCACCTTTTCTTTCATCAGT

ACAGAGGTAAATTCAGTAATTATCGGATTTGAAGAGTCCTTGGCCGTTGTACGATAAATATCCTTGATATCCGATTCCTT

AATATCAATATTGAGTGTTTTACCCATTTGTTGTGCTATTAGGCAGAGTTCAGACTTGGTTTCATTCCTGTCAGGTTTCG

TTTTAGGGATATTGCGAATTTCAATTCCTGATGCCCGTGTTTTACGTTCTAAAGTGTCAATCTTCTGCTCCAATGAATTA

ATGTATTTTTTATCCTCCTTTTTTGCGTCTTCCAGTAATTTAATGCGAGATAAAATTTCATCATATTTATGAGACATTGT

CTCGACAGATTTAGTTAATTCGGCATTCTGTTCCCGAAGACTATTGACAGAAACCAGCAAATCTTGAAAGCGTTTATCTT

GCTCATTCGAAAGAGTGTCGAACATTTTTTTTAGTGTACACATAAAACTATTTATGTCATCACCATCAAACTTGCGTTTT

TTTCGCTCCGTAACATTCATGGCAAGTTGATGTAGATTTGGTTCAGACAGCGACACCGAAACGTTGTTTCCAATAGTCGC

TTCCGCGTCAGTGCTCTGCGTAATCGGCGACTTTTGCGACACAGGCGGCGTAGGAGCATTGAGGAGTGTAGGGGGGGGCG

ATCGACGAACTGACATAATGACAGAAATGTACTAGGGCCGCAGCAATTTGTAGGCGGTAAATAAATAAACTCCTTTGCCT

TAAATGCAAGACCAGTTTTAACAGGGGTCTAATAACCTGGGCACGTATCGACGATTTAGTACAAATCTAGCGGTGGCCAC

TATAACGTTGACCCCAGGTACTCACACGTCGGAGTTAATAGAAAAGTGTATGGAAGGAAAGAATCATCCCAAGTTGTTGT

TGAGACGGA

>TRINITY_DN14445_c0_g1_i2_Mariner-1_TCa_tp#DNA/TcMar-Tc1417-1479viatfastywithMARINCE1_tp,1frameshift.[Triboliumcastaneum]

TAAGTTGTTCCTGTGATTTAATGTATTATACAGGGTTACAGGAAAAGTCGACCTAGATCTGCTAAGGGCGTGTAGTTTGC

AACGTTTCTGACTGATTCTACTATGAAATACCCTATCCTAAACTCAACGGTTTTCGAGATATTCAATTTTTTACTTTTTT

TGAAAAATTATCAAATTTTTGTCAATTTAAGCACATAAATTAAAATTTGTGAGTCCAATTCATCGTTTTATTTGTTTTTT

ACACAAACAATAAGATGTTCTATTGTTTCCATAACCAAAATTGTGAGTAATGTAACTAAATTAGGTCATAAAAGGGGTTG

AAAGTTAAAAAAAGTAAGTCAAATTTTTGAAACTTGTTTTTCGTTAGTGAATACCTAAAAAAAAATGTATGGGAGATAGT

CGGCGGATGTGCTATAAAATATCTACGTTTCAACAGCTAACCAACAAGGTACAACAGTCAAGGTTTTCTTTTTTTAATCA

TCCAAAAACTTAGCTACTAAGCTACTGAGAGGGTAAAAAATTACCGTGTTGATTACGTTTAATTTAACACCTGCACTCAG

AAACGAAAATAAAACAAGATTGATAATAATAAACATTTATTGGATTGTGTAACAATTTTTAAATTTCGTGCTCAACATGT

CCACCTCTGTTACGTACGCATAGTCGCAATCTTCTCCTCAATCCACTTTTAACAACACTTGTTGTTAATGATCCGCGTGT

CTCCGTGTTATTTGATCCGCCGCGTTAATTATTTTGGCTCTCAACTCTTCCACAGTGGAAATTGGGTTGGGCTCCCTGTA

CACGAGGCTCTTCATGTGCCCCCATACATAAAAATCCATGGGGGTAAGGTCCGGACTTCTGGCTGGCCACGGTAAAGGCC

CACCTCGTCCGATCCATTTATTAGCGTAGTTTGTGTCTAACCATACTCGGACACTTCTCCTGTAATGTGCAGGGCAGCCA

TCGTGTTGAAACCACATTTCTTGAAATAGTTGTAATGGTAAGTCGTCGAGCAAATCGCTTAGGTCCCCTCTTAGGAAATT

TTCGTAATCTTCTCCATTTAAATTGTCTGGGAGAAAGTAAGGCCCAACCAAATAATTTGAAATTATTCCCATCCACACAT

TTACGCTAAATCTGCGTTGTGACCCTTTCACTCTCTTCTTTTTAGGATTACCTTGTTGTTGTGGGGCCCAATAATGAGCA

TTATGGTAGTTGGTAATACCGTCTTTATCAAACTTTGACTCGTCCGTCCACAATTTTTTTTAAAAAATGAAGGGTCTTCT

GCATCGGCGT

>TRINITY_DN14445_c0_g1_i3_Mariner-1_TCa_tp#DNA/TcMar-Tc1417-1479viatfastywithMARINCE1_tp,1frameshift.[Triboliumcastaneum]

TAAGTTGTTCCTGTGATTTAATGTATTATACAGGGTTACAGGAAAAGTCGACCTAGATCCGCTAAGGGCGTGTAGTTTGC

AACGTTTCTGACTGATTCTACTATGAAATACCCTATCCTAAACTCAACGGTTTTCGAGATATTCAATTTTTTACTTTTTT

TGAAAAATTATCAAATTTTTGTCAATTTAAGCACATAAATTAAAATTTGTGAGTCCAATTCATCGTTTTATTTGTTTTTT

ACACAAACAATAAGATGTTCTATTGTTTCCATAACCAAAATTGTGAGTAATGTAACTAAATTAGGTCATAAAAGGGGTTG

AAAGTTAAAAAAAGTAAGTCAAATTTTTGAAACTTGTTTTTCGTTAGTGAATACCTAAAAAAAAATGTATGGGAGATAGT

CGGCGGATGTGCTATAAAATATCTACGTTTCAACAGCTAACCAACAAGGTACAACAGTCAAGGTTTTCTTTTTTTAATCA

TCCAAAAACTTAGCTACTAAGCTACTGAGAGGGTAAAAAATTACCGTGTTGATTACGTTTAATTTAACACCTGCACTCAG

AAACGAAAATAAAACAAGATTGATAATAATAAACATTTATTGGATTGTGTAACAATTTTTAAATTTCGTGCTCAACATGT

CCACCTCTGTTACGTACGCATAGTCGCAATCTTCTCCTCAATCCACTTTTAACAACACTTGTTGTTAAATTATGCCTTAT

TTGATCCGCCGCGTTAATTATTTTGGCTCTCAACTCTTCCACAGTGGAAATTGGGTTGGGCTCCCTGTACACGAGGCTCT

TCATGTGCCCCCATACATAAAAATCCATGGGGGTAAGGTCCGGACTTCTGGCTGGCCACGGTAAAGGCCCACCTCGTCCG

ATCCATTTATTAGCGTAGTTTGTGTCTAACCATACTCGGACACTTCTCCTGTAATGTGCAGGGCAGCCATCGTGTTGAAA

CCACATTTCTTGAAATAGTTGTAATGGTAAGTCGTCGAGCAAATCGCTTAGGTCCCCTCTTAGGAAATTTTCGTAATCTT

CTCCATTTAAATTGTCTGGGAGAAAGTAAGGCCCAACCAAATAATTTGAAATTATTCCCATCCACACATTTACGCTAAAT

CTGCGTTGTGACCCTTTCACTCTCTTCTTTTTAGGATTACCTTGTTGTTGTGGGGCCCAATAATGAGCATTATGGTAGTT

GGTAATACCGTCTTTATCAAACTTTGACTCGTCCGTCCACAATTTTTTTTAAAAAATGAAGGGTCTTCTGCATCGGCGT

>TRINITY_DN14417_c2_g2_i1_Proto2-8_CS1_pol#LINE/Proto21280-4168[Capitellasp.1]

TTTTTTTTTTCTATAAAATATTTTTATTTGAGCGGTATACGATCCGCGATAATTTCTCGATAACTACAAAATTCCTAACT

AAAGGAATGTACAGGTTACACACAACTTTGCGTCCGACACATCATACATATATTACAGGCTGTATGTAACTGACAATGAA

TCACATAATATATGAAAGAAATACATAGTCAGTTAAGTACAGCTGAGACCAGAAAAAATATATTGACTCTTCAAAATGCA

ACATTTTAAAACTTAAGATCGCGGTACATTTTCTTTGACCAAAGAGTTTTTAAGTCTTCAAGAGTACTGTGTTACCGAGT

AAAATGGGTTTGCAAAAATCAGATAAACAGTCGCTCATTGTGAACTATAGTTAGGTAAATCTATTGAATTTGGCGACAAC

GGAAAAAACCTTCATATAATTTGGTCGAGATCACATTTTGTTTAGTCTATATTTTTCCTGGTCCATAACATATTCTAAAT

ACACTTATTACAACAAGGATCTTGGTACTTGGGCGTATTAACCCCCTAACAAGAGATATATTTATACATACATTAAATGT

AACCTAAAAATTAAATTAAAAACTAGAAAATGCTATTGTGTTACCCTAAAGTGTCCTTTCCTACATATTTACGCGTAATG

CGTAAAAATAAAGATTAAATTAATTAATTAAATAACTGTTATAATTAAATCGTAAATCCCGCGTAAATTATTAATTGAAA

ACCATAATGGCGTCGCCATTCCACTCATGAAATCAAACTATTATATGCAAAGGTCTTAATTTAAACAGCCACGTGAGACG

TCGCATTTTTGAGATCGGACGTATTGATCGAAATCAAAAATGTCGACGGTGGTAAAGTGTAATAACTGCAATGTTGTTAT

TAATGAAGTCCTTGCATTTATTTGCAACAAGATTGATGTGATGGATGAAGAGAGCATTAGTCGAATATGTATTAGTGCAT

TCTCGGAATACGAAGTGAAAAATGCAAAAGATTTGCTATACGAGTCAATTTCGACAACAAGGCGCAAAAAGACAAGAAAA

CGTGATGGTAAAACTCTCCGTGAGATTGATGATATTATTTGTTTGCTCAAAGAAACGGATCCCGAAGAAATACCAATTTT

TGTGGCACGGGATTTGCAAAAGTTGCCACCAGTACTGTTCGACCACGTAGATGTTACGCGGCTTCTCAAAGACTTATTAA

AAATGCAAGGTGATATTAAGCAAATCAAGGAACAATATGTGACTGTGGAACAGATACAACCGTTGAAATCTGAGTTTGAA

TTATTAAAAAACGAGGTAACAGCAACGAGAACATCAATTTATGTTGAGGGTAGTAAGAATACATCATCCCAGCAGAACAT

CGAGACCGCGGATATTACGATTGTGAATCATGAAGAGGGTAAAAAATGTAAACAACGAAATATGCGCGGGAATCGGGCGT

TGCACCCACCTCAGCCGAGCAGCGCGAGCGCGTCGGCCGTGGCGTCGGCGTACATGAGACCCGAAGATATGCCGAACAAA

CACGATCGCGAGACGAACGCGCCCGCTATGCAATTTGTCGTCCCCGAGTCTCAAGATAAAACGGCTCAGAGTCACGAACC

CAACCCGAACATACACGAGCGGCAGCCGAGCGCGTCGCAAGTCGCAGCGGGACCTCAAGGTCAAGCGTCGAACTGTAACG

CGGTGAAGCCGAAAATTCACGAGCGAGAGCCGAACCGAGCCGAGCAGGAGTCGCATGGACATACGGTCGCGATGAATGTA

CAATTGCATCAGAAATCATTTGCCGATGTTGCACAAGACGGAACATGGAAATCTAAAACTGTGCCTGAAGAATGGATACA

GGTACAGAGAAAAAGATACCGGAATAGATTTGTGGGGAACAGAGGAAAAGCAATTGTTACGCCGGATTCTAATTTCAAGG

CGGCAGAAATAAAAATTCCGATATATATTTATAATGTTGCGAAGGCTGTAACCGTGTGTAGCATAACCGAGTATATTCAA

AATAAGGCGAATATTGAAGTTACCATGGAAAAAATGAATATGACAGTTGCAAAAGATTATGATTCATACAAAATATTTAT

ACCTAGAAATAAATTGGACATTTTTATGGAACATGATTTTTGGCCGGAGGGTATAGCTTTTCGTCGTTTTGTAGATTTTA

AATGGCGAACAAATGGCAGACATAACAAATTGAATTTAAACTTAAATAATCATAATGAATGTAGCCAAGTTAATTAGTTT

TAATTGTAAAGGAGTGAGCCGCTCGAAAGAATGTGTCAGGCGAATTTGTAACTCAGCGGATGTCATAGCCTTGCAAGAGA

CTTGGCTCCTACCGCATGATATCCCTTGCTTGGGTAGTATAGACGACAGTTTTGCATACACCGGCAAGTCAGCAGTGGAT

ACTTCGGCCGGTATACTGAGAGGCAGGCCATATGGGGGTGTGGCTTTACTTTGGAAGAAGGACGTGTTCCCAATAGCCAC

AGTCATCGAGTGTACCAGTGTGCGTTTATGTGCGGTGAAATTAGGCATAAATAATCGCTCTTTACTAGTATTTTCTGTAT

ACATGCCTACAAACTCTATTGATAATTTATTAGAATTCACCGAAACTTTGTGCGAAATTAATGCAATTATCGAAAACAAC

AGTGTAGAGTCAGTGTACATGTTGGGGGACTTTAATGCCCACCCGGGTGAGCTATTTTATAATGAACTAAGTAGTTTTTG

CATTGAACAGAGGTGGTCATGCGCGGATATAGAACTATTGCCACCAGAGTCGTATACGTTTGTCAGCGACGCGCACGGTT

CGCGAAGATGGCTCGACCACTGCGTGCTGACGAGTGCCGCGCGGCTCTCTGTGTTAAATATTTCAATTTTGTATGATATT

TATTGGTCAGACCATTACCCTGTCCTTATGGAGTGCAATTTAGATTTAGTTAAAGCCAAAGTTGTTTCAATTAGTTATAG

TAAAAACAAGGTTATATGGGGAGACAGGGACAGTAGTCAAATTAAGAAATATATTAATATATGTAACTCACAGCTTAAGT

TTATTGATTTTCCTGAGCAATGTAGTGAATGTGCAGATAGATTTTGTGATAACGTTAACCATAGAGTAATATTAGATAAA

ATGTACAATGATTTAGTTACAGTATTGACAGAAGCTTCAAAAGCGAGTAAACAATGTTGTAAAATACACAAGCGTAAAAA

GTATGTCACAGGATGGAACAAACATGTCAGAGATAGTCACGTTATTGCCAGGCGTTGTTACATAGATTGGTTAGCTAGTG

GGAAACCGACTTCGGGAGAAAAATATAATGATATGTGTAATTCTAGAATTATATTTAAACAGAAATTAAAATGGTGTCAA

AATAATGAACAACAGATTAAAATGGATAAAATTGCAGATCAACATAACAAGAAAGATTTTAAAAATTTTTGGAAGAATAC

AAATAGCTTAAATCCGAAGCCTGGCCATCCCGTGAGTGTTTCTGGCATTCATGAACCAACTGAAATAGCTGAAGCGTTTC

GTCTTCAGTTAAAGATAGACTCCTTGTCTCCTACAGACACTCACAAGGACGTAAAGAGATGTAAAAGTGCGGAGTCGGAT

GTCGCTGTCAGGTTCACTAATAAGCAGGTGAAGAATGTTATCCGAGGTATGGTCAAAGGCAAGTCACCAGGTCATGATGG

TCTAAGTATAGAACATCTAAAGTACGCAGGAGCTCATTTACCGCGGATTTTAACAATGTTTTTTAATCTCTGTATTAGCC

ATTCATACCTACCAGATCGGTTAATGCATACAGTTGTAATTCCCATAATTAAAAACAGGAGTGGAGATGCCTCGGATATA

TCAAATTACAGGCCGATATCTTTAGCCACAATAGTGGCCAAAGTATTGGACAGTCTGCTTGACCAGCACTTGAATAAACA

TATCAACCTCCACGACCAACAATTTGGCTTCCGGACAGGTTTGTCAACAGAGAGTGCTATCATGTGCCTCAAGGAGACTG

TTCAGTACTACGTGACCAGAAAGACGCCGGTGTATGCTTGCTTTTTGGACCTGTCAAGGGCGTTCGACCTCGTTTCTTAC

AGAAAGTTATGGGAAAAGTTGGAAGATGAAACGTCTTGTAACCAGGAGGTTGTGTCCTTATTGAAACATTGGTACAACAA

TCAGACTAATGTAGTGAAATGGGCTGGGGCGTCATCAGCAGTGTACAGGTTGAATTGTGGAGTGAGACAGGGTGGCCTGA

CCTCACCCAGACTCTTCAACCTGTATATGAACGGACTTATTGGTGAGCTCAACAGTACCGGCGTCGGATGTCACATAGAC

GGTGTTTGTGTTAACAACATAAGCTACGCAGACGACATGGTACTGTTGAGCCCTTCAATGGCAGCGTTAAAAAAGTTAGT

TAGAATATGTGAAGTGTATGCGGAGGCTCATGGACTGAAATACAATTCGAGAAAGAGTGAGATTATGATATTTAAGGCTA

GCAACAAAAGTTATGCAACAGTTCCTGTCACCTTAAGTGGTAGCGAGTTGAAGCAAGTCTCGAATTTCAAGTATCTCGGT

CACTGGGTCTCCGAGGACTTGAAGGATGATAAAGACTTGGAGAGGGAACGTAGGGCGCTGGCGGTCAGAAGCAATATGTT

GGCCCGCAGGTTTCGGCGATGCAGTGATTCGGTAAAAGTGACTTTGTTTAAAGCCTATTGCCAGTCATTGTATACGTGTT

CTCTGTGGGTAAACTATACGCGGATGACGTATAACGCCCTGCGTGTACAATACAACAATACATTTAGGACGCTGCTCAGG

TTGCCGCGGTTCTGCAGTGCCTCGGGCATGTTTGCTGACGCGCACACTGATGGTTTTAATGCCATCATCAGAAAACGCAG

TGCTTCACTTCTTCACCGAGTGCGGACTAGCACCAATAGCGTCCTAAACGTATTGACTGATAGGTGGGACTCTCAGTTGC

TAAGACACTGGTTAAGCCTTCACACTGTGTTGTAATTTGTATTTTATTTTATTTCTTTGATGTTTTGTGTTACCTACTTT

TTATATGGACCTGGTTTTATTAGTGGCCCTACAATTTGGGCTGGAATGGCCGGCAGGGTAAACCATAA

>TRINITY_DN14417_c2_g2_i6_Proto2-8_CS1_pol#LINE/Proto21280-4168[Capitellasp.1]

TTTTTTTTTTCTATAAAATATTTTTATTTGAGCGGTATACGATCCGCGATAATTTCTCGATAACTACAAAATTCCTAACT

AAAGGAATGTACAGGTTACACACAACTTTGCGTCCGACACATCATACATATATTACAGGCTGTATGTAACTGACAATGAA

TCACATAATATATGAAAGAAATACATAGTCAGTTAAGTACAGCTGAGACCAGAAAAAATATATTGACTCTTCAAAATGCA

ACATTTTAAAACTTAAGATCGCGGTACATTTTCTTTGACCAAAGAGTTTTTAAGTCTTCAAGAGTACTGTGTTACCGAGT

AAAATGGGTTTGCAAAAATCAGATAAACAGTCGCTCATTGTGAACTATAGTTAGGTAAATCTATTGAATTTGGCGACAAC

GGTCGAGATCACATTTTGTTTAGTCTATATTTTTCCTGGTCCATAACATATTCTAAATACACTTATTACAACAAGGATCT

TGGTACTTGGGCGTATTAACCCCCTAACAAGAGATATATTTATACATACATTAAATGTAACCTAAAAATTAAATTAAAAA

CTAGAAAATGCTATTGTGTTACCCTAAAGTGTCCTTTCCTACATATTTACGCGTAATGCGTAAAAATAAAGATTAAATTA

ATTAATTAAATAACTGTTATAATTAAATCGTAAATCCCGCGTAAATTATTAATTGAAAACCATAATGGCGTCGCCATTCC

ACTCATGAAATCAAACTATTATATGCAAAGGTCTTAATTTAAACAGCCACGTGAGACGTCGCATTTTTGAGATCGGACGT

ATTGATCGAAATCAAAAATGTCGACGGTGGTAAAGTGTAATAACTGCAATGTTGTTATTAATGAAGTCCTTGCATTTATT

TGCAACAAGATTGATGTGATGGATGAAGAGAGCATTAGTCGAATATGTATTAGTGCATTCTCGGAATACGAAGTGAAAAA

TGCAAAAGATTTGCTATACGAGTCAATTTCGACAACAAGGCGCAAAAAGACAAGAAAACGTGATGGTAAAACTCTCCGTG

AGATTGATGATATTATTTGTTTGCTCAAAGAAACGGATCCCGAAGAAATACCAATTTTTGTGGCACGGGATTTGCAAAAG

TTGCCACCAGTACTGTTCGACCACGTAGATGTTACGCGGCTTCTCAAAGACTTATTAAAAATGCAAGGTGATATTAAGCA

AATCAAGGAACAATATGTGACTGTGGAACAGATACAACCGTTGAAATCTGAGTTTGAATTATTAAAAAACGAGGTAACAG

CAACGAGAACATCAATTTATGTTGAGGGTAGTAAGAATACATCATCCCAGCAGAACATCGAGACCGCGGATATTACGATT

GTGAATCATGAAGAGGGTAAAAAATGTAAACAACGAAATATGCGCGGGAATCGGGCGTTGCACCCACCTCAGCCGAGCAG

CGCGAGCGCGTCGGCCGTGGCGTCGGCGTACATGAGACCCGAAGATATGCCGAACAAACACGATCGCGAGACGAACGCGC

CCGCTATGCAATTTGTCGTCCCCGAGTCTCAAGATAAAACGGCTCAGAGTCACGAACCCAACCCGAACATACACGAGCGG

CAGCCGAGCGCGTCGCAAGTCGCAGCGGGACCTCAAGGTCAAGCGTCGAACTGTAACGCGGTGAAGCCGAAAATTCACGA

GCGAGAGCCGAACCGAGCCGAGCAGGAGTCGCATGGACATACGGTCGCGATGAATGTACAATTGCATCAGAAATCATTTG

CCGATGTTGCACAAGACGGAACATGGAAATCTAAAACTGTGCCTGAAGAATGGATACAGGTACAGAGAAAAAGATACCGG

AATAGATTTGTGGGGAACAGAGGAAAAGCAATTGTTACGCCGGATTCTAATTTCAAGGCGGCAGAAATAAAAATTCCGAT

ATATATTTATAATGTTGCGAAGGCTGTAACCGTGTGTAGCATAACCGAGTATATTCAAAATAAGGCGAATATTGAAGTTA

CCATGGAAAAAATGAATATGACAGTTGCAAAAGATTATGATTCATACAAAATATTTATACCTAGAAATAAATTGGACATT

TTTATGGAACATGATTTTTGGCCGGAGGGTATAGCTTTTCGTCGTTTTGTAGATTTTAAATGGCGAACAAATGGCAGACA

TAACAAATTGAATTTAAACTTAAATAATCATAATGAATGTAGCCAAGTTAATTAGTTTTAATTGTAAAGGAGTGAGCCGC

TCGAAAGAATGTGTCAGGCGAATTTGTAACTCAGCGGATGTCATAGCCTTGCAAGAGACTTGGCTCCTACCGCATGATAT

CCCTTGCTTGGGTAGTATAGACGACAGTTTTGCATACACCGGCAAGTCAGCAGTGGATACTTCGGCCGGTATACTGAGAG

GCAGGCCATATGGGGGTGTGGCTTTACTTTGGAAGAAGGACGTGTTCCCAATAGCCACAGTCATCGAGTGTACCAGTGTG

CGTTTATGTGCGGTGAAATTAGGCATAAATAATCGCTCTTTACTAGTATTTTCTGTATACATGCCTACAAACTCTATTGA

TAATTTATTAGAATTCACCGAAACTTTGTGCGAAATTAATGCAATTATCGAAAACAACAGTGTAGAGTCAGTGTACATGT

TGGGGGACTTTAATGCCCACCCGGGTGAGCTATTTTATAATGAACTAAGTAGTTTTTGCATTGAACAGAGGTGGTCATGC

GCGGATATAGAACTATTGCCACCAGAGTCGTATACGTTTGTCAGCGACGCGCACGGTTCGCGAAGATGGCTCGACCACTG

CGTGCTGACGAGTGCCGCGCGGCTCTCTGTGTTAAATATTTCAATTTTGTATGATATTTATTGGTCAGACCATTACCCTG

TCCTTATGGAGTGCAATTTAGATTTAGTTAAAGCCAAAGTTGTTTCAATTAGTTATAGTAAAAACAAGGTTATATGGGGA

GACAGGGACAGTAGTCAAATTAAGAAATATATTAATATATGTAACTCACAGCTTAAGTTTATTGATTTTCCTGAGCAATG

TAGTGAATGTGCAGATAGATTTTGTGATAACGTTAACCATAGAGTAATATTAGATAAAATGTACAATGATTTAGTTACAG

TATTGACAGAAGCTTCAAAAGCGAGTAAACAATGTTGTAAAATACACAAGCGTAAAAAGTATGTCACAGGATGGAACAAA

CATGTCAGAGATAGTCACGTTATTGCCAGGCGTTGTTACATAGATTGGTTAGCTAGTGGGAAACCGACTTCGGGAGAAAA

ATATAATGATATGTGTAATTCTAGAATTATATTTAAACAGAAATTAAAATGGTGTCAAAATAATGAACAACAGATTAAAA

TGGATAAAATTGCAGATCAACATAACAAGAAAGATTTTAAAAATTTTTGGAAGAATACAAATAGCTTAAATCCGAAGCCT

GGCCATCCCGTGAGTGTTTCTGGCATTCATGAACCAACTGAAATAGCTGAAGCGTTTCGTCTTCAGTTAAAGATAGACTC

CTTGTCTCCTACAGACACTCACAAGGACGTAAAGAGATGTAAAAGTGCGGAGTCGGATGTCGCTGTCAGGTTCACTAATA

AGCAGGTGAAGAATGTTATCCGAGGTATGGTCAAAGGCAAGTCACCAGGTCATGATGGTCTAAGTATAGAACATCTAAAG

TACGCAGGAGCTCATTTACCGCGGATTTTAACAATGTTTTTTAATCTCTGTATTAGCCATTCATACCTACCAGATCGGTT

AATGCATACAGTTGTAATTCCCATAATTAAAAACAGGAGTGGAGATGCCTCGGATATATCAAATTACAGGCCGATATCTT

TAGCCACAATAGTGGCCAAAGTATTGGACAGTCTGCTTGACCAGCACTTGAATAAACATATCAACCTCCACGACCAACAA

TTTGGCTTCCGGACAGGTTTGTCAACAGAGAGTGCTATCATGTGCCTCAAGGAGACTGTTCAGTACTACGTGACCAGAAA

GACGCCGGTGTATGCTTGCTTTTTGGACCTGTCAAGGGCGTTCGACCTCGTTTCTTACAGAAAGTTATGGGAAAAGTTGG

AAGATGAAACGTCTTGTAACCAGGAGGTTGTGTCCTTATTGAAACATTGGTACAACAATCAGACTAATGTAGTGAAATGG

GCTGGGGCGTCATCAGCAGTGTACAGGTTGAATTGTGGAGTGAGACAGGGTGGCCTGACCTCACCCAGACTCTTCAACCT

GTATATGAACGGACTTATTGGTGAGCTCAACAGTACCGGCGTCGGATGTCACATAGACGGTGTTTGTGTTAACAACATAA

GCTACGCAGACGACATGGTACTGTTGAGCCCTTCAATGGCAGCGTTAAAAAAGTTAGTTAGAATATGTGAAGTGTATGCG

GAGGCTCATGGACTGAAATACAATTCGAGAAAGAGTGAGATTATGATATTTAAGGCTAGCAACAAAAGTTATGCAACAGT

TCCTGTCACCTTAAGTGGTAGCGAGTTGAAGCAAGTCTCGAATTTCAAGTATCTCGGTCACTGGGTCTCCGAGGACTTGA

AGGATGATAAAGACTTGGAGAGGGAACGTAGGGCGCTGGCGGTCAGAAGCAATATGTTGGCCCGCAGGTTTCGGCGATGC

AGTGATTCGGTAAAAGTGACTTTGTTTAAAGCCTATTGCCAGTCATTGTATACGTGTTCTCTGTGGGTAAACTATACGCG

GATGACGTATAACGCCCTGCGTGTACAATACAACAATACATTTAGGACGCTGCTCAGGTTGCCGCGGTTCTGCAGTGCCT

CGGGCATGTTTGCTGACGCGCACACTGATGGTTTTAATGCCATCATCAGAAAACGCAGTGCTTCACTTCTTCACCGAGTG

CGGACTAGCACCAATAGCGTCCTAAACGTATTGACTGATAGGTGGGACTCTCAGTTGCTAAGACACTGGTTAAGCCTTCA

CACTGTGTTGTAATTTGTATTTTATTTTATTTCTTTGATGTTTTGTGTTACCTACTTTTTATATGGACCTGGTCTGAAAT

AAAGATTTATTATTATTATTATTATTATTATTGTCTCCGTGCTCGTTAGTCGTATCCTTCATTTTATAAGTCTGATTT

>TRINITY_DN14417_c2_g2_i7_Proto2-8_CS1_pol#LINE/Proto21280-4168[Capitellasp.1]

TTTTTTTTTTCTATAAAATATTTTTATTTGAGCGGTATACGATCCGCGATAATTTCTCGATAACTACAAAATTCCTAACT

AAAGGAATGTACAGGTTACACACAACTTTGCGTCCGACACATCATACATATATTACAGGCTGTATGTAACTGACAATGAA

TCACATAATATATGAAAGAAATACATAGTCAGTTAAGTACAGCTGAGACCAGAAAAAATATATTGACTCTTCAAAATGCA

ACATTTTAAAACTTAAGATCGCGGTACATTTTCTTTGACCAAAGAGTTTTTAAGTCTTCAAGAGTACTGTGTTACCGAGT

AAAATGGGTTTGCAAAAATCAGATAAACAGTCGCTCATTGTGAACTATAGTTAGGTAAATCTATTGAATTTGGCGACAAC

GGTCGAGATCACATTTTGTTTAGTCTATATTTTTCCTGGTCCATAACATATTCTAAATACACTTATTACAACAAGGATCT

TGGTACTTGGGCGTATTAACCCCCTAACAAGAGATATATTTATACATACATTAAATGTAACCTAAAAATTAAATTAAAAA

CTAGAAAATGCTATTGTGTTACCCTAAAGTGTCCTTTCCTACATATTTACGCGTAATGCGTAAAAATAAAGATTAAATTA

ATTAATTAAATAACTGTTATAATTAAATCGTAAATCCCGCGTAAATTATTAATTGAAAACCATAATGGCGTCGCCATTCC

ACTCATGAAATCAAACTATTATATGCAAAGGTCTTAATTTAAACAGCCACGTGAGACGTCGCATTTTTGAGATCGGACGT

ATTGATCGAAATCAAAAATGTCGACGGTGGTAAAGTGTAATAACTGCAATGTTGTTATTAATGAAGTCCTTGCATTTATT

TGCAACAAGATTGATGTGATGGATGAAGAGAGCATTAGTCGAATATGTATTAGTGCATTCTCGGAATACGAAGTGAAAAA

TGCAAAAGATTTGCTATACGAGTCAATTTCGACAACAAGGCGCAAAAAGACAAGAAAACGTGATGGTAAAACTCTCCGTG

AGATTGATGATATTATTTGTTTGCTCAAAGAAACGGATCCCGAAGAAATACCAATTTTTGTGGCACGGGATTTGCAAAAG

TTGCCACCAGTACTGTTCGACCACGTAGATGTTACGCGGCTTCTCAAAGACTTATTAAAAATGCAAGGTGATATTAAGCA

AATCAAGGAACAATATGTGACTGTGGAACAGATACAACCGTTGAAATCTGAGTTTGAATTATTAAAAAACGAGGTAACAG

CAACGAGAACATCAATTTATGTTGAGGGTAGTAAGAATACATCATCCCAGCAGAACATCGAGACCGCGGATATTACGATT

GTGAATCATGAAGAGGGTAAAAAATGTAAACAACGAAATATGCGCGGGAATCGGGCGTTGCACCCACCTCAGCCGAGCAG

CGCGAGCGCGTCGGCCGTGGCGTCGGCGTACATGAGACCCGAAGATATGCCGAACAAACACGATCGCGAGACGAACGCGC

CCGCTATGCAATTTGTCGTCCCCGAGTCTCAAGATAAAACGGCTCAGAGTCACGAACCCAACCCGAACATACACGAGCGG

CAGCCGAGCGCGTCGCAAGTCGCAGCGGGACCTCAAGGTCAAGCGTCGAACTGTAACGCGGTGAAGCCGAAAATTCACGA

GCGAGAGCCGAACCGAGCCGAGCAGGAGTCGCATGGACATACGGTCGCGATGAATGTACAATTGCATCAGAAATCATTTG

CCGATGTTGCACAAGACGGAACATGGAAATCTAAAACTGTGCCTGAAGAATGGATACAGGTACAGAGAAAAAGATACCGG

AATAGATTTGTGGGGAACAGAGGAAAAGCAATTGTTACGCCGGATTCTAATTTCAAGGCGGCAGAAATAAAAATTCCGAT

ATATATTTATAATGTTGCGAAGGCTGTAACCGTGTGTAGCATAACCGAGTATATTCAAAATAAGGCGAATATTGAAGTTA

CCATGGAAAAAATGAATATGACAGTTGCAAAAGATTATGATTCATACAAAATATTTATACCTAGAAATAAATTGGACATT

TTTATGGAACATGATTTTTGGCCGGAGGGTATAGCTTTTCGTCGTTTTGTAGATTTTAAATGGCGAACAAATGGCAGACA

TAACAAATTGAATTTAAACTTAAATAATCATAATGAATGTAGCCAAGTTAATTAGTTTTAATTGTAAAGGAGTGAGCCGC

TCGAAAGAATGTGTCAGGCGAATTTGTAACTCAGCGGATGTCATAGCCTTGCAAGAGACTTGGCTCCTACCGCATGATAT

CCCTTGCTTGGGTAGTATAGACGACAGTTTTGCATACACCGGCAAGTCAGCAGTGGATACTTCGGCCGGTATACTGAGAG

GCAGGCCATATGGGGGTGTGGCTTTACTTTGGAAGAAGGACGTGTTCCCAATAGCCACAGTCATCGAGTGTACCAGTGTG

CGTTTATGTGCGGTGAAATTAGGCATAAATAATCGCTCTTTACTAGTATTTTCTGTATACATGCCTACAAACTCTATTGA

TAATTTATTAGAATTCACCGAAACTTTGTGCGAAATTAATGCAATTATCGAAAACAACAGTGTAGAGTCAGTGTACATGT

TGGGGGACTTTAATGCCCACCCGGGTGAGCTATTTTATAATGAACTAAGTAGTTTTTGCATTGAACAGAGGTGGTCATGC

GCGGATATAGAACTATTGCCACCAGAGTCGTATACGTTTGTCAGCGACGCGCACGGTTCGCGAAGATGGCTCGACCACTG

CGTGCTGACGAGTGCCGCGCGGCTCTCTGTGTTAAATATTTCAATTTTGTATGATATTTATTGGTCAGACCATTACCCTG

TCCTTATGGAGTGCAATTTAGATTTAGTTAAAGCCAAAGTTGTTTCAATTAGTTATAGTAAAAACAAGGTTATATGGGGA

GACAGGGACAGTAGTCAAATTAAGAAATATATTAATATATGTAACTCACAGCTTAAGTTTATTGATTTTCCTGAGCAATG

TAGTGAATGTGCAGATAGATTTTGTGATAACGTTAACCATAGAGTAATATTAGATAAAATGTACAATGATTTAGTTACAG

TATTGACAGAAGCTTCAAAAGCGAGTAAACAATGTTGTAAAATACACAAGCGTAAAAAGTATGTCACAGGATGGAACAAA

CATGTCAGAGATAGTCACGTTATTGCCAGGCGTTGTTACATAGATTGGTTAGCTAGTGGGAAACCGACTTCGGGAGAAAA

ATATAATGATATGTGTAATTCTAGAATTATATTTAAACAGAAATTAAAATGGTGTCAAAATAATGAACAACAGATTAAAA

TGGATAAAATTGCAGATCAACATAACAAGAAAGATTTTAAAAATTTTTGGAAGAATACAAATAGCTTAAATCCGAAGCCT

GGCCATCCCGTGAGTGTTTCTGGCATTCATGAACCAACTGAAATAGCTGAAGCGTTTCGTCTTCAGTTAAAGATAGACTC

CTTGTCTCCTACAGACACTCACAAGGACGTAAAGAGATGTAAAAGTGCGGAGTCGGATGTCGCTGTCAGGTTCACTAATA

AGCAGGTGAAGAATGTTATCCGAGGTATGGTCAAAGGCAAGTCACCAGGTCATGATGGTCTAAGTATAGAACATCTAAAG

TACGCAGGAGCTCATTTACCGCGGATTTTAACAATGTTTTTTAATCTCTGTATTAGCCATTCATACCTACCAGATCGGTT

AATGCATACAGTTGTAATTCCCATAATTAAAAACAGGAGTGGAGATGCCTCGGATATATCAAATTACAGGCCGATATCTT

TAGCCACAATAGTGGCCAAAGTATTGGACAGTCTGCTTGACCAGCACTTGAATAAACATATCAACCTCCACGACCAACAA

TTTGGCTTCCGGACAGGTTTGTCAACAGAGAGTGCTATCATGTGCCTCAAGGAGACTGTTCAGTACTACGTGACCAGAAA

GACGCCGGTGTATGCTTGCTTTTTGGACCTGTCAAGGGCGTTCGACCTCGTTTCTTACAGAAAGTTATGGGAAAAGTTGG

AAGATGAAACGTCTTGTAACCAGGAGGTTGTGTCCTTATTGAAACATTGGTACAACAATCAGACTAATGTAGTGAAATGG

GCTGGGGCGTCATCAGCAGTGTACAGGTTGAATTGTGGAGTGAGACAGGGTGGCCTGACCTCACCCAGACTCTTCAACCT

GTATATGAACGGACTTATTGGTGAGCTCAACAGTACCGGCGTCGGATGTCACATAGACGGTGTTTGTGTTAACAACATAA

GCTACGCAGACGACATGGTACTGTTGAGCCCTTCAATGGCAGCGTTAAAAAAGTTAGTTAGAATATGTGAAGTGTATGCG

GAGGCTCATGGACTGAAATACAATTCGAGAAAGAGTGAGATTATGATATTTAAGGCTAGCAACAAAAGTTATGCAACAGT

TCCTGTCACCTTAAGTGGTAGCGAGTTGAAGCAAGTCTCGAATTTCAAGTATCTCGGTCACTGGGTCTCCGAGGACTTGA

AGGATGATAAAGACTTGGAGAGGGAACGTAGGGCGCTGGCGGTCAGAAGCAATATGTTGGCCCGCAGGTTTCGGCGATGC

AGTGATTCGGTAAAAGTGACTTTGTTTAAAGCCTATTGCCAGTCATTGTATACGTGTTCTCTGTGGGTAAACTATACGCG

GATGACGTATAACGCCCTGCGTGTACAATACAACAATACATTTAGGACGCTGCTCAGGTTGCCGCGGTTCTGCAGTGCCT

CGGGCATGTTTGCTGACGCGCACACTGATGGTTTTAATGCCATCATCAGAAAACGCAGTGCTTCACTTCTTCACCGAGTG

CGGACTAGCACCAATAGCGTCCTAAACGTATTGACTGATAGGTGGGACTCTCAGTTGCTAAGACACTGGTTAAGCCTTCA

CACTGTGTTGTAATTTGTATTTTATTTTATTTCTTTGATGTTTTGTGTTACCTACTTTTTATATGGACCTGGTTTTATTA

GTGGCCCTACAATTTGGGCTGGAATGGCCGGCAGGGTAAACCATAA

>TRINITY_DN14417_c2_g2_i9_Proto2-8_CS1_pol#LINE/Proto21280-4168[Capitellasp.1]

TTTTTTTTTTCTATAAAATATTTTTATTTGAGCGGTATACGATCCGCGATAATTTCTCGATAACTACAAAATTCCTAACT

AAAGGAATGTACAGGTTACACACAACTTTGCGTCCGACACATCATACATATATTACAGGCTGTATGTAACTGACAATGAA

TCACATAATATATGAAAGAAATACATAGTCAGTTAAGTACAGCTGAGACCAGAAAAAATATATTGACTCTTCAAAATGCA

ACATTTTAAAACTTAAGATCGCGGTACATTTTCTTTGACCAAAGAGTTTTTAAGTCTTCAAGAGTACTGTGTTACCGAGT

AAAATGGGTTTGCAAAAATCAGATAAACAGTCGCTCATTGTGAACTATAGTTAGGTAAATCTATTGAATTTGGCGACAAC

GGAAAAAACCTTCATATAATTTGGTCGAGATCACATTTTGTTTAGTCTATATTTTTCCTGGTCCATAACATATTCTAAAT

ACACTTATTACAACAAGGATCTTGGTACTTGGGCGTATTAACCCCCTAACAAGAGATATATTTATACATACATTAAATGT

AACCTAAAAATTAAATTAAAAACTAGAAAATGCTATTGTGTTACCCTAAAGTGTCCTTTCCTACATATTTACGCGTAATG

CGTAAAAATAAAGATTAAATTAATTAATTAAATAACTGTTATAATTAAATCGTAAATCCCGCGTAAATTATTAATTGAAA

ACCATAATGGCGTCGCCATTCCACTCATGAAATCAAACTATTATATGCAAAGGTCTTAATTTAAACAGCCACGTGAGACG

TCGCATTTTTGAGATCGGACGTATTGATCGAAATCAAAAATGTCGACGGTGGTAAAGTGTAATAACTGCAATGTTGTTAT

TAATGAAGTCCTTGCATTTATTTGCAACAAGATTGATGTGATGGATGAAGAGAGCATTAGTCGAATATGTATTAGTGCAT

TCTCGGAATACGAAGTGAAAAATGCAAAAGATTTGCTATACGAGTCAATTTCGACAACAAGGCGCAAAAAGACAAGAAAA

CGTGATGGTAAAACTCTCCGTGAGATTGATGATATTATTTGTTTGCTCAAAGAAACGGATCCCGAAGAAATACCAATTTT

TGTGGCACGGGATTTGCAAAAGTTGCCACCAGTACTGTTCGACCACGTAGATGTTACGCGGCTTCTCAAAGACTTATTAA

AAATGCAAGGTGATATTAAGCAAATCAAGGAACAATATGTGACTGTGGAACAGATACAACCGTTGAAATCTGAGTTTGAA

TTATTAAAAAACGAGGTAACAGCAACGAGAACATCAATTTATGTTGAGGGTAGTAAGAATACATCATCCCAGCAGAACAT

CGAGACCGCGGATATTACGATTGTGAATCATGAAGAGGGTAAAAAATGTAAACAACGAAATATGCGCGGGAATCGGGCGT

TGCACCCACCTCAGCCGAGCAGCGCGAGCGCGTCGGCCGTGGCGTCGGCGTACATGAGACCCGAAGATATGCCGAACAAA

CACGATCGCGAGACGAACGCGCCCGCTATGCAATTTGTCGTCCCCGAGTCTCAAGATAAAACGGCTCAGAGTCACGAACC

CAACCCGAACATACACGAGCGGCAGCCGAGCGCGTCGCAAGTCGCAGCGGGACCTCAAGGTCAAGCGTCGAACTGTAACG

CGGTGAAGCCGAAAATTCACGAGCGAGAGCCGAACCGAGCCGAGCAGGAGTCGCATGGACATACGGTCGCGATGAATGTA

CAATTGCATCAGAAATCATTTGCCGATGTTGCACAAGACGGAACATGGAAATCTAAAACTGTGCCTGAAGAATGGATACA

GGTACAGAGAAAAAGATACCGGAATAGATTTGTGGGGAACAGAGGAAAAGCAATTGTTACGCCGGATTCTAATTTCAAGG

CGGCAGAAATAAAAATTCCGATATATATTTATAATGTTGCGAAGGCTGTAACCGTGTGTAGCATAACCGAGTATATTCAA

AATAAGGCGAATATTGAAGTTACCATGGAAAAAATGAATATGACAGTTGCAAAAGATTATGATTCATACAAAATATTTAT

ACCTAGAAATAAATTGGACATTTTTATGGAACATGATTTTTGGCCGGAGGGTATAGCTTTTCGTCGTTTTGTAGATTTTA

AATGGCGAACAAATGGCAGACATAACAAATTGAATTTAAACTTAAATAATCATAATGAATGTAGCCAAGTTAATTAGTTT

TAATTGTAAAGGAGTGAGCCGCTCGAAAGAATGTGTCAGGCGAATTTGTAACTCAGCGGATGTCATAGCCTTGCAAGAGA

CTTGGCTCCTACCGCATGATATCCCTTGCTTGGGTAGTATAGACGACAGTTTTGCATACACCGGCAAGTCAGCAGTGGAT

ACTTCGGCCGGTATACTGAGAGGCAGGCCATATGGGGGTGTGGCTTTACTTTGGAAGAAGGACGTGTTCCCAATAGCCAC

AGTCATCGAGTGTACCAGTGTGCGTTTATGTGCGGTGAAATTAGGCATAAATAATCGCTCTTTACTAGTATTTTCTGTAT

ACATGCCTACAAACTCTATTGATAATTTATTAGAATTCACCGAAACTTTGTGCGAAATTAATGCAATTATCGAAAACAAC

AGTGTAGAGTCAGTGTACATGTTGGGGGACTTTAATGCCCACCCGGGTGAGCTATTTTATAATGAACTAAGTAGTTTTTG

CATTGAACAGAGGTGGTCATGCGCGGATATAGAACTATTGCCACCAGAGTCGTATACGTTTGTCAGCGACGCGCACGGTT

CGCGAAGATGGCTCGACCACTGCGTGCTGACGAGTGCCGCGCGGCTCTCTGTGTTAAATATTTCAATTTTGTATGATATT

TATTGGTCAGACCATTACCCTGTCCTTATGGAGTGCAATTTAGATTTAGTTAAAGCCAAAGTTGTTTCAATTAGTTATAG

TAAAAACAAGGTTATATGGGGAGACAGGGACAGTAGTCAAATTAAGAAATATATTAATATATGTAACTCACAGCTTAAGT

TTATTGATTTTCCTGAGCAATGTAGTGAATGTGCAGATAGATTTTGTGATAACGTTAACCATAGAGTAATATTAGATAAA

ATGTACAATGATTTAGTTACAGTATTGACAGAAGCTTCAAAAGCGAGTAAACAATGTTGTAAAATACACAAGCGTAAAAA

GTATGTCACAGGATGGAACAAACATGTCAGAGATAGTCACGTTATTGCCAGGCGTTGTTACATAGATTGGTTAGCTAGTG

GGAAACCGACTTCGGGAGAAAAATATAATGATATGTGTAATTCTAGAATTATATTTAAACAGAAATTAAAATGGTGTCAA

AATAATGAACAACAGATTAAAATGGATAAAATTGCAGATCAACATAACAAGAAAGATTTTAAAAATTTTTGGAAGAATAC

AAATAGCTTAAATCCGAAGCCTGGCCATCCCGTGAGTGTTTCTGGCATTCATGAACCAACTGAAATAGCTGAAGCGTTTC

GTCTTCAGTTAAAGATAGACTCCTTGTCTCCTACAGACACTCACAAGGACGTAAAGAGATGTAAAAGTGCGGAGTCGGAT

GTCGCTGTCAGGTTCACTAATAAGCAGGTGAAGAATGTTATCCGAGGTATGGTCAAAGGCAAGTCACCAGGTCATGATGG

TCTAAGTATAGAACATCTAAAGTACGCAGGAGCTCATTTACCGCGGATTTTAACAATGTTTTTTAATCTCTGTATTAGCC

ATTCATACCTACCAGATCGGTTAATGCATACAGTTGTAATTCCCATAATTAAAAACAGGAGTGGAGATGCCTCGGATATA

TCAAATTACAGGCCGATATCTTTAGCCACAATAGTGGCCAAAGTATTGGACAGTCTGCTTGACCAGCACTTGAATAAACA

TATCAACCTCCACGACCAACAATTTGGCTTCCGGACAGGTTTGTCAACAGAGAGTGCTATCATGTGCCTCAAGGAGACTG

TTCAGTACTACGTGACCAGAAAGACGCCGGTGTATGCTTGCTTTTTGGACCTGTCAAGGGCGTTCGACCTCGTTTCTTAC

AGAAAGTTATGGGAAAAGTTGGAAGATGAAACGTCTTGTAACCAGGAGGTTGTGTCCTTATTGAAACATTGGTACAACAA

TCAGACTAATGTAGTGAAATGGGCTGGGGCGTCATCAGCAGTGTACAGGTTGAATTGTGGAGTGAGACAGGGTGGCCTGA

CCTCACCCAGACTCTTCAACCTGTATATGAACGGACTTATTGGTGAGCTCAACAGTACCGGCGTCGGATGTCACATAGAC

GGTGTTTGTGTTAACAACATAAGCTACGCAGACGACATGGTACTGTTGAGCCCTTCAATGGCAGCGTTAAAAAAGTTAGT

TAGAATATGTGAAGTGTATGCGGAGGCTCATGGACTGAAATACAATTCGAGAAAGAGTGAGATTATGATATTTAAGGCTA

GCAACAAAAGTTATGCAACAGTTCCTGTCACCTTAAGTGGTAGCGAGTTGAAGCAAGTCTCGAATTTCAAGTATCTCGGT

CACTGGGTCTCCGAGGACTTGAAGGATGATAAAGACTTGGAGAGGGAACGTAGGGCGCTGGCGGTCAGAAGCAATATGTT

GGCCCGCAGGTTTCGGCGATGCAGTGATTCGGTAAAAGTGACTTTGTTTAAAGCCTATTGCCAGTCATTGTATACGTGTT

CTCTGTGGGTAAACTATACGCGGATGACGTATAACGCCCTGCGTGTACAATACAACAATACATTTAGGACGCTGCTCAGG

TTGCCGCGGTTCTGCAGTGCCTCGGGCATGTTTGCTGACGCGCACACTGATGGTTTTAATGCCATCATCAGAAAACGCAG

TGCTTCACTTCTTCACCGAGTGCGGACTAGCACCAATAGCGTCCTAAACGTATTGACTGATAGGTGGGACTCTCAGTTGC

TAAGACACTGGTTAAGCCTTCACACTGTGTTGTAATTTGTATTTTATTTTATTTCTTTGATGTTTTGTGTTACCTACTTT

TTATATGGACCTGGTCTGAAATAAAGATTTATTATTATTATTATTATTATTATTGTCTCCGTGCTCGTTAGTCGTATCCT

TCATTTTATAAGTCTGATTT

>TRINITY_DN14411_c0_g3_i1_MAG_pol#LTR/Gypsy1045-4368[Bombyxmori]

TAATTAAGGGTGGAGGTGTTACATATGATTATAAATATTTGTGTTGTCTGTGGTTAAGTGAGGAGCGACGCGCCAGATTT

GGAATGGCGGCAATTGAAAATAGAAGCTTGAACTATGCAAACGTCTATTATTTATCAGTGGCGACGAGGATAACGGAACG

ATATTCGGTGCAGAATAAAGTCTTAAATTATTGTGGTATAGTGCTTTGGGGGTGATTAAAAATGTCTGTTGGGAAAGTTC

GAGATTTTGACATAAAAAATGGGAATTGGTCGGCGTACGTCGATCGTCTCGAAATGTATTTTGTTGCTAACAAAATAGCG

GAAGATTTGAAGTTACCAACATTAATAGCTCTTATCGGCGAACCGGCTTATGAGTTACTGTCTACTTTGGCGAGCCCGAG

AAAACCATCAACCCTGAAATATAAAGAAGCGGTGGAATTACTGCAAGCACATCTACAGCCGAAACCATCAATTCTCGCGG

AGAGGTATAGGTTCCGACAAAGACGCCAATCAGCGGGAGAAACGATAGCTGATTACGTGGCAGATTTGAAAAAGATGTCA

CGTTATTGTGAATTCAAGATCAATTTAGAAGAAAATTTGAGGGATCAGTTTGTGTGCGGGTTACGTAGCGAATATATACG

TCAGAGGTTATTCGCGGAAGATAACATAGATTATCAAAAAGCGCTAGTATTAGCCAATACTCTGGAGGCAGCTGAGCGGG

ACGCCGGGGCCGTAGAGGGAACGCAGGAACAAACTACCAGCCGGGAACTAAGCGAGAGAATACACAAGTTGGAACTCAAT

AAATGTTCAGCCTGCGGGGTCAACGGTCATGGGGCACATAACTGTAGGTACAAGGAGTTCGAATGTAGTTATTGTGGACA

ACCTGGCCATTTACGAAGAGTTTGTAGAAAAAAAGAATTTGATCGTCGCATAAAATCTACGATTTCAAACCGCGGGAATA

ATGGCGCGCGAGGAAGGTCACGTAGAGGACGCGTGATCGGGTCGAGCGCCTACGGAGCGGGTGTATGGCGGAGCGGCAAC

ATGGCGCGCGGGGGGCGCAGCAACGCTCGCACCGCACGCAGCAACGACGTTACCGACGCGGCGTACTGGCTGAGCGAGCC

AGCGATGGATGACAACTCCGGCTCGGAGCAAGACGTTGAGGGTACAAACGAGGAACCGATGTATCAAATGTCACTAACCA

ACTATAAACCGGTGTGTATAAAGCTTCTAATTAATAATAAAACCTTATCGATGGAGATTGATACAGGCTCTGCGTTATCC

TGTATAAGCAAGTCTACGTATAAGGAGCATTTTAAAGAATTACCTTTGAAACCATGTAAAATGAAAGTAAAATTTTATGA

TGGCTCTGTCATTCAACCAATAGGGTATTTAGAGACTGATGTGCAATATATGAAAAATATGAAGAAACTAGATCTCTATG

TGATCGATAAGGGTACTACTAATTTACTAGGTAGACAATGGTTATCGGAATTGAATATTGAGATACCAAAATTTACTAGA

TGTAACCACATTAAATTATGTAAAAATAAAATGTTTAACGATATCATTTCCAGACATGACAACCTATTCGATGGCACGTT

GGGCAAATATACCGGTGAAGAAGTCAAGTTATGCGTGCGGGATGGGACCGAGCCAGTATTTTGTCGAGCGCGCCCCGTGC

CTTATGCATTGCTGGCGCGGGTCAATGCTGAGTTAGACGCGATGCTGCGCGCCGGCGTCATCGAGCCCGTTGAACGGTCC

GACTGGGCCACACCGCTAGTTATAGCGAGGAAGGGAGACGGTGGAATACGCTTATGCGCGGATTATAAGGTGACACTTAA

CAAGGCTTTACTGGTTGACAGGTACCCGGTCCCGAAGGTAGAGGACTTATTTAGTAATTTGAGTGGCAATCATTATTTTA

CTAAGCTAGATTTGTCACAGGCTTACAATCAATTAATTTTGGATGAAACTTCTCGGAACTACACAGTAATTAATACACAC

AGAGGACTTTTTAAATACAATCGCCTCGTTTATGGTCTCTCTTCCAGTCCGGGCATTTTTCAAAAGTTCATGATGAATCT

CTTTAAAAACGTGCAGGACGTAGTCATATTCTATGATGATATTTTAATAAAGAATAAGTCATTAGAATCTCATTTAGAAA

GAGTTGAACAGGTTTTTAGTATTTTAGAGAGGAATGGGTTGAAAATAAAAAAAGAAAAGTGTGAGTTTATGGTAGGCCAA

GTAAAATATTTAGGATTCATAATTGATAAACATGGCGTACGGGTAGATGCAAATAAAATAAAACCGATCCTATCTATGCC

CGACCCAACTAACGTCTCAGAGTTGAAGTCTTTTCTCGGTATGGTTAATTTCTATGGAAAATTCATTAAGAATTTGAGTA

CTCATATTACACCACTTTACGAATTACTTAAGAAAGGTAAACATTGGCAATGGACTAAAACACATAGATATGTGTTCAAT

AAAATTAAACAACTTCTATGTAGTACTGAAGTATTGACACACTTTGACATATCGCTGGAGAGCATAGTAACATGCGACGC

GAGCGCACGCGGGCTGGGGGCCGTGCTGGCACAGCGCGCGTCTGACGGCAGCGAGCGGGTTGTGGCGTACGCATCGCGGG

CGCTCACTGCCGCGGAGTTACACTACAGCCAGATACATAAAGAAGCCTTGGCTATAATTTTCGCGGTAGACAAGTTTCAT

CAGTATCTTTATGGCAGAAAGTTTACACTACGTACAGATCATAAACCGCTGGTCACAATTTTTGGGCCCCATGCAGGAAT

ACCGAACACGGCGGCGAGTCGTTTACAGCGGTGGGCCATTAAACTATCAGCTTACGATTTTAATATAGAGTACATTCGAT

CAGATAAAAACACGGCAGATATTTTGTCTAGGTTAATTAGCACTCATAAAGAAGGGGTTATTAGTGAAGAATTAGACACA

CCTGAACAAACGTATTTACACTTTGCCGCGGAAGCATTGTTATTAGATTATCAAACGTTAAAAAAAGAAACCGTTTCGGA

TAGTGTATTGAGCAGAGTAGCCAGATATATAAACGATGGCTGGCCGGTCGAGGTCGAAATGAAAGAATTAAAACCATATT

TTAACCGTAGAAAGGAATTATATATTGAGCTAGGGTGTGTAATGTGGGGACATAGGCTAGTAATTCCTAGTACGTGTAGG

AATAAAGTGATCGCTGAGCTTCATGAGAGTCACATGGGCATAGTCAAAACTAAATCGTTAGCGCGAAGTTACGTGTGGTG

GCCGGGAATAGGCGAGGCACTAGAGGCAGCGTGTTGCAGCTGCACCGTGTGCGCTGAAGTGGCGGACGCACCACCTGCGC

ATGCGCCCCGCGCCTGGCCGTGGCCAGATCGACCGTGGACCGGGATACACGTAGACTTCTTAGGCCCGGTAGCCGGTCTT

ACTTATCTAGTGGTAGTCGATGCACATTCAAAGTGGATTGAAGCTATTAGAATGCTGAGCACCACATCGCAAGCGGTAAA

AGAATGACGAGAGAATTAAAGCTCGCCAAGAGAGTGTAGAAGCTACAAGTGGCGTCACAAGAAAGTTTAATCCGGGGGAC

CCTGTATGGTTCAGAGAGTACCGTAGCTCGAATAAGTGGTCTGCAGGGACTGTTATAGAGAAAACAGGTAGTACAGATTA

TAATGTTAAATCTATCCACGGCACTGAG

>TRINITY_DN14411_c0_g2_i3_HAT-22_SM#DNA/hAT-Tip1001217-3385[Schmidteamediterranea]

AAATATTTTTGCAATACGGAATCCTAAAAAGTAGGATCGCGTTTCATTTCCAAAAGCAGGTTTTTTTCTATTGACATAAT

GGAAAGATCTGTAAGTCTTTCCTGGCCTTGGGTGTTTCTTAAATAGGTTTGAATACGTTTTAGCGACGAAAATGACCTTT

CAGTTGAAGCACTGCTCGCGGGCACCGTAACAATTAATTTTGACAAACGCAGTACTTCGGGAAAAATTGTACTTAAGCCG

GTTGTGTAAAAATAATCTATAATTTGGCAAATATTCATATTTTTTAATTCGCTGTTTTTATAAACACATTTGAGCTCGTT

CATGAGGCGTTCTAAGTTAAAGTTAAAAAATTTGTAGAAATTGCGTTACTAAATCTATCGGAAAATTATAATTATATTTT

TCAAAGTTTTCGCACGACAATAATTCAAAATATTTTAGATCTTTTAGATTTTTATAACGCTCGCGACATTCGTTTGATAT

ATTATCTACTATTTCAATAAAAACCCGCTTAAAAAATGATTTTGTATCTACTACATTACGTTCTTGTCTTGGCGGTCCTG

TTACCCTTTTAACGCGTTCAAAAATTATGTCGAACTTAGATCTGAATTCCTCTAAAAACGATTCAAATTCTTTTATTTTT

TTCACGCAATAAACTACATCCGATGTCTTTTTTTGAATAATATCGTAAAGAATATCGGTTTCCGCGAATATTTCATTAAA

CACGTTAAGGAAAAACTGAAATTGAAAATCTTTTAAAAAAATTAAAAATGACGAAACTTGACTTAAGGTTTCGCCATCCC

ACGAACTGACATTTTCTTCGATATCCTCAAATAAAGTAATTAAAACATTTAAGGAATTATAAATGACGTTTACAGTACGC

GAATTATAAGTCCACCGAGTTGGAGCAACGCGAGGTAATTTTTTAATATCAGCAAATTTATTATAGTGACTTTGCCTTTT

AGTACTATTATTAAAGAAATTGGCTAATATTTTTATATCGTTAAAAAAACTTTTTGTTTCGGGAATACGGTTACACGATT

TTGAAAGTACTAAATTAAGAACGTGAGCGGAACACCAGACGTATGCAGCGAGAGGGCATTCCTGGCGAATCAACGACTGA

ACGCCGTTGAGTTCAGATGACAATACAGCGGCGCCGTCATAGGTCTGAGCGATTAATTTTGAAGAACACTTAAATTCTGA

TAAAATCTGCAGAATGACAGTACTTAAAGTTTGCGCGGTGCGGTCGGAACTAACGTCAATGTATTTAATGAAACGATCAT

TAACTTCCGACTTATAAAAATATCTAAAAACAATAACTAGTTGCTCTTTACCGCTCACGTCTGGTGTTTCGTCAACCATC

ACGGCAACGAAATCAGTTTCGGCGATTTGTTGTTTTATTTTTTCTATCATTAATGACGCTACACTAGAGATAAGGTCATT

TTGAATTCGATTTGACAAACCCGTAAATGAGATTGTGCTATTTTGTAAGTGATTTTCCAAAACAACGTCATAAGCCGCGA

GCAAGTTGACCATCTCTAAATAATTTCCTTGATTCGACGACGAAGCCGATTCATCATGTCCGCGAAACGCAAGTTCTTGT

TTACCTAAAAAGCAAACAATGTCTATAAGTCGCCGAAAAATATTTCGATTCCGATCAACTTTTGCATTATGCCTATCGAT

ATCGATTTTAATTTGTGAATCTAAACACGTCTCTATACGATTTTTACCAAAGCCGTGATATTTCATACAAGATTCTAAAT

GGTTTTCCGAGAGCTCGTGTTTTTTAGCCGCGCTGAGAAAATTATGAATGTCCGAGTAACCGGTGTTATTCCAGACGGTT

TTGTTATTTGAAAATAATAAACAAGGAAAACAGAACAATCGGTTACTATGAATACAACCCGTTAGCCAAATAAATGAATT

ATATTGTTTTATATTAAATTTACGATTAACTGTACCTTTCGTTTGTATCAAATTCAAAAAAGGCGTACTGCGGCCTTTCG

CGATCACTTGTTTTTTGTCACCGAAAGATAGTTTTAAAAACGCACCAGTTTTTATTTTCTGTACACACACACCACTAGAA

CAACTTTCACAGTTAAAAACTTCCATTATTTATAATAATTATACAATTATCAACGATAAAATCTCAAAGAGATGCGTTTA

CGAAACGAATCAAACAAACAATAATAATGGCGTCCAGGGTTACCCAATTTGAAAAAATACCCATATATTTTACTTAATAT

TACCCATTTACCCCAAAAAAGTTGAAAAAGAAAAACCCGAATAATCGCAAGGTTTACTTGTTCATTTGATAAAAGTAAAG

TATTTAGAATAAAACAAAGAAACTTTATTATTTTGGTTAAAATCAATGAATAAAAAATCTGACGTCGTTGAAAAATGACA

AAGAATATTTTTTAAATGAATAACAATGAAACACATTGGAGAAAAACTATAGTCGTAATTTTTAAATGCTAAAAATATAA

TGTAAAAATTCCATACTCCTTCTTAATTACCCACCTTAGAGGGTAAATCTTTTCAGTTGGGTGGTAACACTGATTTACGA

CAAAATTTCGCGGTTGTAATAAAAAGTCACTGCCTACAGTTGTGTTAGTGTGCGTAGTGTTGACGTAGTGTGTACAAATG

ATTACATCAACACACACAACAGTGTTTTTGTATGACTACGAGACTTTCACACGCACACATAAACACTCCACTGAATCAGT

TAGACATTTAGTTTAAGTGTCTATACGCTTGGTGGCGCTAGCGCAGCGTTGTTAAGAATGCAAAAATGTTCGTAGTAGTG

TACTAGTATAAATGACAGATGGCGTTGTTTTAAGTTTTAAACACTTCACTTATTTAATTGTTCTATAATTAGATTAATAT

ATGTTATGTAAAAATAATTATTAATATTTTAAATAAAATATTATTGTGAATGTCTGTCTTAGATTCTCAGGGTAGGCAGT

GTCTTTTTGTCTCTATGGACTGCACGCCACTGCTGGCCGGTCGAGGTAGAAATGAAAGAATTAAAACCATA

>TRINITY_DN14411_c0_g3_i4_MAG_pol#LTR/Gypsy1045-4368[Bombyxmori]

TAATTAAGGGTGGAGGTGTTACATATGATTATAAATATTTGTGTTGTCTGTGGTTAAGTGAGGAGCGACGCGCCAGATTT

GGAATGGCGGCAATTGAAAATAGAAGCTTGAACTATGCAAACGTCTATTATTTATCAGTGGCGACGAGGATAACGGAACG

ATATTCGGTGCAGAATAAAGTCTTAAATTATTGTGGTATAGTGCTTTGGGGGTGATTAAAAATGTCTGTTGGGAAAGTTC

GAGATTTTGACATAAAAAATGGGAATTGGTCGGCGTACGTCGATCGTCTCGAAATGTATTTTGTTGCTAACAAAATAGCG

GAAGATTTGAAGTTACCAACATTAATAGCTCTTATCGGCGAACCGGCTTATGAGTTACTGTCTACTTTGGCGAGCCCGAG

AAAACCATCAACCCTGAAATATAAAGAAGCGGTGGAATTACTGCAAGCACATCTACAGCCGAAACCATCAATTCTCGCGG

AGAGGTATAGGTTCCGACAAAGACGCCAATCAGCGGGAGAAACGATAGCTGATTACGTGGCAGATTTGAAAAAGATGTCA

CGTTATTGTGAATTCAAGATCAATTTAGAAGAAAATTTGAGGGATCAGTTTGTGTGCGGGTTACGTAGCGAATATATACG

TCAGAGGTTATTCGCGGAAGATAACATAGATTATCAAAAAGCGCTAGTATTAGCCAATACTCTGGAGGCAGCTGAGCGGG

ACGCCGGGGCCGTAGAGGGAACGCAGGAACAAACTACCAGCCGGGAACTAAGCGAGAGAATACACAAGTTGGAACTCAAT

AAATGTTCAGCCTGCGGGGTCAACGGTCATGGGGCACATAACTGTAGGTACAAGGAGTTCGAATGTAGTTATTGTGGACA

ACCTGGCCATTTACGAAGAGTTTGTAGAAAAAAAGAATTTGATCGTCGCATAAAATCTACGATTTCAAACCGCGGGAATA

ATGGCGCGCGAGGAAGGTCACGTAGAGGACGCGTGATCGGGTCGAGCGCCTACGGAGCGGGTGTATGGCGGAGCGGCAAC

ATGGCGCGCGGGGGGCGCAGCAACGCTCGCACCGCACGCAGCAACGACGTTACCGACGCGGCGTACTGGCTGAGCGAGCC

AGCGATGGATGACAACTCCGGCTCGGAGCAAGACGTTGAGGGTACAAACGAGGAACCGATGTATCAAATGTCACTAACCA

ACTATAAACCGGTGTGTATAAAGCTTCTAATTAATAATAAAACCTTATCGATGGAGATTGATACAGGCTCTGCGTTATCC

TGTATAAGCAAGTCTACGTATAAGGAGCATTTTAAAGAATTACCTTTGAAACCATGTAAAATGAAAGTAAAATTTTATGA

TGGCTCTGTCATTCAACCAATAGGGTATTTAGAGACTGATGTGCAATATATGAAAAATATGAAGAAACTAGATCTCTATG

TGATCGATAAGGGTACTACTAATTTACTAGGTAGACAATGGTTATCGGAATTGAATATTGAGATACCAAAATTTACTAGA

TGTAACCACATTAAATTATGTAAAAATAAAATGTTTAACGATATCATTTCCAGACATGACAACCTATTCGATGGCACGTT

GGGCAAATATACCGGTGAAGAAGTCAAGTTATGCGTGCGGGATGGGACCGAGCCAGTATTTTGTCGAGCGCGCCCCGTGC

CTTATGCATTGCTGGCGCGGGTCAATGCTGAGTTAGACGCGATGCTGCGCGCCGGCGTCATCGAGCCCGTTGAACGGTCC

GACTGGGCCACACCGCTAGTTATAGCGAGGAAGGGAGACGGTGGAATACGCTTATGCGCGGATTATAAGGTGACACTTAA

CAAGGCTTTACTGGTTGACAGGTACCCGGTCCCGAAGGTAGAGGACTTATTTAGTAATTTGAGTGGCAATCATTATTTTA

CTAAGCTAGATTTGTCACAGGCTTACAATCAATTAATTTTGGATGAAACTTCTCGGAACTACACAGTAATTAATACACAC

AGAGGACTTTTTAAATACAATCGCCTCGTTTATGGTCTCTCTTCCAGTCCGGGCATTTTTCAAAAGTTCATGATGAATCT

CTTTAAAAACGTGCAGGACGTAGTCATATTCTATGATGATATTTTAATAAAGAATAAGTCATTAGAATCTCATTTAGAAA

GAGTTGAACAGGTTTTTAGTATTTTAGAGAGGAATGGGTTGAAAATAAAAAAAGAAAAGTGTGAGTTTATGGTAGGCCAA

GTAAAATATTTAGGATTCATAATTGATAAACATGGCGTACGGGTAGATGCAAATAAAATAAAACCGATCCTATCTATGCC

CGACCCAACTAACGTCTCAGAGTTGAAGTCTTTTCTCGGTATGGTTAATTTCTATGGAAAATTCATTAAGAATTTGAGTA

CTCATATTACACCACTTTACGAATTACTTAAGAAAGGTAAACATTGGCAATGGACTAAAACACATAGATATGTGTTCAAT

AAAATTAAACAACTTCTATGTAGTACTGAAGTATTGACACACTTTGACATATCGCTGGAGAGCATAGTAACATGCGACGC

GAGCGCACGCGGGCTGGGGGCCGTGCTGGCACAGCGCGCGTCTGACGGCAGCGAGCGGGTTGTGGCGTACGCATCGCGGG

CGCTCACTGCCGCGGAGTTACACTACAGCCAGATACATAAAGAAGCCTTGGCTATAATTTTCGCGGTAGACAAGTTTCAT

CAGTATCTTTATGGCAGAAAGTTTACACTACGTACAGATCATAAACCGCTGGTCACAATTTTTGGGCCCCATGCAGGAAT

ACCGAACACGGCGGCGAGTCGTTTACAGCGGTGGGCCATTAAACTATCAGCTTACGATTTTAATATAGAGTACATTCGAT

CAGATAAAAACACGGCAGATATTTTGTCTAGGTTAATTAGCACTCATAAAGAAGGGGTTATTAGTGAAGAATTAGACACA

CCTGAACAAACGTATTTACACTTTGCCGCGGAAGCATTGTTATTAGATTATCAAACGTTAAAAAAAGAAACCGTTTCGGA

TAGTGTATTGAGCAGAGTAGCCAGATATATAAACGATGGCTGGCCGGTCGAGGTCGAAATGAAAGAATTAAAACCATATT

TTAACCGTAGAAAGGAATTATATATTGAGCTAGGGTGTGTAATGTGGGGACATAGGCTAGTAATTCCTAGTACGTGTAGG

AATAAAGTGATCGCTGAGCTTCATGAGAGTCACATGGGCATAGTCAAAACTAAATCGTTAGCGCGAAGTTACGTATGGTG

GCCGGGAATAGACGAGGCACTAGAGACAGCATGTTGCAGCTGCACCGTGTGCGCTGAAGTGGCGGACGCACCACCTGCGC

ATGCGCCCCGCGCCTGGCCGTGGCCAGATCGACCGTGGACCAGGATACACGTAGACTTCTTAGGACCGGTAGCCGGTCTT

ACTTATCTAGTGGTAGTCGATGCACATTCAAAGTGGATTGAAGCTATTAGAATGCTGAGCACCACATCGCAAGCGGTAAT

AAAAGAATTACGAGAGATGTGGGCGAGATTTGGGTTACCTAAACAATTGGTTAGTGATAATGGCCCTCCGTTTTTCAGTG

GAGAATTTCAGCAATTCTTAAATAATAATGGTATAGAACATATATTTTCGGCACCTTATCACCCAGCTTCTAATGGGGCA

GCGGAAAACGCTGTTAAAATTTGTAAAAAGAGCATTAAAAAGGCGATTAAATCGAAATCCGACGTACACTCCACATTGTG

TCGTTTTCTGCTGGCGTACAGAAACACACCACACTATACAACCGGGGAAAGCCCCGCCAAGATGTTATTAGGGCGCAACT

TGCGCATGCGGTTAGATTGTTTGAAACCAGACCAGAAGGCACTTATTAGAGCTCGCCAAGAGACAGTAGAAGCTACATCG

AGTGGCGTCACAAGACAGTTTAATCCGGGGGACCTTGTATGGTTCAGAGAATACCGTAGCTCAAACAAGTGGTCTGCAGG

GACTGTTATAGAGAAAACAGGTAGTACAGATTATAATGTTAAATCTATCCACGGCACTGAGGTGCATAGACACATTGACC

AACTAAAACCAAGAGTGATAAATAGGACAGCTAGGCTAGTAGATGCAAACTTTAGGAACTTTCAACCAGTTCAGCCTGTT

AAGAGCTCTCGCTCTTCTCTCGTGTTTCCTTCGGACAATAATCGAGCGCCGAACACAGAACCGGACCGGACAAACGGAAC

CAGCTTGACGAAAGCATTGGTCGAGGAAACATCCCGCACGCAAACAGACGGAAGTGATTTGGTTACAAGTGAACCCTTGT

CAACGTCAAGTGTACCTTTAAATATGCCCAAGGTGCAGGAGACGGGTAGTCCTAGGGTTCCTATTACTCGGGATATACGA

ACTAGTAATCGTACACGCAAACCGGTTAAGAGATATGGGTTTGATGATGATTGAAGTAAGAAAATATATTATAAGATTGT

TATGATTATTATTGATACATTGTTTAAATATAATTTAGTAAGTAAGTTTATCTGTAATAATTAAGGGTGGAGGTGTTACA

AATAATTATAAATATATGTGTTATCTGTGGACAAGTCATGGTGATGAGCGGAAATTTGGATTGGCGGTAATTGAAAAATT

TGAAAATATAAACTTGAACTATTCAAACGTCTATTATTTATCAAAAGTATGTATATGCTACTAGTTGGAAGTTTTTAAAC

CCATGCTGAGTTAGATTACAAGAAATATACAAATATTGAGAACAAGGGTCTATGCTATCT

>TRINITY_DN14411_c0_g3_i5_MAG_pol#LTR/Gypsy1045-4368[Bombyxmori]

TAATTAAGGGTGGAGGTGTTACATATGATTATAAATATTTGTGTTGTCTGTGGTTAAGTGAGGAGCGACGCGCCAGATTT

GGAATGGCGGCAATTGAAAATAGAAGCTTGAACTATGCAAACGTCTATTATTTATCAGTGGCGACGAGGATAACGGAACG

ATATTCGGTGCAGAATAAAGTCTTAAATTATTGTGGTATAGTGCTTTGGGGGTGATTAAAAATGTCTGTTGGGAAAGTTC

GAGATTTTGACATAAAAAATGGGAATTGGTCGGCGTACGTCGATCGTCTCGAAATGTATTTTGTTGCTAACAAAATAGCG

GAAGATTTGAAGTTACCAACATTAATAGCTCTTATCGGCGAACCGGCTTATGAGTTACTGTCTACTTTGGCGAGCCCGAG

AAAACCATCAACCCTGAAATATAAAGAAGCGGTGGAATTACTGCAAGCACATCTACAGCCGAAACCATCAATTCTCGCGG

AGAGGTATAGGTTCCGACAAAGACGCCAATCAGCGGGAGAAACGATAGCTGATTACGTGGCAGATTTGAAAAAGATGTCA

CGTTATTGTGAATTCAAGATCAATTTAGAAGAAAATTTGAGGGATCAGTTTGTGTGCGGGTTACGTAGCGAATATATACG

TCAGAGGTTATTCGCGGAAGATAACATAGATTATCAAAAAGCGCTAGTATTAGCCAATACTCTGGAGGCAGCTGAGCGGG

ACGCCGGGGCCGTAGAGGGAACGCAGGAACAAACTACCAGCCGGGAACTAAGCGAGAGAATACACAAGTTGGAACTCAAT

AAATGTTCAGCCTGCGGGGTCAACGGTCATGGGGCACATAACTGTAGGTACAAGGAGTTCGAATGTAGTTATTGTGGACA

ACCTGGCCATTTACGAAGAGTTTGTAGAAAAAAAGAATTTGATCGTCGCATAAAATCTACGATTTCAAACCGCGGGAATA

ATGGCGCGCGAGGAAGGTCACGTAGAGGACGCGTGATCGGGTCGAGCGCCTACGGAGCGGGTGTATGGCGGAGCGGCAAC

ATGGCGCGCGGGGGGCGCAGCAACGCTCGCACCGCACGCAGCAACGACGTTACCGACGCGGCGTACTGGCTGAGCGAGCC

AGCGATGGATGACAACTCCGGCTCGGAGCAAGACGTTGAGGGTACAAACGAGGAACCGATGTATCAAATGTCACTAACCA

ACTATAAACCGACATGACAACCTATTCGATGGCACGTTGGGCAAATATACCGGTGAAGAAGTCAAGTTATGCGTGCGGGA

TGGGACCGAGCCAGTATTTTGTCGAGCGCGCCCCGTGCCTTATGCACTGCTGGCGCGGGTCGATGCTGAGTTAGACGCGA

TGCTGCGCGCCGGCGTCATCGAGCGAGTTGAACGATCCGACTGGGCTACACCGCTAGTTATAGCGATGAAGGGAGACGGT

GGAATACGCTTATGCGCGGATTATAAGGTGACACTTAACAAGGCTTTACTGGTTGACAATTACCCAGTCTAACTACACAG

TAATTAATACACACAGAGGACTTTTTAAATACAATCGCCTCGTTTATGGTCTCTCTTCCAGTCCGGGCATTTTTCAAAAG

TTCATGATGAATCTCTTTAAAAACGTGCAGGACGTAGTCATATTCTATGATGATATTTTAATAAAGAATAAGTCATTAGA

ATCTCATTTAGAAAGAGTTGAACAGGTTTTTAGTATTTTAGAGAGGAATGGGTTGAAAATAAAAAAAGAAAAGTGTGAGT

TTATGGTAGGCCAAGTAAAATATTTAGGATTCATAATTGATAAACATGGCGTACGGGTAGATGCAAATAAAATAAAACCG

ATCCTATCTATGCCCGACCCAACTAACGTCTCAGAGTTGAAGTCTTTTCTCGGTATGGTTAATTTCTATGGAAAATTCAT

TAAGAATTTGAGTACTCATATTACACCACTTTACGAATTACTTAAGAAAGGTAAACATTGGCAATGGACTAAAACACATA

GATATGTGTTCAATAAAATTAAACAACTTCTATGTAGTACTGAAGTATTGACACACTTTGACATATCGCTGGAGAGCATA

GTAACATGCGACGCGAGCGCACGCGGGCTGGGGGCCGTGCTGGCACAGCGCGCGTCTGACGGCAGCGAGCGGGTTGTGGC

GTACGCATCGCGGGCGCTCACTGCCGCGGAGTTACACTACAGCCAGATACATAAAGAAGCCTTGGCTATAATTTTCGCGG

TAGACAAGTTTCATCAGTATCTTTATGGCAGAAAGTTTACACTACGTACAGATCATAAACCGCTGGTCACAATTTTTGGG

CCCCATGCAGGAATACCGAACACGGCGGCGAGTCGTTTACAGCGGTGGGCCATTAAACTATCAGCTTACGATTTTAATAT

AGAGTACATTCGATCAGATAAAAACACGGCAGATATTTTGTCTAGGTTAATTAGCACTCATAAAGAAGGGGTTATTAGTG

AAGAATTAGACACACCTGAACAAACGTATTTACACTTTGCCGCGGAAGCATTGTTATTAGATTATCAAACGTTAAAAAAA

GAAACCGTTTCGGATAGTGTATTGAGCAGAGTAGCCAGATATATAAACGATGGCTGGCCGGTCGAGGTCGAAATGAAAGA

ATTAAAACCATATTTTAACCGTAGAAAGGAATTATATATTGAGCTAGGGTGTGTAATGTGGGGACATAGGCTAGTAATTC

CTAGTACGTGTAGGAATAAAGTGATCGCTGAGCTTCATGAGAGTCACATGGGCATAGTCAAAACTAAATCGTTAGCGCGA

AGTTACGTATGGTGGCCGGGAATAGACGAGGCACTAGAGACAGCATGTTGCAGCTGCACCGTGTGCGCTGAAGTGGCGGA

CGCACCACCTGCGCATGCGCCCCGCGCCTGGCCGTGGCCAGATCGACCGTGGACCAGGATACACGTAGACTTCTTAGGAC
[truncated: 2,490,153 more chars]
